# Novel Homologs of Isopentenyl Phosphate Kinase Reveal Class-Wide Substrate Flexibility

**DOI:** 10.1002/cctc.202100595

**Published:** 2021-06-23

**Authors:** Vikas Kumar, Bryce P. Johnson, Dustin A. Dimas, Shanteri Singh

**Affiliations:** [a] Institute for Natural Products Applications and Research Technologies, Department of Chemistry and Biochemistry, University of Oklahoma, 101 Stephenson Parkway, Norman, Oklahoma 73019 (USA)

**Keywords:** alternate mevalonate pathway, biocatalysis, enzyme promiscuity, natural products, terpenoids

## Abstract

The widespread utility of isoprenoids has recently sparked interest in efficient synthesis of isoprene-diphosphate precursors. Current efforts have focused on evaluating two-step “isoprenol pathways,” which phosphorylate prenyl alcohols using promiscuous kinases/phosphatases. The convergence on isopentenyl phosphate kinases (IPKs) in these schemes has prompted further speculation about the class’s utility in synthesizing non-natural isoprenoids. However, the substrate promiscuity of IPKs in general has been largely unexplored. Towards this goal, we report the biochemical characterization of five novel IPKs from *Archaea* and the assessment of their substrate specificity using 58 alkyl-monophosphates. This study reveals the IPK-catalyzed synthesis of 38 alkyl-diphosphate analogs and discloses broad substrate specificity of IPKs. Further, to demonstrate the biocatalytic utility of IPK-generated alkyl-diphosphates, we also highlight the synthesis of alkyl-l-tryptophan derivatives using coupled IPK-prenyltransferase reactions. These results reveal IPK-catalyzed reactions are compatible with downstream isoprenoid enzymes and further support their development as biocatalytic tools for the synthesis of non-natural isoprenoids.

## Introduction

**Scheme 1.**
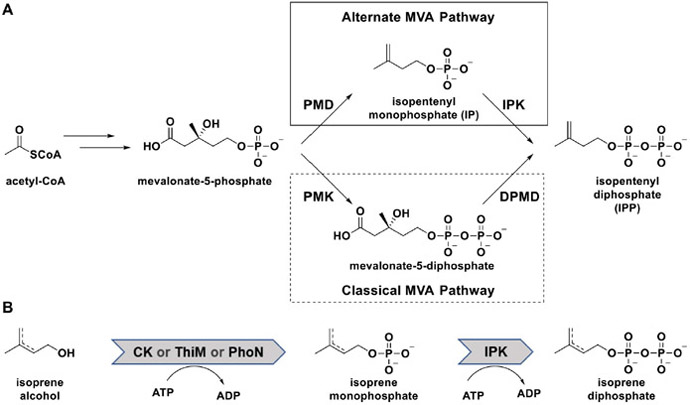
(**A**) The natural mevalonate pathway bifurcated into the classical (dotted box) and alternate (solid box) variants. (**B**) Isoprenol pathway engineered for facile synthesis of the isoprenoid precursors IPP and DMAPP. *Abbreviations:* PMD = phosphomevalonate decarboxylase, IPK = isopentenyl phosphate kinase, PMK = phosphomevalonate kinase, DPMD = diphoshomevalonate decarboxylase, CK = choline kinase from *S. cerevisiae*, ThiM = hydroxyethylthiazole kinase from *E. coli*, PhoN = acid phosphatase from *S. flexneri*.

Isoprenoids belong to one the most structurally and chemically diverse classes of natural products in existence and are utilized in a broad range of applications throughout the pharmaceutical and biotechnological industries.^[1]^ Despite their diverse forms and functions, all isoprenoids are derived from the two universal precursors isopentenyl diphosphate (IPP) and dimethylallyl diphosphate (DMAPP), which are derived from either the mevalonate (MVA, Scheme 1A) or deoxy-xylulose-5-phosphate (DXP) pathways in nature.^[2]^ The recent discovery of isopentenyl phosphate kinase (IPK) in *Archaea* has also led to the identification of an alternate MVA pathway that bifurcates from the classical pathway following the formation of mevalonate-5-phosphate (M5P).^[2a,b,d,3]^ In the classical pathway (dotted box in Scheme 1A), M5P is phosphorylated to a diphosphate before being decarboxylated and forming IPP, but the alternate pathway sees these steps reversed, with IPK performing the final phosphorylation of isopentenyl monophosphate (IP) to IPP (solid box in Scheme 1A).^[2d,3a,b,d-h]^

Due to the inherent value of these precursors, significant effort has been invested into engineering the pathways for large-scale production of valuable isoprenoids.^[1b,4]^ However, several prominent factors, including essential isoprene production for cellular function, impractical metabolic fluxes, inherent reaction complexity, limited availability of precursors, and the number of required enzymes, have made optimizing the natural pathways a challenging endeavor. To overcome these barriers, several groups have attempted to simplify the process by creating artificial “isoprenol pathways” (Scheme 1B), which leverage the promiscuity of various kinases to bypass the natural pathways and radically reduce the number of required enzymes.^[4b,5]^ The artificial pathways described thus far utilize promiscuous kinases or phosphatases (acid phosphatase PhoN from *Shigella flexneri*,^[4b]^ hydroxyethylthiazole kinase ThiM from *Escherichia coli*,^[5b]^ and choline kinase from *Saccharomyces cerevisiae*^[5a,d]^) to phosphorylate the isoprene alcohols (isopentenyl alcohol, dimethylallyl alcohol), which are subsequently diphosphorylated by an IPK to form the diphosphate precursors (Scheme 1B). Together, the two reactions have formed the basis of several platforms capable of producing both IPP and DMAPP in high quantities,^[4b,5]^ and the convergence of the schemes on IPKs for the second reaction has implied IPKs as the best candidate for the diphosphorylation step.

Thus far, the biochemical studies of the IPKs from *Methanocaldococcus jannaschii* (MJ),^[3a,6]^ *Methanothermobacter thermautotrophicus* (MTH),^[7]^ *Thermoplasma acidophilum* (THA),^[7]^ *Arabidopsis thaliana* (AT)^[8]^ and *Haloferax volcanii* (HV)^[2d]^ have revealed their high catalytic efficiencies (100–1000 mM^−1^s^−1^).^[2d,3a,6-8]^ In addition, the apo and ligand-bound crystal structures of MJ, MTH, and THA have illuminated the importance of catalytic His and Asp residues in the active site, as well as residues responsible for ATP and alkyl-substrate binding.^[6,9]^ These structural insights have further provided the basis for mutational studies resulting in variant IPKs capable of accepting 10-carbon geranyl and 15-carbon farnesyl monophosphates (GP and FP, respectively) as substrates.^[6,10]^ In addition, substrate specificity studies of THA and MTH revealed them to be promiscuous towards a small set of non-natural alkyl-monophosphate (alkyl-P) analogs.^[7,11]^ However, the breadth and depth of the substrate promiscuity of IPKs as an enzyme class remains underexplored.

To address this question, we describe herein the biochemical characterization and broad substrate specificity assessment of 5 new IPK homologs from various *Archaea* using a library of 58 synthetic alkyl-P analogs (natural and non-natural). Consistent with previous work,^[6-7,9,11b]^ our studies reveal most IPKs to have broad substrate specificities driven largely by steric factors in the active site, and our analysis of the 5 homologs has enabled the synthesis of 38 unique alkyl-diphosphate (alkyl-PP) analogs. Further, to unequivocally confirm the generation of non-natural alkyl-PPs, we coupled the activity of the IPKs to that of FgaPT2, one of several aromatic prenyltransferases (PTs) known to utilize non-natural alkyl-PPs as substrates.^[12]^ Our results with a representative set of alkyl-Ps indeed confirm the generation and utilization of alkyl-PPs by IPKs and FgaPT2, respectively. Overall, the study illuminates the hypothesized promiscuity of IPKs as an enzyme class while simultaneously providing a toolbox of enzymes for the synthesis of non-natural alkyl-PPs, which may have further utility in generating non-natural isoprenoids.

## Results and Discussion

### Synthesis of Alkyl-P Library

**Figure 1.**
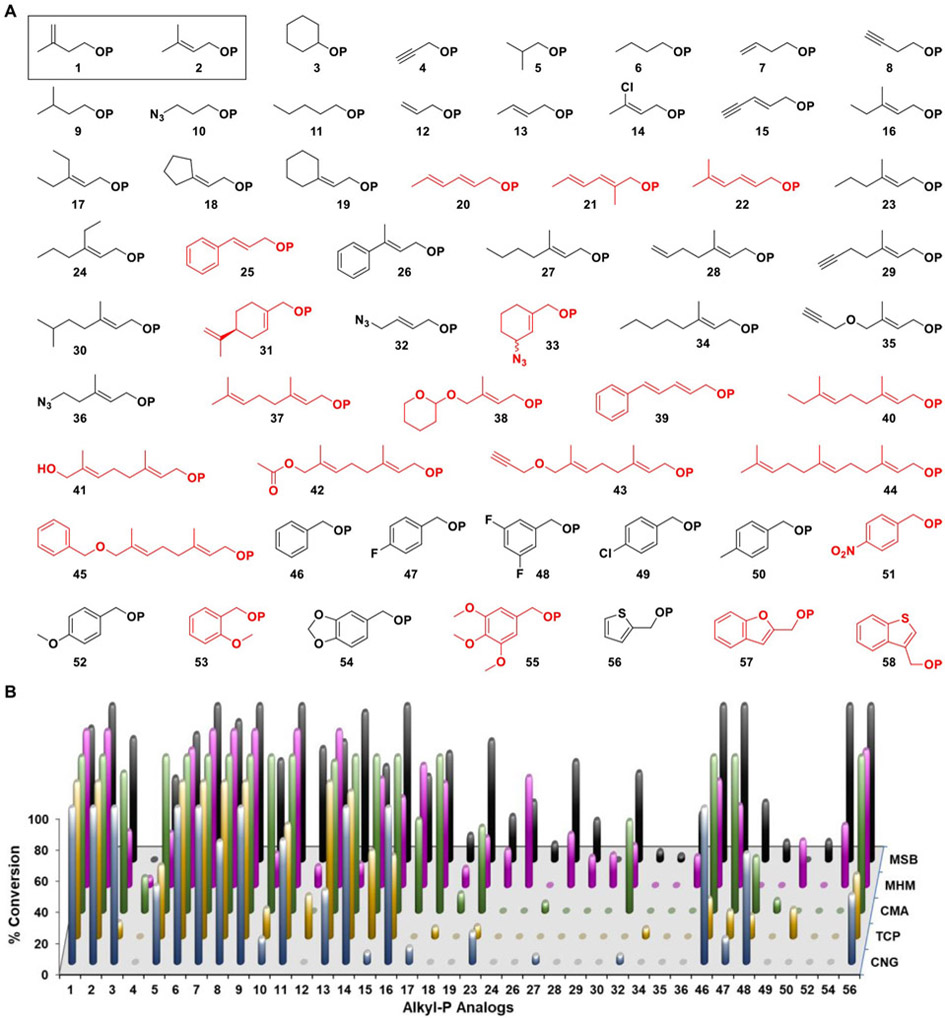
(**A**) The library of alkyl-Ps utilized in this study. The natural substrate IP and its isomer DMAP are shown in a solid box. Alkyl-P analogs that did not show turnover with any IPK are shown in red. (**B**) Screening of five novel IPK homologs against the library of alkyl-Ps. Conversions were calculated by measuring the absorbance at 340 nm of each enzymatic reaction just before and 30 min after the addition of IPK at 37 °C and comparing it to a positive control (n = 2). Appropriate controls were conducted to account for any ATPase activity. Each reaction consisted of 2 U PK, 2 U LDH, 0.6 mM NADH, 1.5 mM PEP, 2 mM ATP, and 4 μg of IPK incubated in a buffered solution (25 mM Tris pH 7.8, 5 mM MgCl_2_). All positive reactions were verified using HRMS.

To begin our investigation, we synthesized a library of 58 alkyl-P analogs (Figure 1A) that included the natural substrate (1), its dimethylallyl isomer (**2**), and seven previously identified non-natural substrates of THA/MTH (**6–8**, **12**, **16**, **25**, **37**).^[6,10-11,13]^ The syntheses were carried out using previously established protocols (see Supplementary Information),^[12,14]^ and the resulting library was divided into four categories based on generalized chemical structures: non-allylic (**1**, **3**–**11**), allylic (**2**, **12**–**45**), benzylic (**46**–**55**), and heterocyclic (**56**–**58**). While all groups were designed to probe substrate promiscuity, analogs **2** and **12**–**58** were synthesized with the utility of their diphosphate products with downstream isoprenoid enzymes (such as PTs) in mind.^[12,14]^ Additionally, the alkyl-P analogs were designed to display unique steric and electronic characteristics through variation of their chain length, methyl-substituents, heteroatoms, *π* systems, and synthetic handles (alkynes, azides, terminal alkenes, dienes).

### Initial Screening of IPKs

A set of five novel IPKs were identified from various *Archaea* [*Candidatus methanomethylophilus alvus* (CMA), *Candidatus Nitrososphaera gargensis* Ga9.2 (CNG), *Methanohalophilus mahii* DSM 5219 (MHM), *Methanosarcina barkeri* 3 (MSB), and *Thermococcus paralvinellae* (TCP)] using a BLAST search against a template IPK sequence from MJ.^[3a,6]^ Recombinant IPK constructs were then overexpressed in *Escherichia coli* Rosetta2 cells transformed with codon-optimized synthetic genes in pET28a vectors, and the resulting N-His_6_-fusion proteins were purified using Ni-NTA chromatography. Assessment of IPK activities with the library of alkyl-P analogs (Figure 1A) was performed using the pyruvate kinase-lactate dehydrogenase (PK-LDH) assay under standardized conditions (150-μL reaction, 25 mM Tris pH 7.8, 50 mM KCl, 5 mM MgCl_2_, 4 μg IPK, 2 U PK, 2 U LDH, 0.6 mM NADH, 1.5 mM PEP, 2 mM ATP, 1 mM alkyl-P; incubated for 30 min at 37°C Figure 1B, Table S6), and the formation of putative alkyl-PP analogs was subsequently confirmed by high-resolution mass spectrometry (HRMS) for all positive reactions (Table S7).

Cumulative analysis revealed 38 of the 58 tested alkyl-P analogs acted as substrates across the 5 IPKs, with MSB, MHM, CMA, TCP, and CNG accepting 35, 32, 26, 23, and 22 analogs, respectively. In general, the non-allylic and allylic alkyl-Ps bearing 4–6 atoms in their linear chain were good substrates (> 50% conversion), those with <4 or 7–9 atoms displayed moderate conversion (10–50%), and those with ≥10 atoms were poor substrates (<10%) or not taken at all. Interestingly, dienes (**20**–**22**) did not serve as substrates for any IPKs even with <10-atoms chain lengths, and the substrate scope of IPKs with benzylic and heterocyclic alkyl-Ps was generally limited to the core benzylic scaffold with less bulky substituents or heterocyclic analogs of smaller size (**56**). However, the benzodioxole analog (**54**) acted as a substrate for MHM and MSB, and similar methoxy-substituted analogs were accepted to either a small extent (**52**) or not at all (**53** and **55**) by the IPKs. In cases where direct comparisons could be made within the same category, increasing the size of the alkyl chain correlated with a reduction in turnover (e.g., **2** > **16**> **23**; **8** > **15** > **29** > **35**; **10** > **32** > **36**; **46** >**47** >**48** > **50**). Collectively, these analyses indicated steric interactions were the major contributing factors to the substrate scope of the IPKs, with MSB and MHM hypothesized to contain the least constrained active sites based on their broad substrate specificities.

### Kinetic Studies

In addition to their generalized ability to utilize non-natural substrates, we also wanted to understand the enzymes’ catalytic efficiencies (k_cat_/K_M_) with the alkyl-P analogs as an initial gauge of their biosynthetic utility. As such, kinetic analyses were performed with a representative set of substrate/IPK pairs (Table 1) using the PK-LDH assay (1 mM **1** and 0.01–2 mM ATP or 1 mM ATP and 0.025–6 mM alkyl-P analog in 25 mM Tris pH 7.8, 5 mM MgCl_2_, 2 U PK, 2 U LDH, 0.6 mM NADH, 1 mM PEP at 37°C). The K_M_ values of all five homologs with ATP and the natural substrate IP (**1**) were consistent with those previously reported for IPKs.^[2d,3a,6-8]^ However, while the catalytic efficiencies of CMA, MSB, and MHM with the natural substrate IP (**1**) were consistent with those of previously characterized IPKs (100–1000 mM^−1^ s^−1^),^[2d,3a,6-8]^ the values for TCP and CNG were an order of magnitude lower (18 and 28 mM^−1^ s^−1^, respectively) mainly due to low k_cat_ values. Furthermore, while DMAP (**2**) displayed similar efficiencies with MSB/MHM compared to IP, it decreased 3-fold (for CMA/TCP) and 8-fold (for CNG) due to higher K_M_ values. Interestingly, the chloro-substituted DMAP analog (**14**) displayed similar efficiency as IP for TCP/CNG, a 3-fold lower value for CMA/MSB, and a 7-fold decrease for MHM, while removing the methyl-group of DMAP (**13**) and cyclizing C4 and C5 with an additional carbon (**18**) decreased efficiencies by 7–50 fold and 30–600 fold, respectively, compared to **1**. The alkyne (**15**) and azide (**32**) installations decreased efficiencies significantly across the board compared to **1** and **2**, and though the addition of carbons to the alkyl-chain of **2** (**23** and **28**) caused decreases in catalytic efficiency, it was not necessarily due to large losses in K_M_. The most interesting cases of this phenomenon were with MHM and MSB, which appeared to arise from a notable combination of increased K_M_ and decreased k_cat_. Additionally, the binding efficiencies for **23** and **28** resembled values obtained for the butene analog **13** with either similar or decreased k_cat_ values.

As for the benzylic and heterocyclic analogs, steric factors contributed prominently, although differences in electron density also played a more pronounced role in determining catalytic efficiency compared to the allylic alkyl-Ps. The K_M_ values for the unsubstituted benzyl analog **46** were similar to those of **2**, but the k_cat_ values fell by more than an order of magnitude in all cases except CNG (no change in k_cat_, increased K_M_). Such similar K_M_ values between **2** and **46** could be related to their similar carbon chain length and planarity, but the effect of these factors appeared to lessen with the sequential addition of fluorine atoms (**47**, **48**), which degraded the homologs’ specificity (**48** <**47** <**46**) mostly by increasing K_M_ values. Considering the similar sizes of fluorine and hydrogen, this was likely caused by slightly unfavorable electronic interactions between the electron-rich substituent and the hydrophobic binding pockets. Chlorination at the *para*-position (**49**) had an even more dramatic effect on the enzymes’ activity with benzyl analogs (**49**<**47** <**46**). Three IPKs (CNG, MHM, and TCP) displayed no detectable activity with **49**, while the catalytic efficiencies of CMA and MSB were reduced by 3–4 orders of magnitude. These reductions in activity were likely caused by the greater steric bulk and electron density of the chlorine atom compared to fluorine, which would exacerbate the unfavorable interactions hypothesized with **48**. Adding other bulky substituents to the benzyl group appeared to further prohibit them from acting as substrates, except in the case of the benzodioxole group (**54**). Interestingly, this analog was accepted by MSB with catalytic efficiency an order of magnitude lower than **46**, mostly due to higher K_M_ values. This phenomenon could be related to the high conformational constraint imposed by the dioxole ring (compared to methoxy substituents) coupled with the increased binding pocket space hypothesized for MSB. Finally, the heterocyclic thiophene ring of **56** displayed 2-fold higher k_cat_ as well as K_M_ values compared to **46**, resulting in similar catalytic efficiency to the core benzylic scaffold. This could result from the reduced steric bulk of the thiophene ring being compensated by the increased electron density of the sulfur atom. Nevertheless, the demonstrated ability of the IPKs to utilize benzylic and heterocyclic analogs at all implied the class may be suitable for drug discovery purposes.^[15]^

In general, the lower catalytic efficiencies of the non-natural analogs compared to IP was due to a combination of decreased k_cat_ and increased K_M_ values, which correlated to the overall size of the substrate. Based on these values, TCP and CNG were the worst catalysts for all substrates; CMA was the best catalyst for **1**, **2**, **13**, and **14**; and MSB served as a suitable catalyst for analogs with longer allylic chains (**23** and **28**) and alkyne (**15**), azide (**32**), benzylic (**46**--**49**, **54**), and heterocyclic (**56**) substituents. The comparative analysis with natural and non-natural analogs indicated that the IPK with the highest catalytic efficiency with the natural substrate (CMA) may not the best catalyst for diverse non-natural substrates due to higher K_M_ values compared to homologs with lower efficiency for the natural reaction (MSB and MHM). Alongside the turnover data, this suggests MHM and MSB contain IP binding pockets with additional space to accommodate alkyl-chain variations.

### Sequence Alignment

**Figure 2.**
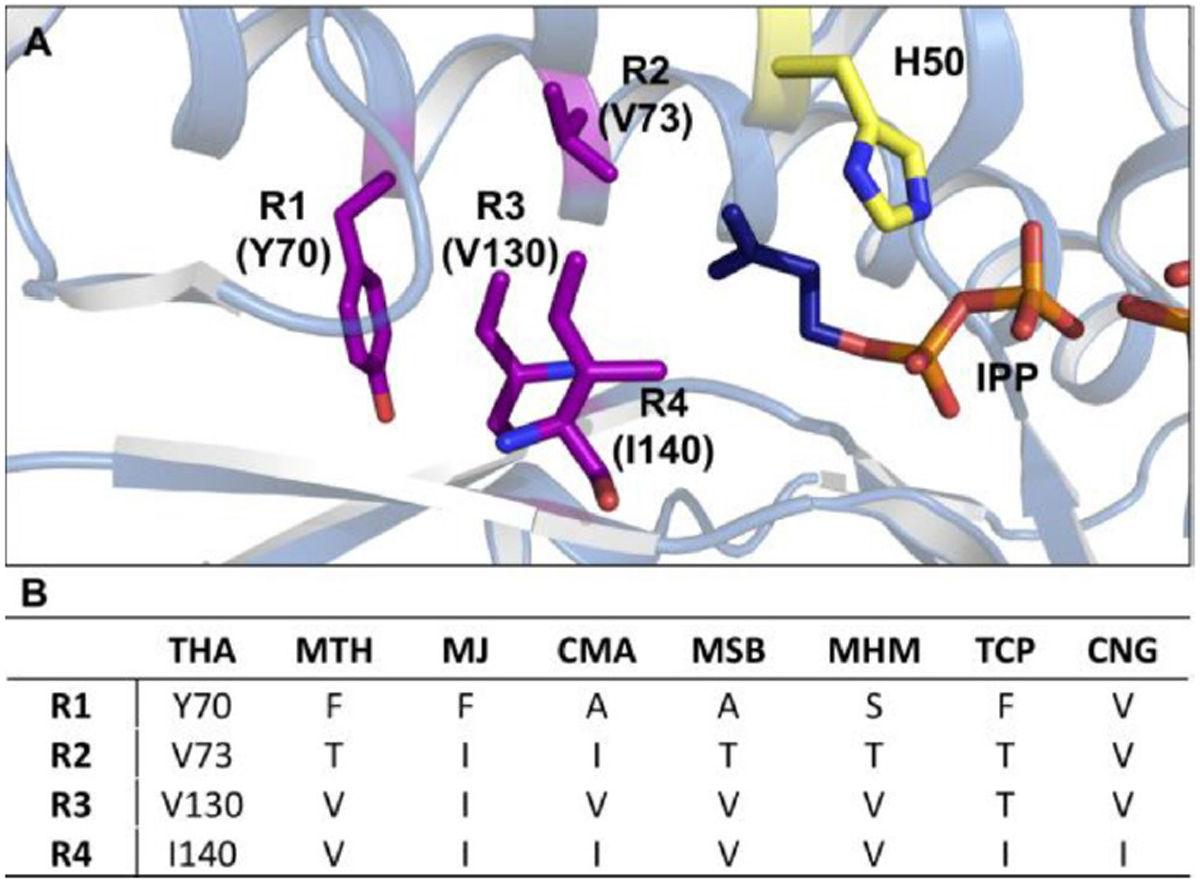
(**A**) Active site structure of THA bound to IPP (dark blue) and ADP (PDB: 3LL5).^[9]^ The catalytic His residue is shown in yellow, and the four residues forming the alkyl-chain binding pocket are shown in purple. (**B**) Identities of active site residues R1-R4 in all biochemically characterized IPKs as determined by sequence alignment using PROMALS3D (see Figure S115).^[16]^

To understand the differences in binding pocket architecture, we carried out a sequence alignment of the five IPKs from the current study with homologs whose crystal structures^[6,9]^ have been solved previously (Figure S115). Among the 8 sequences, 25–38% sequence identity was observed between any two IPKs, except in the case of MSB and MHM (51 %). Upon closer inspection, alignment of the enzymes’ alkyl-binding sites revealed major differences in 4 previously identified residues corresponding to Y70, V73, V130 and I140 in THA (mapped as R1-R4, respectively, in Figure 2). In most cases, the 4 residues featured hydrophobic side chains (R1 = Y/F/A, R2 = I/V/T, R3 = I/V/T, R4 = I/V).^[6,9]^ However, 2 of the 4 amino acid residues displayed noticeably smaller side chains in MSB, MHM and TCP (R1 = A/S, R2 = T for MSB/MHM; R2 = R3 = T for TCP), while CMA and CNG had only one or no smaller side chains in any of the 4 positions. The corresponding increase in binding pocket space may thus explain the lower K_M_ values for MSB, MHM, and TCP with most of the non-natural alkyl-P analogs compared to CMA and CNG. Furthermore, a comparison of kinetic parameters between CMA, MSB, MHM, and TCP suggests the presence of smaller side chains at R1 and R2 is more beneficial for promiscuity than when similar residues are found at R3. Taken together, these observations suggest that smaller side chains at specific positions in the binding pocket can accommodate substantial changes to the structure of the natural substrate.

More specifically, we hypothesize the substitution of bulkier residues by A/S and T at R1 and R2, respectively, create larger and more flexible binding pockets in MHM and MSB that can accommodate longer alkyl chains. This hypothesis is supported by both the initial screening data (Figure 1B) and the two enzymes’ specificities with analogs **15**, **18**, **23**, **28**, and **32** (Table 1). Although specificity does begin to decrease 15–30 fold with the longer chains of **28** and **32**, the closeness in value of k_cat_/K_M_ between **15**, **18**, and **23** suggests the two enzymes maintain reasonable activity with linear alkyl-chains of 6 atoms or less. Furthermore, in the case of MSB, the trends in K_M_ and k_cat_ showed **23** to bind more efficiently and be phosphorylated at a higher rate than either **15** or **18**, which cements its preference for longer-chain non-natural alkyl-Ps. In terms of biocatalytic utility, both MHM and MSB have demonstrated the potential for synthesizing longer-chain precursors for non-natural isoprenoid scaffolds. Overall, the good catalytic efficiencies of the IPKs with most of the tested alkyl-P analogs (≥ 1 mM^−1^ s^−1^) points to a generalized promiscuity within the enzyme class that can be harvested as a biocatalytic tool.

### IPK-PT Coupled Platform

**Scheme 2.**
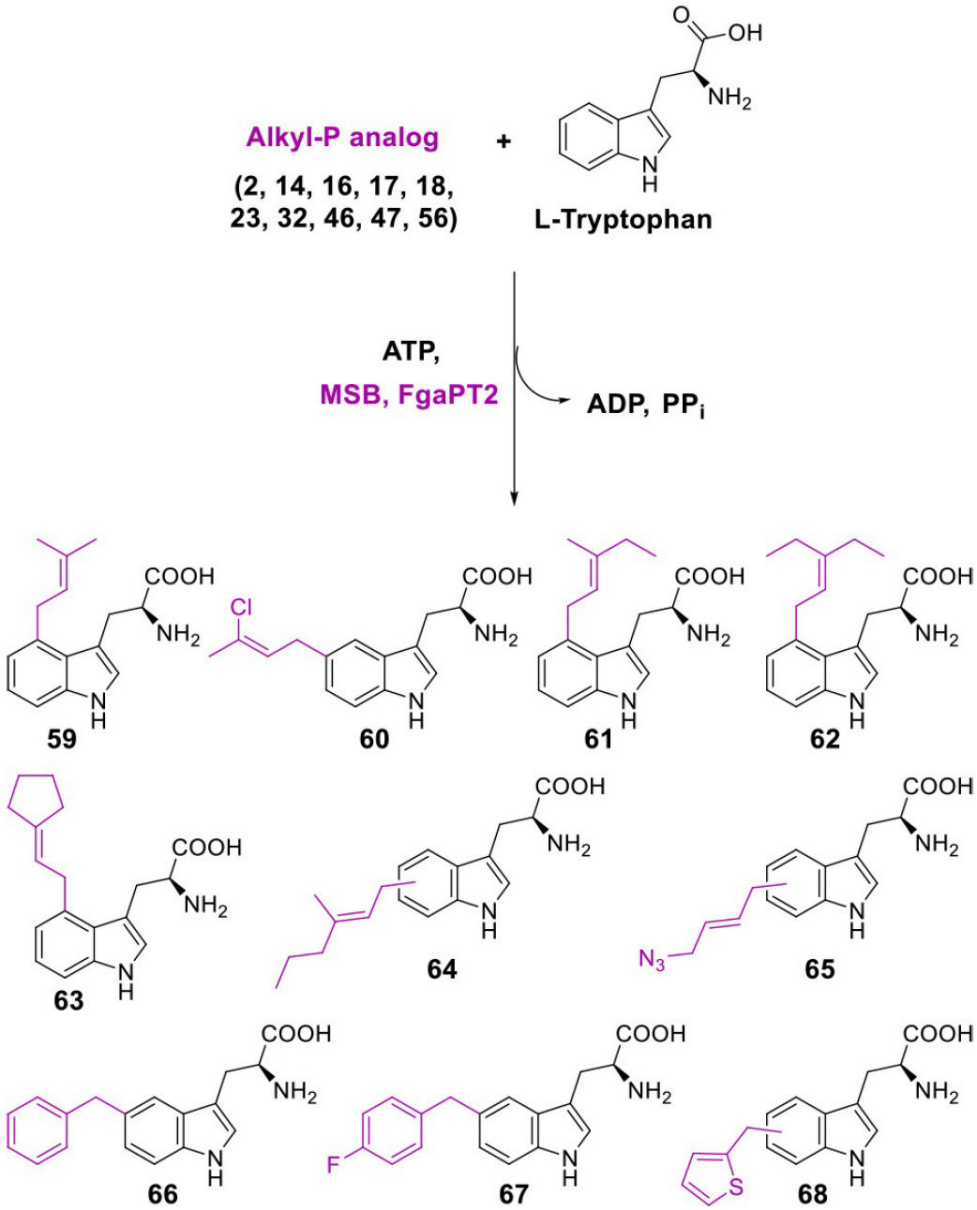
MSB-FgaPT2 coupled platform for the generation of alkylated l-Trp analogs. The formation of products was observed using RP-HPLC (Figure S159) and confirmed by HRMS (Table S8). The regiospecificity of **59**–**63**, **66**, and **67** has been determined previously for FgaPT2-catalyzed reactions with synthetic alkyl-PP analogs.^[12]^

As an initial test of this utility, we decided to couple the IPKs’ ability to generate alkyl-PPs to an isoprenoid enzyme with compatible substrate requirements in a one-pot synthesis. Considering the demonstrated promiscuity of PTs towards non-natural allylic and benzylic alkyl-PPs,^[12,14]^ we chose to utilize one such catalyst as the auxiliary enzyme. Specifically, the l-Trp C4-PT FgaPT2 from fumigaclavine biosynthesis^[17]^ was selected for its abilities to utilize chemically diverse alkyl-PPs (both allylic and benzylic) as donor substrates.^[12]^ The resulting IPK-FgaPT2 coupled system employed standard assay conditions (1.5 mM alkyl-P analog, 2 mM ATP, 1 mM l-Trp, 5 μM MSB, 20 μM FgaPT2, 25 mM Tris pH 7.8, 5 mM MgCl_2_, 50 mM KCl; incubated for 16 h at 37 °C) and included select allylic/benzylic/heterocylic alkyl-P analogs (non-allylic alkyl-PPs are not substrates of PTs)^[12,14]^ that afforded ≥50% turnover with MSB under standard conditions (Scheme 2). The resulting HPLC analysis revealed incubation of the coupled system with the 10 selected alkyl-P analogs led to good yields of alkylated l-Trp analogs in all reactions (≥75%, Scheme 2, Figure S159), and the regiospecificity of 7 out of 10 products was assigned based on previous work.^[12,14]^ Importantly, this is the first report of an IPK-coupled, PT-catalyzed generation of alkylated l-Trp derivatives using non-natural alkyl-P starting materials.

## Conclusion

In summary, the biochemical characterization of 5 new IPKs from *Archaea* has revealed that the enzymes share a class-wide promiscuity toward non-natural alkyl-Ps and display catalytic efficiencies across two orders of magnitude with the natural substrate. Subsequent analysis of active-site sequence alignments points to residues with smaller side chains at key positions in the alkyl-binding site as being responsible for broader substrate specificity in the most promiscuous IPKs. In addition, the work herein has demonstrated that the IPK-catalyzed synthesis of non-natural alkyl-PP analogs is directly compatible with downstream alkyl-PP-utilizing enzymes (specifically PTs). Additional engineering studies will be needed to understand the role of different residues in the optimal binding of non-natural analogs, but the data presented here implies the fully optimized IPKs could be utilized as a biocatalytic tool in the two-enzyme isoprenol pathways or other metabolic pathways for the synthesis of non-natural isoprenoids. Furthermore, the IPKs’ activity with diverse allylic and benzylic analogs, as well as analogs bearing synthetic handles, speaks to the class’s overall biosynthetic potential, both in terms of unique scaffold generation and late-stage diversification of existing aromatics. Thus, the current study continues our collective advance towards the efficient synthesis of novel isoprenoids.

## Experimental Section

### General Materials

Unless otherwise stated, all chemicals and reagents were purchased from Sigma-Aldrich (St. Louis, MO, USA), Acros Organics (Fair Lawn, NJ, USA), Alfa-Aesar (Ward Hill, MA, USA), TCI (Portland, OR, USA), Ambeed (Arlington Heights, IL, USA), or AK Scientific (Union City, CA, USA) and were reagent grade or better. PD-10 and Ni-NTA super-flow columns were purchased from GE Healthcare (Piscat-away, NJ). Farnesyl monophosphate (**44**) was obtained from Isoprenoids LC (Tampa, FL, USA).

### Synthesis of Alkyl-P Analogs

Detailed methods for the synthesis of alkyl-P analogs are summarized in the Supporting Information.

### IPK Homolog Expression and Purification

Using a BLAST search, hypothetical IPKs were identified in the genomes of *Candidatus methanomethylophilus alvus, Candidatus Nitrososphaera gargensis* Ga9.2, *Methanohalophilus mahii* DSM 5219, *Methanosarcina barkeri* 3, and *Thermococcus paralvinellae* using the input sequence of MJ. The corresponding genes were then synthesized and codon-optimized for expression in *E. coli* by GenScript (Supplementary Information Tables S3-S4). For the purposes of expression and purification, the synthetic genes were inserted into the pET28a vector between the NdeI and EcoRI sites using restriction digest, ligating a His_6_-tag to the N-terminus of the expressed proteins (Supplementary Information Table S5). The recombinant plasmids were transformed into *E. coli* Rosetta2 cells using heat shock, and the transformed cultures were then plated on Luria-Bertani (LB) agar supplemented with 50 μg mL^−1^ kanamycin (KAN) to grow at 37°C. Resulting colonies were individually sequenced, and those bearing the recombinant plasmids were used to make LB broth cultures (supplemented with 50 μg mL^−1^ KAN) grown at 37°C and stored long-term in 25% glycerol at −80°C.

Expression protocols began with the inoculation of 5-mL broth cultures containing 50 μg mL^−1^ KAN from glycerol stocks, which were subsequently incubated overnight at 37°C and 200 rpm. “Overnight” cultures were then utilized to inoculate 1-L broth cultures containing 50 μg mL^−1^ KAN grown at 37°C and 200 rpm. When the OD_600_ each reached 0.6–0.8 (~4 h), the cultures were induced with 0.5 mM IPTG (final concentration) and incubated at 20°C and 200 rpm for 16–20 hr. Cells were then separated from broth through centrifugation (4000 rpm, 4°C for 30 min), resuspended in lysis buffer (50 mM NaH_2_PO_4_ pH 8.0, 300 mM NaCl, 10 mM imidazole), and frozen at −80 °C.

Purification began with three complete cycles of freezing at −80 °C and thawing at room temperature. Cultures were then lysed through a combination of lysozyme treatment (30 min on ice) and sonication (40 min total, in cycles of 10-s pulses and 25-s breaks). Centrifugation was used to separate the soluble fraction from the insoluble cell debris (16000 rpm, 10°C, 1 h), and the supernatant was subsequently purified using Ni-NTA chromatography. Fractions containing the purified protein were pooled and concentrated in an Amicon centrifugal concentrator (Merck Millipore, Burlington, MA, USA), and repeated cycles of dilution and re-concentration in storage buffer (25 mM Tris pH 8.0, 50 mM KCl, 20% v/v glycerol) removed a majority of the imidazole (final concentration <1 mM). The purity of the concentrated protein was checked by SDS-PAGE, and pure proteins were drop-frozen in liquid N_2_ and stored at −80 °C.

### High-Throughput Screening of Alkyl-P Analogs

Purified IPK variants were screened against the library of alkyl-Ps using the pyruvate kinase-lactate dehydrogenase (PK-LDH) assay, which has been utilized previously to characterize similar proteins.^[5b]^ Briefly, reactions were conducted in a high-throughput (HT) manner using 96-well plates. Each well contained a 150-μL reaction mixture composed of 4 μg IPK, 2 U PK, 2 U LDH, 0.6 mM NADH, 1.5 mM PEP, 2 mM ATP, and 1 mM alkyl-P in buffer (25 mM Tris pH 7.8, 5 mM MgCl_2_). Before the IPK was added (10 μL), an absorbance reading at 340 nm was conducted to establish each well’s initial A_340_ before any NADH was consumed. After the addition of IPK, the absorbance at 340 nm was monitored every 30 s for 1 h at 37°C, and the difference between the final A_340_ at 1 h and the initial A_340_ before the addition of IPK was used to calculate the percentage turnover of NADH. All positive reactions were subsequently confirmed using HRMS as described in the Supplementary Information. Additionally, two control reactions were conducted: one without any alkyl-P to establish the baseline, and one using ADP instead of ATP to identify full conversion.

### Kinetic Studies of IPK Homologs

From the initial screening data, pairs of enzymes and substrates were selected for kinetic characterization using the PK-LDH assay. For studies of the alkyl-P analogs as substrates, each well contained a 150-μL buffered reaction mixture (25 mM Tris pH 7.8, 5 mM MgCl_2_) composed of 2 U PK, 2 U LDH, 0.6 mM NADH, 1 mM PEP, 2 mM ATP, 0.025–6 mM alkyl-P, and a concentration of IPK suitable to observe ≤20% conversion of NADH in 30 min. The addition of IPK initiated the reaction, and A_340_ was measured every 30 s for 30 min at 37°C. For kinetic studies of ATP as a substrate, the same conditions were used except *1)* IP (**1**) was used as the phosphoryl acceptor for all enzymes at a constant concentration of 1 mM, and *2)* the concentration of ATP was varied in the range 0.01–2 mM. Initial rates were determined from the slope of the line of best fit for the time period in each reaction giving ~10% conversion after excluding the first 3 min (considered an equilibration period). Slopes were corrected for the degradation of NADH using the slope of a control reaction containing no alkyl-P. The kinetic constants k_cat_ and K_M_ and their associated errors were determined by inputting initial rate data for each substrate concentration into GraphPad Prism (GraphPad Software, San Diego, CA, USA) and conducting a nonlinear regression. Values for the specificity constant k_cat_/K_M_ were obtained by performing the calculation in Microsoft Excel (Microsoft Corporation, Redmond, WA, USA) and propagating the errors.

### MSB-FgaPT2 Coupled Assay

Recombinant FgaPT2 was purified as described previously.^[12]^ MSB-FgaPT2 reactions were conducted in vitro (40 μL) using 2 mM ATP, 1.5 mM alkyl-P analog (**2**, **14**, **16**, **17**, **18**, **23**, **32**, **46**, **47**, or **48**), 1 mM l-Trp, 5 μM purified MSB, and 20 μM purified FgaPT2 in a buffered solution (25 mM Tris buffer pH 7.5, 5 mM MgCl_2_, 50 mM KCl). Reactions were incubated at 37 °C for 16 h, after which they were quenched with an equal volume of cold methanol. The quenched reactions were then centrifuged (10,000×*g* for 15 min) to remove the precipitated protein, and product formation was monitored by analytical reverse-phase (RP)-HPLC. The method employed a Gemini-NX, C-18 (5 μm, 4.6 mm×250 mm) column (Phenomenex, Torrance, California, USA) [gradient of 5% B to 15% B over 15 min, 15% B to 50% B over 13 min, 50% B to 100% B over 5 min, 100% B to 5% B over 0.1 min, 5% B for 7 min (A = ddH_2_O with 0.1 % TFA; B = acetonitrile); flow rate = 1 mL min^™1^ A_254_], and reactions were monitored by the retention time difference between starting material and product. Negative controls included reactions in the absence of enzyme or alkyl-P analog. All putative products were subsequently confirmed by HRMS with positive (+) and/or negative (−) mode (see General Methods and Table S8).

## Supplementary Material

Supplementary Information

## Figures and Tables

**Table 1.** Pseudo first order kinetic constants for IPKs using either 1 mM 1 and 0.01–2 mM ATP or 1 mM ATP and 0.025–6 mM alkyl-P analog in 25 mM Tris pH 7.8, 5 mM MgCl_2_ at 37 °C.

Substrate	*k_cat_*[s^−1^]					*K_M_* [mM]					*k_cat_/K_M_* [mM^−1^ s^−1^]				
	*CMA*	*MSB*	*MHM*	*TCP*	*CNG*	*CMA*	*MSB*	*MHM*	*TCP*	*CNG*	*CMA*	*MSB*	*MHM*	*TCP*	*CNG*
ATP	12.3±0.7	9.1±0.4	6.3±0.3	0.59±0.03	0.81 ±0.06	0.036 ± 0.009	0.09 ±0.01	0.10 ±0.03	0.015 ±0.003	0.021 ±0.005	340	100	63	39	39
1	19±1	10.3±0.4	5.2±0.4	0.62±0.03	1.12±0.05	0.018±0.005	0.027±0.005	0.05±0.01	0.022±0.006	0.061±0.009	1000	380	100	28	18
2	24±1	10.1±0.4	4.1±0.1	0.63±0.03	0.87±0.07	0.07±0.01	0.032±0.007	0.036±0.004	0.07±0.01	0.39±0.09	360	310	120	9	2.2
13	8.0±0.7	4.2±0.2	1.8±0.1	0.46±0.03	0.25±0.02	0.13±0.02	0.10±0.01	0.12±0.02	0.31±0.05	0.8±0.2	63	40	15	15	0.33
14	17.0±0.9	11±1	0.62±0.03	0.69±0.04	2.9±0.2	0.06±0.01	0.09±0.02	0.05±0.01	0.03±0.01	0.15 ±0.04	280	110	14	24	19
15	3.3±0.6	1.78±0.08	0.076±0.008	ND	ND	1.5±0.5	0.25±0.03	0.2±0.1	ND	ND	2.2	7.1	0.4	ND	ND
18	1.22±0.08	2.7±0.2	0.25±0.02	0.020±0.001	0.20±0.06	0.32±0.06	0.23±0.04	0.12±0.03	0.51±0.07	7±2	3.7	12	2.0	0.039	0.03
23	0.41±0.04	4.9±0.3	0.85±0.08	0.011±0.001	0.034±0.002	0.9±0.2	0.12±0.02	0.22±0.04	0.6±0.2	0.51±0.05	0.5	41	4.0	0.018	0.065
28	ND	0.23±0.01	0.62±0.03	ND	ND	ND	0.16±0.03	0.13±0.02	ND	ND	ND	1.5	4.7	ND	ND
32	1.6±0.4	0.42±0.03	0.062±0.004	ND	ND	4±1	0.25±0.05	0.47±0.08	ND	ND	0.4	1.6	0.14	ND	ND
46	2.0±0.1	1.23±0.07	0.139±0.008	0.039±0.002	0.83±0.07	0.09±0.01	0.045±0.008	0.07±0.01	0.069±0.010	1.2±0.2	21	27	1.9	0.56	0.7
47	0.93±0.03	1.41±0.04	0.138±0.006	0.0312±0.0009	0.27±0.02	0.12±0.01	0.074±0.007	0.19±0.03	0.055±0.006	1.9±0.2	7.8	19	0.69	0.57	0.14
48	0.26±0.02	0.195±0.007	ND	0.019±0.001	1.2±02	0.28±0.05	0.33±0.03	ND	0.08±0.02	3.3±0.7	0.9	0.58	ND	0.23	0.33
49	0.027±0.002	0.052±0.004	ND	ND	ND	0.27±0.05	0.10±0.03	ND	ND	ND	0.10	0.5	ND	ND	ND
54	ND	0.82±0.02	ND	ND	ND	ND	0.33±0.02	ND	ND	ND	ND	2.5	ND	ND	ND
56	4.3±0.4	2.4±0.1	0.40±0.04	0.140±0.004	0.17±0.02	0.34±0.08	0.15±0.02	0.20±0.06	0.13±0.02	0.5±0.1	13	16	2.0	1.1	0.4
