# Supplementary material for: Novel Homologs of Isopentenyl Phosphate Kinase Reveal Class‐Wide Substrate Flexibility
Source: ChemCatChem. 2021 Jul 12;13(17):3781–8. doi: 10.1002/cctc.202100595 (PMC8500459; doi:10.1002/cctc.202100595)
Supplement: Supplementary file 1 — Supporting Information [file CCTC-13-3781-s001.pdf]

ChemCatChem

Supporting Information

Novel Homologs of Isopentenyl Phosphate Kinase Reveal Class-Wide Substrate Flexibility

Vikas Kumar[+], Bryce P. Johnson[+], Dustin A. Dimas, and Shanteri Singh*

Author Contributions

V.K. Data curation:Lead; Formal analysis:Equal; Investigation:Equal; Methodology:Lead
B.J. Data curation:Supporting; Formal analysis:Equal; Investigation:Equal; Methodology:Equal; Writing – original draft:Lead; Writing – review & editing:Lead
D.D. Formal analysis:Equal; Methodology:Equal; Validation:Equal; Writing – original draft:Supporting
S.S. Conceptualization:Lead; Formal analysis:Supporting; Funding acquisition:Lead; Investigation:Equal; Supervision:Lead; Writing – original draft:Equal; Writing – review & editing:Equal

Supporting Information

Table of Contents

1. General Methods 2
2. General Procedure for the Synthesis of Alkyl-P Analogs 2-7
    2.1. Protection and Deprotection of Alcohols....................2
    2.2. Synthesis of α,β-Unsaturated Esters 3-4
    2.3. Synthesis of α,β-Unsaturated Alcohols.................... 4-5
    2.4. Synthesis of Alkynes 5
    2.5. Synthesis of Azides 5-6
    2.6. Synthesis of Geraniol Derivatives 6-7
    2.7. Phosphorylation....................7
3. **Scheme S1.** General Methods for the Synthesis of Alkyl-Esters and Alkyl-Alcohols....................8
4. **Scheme S2.** General Methods for the Synthesis of Alkyl-Monophosphates....................9
5. **Table S1.** Summary of HRMS Data for Synthetic Alkyl-Ps **1-36** 10
6. **Table S2.** Summary of HRMS Data for Synthetic Alkyl-Ps **37-58** 11
7. NMR and High-Resolution Mass Spectra of Synthetic Alkyl-Ps.................... 12-125
8. **Table S3.** Codon-Optimized Genes (CMA, CNG) Used in This Study....................126
9. **Table S4.** Codon-Optimized Genes (MHM, MSB, TCP) Used in This Study....................127
10. **Table S5.** Amino Acid Sequences of the IPK Homologs Utilized in This Study 128
11. **Figure S153.** Sequence Alignment and Identity Matrix of IPK Homologs 129
12. **Table S6.** NADH Conversion Data for IPK Homologs 130
13. **Table S7.** HRMS Confirmation of IPK-Catalyzed Reactions....................131
14. High-Resolution Mass Spectra of IPK-Catalyzed Reactions 132-169
15. **Figure S154.** Michaelis-Menten Curves for CMA 170
16. **Figure S155.** Michaelis-Menten Curves for CNG....................171
17. **Figure S156.** Michaelis-Menten Curves for MHM 172
18. **Figure S157.** Michaelis-Menten Curves for MSB....................173
19. **Figure S158.** Michaelis-Menten Curves for TCP 174
20. **Figure S159.** HPLC Traces of MSB-FgaPT2 Coupled Reactions....................175
21. **Table S8.** HRMS Confirmation of MSB-FgaPT2 Coupled Reactions....................176
22. High-Resolution Mass Spectra of MSB-FgaPT2 Coupled Reactions 177-186
23. ^{1}H-NMR Spectra of Alkyl-Alcohol Intermediates 187-217
24. References 218

1. General Methods

All synthetic reactions were conducted in oven-dried glassware under a nitrogen atmosphere with anhydrous solvents, unless otherwise noted. Reactions were monitored by thin-layer chromatography (TLC) (EMD Millipore Corp, Billerica, MA, USA), and visualization was accomplished with UV light (254 nm) followed by 1) staining with phosphomolybdic acid solution or anisaldehyde solution and heating, and 2) exposure to iodine and cerium ammonium molybdate with no heating. Flash column chromatography was performed using ACS-grade solvents and silica gel (SiliCycle Inc, P60, particle size 40-63μm). High-resolution mass spectrometric (HRMS) data and liquid chromatography mass spectrometric (LCMS) were obtained on Agilent6545-QTOF W/1290 HPLC mass spectrometer at the University of Oklahoma, Department of Chemistry and Biochemistry. NMR spectra were obtained on Varian VNMRS 300, 400 MHz instruments at the NMR facility of the Department of Chemistry and Biochemistry of University of Oklahoma using 99.8% D2O (Cambridge Isotope Laboratories, MA, USA). ^{1}H and ^{31}P chemical shifts were referenced to internal solvent resonances. Multiplicities are indicated by s (singlet), d (doublet), t (triplet), q (quartet), quin (quintet), m (multiplet), and br (broad). Chemical shifts are reported in parts per million (ppm) and coupling constants J are given in Hz. All NMR spectra were recorded at ambient temperature and processed using MestReNova software.

2. General Procedure for the Synthesis of Alkyl-P Analogs

2.1 Protection and Deprotection of Alcohols

2.1a Protection of Hydroxyacetone (Scheme S1B, used for compounds 31 and 36).

Hydroxyacetone (135.0 mmol, 1 equiv), pyridinium toluene-4-sulfonate (PPTs) (1.35 mmol, 0.01 equiv), and 3,4-dihydro-2*H*-pyran (DHP) (270.0 mol, 2 equiv) were combined and stirred at room temperature for 2.5 h, after which residual DHP was removed by evaporation under reduced pressure. The remaining mixture was then partitioned between dichloromethane (200 mL) and saturated $NaHCO_3$ (2 x 100 mL). The combined organic layers were washed with brine (100 mL), dried over Na_2SO_4, filtered, and concentrated to give the crude product. Purification of the desired compound was achieved by silica gel column chromatography using 25% acetone: hexane.

2.1b. Protection of Geraniol (Scheme S1B, used for compounds 40, 41, 42, 43, and 45).

A solution of geraniol (1.0 mmol, 1.0 equiv), PPTs (0.1mmol, 0.1 equiv), and DHP (1.5mmol, 1.5 equiv) in dichloromethane (10 mL) was stirred for 4 h at room temperature, after which residual DHP was removed by evaporation under reduced pressure. The remaining mixture was then partitioned between dichloromethane (50 mL) and saturated $NaHCO_3$ (2 x 35 mL). The combined organic layers were washed with brine (35 mL), dried over Na_2SO_4, filtered, and concentrated to give the crude product. Purification of the desired compound was achieved by silica gel column chromatography using 25% acetone: hexane.

2.1c. Deprotection (Scheme S1C, used for compounds 29, 36, 40, 41, 42, 43, and 45).

Once the allylic scaffold had been prepared, the last synthetic step before phosphorylation was the removal of the THP protecting group. To begin, a solution of the protected compound (0.5 mmol, 1 equiv) and PPTs (0.05 mmol, 0.1 equiv) was added to ethanol (4 mL) and stirred at 55°C for 3 h. Once complete, the reaction was concentrated under reduced pressure to remove ethanol. The raw product was then purified by silica gel chromatography using 10% ethyl acetate: hexane.

2.2. Synthesis of α,β-Unsaturated Esters

2.2a. Wittig Reaction Using Methyl Diethylphosphonoacetate (Scheme S1A, Method a, used for compound 26).

In a 100-mL round-bottom flask, NaH (10.0 mmol, 1.0 equiv) and methyl diethylphosphonoacetate (11.0 mmol, 1.1 equiv) were dissolved in dry THF (20 mL), and the mixture was stirred for 30 mins at 0°C under a nitrogen atmosphere. The commercially available ketone (10.0 mmol, 1.0 equiv) in dry THF (20 mL) was added dropwise to the cooled ylide solution, and the mixture was stirred at 40°C for 12 h. Once the reaction was identified as complete by TLC, it was quenched with a saturated NH_4Cl solution (20 mL) and extracted with ethyl acetate (30 mL x 3) and water (30 mL x 3). The aqueous layer was further extracted with ethyl acetate (25 mL x 2). The resulting organic layers were combined, dried over anhydrous Na_2SO_4, filtered, and concentrated to obtain the crude compound, which was then purified on a silica gel column using 10% ethyl acetate: hexane.

2.2b. Wittig Reaction Using Ethyl(triphenylphosphoranylidene)acetate (Scheme S1A, Method b, used for compounds 13, 17, 19, 20, 22, 30, and 39).

In a 100-mL round-bottom flask, dry ethyl(triphenylphosphoranylidene)acetate (11.0 mmol, 1.1 equiv) was added to the commercially available aldehyde or ketone (10.0 mmol, 1.0 equiv) in dichloromethane (0.5 M) and stirred overnight at room temperature under nitrogen. Once the reaction was identified as complete by TLC, it was quenched with a saturated NH_4Cl solution (20 mL) and extracted with ethyl acetate (30 mL x 3) and water (30 mL x 3). The aqueous layer was further extracted with ethyl acetate (25 mL x 2). The resulting organic layers were combined, dried over anhydrous Na_2SO_4, filtered, and concentrated to obtain the crude compound, which was then purified on a silica gel column using a 10% ethyl acetate: hexane.

2.2c. Wittig Reaction Using Ethyl-2-(triphenylphosphoranylidene)propanoate (Scheme S1A, Method c, used for compound 37).

In a 100-mL round-bottom flask, a solution of crotonaldehyde or acrolein (10.0 mmol, 1.0 equiv) and ethyl-2-(triphenylphosphoranylidene)propanoate (11.0 mmol, 1.1 equiv) were dissolved in DCM (0.5 M), and the resulting mixture was stirred at room temperature under a nitrogen atmosphere. After 15 min, the reaction was heated at 40°C and continued to stir for 12 h. Once the reaction was identified as complete by TLC, it was concentrated to half its original volume and purified on a silica gel column using 10% ethyl acetate: hexane.

2.2d. Wittig Reaction Using Ethyl 2-(diethoxyphosphoryl)acetate (Scheme S1A, Method b, used for compounds 18, 24, 27, 28, 34, and 53).

Dry tetrahydrofuran (0.5 M) was added to sodium hydride (10.0 mmol, 1.0 equiv) in a round bottom flask, followed by the addition of ethyl 2-(diethoxyphosphoryl)acetate (11.0 mmol, 1.1 equiv) under nitrogen at 0°C. The reaction was stirred for 30 min at room temperature and cooled to 0°C. The commercial aldehyde or ketone (10.0 mmol, 1.0 equiv) in tetrahydrofuran (0.5 M) was then added to the cool ylide solution and stirred under nitrogen overnight at room temperature. After the addition of saturated NH_4Cl solution (20 mL), the aqueous layer was washed twice with dichloromethane (50 mL). The combined organic layers were dried over anhydrous Na_2SO_4, filtered, and concentrated to give a crude product, which was subsequently purified by silica gel column chromatography using 10% ethyl acetate: hexane.

2.2e. Synthesis of Wittig-Horner Reaction Derivative (Scheme S1A, used for compounds 15 and 32).

To a solution of ethyl 2-(diethoxyphosphoryl)acetate (1.0 mmol, 1 equiv) in THF (10 mL), MeMgBr (0.9 M in THF, 1.0 mmol, 1 equiv) was added dropwise at room temperature and stirred for 15 min. The starting aldehyde or ketone (1.1 mmol, 1.1 equiv) in THF (5 mL) was then added dropwise to the mixture and refluxed for 18 h. The resulting reaction was quenched with saturated NH_4Cl solution (15 mL) and extracted with ether (3 × 20 mL). The combined organic layers were dried over anhydrous Na_2SO_4, filtered, and concentrated to give a crude product, which was subsequently purified by silica gel column chromatography using 10% ethyl acetate: hexane.

2.3. Synthesis of α,β-Unsaturated Alcohols

2.3a. Reduction of Esters I (Scheme S1B, used for compounds 17, 19, 20, 21, 22, 24, 26, 27, 28, 30, 33, 34, 39, and 53).

Unsaturated ethyl or methyl ester (4.0 mmol, 1.0 equiv) dissolved in ether (10 mL) was added to a suspension of $LiAlH_4$ (5.0 mmol, 1.3 equiv) in dry ether (5 mL) at 0°C. The reaction was stirred under nitrogen for 15 min at 0°C, and after completion was confirmed by TLC, it was quenched with the sequential addition of 0.2 mL water, 0.2 mL of 2 N NaOH, and 0.6 mL water and stirred at room temperature for 1 h. The mixture was subsequently filtered using a celite pad, washed with ether (50 mL), concentrated, and purified on a silica gel column using 25% ethyl acetate: hexane.

2.3b. Reduction of Esters II (Scheme S1B, used for compounds 15, 18, 29, 32, 33, and 36).

Unsaturated ethyl ester (4.0 mmol, 1.0 equiv) was dissolved in ether (10mL) and cooled to -78°C, after which diisobutylaluminum hydride (1.0 M in hexane, 8.4 mmol, 2.1 equiv) was added dropwise to the reaction. The resulting mixture was allowed to warm to room temperature slowly and stirred for 14 h. The reaction was then cooled again to 0°C and quenched with the sequential addition of 10 mL of methanol and 10 mL of 1 M HCl. After stirring to room temperature for 1 h, the aqueous mixture was extracted with dichloromethane (3 x 20 mL), and the combined organic layer was washed with brine (10 mL), dried over Na_2SO_4, and filtered. Finally, the raw product was purified by column chromatography with 25% ethyl acetate: hexane.

2.3c. Reduction of Esters III (Scheme S1B, used for compound 14).

A solution of 2-butyn-1-ol (4.6 mmol, 1 equiv) in dry tetrahydrofuran (20 mL) was added dropwise to Red-Al (7.8 mmol, 1.7 equiv), and the resulting mixture was allowed to stir at room temperature for 18 h. Next, the solution was cooled to -78°C before the dropwise addition of *N*-chlorosuccinimide or selectfluor™ (8.8 mmol, 1.9 equiv) in tetrahydrofuran (10 mL) after which the reaction continued to stir for 1 h. The mixture was then stirred at 0°C for 2 h and subsequently quenched with saturated Rochelle's salt. The aqueous layer was extracted with ether (3 x 30 mL), and the combined organic layers were washed with brine (10 mL), dried over Na_2SO_4, and filtered. Finally, the raw product was purified by column chromatography with 60% hexane: ether.

2.3d. Reduction of Aldehydes (Scheme S1B, used for compounds 8, 12, 16, 38, 46, 47, 49, 50, 51, 52, 54, 55, 56, and 58).

In a flame-dried round-bottom flask charged with a stir bar, $NaBH_4$ (3-6 mmol, 1.5 equiv) was added in a single portion to a solution of the starting aldehyde (2-4 mmol, 1.0 equiv) in methanol (10-20 mL) under a nitrogen atmosphere at 0°C. The mixture was allowed to warm to room temperature and stirred for 2 h. Once the reaction was identified as complete by TLC, it was

quenched with 10% NaOH (w/v) and stirred for another 10 min at room temperature. The crude mixture was concentrated under reduced pressure and then extracted with DCM (20 mL x 3) and water (20 mL x 3). The combined organic layers were dried over Na_2SO_4, filtered, and concentrated under reduced pressure to yield the corresponding alcohol, which was taken to the next step without further purification.

2.4 Synthesis of Alkynes

2.4a. Chlorination (used for compound 29).

Following deprotection, the hydroxyacetone ester (2.0 mmol, 1 equiv) and triethylamine (2.0 mmol, 1.0 equiv) were dissolved in dichloromethane (20 mL). Then, methane sulfonyl chloride (1.0 mmol, 0.5 equiv) and a catalytic amount of 4-dimethylaminopyridine were added to the solution at 0°C under nitrogen, and the resulting mixture was stirred for 3 h. This crude mixture was concentrated under reduced pressure to remove the dichloromethane and yield the corresponding chloride product, which was taken to the next step without further purification.

2.4b. Addition of Alkyne (Scheme S1C, used for compound 29).

The chlorinated hydroxyacetone ester (2.0 mmol, 1 equiv) was then directly dissolved in ethanol (5 mL), after which propargyl chloride (3.0 mmol, 1.5 equiv) was added. The solution was heated to 60°C for 24 h and then was concentrated under reduced pressure to remove the ethanol. The resulting aqueous mixture was extracted with ethyl acetate (3 x 20 mL), and the combined organic layers were washed with brine (10 mL), dried over Na_2SO_4, and filtered. The raw product was purified by column chromatography with 25% ethyl acetate: hexane.

2.5. Synthesis of Azides (used for compounds 10, 32, 33 and 36).

2.5a. Method A: (used for compounds 10, 32, and 36).

To a stirred solution of starting diol (20.0 mmol, 1.0 equiv) and triethylamine (20.0 mmol, 1.0 equiv) in dichloromethane (20 mL), methane sulfonyl chloride (10 mmol, 0.5 equiv) and a catalytic amount of 4-dimethylaminopyridine were added at 0°C under nitrogen and stirred for 3 h. The resulting crude mixture was concentrated under reduced pressure to remove the dichloromethane, and the oily raw product was directly dissolved in 80 mL ethanol: water (5:1 [v/v]) without further purification. Sodium azide (30.0 mmol, 1.5 equiv) was added, and the solution was heated to 60°C for 24 h. Once complete, the reaction was concentrated under reduced pressure to remove the ethanol, with the remaining aqueous solution then being extracted with ethyl acetate (3 x 20 mL). The combined organic layers were washed with brine (10 mL), dried over Na_2SO_4, and filtered, and the raw product was purified by column chromatography with 30% ethyl acetate: hexane.

2.5b. Method B: (used for compound 33).

To a solution of 1-(carbomethoxy)cyclohexene (3.0 mmol, 1.0 equiv) in 20 mL of carbon tetrabromide N-bromosuccinamide (6.0 mmol, 2.0 equiv) was added, and the mixture was stirred at 0°C for 1 h. The remaining mixture was then partitioned between dichloromethane (50 mL) and water (50 mL). The combined organic layers were washed with brine (35 mL), dried over Na_2SO_4, filtered, and concentrated to give the crude product. Next, a solution of Methyl 3-bromo-1-cyclohexene-1-carboxylate (2.0 mmol, 1.0 equiv) in 5 mL of DMF was cooled to 0°C, followed by the addition of sodium azide (4.1 mmol, 2.0 equiv) was added, and the solution was heated to 60°C for overnight. The remaining mixture was then partitioned between ether (50 mL) and water (50 mL). The combined organic layers were washed with brine (35 mL), dried over Na_2SO_4,

filtered, and concentrated to give the crude product and the raw product was purified by column chromatography with 30% ethyl acetate: hexane.

2.6. Synthesis of Geraniol Derivatives

2.6a. Oxidation of THP-Protected Geraniol (used for compounds 40, 41, 42, 43, and 45).

THP-protected geraniol (5.0 mmol, 1.0 equiv) in 30 mL dichloromethane was added to a solution of salicylic acid (2.5 mmol, 0.5 equiv), selenium dioxide (0.6 mmol, 0.12 equiv), and *t*-butyl hydroperoxide (70% wt in water 10.0 mmol, 2.0 equiv) and stirred overnight at room temperature. The reaction mixture was quenched with saturated $NaHCO_3$ (15 mL) and extracted with dichloromethane (2 x 30 mL), and the combined organic layer was washed with brine (10 mL), dried over Na_2SO_4, and filtered. The raw product was purified by column chromatography with 5-15% ethyl acetate: hexane.

2.6b. Synthesis of Geraniol Chlorides (used for compounds 40, 42, 43, and 45).

After the oxidation of the protected geraniol, the resulting compound (3.0 mmol, 1.0 equiv) was dissolved in dry dichloromethane (20 mL) with 4-dimethylaminopyridine (1.8 mmol, 0.6 equiv), tosylchloride (3.8 mmol, 1.25 equiv), and triethylamine (3.0 mmol, 1.0 equiv) under nitrogen at room temperature, and the resulting mixture was stirred for 3 h at room temperature. The solution was then treated with lithium chloride (6.0 mmol, 2.0 equiv) and stirred for another 2 h, with the resulting crude mixture being concentrated under reduced pressure to remove dichloromethane. The residue was treated with water (10 mL) and extracted with ether (3 × 15 mL). and the combined organic layers were washed with brine (10 mL), dried over Na_2SO_4, and filtered, which was taken to the next step without further purification.

2.6c. Grignard Reactions of Geraniol Chloride (used for compound 40).

Grignard reagents (2.0 M in THF, 2.1 mmol, 2.0 equiv) were added dropwise to a solution of geraniol chloride (1.1 mmol, 1.0 equiv) in THF (10 mL) at 0°C and stirred for 2 h. The reaction was then quenched with saturated NH_4Cl solution (10 mL) and extracted with ether (3 × 20 mL). The combined organic layers were dried over anhydrous Na_2SO_4, filtered, and concentrated to give a crude product, which was subsequently purified by silica gel chromatography using 10% ethyl acetate: hexane.

2.6d. Synthesis of TMS-Protected Alcohol (used for compound 41).

tert-Butylchlorodimethylsilane (2.0 mmol, 1.0 equiv) and *N*,*N*-dimethylaminopyridine (0.02 mmol, 0.01 equiv) were added to a stirred solution of the THP-protected geraniol ether (2.0 mmol, 1.0 equiv) in dichloromethane (10 mL) at 0ºC. After the addition of imidazole (4.0 mmol, 2.0 equiv), the mixture was allowed to react for 1 h. It was then extracted with water (3 x 20 mL), and the combined organic layers were dried over Na_2SO_4, filtered, and concentrated under reduced pressure. The crude product was subsequently purified by silica gel chromatography using 25% ethyl acetate: hexane.

2.6e. Williamson Ether Synthesis (used for compounds 43 and 45).

Dry acetonitrile (0.5 M) was combined with sodium hydride (1.3 mmol, 1.3 equiv) in a round-bottom flask, followed by the dropwise addition of alkyl or aromatic alcohol (1.0 mmol, 1.0 equiv) in acetonitrile (1 mL). Finally, alkyl halide (1.3 mmol, 1.3 equiv) was added, and the reaction mixture was stirred under nitrogen at room temperature for 1 h. The reaction was then quenched

with saturated NH_4Cl solution (10 mL) and extracted with ethyl acetate (20 mL x 3). The combined organic layers were dried over anhydrous Na_2SO_4, filtered, and concentrated to give a crude product, which was subsequently purified by silica gel chromatography using 10% ethyl acetate: hexane.

2.7. Phosphorylation

2.7a. Synthesis of Alkyl-Monophosphates Using Bis(triethylammonium)phosphate (TEAP) (Scheme S2, Method a, used for compounds 1, 5, 6, 7, 9, 11, 12, 13, 15, 16, 17, 18, 19, 20, 21, 23, 24, 25, 26, 27, 28, 29, 32, 33, 34, 35, 36, 37, 38, 39, 40, 41, 42, 43, 45, 47, 48, 49, 53, 54, 55, and 56).

TEAP solution was prepared fresh for each reaction by combining 36 mL solution A (94 mL acetonitrile and 25 mL H_3PO_4) and 60 mL solution B (110 mL triethylamine and 100 mL acetonitrile). Then, to a stirred solution of alkyl alcohol (1.0-2.0 mmol, 1.0 equiv) in trichloroacetonitrile (25.0-50.0 mmol, 25.0 equiv), 2.5-5 mL of TEAP solution was added and stirred at room temperature for 5 min. Another 2.5-5 mL of TEAP solution was added in two portions at 5 min interval and continued to stir at room temperature for 15 min. Once the reaction was identified as complete by TLC, the crude residue was purified by flash column chromatography on silica gel using isopropanol/NH_4OH/H_2O as the solvent in a 7:2:1 ratio. The combined fractions containing the desired product were concentrated under reduced pressure, and the resulting alkyl monophosphate was dissolved in 0.025 M NH_4HCO_3, frozen, and lyophilized until dry. **NOTE:** ^{1}H NMR of several alkyl-Ps generated using this method were contaminated with TEAP.

2.7b. Synthesis of Alkyl-Monophosphates Using Tris(tetrabutylammonium) Phosphate (TBAP) (Scheme S2, Method b, used for compounds 2, 3, 4, 8, 10, 14, 22, 30, 31, 44, 46, 50, 51, 52, 57, and 58).

To a stirred solution of alkyl-chloride/tosylate/mesylate (1.0 mmol, 1.0 equiv) in acetonitrile (1 mL), TBAP (2.0 mmol, 2.0 equiv) in acetonitrile (5 mL) was added and allowed to stir overnight at room temperature. Subsequently, the solvent was removed under the rotary evaporator, and the crude residue was purified by flash column chromatography on silica gel using isopropanol/NH_4OH/H_2O as the solvent in a 7:2:1 ratio. The combined fractions containing the desired product were concentrated under reduced pressure and passed through a Dowex 50WX8 ion exchange column (1 x 8 cm) that had been 1) pre-equilibrated with concentrated NH_4OH: H_2O (3:1) and 2) flushed with buffer (0.025 M NH_4HCO_3) until the pH was 8.0. Finally, the alkyl-monophosphate was eluted with two column volumes of 0.025 M NH_4HCO_3 buffer, frozen in liquid N_2, and lyophilized until dry.

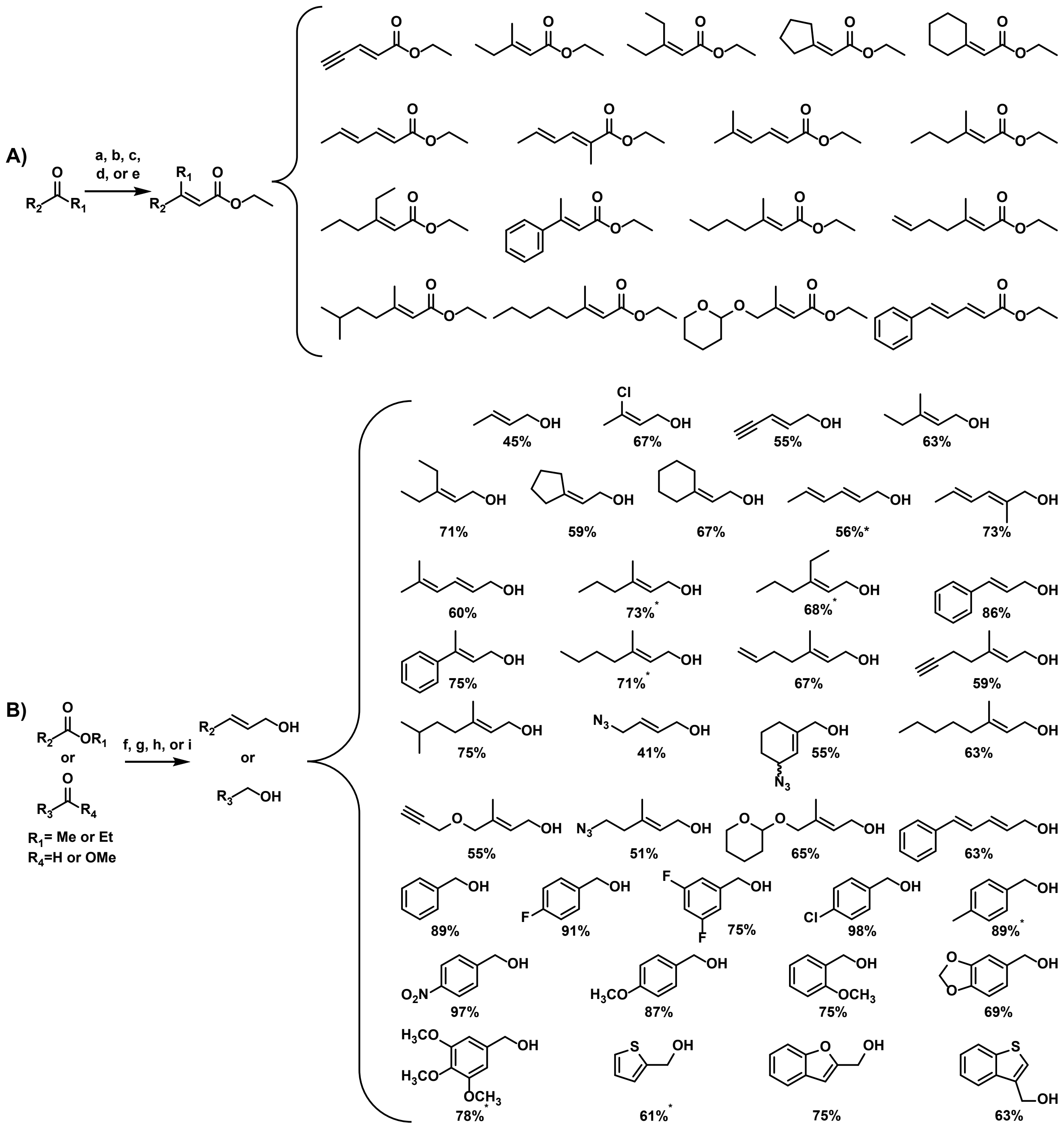

Scheme S1. General Methods for the Synthesis of Alkyl-Esters (**A**) and Alkyl-Alcohols (**B**), where reaction conditions are **a.** NaH, $(C_2H_5O)_2P(O)CH_2CO_2CH_3$, THF, 40ºC; **b.** $PPh_3CHCOOEt$, DCM; **c.** PPh_3CCH_3COOEt, DCM; **d.** $(C_2H_5O)_2P(O)CH_2CO_2C_2H_5$, THF, 0ºC; **e.** $(C_2H_5O)_2P(O)CH_2CO_2C_2H_5$, MeMgBr, THF, reflux; **f.** $LiAlH_4$, Ether, 0ºC; **g.** $[(CH_3)_2CHCH_2]_2AlH$, Ether, -78ºC; **h.** $NaAlH_2(OCH_2CH_2OCH_3)_2$, THF, rt 18 h→-78ºC 1 h→ 0ºC 2h; **i.** $NaBH_4$, MeOH, 0ºC. Yields of purified products are shown below the structures. *^{1}H NMR data not available.

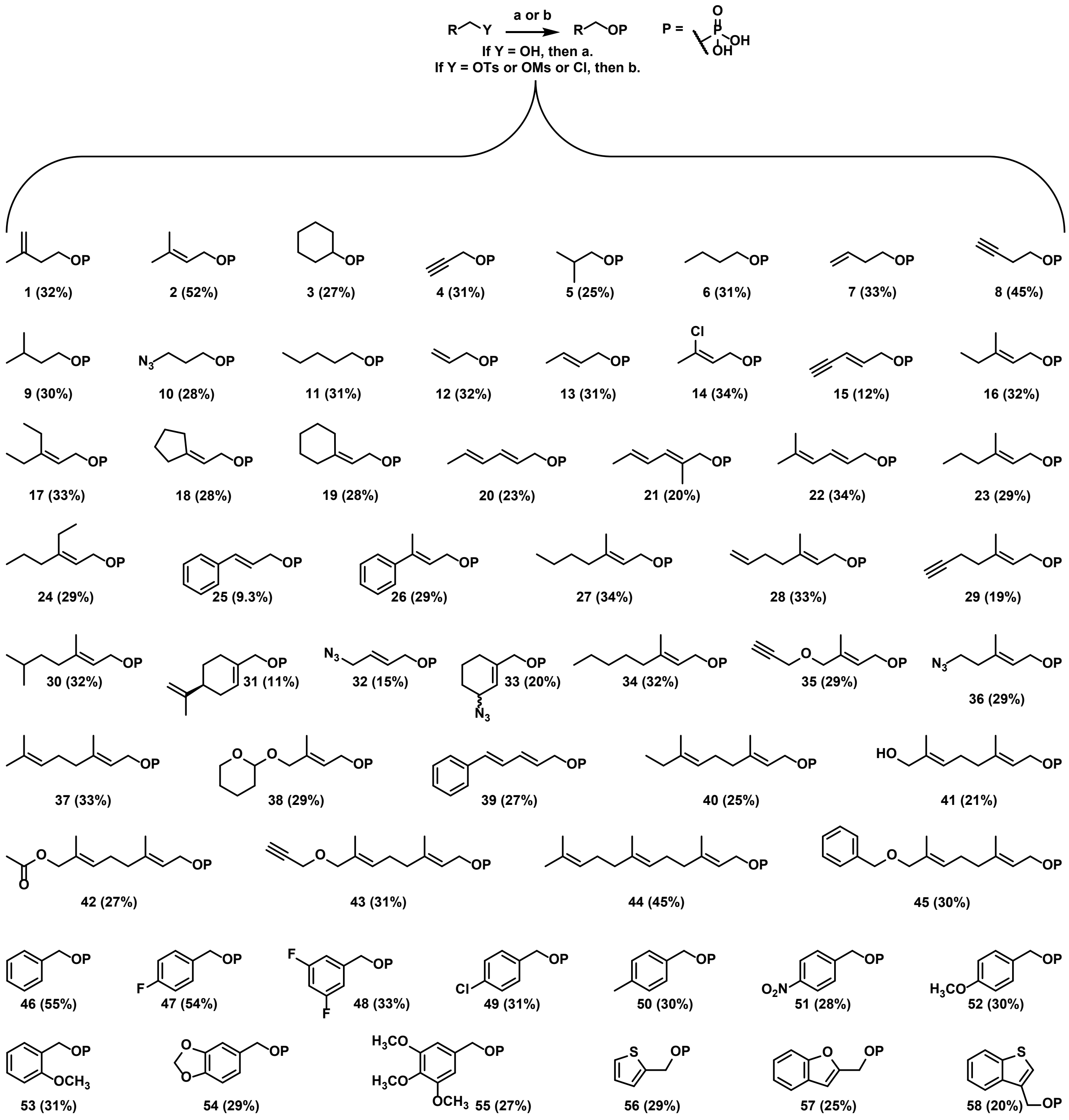

Scheme S2. General Methods for the Synthesis of Alkyl-Monophosphates: **a.** Bis(triethylammonium)phosphate, CCl_3CN, CH_3CN; **b.** Tris(tetrabutylammonium) phosphate, CH_3CN. Final product yields are shown in parentheses.

Table S1. Summary of HRMS Data for Synthetic Alkyl-Ps **1-36**

Alkyl-P	Chemical Formula	Calculated Mass (Da)	Observed Mass (Da)	Error (ppm)
**1**	$C_5H_{10}O_4P$ [M-H]$^-$	165.03166	165.0317	0.24
**2**	$C_5H_{10}O_4P$ [M-H]$^-$	165.03166	165.0311	3.4
**3**	$C_6H_{12}O_4P$ [M-H]$^-$	179.04731	179.0476	1.6
**4**	$C_3H_4O_4P$ [M-H]$^-$	134.98471	134.9846	0.81
**5**	$C_4H_{10}O_4P$ [M-H]$^-$	153.03166	153.0317	0.26
**6**	$C_4H_{10}O_4P$ [M-H]$^-$	153.03166	153.0310	4.3
**7**	$C_4H_8O_4P$ [M-H]$^-$	151.01601	151.0161	0.6
**8**	$C_4H_6O_4P$ [M-H]$^-$	149.00036	148.9996	5.1*
**9**	$C_5H_{12}O_4P$ [M-H]$^-$	167.04731	167.0472	0.66
**10**	$C_3H_7N_3O_4P$ [M-H]$^-$	180.01741	180.0177	1.6
**11**	$C_5H_{12}O_4P$ [M-H]$^-$	167.04731	167.0474	0.54
**12**	$C_3H_6O_4P$ [M-H]$^-$	137.00036	137.0001	1.9
**13**	$C_4H_8O_4P$ [M-H]$^-$	151.01601	151.0159	0.73
**14**	$C_4H_7ClO_4P$ [M-H]$^-$	184.97704	184.9767	1.8
**15**	$C_5H_6O_4P$ [M-H]$^-$	161.00036	161.0008	1.5
**16**	$C_6H_{12}O_4P$ [M-H]$^-$	179.04731	179.0463	5.6*
**17**	$C_7H_{14}O_4P$ [M-H]$^-$	193.06296	193.0627	1.3
**18**	$C_7H_{12}O_4P$ [M-H]$^-$	191.04731	191.0476	1.5
**19**	$C_8H_{14}O_4P$ [M-H]$^-$	205.06296	205.0628	0.78
**20**	$C_6H_{10}O_4P$ [M-H]$^-$	177.03166	177.0316	0.34
**21**	$C_7H_{12}O_4P$ [M-H]$^-$	191.04731	191.0472	0.58
**22**	$C_7H_{12}O_4P$ [M-H]$^-$	191.04731	191.0473	0.050
**23**	$C_7H_{14}O_4P$ [M-H]$^-$	193.06296	193.0629	0.31
**24**	$C_8H_{16}O_4P$ [M-H]$^-$	207.07861	207.0800	13*
**25**	$C_9H_{10}O_4P$ [M-H]$^-$	213.03166	213.0307	4.5
**26**	$C_{10}H_{12}O_4P$ [M-H]$^-$	227.04731	227.0473	0.040
**27**	$C_8H_{16}O_4P$ [M-H]$^-$	207.07861	207.0798	5.7*
**28**	$C_8H_{14}O_4P$ [M-H]$^-$	205.06296	205.0629	0.29
**29**	$C_8H_{12}O_4P$ [M-H]$^-$	203.04731	203.0476	1.4
**30**	$C_9H_{18}O_4P$ [M-H]$^-$	221.09426	221.0952	4.3
**31**	$C_{10}H_{16}O_4P$ [M-H]$^-$	231.07861	231.0791	2.1
**32**	$C_4H_7N_3O_4P$ [M-H]$^-$	192.01741	192.0166	4.2
**33**	$C_7H_{11}N_3O_4P$ [M-H]$^-$	232.04871	232.0491	1.7
**34**	$C_9H_{18}O_4P$ [M-H]$^-$	221.09426	221.0954	5.2*
**35**	$C_8H_{12}O_5P$ [M-H]$^-$	219.04222	219.0421	0.55
**36**	$C_6H_{11}N_3O_4P$ [M-H]$^-$	220.04871	220.0485	0.95
*Error higher than expected.				

Table S2. Summary of HRMS Data for Synthetic Alkyl-Ps **37-58**

Alkyl-P	Chemical Formula	Calculated Mass (Da)	Observed Mass (Da)	Error (ppm)
**37**	$C_{10}H_{18}O_4P$ [M-H]$^-$	233.09426	233.0945	1.0
**38**	$C_{10}H_{18}O_6P$ [M-H]$^-$	265.08409	265.0839	0.72
**39**	$C_{11}H_{12}O_4P$ [M-H]$^-$	239.04731	239.0476	1.2
**40**	$C_{11}H_{20}O_4P$ [M-H]$^-$	247.10991	247.1058	16.6*
**41**	$C_{10}H_{18}O_5P$ [M-H]$^-$	249.08917	249.0893	0.52
**42**	$C_{12}H_{20}O_6P$ [M-H]$^-$	291.09974	291.0995	0.82
**43**	$C_{13}H_{20}O_5P$ [M-H]$^-$	287.10482	287.1062	4.8
**45**	$C_{17}H_{24}O_5P$ [M-H]$^-$	339.13612	339.1373	3.5
**46**	$C_7H_8O_4P$ [M-H]$^-$	187.01601	187.0160	0.053
**47**	$C_7H_7FO_4P$ [M-H]$^-$	205.00659	205.0060	2.9
**48**	$C_7H_6F_2O_4P$ [M-H]$^-$	222.99717	222.9980	3.7
**49**	$C_7H_7ClO_4P$ [M-H]$^-$	220.97704	220.9778	3.4
**50**	$C_8H_{10}O_4P$ [M-H]$^-$	201.03166	201.0326	4.7
**51**	$C_7H_7NO_6P$ [M-H]$^-$	232.00109	232.0021	4.4
**52**	$C_8H_{10}O_5P$ [M-H]$^-$	217.02658	217.0270	1.9
**53**	$C_8H_{10}O_5P$ [M-H]-	217.02658	217.0256	4.5
**54**	$C_8H_8O_6P$ [M-H]$^-$	231.00584	231.0070	5.0
**55**	$C_{10}H_{14}O_7P$ [M-H]$^-$	277.04770	277.0476	0.36
**56**	$C_5H_6O_4PS$ [M-H]$^-$	192.97244	192.9729	2.4
**57**	$C_9H_8O_5P$ [M-H]$^-$	227.01093	227.0109	0.13
**58**	$C_9H_8O_4PS$ [M-H]$^-$	242.98808	242.9887	2.6

*Error higher than expected.
NOTE: 44 was obtained commercially, so HRMS was not performed.

3-Methylbut-3-en-1-yl Phosphate (1):

The title product was obtained as an ivory solid from 3-methylbut-3-en-1-ol following the procedure described in *Method 2.7b.*

TLC: (^{i}PrOH: NH_4OH: H_2O 7:2:1 v/v): R_f = 0.68.

^{1}H NMR (300 MHz, D_2O): δ 3.94 (dd, J = 9.0, 4.3 Hz, 2H), 2.34 (t, J = 6.6 Hz, 2H), 1.73 (s, 3H).

^{31}P NMR (122 MHz, D_2O): δ 0.29.

HRMS-ESI: Calculated for $C_5H_{10}O_4P$ $[M-H]^-$: 165.03166; Found: 165.0317.

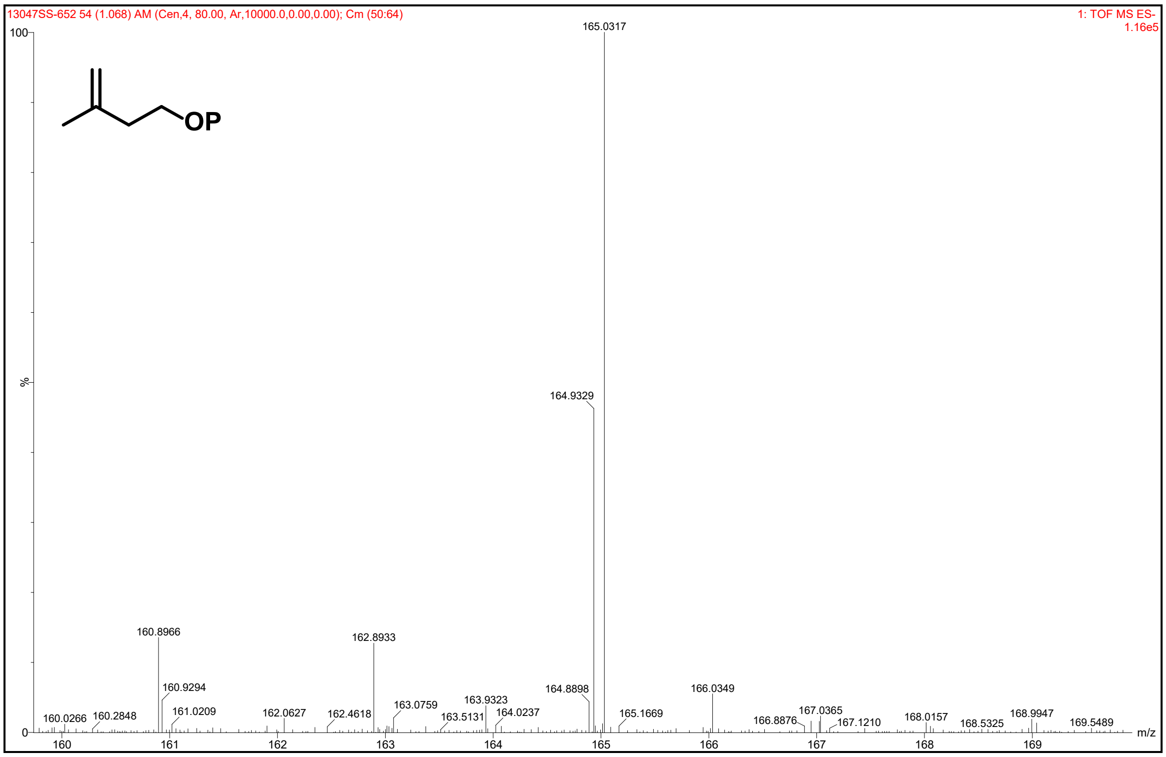

Figure S1. HRMS-ESI$^-$ of **1.**

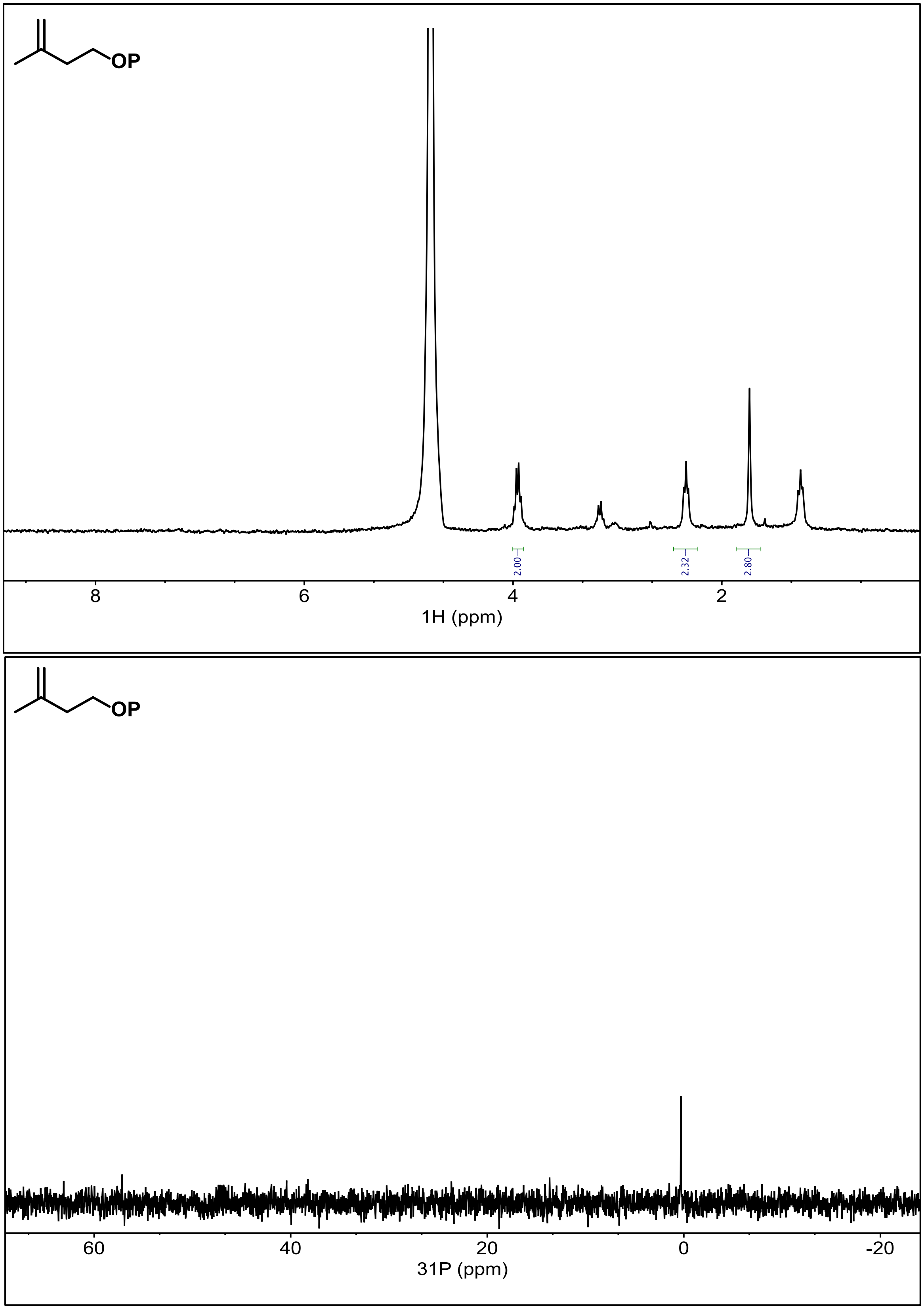

Figure S2. ^{1}H NMR (300 MHz, D_2O) and ^{31}P NMR of **1** (122 MHz, D_2O).

3-Methylbut-2-en-1-yl Phosphate (2):

The title product was obtained as an ivory solid from 3-methylbut-2-en-1-ol following the procedure described in *Method 2.7b*.

<u>TLC (^{i}PrOH: NH_4OH: H_2O 7:2:1 v/v):</u> R_f = 0.67.

<u>^{1}H NMR (400 MHz, D_2O):</u> δ 5.55 – 5.32 (m, 1H), 4.34 (t, *J* = 6.5 Hz, 3H), 1.74 (s, 6H)

<u>^{31}P NMR (162 MHz, D_2O):</u> δ 2.08.

<u>HRMS-ESI:</u> Calculated for $C_5H_{10}O_4P$ $[M-H]^-$: 165.03166; Found: 165.0311.

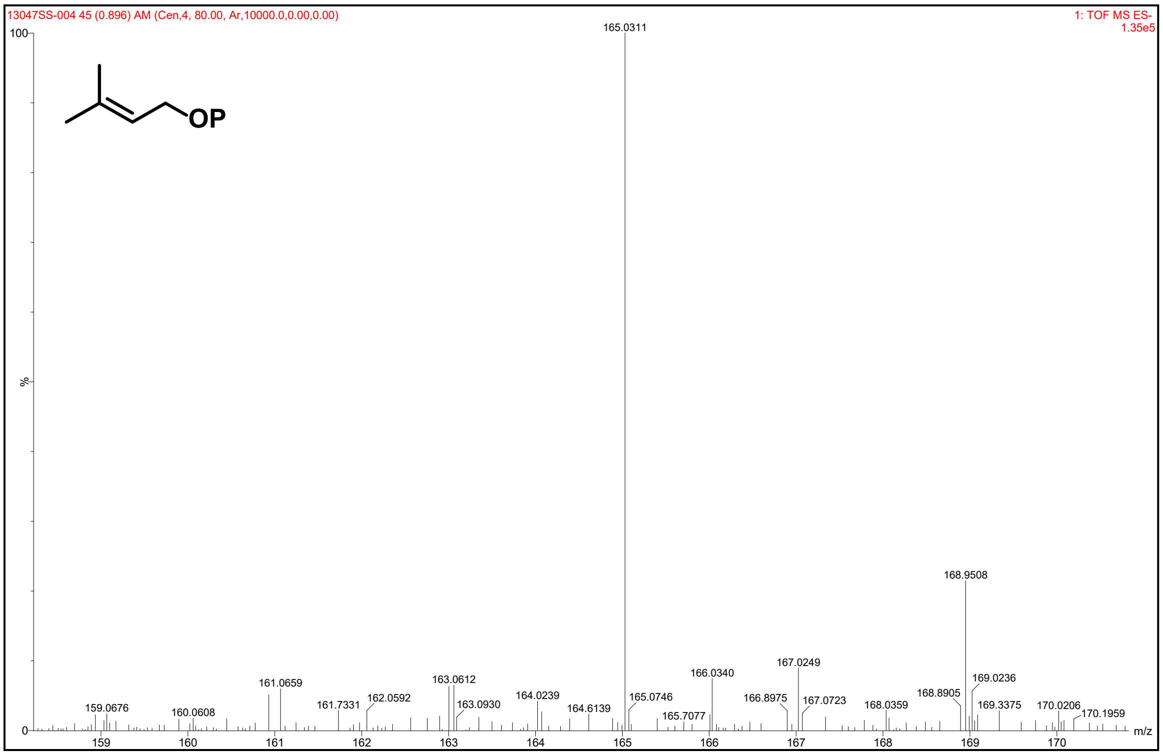

Figure S3. HRMS-ESI$^-$ of **2.**

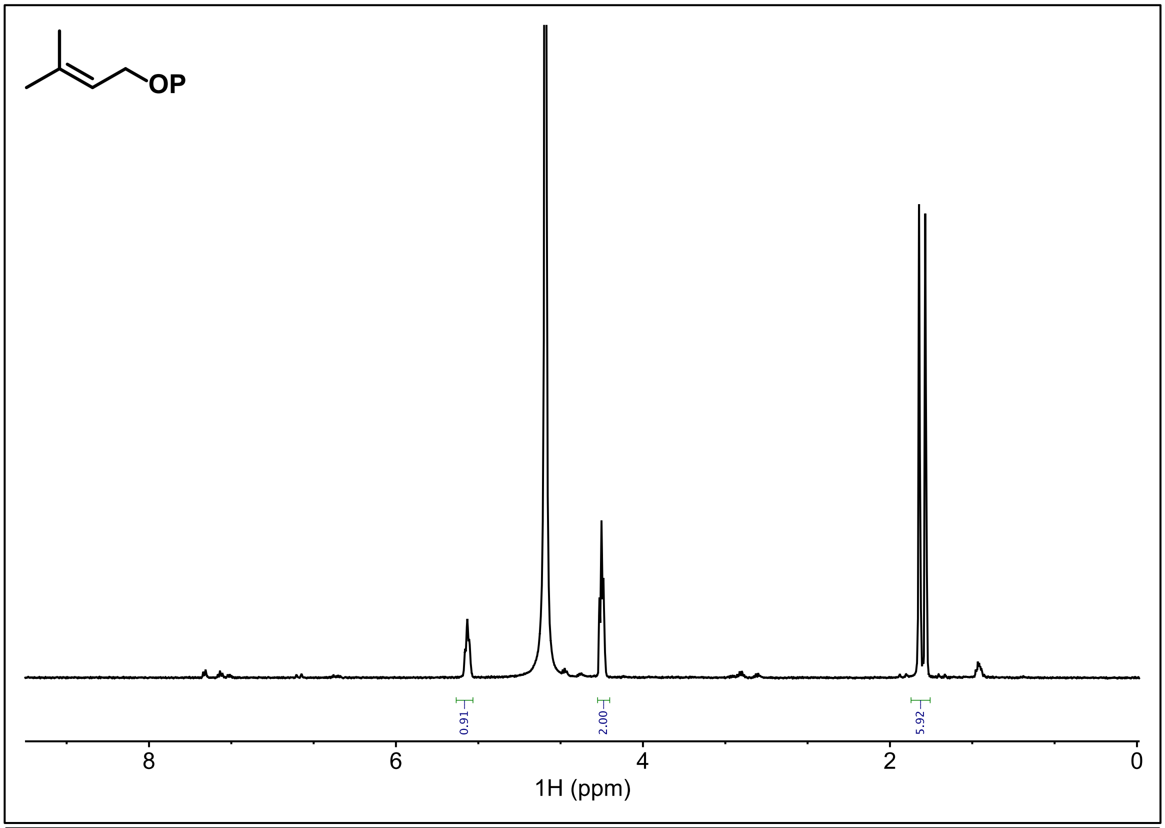

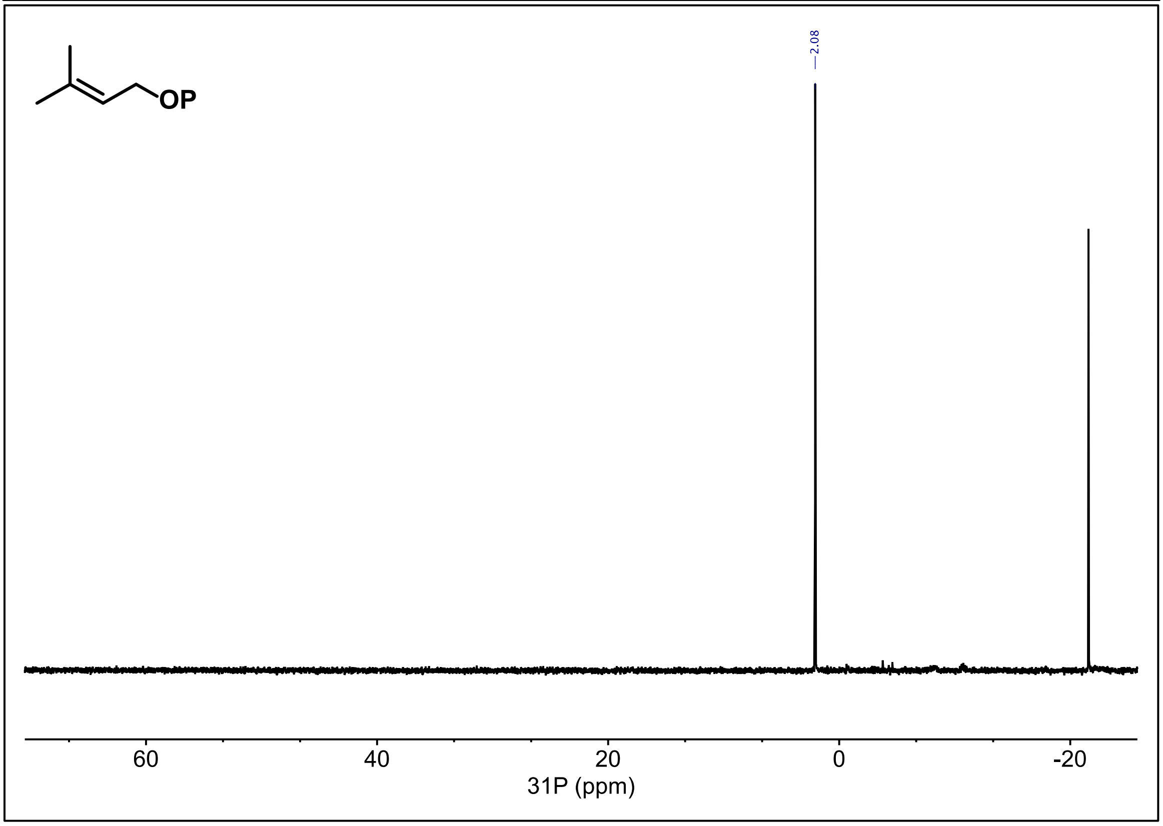

Figure S4. ^{1}H NMR (400 MHz, D_2O) and ^{31}P NMR of **2** (162 MHz, D_2O).

Cyclohexyl Phosphate (3):

The title product was obtained as a white solid from cyclohexanol following the procedure described in *Method 2.7b.*

TLC (^{i}PrOH: NH_4OH: H_2O 7:2:1 v/v): R_f = 0.68.

^{1}H NMR (300 MHz, D_2O): δ 4.03 (dtd, J = 12.3, 8.8, 4.2 Hz, 1H), 1.90 (d, J = 11.1 Hz, 2H), 1.81 – 1.59 (m, 2H), 1.28 (dt, J = 25.6, 12.4 Hz, 6H).

^{31}P NMR (122 MHz, D_2O): δ -0.29.

HRMS-ESI: Calculated for $C_6H_{12}O_4P$ [M-H]$^-$: 179.04731; Found: 179.0476.

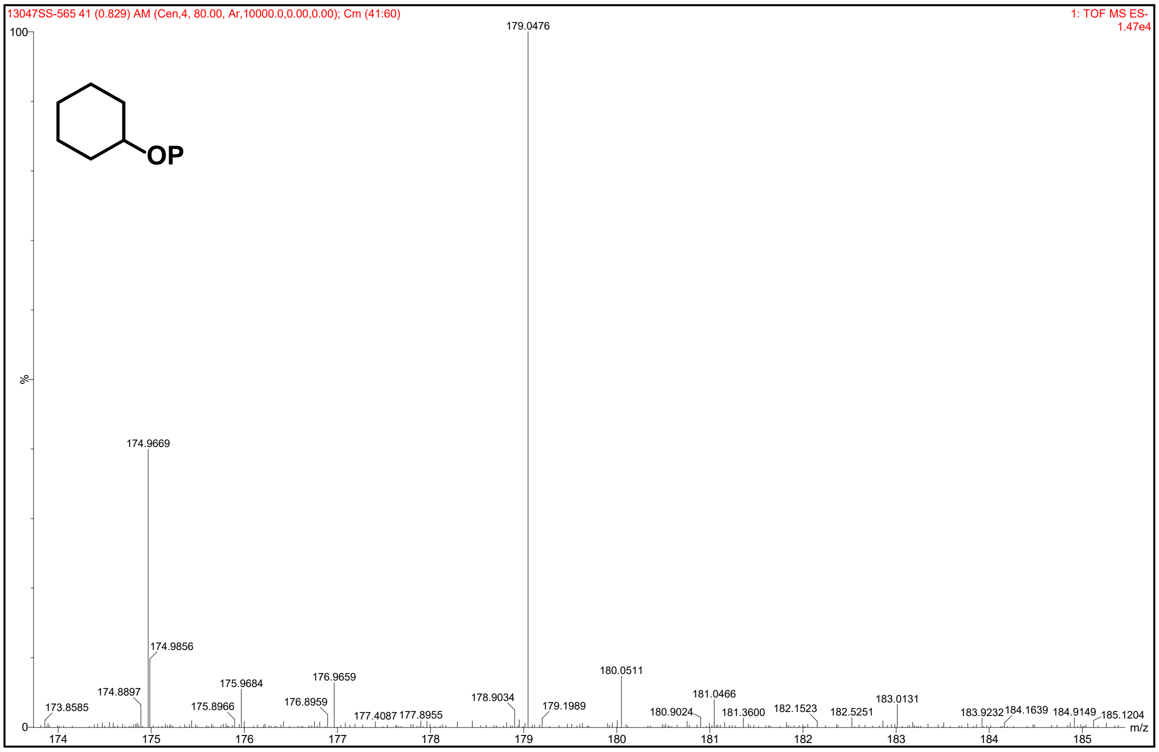

Figure S5. HRMS-ESI$^-$ of **3.**

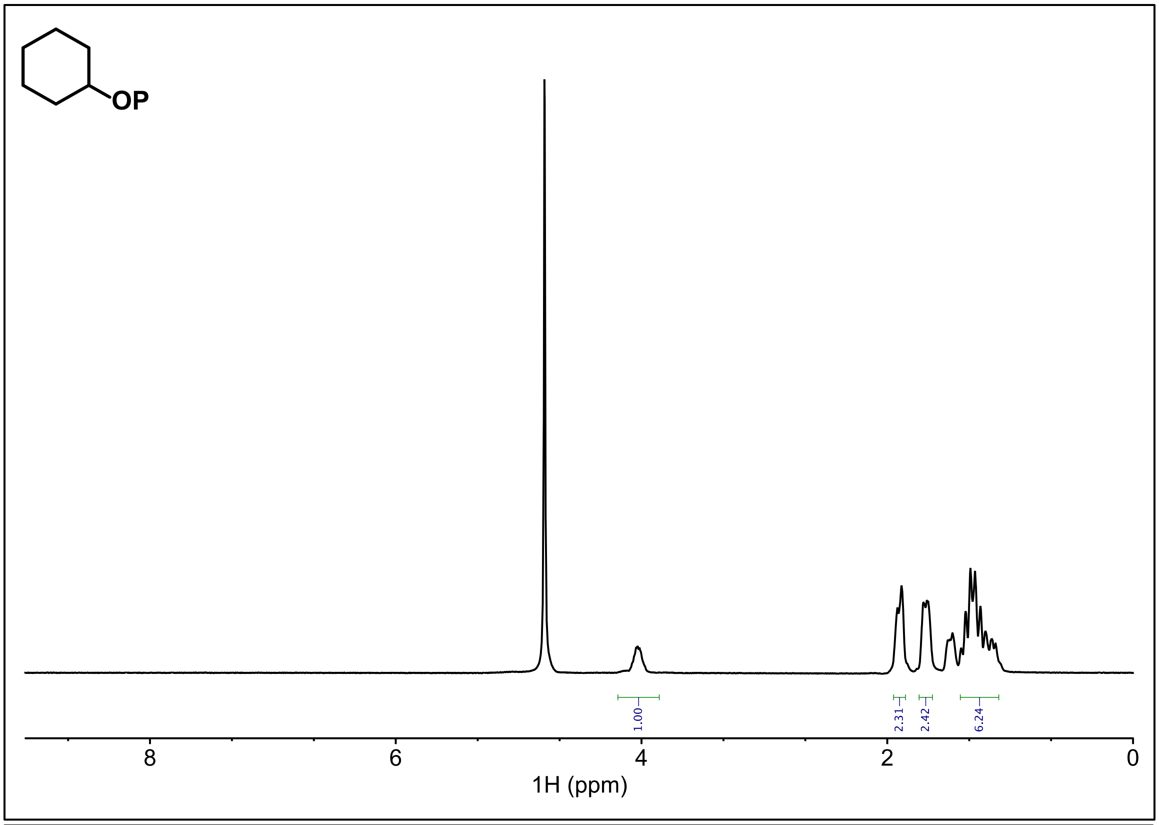

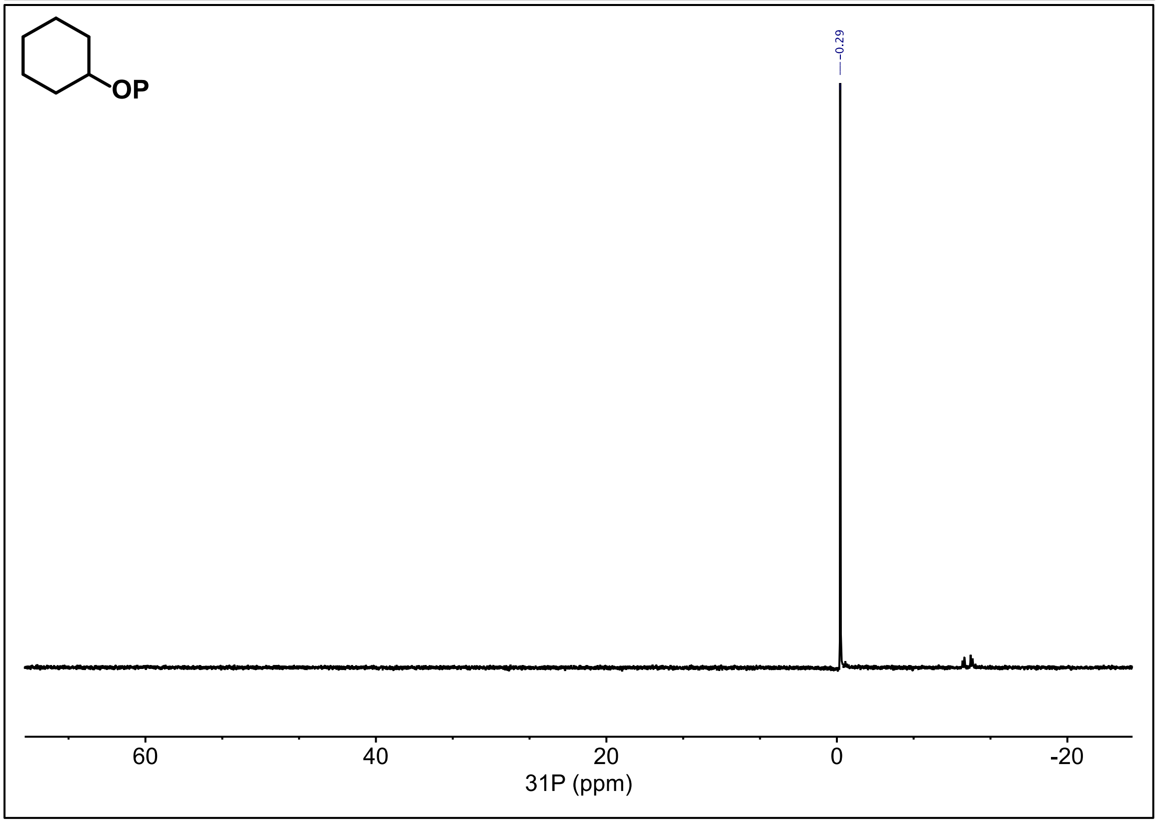

Figure S6. ^{1}H NMR (300 MHz, D_2O) and ^{31}P NMR of **3** (122 MHz, D_2O).

Prop-2-yn-1-yl Phosphate (4):
The title product was obtained as a brown solid from prop-2-yn-1-ol following the procedure described in *Method 2.7b.*
TLC (^{i}PrOH: NH_4OH: H_2O 7:2:1 v/v): R_f = 0.64.
^{1}H NMR (400 MHz, D_2O): δ 4.49 (dd, J = 8.8, 2.4 Hz, 2H), 2.88 (d, J = 2.3 Hz, 1H).
^{31}P NMR (162 MHz, D_2O): δ 1.16.
HRMS-ESI: Calculated for $C_3H_4O_4P$ $[M-H]^-$: 134.98471; Found: 134.9846.

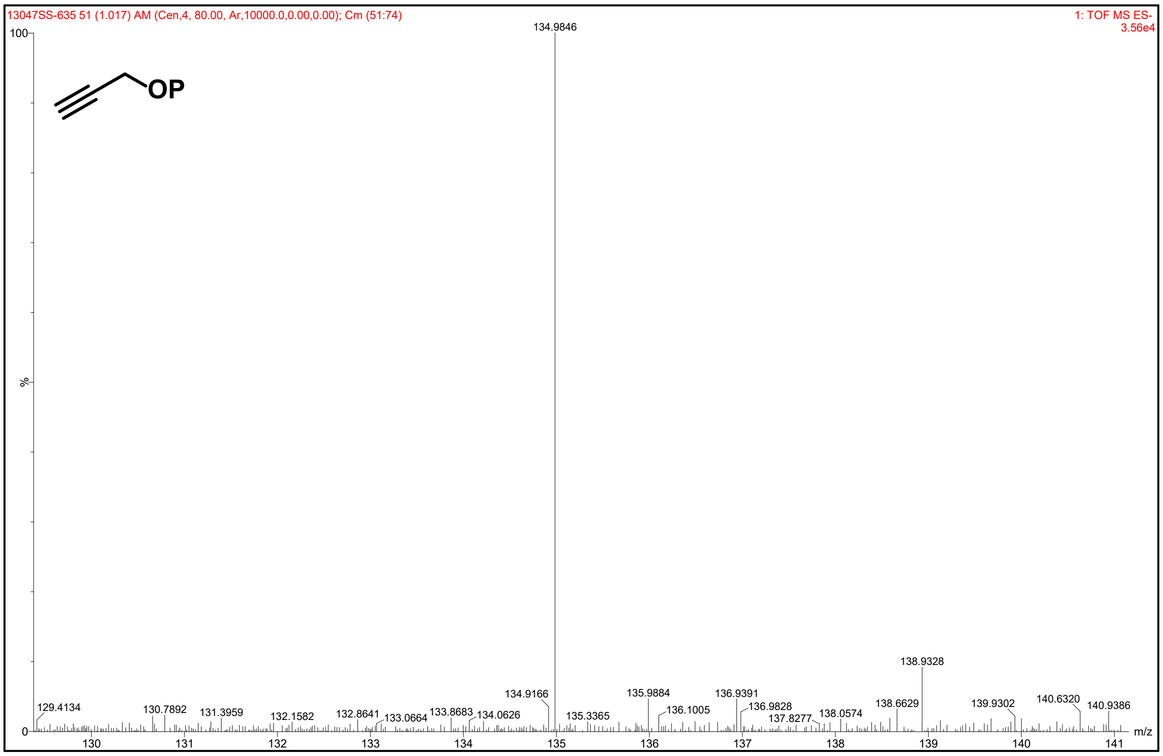

Figure S7. HRMS-ESI$^-$ of **4.**

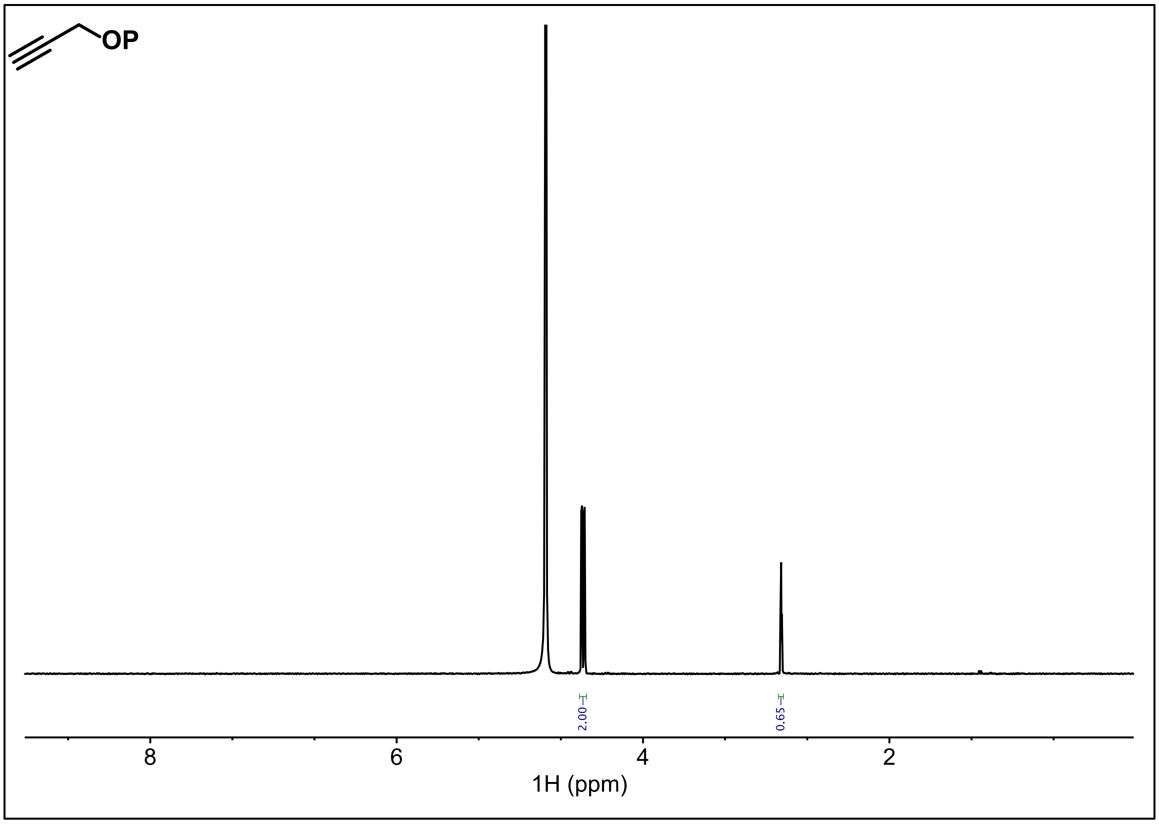

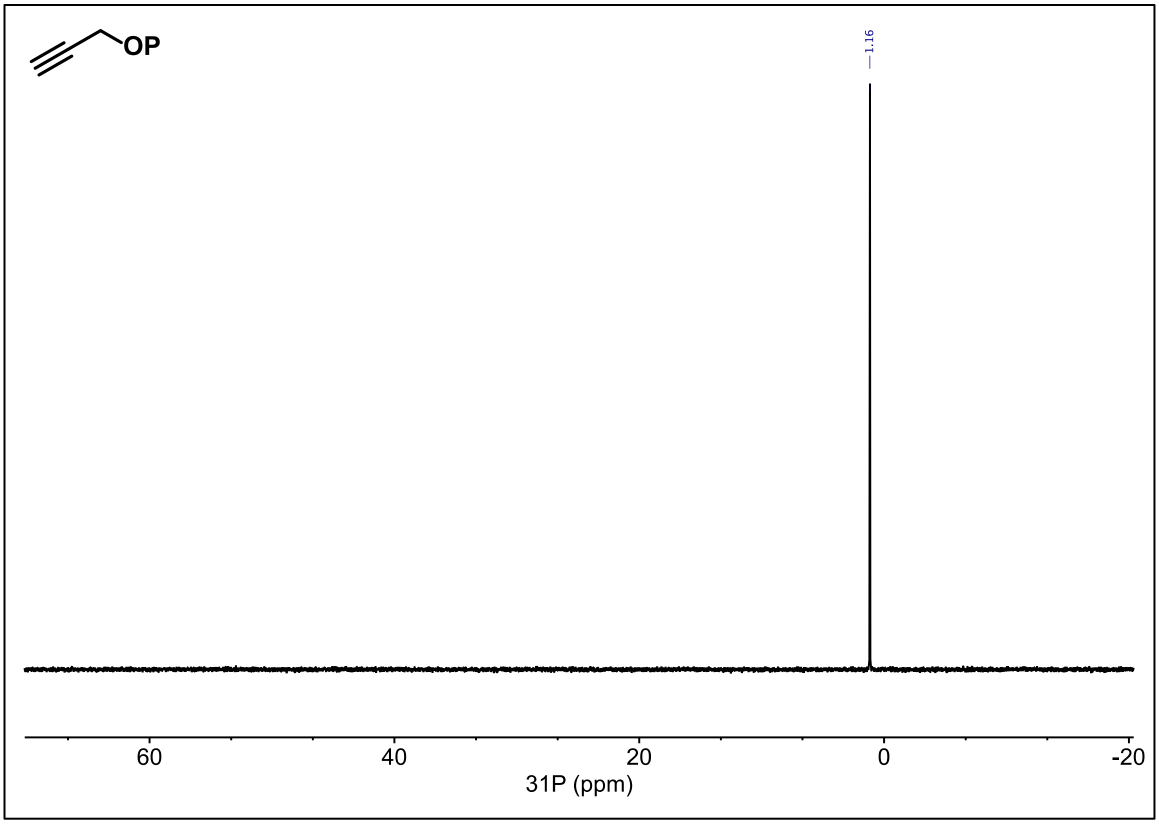

Figure S8. ^{1}H NMR (400 MHz, D_2O) and ^{31}P NMR of **4** (162 MHz, D_2O).

Isobutyl Phosphate (5):
The title product obtained as an ivory solid from 2-methylpropan-1-ol following the procedure described in *Method 2.7b.*
<u>TLC (^{i}PrOH: NH_4OH: H_2O 7:2:1 v/v):</u> R_f = 0.67.
<u>^{1}H NMR (400 MHz, D_2O):</u> δ 3.62 (t, *J* = 6.5 Hz, 2H), 1.88 (dh, *J* = 13.5, 6.6 Hz, 1H), 1.37 (h, *J* = 7.4 Hz, 6H).
<u>^{31}P NMR (162 MHz, D_2O):</u> δ 0.85.
<u>HRMS-ESI:</u> Calculated for $C_4H_{10}O_4P$ $[M-H]^-$: 153.03166; Found: 153.0317.

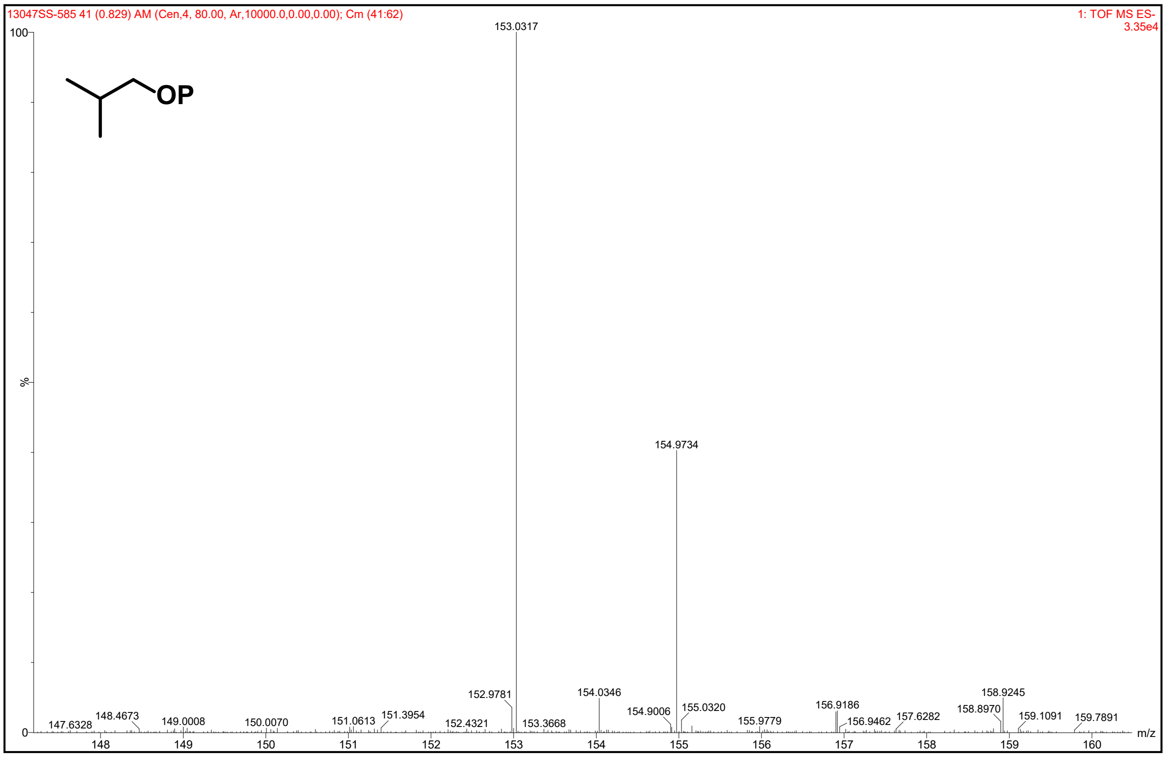

Figure S9. HRMS-ESI$^-$ of **5.**

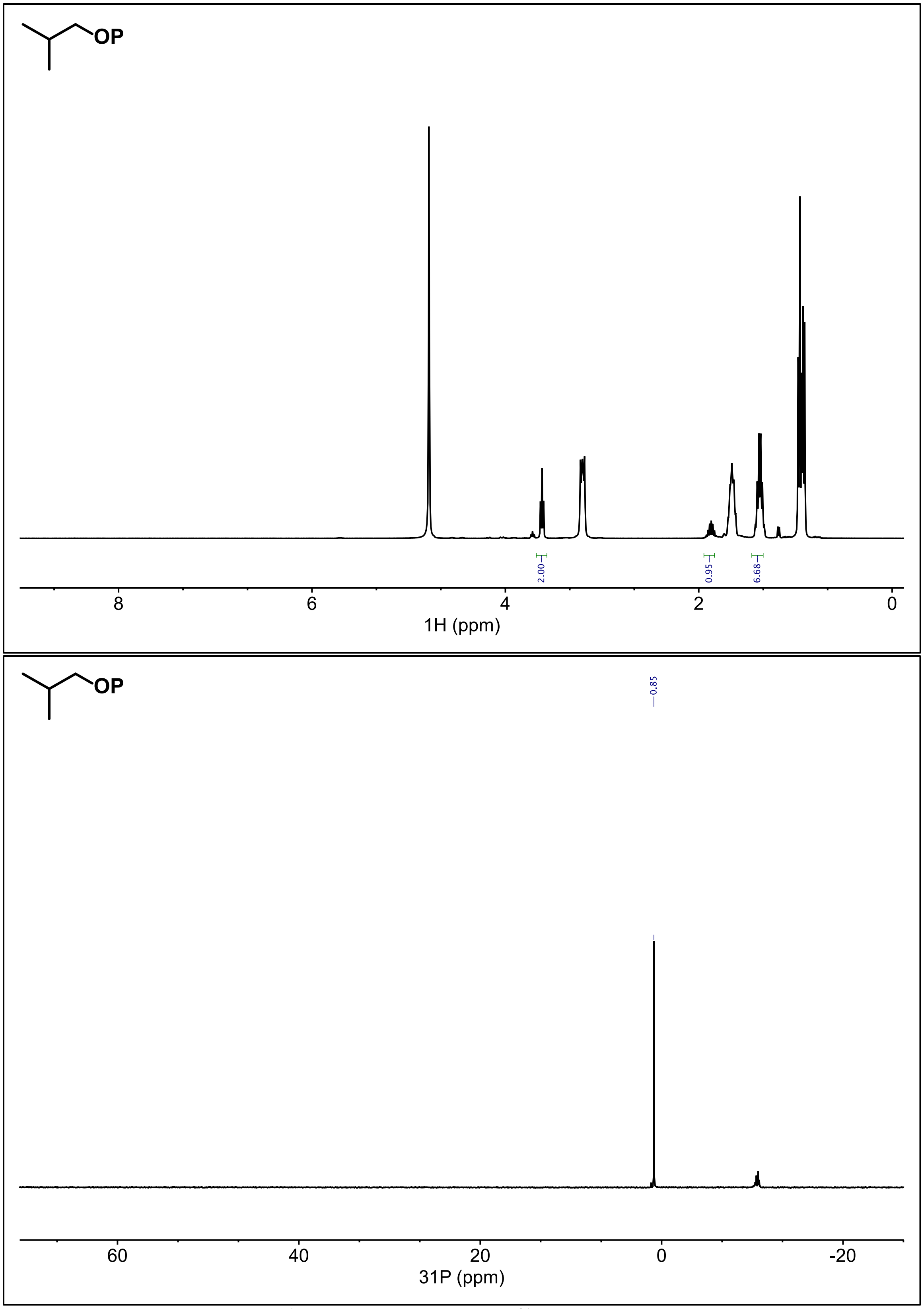

Figure S10. ^{1}H NMR (400 MHz, D_2O) ^{31}P NMR of **5** (162 MHz, D_2O)

Butyl Phosphate (6):
The title product was obtained as a white solid from butan-1-ol following the procedure described in *Method 2.7b.*
TLC (^{i}PrOH: NH_4OH: H_2O 8:1:1 v/v): R_f = 0.68.
^{1}H NMR (300 MHz, D_2O): δ 3.74 (q, J = 6.5 Hz, 2H), 1.52 (p, J = 6.9 Hz, 2H), 1.29 (dp, J = 13.0, 6.8, 6.2 Hz, 2H), 0.85 (t, J = 7.2 Hz, 3H).
^{31}P NMR (122 MHz, D_2O): δ 2.24.
HRMS-ESI: Calculated for $C_4H_{10}O_4P$ $[M-H]^-$: 153.03166; Found: 153.0310.

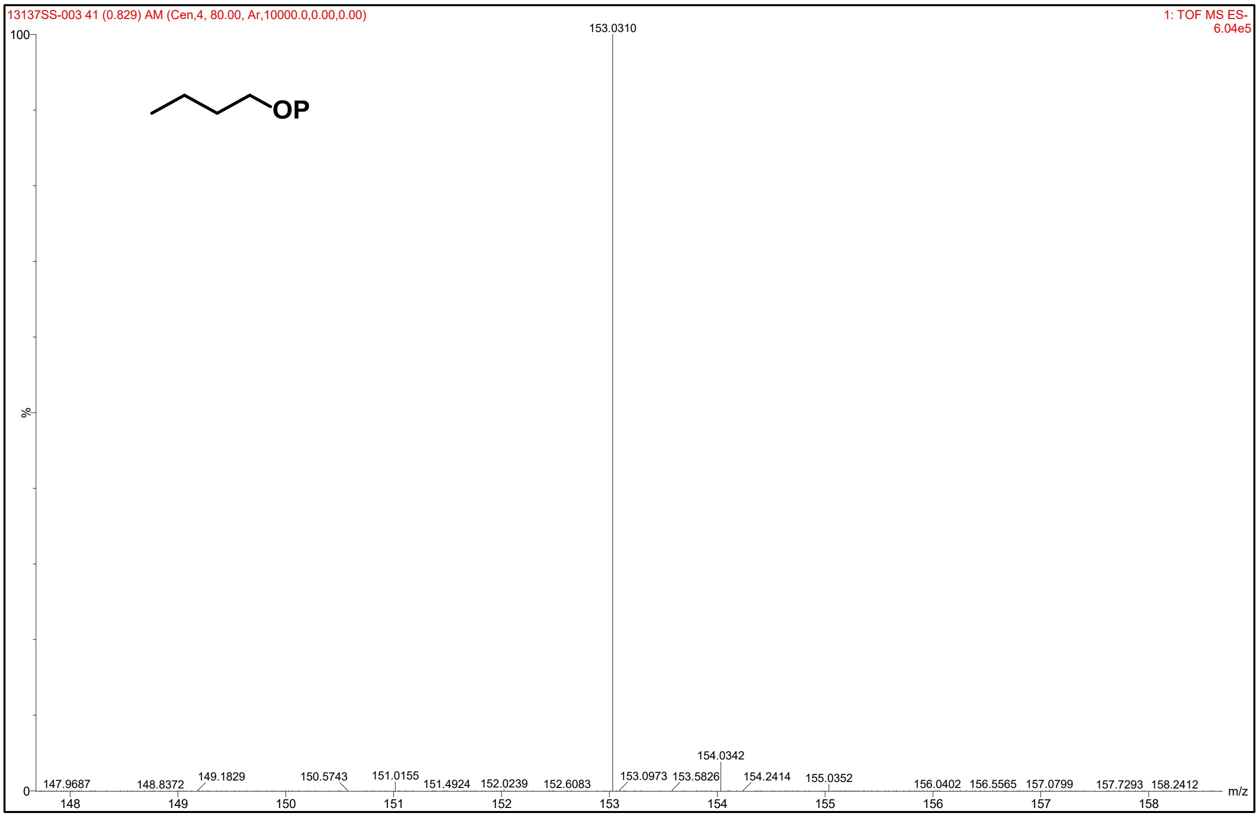

Figure S11. HRMS-ESI$^-$ of **6.**

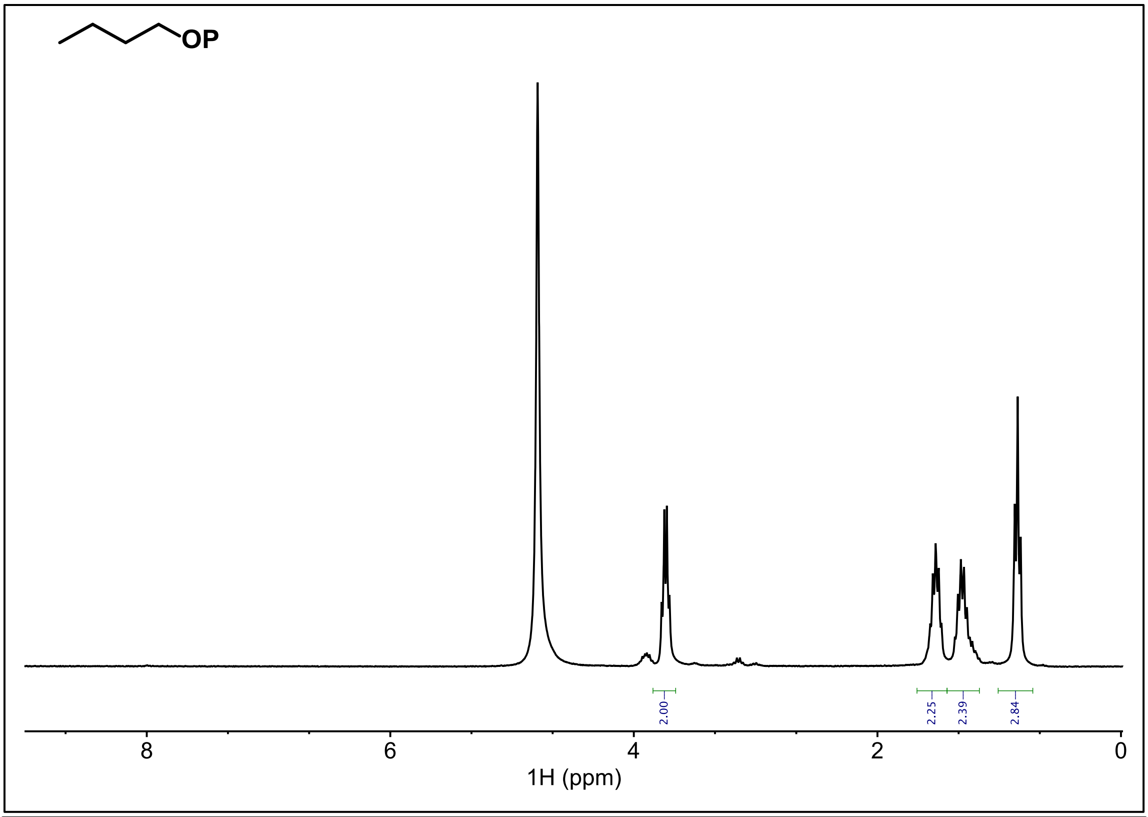

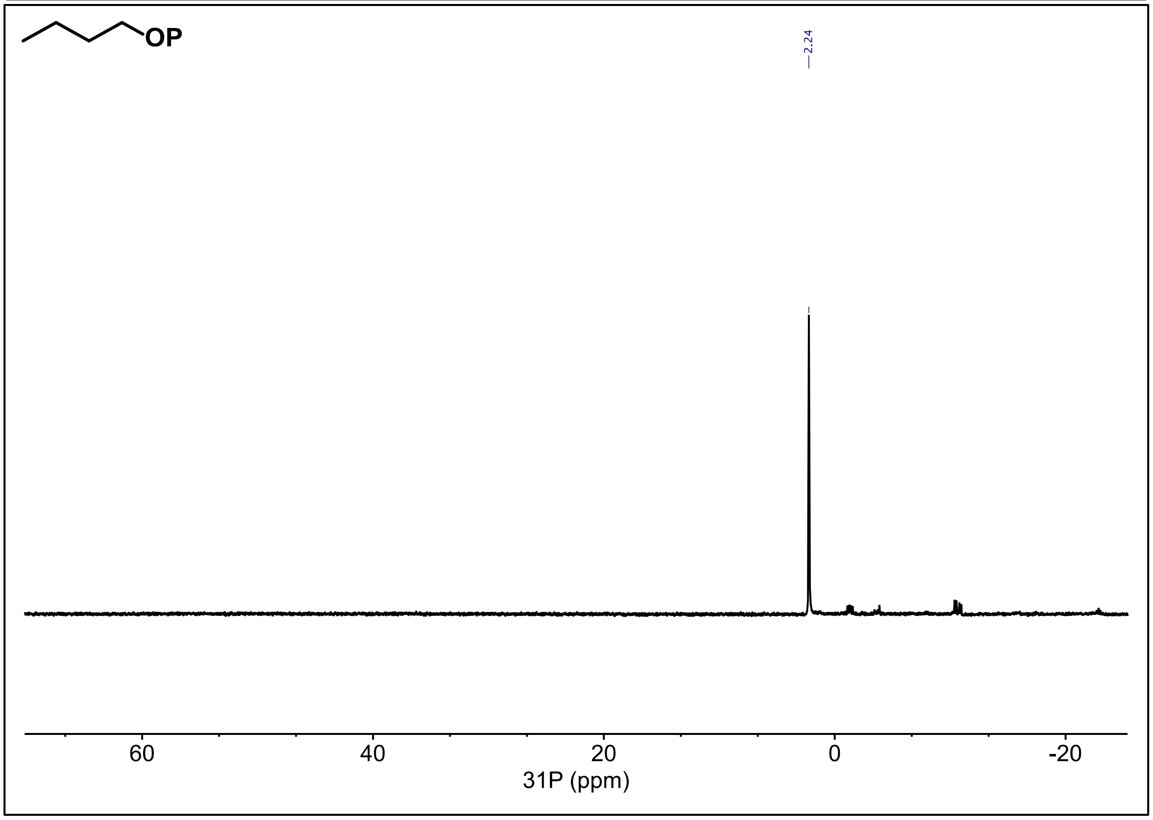

Figure S12. ^{1}H NMR (300 MHz, D_2O) and ^{31}P NMR of **6** (122 MHz, D_2O)**.**

But-3-en-1-yl Phosphate (7):

The title product was obtained as an ivory solid from but-3-en-1-ol following the procedure described in *Method 2.7b.*

TLC (^{i}PrOH: NH_4OH: H_2O 7:2:1 v/v): R_f = 0.68.

^{1}H NMR (400 MHz, D_2O): δ 5.90 (ddt, *J* = 17.1, 10.4, 6.7 Hz, 1H), 5.28 – 4.97 (m, 2H), 3.91 (q, *J* = 6.6 Hz, 2H), 2.69 – 2.10 (m, 2H).

^{31}P NMR (162 MHz, D_2O): δ 0.94.

HRMS-ESI: Calculated for $C_4H_8O_4P$ $[M-H]^-$: 151.01601; Found: 151.0161.

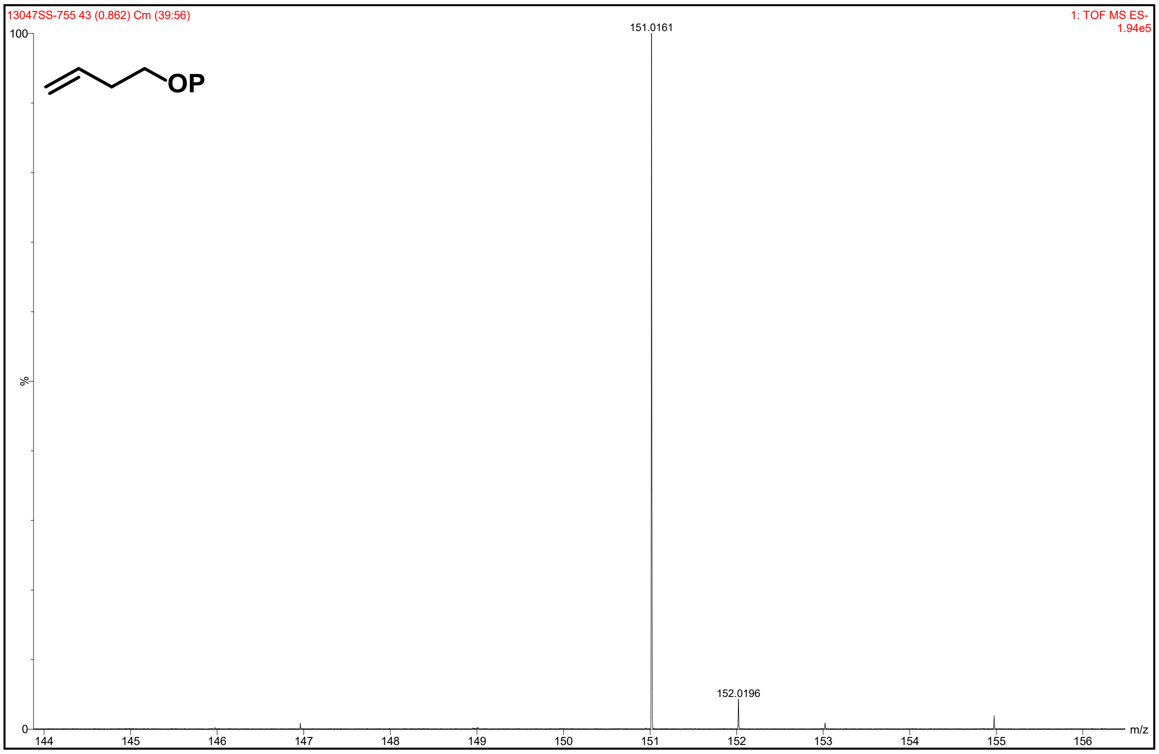

Figure S13. HRMS-ESI$^-$ of **7.**

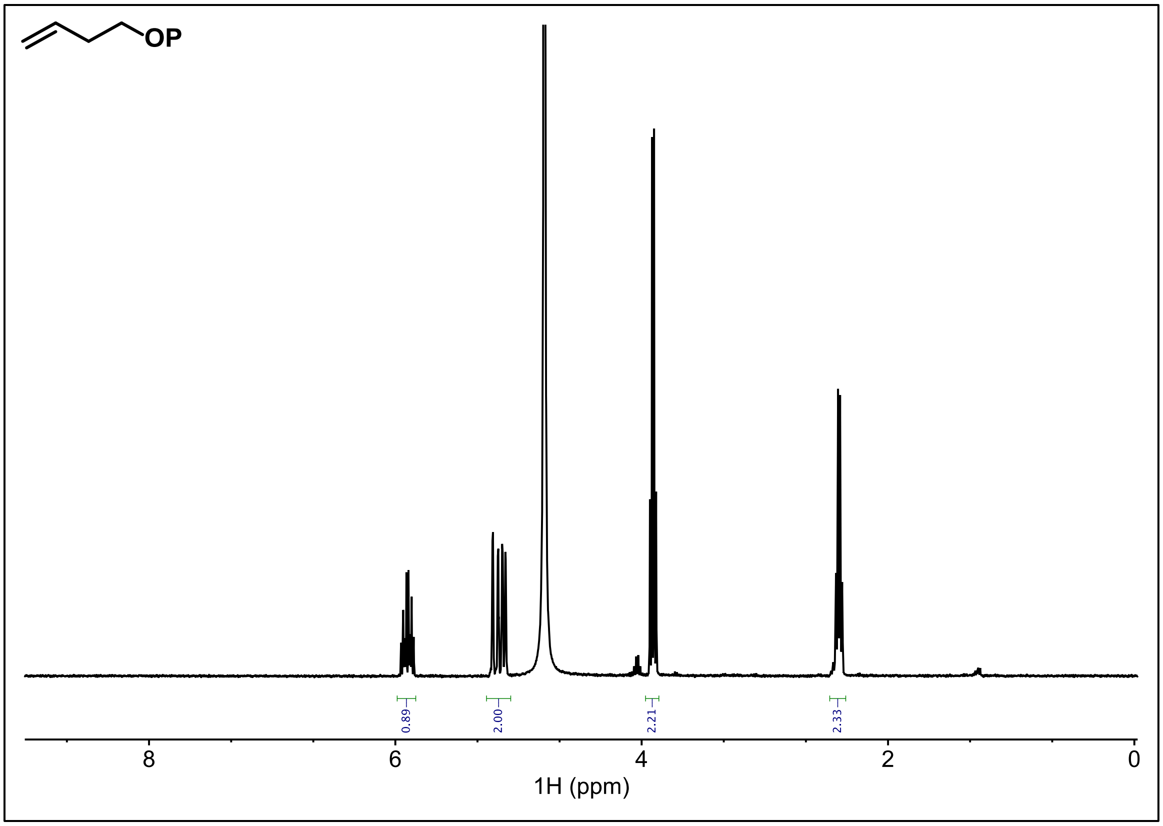

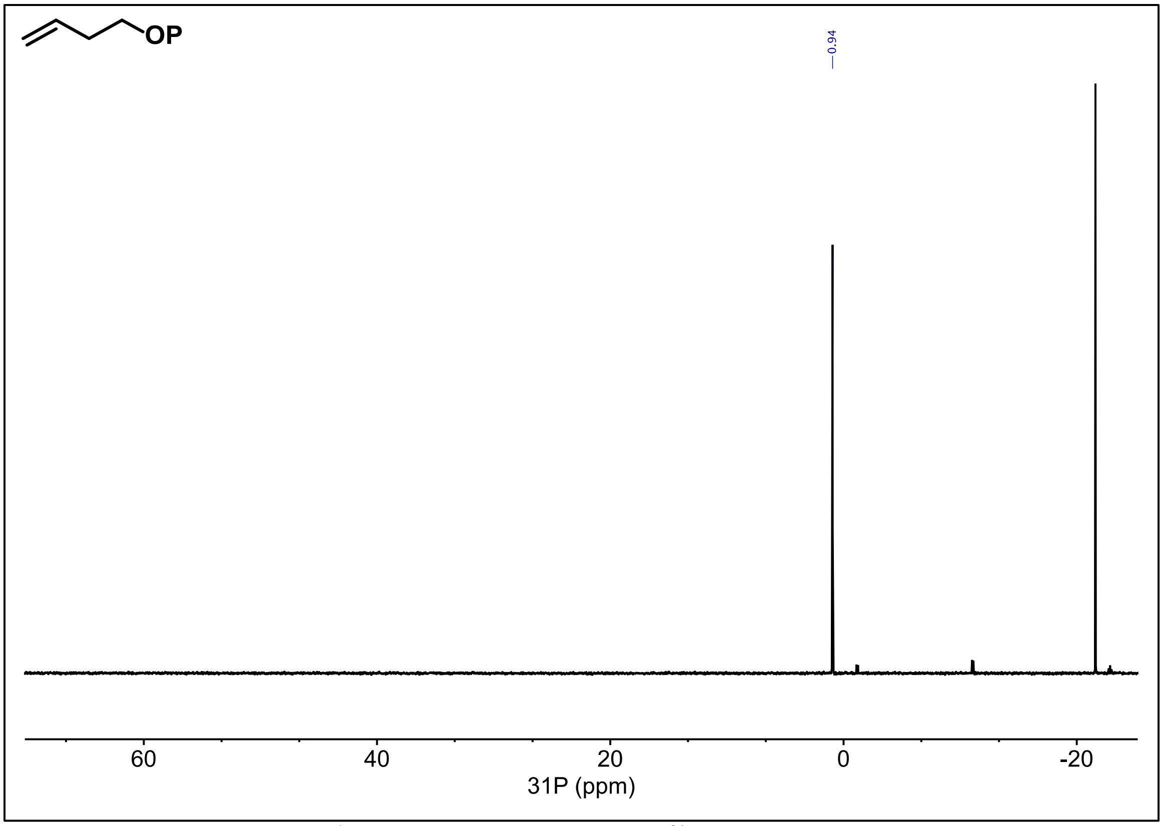

Figure S14. ^{1}H NMR (400 MHz, D_2O) and ^{31}P NMR of **7** (162 MHz, D_2O).

But-3-yn-1-yl Phosphate (8):

The title product was obtained as a yellow solid from but-3-yn-1-ol following the procedure described in *Method 2.7b*.

<u>TLC (^{i}PrOH: NH_4OH: H_2O 7:2:1 v/v):</u> R_f = 0.67.

<u>^{1}H NMR (400 MHz, D_2O):</u> δ 3.94 (d, J = 6.8 Hz, 2H), 2.56 (d, J = 2.6 Hz, 2H), 2.39 (t, J = 2.7 Hz, 1H).

<u>^{31}P NMR (162 MHz, D_2O):</u> δ 0.75.

<u>MS-ESI:</u> Calculated for $C_4H_6O_4P$ $[M-H]^-$: 149.00036; Found: 148.9996.

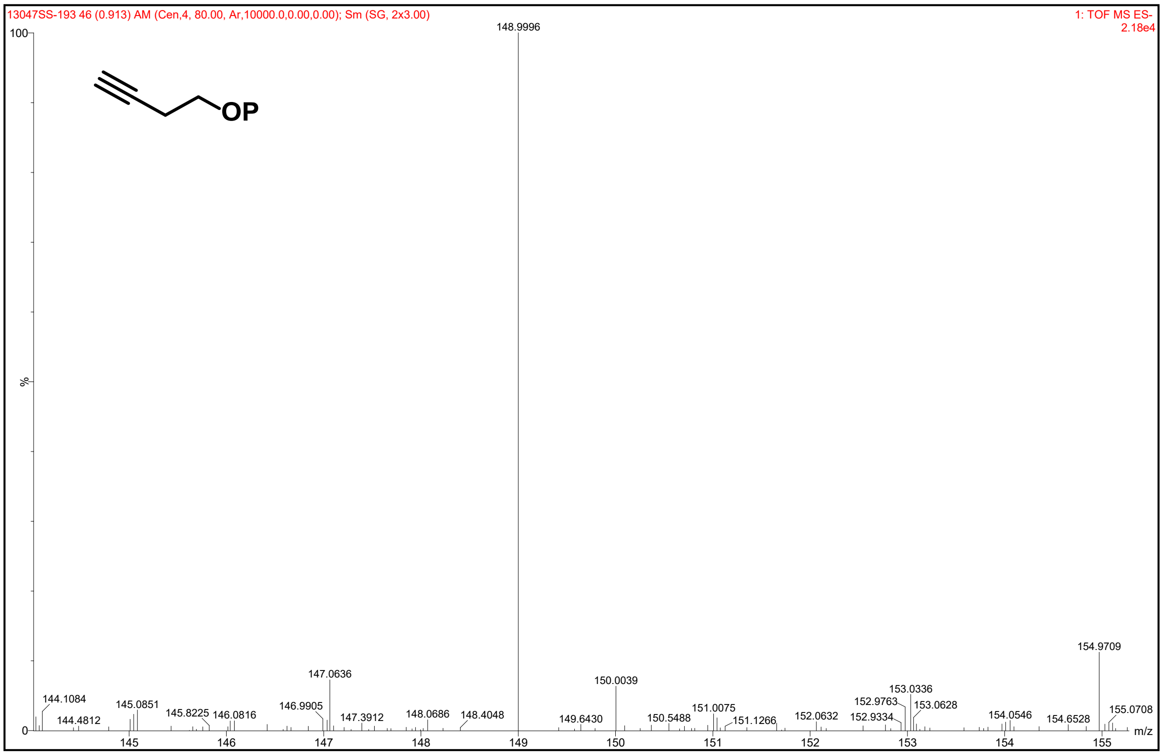

Figure S15. MS-ESI$^-$ of **8.**

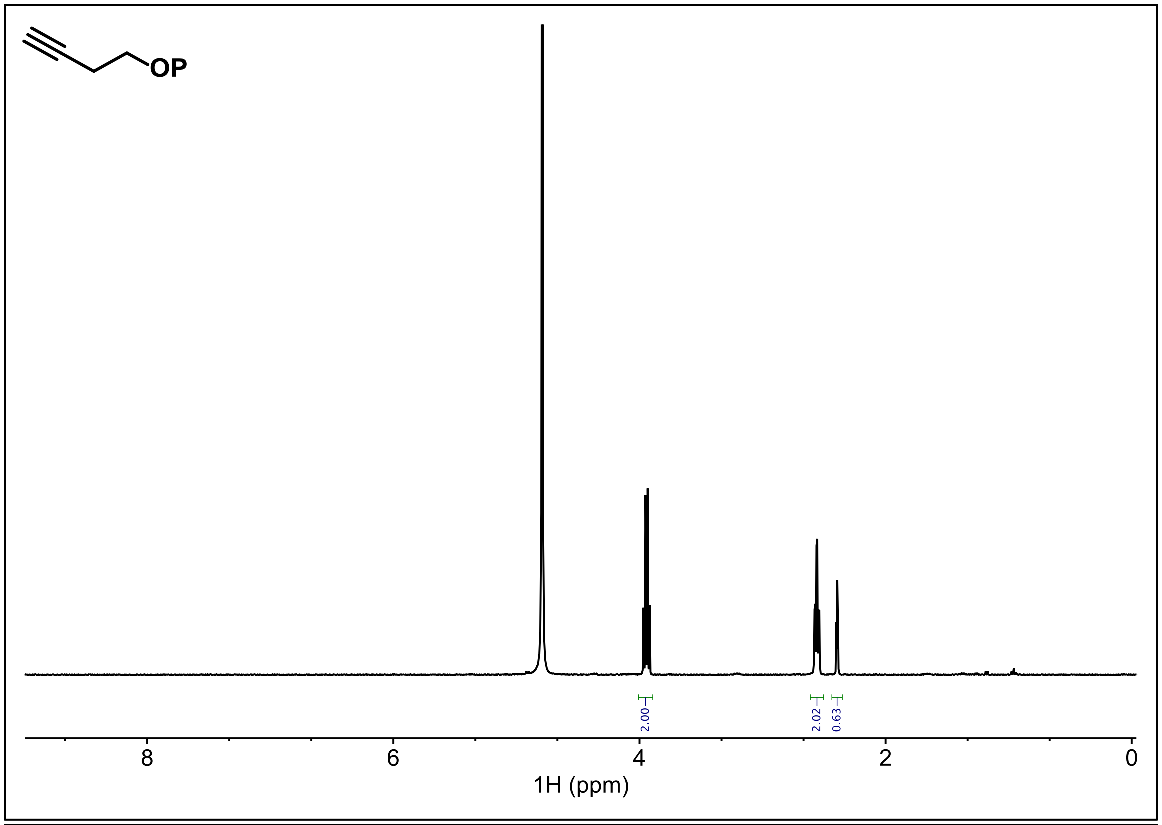

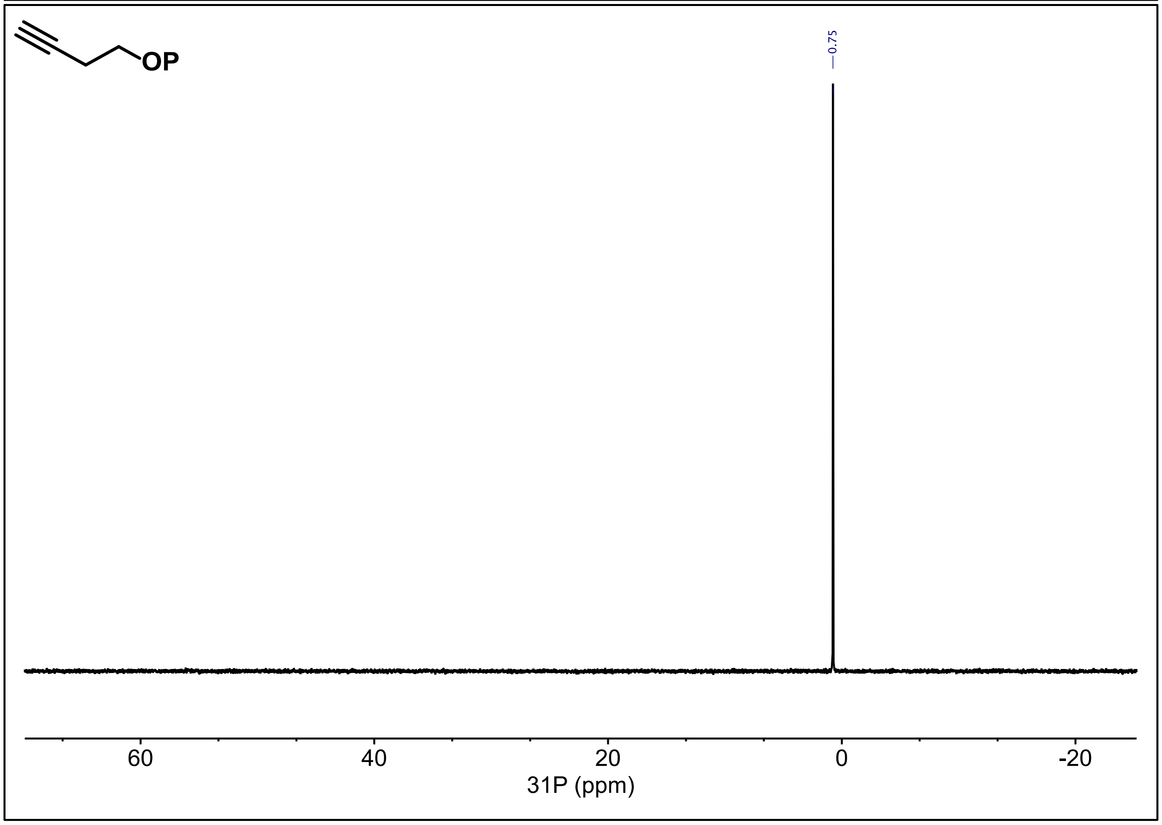

Figure S16. ^{1}H NMR (400 MHz, D_2O) and ^{31}P NMR of **8** (162 MHz, D_2O).

Isopentyl Phosphate (9):
The title product was obtained as a white solid from 3-methylbutan-1-ol following the procedure described in *Method 2.7b.*
TLC (^{i}PrOH: NH_4OH: H_2O 7:2:1 v/v): R_f = 0.64.
^{1}H NMR (400 MHz, D_2O): δ 3.87 (q, *J* = 6.7 Hz, 2H), 1.70 (hept, *J* = 13.5, 6.7 Hz, 1H), 1.51 (q, *J* = 6.9 Hz, 2H), 0.91 (s, 6H).
^{31}P NMR (162 MHz, D_2O): δ 1.47.
HRMS-ESI: Calculated for $C_5H_{12}O_4P$ $[M-H]^-$: 167.04731; Found: 167.0472.

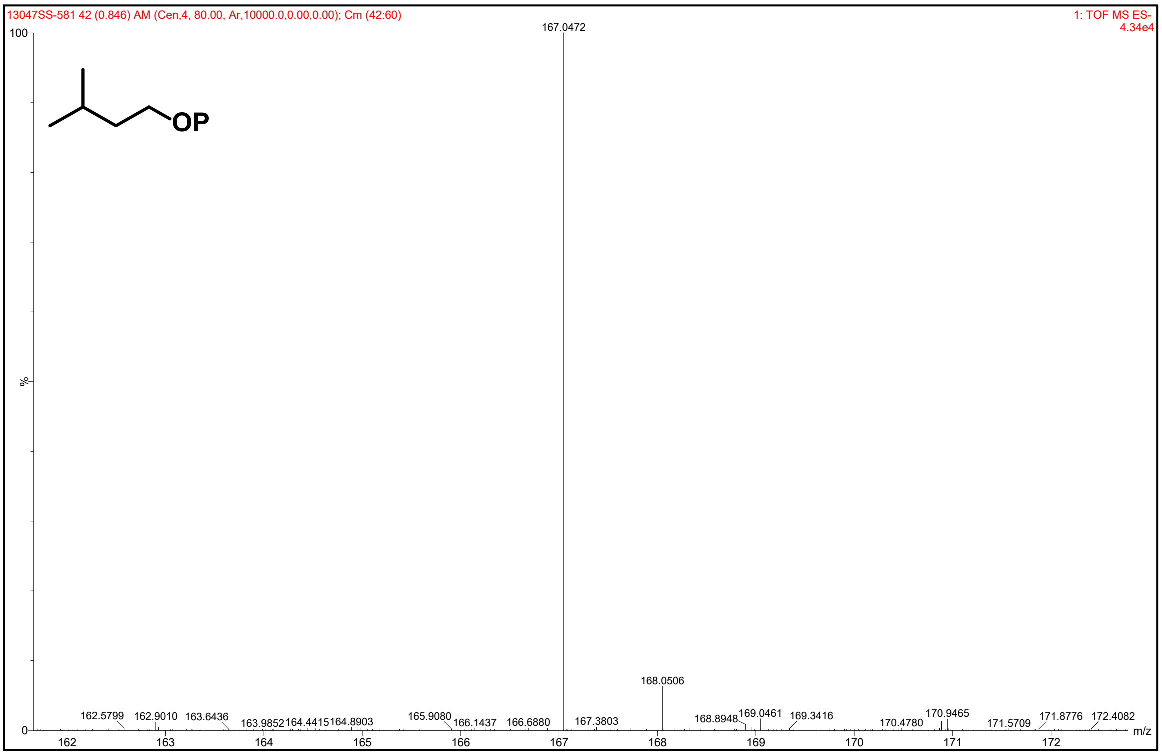

Figure S17. HRMS-ESI$^-$ of **9.**

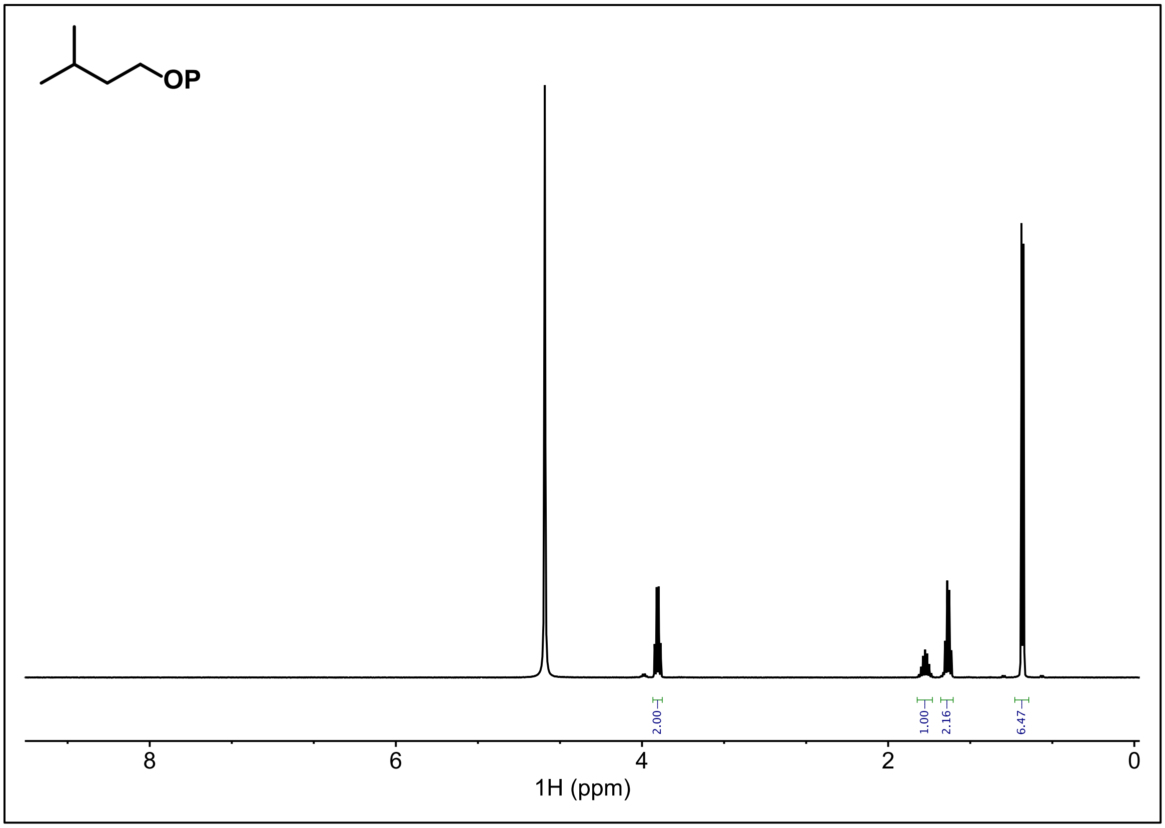

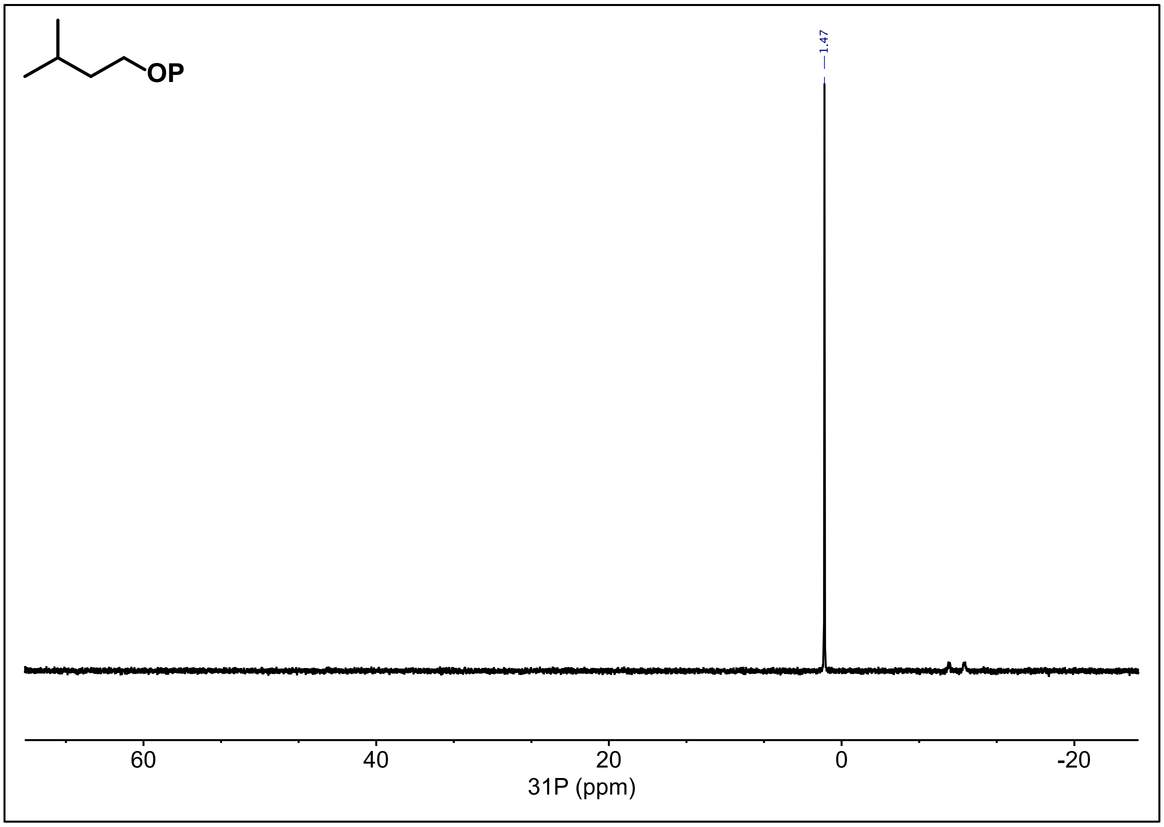

Figure S18. ^{1}H NMR (400 MHz, D_2O) and ^{31}P NMR of **9** (162 MHz, D_2O).

3-Azidopropyl Phosphate (10):
The title product was obtained as a brown solid from 3-azidopropan-1-ol following the procedure described in *Method 2.7b.*
TLC (^{i}PrOH: NH_4OH: H_2O 7:2:1 v/v): R_f = 0.64.
^{1}H NMR (400 MHz, D_2O): δ 3.92 (q, J = 6.3 Hz, 1H), 3.46 (t, J = 6.8 Hz, 1H), 1.92 (q, J = 6.4 Hz, 1H).
^{31}P NMR (162 MHz, D_2O): δ 1.06.
HRMS-ESI: Calculated for $C_3H_7N_3O_4P$ $[M-H]^-$: 180.01741; Found: 180.0177.

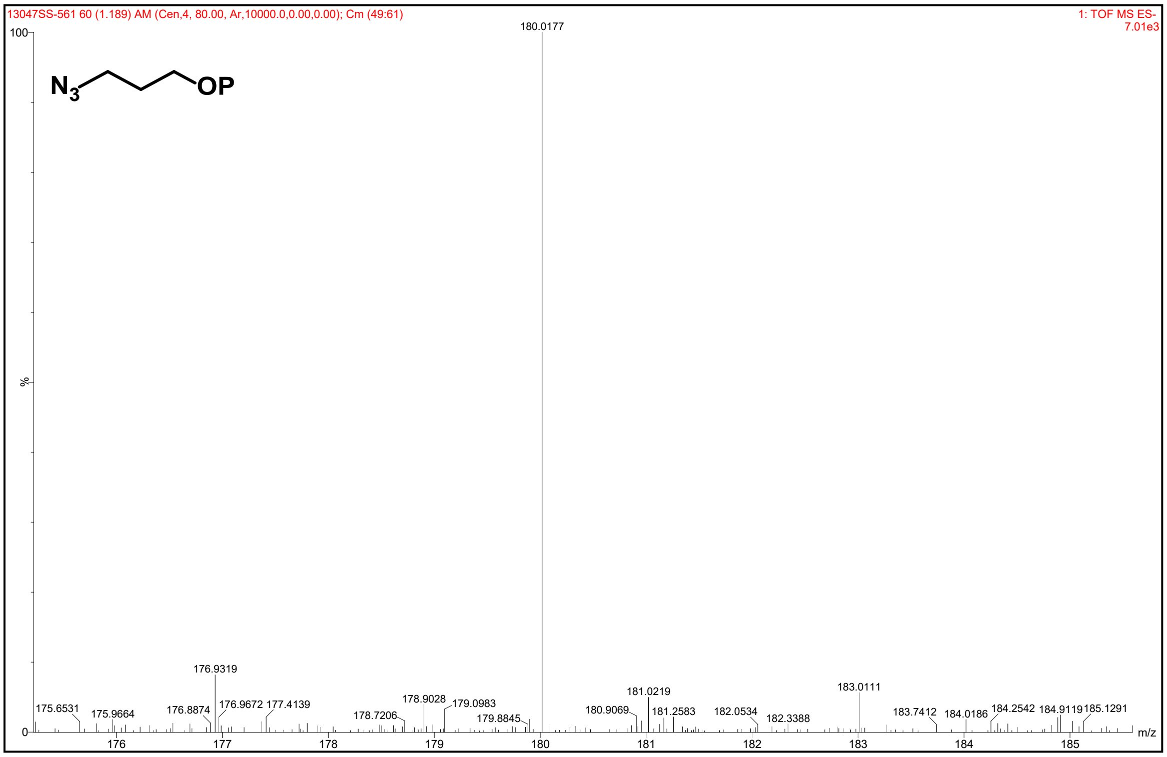

Figure S19. HRMS-ESI$^-$ of **10.**

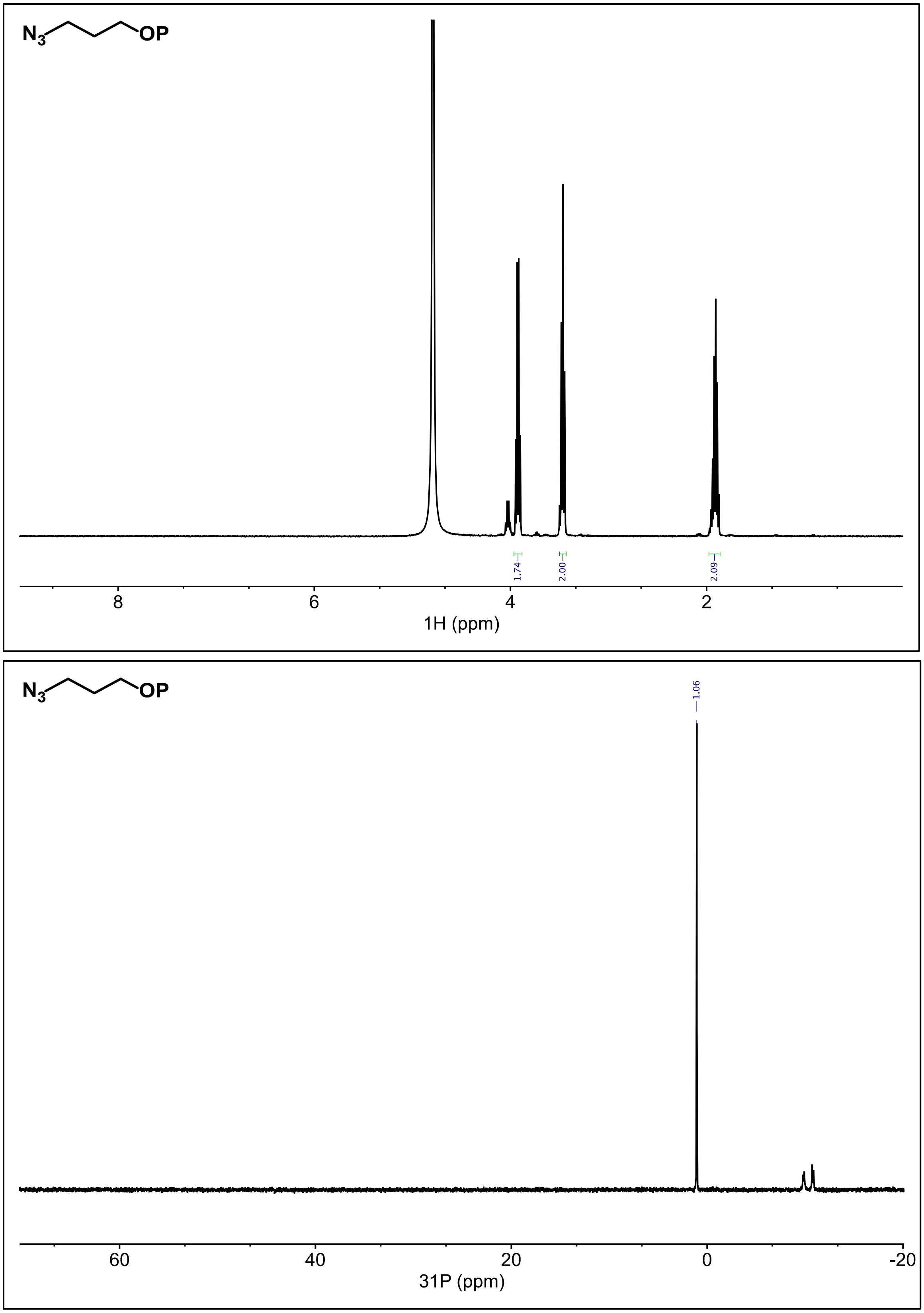

Figure S20. ^{1}H NMR (400 MHz, D_2O) and ^{31}P NMR of **10** (162 MHz, D_2O)**.**

Pentyl Phosphate (11):
The title product was obtained as a white solid from pentan-1-ol following the procedure described in *Method 2.7b.*
TLC (^{i}PrOH: NH_4OH: H_2O 6:2.5:0.5 v/v): R_f = 0.67.
^{1}H NMR (300 MHz, D_2O): δ 7.52 – 7.23 (m, 2H), 7.11 – 6.80 (m, 2H), 4.86 (d, J = 6.1 Hz, 2H), 3.83 (s, 3H).
^{31}P NMR (122 MHz, D_2O): δ 1.78.
HRMS-ESI: Calculated for $C_5H_{12}O_4P$ $[M-H]^-$: 167.04731; Found: 167.0474.

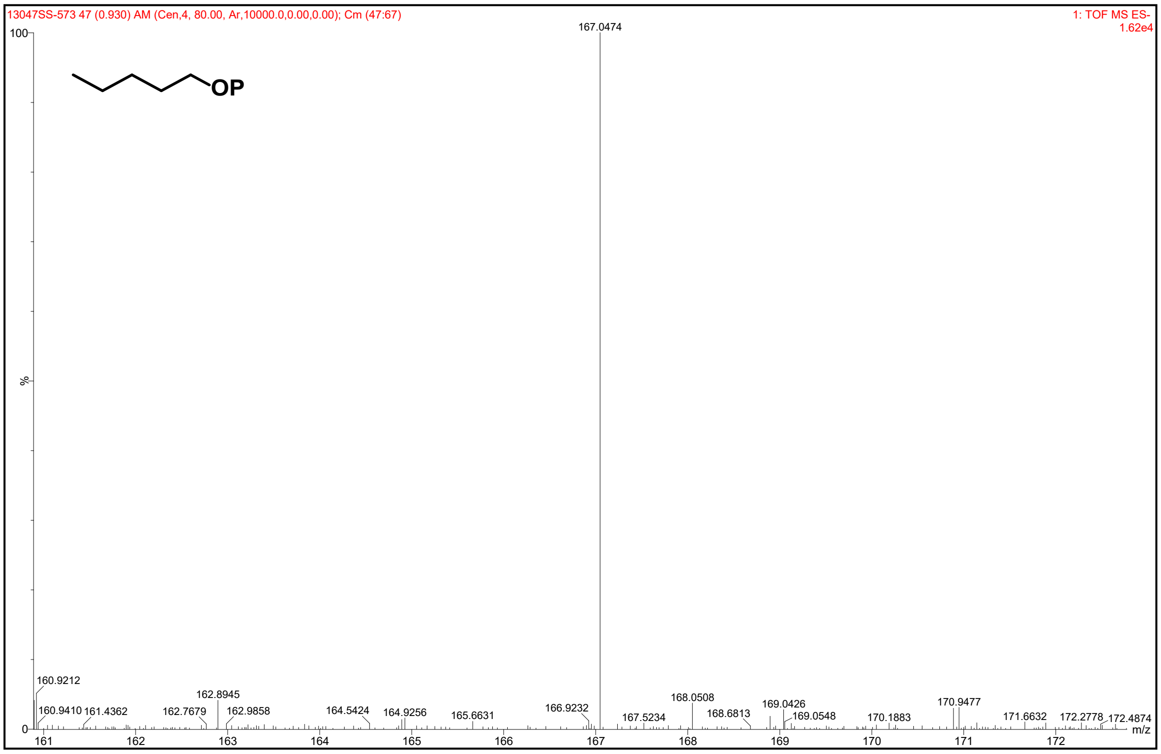

Figure S21. HRMS-ESI$^-$ of **11.**

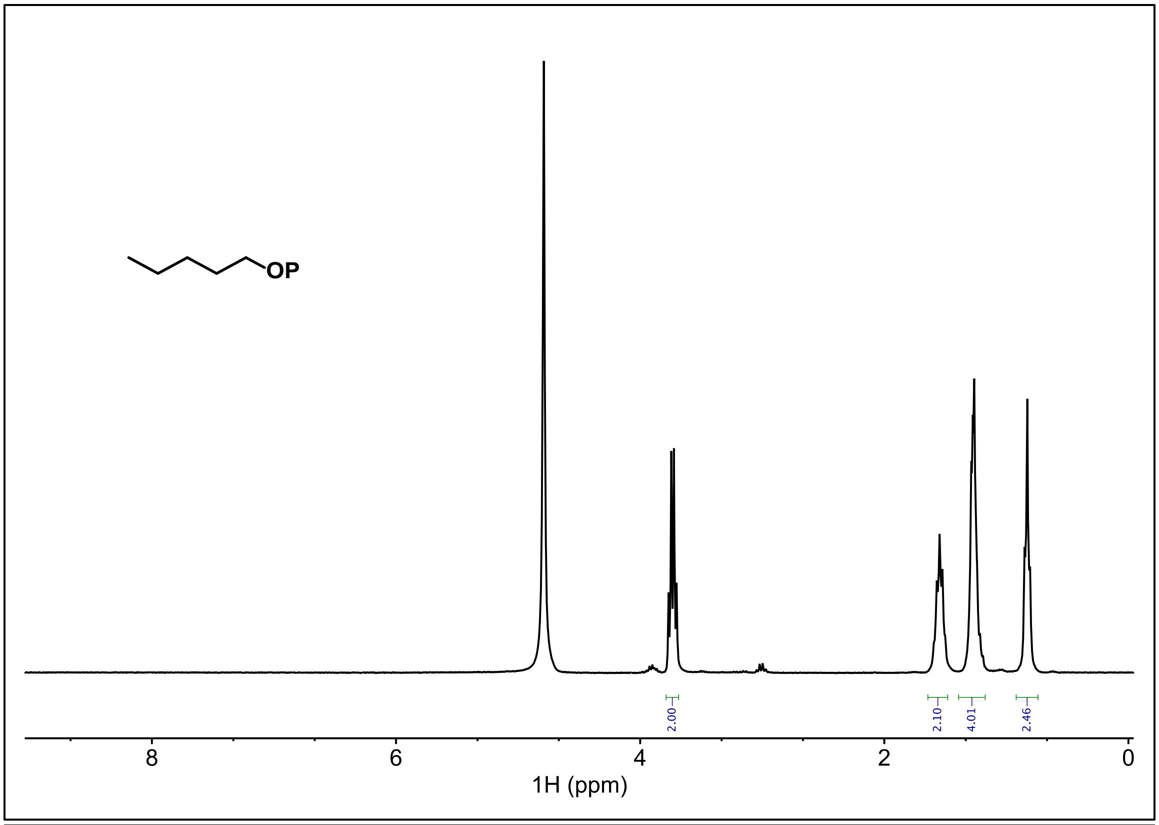

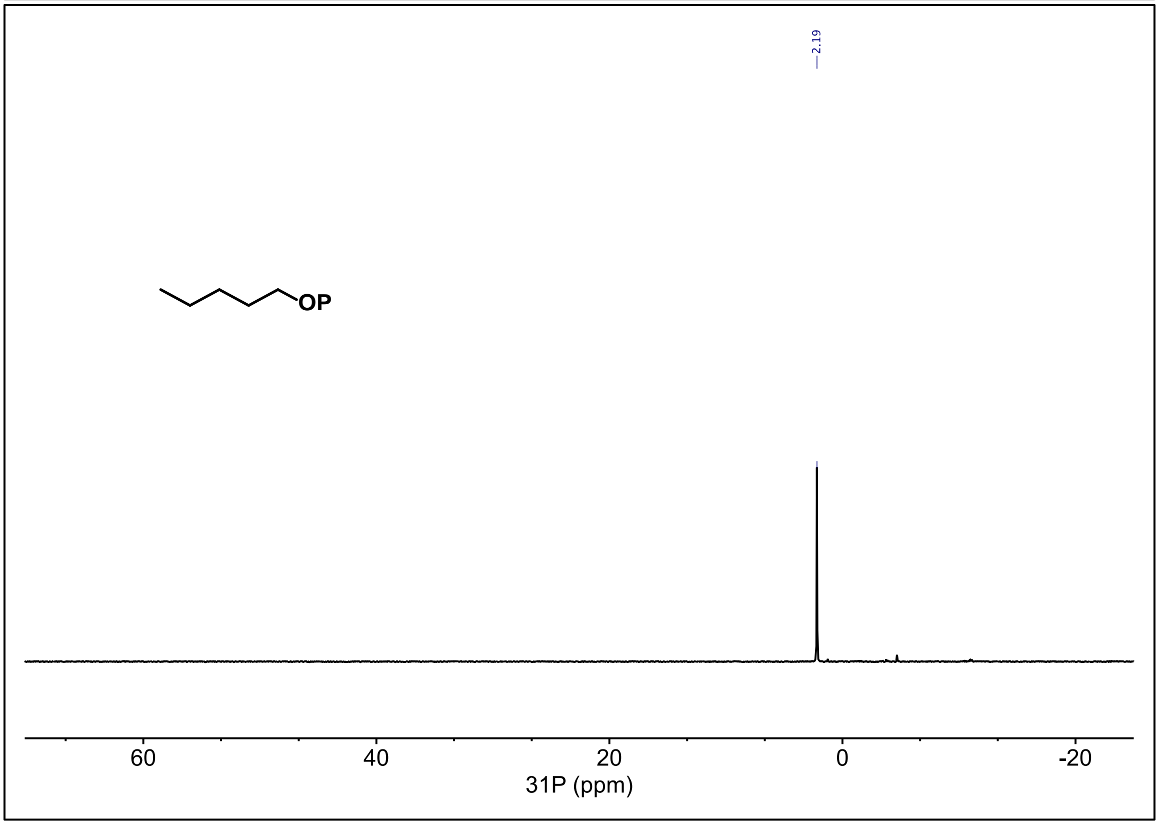

Figure S22. ^{1}H NMR (300 MHz, D_2O) and ^{31}P NMR of **11** (122 MHz, D_2O).

Allyl Phosphate (12):

The title product was obtained as an ivory solid from prop-2-en-1-ol following the procedure described in *Method 2.7a*.

<u>TLC (^{i}PrOH: NH_4OH: H_2O 5:4:1 v/v):</u> R_f = 0.65.

<u>^{1}H NMR (300 MHz, D_2O):</u> δ 5.92 (ddq, *J* = 15.9, 10.3, 5.1 Hz, 1H), 5.28 (d, *J* = 17.4 Hz, 2H), 5.14 (t, *J* = 7.1 Hz, 2H), 4.27 (d, *J* = 6.6 Hz, 2H).

<u>^{31}P NMR (122 MHz, D_2O):</u> δ 1.16.

<u>HRMS-ESI:</u> Calculated for $C_3H_6O_4P$ $[M-H]^-$: 137.00036; Found: 137.0001.

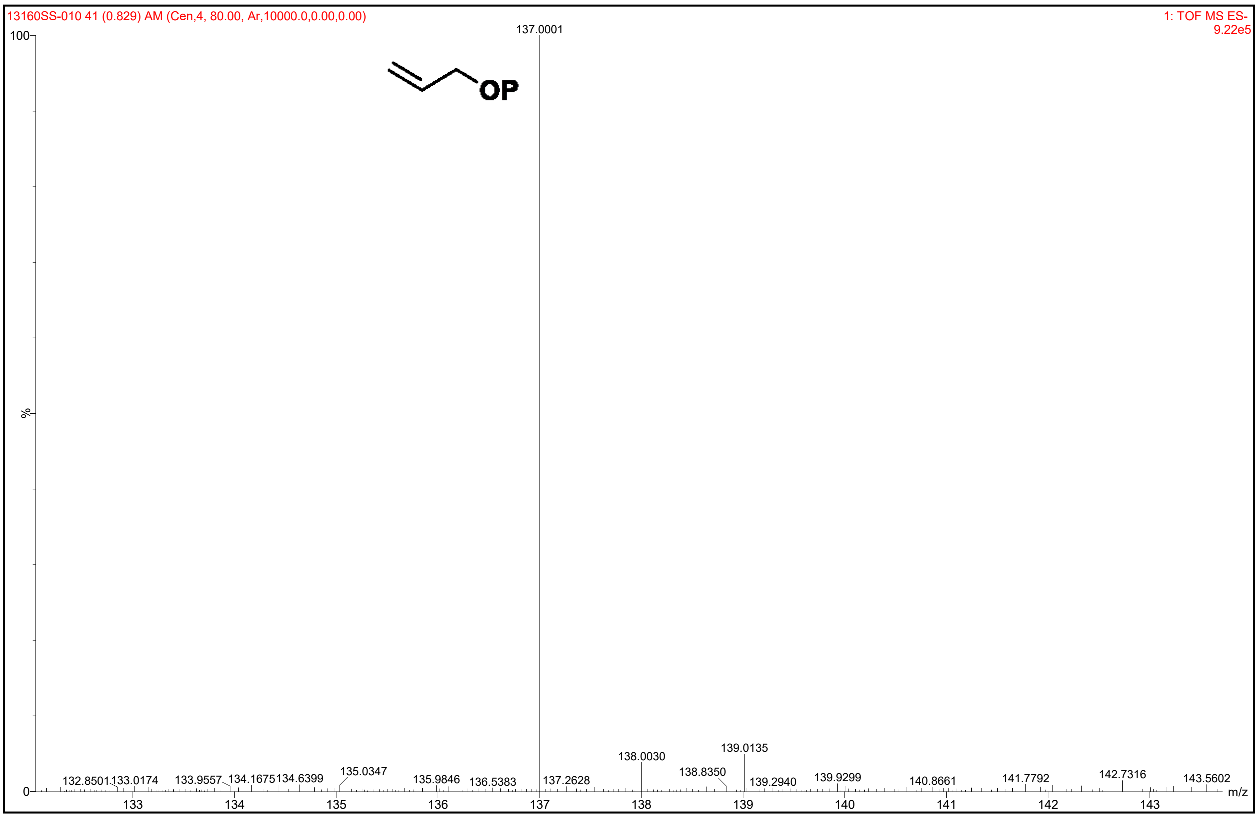

Figure S23. HRMS-ESI$^-$ of **12.**

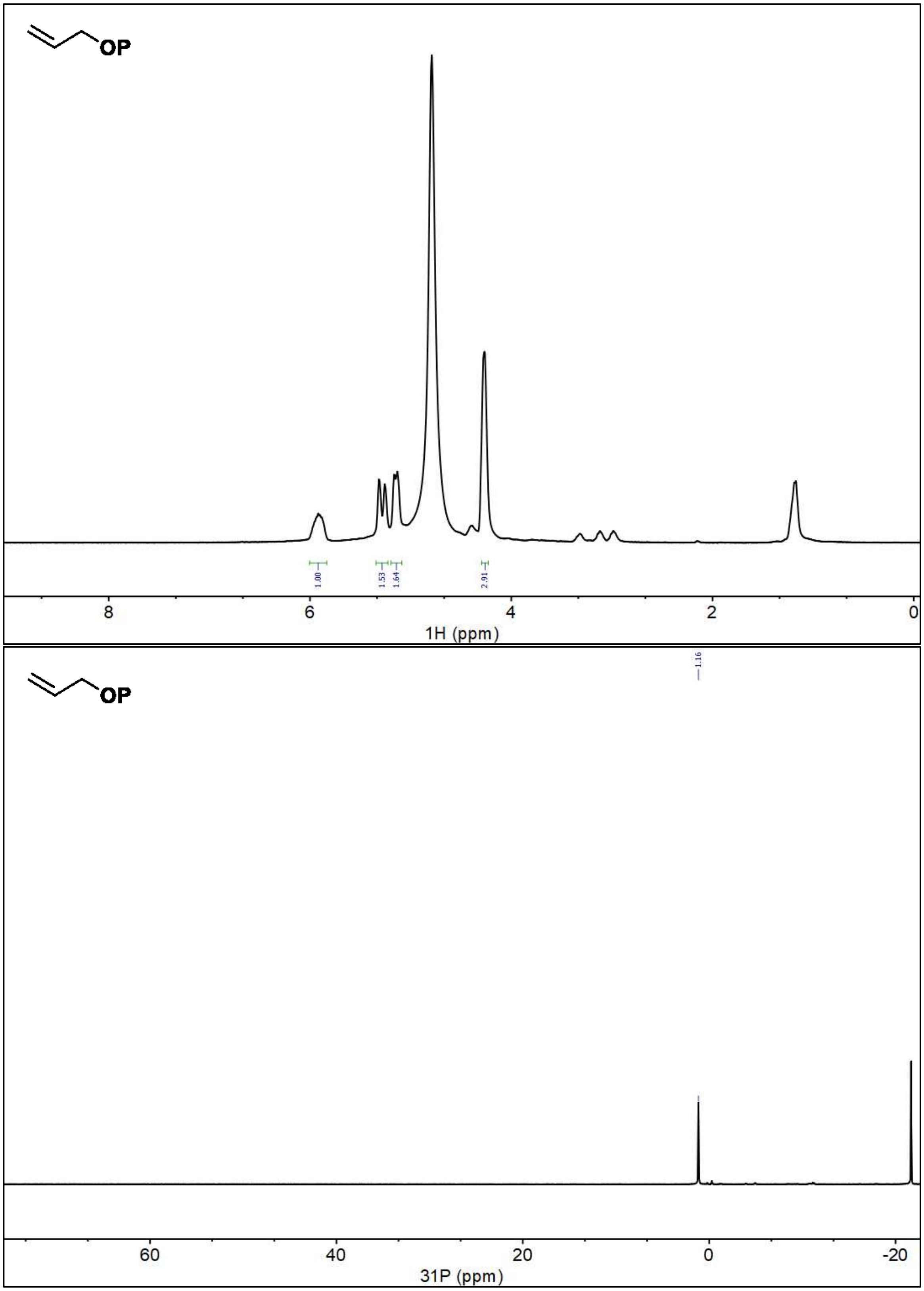

Figure S24. ^{1}H NMR (300 MHz, D_2O) and ^{31}P NMR of **12** (122 MHz, D_2O).

But-2-en-1-yl Phosphate (13):
The title product was obtained as a white solid from (*E*)-but-2-en-1-ol following the procedure described in *Method 2.7a.*
TLC (^{i}PrOH: NH_4OH: H_2O 7:2:1 v/v): R_f = 0.64.
^{1}H NMR (400 MHz, D_2O): δ 5.96 – 5.79 (m, 1H), 5.66 (dtd, *J* = 15.4, 6.3, 1.6 Hz, 1H), 4.30 (ddt, *J* = 7.4, 6.3, 1.2 Hz, 2H), 1.71 (dd, *J* = 6.5, 1.4 Hz, 3H).
^{31}P NMR (162 MHz, D_2O): δ 0.63.
HRMS-ESI: Calculated for $C_4H_8O_4P$ $[M-H]^-$: 151.01601; Found: 151.0159.

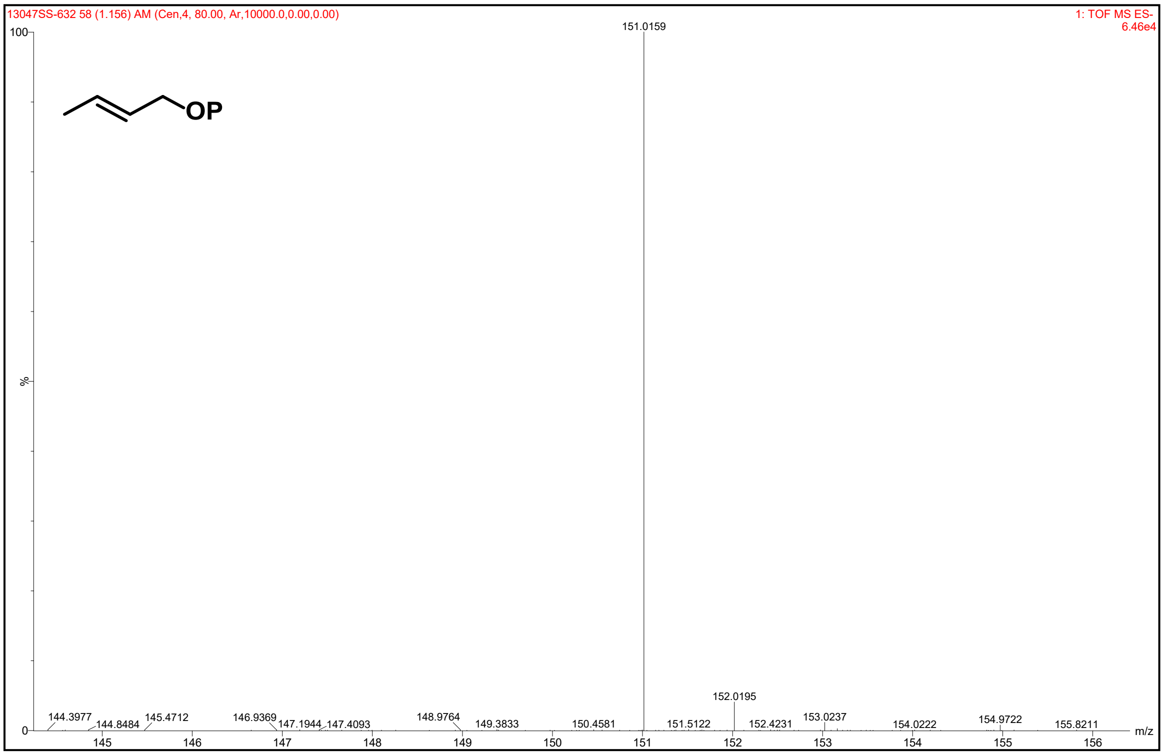

Figure S25. HRMS-ESI$^-$ of **13.**

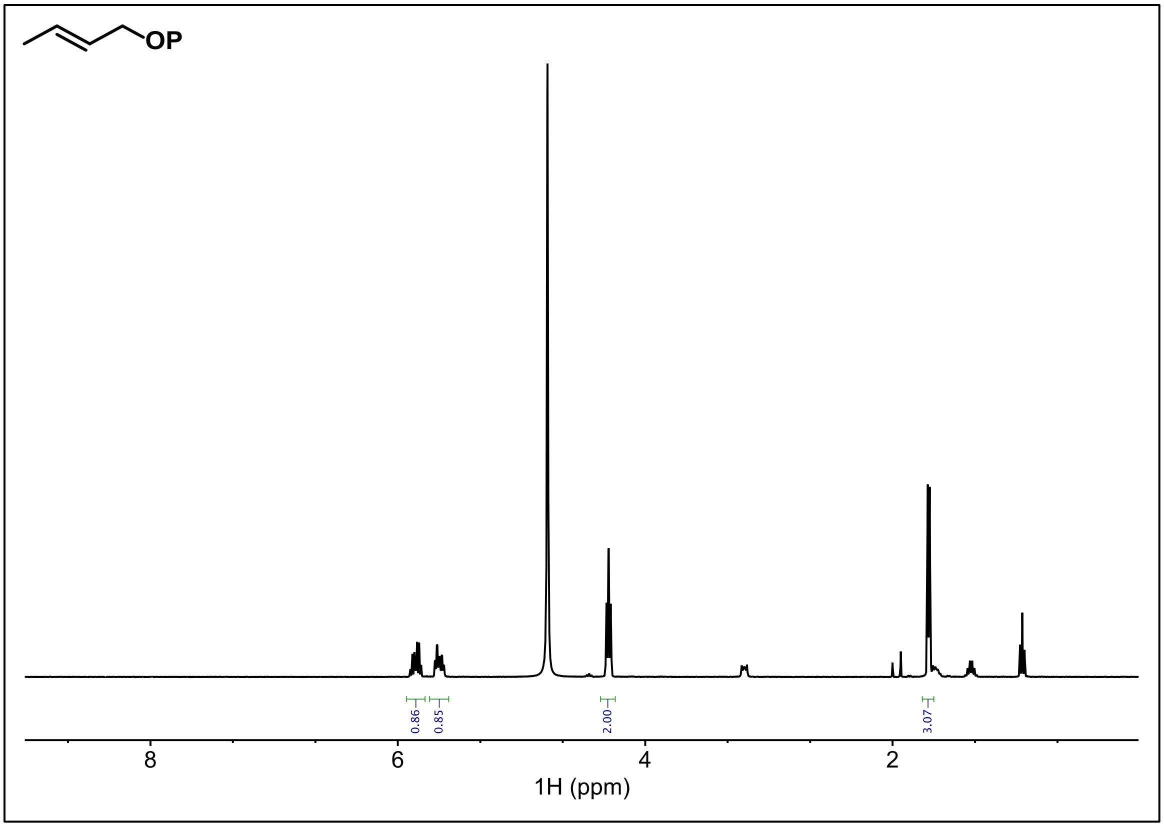

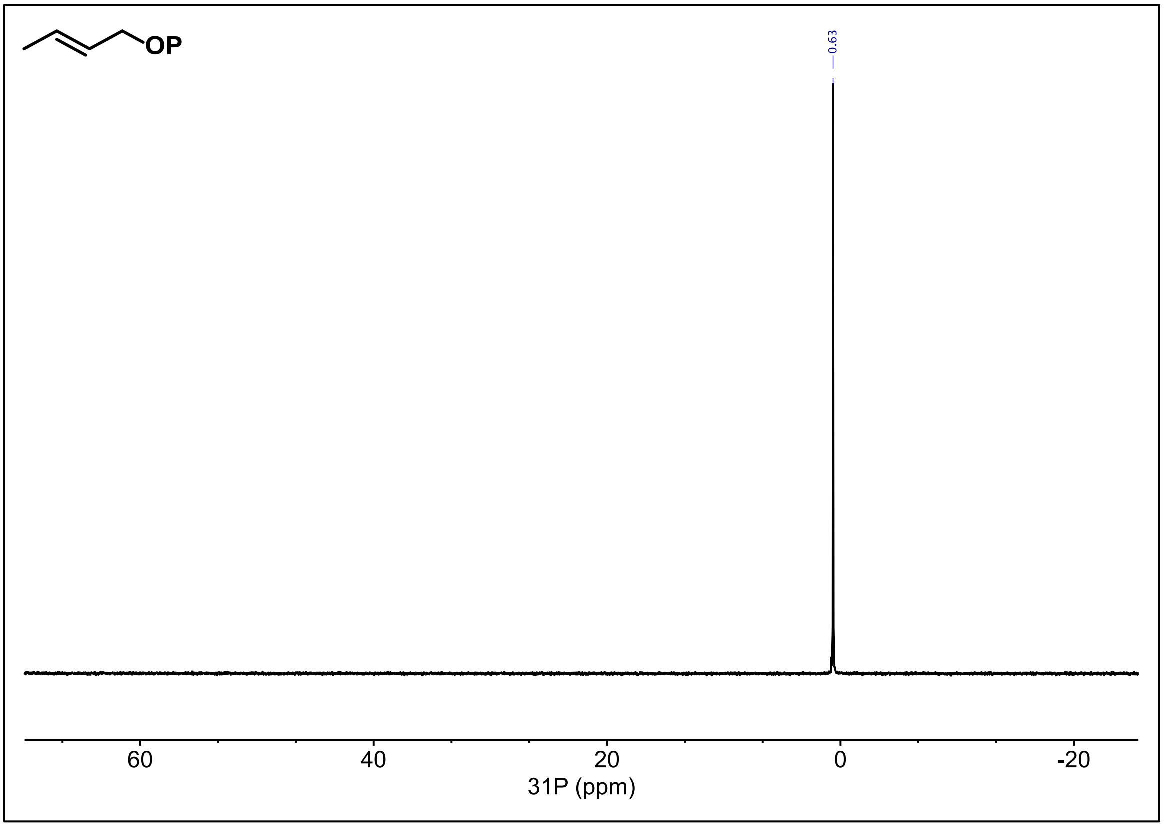

Figure S26. ^{1}H NMR (400 MHz, D_2O) and ^{31}P NMR of **13** (162 MHz, D_2O).

3-Chlorobut-2-en-1-yl Phosphate (14):
The title product was obtained as a yellow-white solid from (*Z*)-3-chlorobut-2-en-1-ol following the procedure described in *Method 2.7b.*
TLC (^{i}PrOH: NH_4OH: H_2O 7:2:1 v/v): R_f = 0.63.
^{1}H NMR (400 MHz, D_2O): δ 5.81 (td, *J* = 6.5, 1.3 Hz, 1H), 4.51 (ddd, *J* = 7.8, 6.6, 1.3 Hz, 2H), 2.16 (d, *J* = 1.2 Hz, 3H).
^{31}P NMR (162 MHz, D_2O): δ 0.81.
HRMS-ESI: Calculated for $C_4H_7ClO_4P$ $[M-H]^-$: 184.97704; Found: 184.9767.

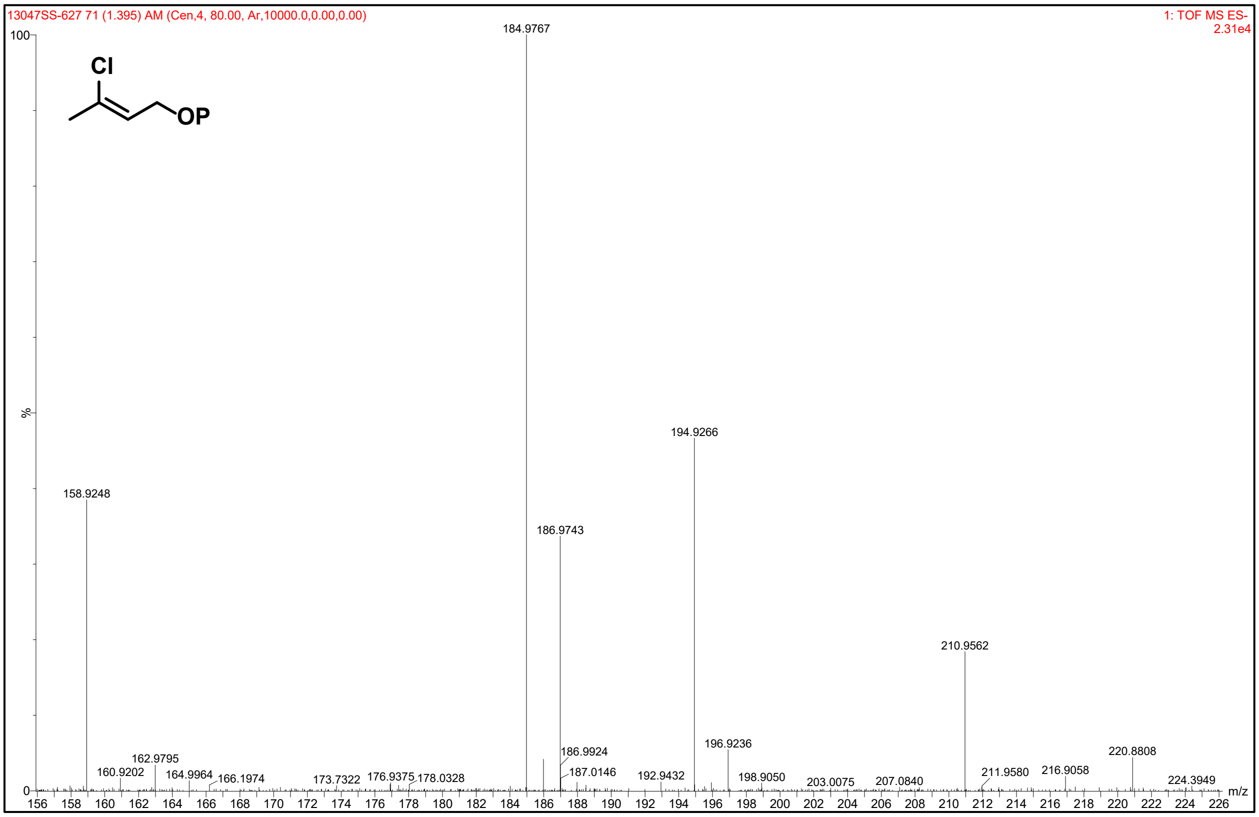

Figure S27. HRMS-ESI$^+$ of **14.**

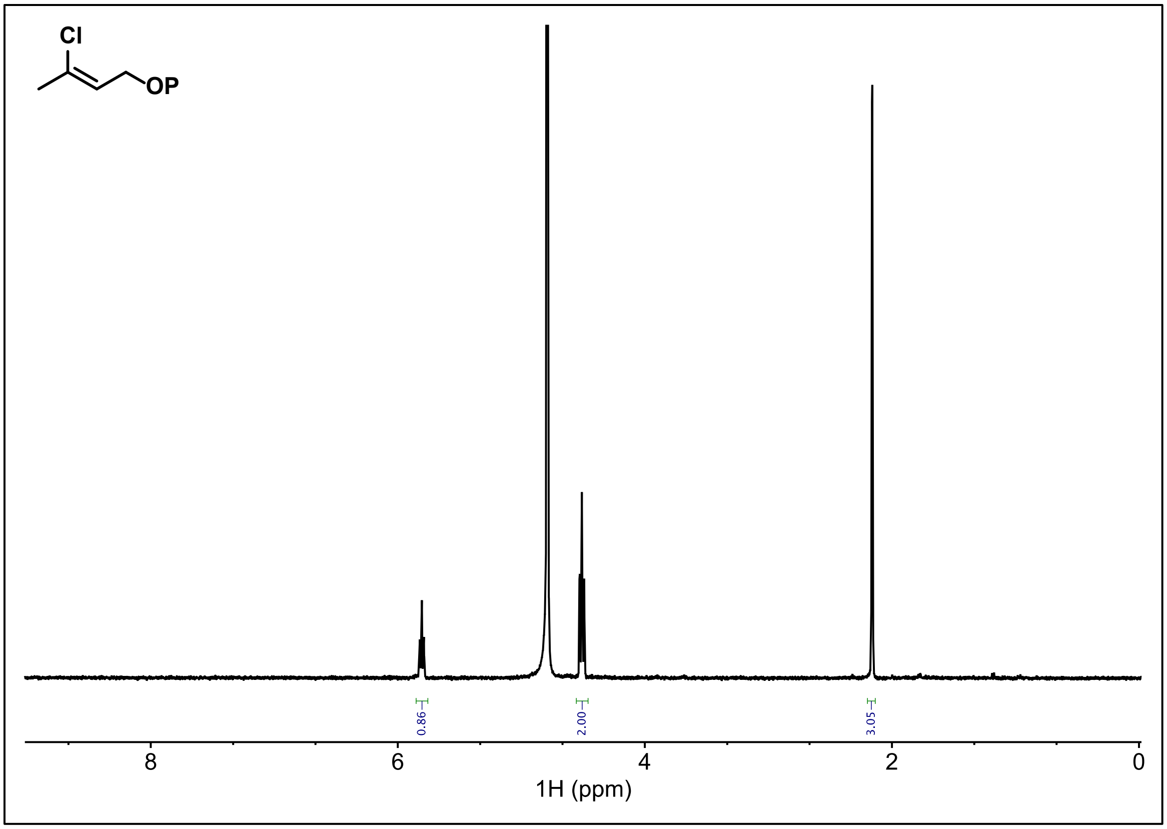

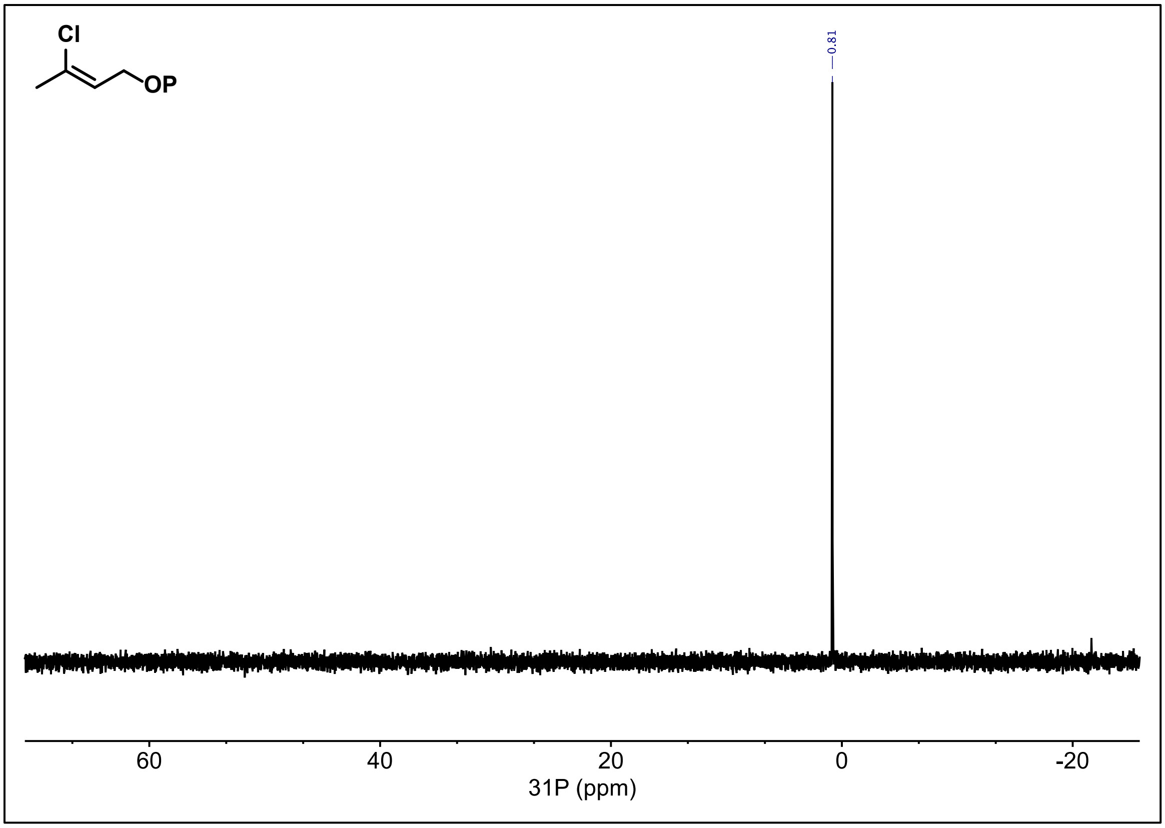

Figure S28. ^{1}H NMR (400 MHz, D_2O) and ^{31}P NMR of **14** (162 MHz, D_2O).

(*E*)-Pent-2-en-4-yn-1-yl Phosphate (15):

The title product was obtained as a brown solid from (E)-pent-2-en-4-yn-1-ol following the procedure described in *Method 2.7b.*

TLC (^{i}PrOH: NH_4OH: H_2O 7:2:1 v/v): R_f = 0.64.

^{1}H NMR (400 MHz, D_2O): δ 6.71 (dt, *J* = 16.9, 10.6 Hz, 1H), 6.19 (d, *J* = 11.1 Hz, 1H), 5.39 – 5.29 (m, 1H), 5.20 (d, *J* = 10.3 Hz, 1H), 4.30 (d, *J* = 6.6 Hz, 2H), 1.81 (s, 3H)

^{31}P NMR (162 MHz, D_2O): δ 1.08.

HRMS-ESI: Calculated for $C_5H_6O_4P$ $[M-H]^-$: 161.00036; Found: 161.0008.

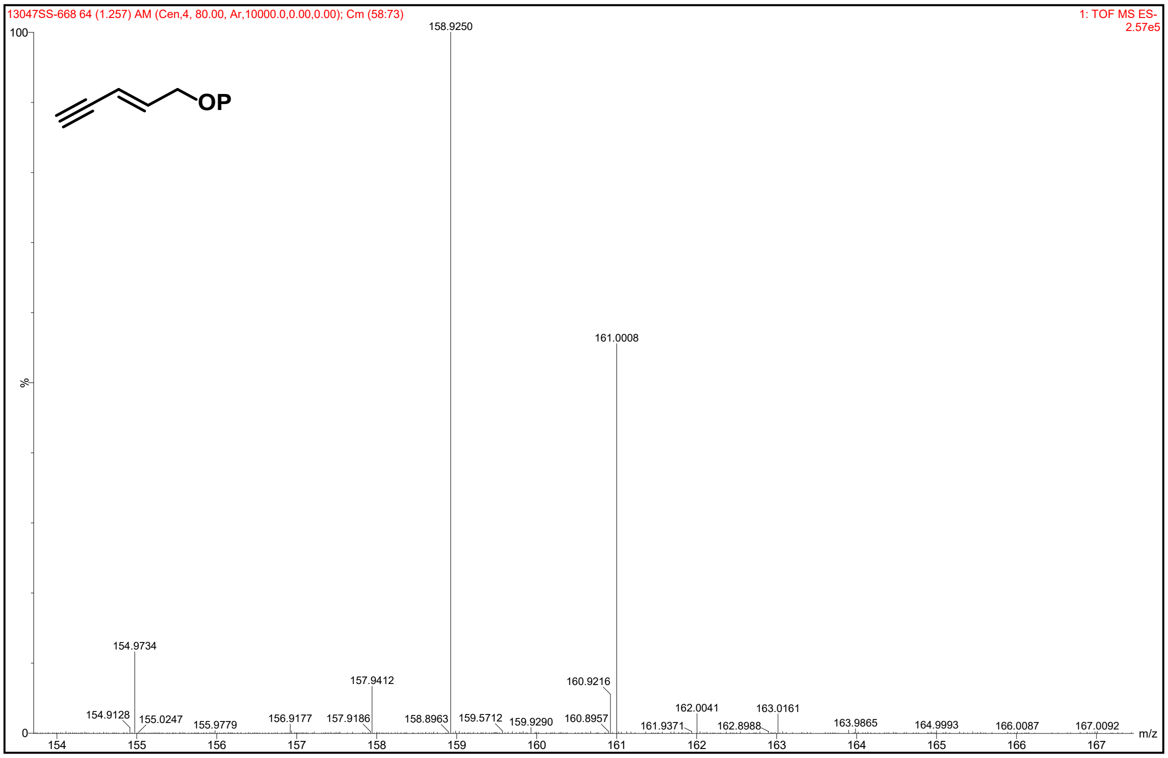

Figure S29. HRMS-ESI$^-$ of **15.**

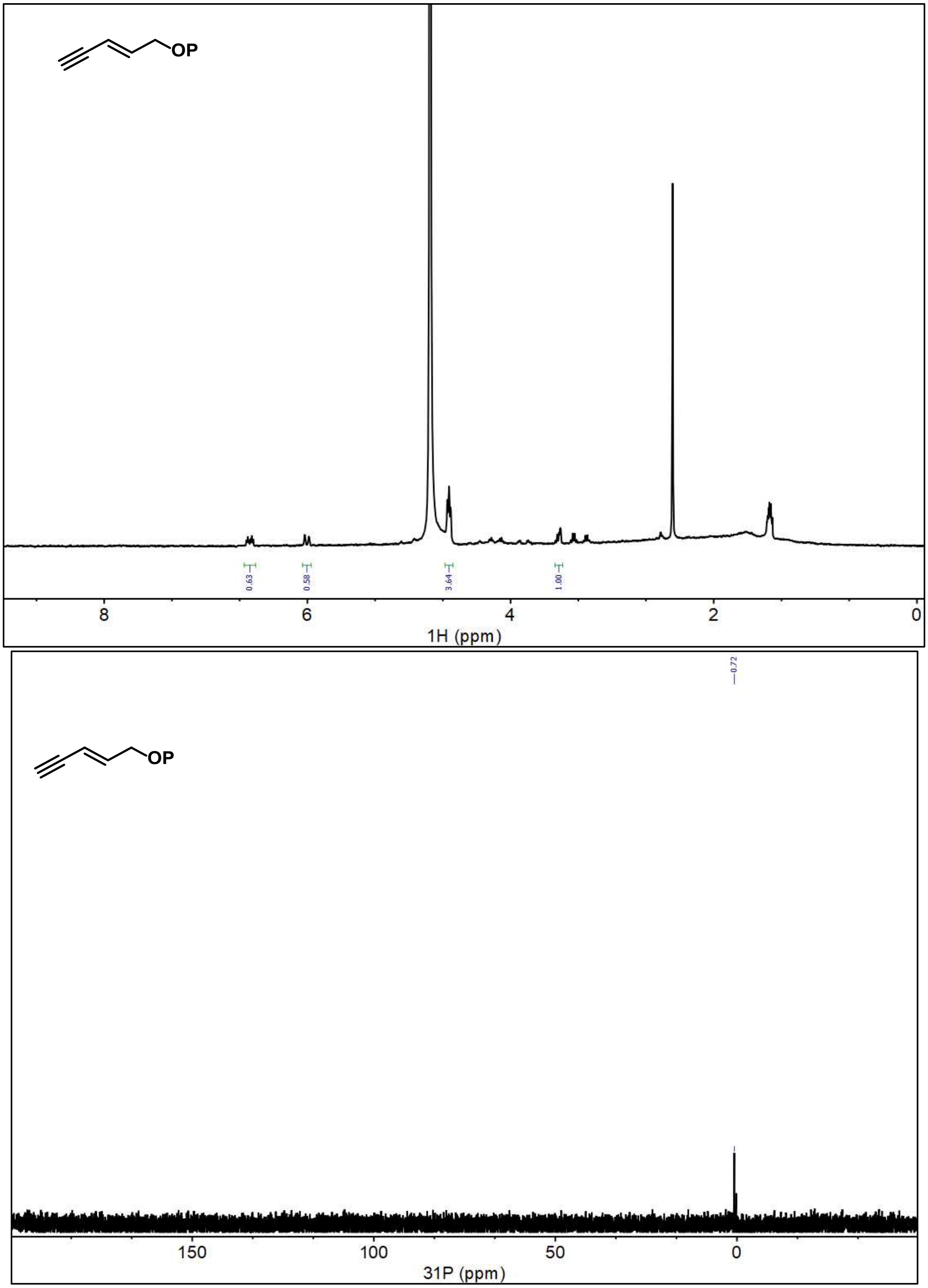

Figure S30. ^{1}H NMR (400 MHz, D_2O) and ^{31}P NMR of **15** (162 MHz, D_2O).

3-Methylpent-2-en-1-yl Phosphate (16):

The title product was obtained as an ivory solid from (*E*)-3-methylpent-2-en-1-ol following the procedure described in *Method 2.7b*.

TLC (^{i}PrOH: NH_4OH: H_2O 7:2:1 v/v): R_f = 0.61.

^{1}H NMR (400 MHz, D_2O): δ 5.42 (t, *J* = 7.1 Hz, 1H), 4.35 (t, *J* = 6.8 Hz, 2H), 2.16 – 1.99 (m, 2H), 1.71 (s, 3H), 1.01 (t, *J* = 7.6 Hz, 3H).

^{31}P NMR (162 MHz, D_2O): δ 2.41.

MS-ESI: Calculated for $C_6H_{12}O_4P$ $[M-H]^-$: 179.04731; Found: 179.0463.

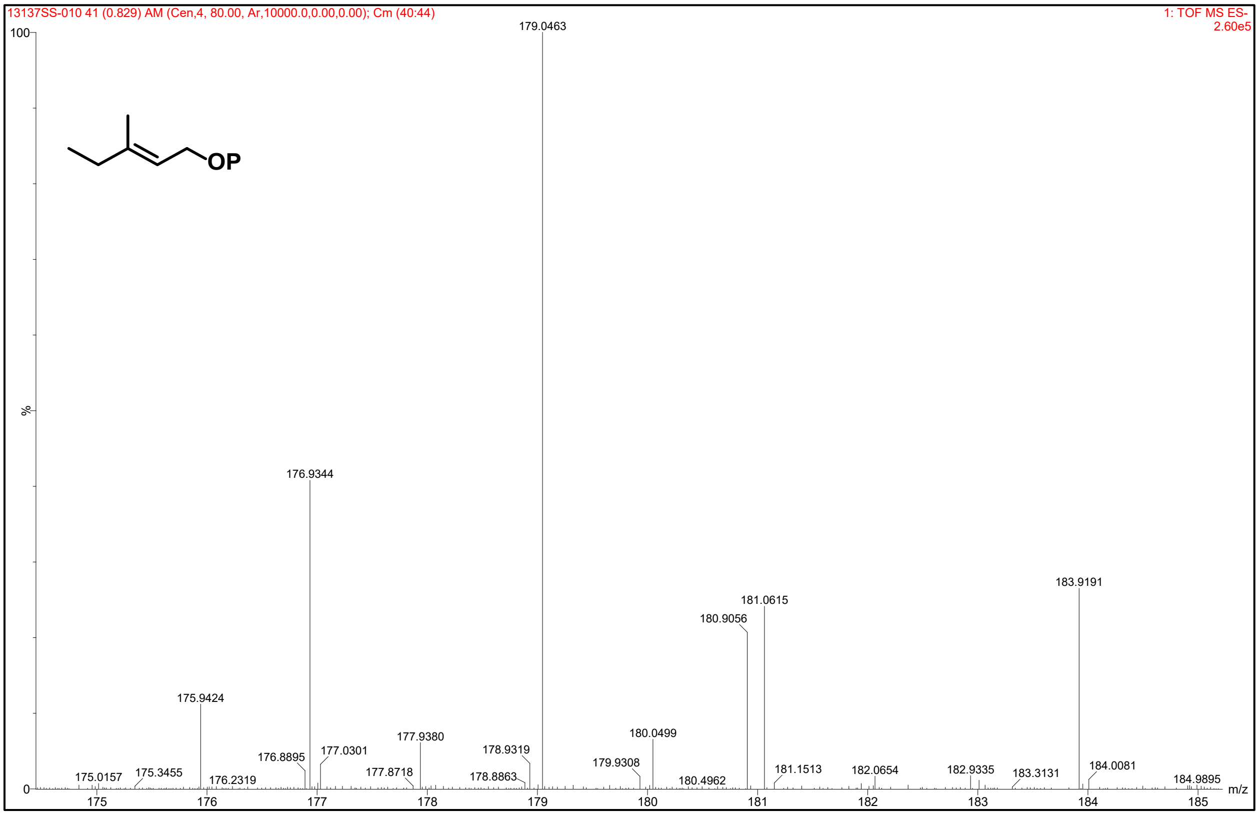

Figure S31. MS-ESI$^-$ of **16**.

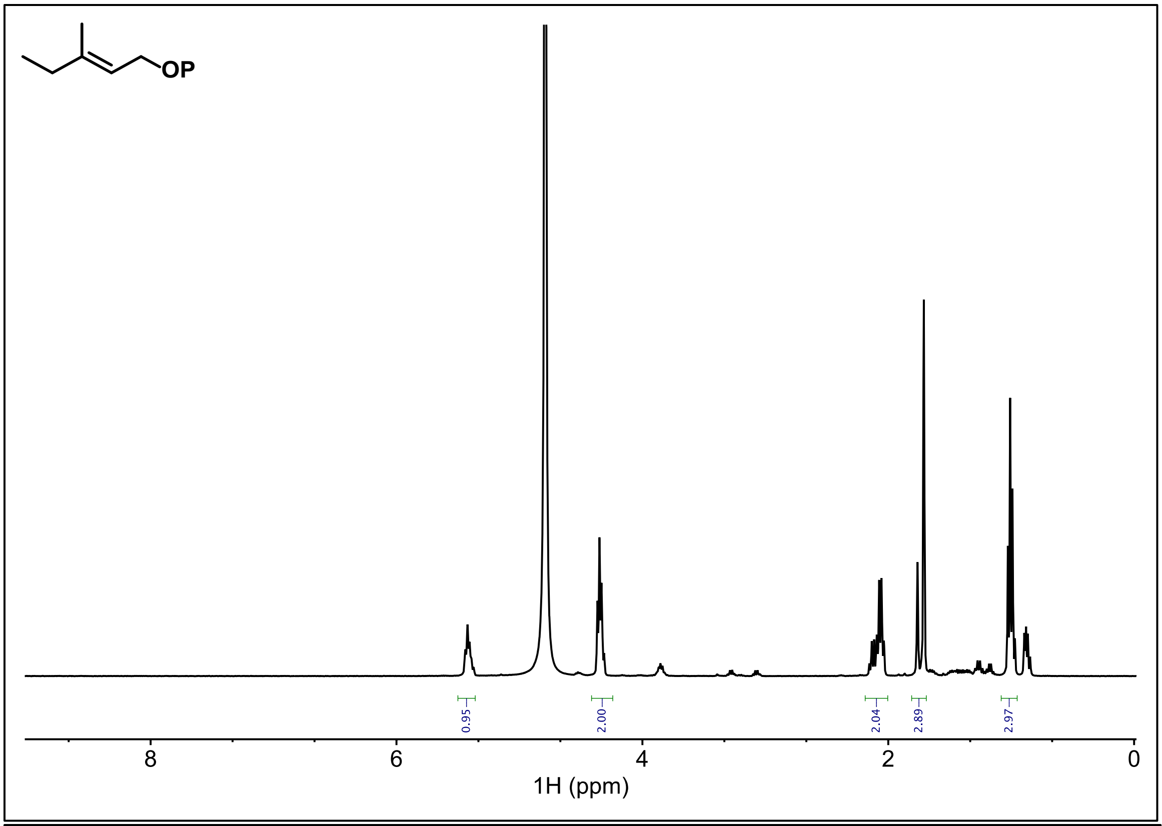

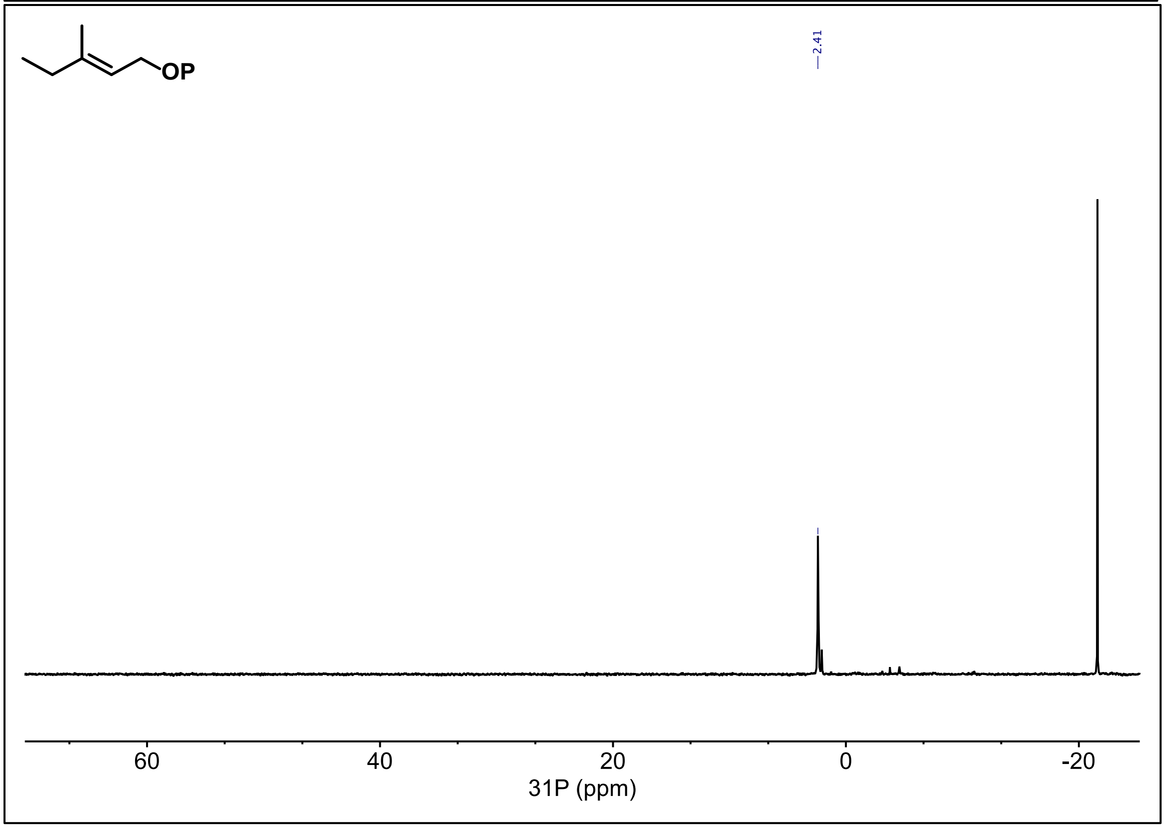

Figure S32. ^{1}H NMR (400 MHz, D_2O) and ^{31}P NMR of **16** (162 MHz, D_2O).

3-Ethylpent-2-en-1-yl Phosphate (17):

The title product was obtained as a light-yellow solid from 3-ethylpent-2-en-1-ol following the procedure described in *Method 2.7b.*

TLC (^{i}PrOH: NH_4OH: H_2O 7:2:1 v/v): $R_f = 0.64$.

^{1}H NMR (300 MHz, D_2O): δ 5.34 (t, $J = 7.2$ Hz, 1H), 4.33 (t, $J = 6.5$ Hz, 2H), 2.19 – 1.98 (m, 4H), 0.95 (q, $J = 7.8$ Hz, 6H).

^{31}P NMR (122 MHz, D_2O): δ 2.02.

HRMS-ESI: Calculated for $C_7H_{14}O_4P$ $[M-H]^-$: 193.06296; Found: 193.0627.

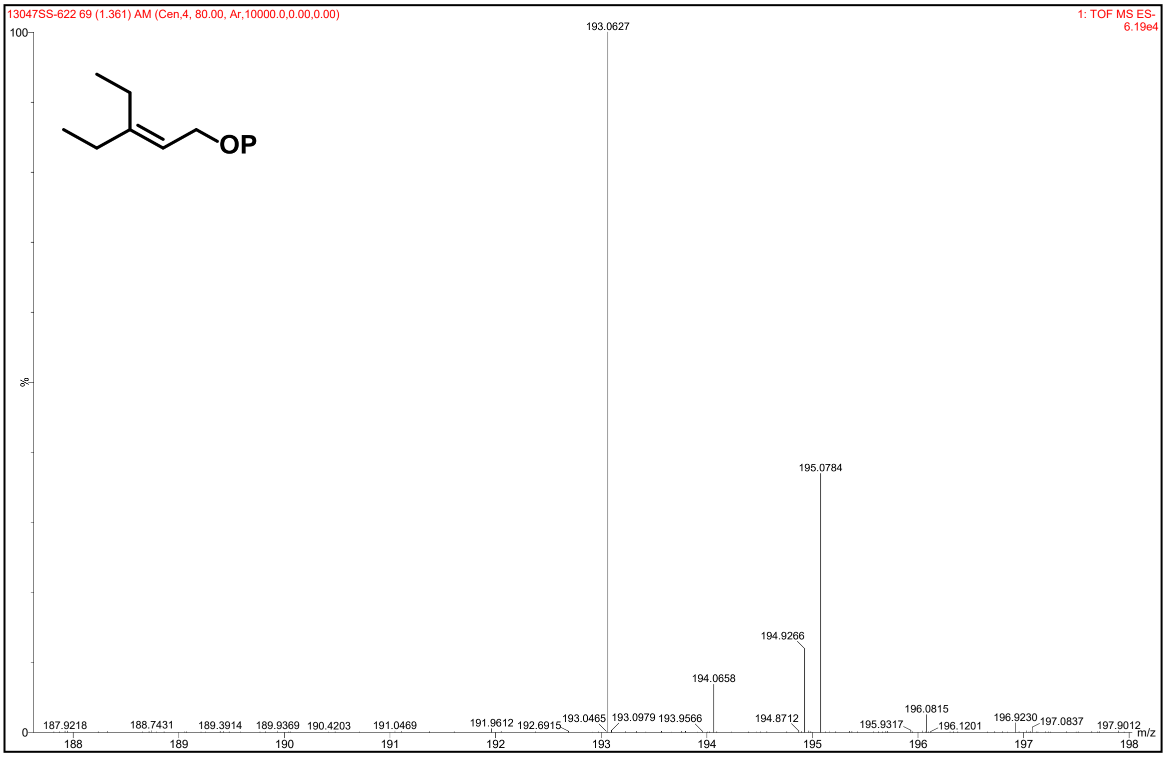

Figure S33. HRMS-ESI$^-$ of **17.**

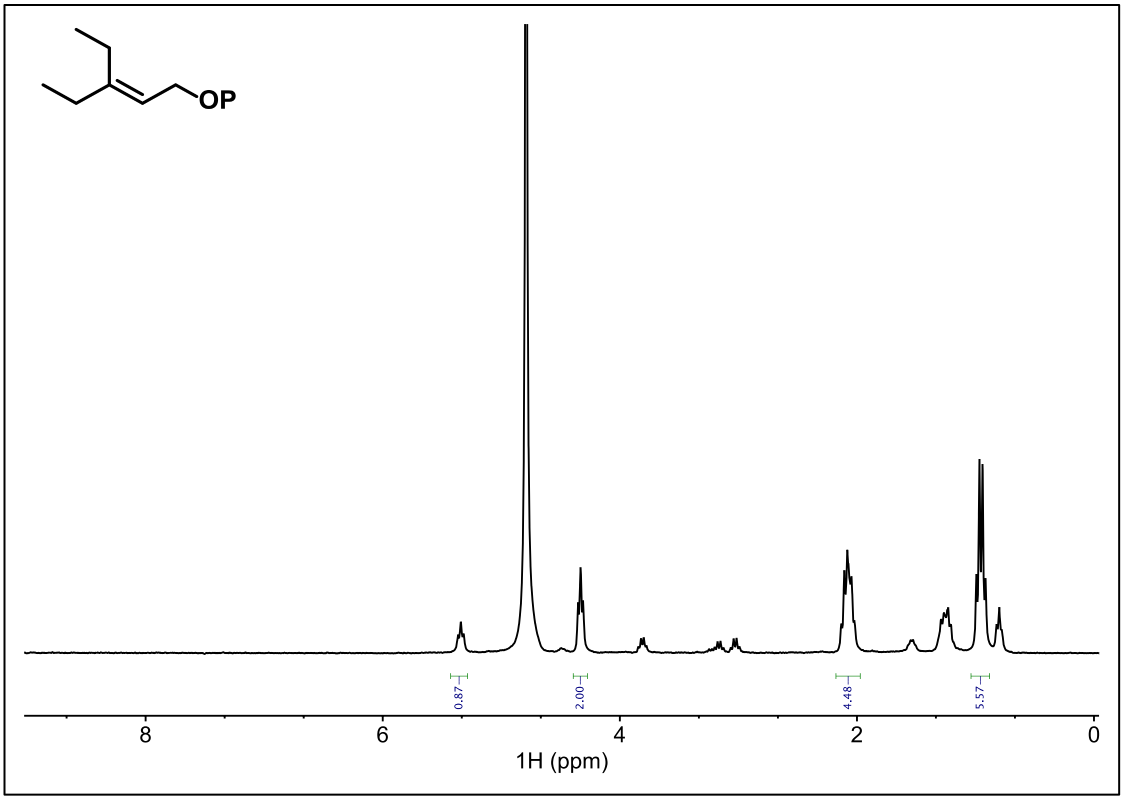

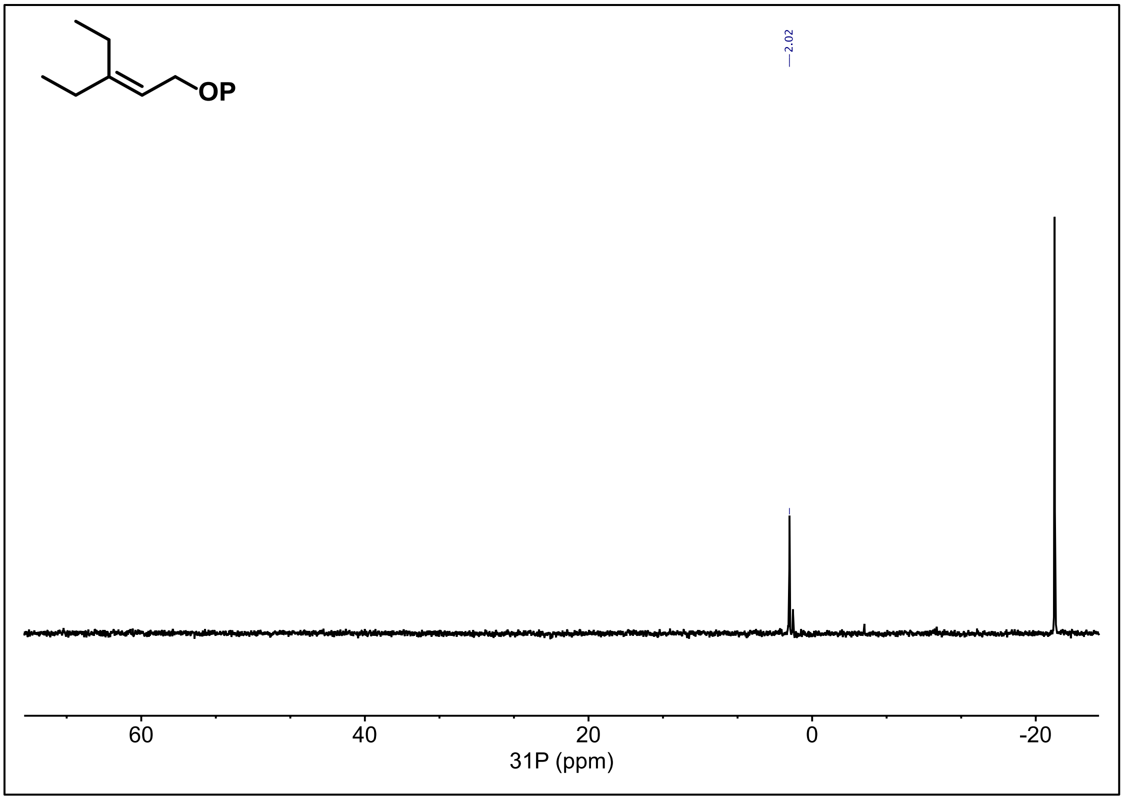

Figure S34. ^{1}H NMR (300 MHz, D_2O) and ^{31}P NMR of **17** (122 MHz, D_2O).

2-Cyclopentylideneethyl Phosphate (18):

The title product was obtained as an ivory solid from 2-cyclopentylideneethan-1-ol following the procedure described in *Method 2.7b.*

TLC (^{i}PrOH: NH_4OH: H_2O 7:2:1 v/v): R_f = 0.62.

^{1}H NMR (300 MHz, D_2O): δ 5.49 (ddt, *J* = 7.2, 4.9, 2.3 Hz, 1H), 4.43 – 4.21 (m, 2H), 2.39 – 2.15 (m, 4H), 1.77 – 1.50 (m, 4H).

^{31}P NMR (122 MHz, D_2O): δ 1.65.

HRMS-ESI: Calculated for $C_7H_{12}O_4P$ $[M-H]^-$: 191.04731; Found: 191.0476.

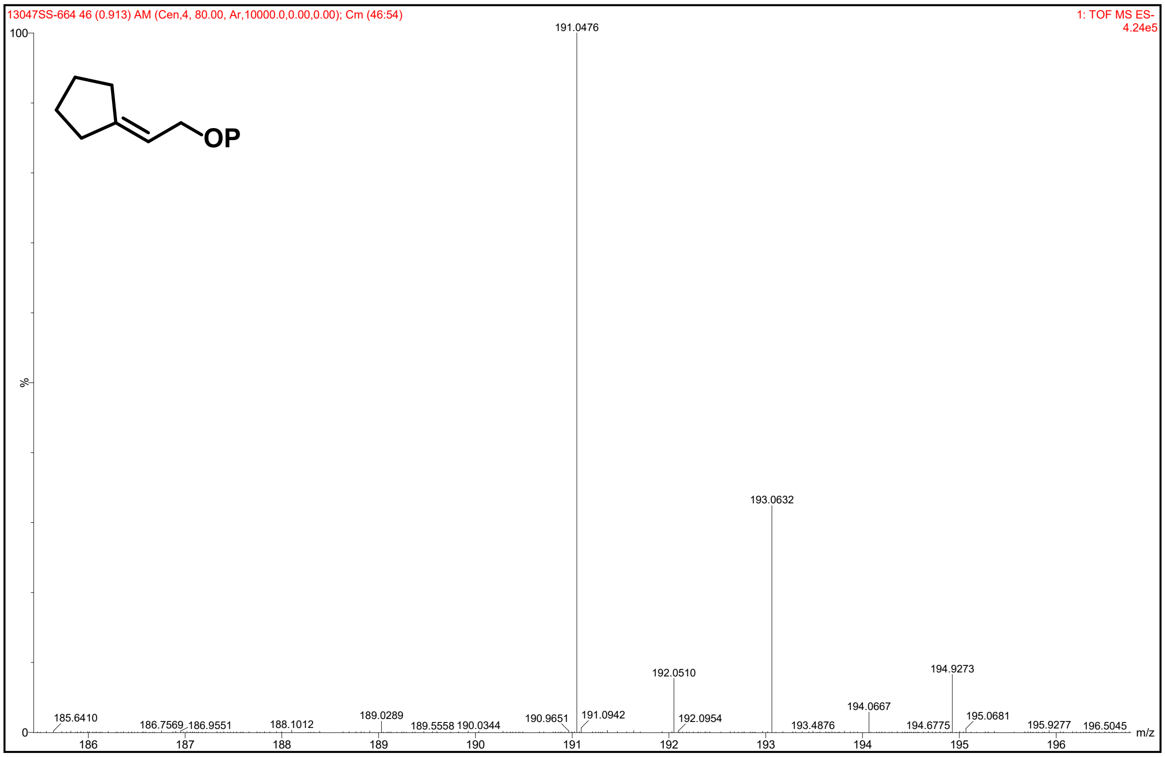

Figure S35. HRMS-ESI$^-$ of **18.**

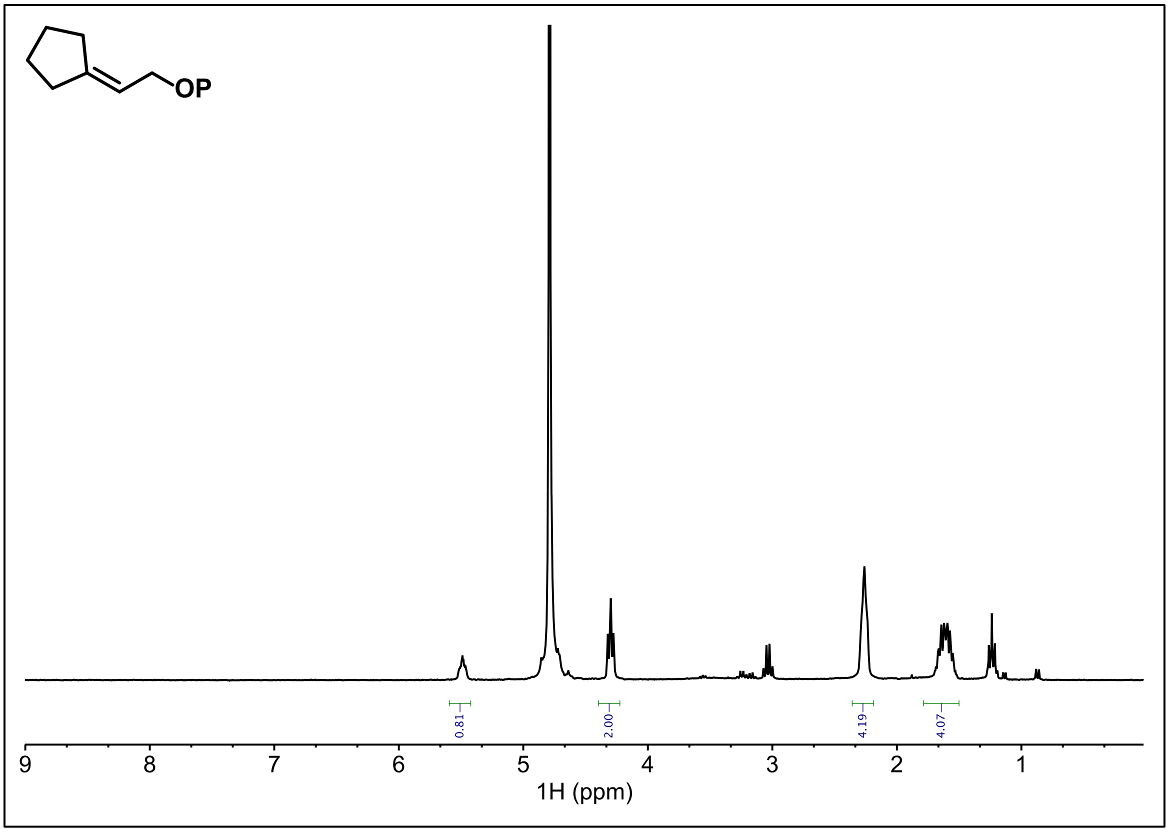

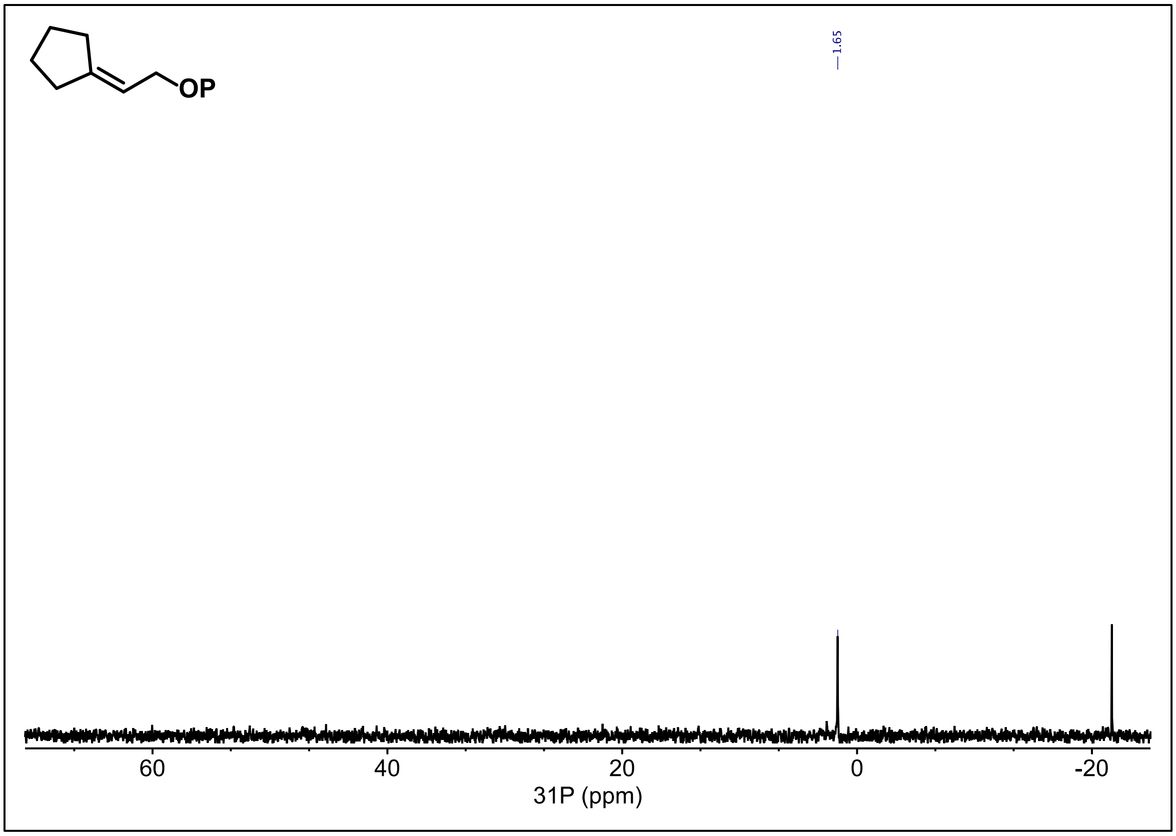

Figure S36. ^{1}H NMR (300 MHz, D_2O) and ^{31}P NMR of **18** (122 MHz, D_2O)**.**

2-Cyclohexylideneethyl Phosphate (19):

The title product was obtained as a white solid from 2-cyclohexylideneethan-1-ol following the procedure described in *Method 2.7b.*

TLC (^{i}PrOH: NH_4OH: H_2O 7:2:1 v/v): R_f = 0.68.

^{1}H NMR (300 MHz, D_2O): δ 5.18 (t, *J* = 7.4 Hz, 1H), 4.13 (t, *J* = 6.5 Hz, 2H), 2.15 – 1.80 (m, 4H), 1.35 (s, 6H).

^{31}P NMR (122 MHz, D_2O): δ 2.58.

HRMS-ESI: Calculated for $C_8H_{14}O_4P$ $[M-H]^-$: 205.06296; Found: 205.0628.

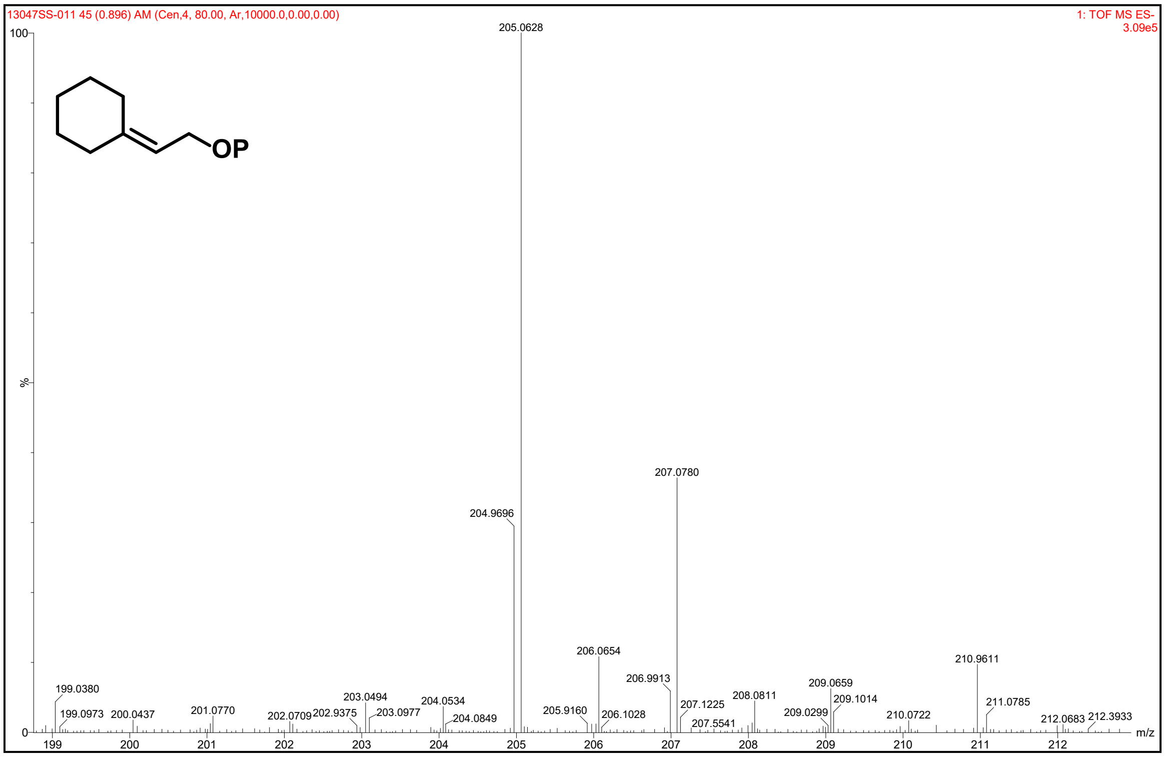

Figure S37. HRMS-ESI$^-$ of **19.**

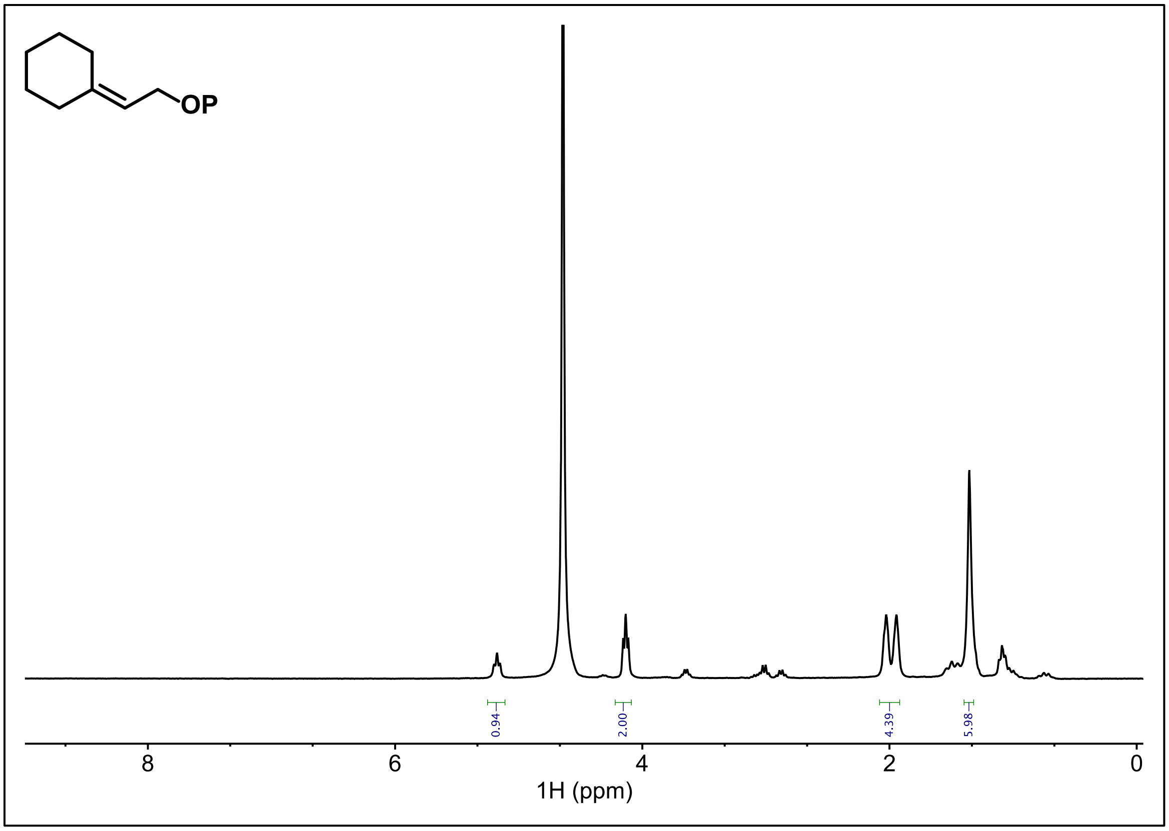

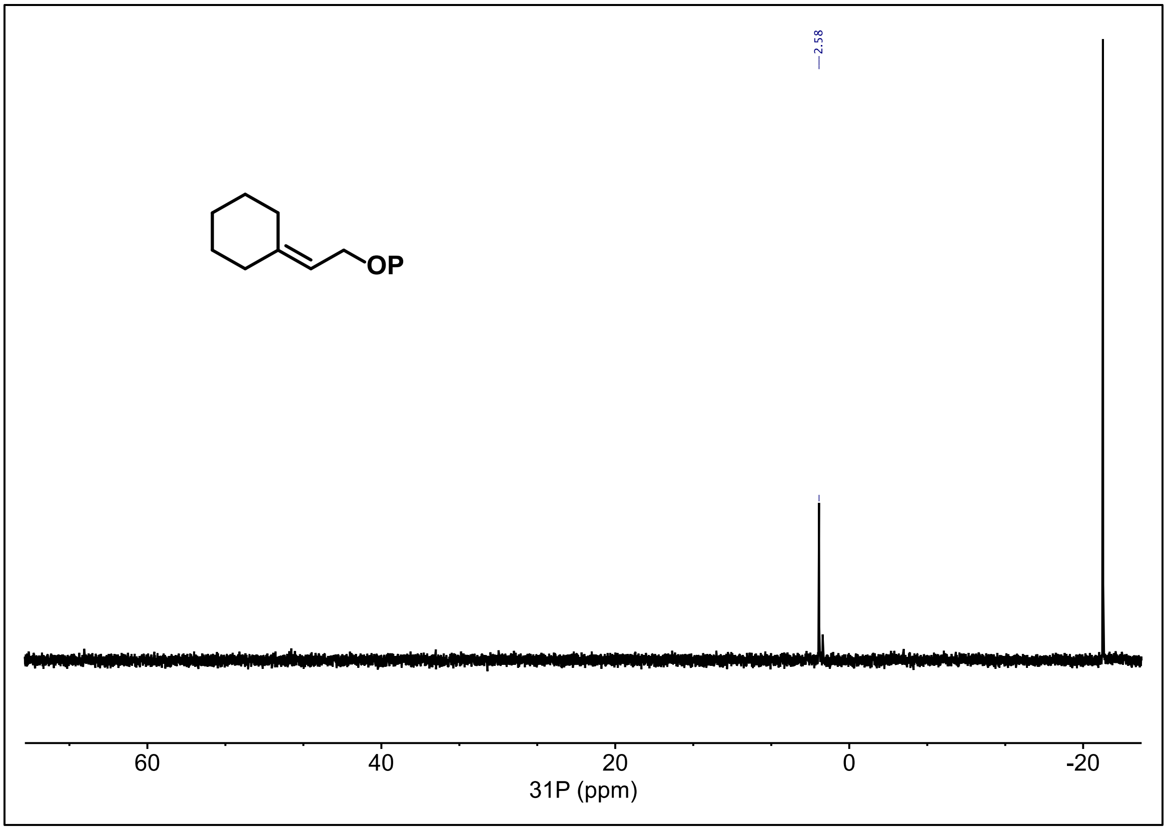

Figure S38. ^{1}H NMR (300 MHz, D_2O) and ^{31}P NMR of **19** (122 MHz, D_2O)**.**

(2*E*,4*E*)-Hexa-2,4-dien-1-yl) Phosphate (20):

The title product was obtained as a brown solid from (2*E*,4*E*)-hexa-2,4-dien-1-ol following the procedure described in *Method 2.7b.*

TLC (^{i}PrOH: NH_4OH: H_2O 7:2:1 v/v): R_f = 0.63.

^{1}H NMR (400 MHz, D_2O): δ 6.33 (dd, *J* = 15.2, 10.4 Hz, 1H), 6.16 (ddd, *J* = 15.1, 10.5, 1.9 Hz, 1H), 5.84 (dd, *J* = 14.9, 7.0 Hz, 1H), 5.73 (d, *J* = 15.2 Hz, 1H), 4.34 (s, 2H), 1.74 (dd, *J* = 6.8, 1.6 Hz, 3H).

^{31}P NMR (162 MHz, D_2O): δ 1.70.

HRMS-ESI: Calculated for $C_6H_{10}O_4P$ $[M-H]^-$: 177.03166; Found: 177.0316.

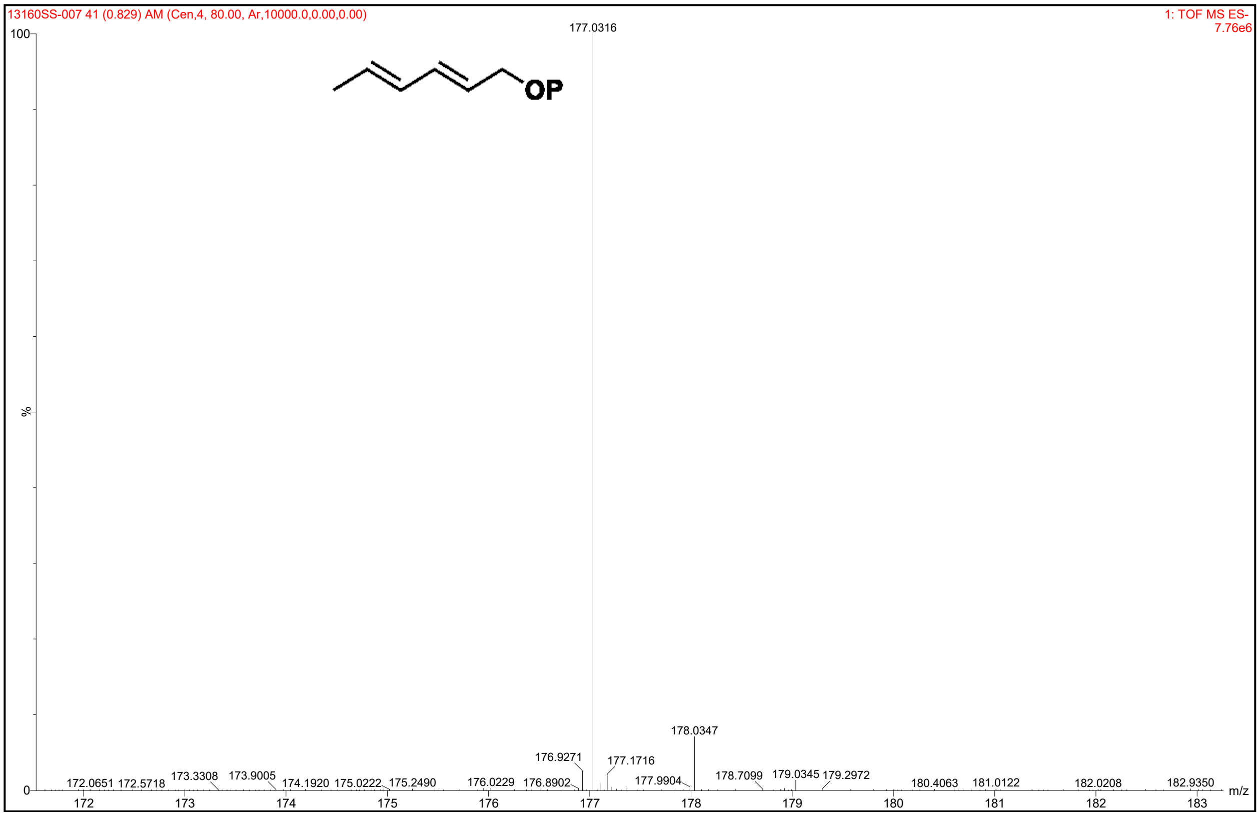

Figure S39. HRMS-ESI$^-$ of **20.**

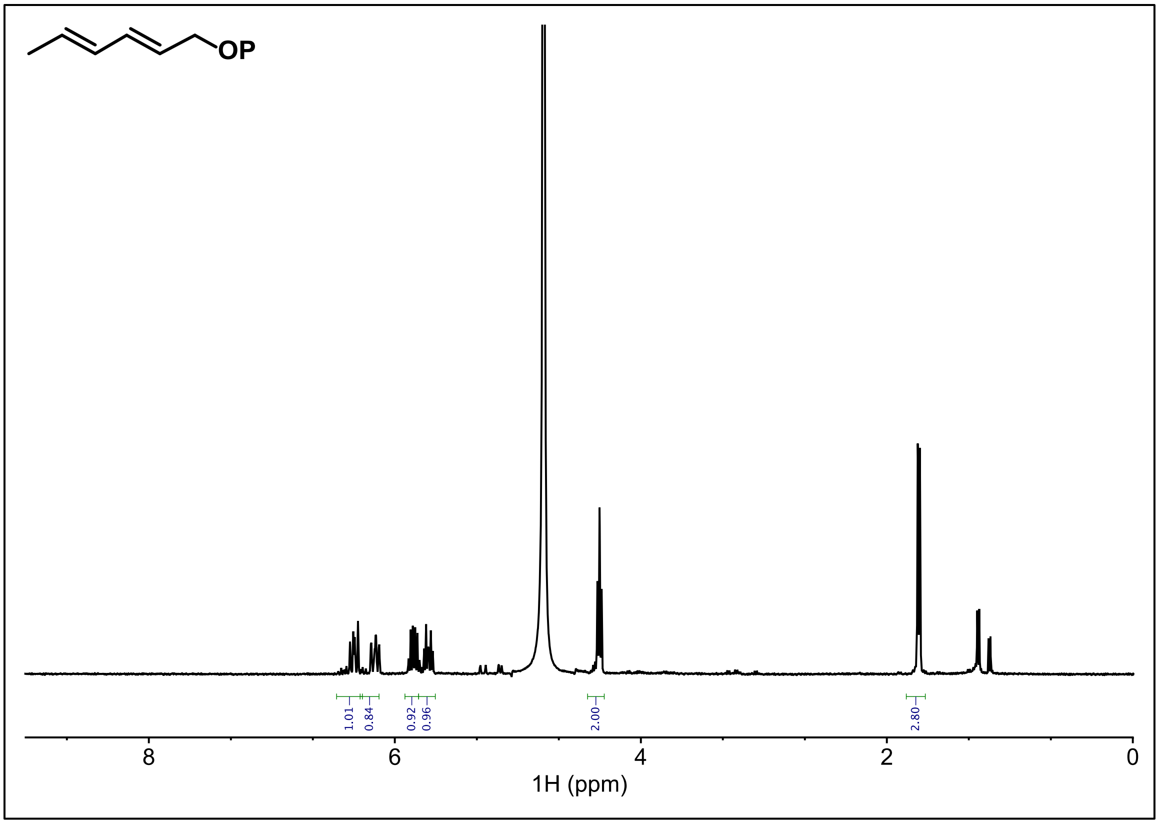

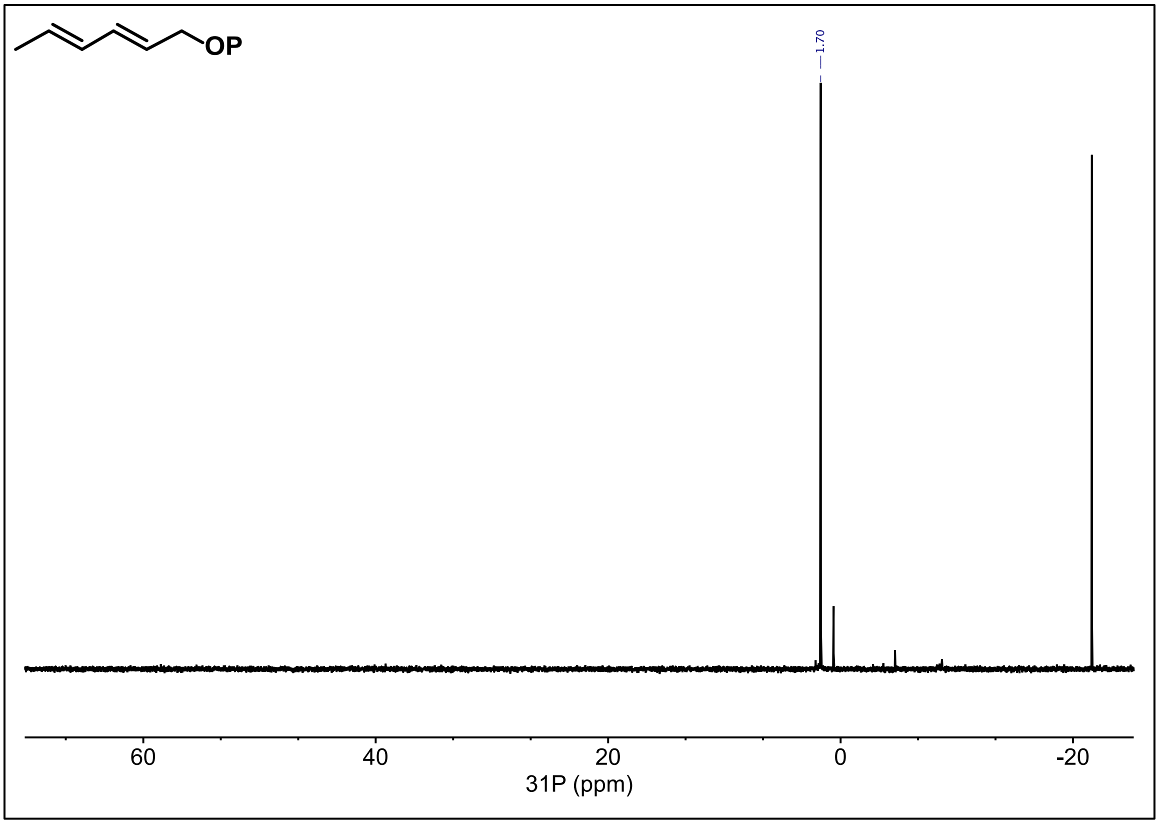

Figure S40. ^{1}H NMR (400 MHz, D_2O) and ^{31}P NMR of **20** (162 MHz, D_2O).

(2*E*,4*E*)-2-Methylhexa-2,4-dien-1-yl Phosphate (21):
The title product was obtained as a white solid from (2*E*,4*E*)-2-methylhexa-2,4-dien-1-ol following the procedure described in *Method 2.7b.*
TLC (^{i}PrOH: NH_4OH: H_2O 7:2:1 v/v): R_f = 0.63.
^{1}H NMR (300 MHz, D_2O): δ 6.45 – 6.27 (m, 1H), 6.08 (d, *J* = 11.8 Hz, 1H), 5.91 – 5.70 (m, 1H), 4.21 (d, *J* = 6.1 Hz, 2H), 1.73 (s, 6H).
^{31}P NMR (122 MHz, D_2O): δ 1.53.
HRMS-ESI: Calculated for $C_7H_{12}O_4P$ $[M-H]^-$: 191.04731; Found: 191.0472.

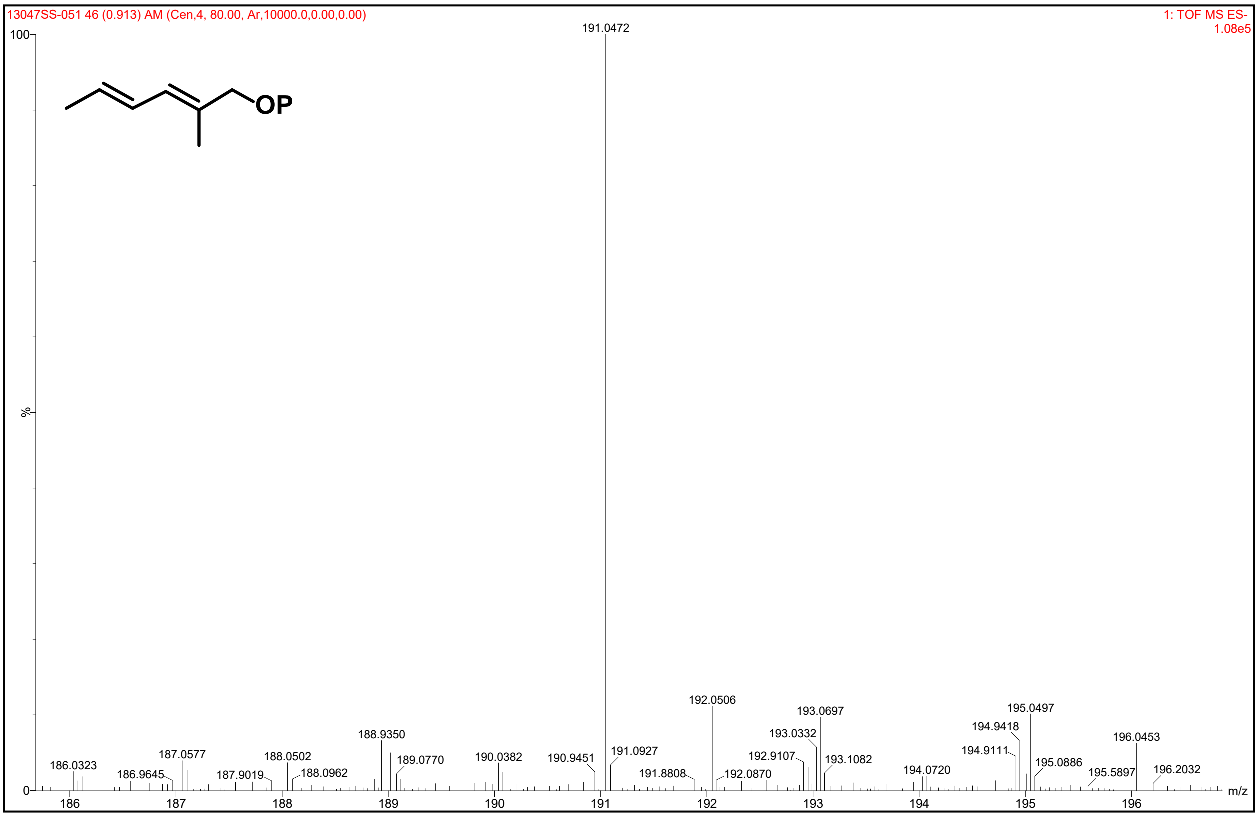

Figure S41. HRMS-ESI$^-$ of **21.**

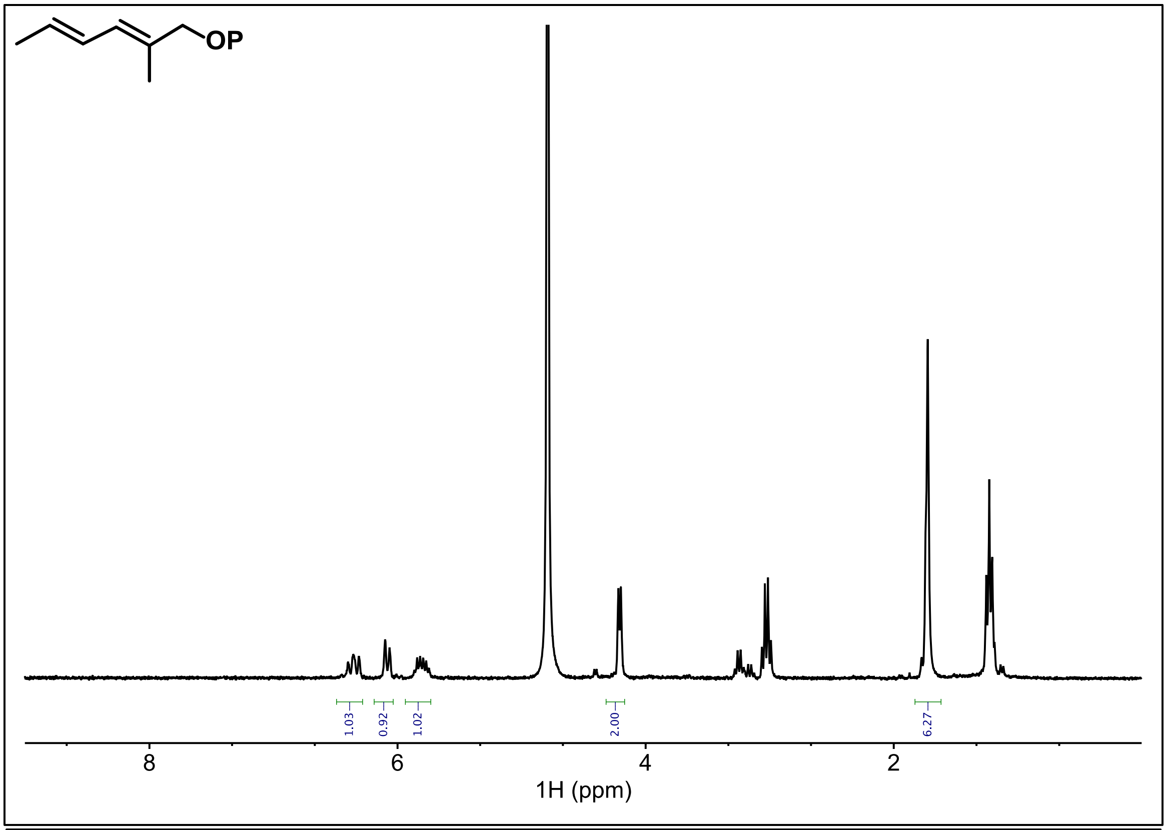

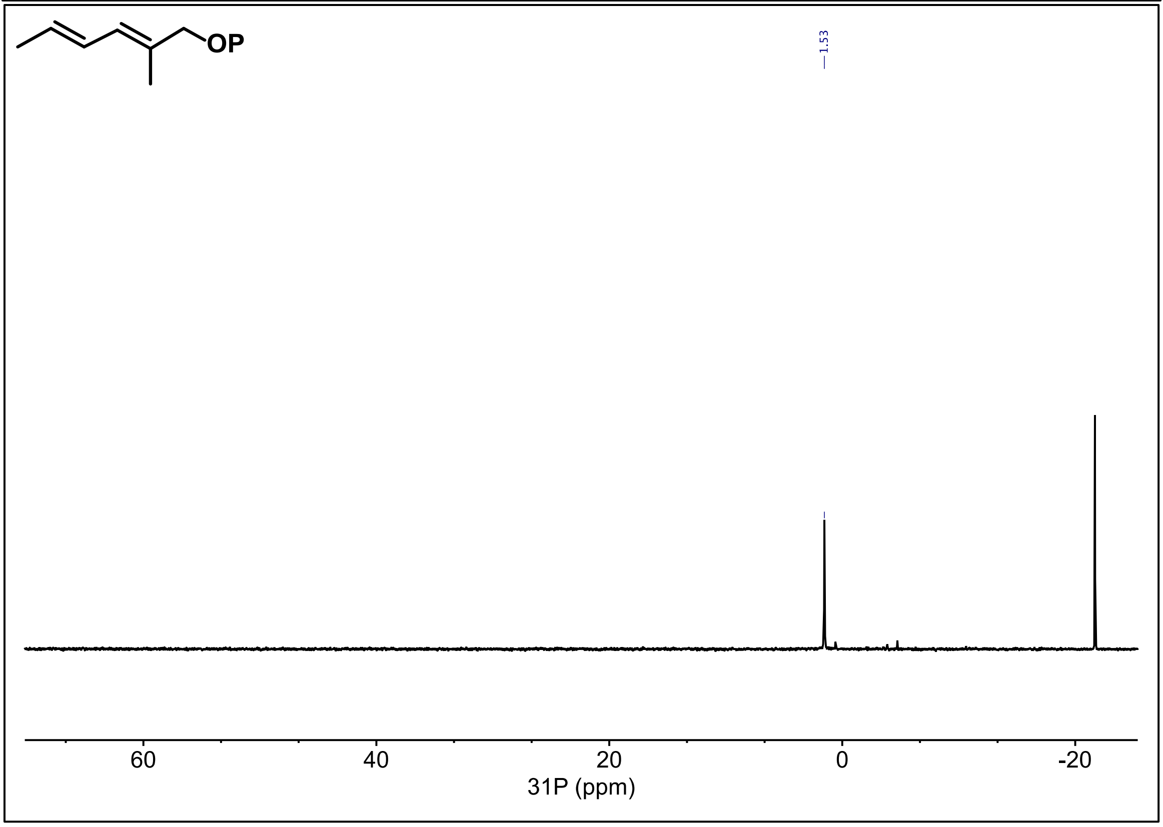

Figure S42. ^{1}H NMR (300 MHz, D_2O) and ^{31}P NMR of **21** (122 MHz, D_2O).

5-Methylhexa-2,4-dien-1-yl Phosphate (22):

The title product was obtained as a yellow-white solid from (*E*)-5-methylhexa-2,4-dien-1-ol following the procedure described in *Method 2.7b.*

TLC (^{i}PrOH: NH_4OH: H_2O 7:2:1 v/v): R_f = 0.63.

^{1}H NMR (400 MHz, D_2O): δ 5.50 (d, J = 6.8 Hz, 1H), 4.44 (t, J = 6.9 Hz, 2H), 3.51 – 3.36 (m, 2H), 2.50 – 2.33 (m, 2H), 2.29 (t, J = 7.3 Hz, 2H), 1.73 (s, 3H).

^{31}P NMR (162 MHz, D_2O): δ 0.54.

HRMS-ESI: Calculated for $C_7H_{12}O_4P$ $[M-H]^-$: 191.04731; Found: 191.0473.

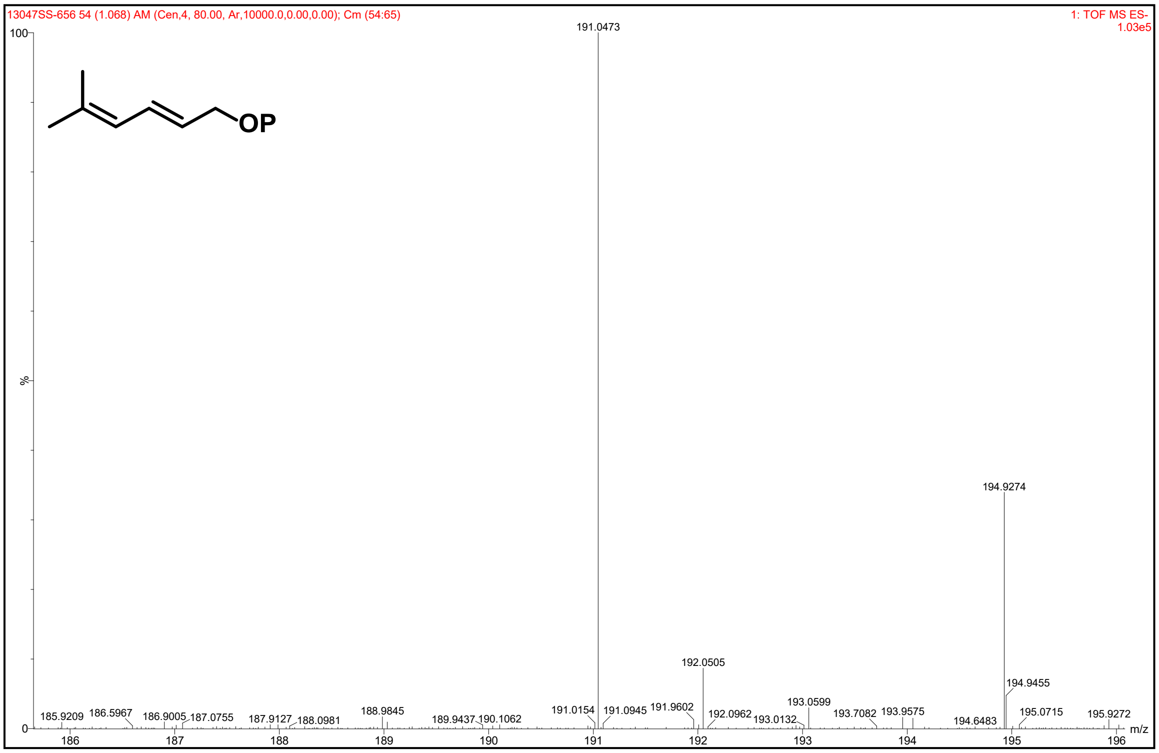

Figure S43. HRMS-ESI$^-$ of **22.**

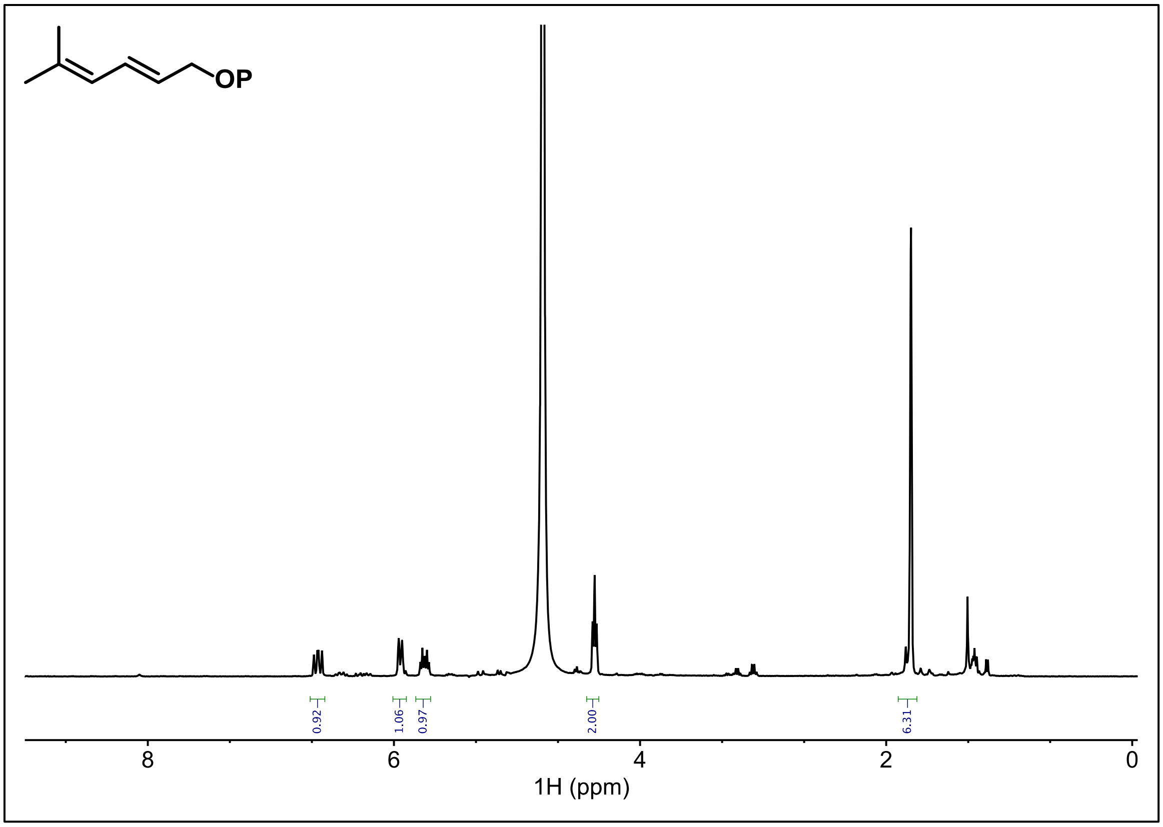

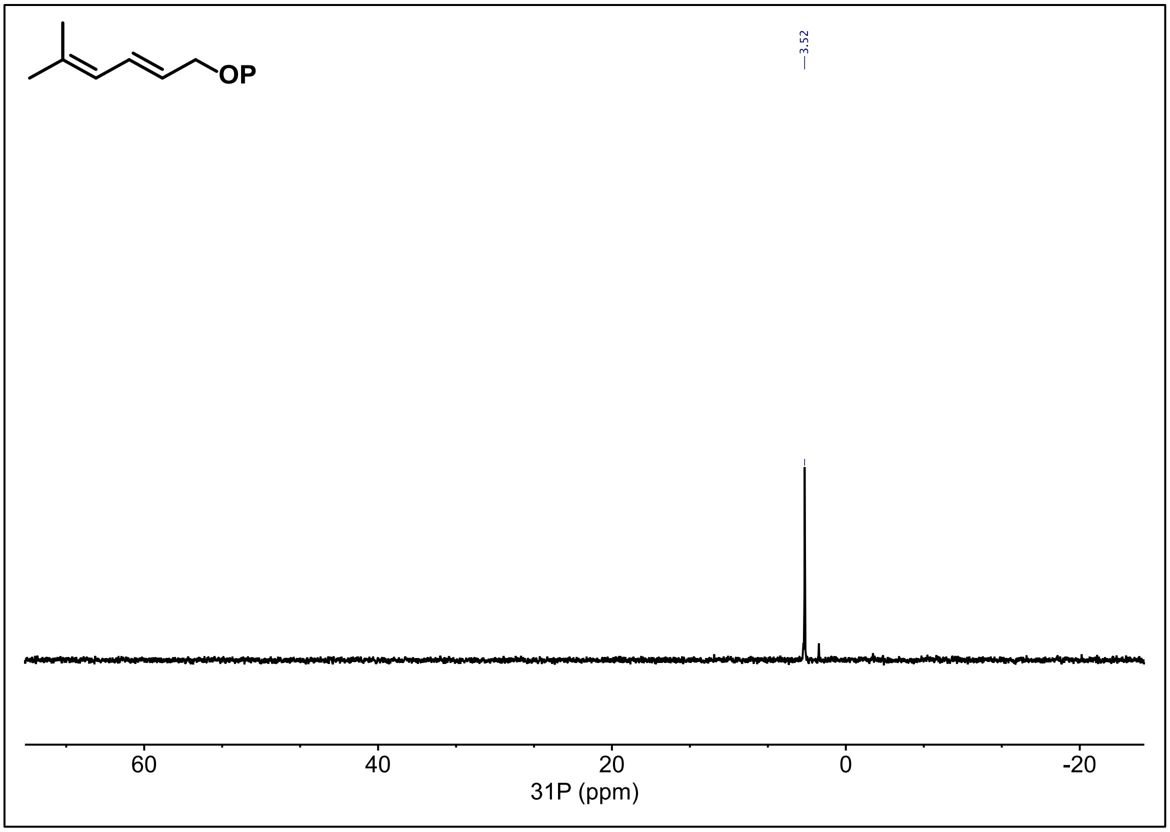

Figure S44. ^{1}H NMR (400 MHz, D_2O) and ^{31}P NMR of **22** (162 MHz, D_2O).

3-Methylhex-2-en-1-yl Phosphate (23):
The title product was obtained as an ivory solid from (*E*)-3-methylhex-2-en-1-ol following the procedure described in *Method 2.7b*.
TLC (^{i}PrOH:NH_4OH:H_2O 7:2:1 v/v): R_f = 0.65.
^{1}H NMR (300 MHz, D_2O): δ 5.48 – 5.29 (m, 1H), 4.34 (t, *J* = 6.6 Hz, 2H), 2.01 (t, *J* = 7.5 Hz, 2H), 1.66 (s, 3H), 1.41 (dd, *J* = 8.5, 6.5 Hz, 2H), 0.84 (t, *J* = 7.4 Hz, 3H).
^{31}P NMR (122 MHz, D_2O): δ 4.13.
HRMS-ESI: Calculated for $C_7H_{14}O_4P$ [M-H]$^-$: 193.06296; Found: 193.0629.

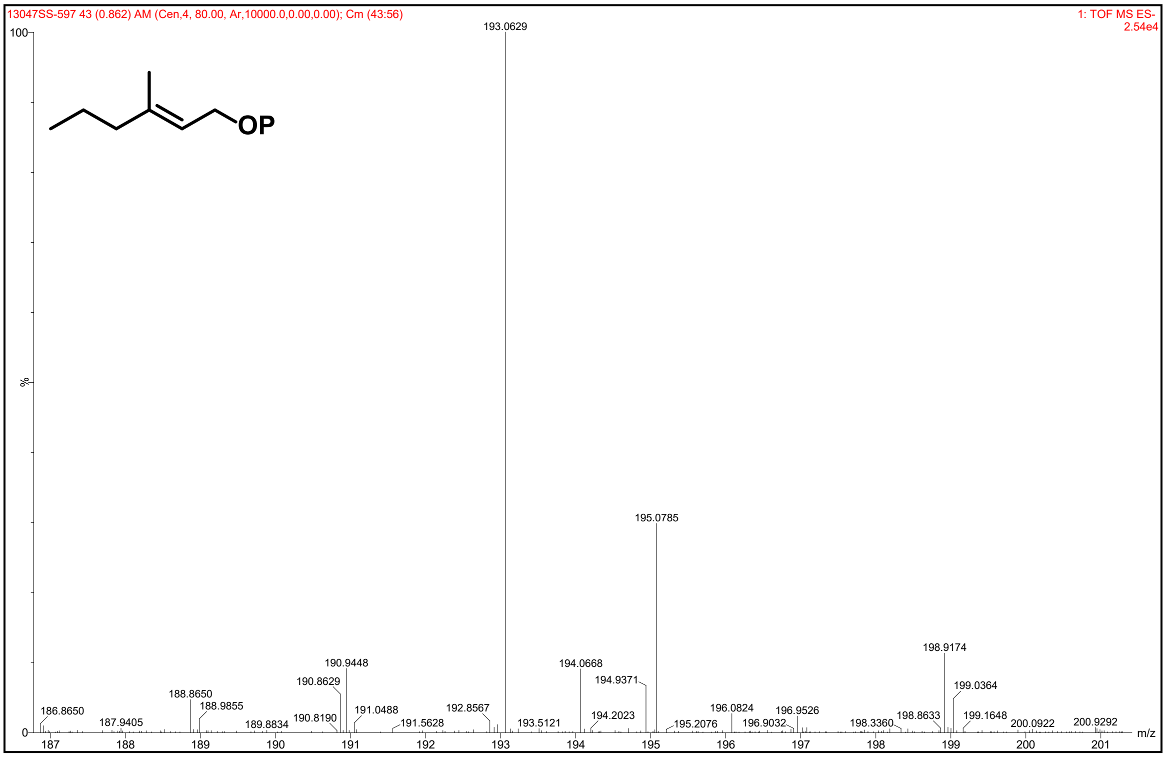

Figure S45. HRMS-ESI$^-$ of **23.**

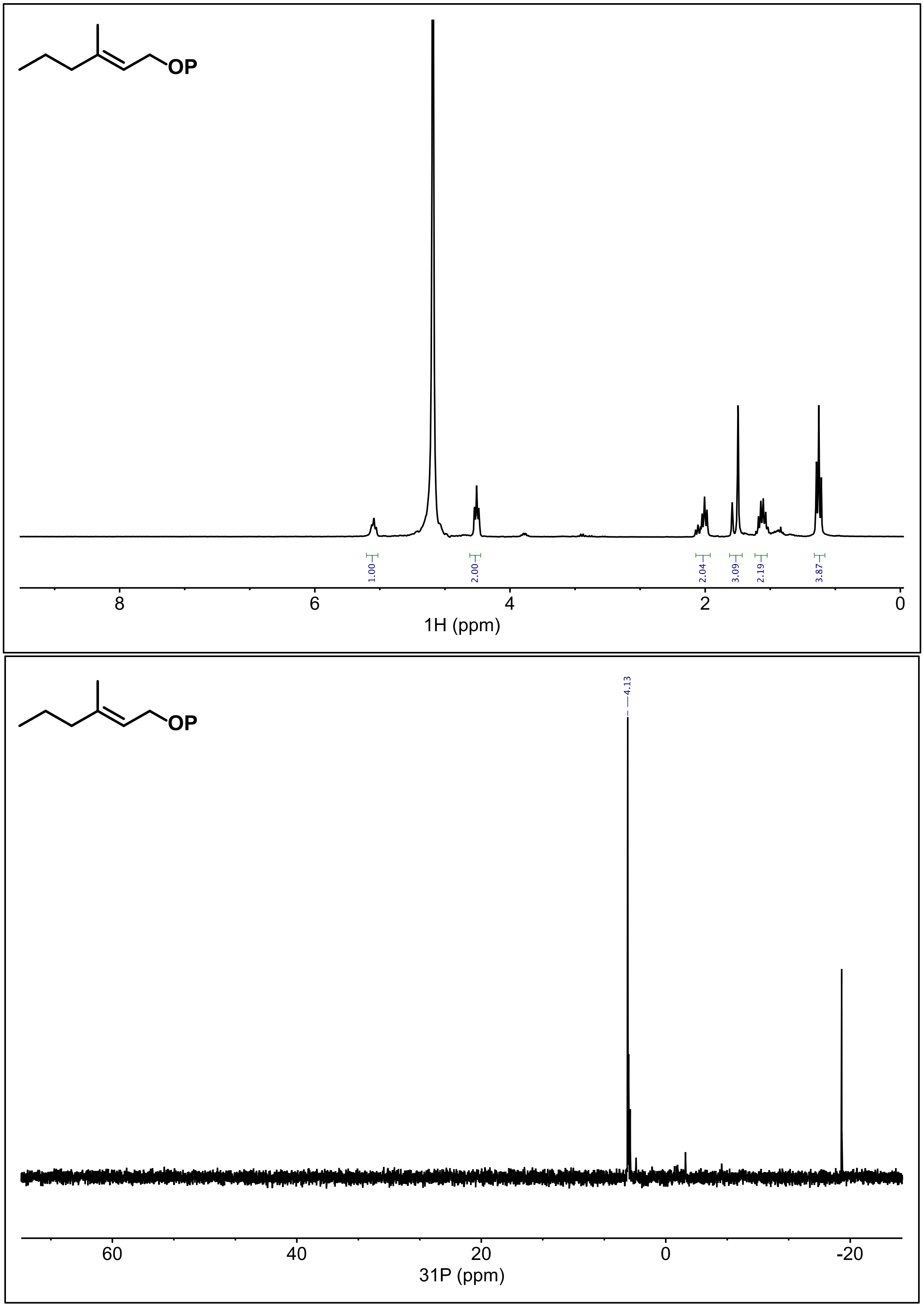

Figure S46. ^{1}H NMR (300 MHz, D_2O) and ^{31}P NMR of **23** (122 MHz, D_2O).

3-Ethylhex-2-en-1-yl Phosphate (24):
The title product was obtained as a white solid from (*E*)-3-ethylhex-2-en-1-ol following the procedure described in *Method 2.7b.*
TLC (^{i}PrOH: NH_4OH: H_2O 7:2:1 v/v): R_f = 0.61.
^{1}H NMR (400 MHz, D_2O): δ 5.41 (dt, *J* = 15.1, 7.1 Hz, 1H), 4.39 (t, *J* = 6.1 Hz, 2H), 2.09 (dt, *J* = 20.5, 7.5 Hz, 4H), 1.53 – 1.23 (m, 4H), 1.09 – 0.77 (m, 6H).
^{31}P NMR (162 MHz, D_2O): δ 1.74.
MS-ESI: Calculated for $C_8H_{16}O_4P$ $[M-H]^-$: 207.07861; Found: 207.0800.

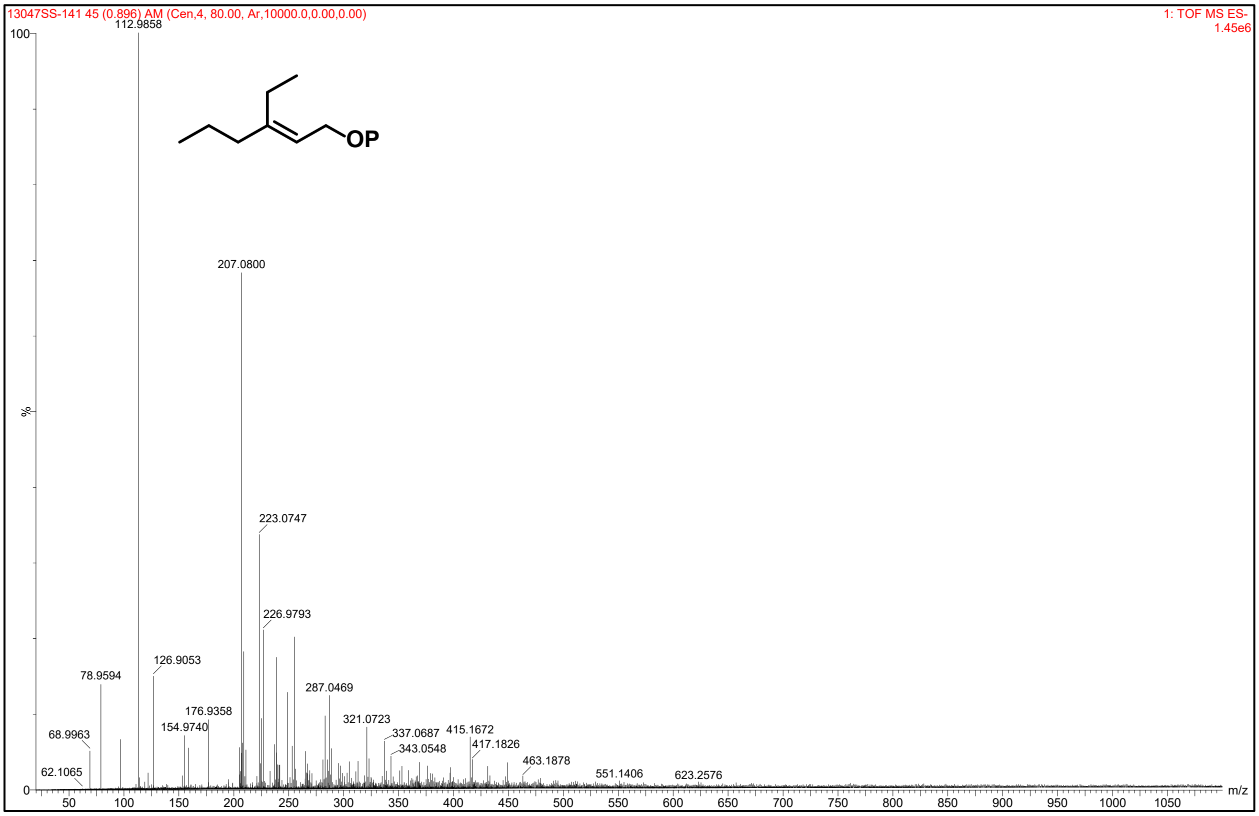

Figure S47. MS-ESI$^-$ of **24.**

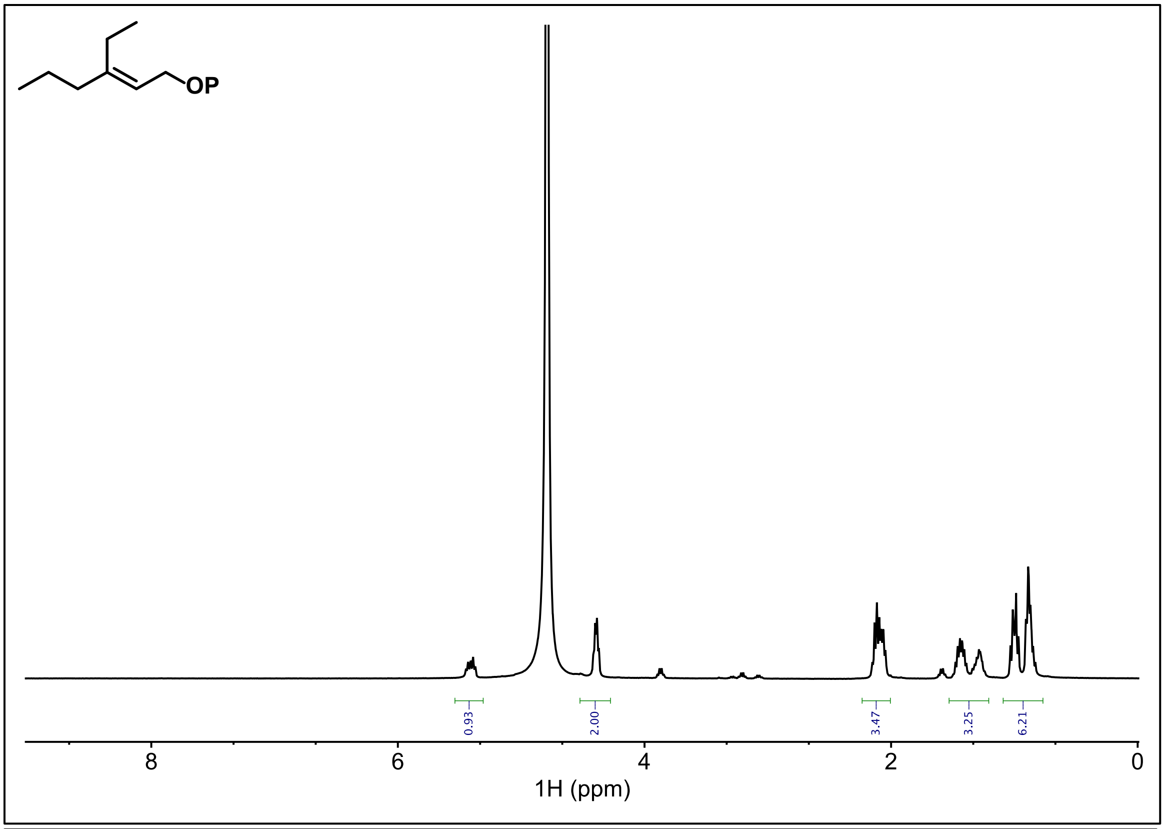

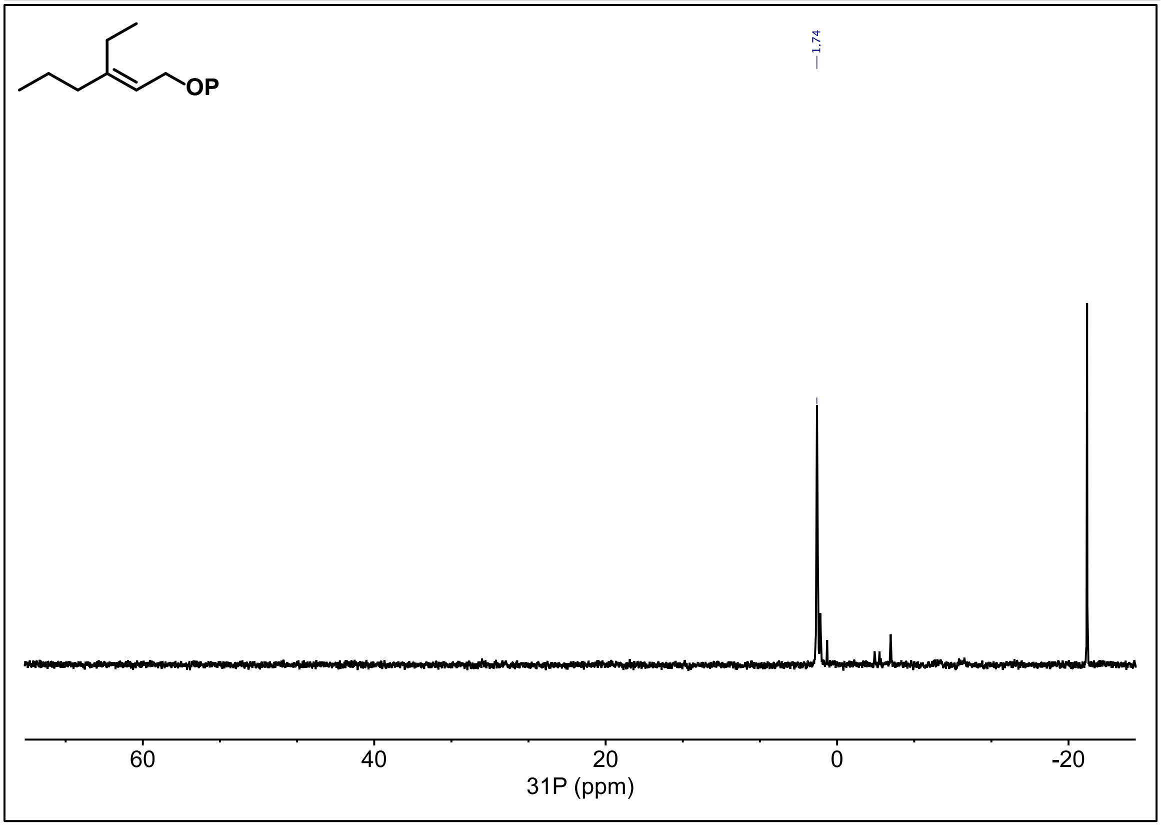

Figure S48. ^{1}H NMR (400 MHz, D_2O) and ^{31}P NMR of **24** (162 MHz, D_2O).

Cinnamyl Phosphate (25):

The title product was obtained as a yellow-white solid from (*E*)-3-phenylprop-2-en-1-ol following the procedure described in *Method 2.7b.*

TLC (^{i}PrOH: NH_4OH: H_2O 8:1:1 v/v): R_f = 0.64.

^{1}H NMR (300 MHz, D_2O): δ 7.49 (d, *J* = 8.1 Hz, 2H), 7.46 – 7.16 (m, 3H), 6.70 (d, *J* = 15.9 Hz, 1H), 6.58 – 6.19 (m, 1H), 4.44 (t, *J* = 6.4 Hz, 2H).

^{31}P NMR (122 MHz, D_2O): δ 2.54.

HRMS-ESI: Calculated for $C_9H_{10}O_4P$ $[M-H]^-$: 213.03166; Found: 213.0307.

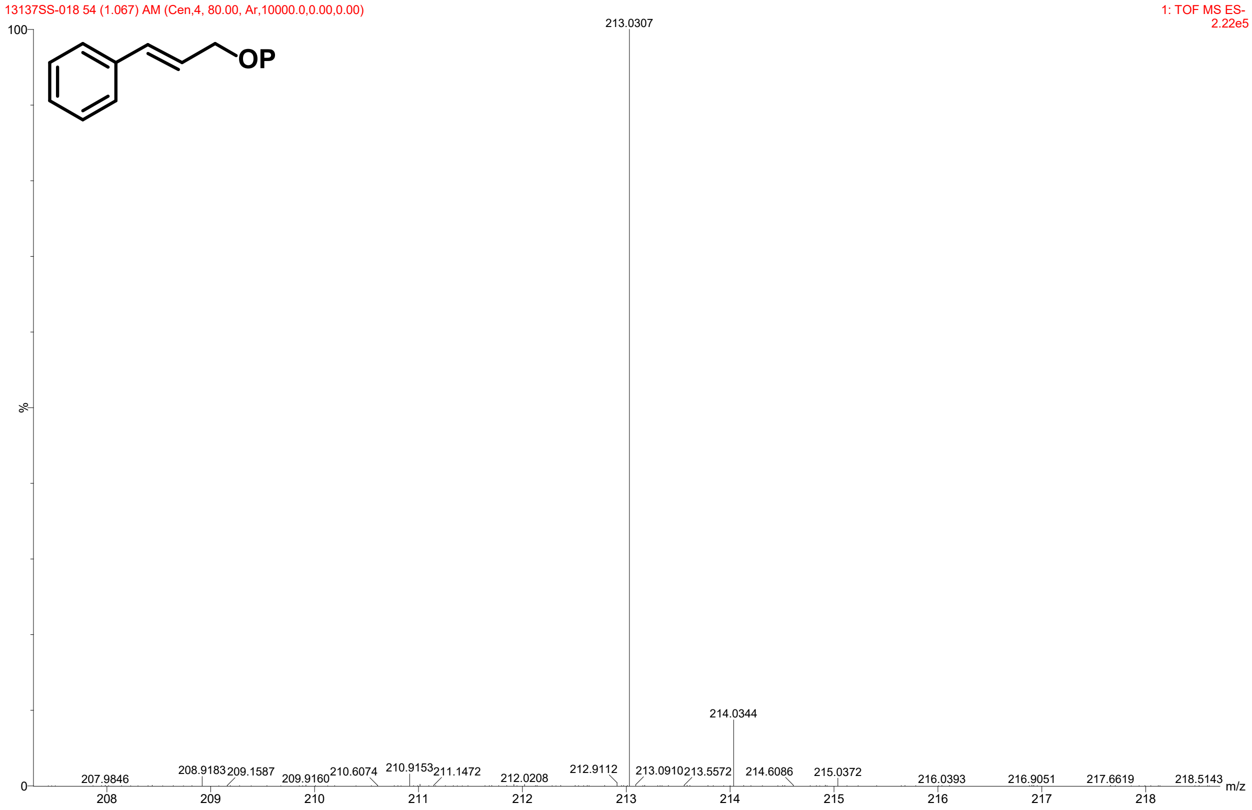

Figure S49. HRMS-ESI$^-$ of **25.**

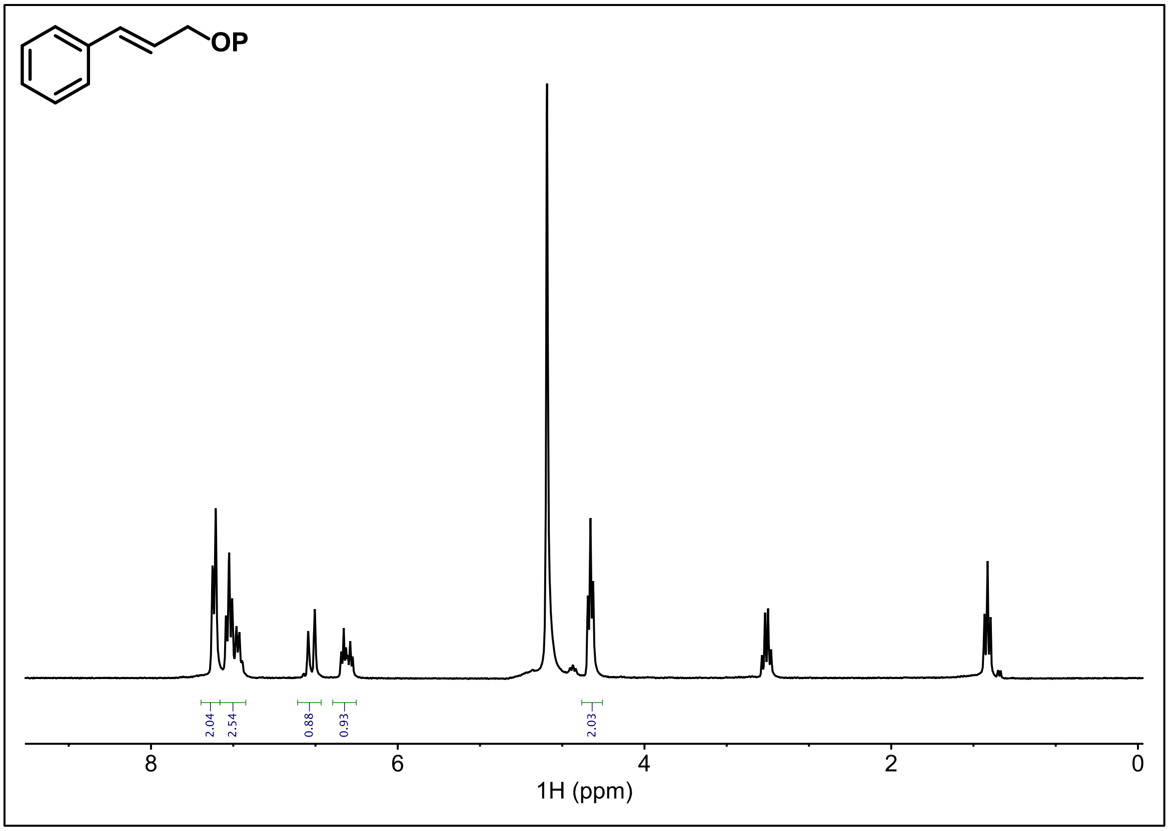

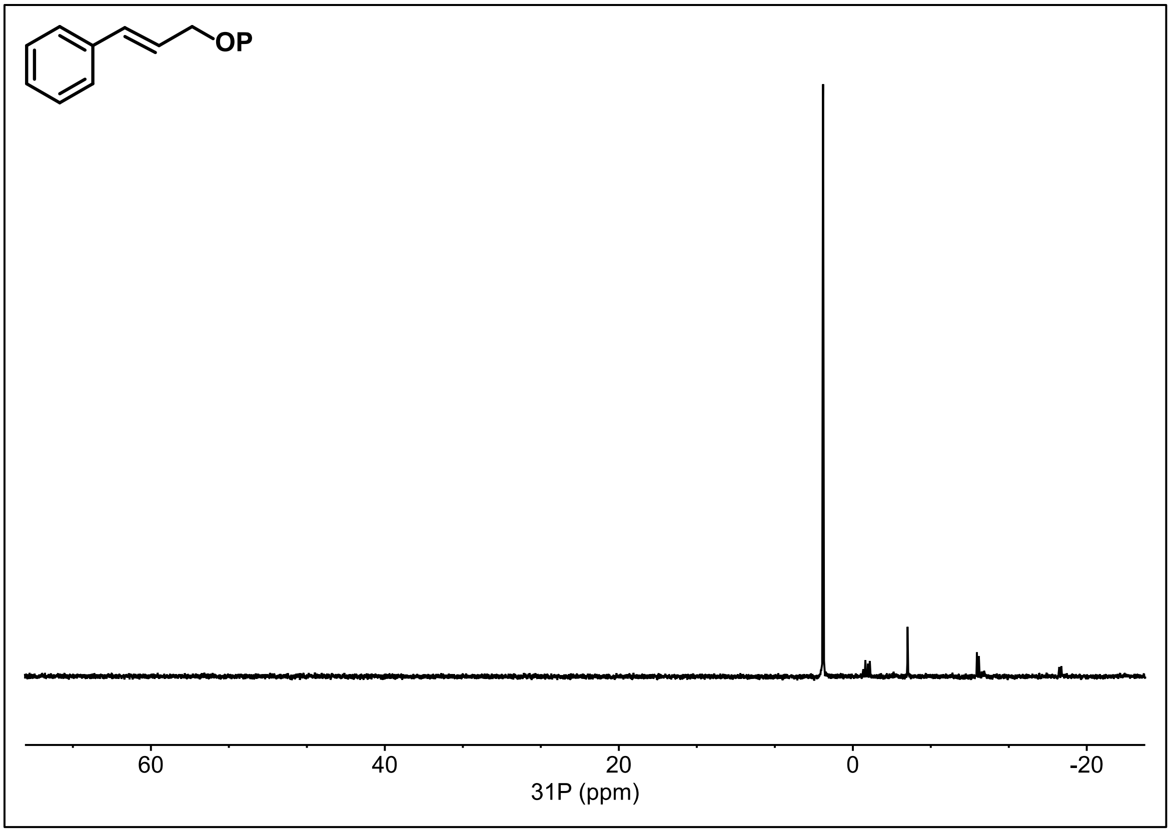

Figure S50. ^{1}H NMR (300 MHz, D_2O) and ^{31}P NMR of **25** (122 MHz, D_2O).

3-Phenylbut-2-en-1-yl Phosphate (26):
The title product was obtained as a white solid from (*E*)-3-phenylbut-2-en-1-ol following the procedure described in *Method 2.7a.*
TLC (^{i}PrOH: NH_4OH: H_2O 7:2:1 v/v): R_f = 0.67.
^{1}H NMR (300 MHz, D_2O): δ 7.66 – 7.43 (m, 1H), 7.50 – 7.18 (m, 4H), 5.99 (dt, *J* = 7.1, 3.6 Hz, 1H), 4.54 (t, *J* = 6.8 Hz, 2H), 2.08 (s, 3H).
^{31}P NMR (122 MHz, D_2O): δ 1.83.
HRMS-ESI: Calculated for $C_{10}H_{12}O_4P$ $[M-H]^-$: 227.04731; Found: 227.0473.

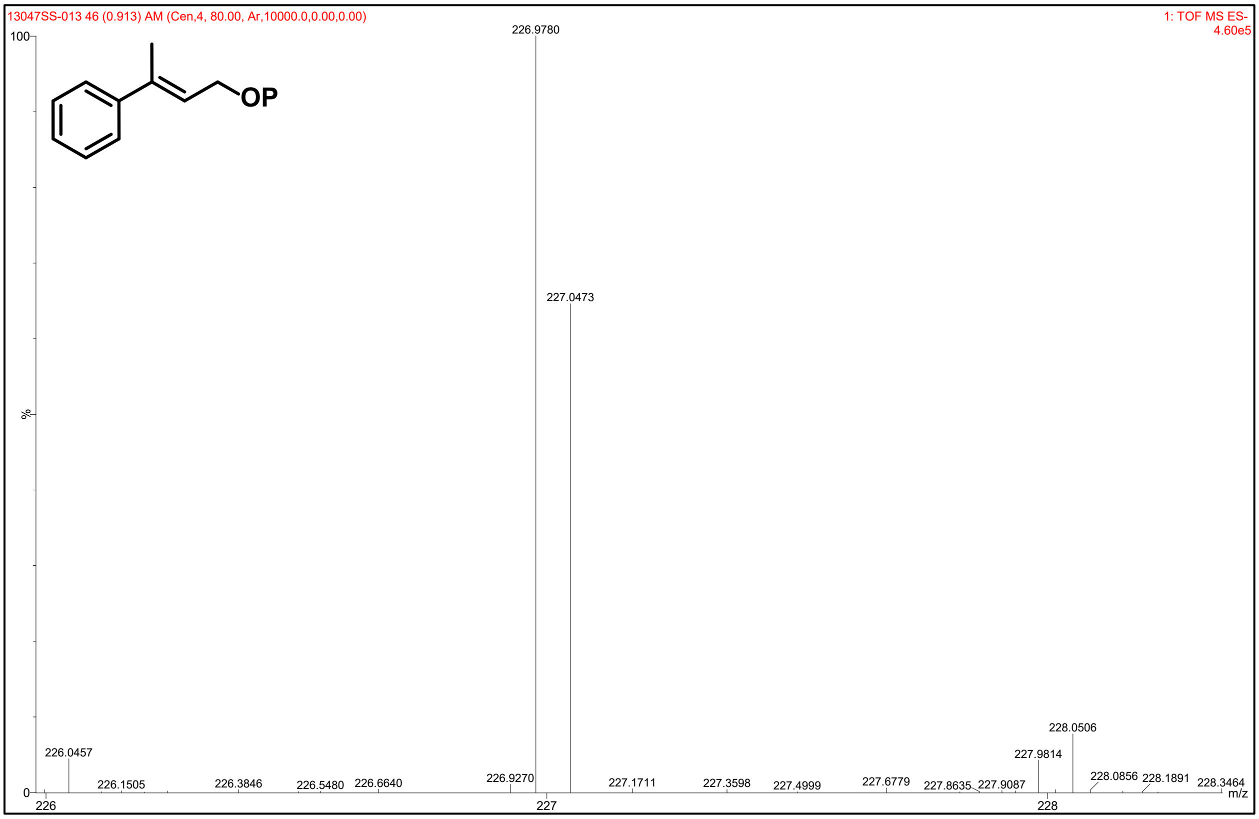

Figure S51. HRMS-ESI$^-$ of **26.**

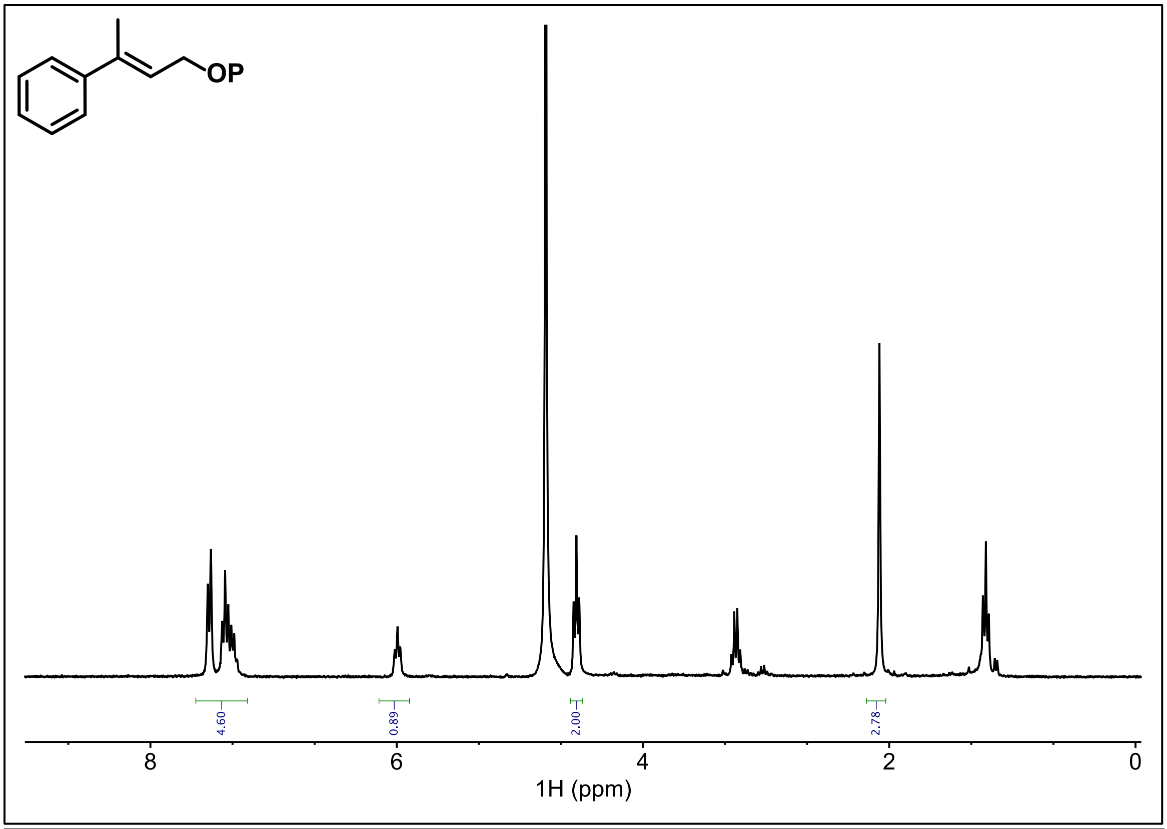

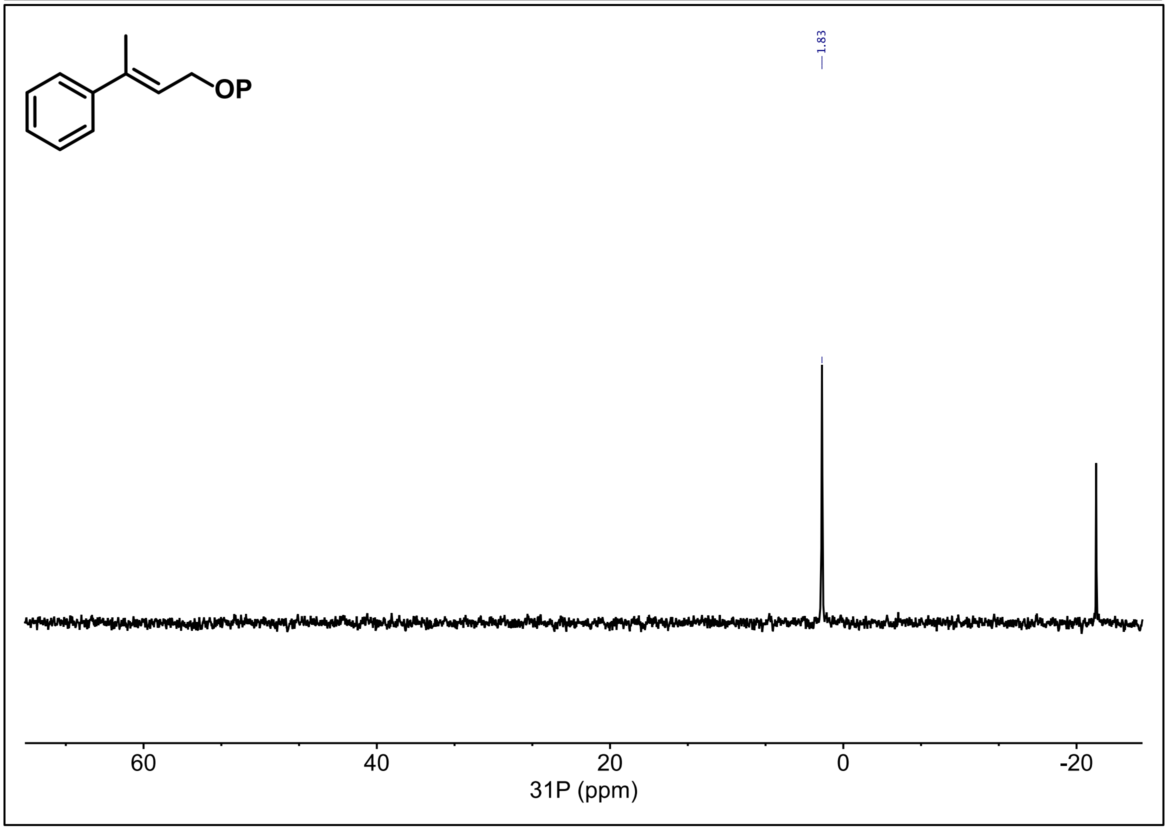

Figure S52. ^{1}H NMR (300 MHz, D_2O) and ^{31}P NMR of **26** (122 MHz, D_2O).

3-Methylhept-2-en-1-yl Phosphate (27):
The title product was obtained as an ivory solid from (*E*)-3-methylhept-2-en-1-ol following the procedure described in *Method 2.7b.*
TLC (^{i}PrOH: NH_4OH: H_2O 7:2:1 v/v): R_f = 0.61.
^{1}H NMR (300 MHz, D_2O): δ 7.32 (d, *J* = 7.8 Hz, 2H), 7.24 (d, *J* = 7.8 Hz, 2H), 4.82 (d, *J* = 6.7 Hz, 2H), 2.31 (s, 3H).
^{31}P NMR (122 MHz, D_2O): δ 0.72.
MS-ESI: Calculated for $C_8H_{16}O_4P$ [M-H]$^-$: 207.07861; Found: 207.0798.

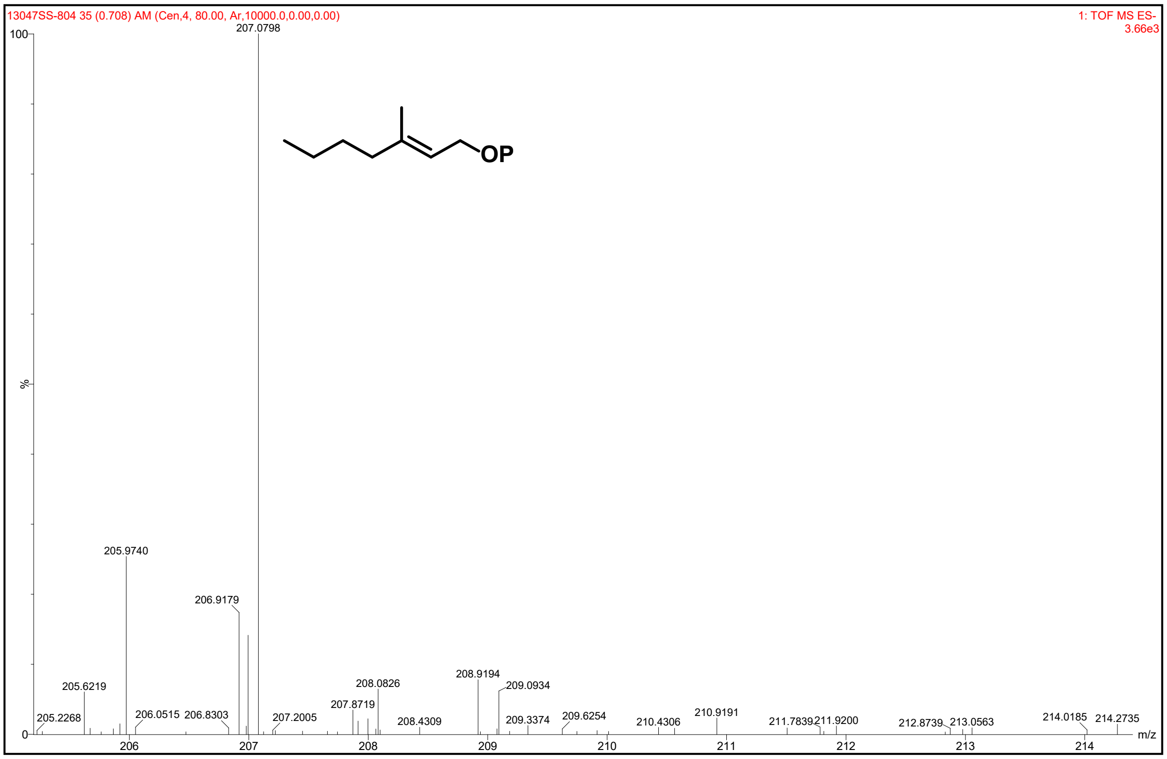

Figure S53. MS-ESI$^-$ of **27.**

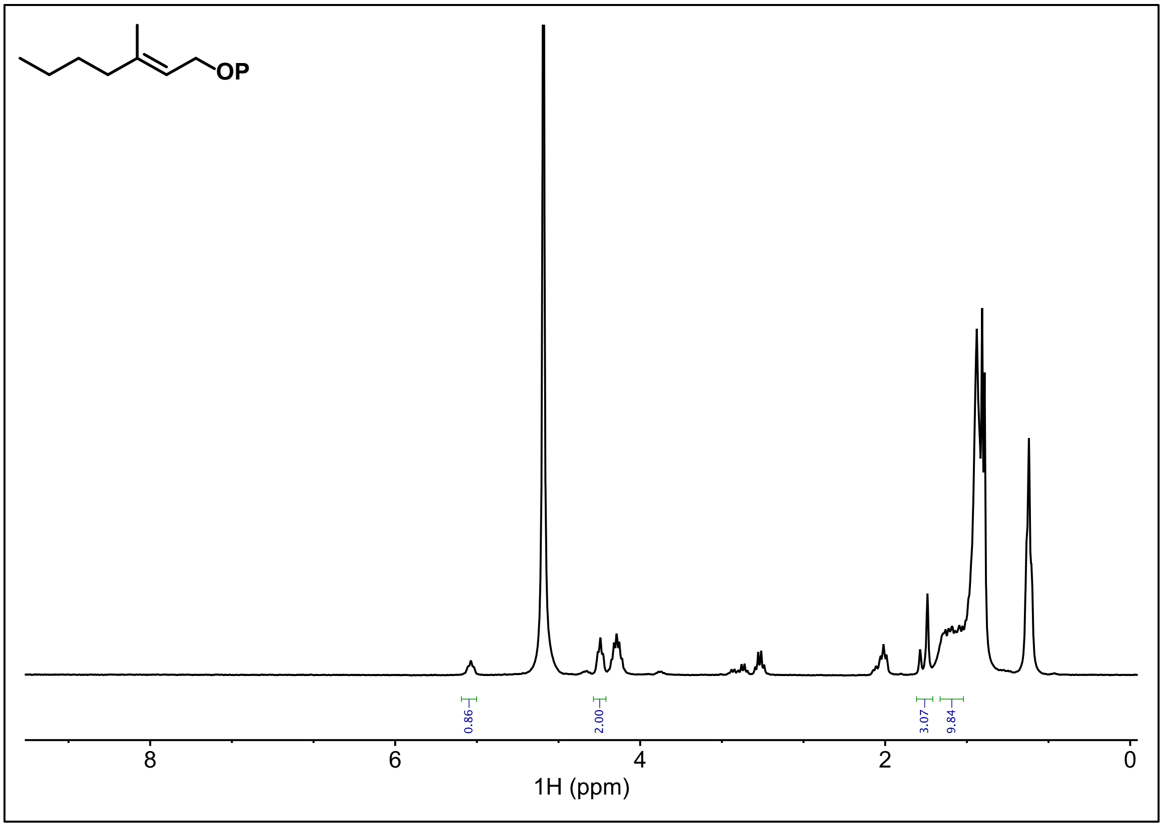

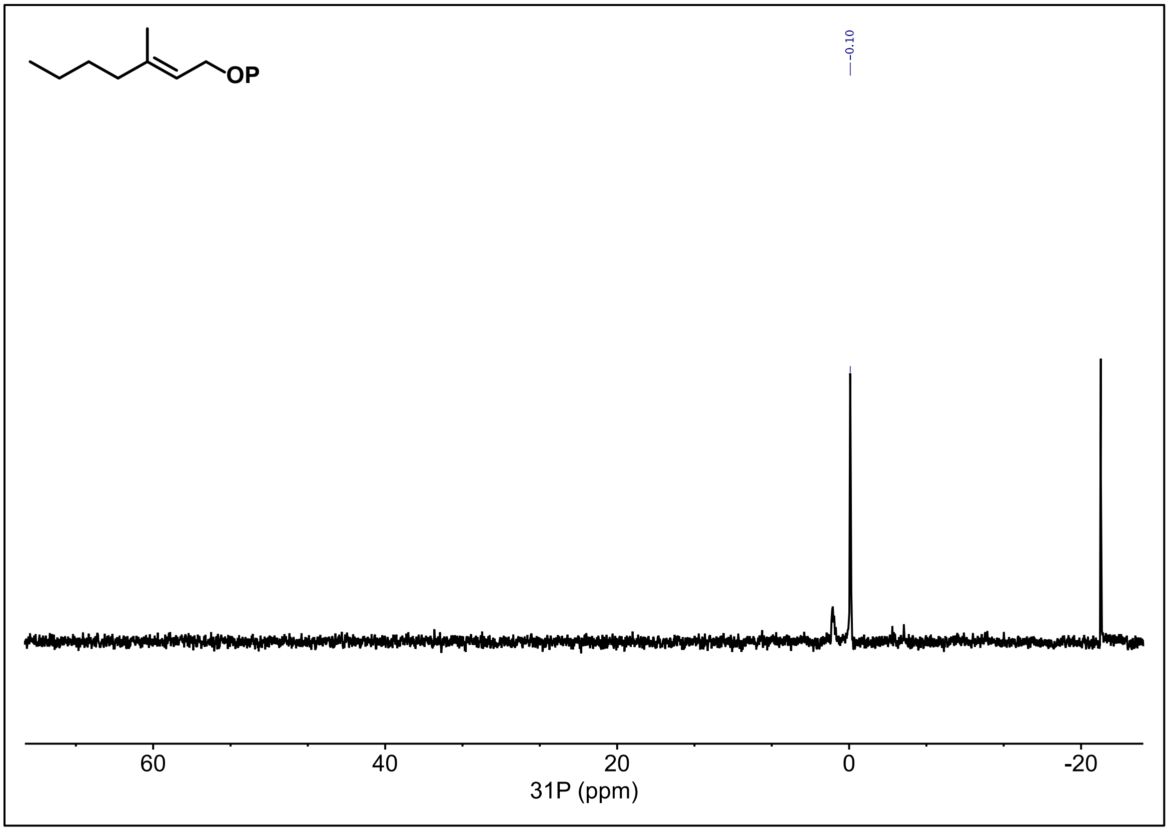

Figure S54. ^{1}H NMR (300 MHz, D_2O) and ^{31}P NMR of **27** (122 MHz, D_2O).

3-Methylhepta-2,6-dien-1-yl Phosphate (28):

The title product was obtained as a white solid from (*E*)-3-methylhepta-2,6-dien-1-ol following the procedure described in *Method 2.7b.*

TLC (^{i}PrOH: NH_4OH: H_2O 7:2:1 v/v): R$_f$ = 0.66.

^{1}H NMR (400 MHz, D_2O): δ 5.91 (ddt, *J* = 17.0, 11.7, 6.0 Hz, 1H), 5.44 (t, *J* = 7.1 Hz, 1H), 5.09 (d, *J* = 17.3 Hz, 1H), 5.00 (d, *J* = 10.1 Hz, 1H), 4.37 (t, *J* = 6.6 Hz, 2H), 2.28 – 2.13 (m, 4H), 1.71 (s, 3H).

^{31}P NMR (162 MHz, D_2O): δ 1.75.

HRMS-ESI: Calculated for $C_8H_{14}O_4P$ [M-H]$^-$: 205.06296; Found: 205.0629.

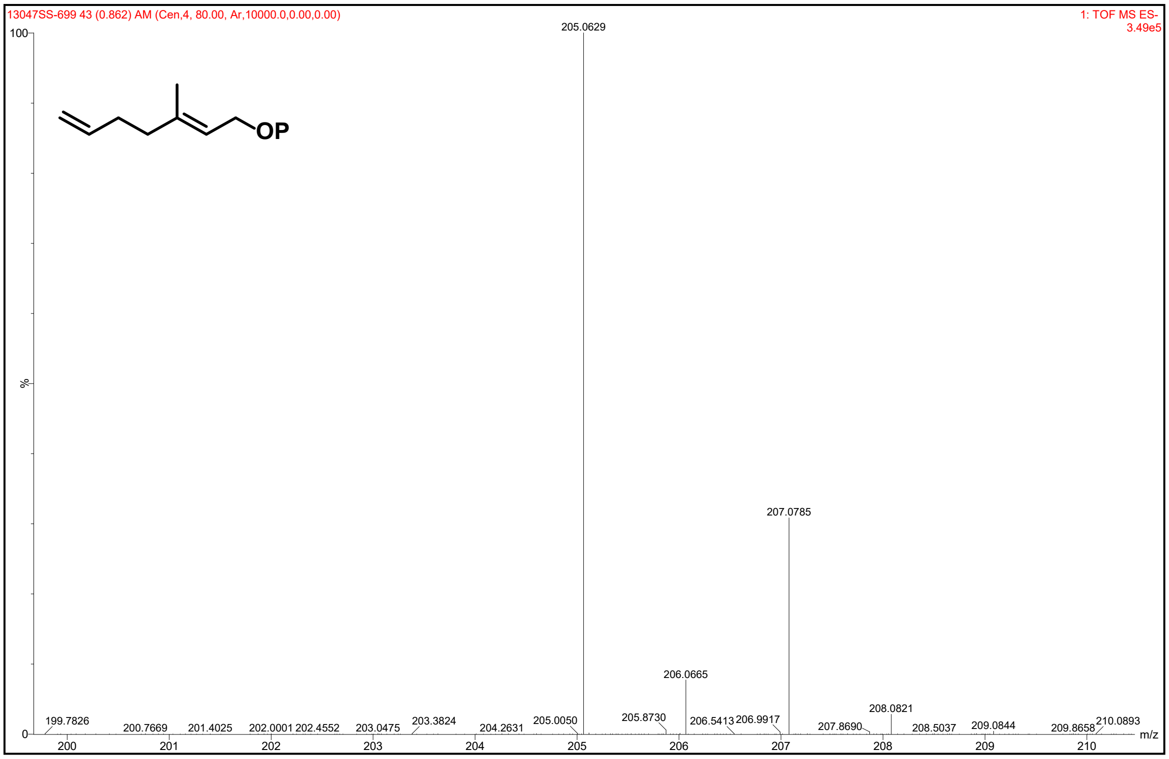

Figure S55. HRMS-ESI$^-$ of **28.**

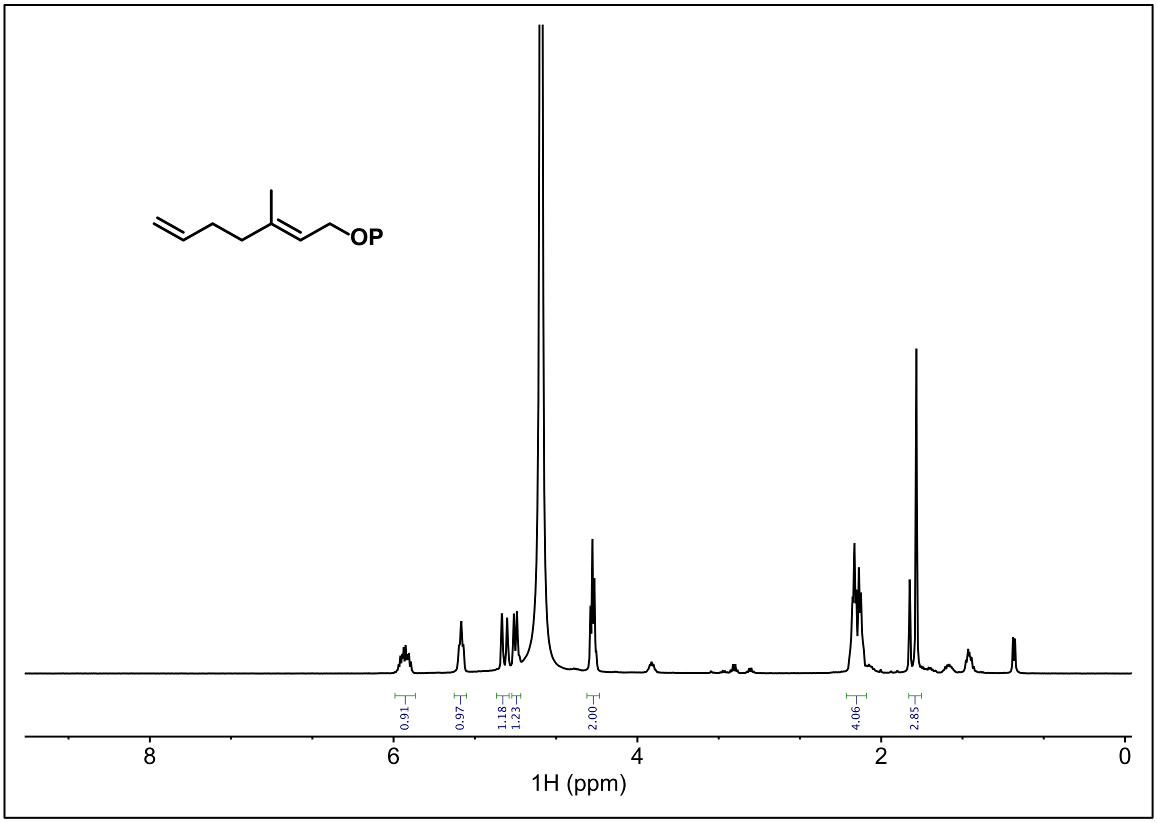

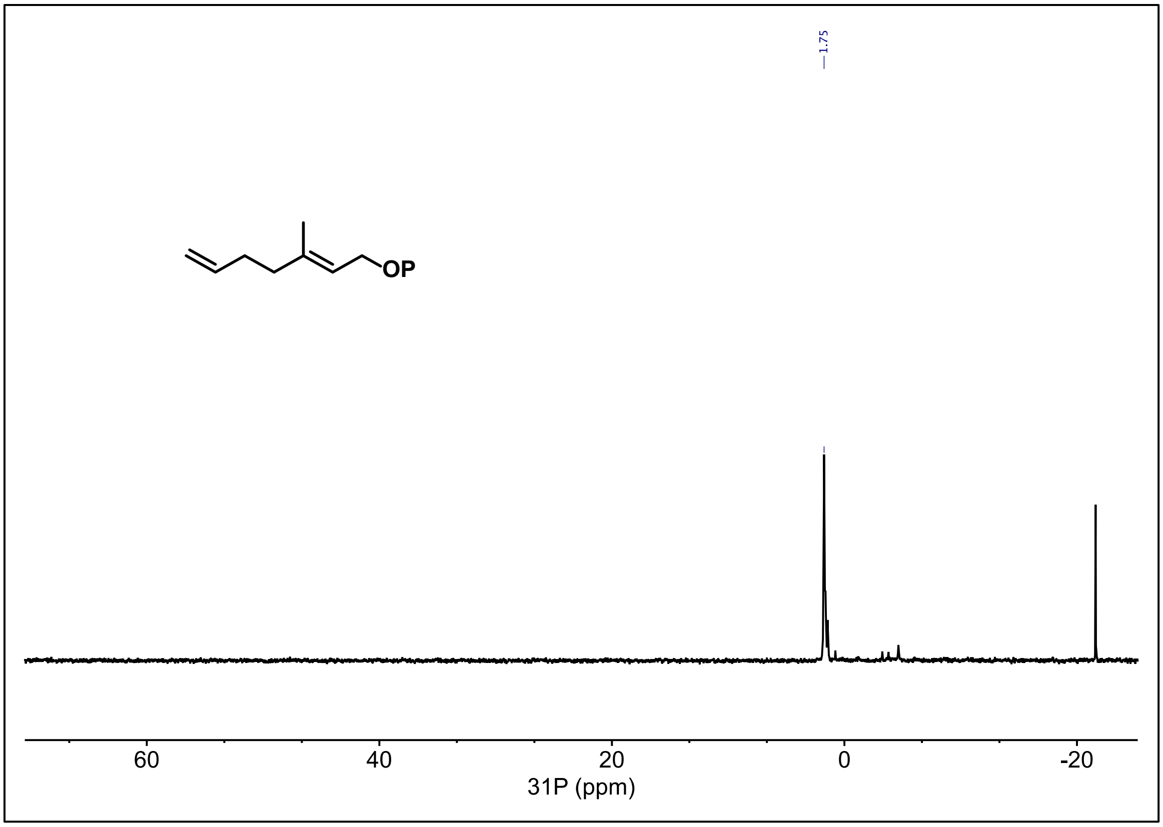

Figure S56. ^{1}H NMR (400 MHz, D_2O) and ^{31}P NMR of **28** (162 MHz, D_2O).

3-Methylhept-2-en-6-yn-1-yl Phosphate (29):

The title product was obtained as a yellow solid from (*E*)-3-methylhept-2-en-6-yn-1-ol following the procedure described in *Method 2.7b.*

TLC (^{i}PrOH: NH_4OH: H_2O 7:2:1 v/v): R_f = 0.68.

^{1}H NMR (400 MHz, D_2O): δ 5.50 (d, *J* = 6.8 Hz, 1H), 4.44 (t, *J* = 6.9 Hz, 2H), 3.51 – 3.36 (m, 2H), 2.50 – 2.33 (m, 2H), 2.29 (t, *J* = 7.3 Hz, 2H), 1.73 (s, 3H).

^{31}P NMR (162 MHz, D_2O): δ 0.54.

HRMS-ESI: Calculated for $C_8H_{12}O_4P$ $[M-H]^-$: 203.04731; Found: 203.0476.

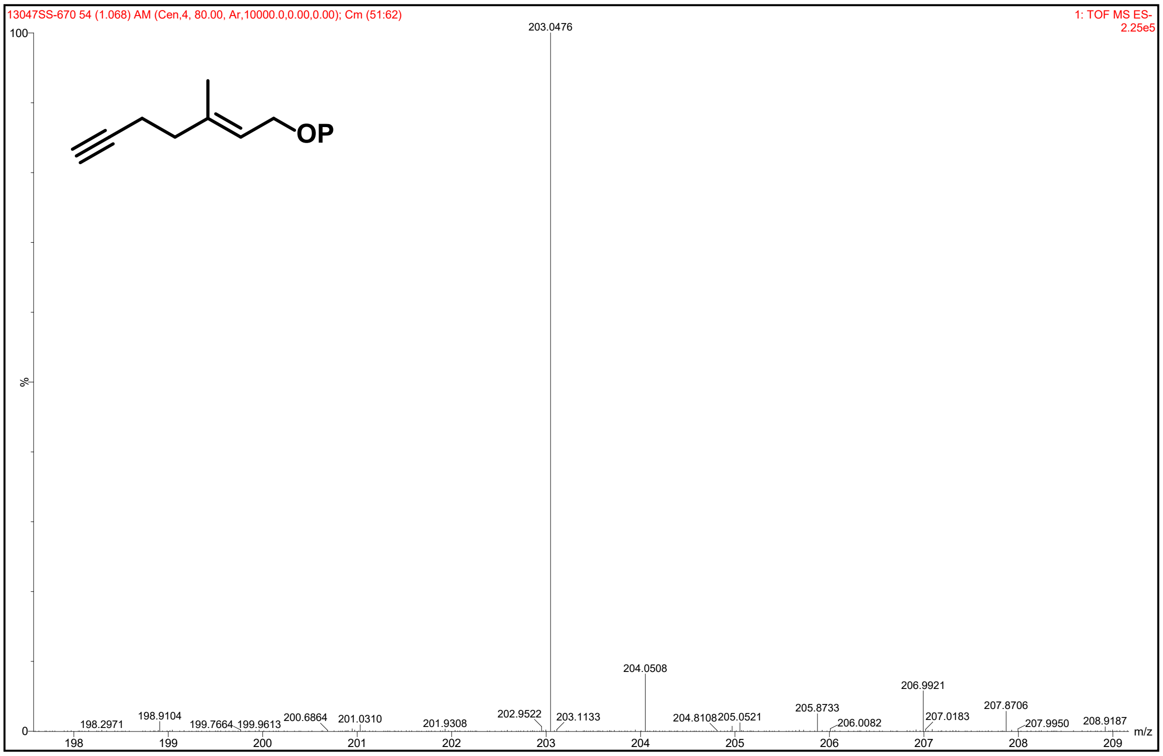

Figure S57. HRMS-ESI$^-$ of **29.**

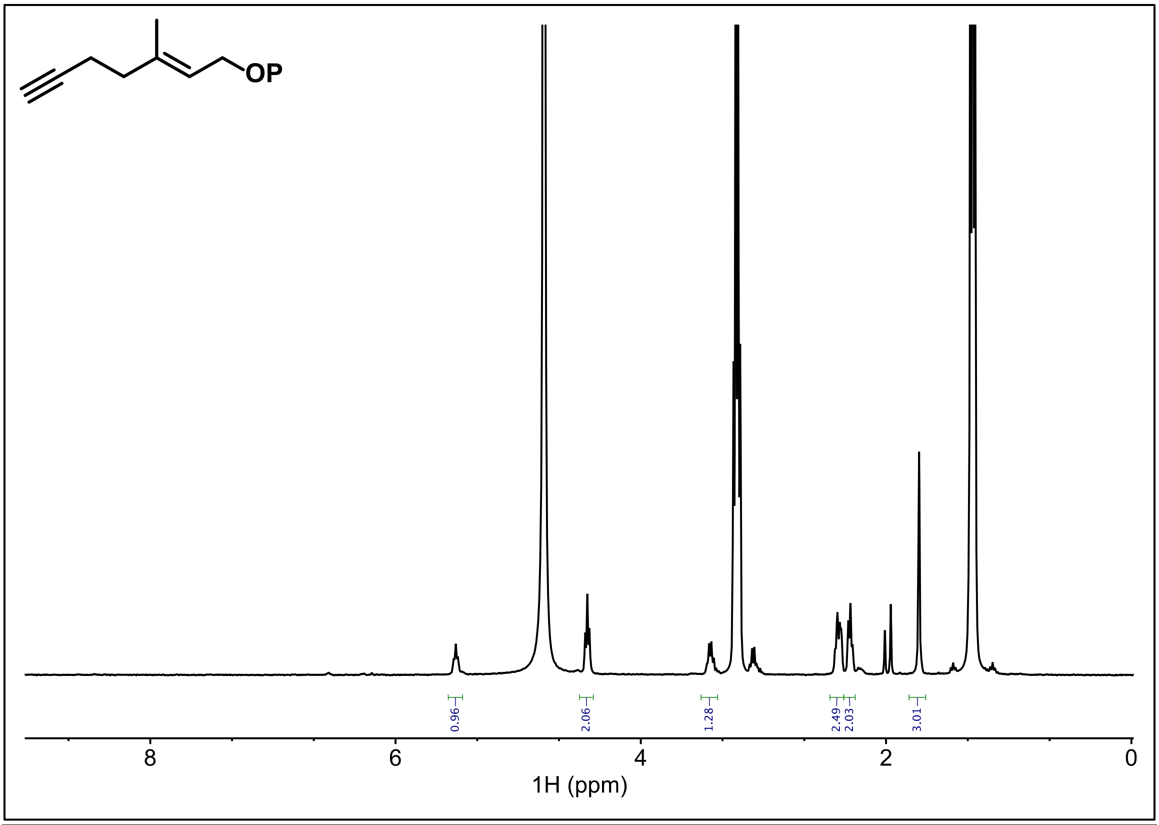

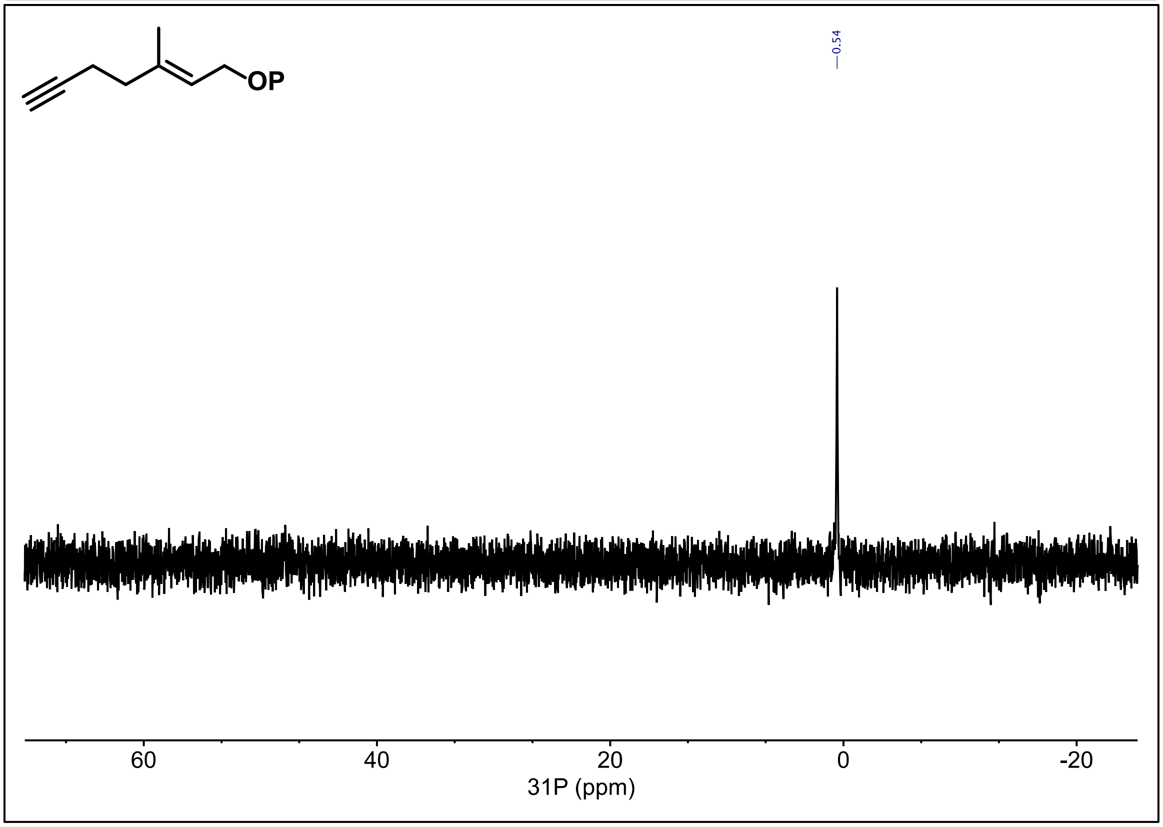

Figure S58. ^{1}H NMR (400 MHz, D_2O) and ^{31}P NMR of **29** (162 MHz, D_2O).

3,6-Dimethylhept-2-en-1-yl Phosphate (30):

The title product was obtained as an ivory solid from (*E*)-3,6-dimethylhept-2-en-1-ol following the procedure described in *Method 2.7b.*

<u>TLC (^{i}PrOH: NH_4OH: H_2O 7:2:1 v/v):</u> $R_f = 0.61$.

<u>^{1}H NMR (400 MHz, D_2O):</u> δ 5.37 (t, *J* = 7.3 Hz, 1H), 4.37 (t, *J* = 6.7 Hz, 2H), 1.90 (d, *J* = 7.3 Hz, 2H), 1.82 – 1.73 (m, 1H), 1.65 (s, 3H), 1.24 (s, 2H), 0.82 (d, *J* = 6.7 Hz, 6H).

<u>^{31}P NMR (162 MHz, D_2O):</u> δ 0.99.

<u>HRMS-ESI:</u> Calculated for $C_9H_{18}O_4P$ $[M-H]^-$: 221.09426; Found: 221.0952.

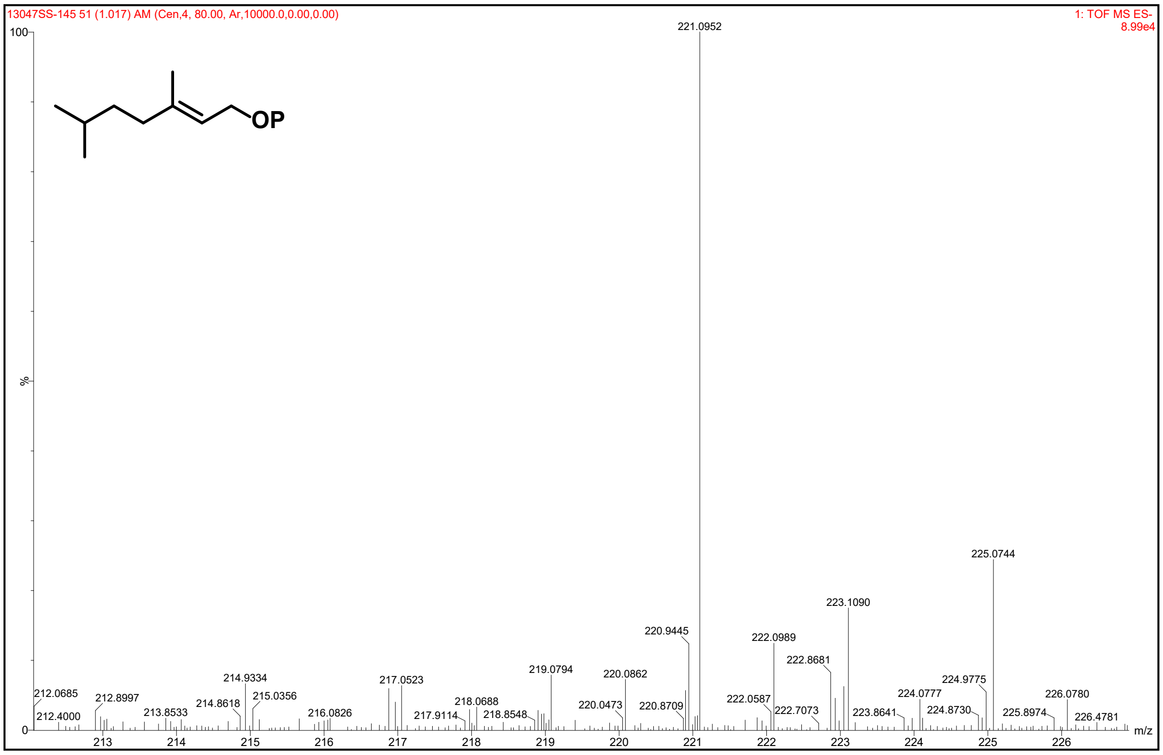

Figure S59. HRMS-ESI$^-$ of **30.**

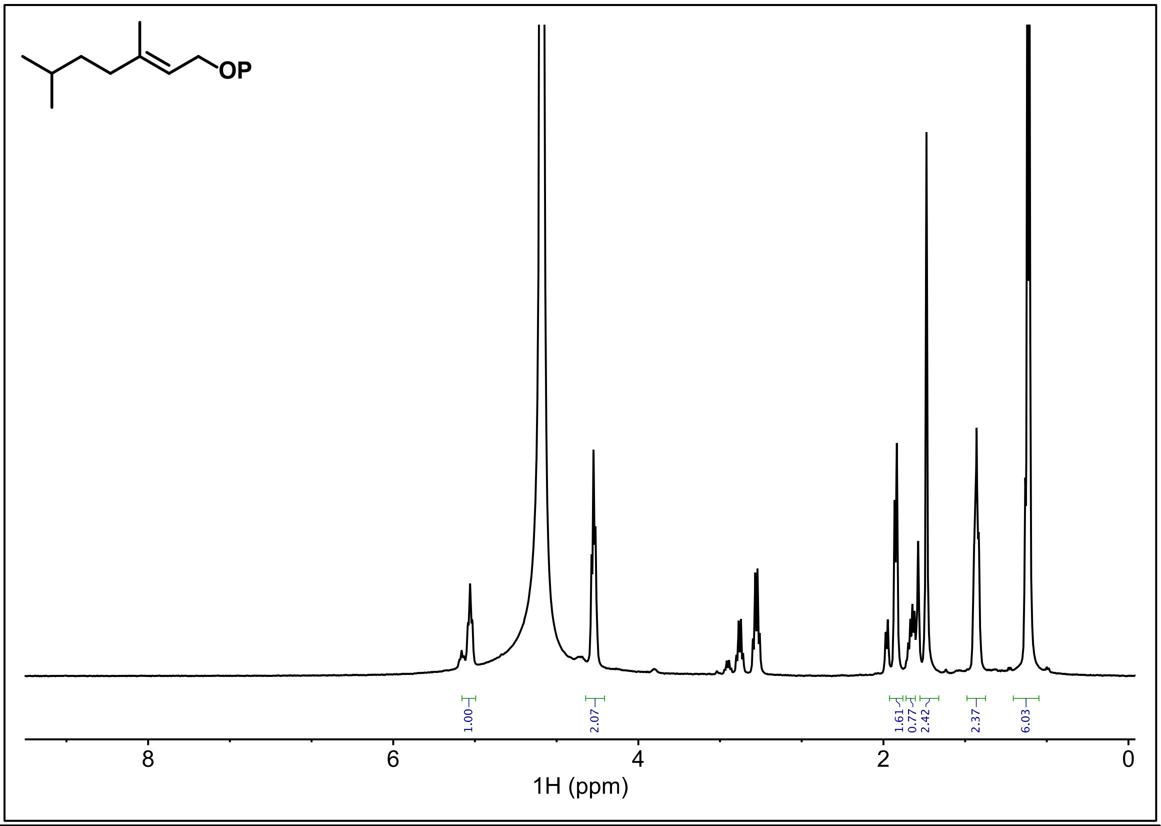

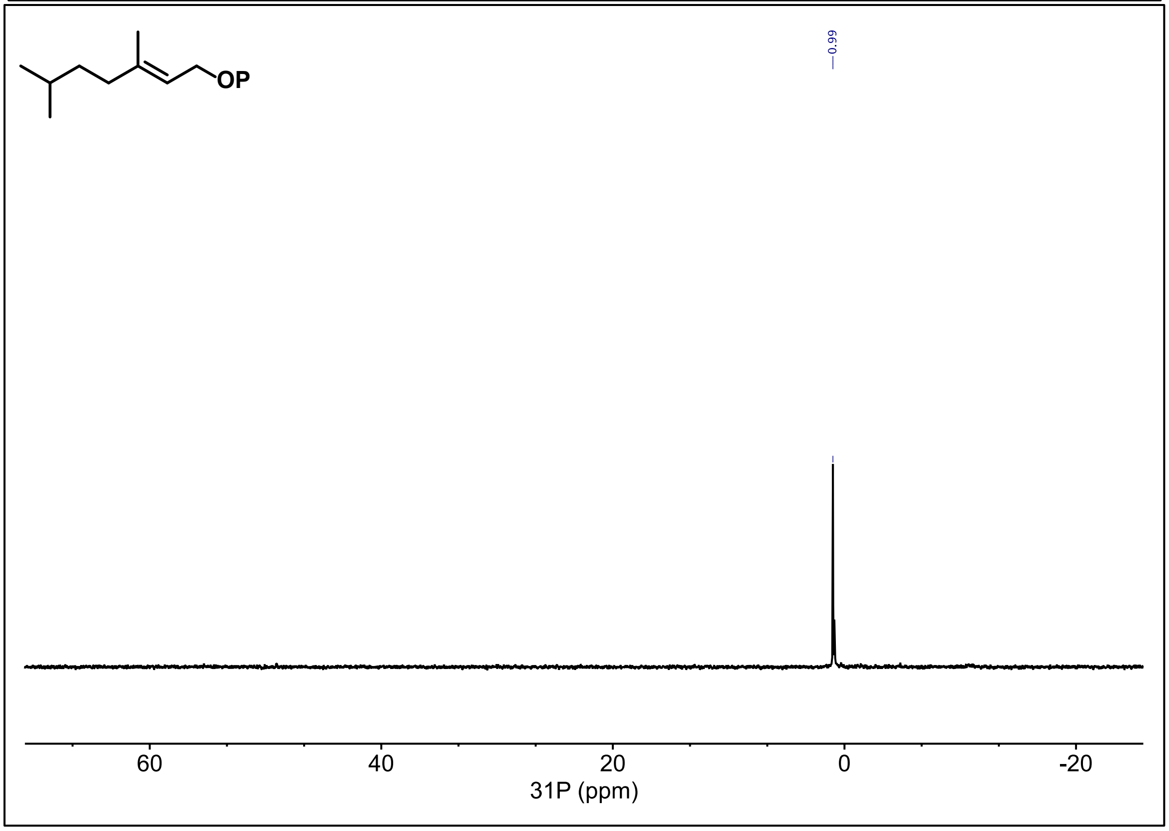

Figure S60. ^{1}H NMR (400 MHz, D_2O) and ^{31}P NMR of **30** (162 MHz, D_2O).

(*1E,5E*)-7-(Benzyloxy)-2,6-dimethylhepta-1,5-dien-1-yl Phosphate (31):
The title product was obtained as a brown solid from (*S*)-(4-(prop-1-en-2-yl)cyclohex-1-en-1-yl)methanol following the procedure described in *Method 2.7b.*
TLC (^{i}PrOH: NH_4OH: H_2O 7:2:1 v/v): R_f = 0.62.
^{1}H NMR (300 MHz, D_2O): δ 5.66 (s, 1H), 4.65 (d, *J* = 1.5 Hz, 2H), 4.16 – 3.94 (m, 2H), 2.23 – 1.83 (m, 4H), 1.76 (dd, *J* = 40.2, 13.7 Hz, 2H), 1.59 (s, 3H), 1.42 – 1.24 (m, 1H).
^{31}P NMR (122 MHz, D_2O): δ 0.54.
HRMS-ESI: Calculated for $C_{10}H_{16}O_4P$ [M-H]$^-$: 231.07861; Found: 231.0791.

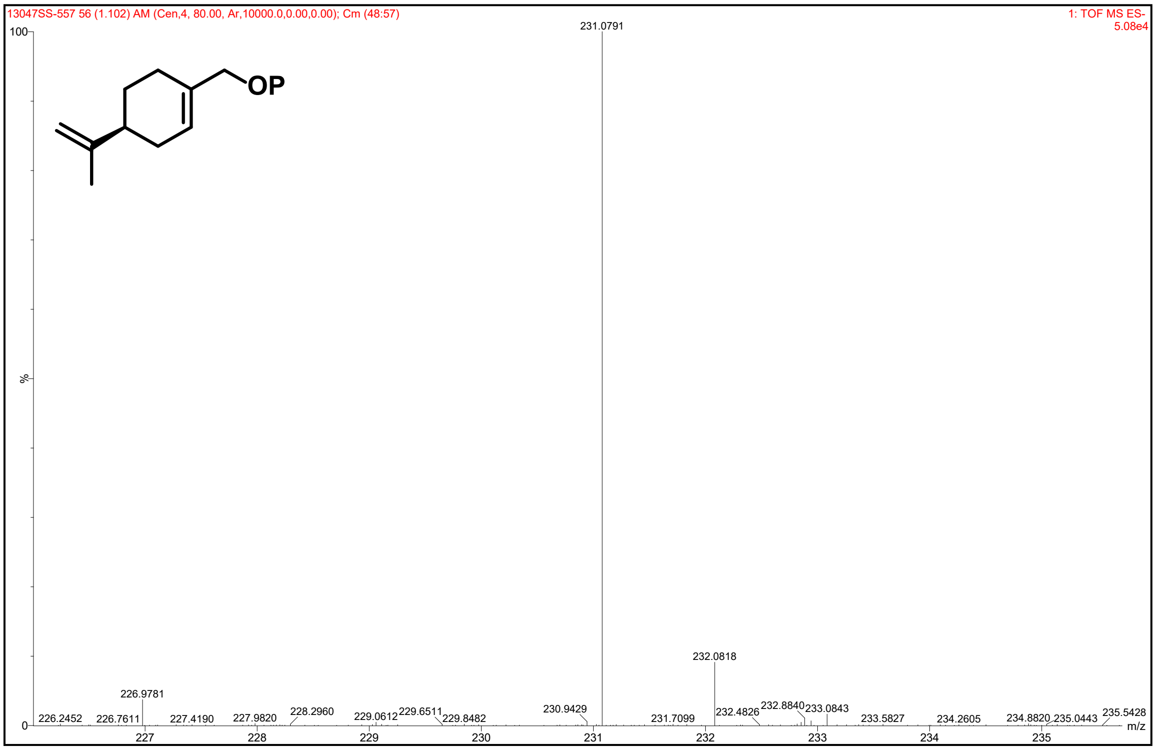

Figure S61. HRMS-ESI$^-$ of **31.**

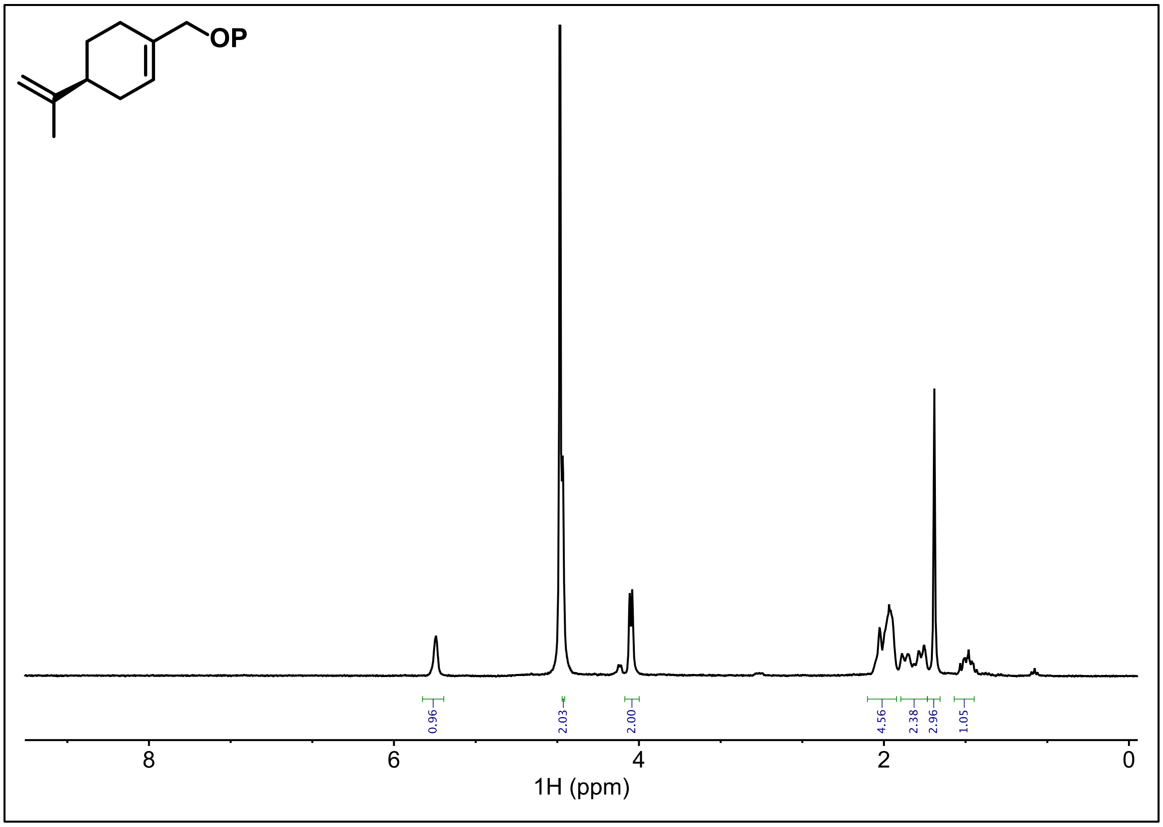

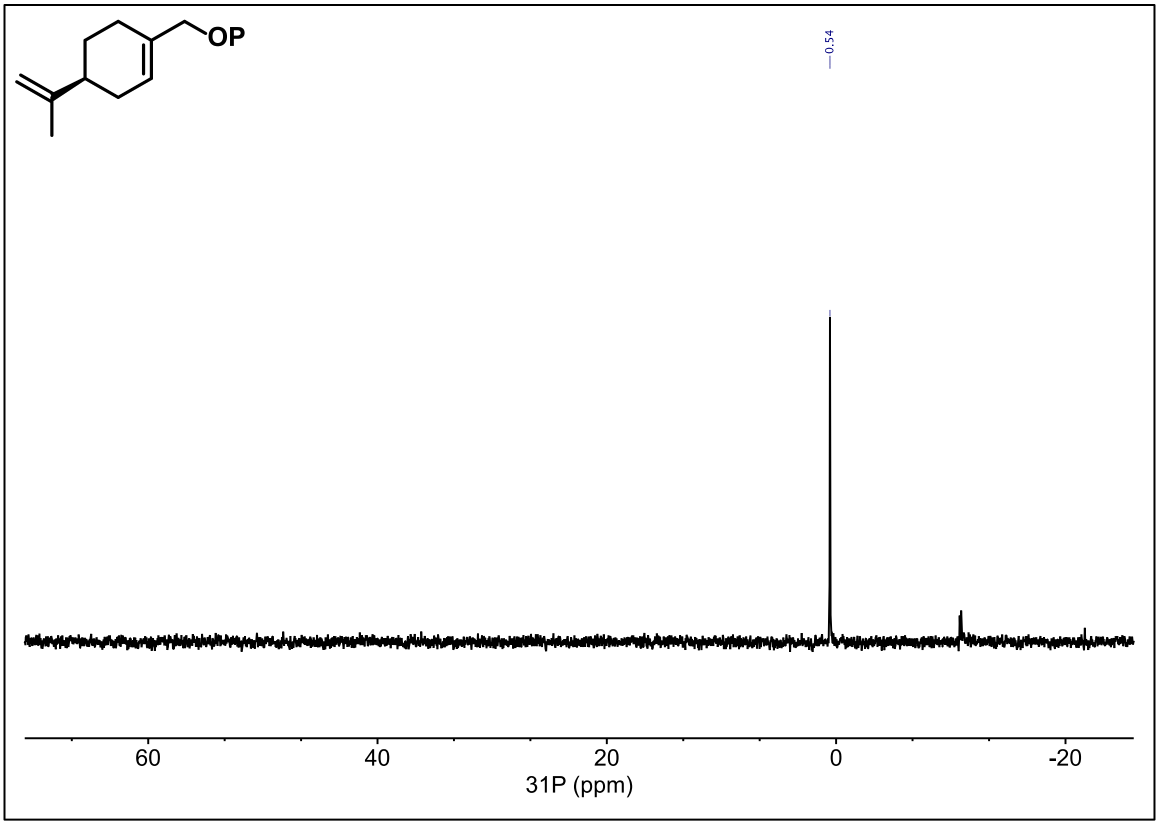

Figure S62. ^{1}H NMR (300 MHz, D_2O) and ^{31}P NMR of **31** (122 MHz, D_2O)**.**

4-Azidobut-2-en-1-yl Phosphate (32):

The title product was obtained as a brown solid from (*E*)-4-azidobut-2-en-1-ol 1 following the procedure described in *Method 2.7b.*

TLC (^{i}PrOH: NH_4OH: H_2O 7:2:1 v/v): R_f = 0.64.

^{1}H NMR (400 MHz, D_2O): δ 5.99 – 5.79 (m, 1H), 5.49 – 5.29 (m, 1H), 4.36 (dd, *J* = 7.7, 5.0 Hz, 2H), 3.83 (d, *J* = 5.7 Hz, 2H).

^{31}P NMR (162 MHz, D_2O): δ 0.70.

HRMS-ESI: Calculated for $C_4H_7N_3O_4P$ $[M-H]^-$: 192.01741; Found: 192.0166.

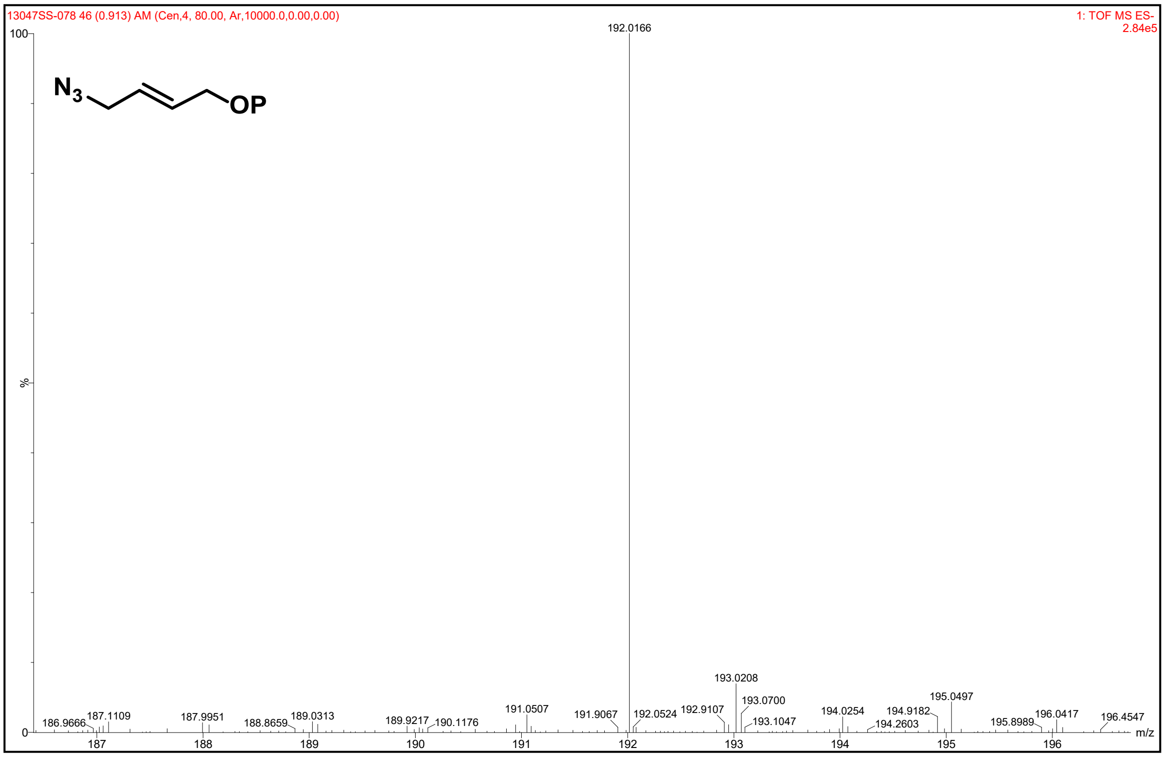

Figure S63. HRMS-ESI$^-$ of **32.**

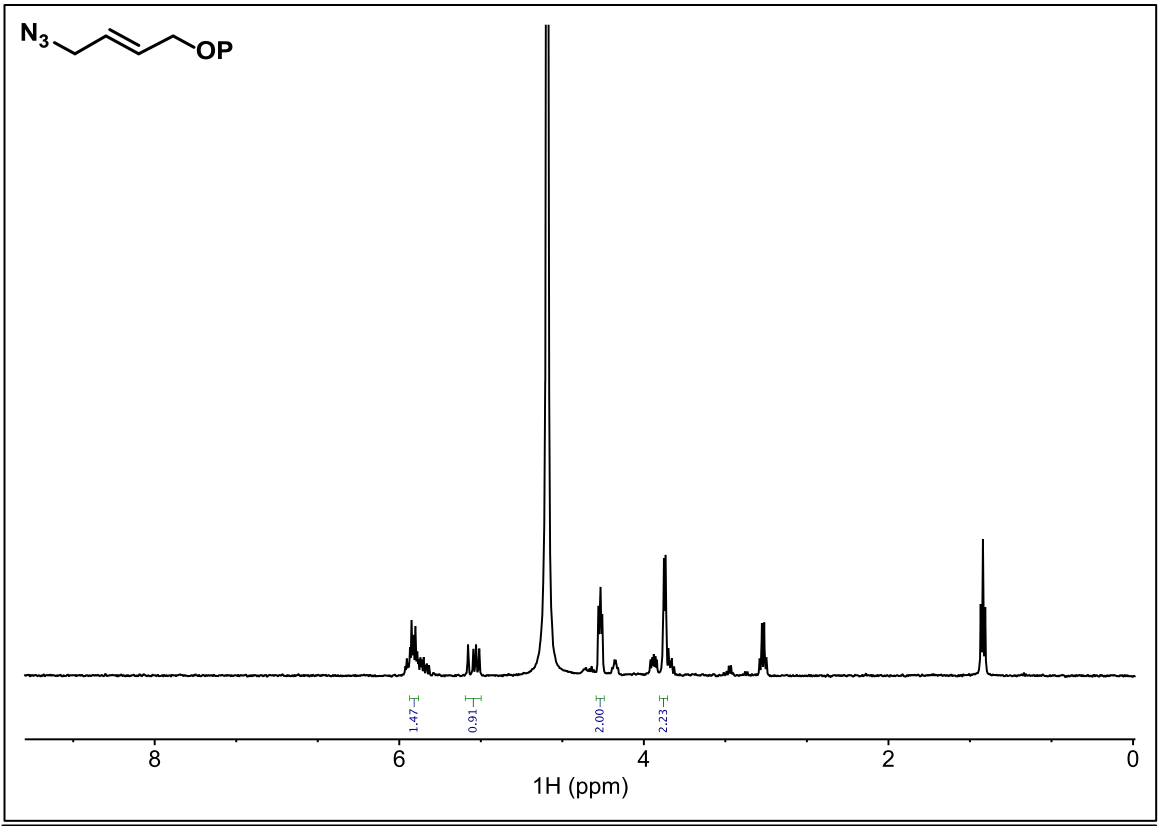

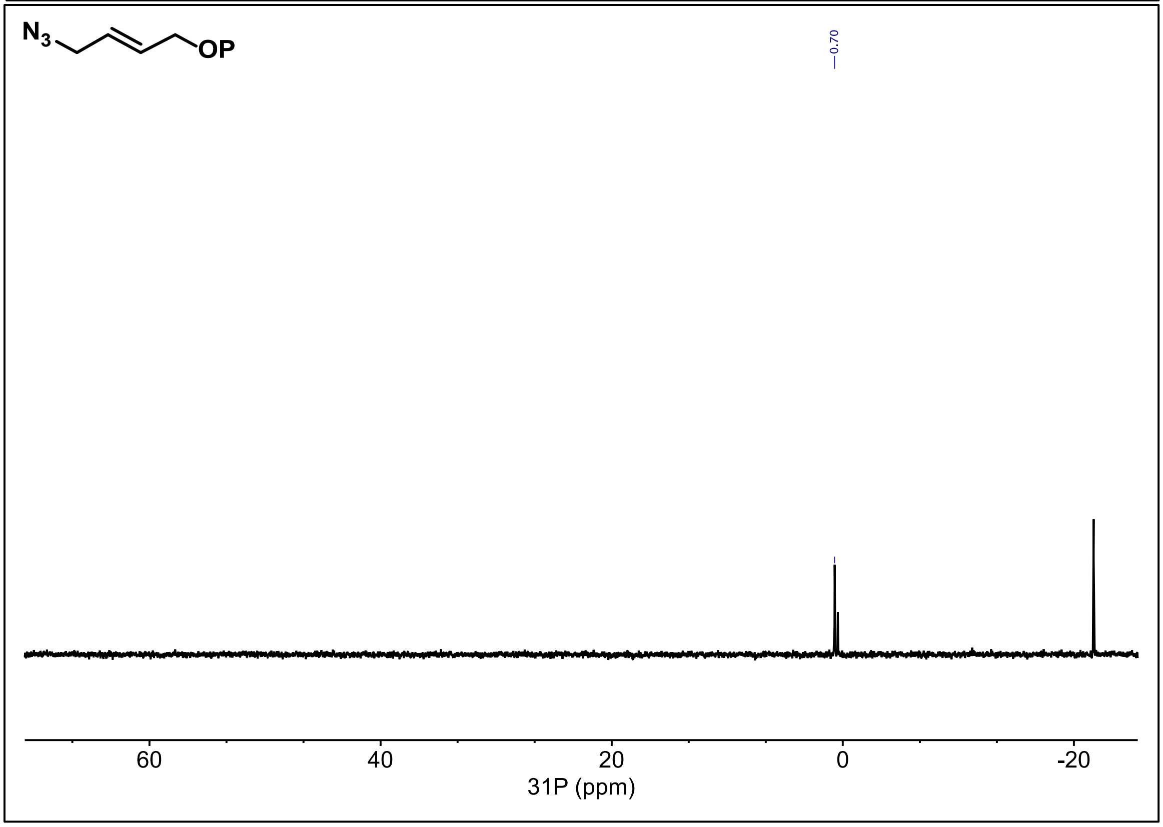

Figure S64. ^{1}H NMR (400 MHz, D_2O) and ^{31}P NMR of **32** (162 MHz, D_2O)**.**

(3-Azidocyclohex-1-en-1-yl)methyl Phosphate (33):

The title product was obtained as a brown solid from (3-azidocyclohex-1-en-1-yl) methanol following the procedure described in *Method 2.7b.*

<u>TLC (^{i}PrOH: NH_4OH: H_2O 7:2:1 v/v):</u> R_f = 0.61.

<u>^{1}H NMR (300 MHz, D_2O):</u> δ 7.01 (d, *J* = 7.4 Hz, 2H), 6.98 – 6.77 (m, 1H), 4.85 (d, *J* = 7.1 Hz, 2H).

<u>^{31}P NMR (122 MHz, D_2O):</u> δ 0.96.

<u>HRMS-ESI:</u> Calculated for $C_7H_{11}N_3O_4P$ [M-H]$^-$: 232.04871; Found: 232.0491.

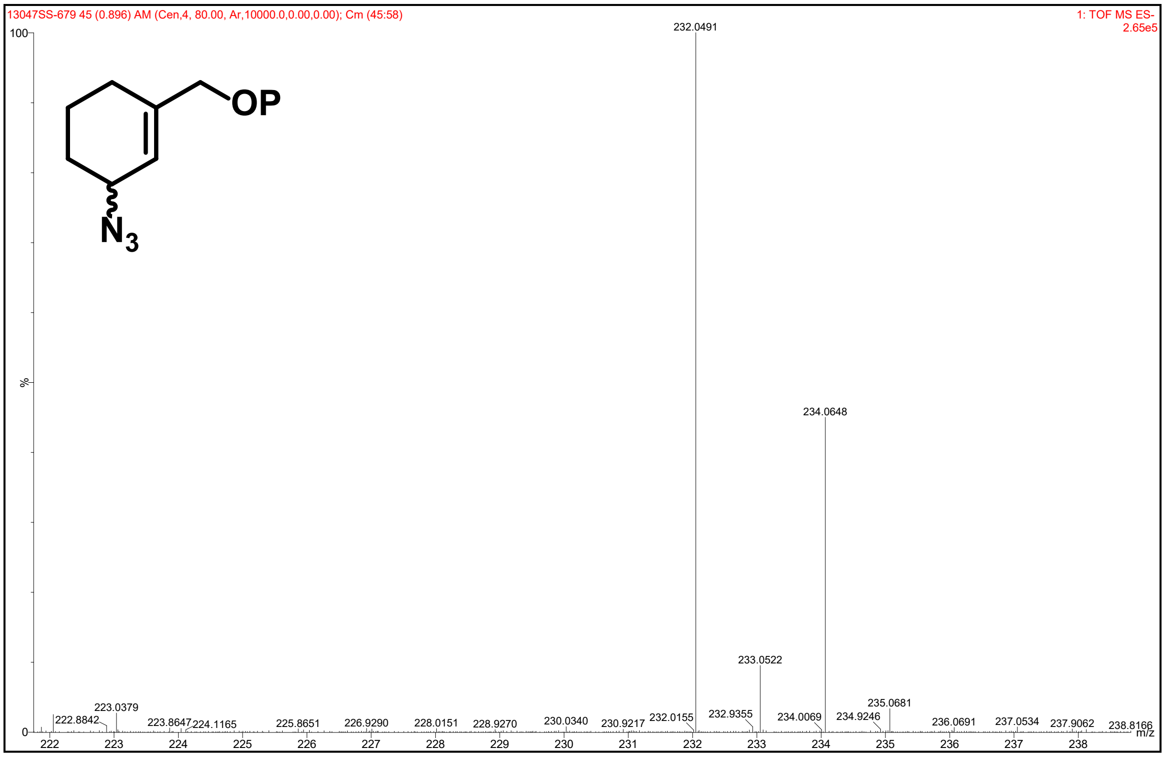

Figure S65. HRMS-ESI$^-$ of **33.**

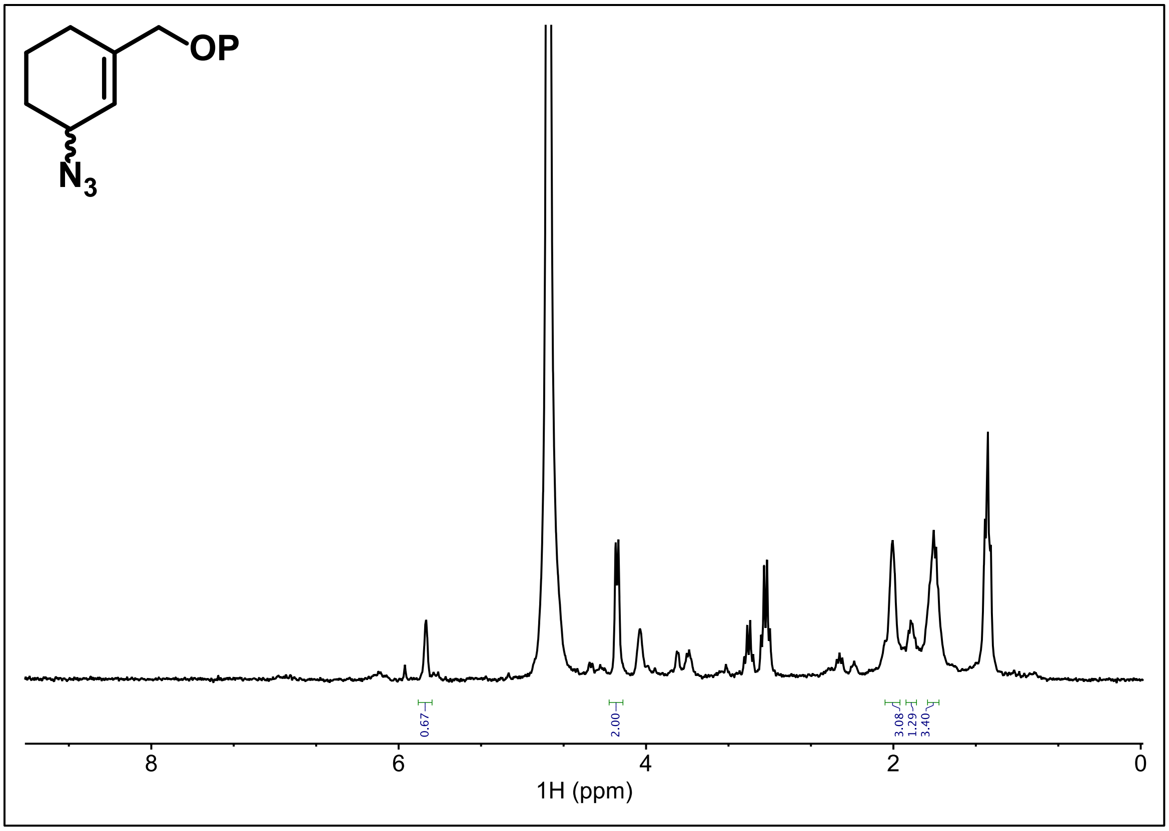

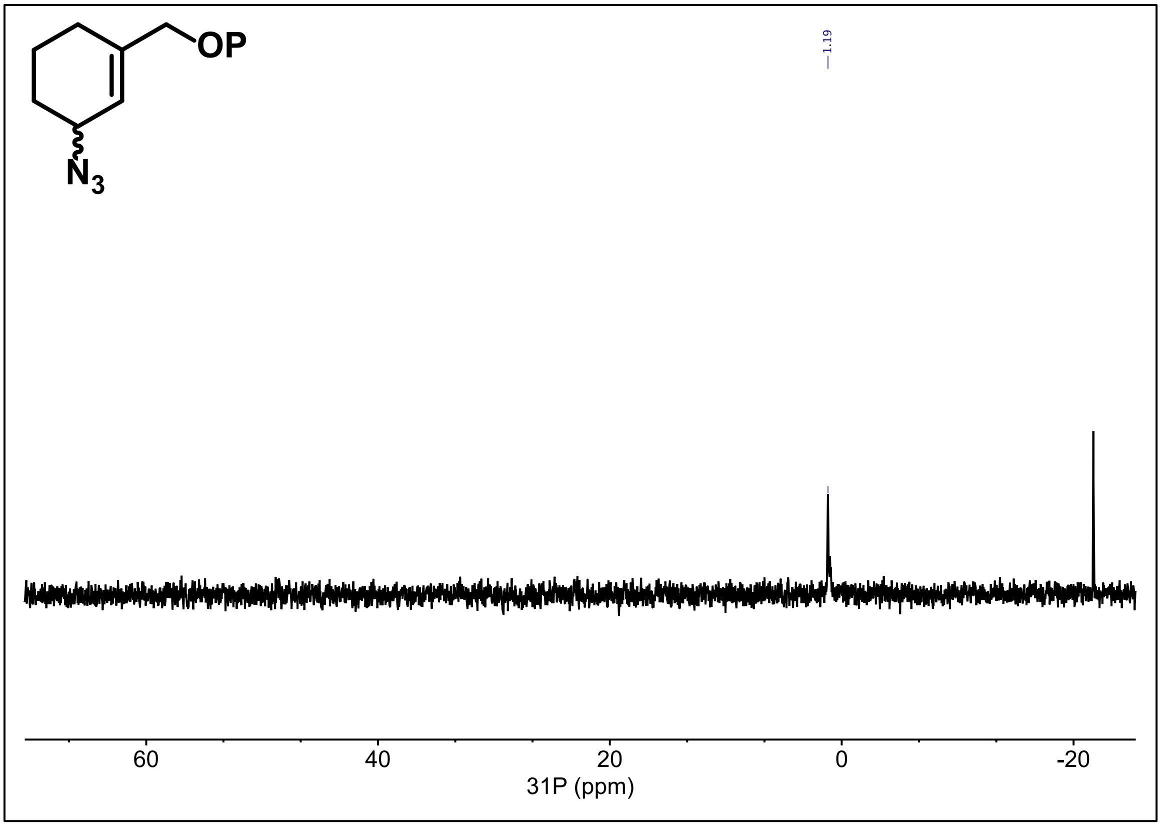

Figure S66. ^{1}H NMR (300 MHz, D_2O) and ^{31}P NMR of **33** (122 MHz, D_2O).

3-Methyloct-2-en-1-yl Phosphate (34):

The title product was obtained as an ivory solid from (*E*)-3-methyloct-2-en-1-ol following the procedure described in *Method 2.7b.*

TLC (^{i}PrOH:NH_4OH:H_2O 7:2:1 v/v): R_f = 0.62.

^{1}H NMR (400 MHz, D_2O): δ 5.44 (dt, *J* = 7.5, 3.4 Hz, 1H), 4.40 (t, *J* = 6.8 Hz, 2H), 1.71 (s, 3H), 1.29 (t, *J* = 7.2 Hz, 8H), 0.89 (t, *J* = 7.3 Hz, 3H).

^{31}P NMR (162 MHz, D_2O): δ 0.86.

MS-ESI: Calculated for $C_9H_{18}O_4P$ [M-H]$^-$: 221.09426; Found: 221.0954.

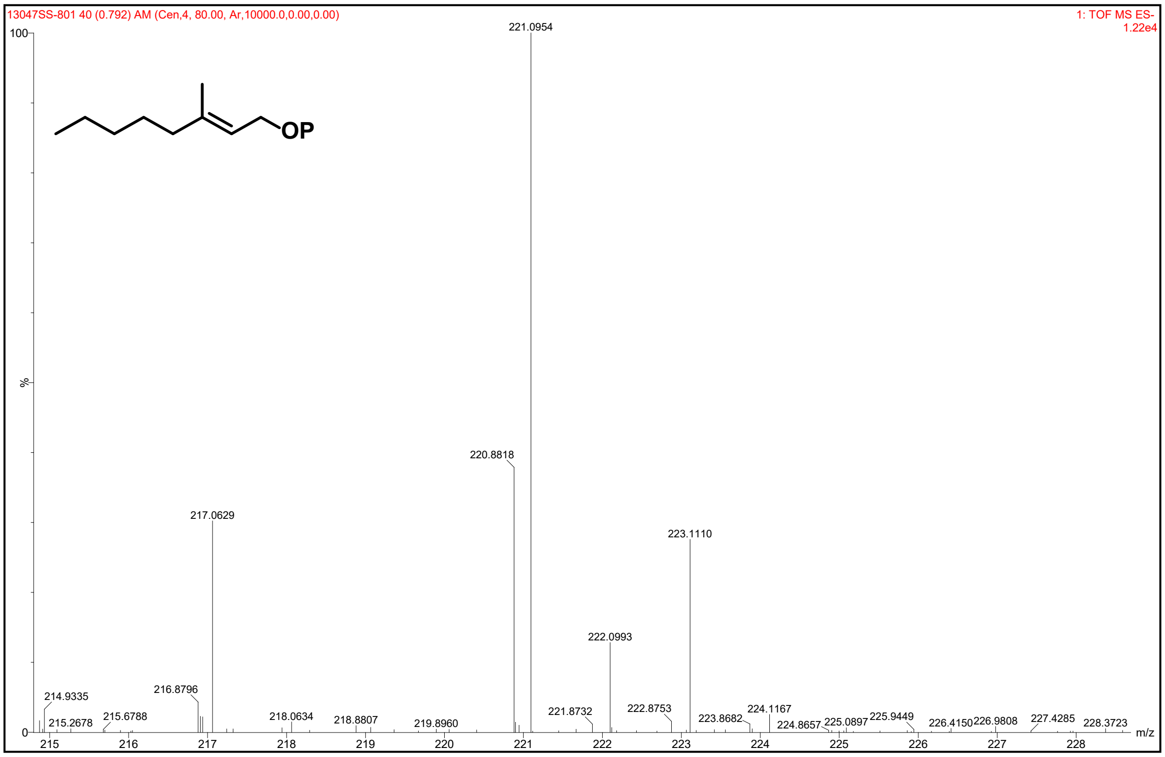

Figure S67. MS-ESI$^-$ of **34.**

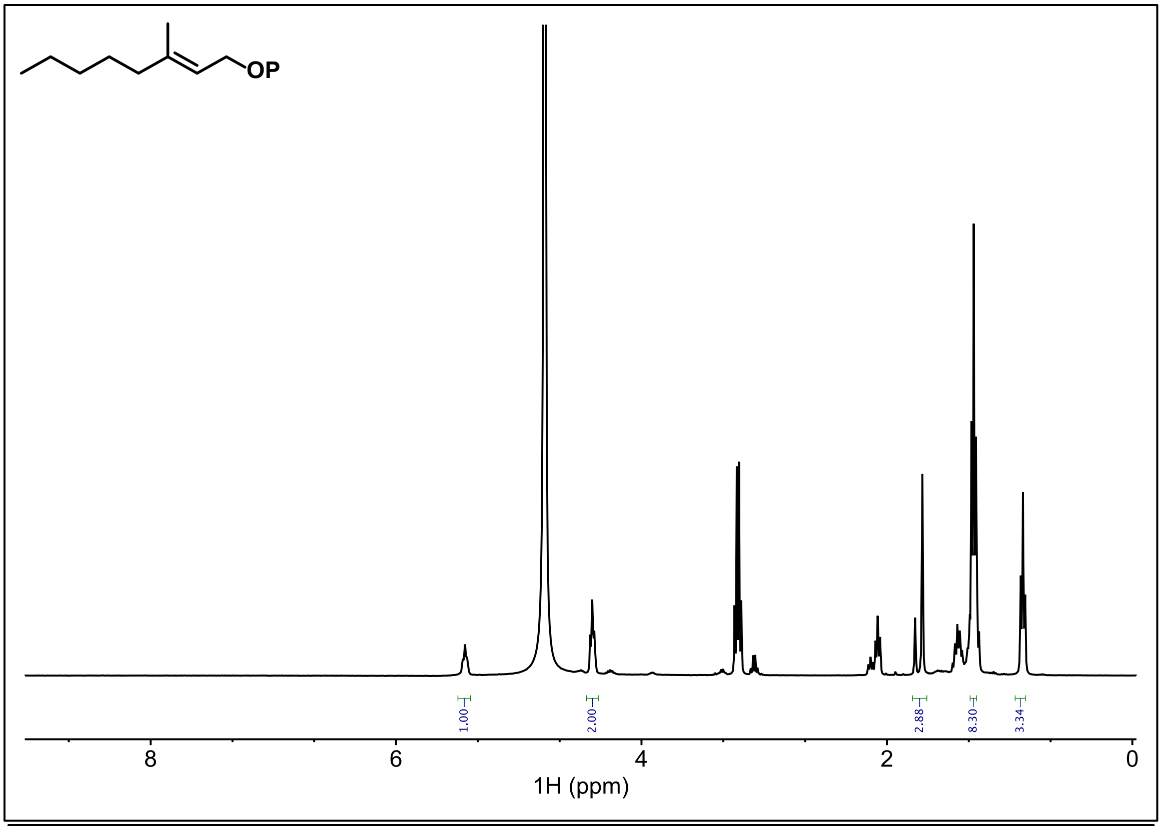

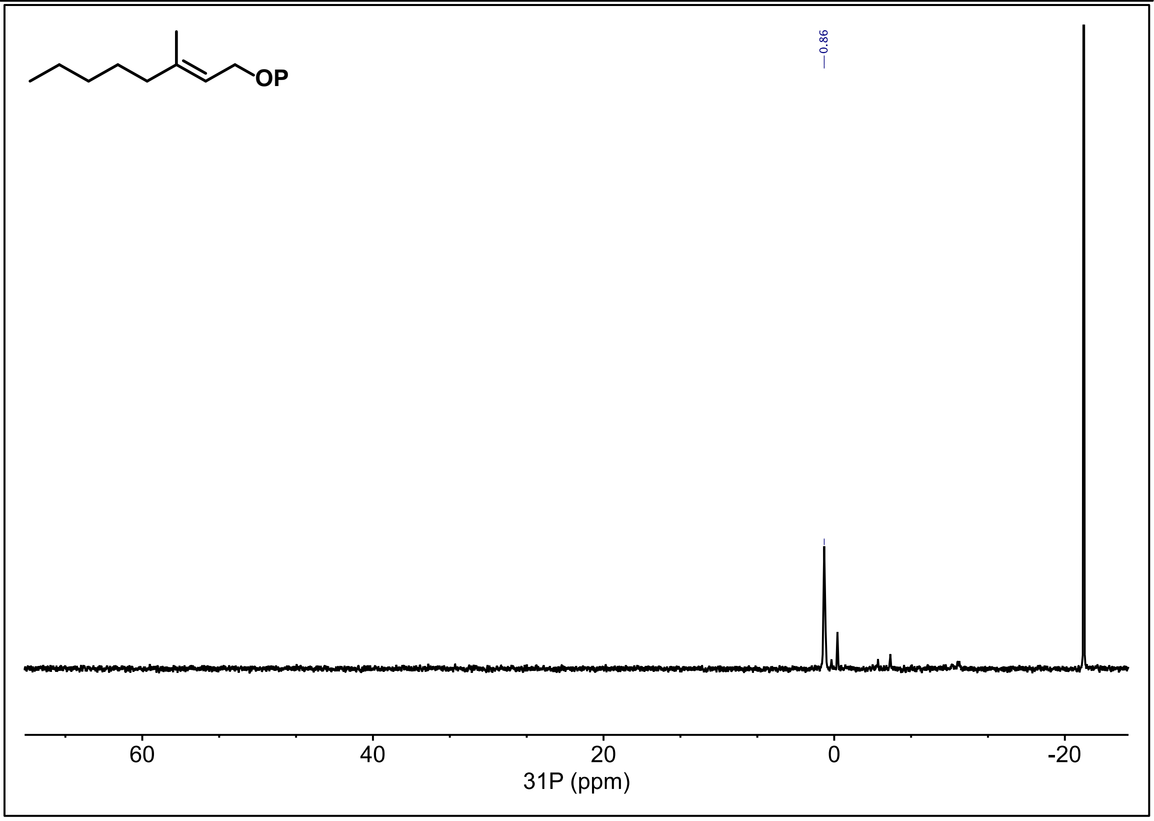

Figure S68. ^{1}H NMR (400 MHz, D_2O) and ^{31}P NMR of **34** (162 MHz, D_2O).

(3-Methyl-4-(prop-2-yn-1-yloxy)but-2-en-1-yl) Phosphate (35):
The title product was obtained as a white solid from (*E*)-3-methyl-4-(prop-2-yn-1-yloxy)but-2-en-1-ol following the procedure described in *Method 2.7b.*
TLC (^{i}PrOH: NH_4OH: H_2O 7:2:1 v/v): R_f = 0.59.
^{1}H NMR (300 MHz, D_2O): δ 5.67 (t, *J* = 6.8 Hz, 1H), 4.35 (t, *J* = 6.5 Hz, 2H), 4.16 (d, *J* = 2.4 Hz, 2H), 4.02 (s, 2H), 2.84 (t, *J* = 2.5 Hz, 1H), 1.68 (s, 3H).
^{31}P NMR (122 MHz, D_2O): δ 2.82.
HRMS-ESI: Calculated for $C_8H_{12}O_5P$ [M-H]$^-$: 219.04222; Found: 219.0421.

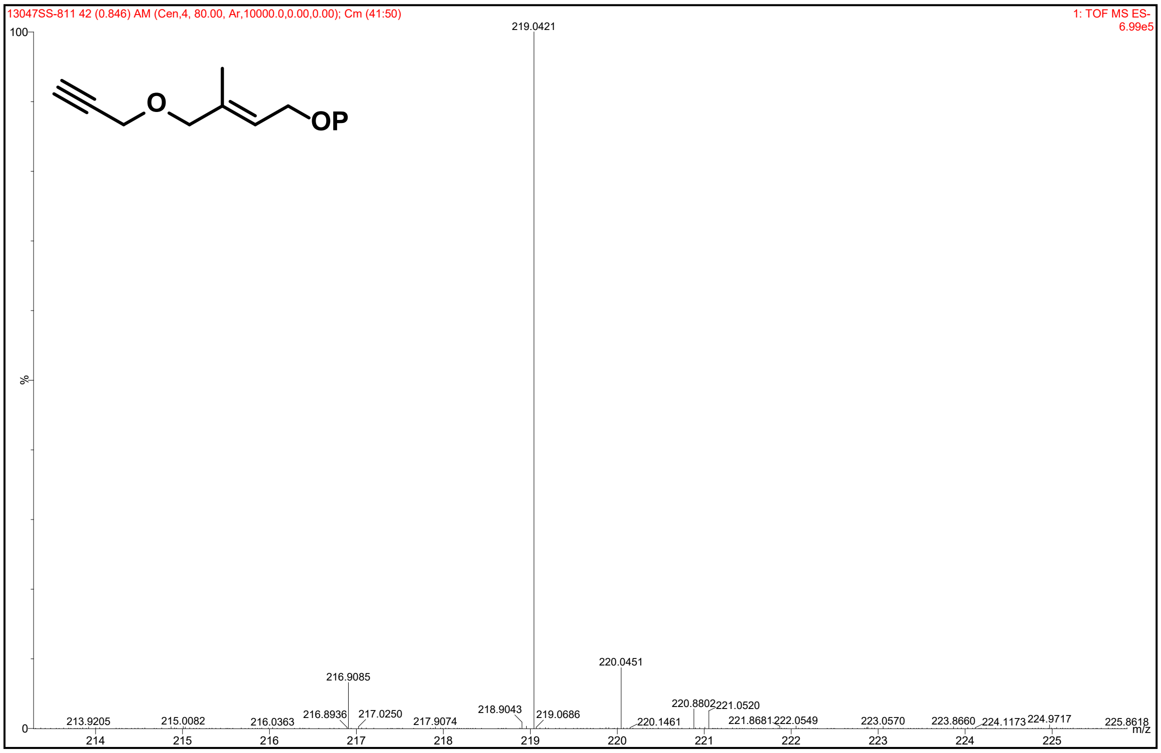

Figure S69. HRMS-ESI$^-$ of **35.**

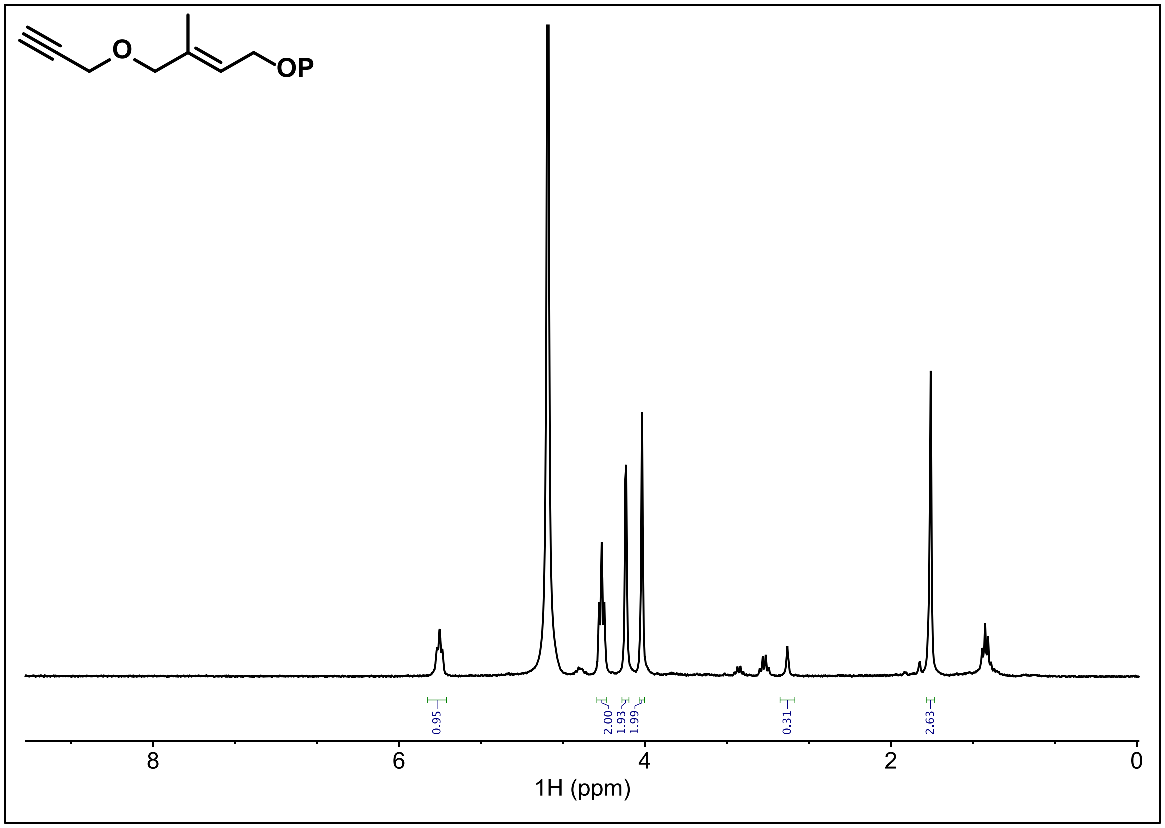

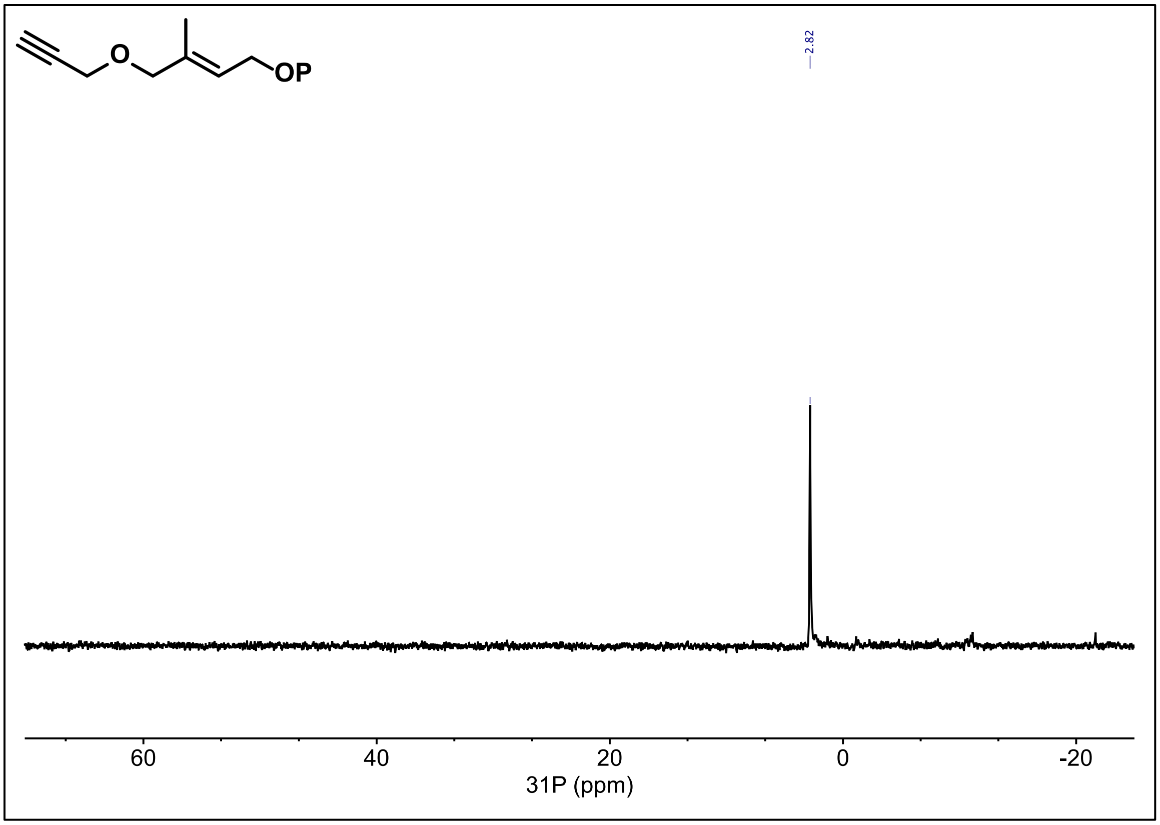

Figure S70. ^{1}H NMR (300 MHz, D_2O) and ^{31}P NMR of **35** (122 MHz, D_2O).

5-Azido-3-methylpent-2-en-1-yl Phosphate (36):

The title product was obtained as a brown solid from (*E*)-5-azido-3-methylpent-2-en-1-ol following the procedure described in *Method 2.7b.*

TLC (^{i}PrOH: NH_4OH: H_2O 7:2:1 v/v): R_f = 0.60.

^{1}H NMR (300 MHz, D_2O): δ 5.48 (t, *J* = 7.1 Hz, 1H), 4.32 (t, *J* = 6.6 Hz, 2H), 3.42 (d, *J* = 7.1 Hz, 2H), 2.32 (s, 2H), 1.69 (s, 3H).

^{31}P NMR (122 MHz, D_2O): δ 2.27.

HRMS-ESI: Calculated for $C_6H_{11}N_3O_4P$ $[M-H]^-$: 220.04871; Found: 220.0485.

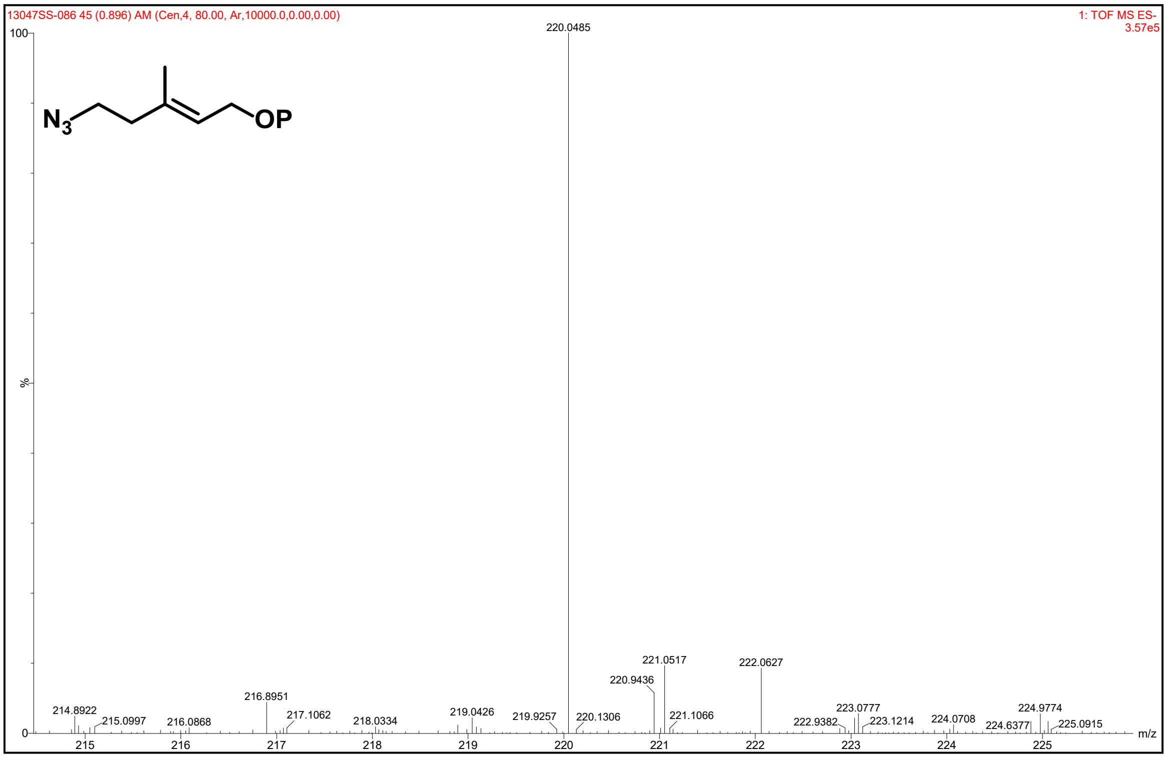

Figure S71. HRMS-ESI$^-$ of **36.**

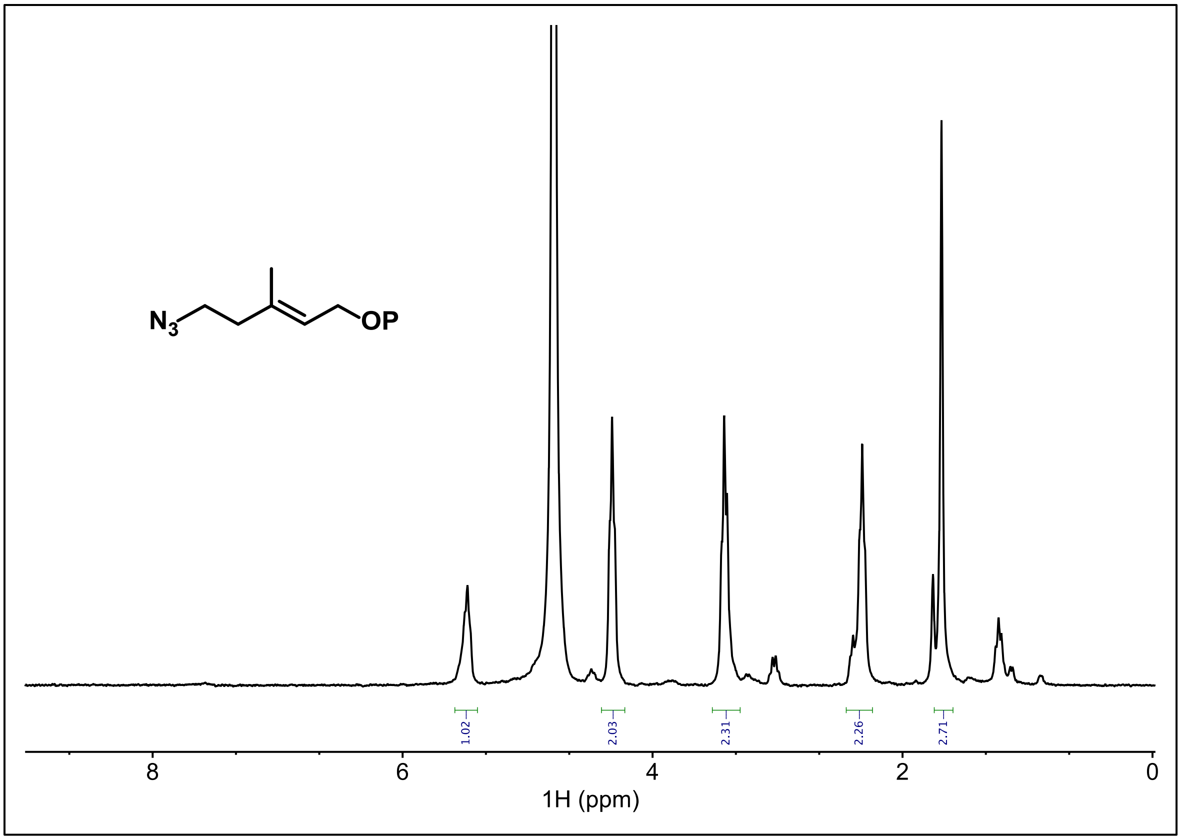

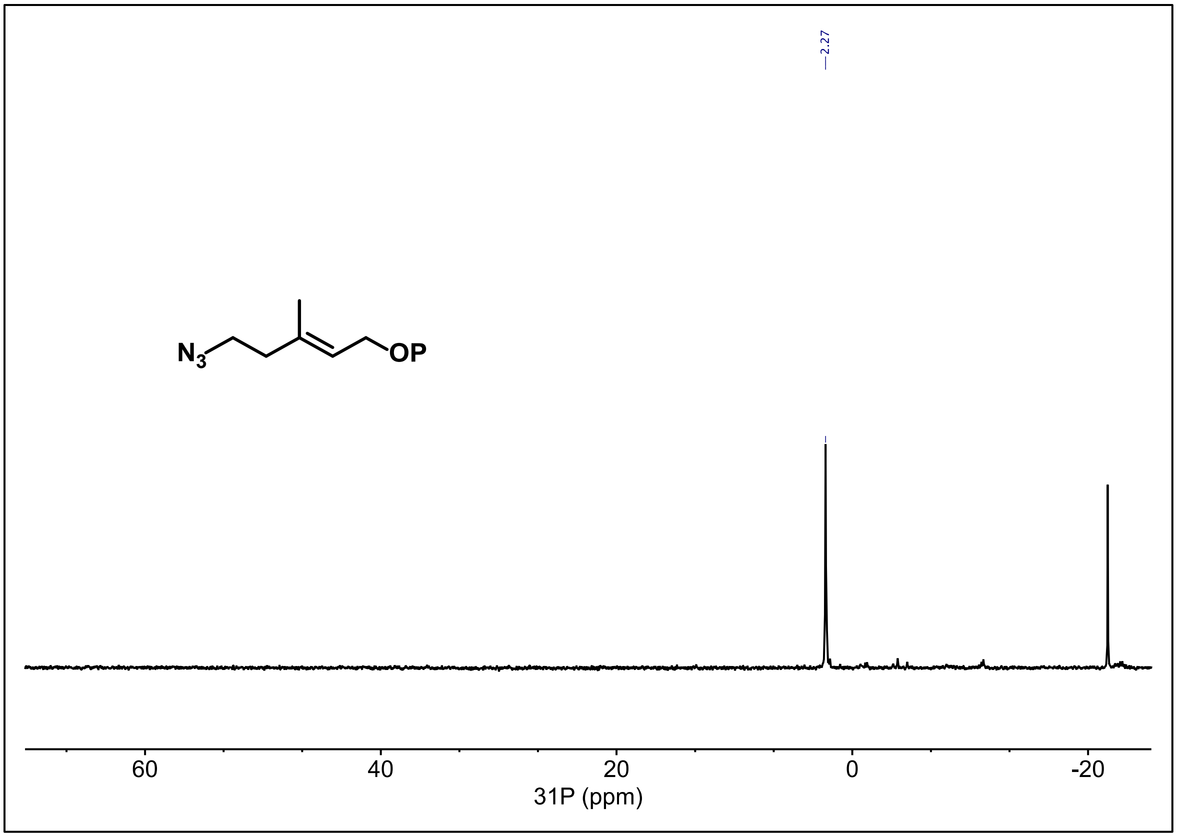

Figure S72. ^{1}H NMR (300 MHz, D_2O) and ^{31}P NMR of **36** (122 MHz, D_2O).

3,7-Dimethylocta-2,6-dien-1-yl Phosphate (37):

The title product was obtained as an ivory solid from (*E*)-3,7-dimethylocta-2,6-dien-1-ol following the procedure described in *Method 2.7a.*

TLC (^{i}PrOH: NH_4OH: H_2O 7:2:1 v/v): R_f = 0.60.

^{1}H NMR (400 MHz, D_2O): δ 5.63 – 5.38 (m, 1H), 5.27 – 5.21 (m, 1H), 4.41 (t, *J* = 6.9 Hz, 2H), 2.25 – 1.96 (m, 4H), 1.72 (s, 9H).

^{31}P NMR (162 MHz, D_2O): δ 0.71.

HRMS-ESI: Calculated for $C_{10}H_{18}O_4P$ [M-H]$^-$: 233.09426; Found: 233.0945.

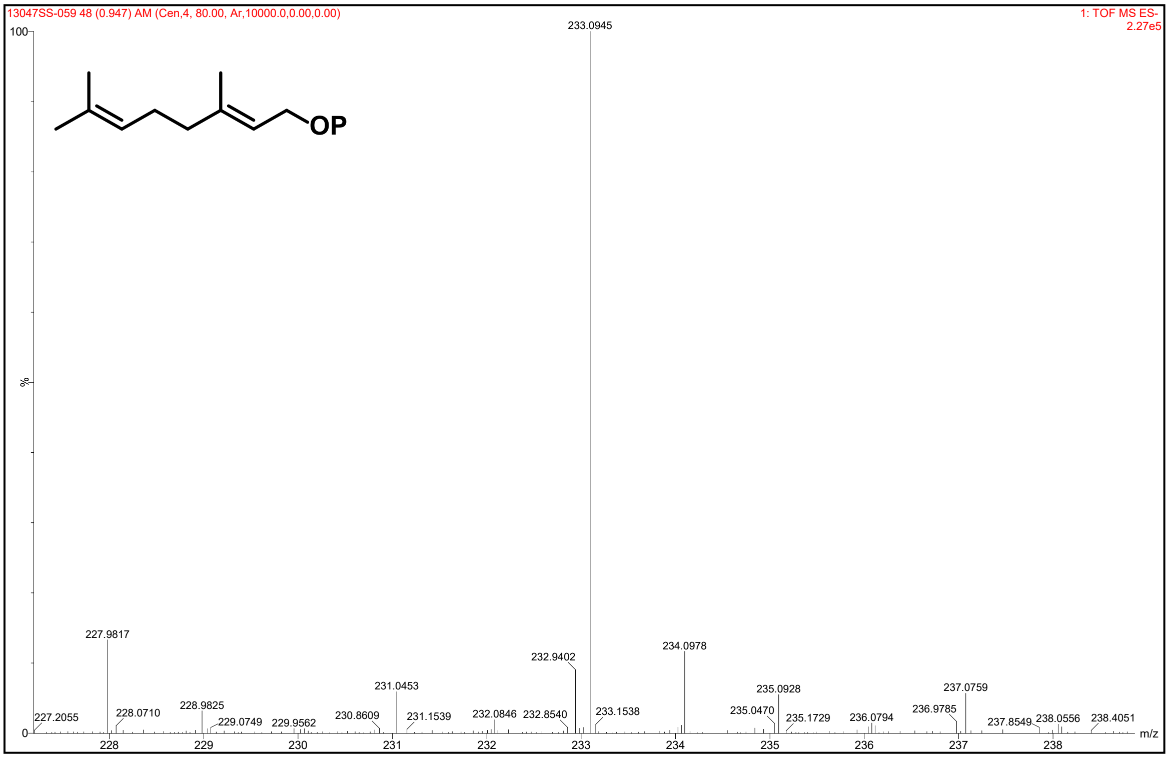

Figure S73. HRMS-ESI$^-$ of **37.**

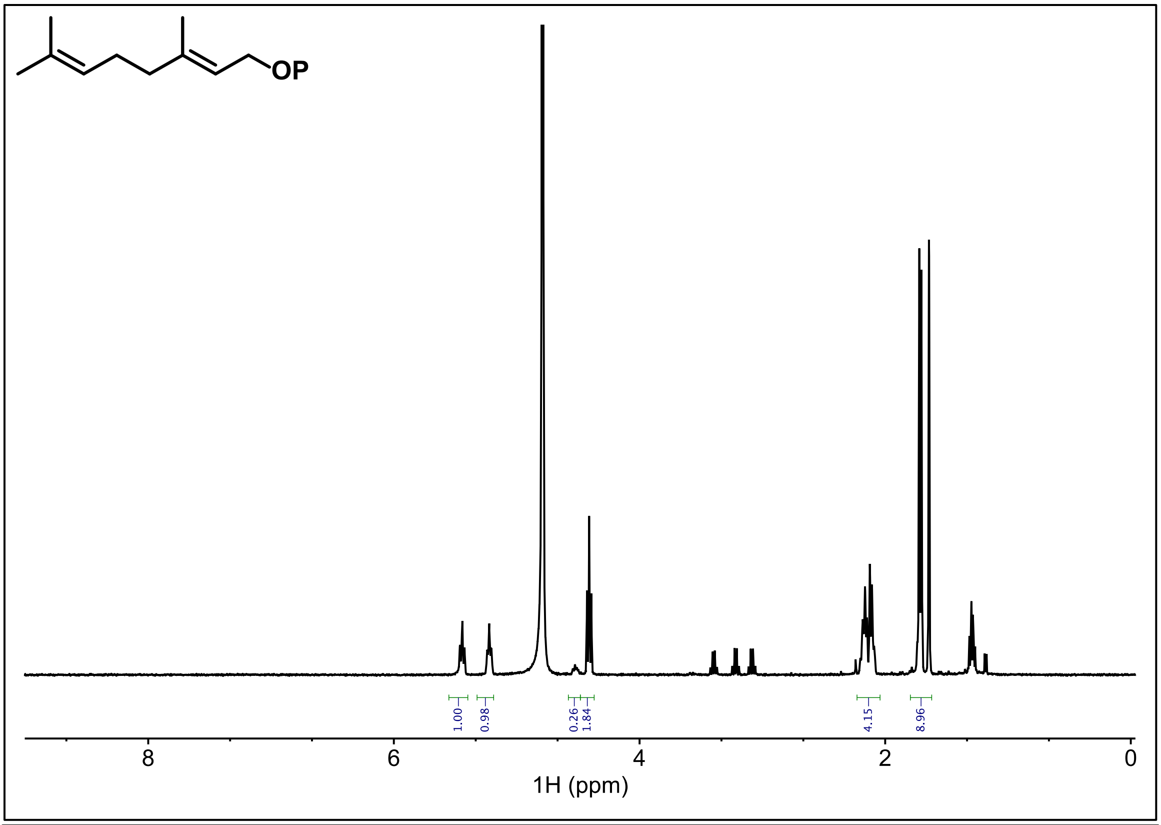

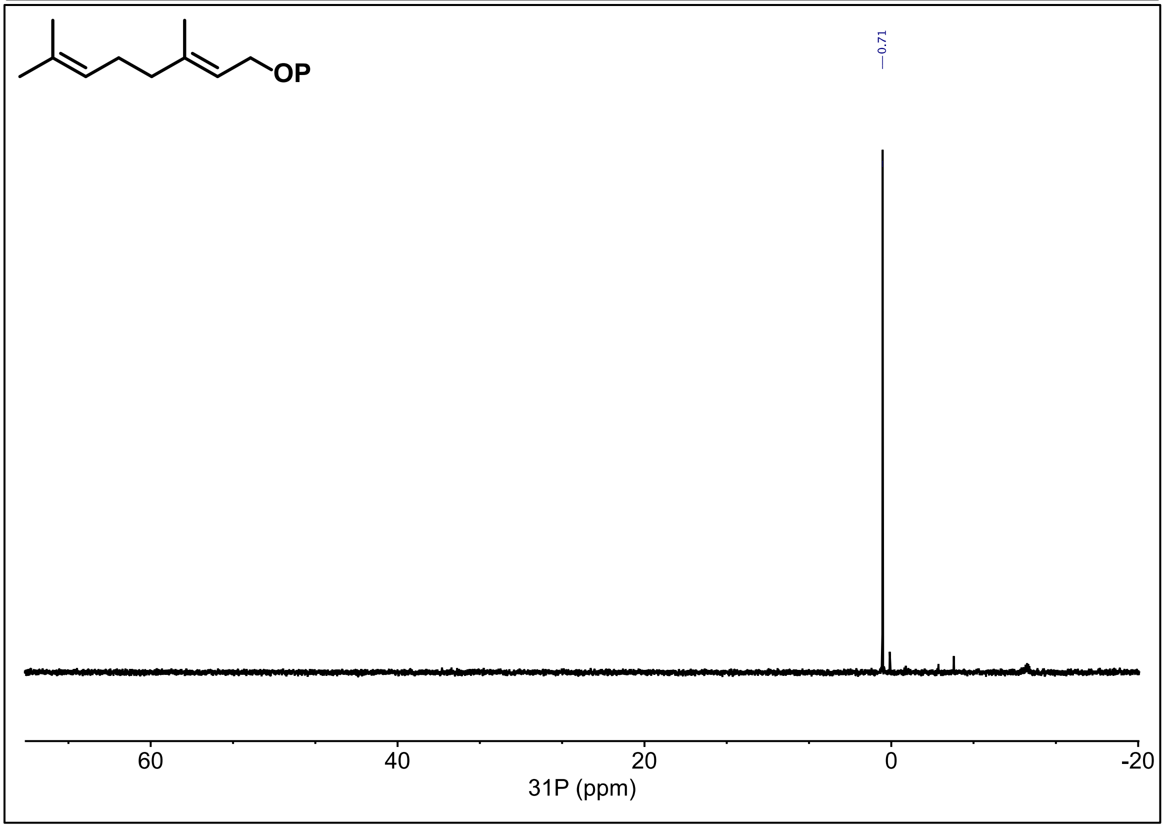

Figure S74. ^{1}H NMR (400 MHz, D_2O) and ^{31}P NMR of **37** (162 MHz, D_2O).

(3-Methyl-4-((tetrahydro-2*H*-pyran-2-yl)oxy)but-2-en-1-yl) Phosphate (38):
The title product was obtained as a brown solid from (*E*)-3-methyl-4-((tetrahydro-2*H*-pyran-2-yl)oxy)but-2-en-1-ol following the procedure described in *Method 2.7b.*
TLC (^{i}PrOH: NH_4OH: H_2O 7:2:1 v/v): R_f = 0.61.
^{1}H NMR (300 MHz, D_2O): δ 5.65 (t, *J* = 7.0 Hz, 1H), 4.76 – 4.62 (m, 1H), 4.35 (d, *J* = 6.9 Hz, 2H), 4.22 – 4.09 (m, 1H), 3.98 (d, *J* = 12.0 Hz, 1H), 3.85 (d, *J* = 7.8 Hz, 1H), 3.56 (dd, *J* = 11.5, 6.6 Hz, 1H), 2.10 – 1.62 (m, 5H), 1.52 (d, *J* = 6.9 Hz, 4H).
^{31}P NMR (122 MHz, D_2O): δ 2.47.
HRMS-ESI: Calculated for $C_{10}H_{18}O_6P$ $[M-H]^-$: 265.08409; Found: 265.0839.

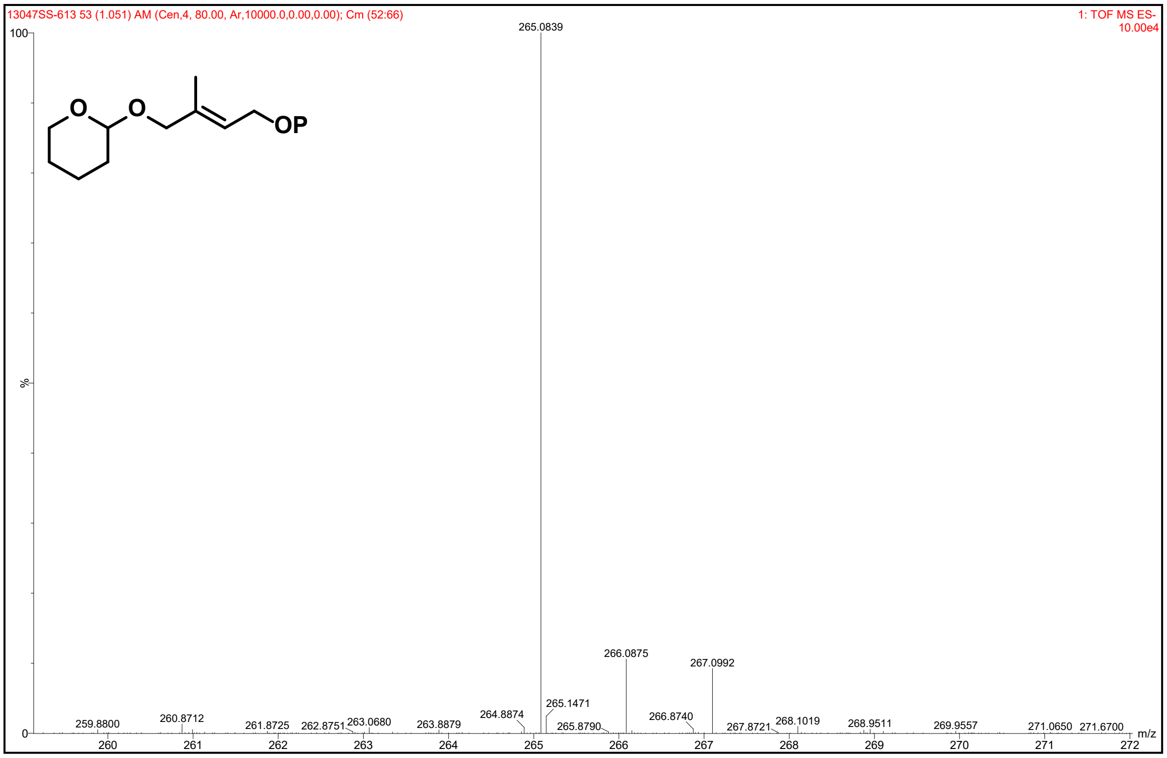

Figure S75. HRMS-ESI$^-$ of **38.**

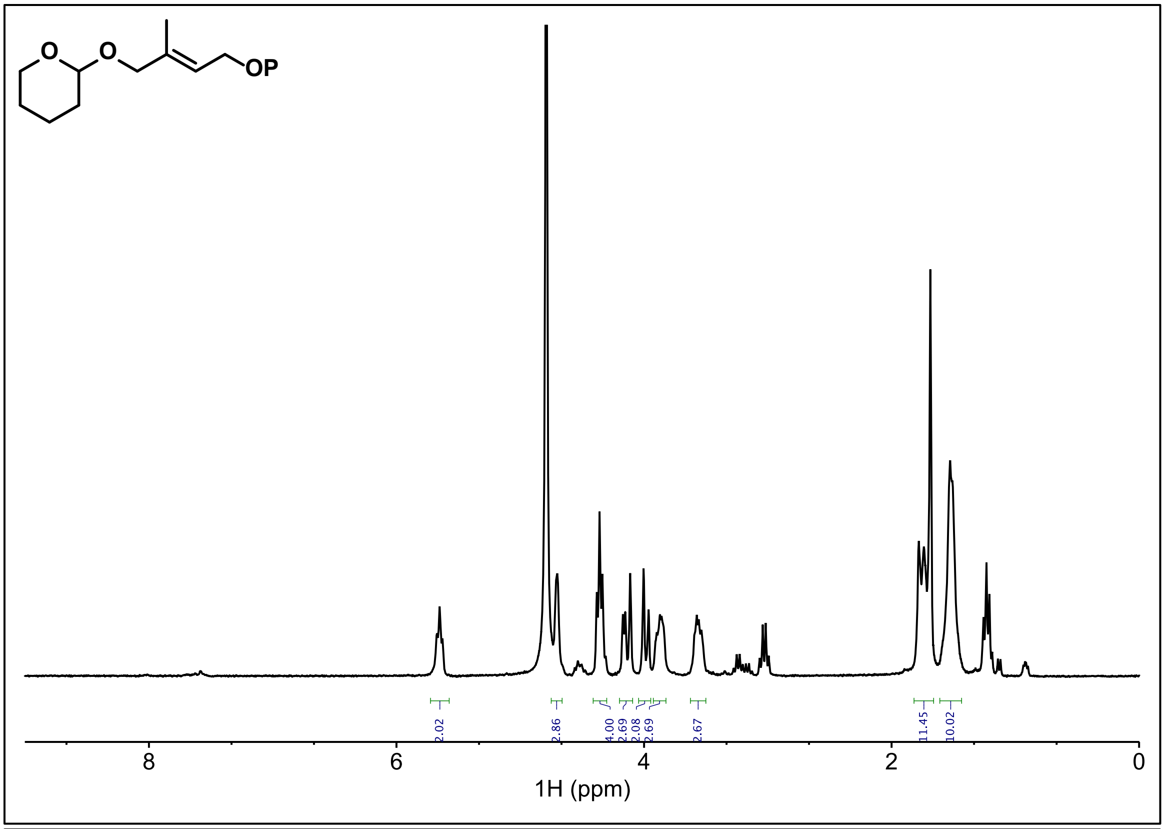

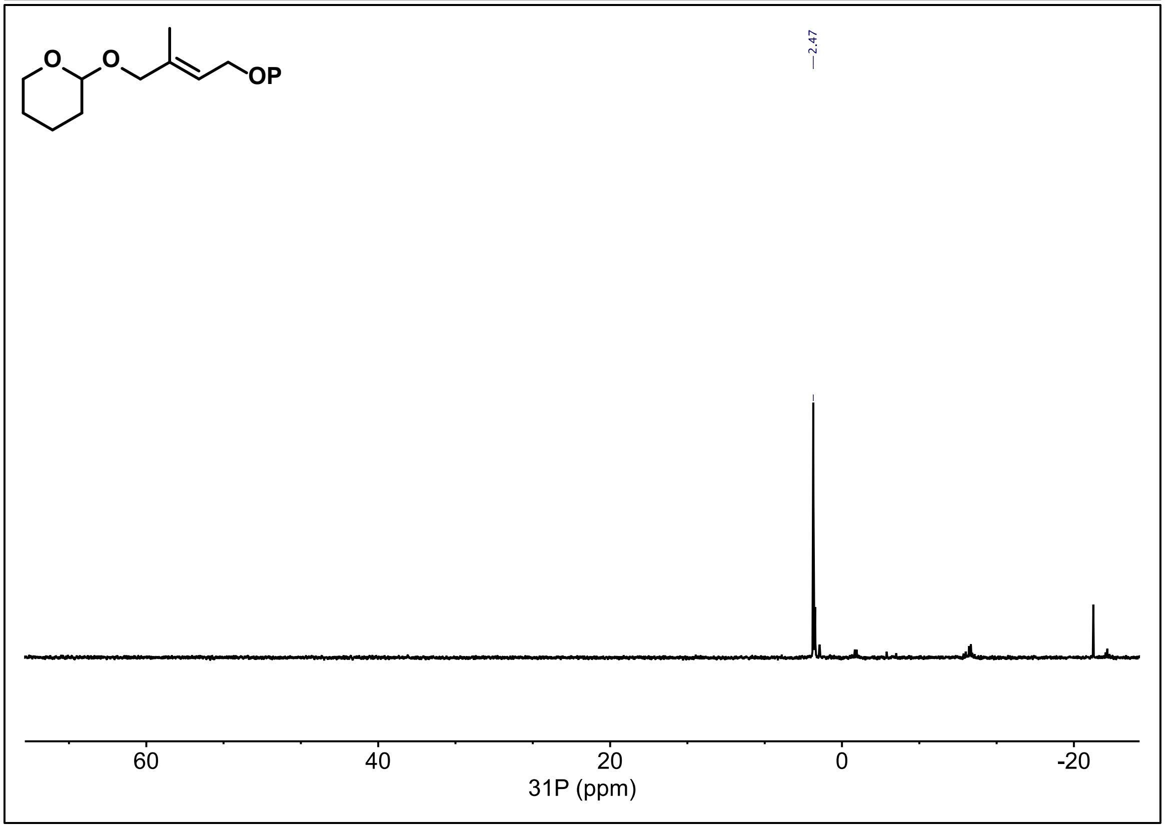

Figure S76. ^{1}H NMR (300 MHz, D_2O) and ^{31}P NMR of **38** (122 MHz, D_2O).

(2*E*,4*E*)-5-Phenylpenta-2,4-dien-1-yl Phosphate (39):

The title product was obtained as a brown-white solid from (2*E*,4*E*)-5-phenylpenta-2,4-dien-1-ol following the procedure described in *Method 2.7a.*

TLC (^{i}PrOH: NH_4OH: H_2O 7:2:1 v/v): R_f = 0.64.

^{1}H NMR (300 MHz, D_2O): δ 7.64 – 7.17 (m, 5H), 6.93 (dd, *J* = 15.7, 10.6 Hz, 1H), 6.65 (d, *J* = 15.7 Hz, 1H), 6.58 – 6.44 (m, 1H), 5.97 (dt, *J* = 15.2, 6.1 Hz, 1H), 4.39 (d, *J* = 6.4 Hz, 2H).

^{31}P NMR (122 MHz, D_2O): δ 1.80.

HRMS-ESI: Calculated for $C_{11}H_{12}O_4P$ $[M-H]^-$: 239.04731; Found: 239.0476.

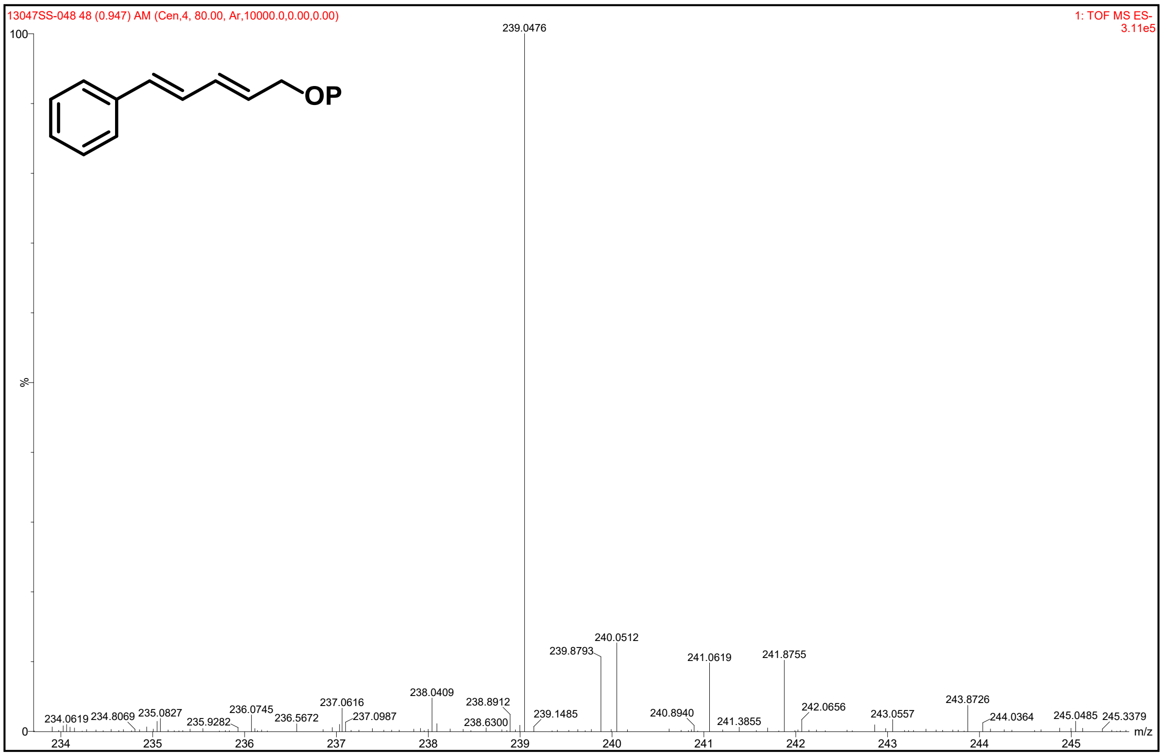

Figure S77. HRMS-ESI$^-$ of **39.**

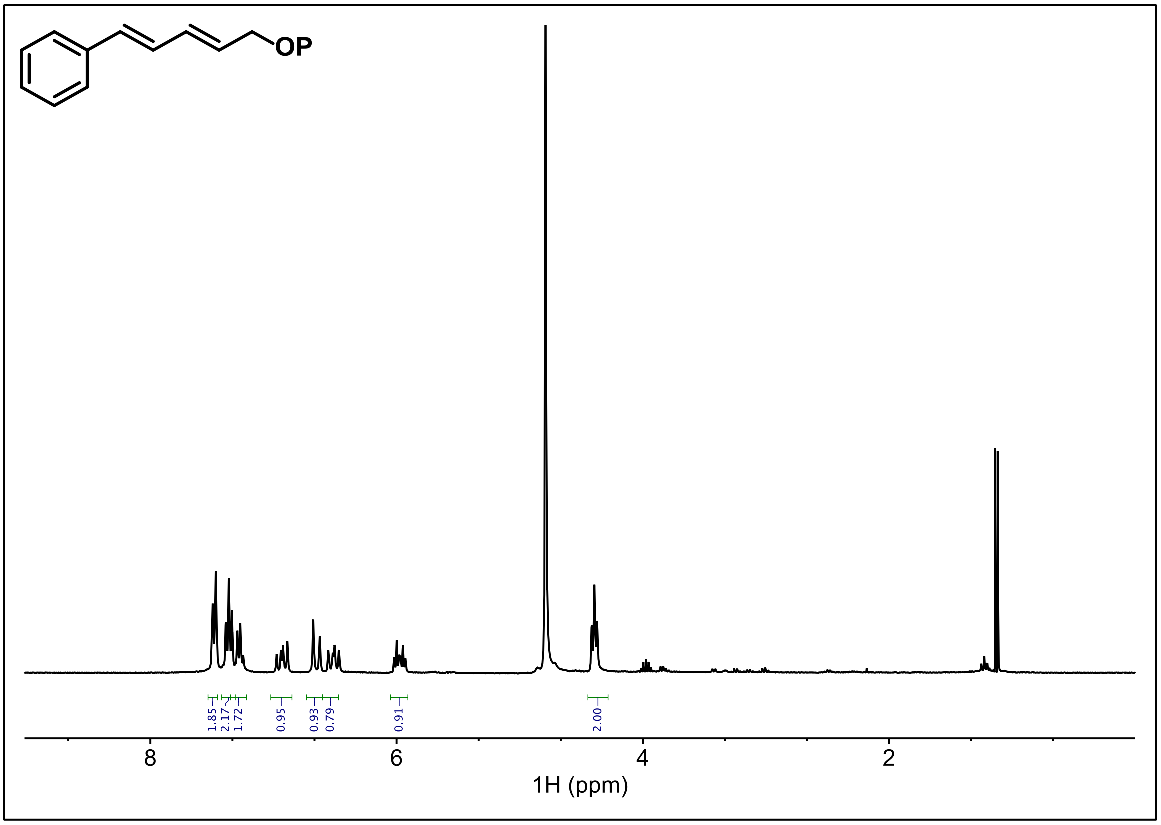

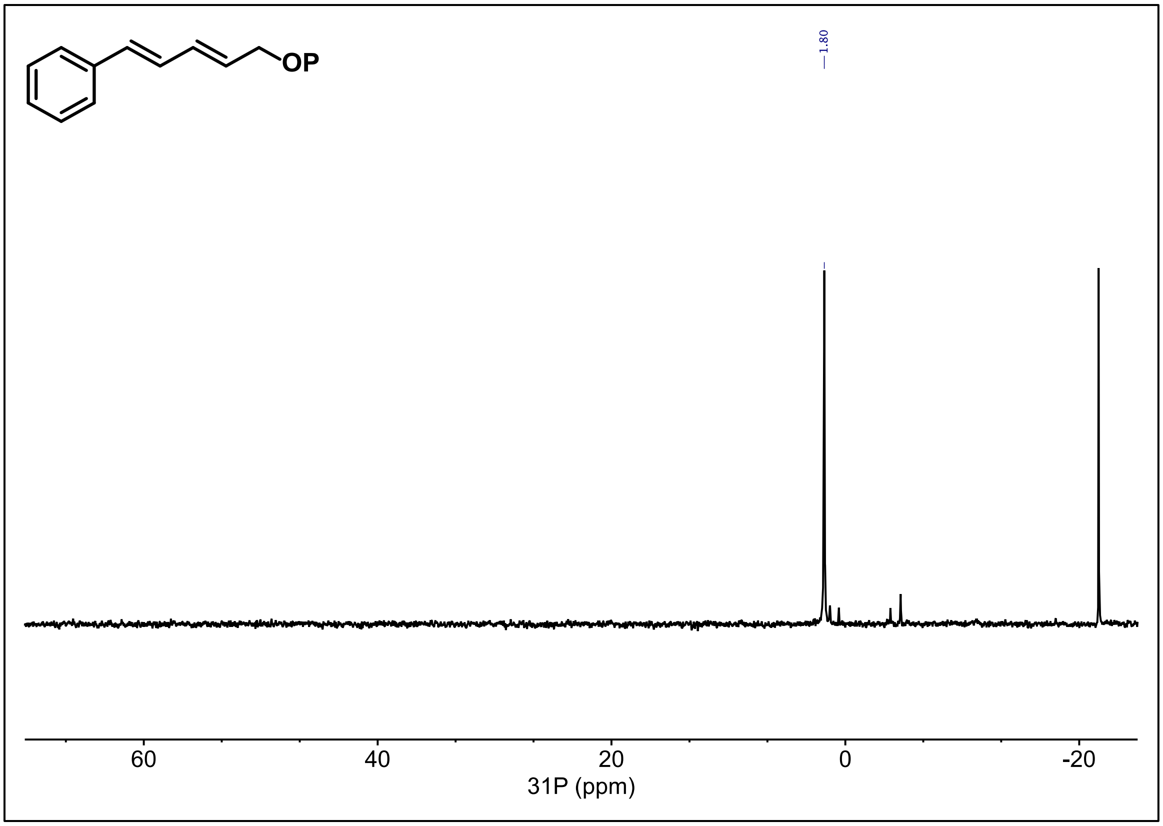

Figure S78. ^{1}H NMR (300 MHz, D_2O) and ^{31}P NMR of **39** (122 MHz, D_2O).

(2*E*,6*E*)-3,7-Dimethylnona-2,6-dien-1-yl Phosphate (40):

The title product was obtained as a yellow-white solid from (2*E*,6*E*)-3,7-dimethylnona-2,6-dien-1-ol following the procedure described in *Method 2.7b.*

TLC (^{i}PrOH:NH_4OH:H_2O 7:2:1 v/v): R_f = 0.60.

^{1}H NMR (400 MHz, D_2O): δ 5.30 (t, *J* = 7.1 Hz, 1H), 5.18 – 5.03 (m, 1H), 4.27 (t, *J* = 6.9 Hz, 2H), 2.14 – 1.93 (m, 4H), 1.87 (d, *J* = 7.5 Hz, 2H), 1.58 (s, 3H), 1.50 (s, 3H), 0.84 (t, *J* = 7.5 Hz, 3H).

^{31}P NMR (162 MHz, D_2O): δ 0.57.

MS-ESI: Calc for $C_{11}H_{20}O_4P$ $[M-H]^-$: 247.10991; Found: 247.1058.

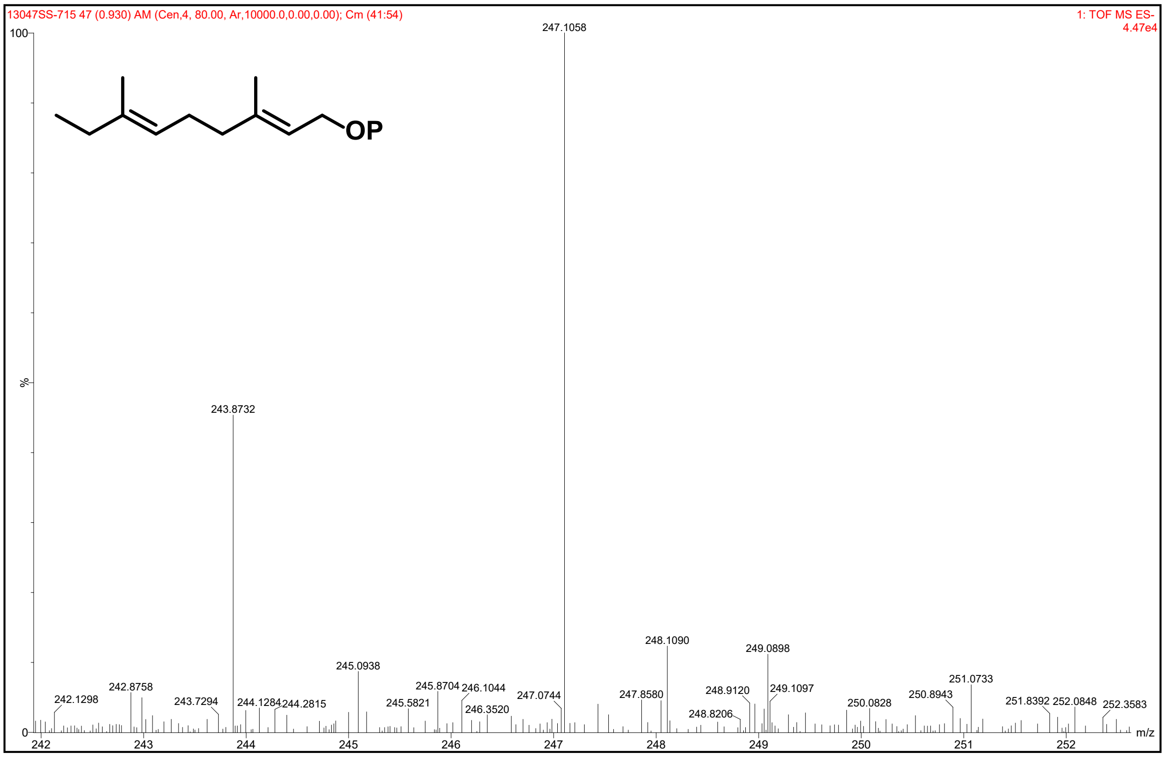

Figure S79. MS-ESI$^-$ of **40.**

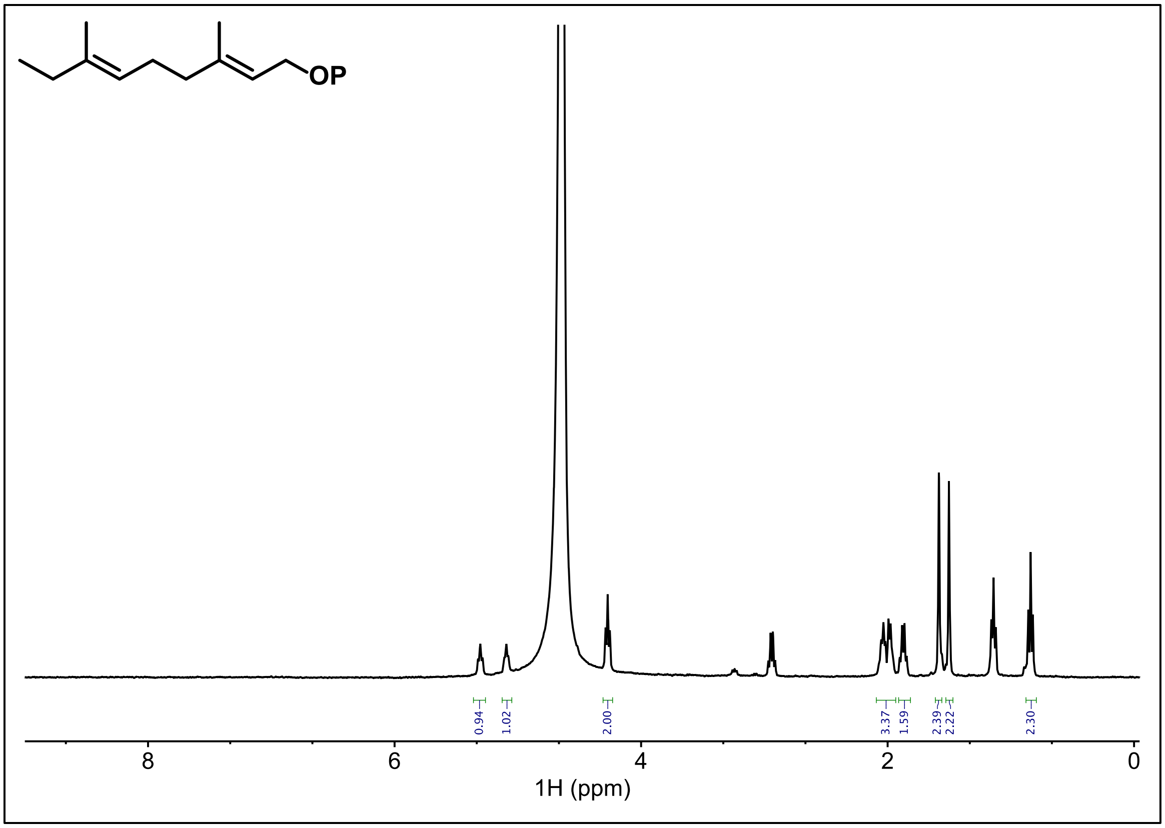

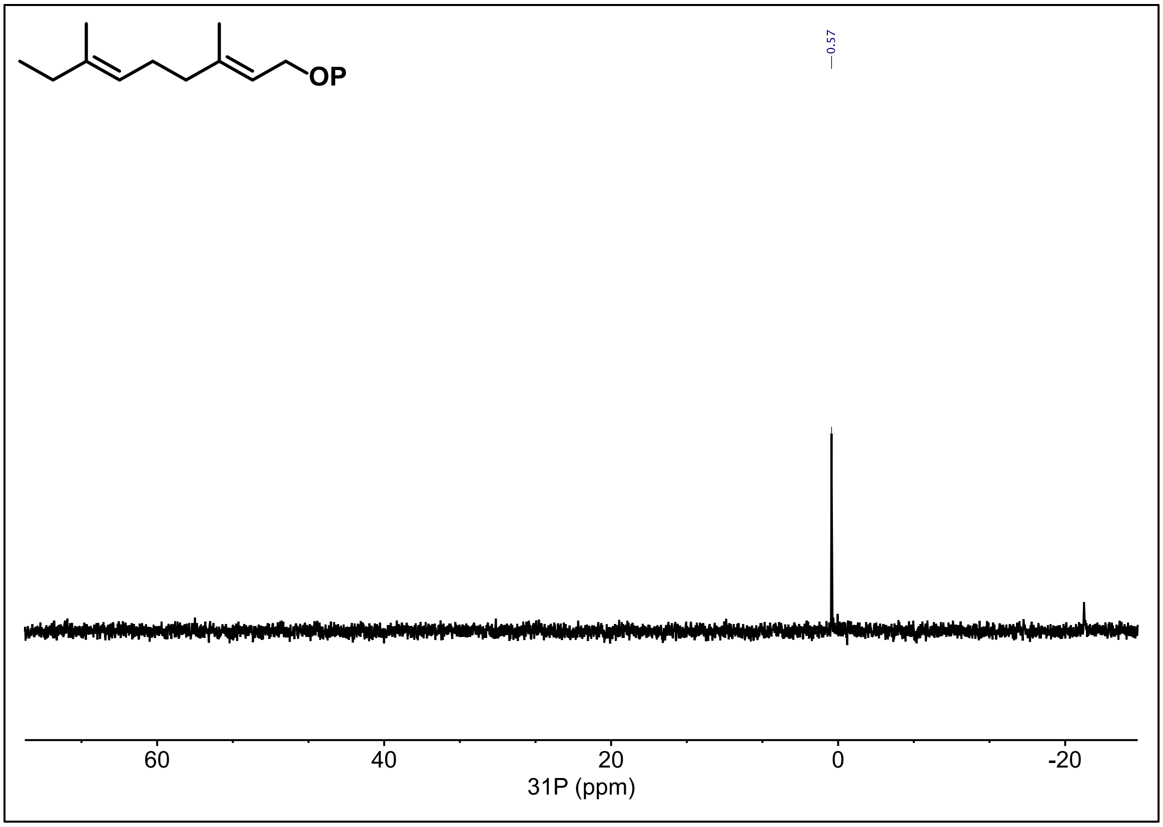

Figure S80. ^{1}H NMR (400 MHz, D_2O) and ^{31}P NMR of **40** (162 MHz, D_2O).

(2*E*,6*E*)-8-Hydroxy-3,7-dimethylocta-2,6-dien-1-yl Phosphate (41):
The title product was obtained as a brown-white solid from (2*E*,6*E*)-8-((*tert*-butyldimethylsilyl)oxy)-3,7-dimethylocta-2,6-dien-1-ol following the procedure described in *Method 2.7b.*
TLC (^{i}PrOH:NH_4OH:H_2O 7:2:1 v/v): R_f = 0.64.
^{1}H NMR (300 MHz, D_2O): δ 5.39 (d, *J* = 7.8 Hz, 2H), 4.36 (t, *J* = 7.0 Hz, 2H), 3.92 (s, 2H), 2.32 – 1.97 (m, 4H), 1.64 (d, *J* = 19.5 Hz, 6H).
^{31}P NMR (122 MHz, D_2O): δ 0.43.
HRMS-ESI: Calculated for $C_{10}H_{18}O_5P$ [M-H]$^-$: 249.08917; Found: 249.0893.

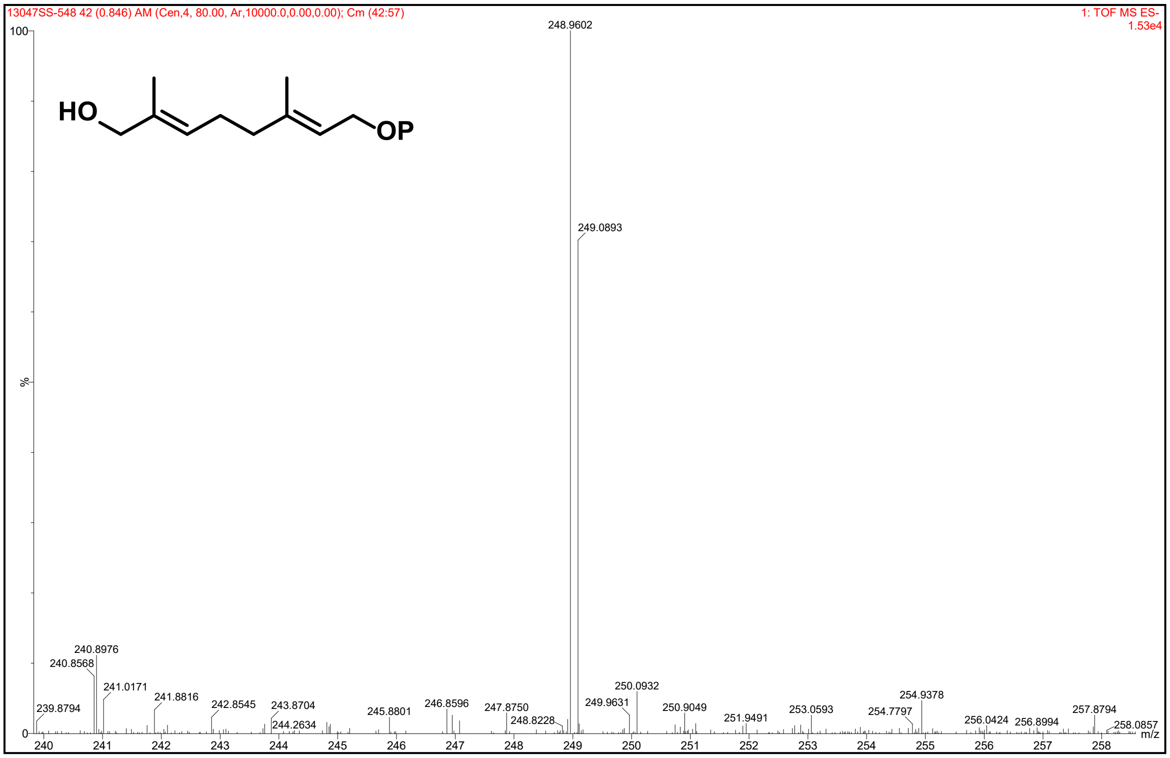

Figure S81. HRMS-ESI$^-$ of **41.**

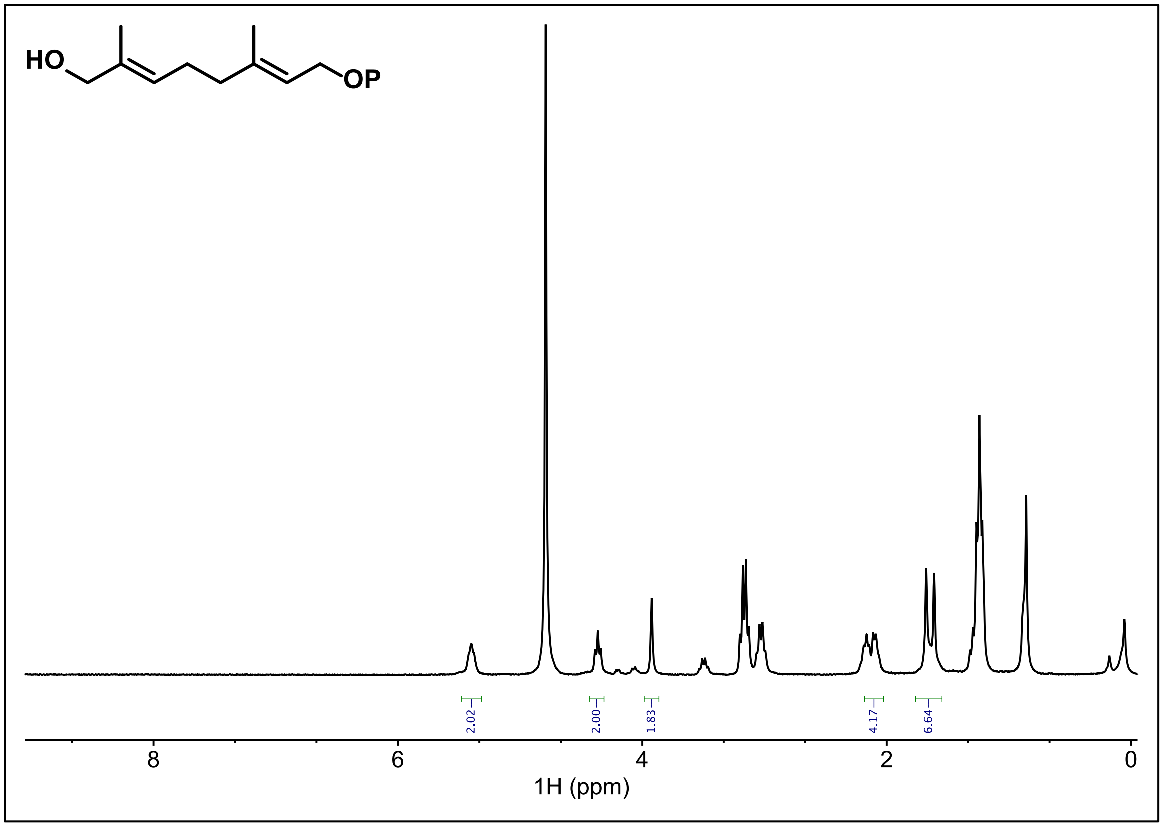

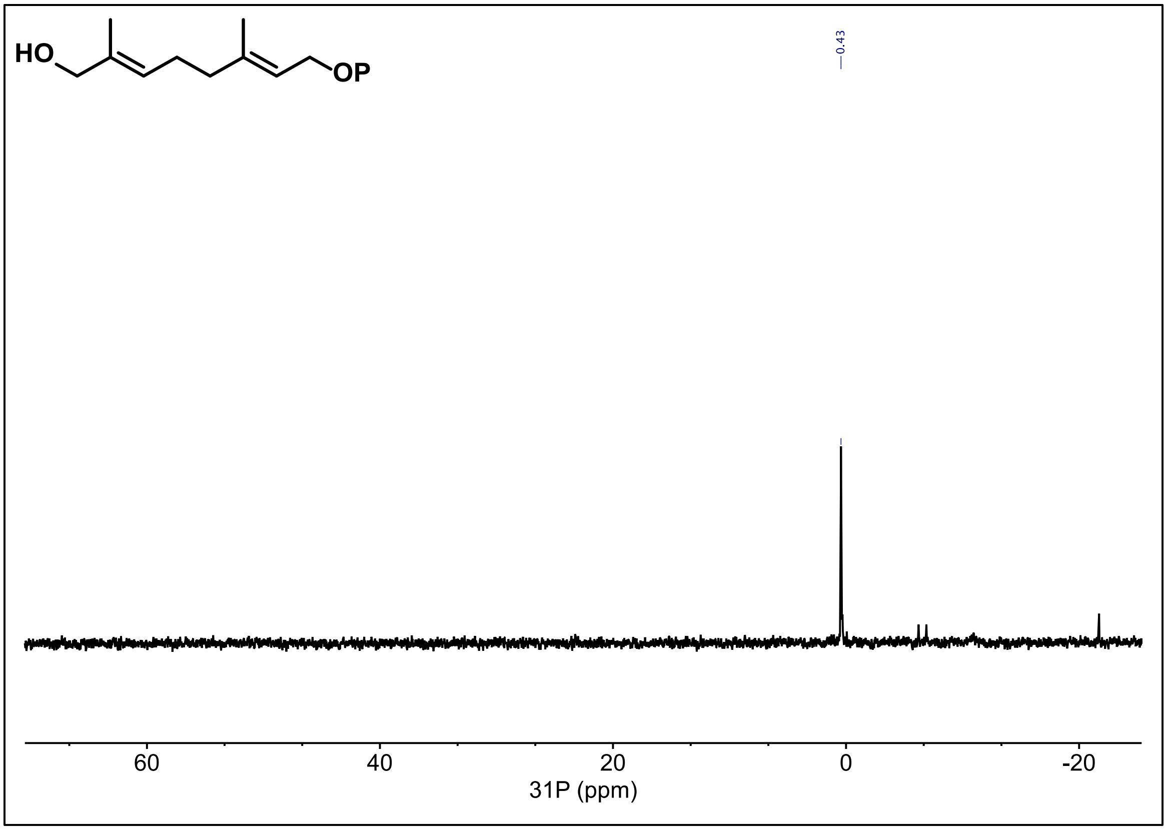

Figure S82. ^{1}H NMR (300 MHz, D_2O) and ^{31}P NMR of **41** (122 MHz, D_2O).

(2*E*,6*E*)-8 Oxy-2,6-dimethylocta-2,6-dien-1-yl acetate Phosphate (42):
The title product was obtained as a brown solid from (2*E*,6*E*)-8-hydroxy-2,6-dimethylocta-2,6-dien-1-yl acetate following the procedure described in *Method 2.7b.*
TLC (^{i}PrOH: NH_4OH: H_2O 7:2:1 v/v): R_f = 0.60.
^{1}H NMR (300 MHz, D_2O): δ 5.52 (d, *J* = 6.6 Hz, 1H), 5.40 (dt, *J* = 7.2, 3.8 Hz, 1H), 4.48 (s, 2H), 4.37 (t, *J* = 6.8 Hz, 2H), 2.29 – 2.16 (m, 2H), 2.16 – 2.05 (m, 5H), 1.74 – 1.59 (m, 6H).
^{31}P NMR (122 MHz, D_2O): δ 0.93.
HRMS-ESI: Calculated for $C_{12}H_{20}O_6P$ $[M-H]^-$: 291.09974; Found: 291.0995.

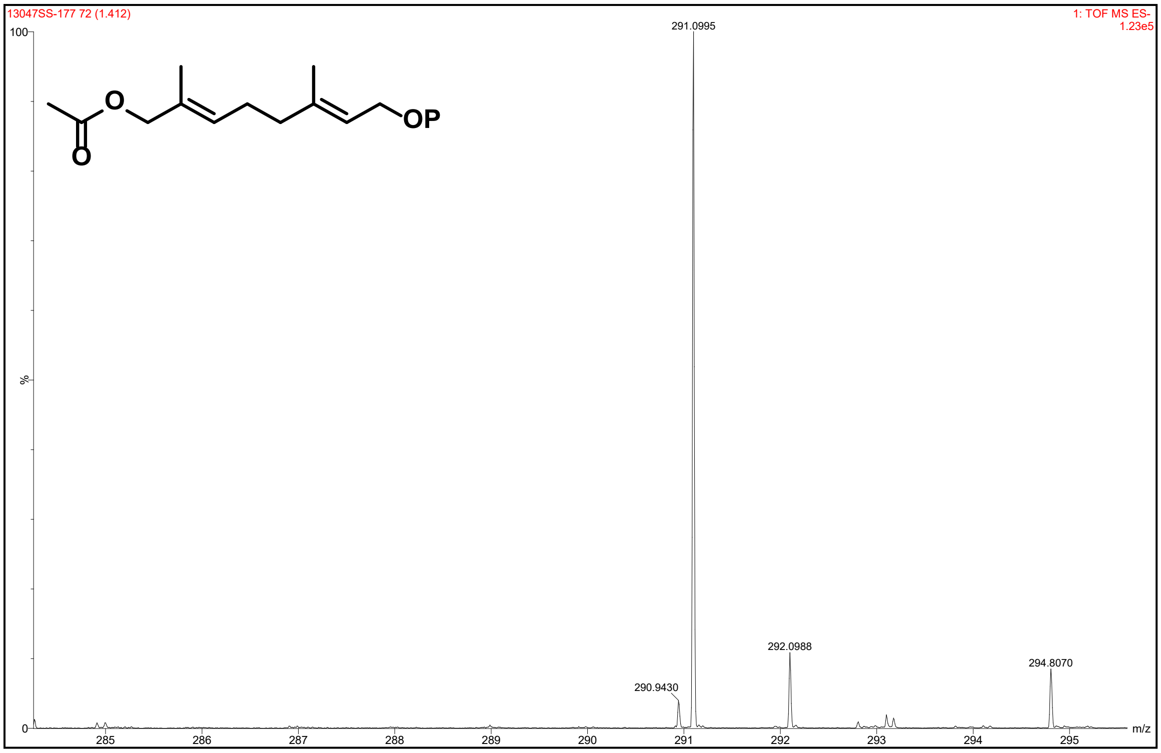

Figure S83. HRMS-ESI$^-$ of **42.**

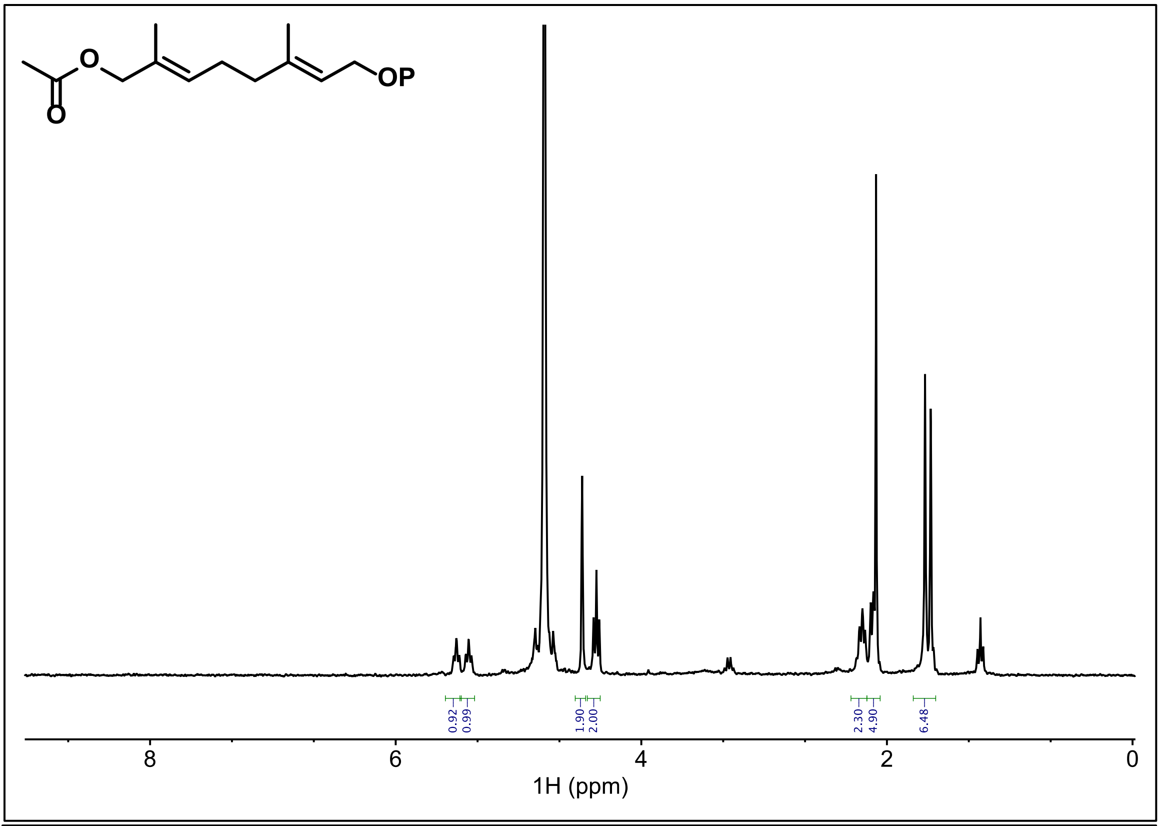

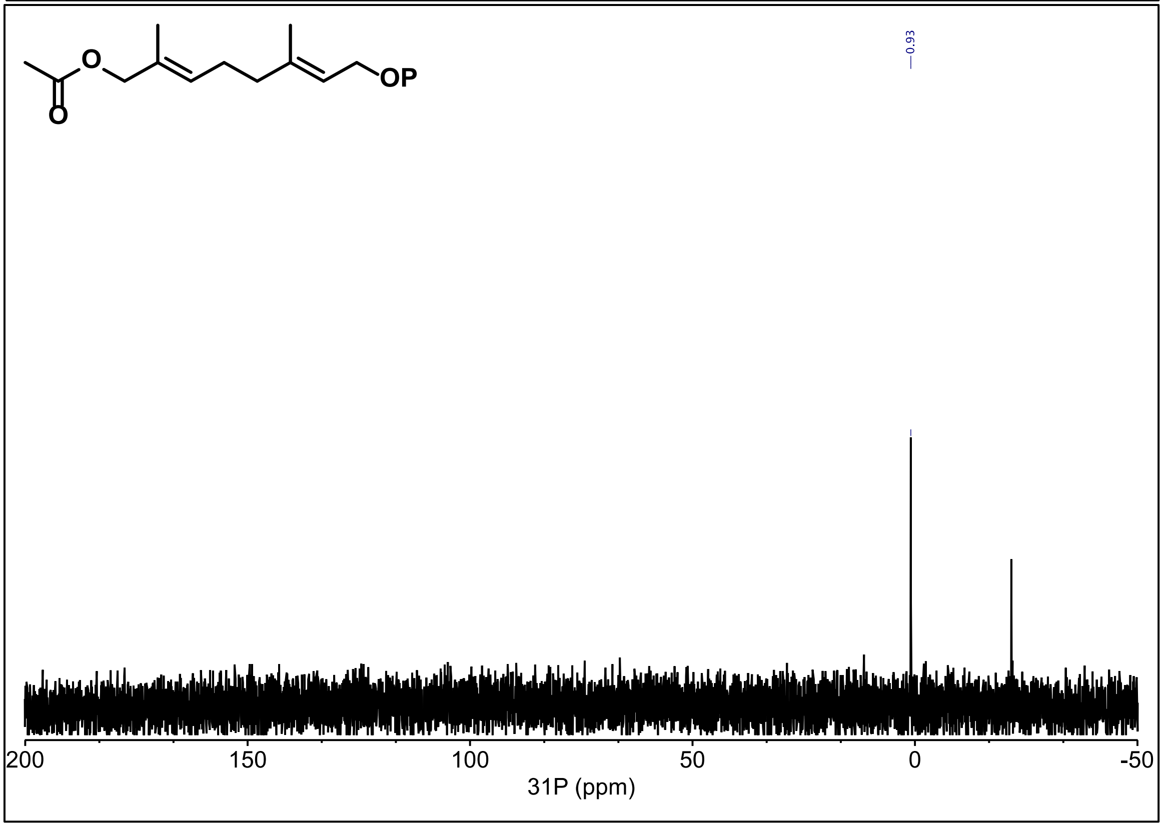

Figure S84. ^{1}H NMR (300 MHz, D_2O) and ^{31}P NMR of **42** (122 MHz, D_2O).

(2*E*,6*E*)-3,7-Dimethyl-8-(prop-2-yn-1-yloxy)octa-2,6-dien-1-yl Phosphate (43):

The title product was obtained as a white solid from (2*E*,6*E*)-3,7-dimethyl-8-(prop-2-yn-1-yloxy)octa-2,6-dien-1-ol following the procedure described in *Method 2.7b.*

TLC (^{i}PrOH: NH_4OH: H_2O 7:2:1 v/v): R_f = 0.60.

^{1}H NMR (300 MHz, D_2O): δ 5.52 (d, *J* = 6.9 Hz, 1H), 5.40 (t, *J* = 7.3 Hz, 1H), 4.32 (t, *J* = 6.6 Hz, 2H), 4.10 (s, 2H), 3.98 (s, 2H), 2.83 (s, 1H), 2.36 – 1.96 (m, 4H), 1.64 (d, *J* = 17.4 Hz, 6H).

^{31}P NMR (122 MHz, D_2O): δ 1.66.

HRMS-ESI: Calculated for $C_{13}H_{20}O_5P$ $[M-H]^-$: 287.10482; Found: 287.1062.

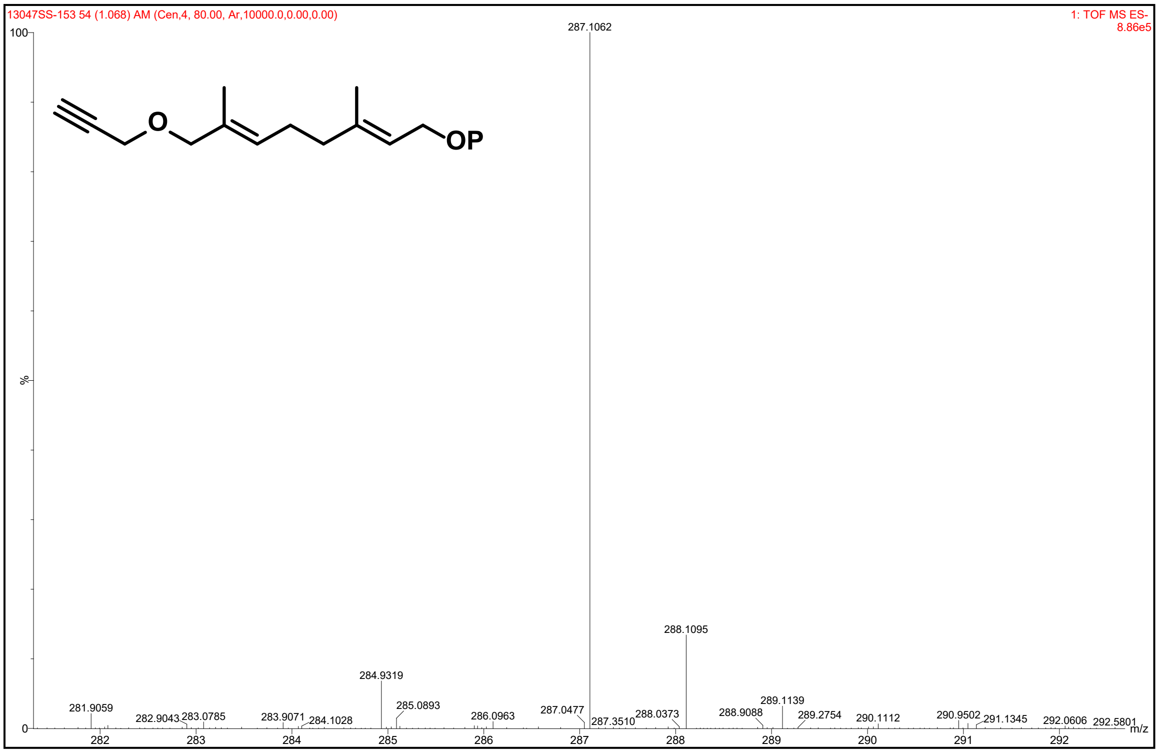

Figure S85. HRMS-ESI$^-$ of **43.**

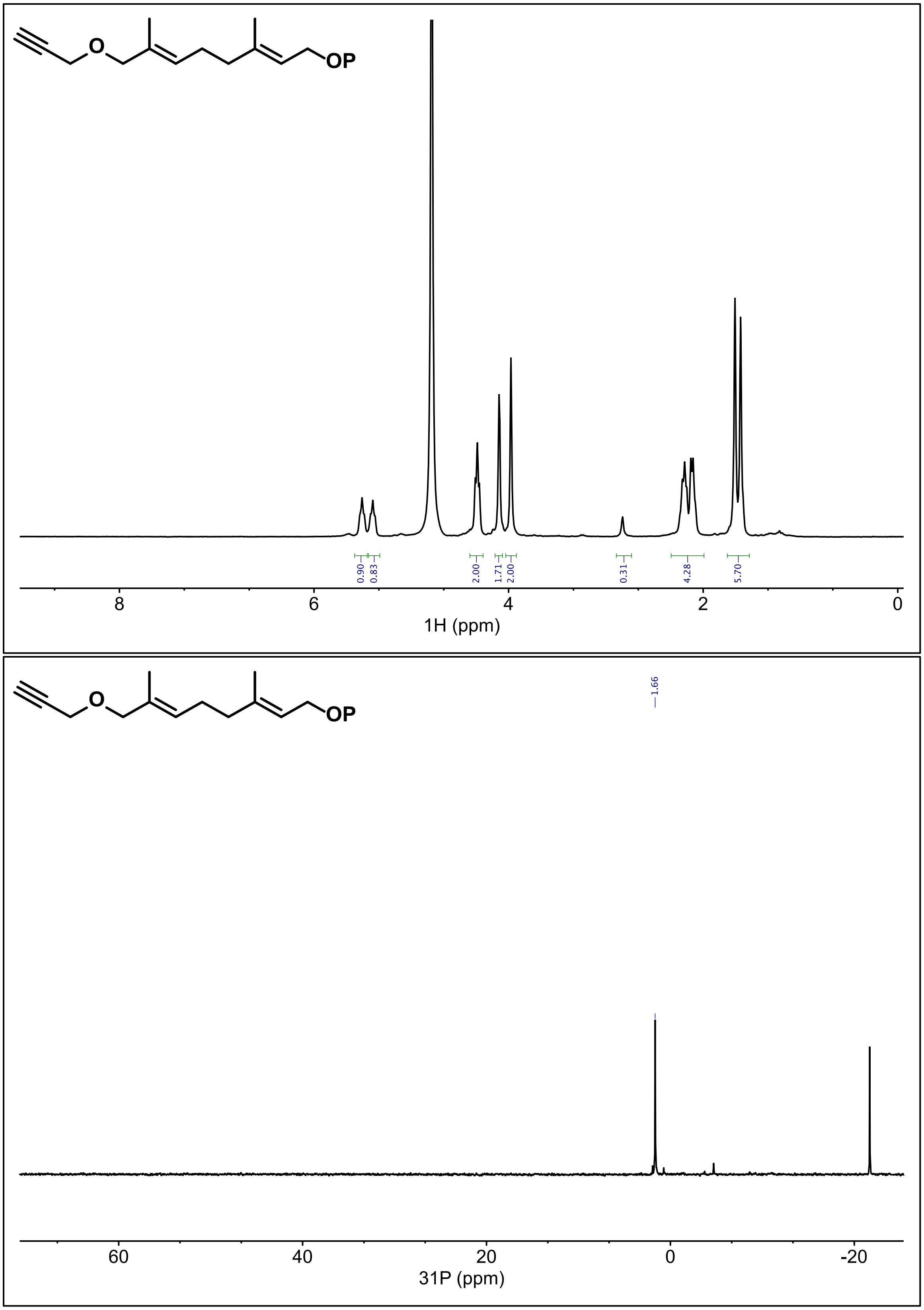

Figure S86. ^{1}H NMR (300 MHz, D_2O) and ^{31}P NMR of **43** (122 MHz, D_2O).

(1*E*,5*E*)-7-(Benzyloxy)-2,6-dimethylhepta-1,5-dien-1-yl Phosphate (45):
The title product obtained was as a light brown solid from (1*E*,5*E*)-7-(benzyloxy)-2,6-dimethylhepta-1,5-dien-1-ol following the procedure described in *Method 2.7b.*
TLC (^{i}PrOH: NH_4OH: H_2O 7:2:1 v/v): R_f = 0.61.
^{1}H NMR (300 MHz, D_2O): δ 7.48 – 7.29 (m, 5H), 5.44 (dt, *J* = 19.5, 7.0 Hz, 2H), 4.45 (s, 2H), 4.35 (t, *J* = 7.0 Hz, 2H), 3.94 (s, 2H), 2.17 (dd, *J* = 25.5, 7.2 Hz, 4H), 1.66 (d, *J* = 14.5 Hz, 6H).
^{31}P NMR (122 MHz, D_2O): δ 0.39.
HRMS-ESI: Calculated for $C_{17}H_{24}O_5P$ $[M-H]^-$: 339.13612; Found: 339.1373.

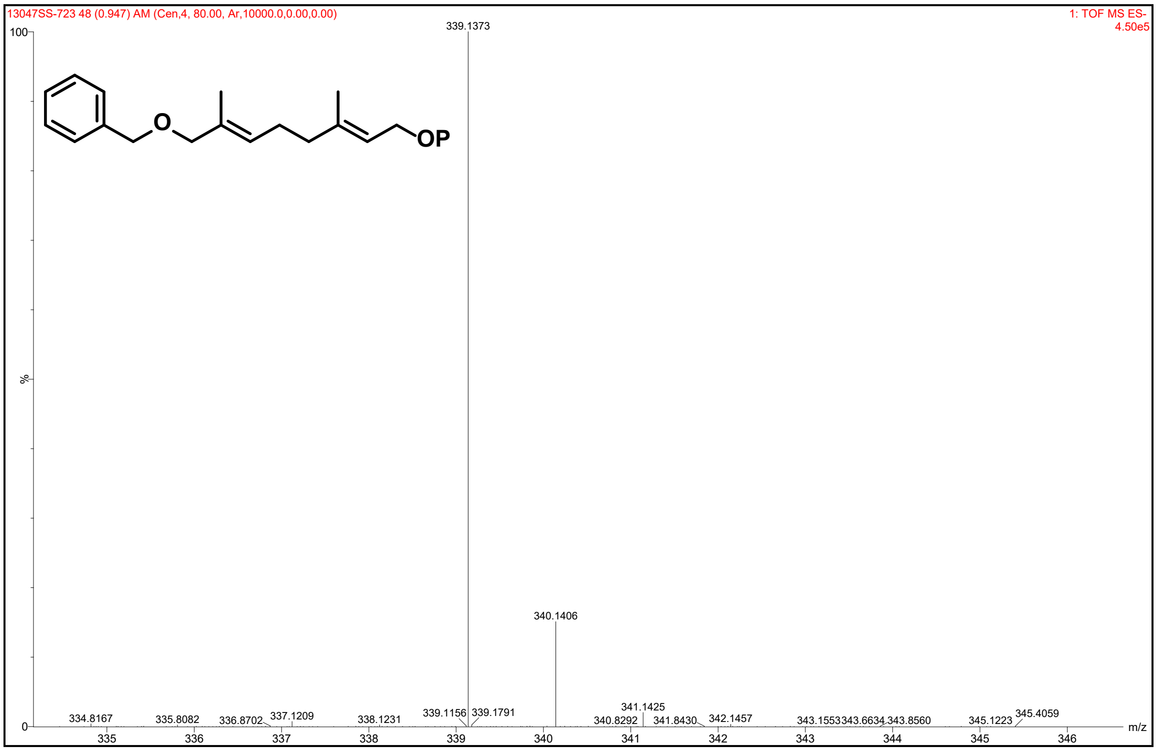

Figure S87. HRMS-ESI$^-$ of **45.**

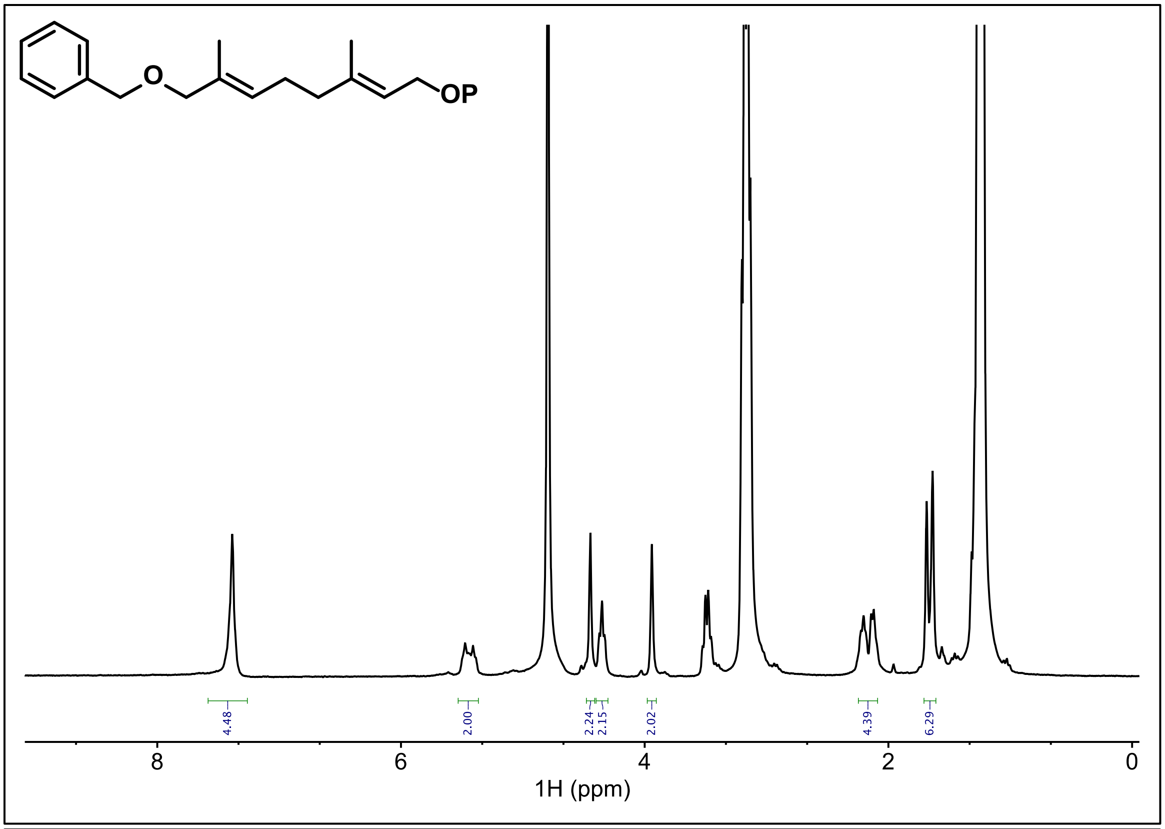

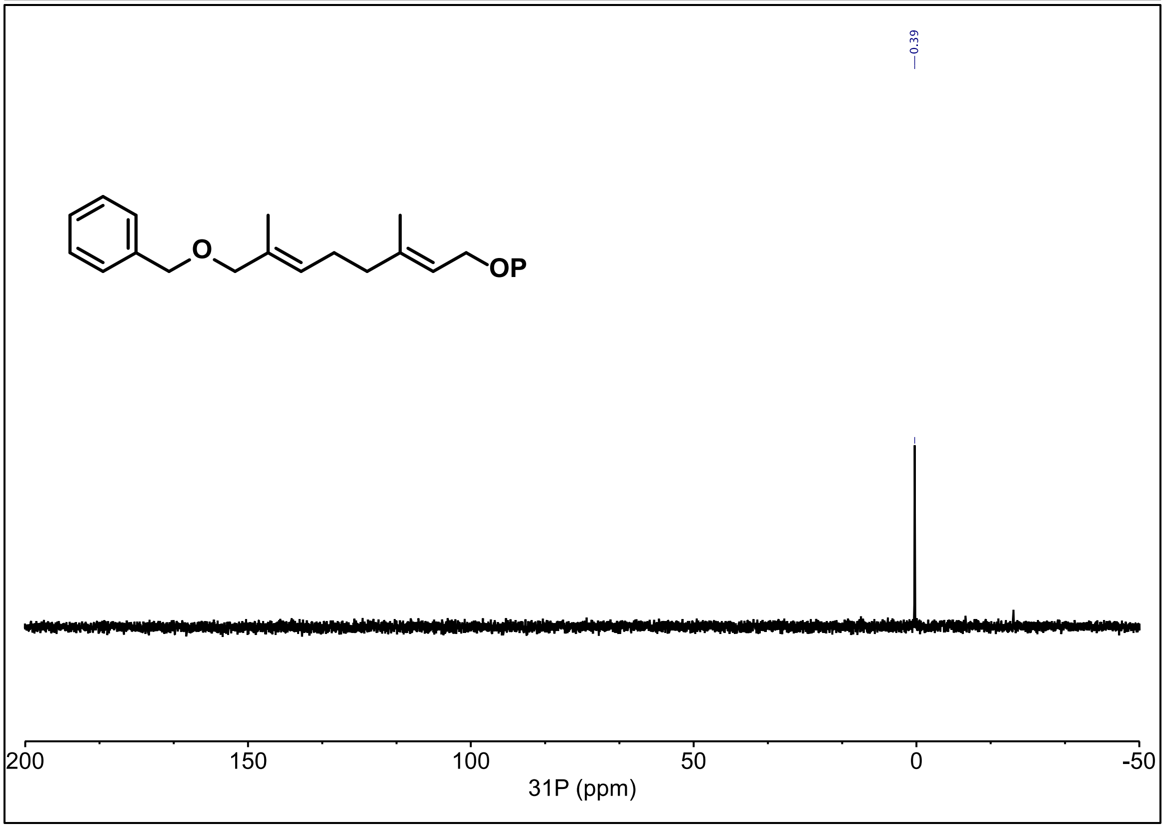

Figure S88. ^{1}H NMR (300 MHz, D_2O) and ^{31}P NMR of **45** (122 MHz, D_2O).

Benzyl Phosphate (46):

The title product was obtained as a white solid from phenylmethanol following the procedure described in *Method 2.7b.*

TLC (^{i}PrOH: NH_4OH: H_2O 7:2:1 v/v): R_f = 0.62.

^{1}H NMR (400 MHz, D_2O): δ 7.01 (d, J = 5.1 Hz, 5H), 4.56 (d, J = 7.7 Hz, 2H).

^{31}P NMR (162 MHz, D_2O): δ -0.41.

HRMS-ESI: Calculated for $C_7H_8O_4P$ [M-H]$^-$: 187.01601; Found: 187.0160.

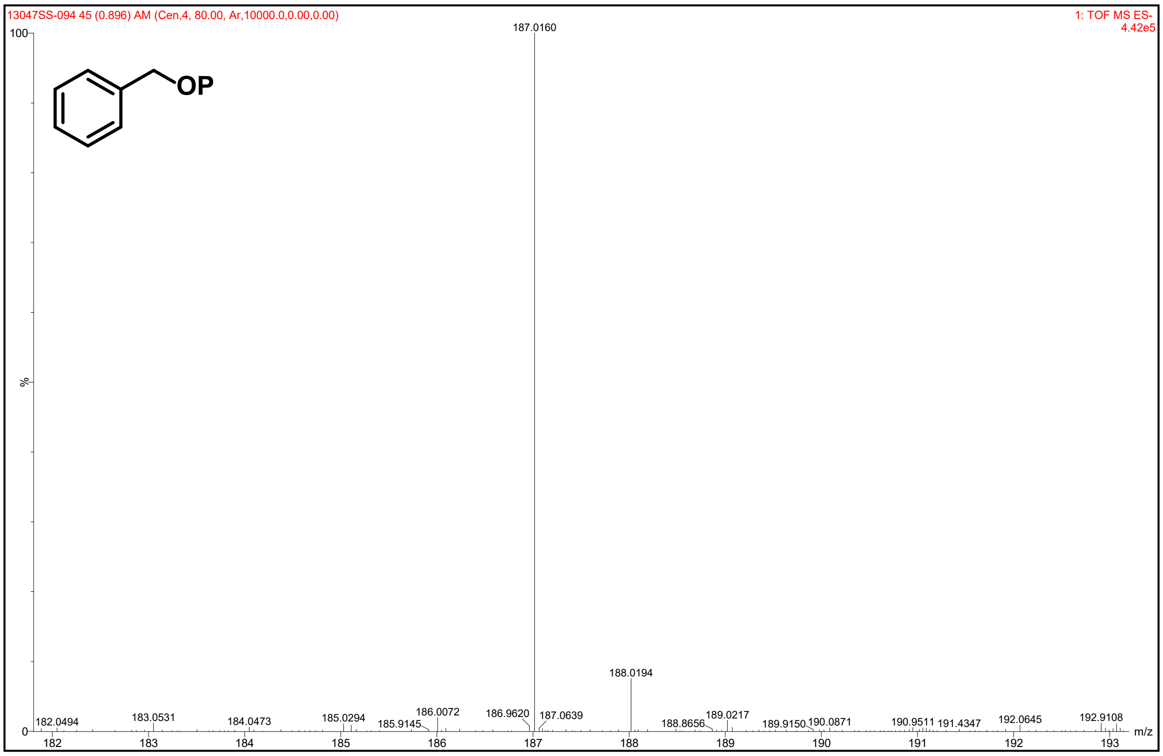

Figure S89. HRMS-ESI$^-$ of **46.**

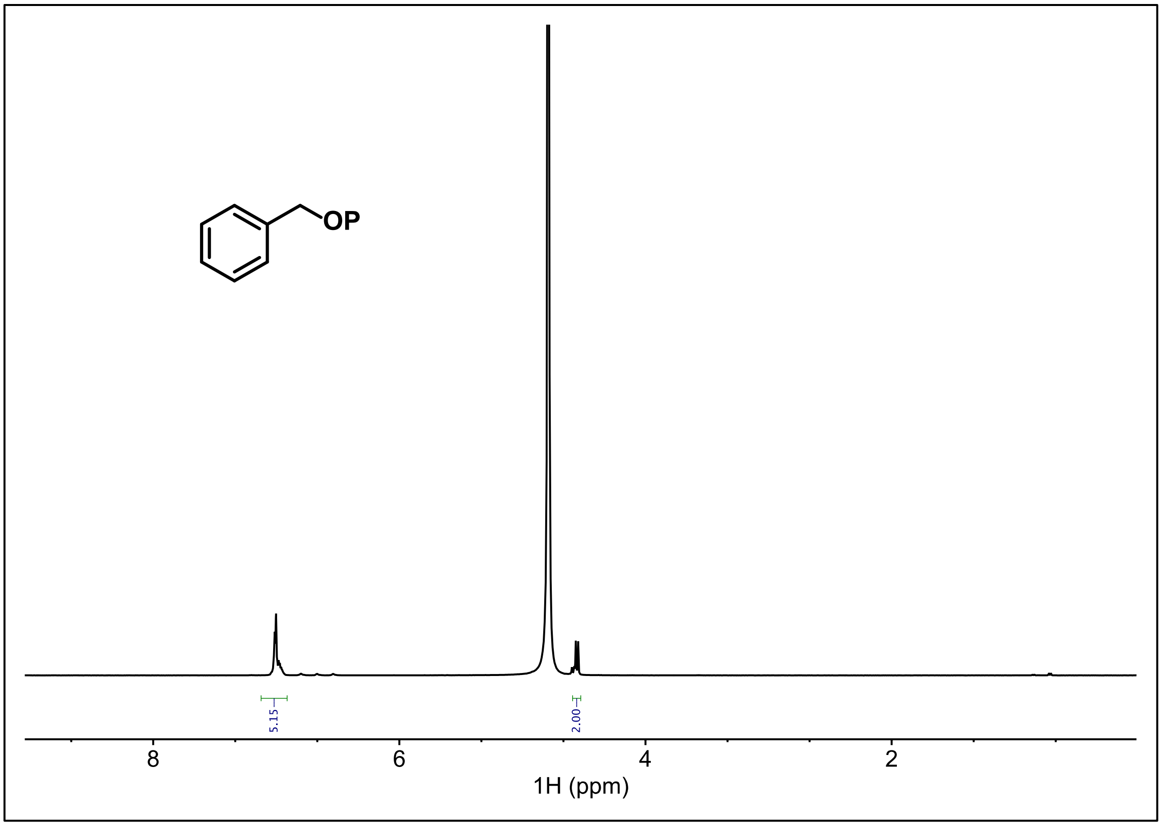

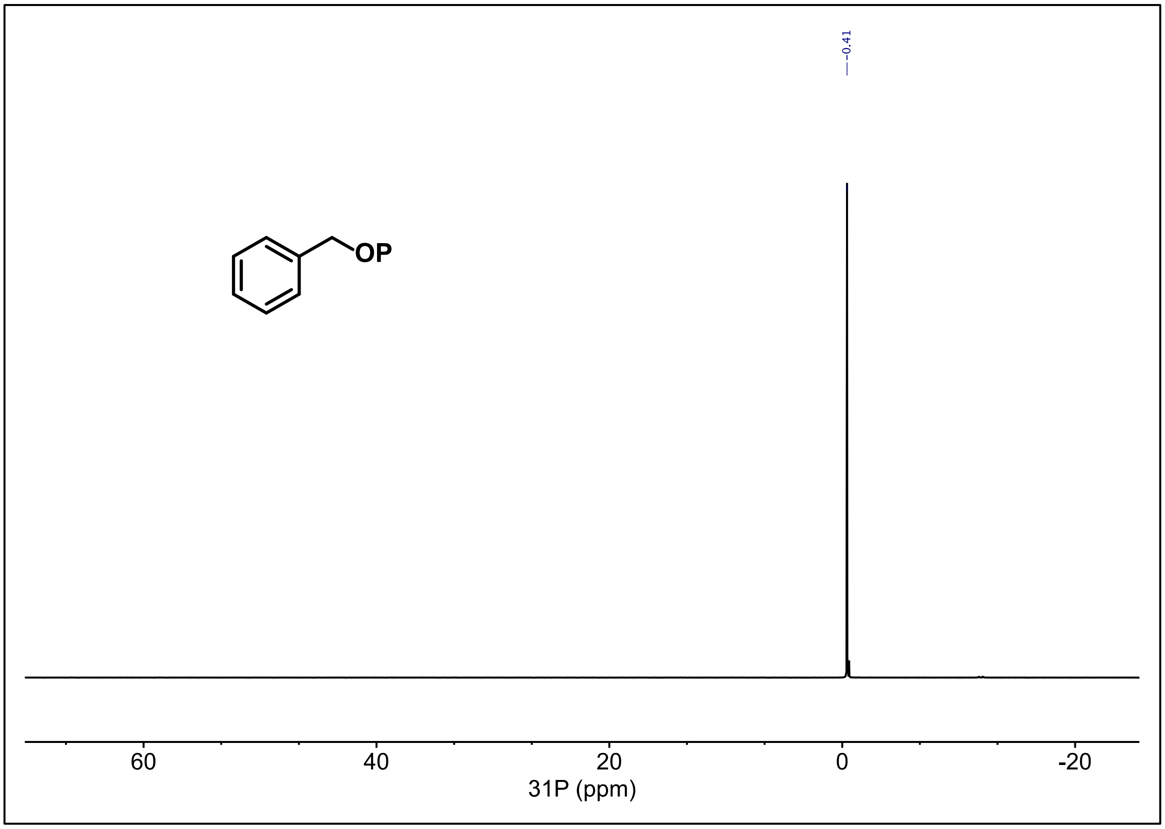

Figure S90. ^{1}H NMR (400 MHz, D_2O) and ^{31}P NMR of **46** (162 MHz, D_2O).

4-Fluorobenzyl Phosphate (47):

The title product was obtained as an ivory solid from (4-fluorophenyl)methanol following the procedure described in *Method 2.7b.*

TLC (^{i}PrOH: NH_4OH: H_2O 7:2:1 v/v): R_f = 0.64.

^{1}H NMR (400 MHz, D_2O): δ 7.53 – 7.42 (m, 2H), 7.17 (t, *J* = 8.9 Hz, 2H), 4.90 (d, *J* = 6.9 Hz, 2H).

^{31}P NMR (162 MHz, D_2O): δ 0.33.

HRMS-ESI: Calculated for $C_7H_7FO_4P$ $[M-H]^-$: 205.00659; Found: 205.0060.

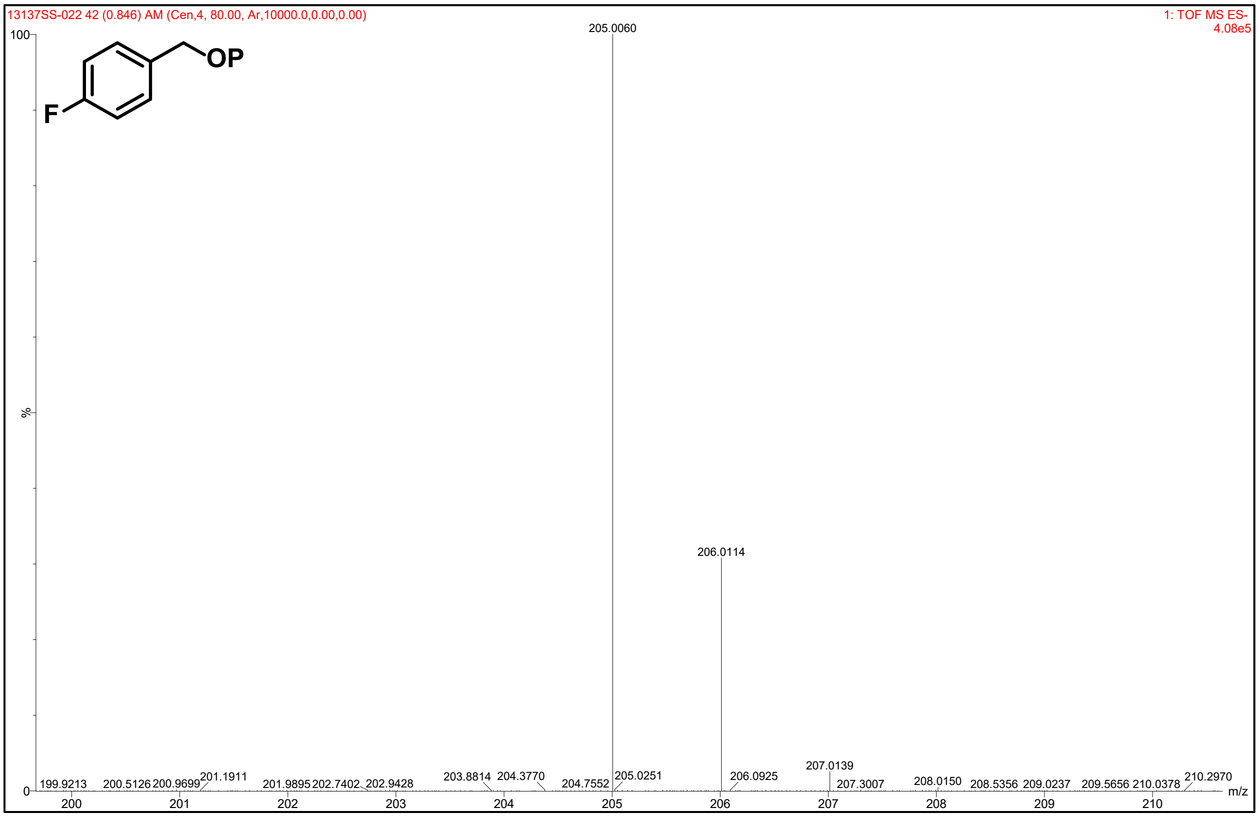

Figure S91. HRMS-ESI$^-$ of **47.**

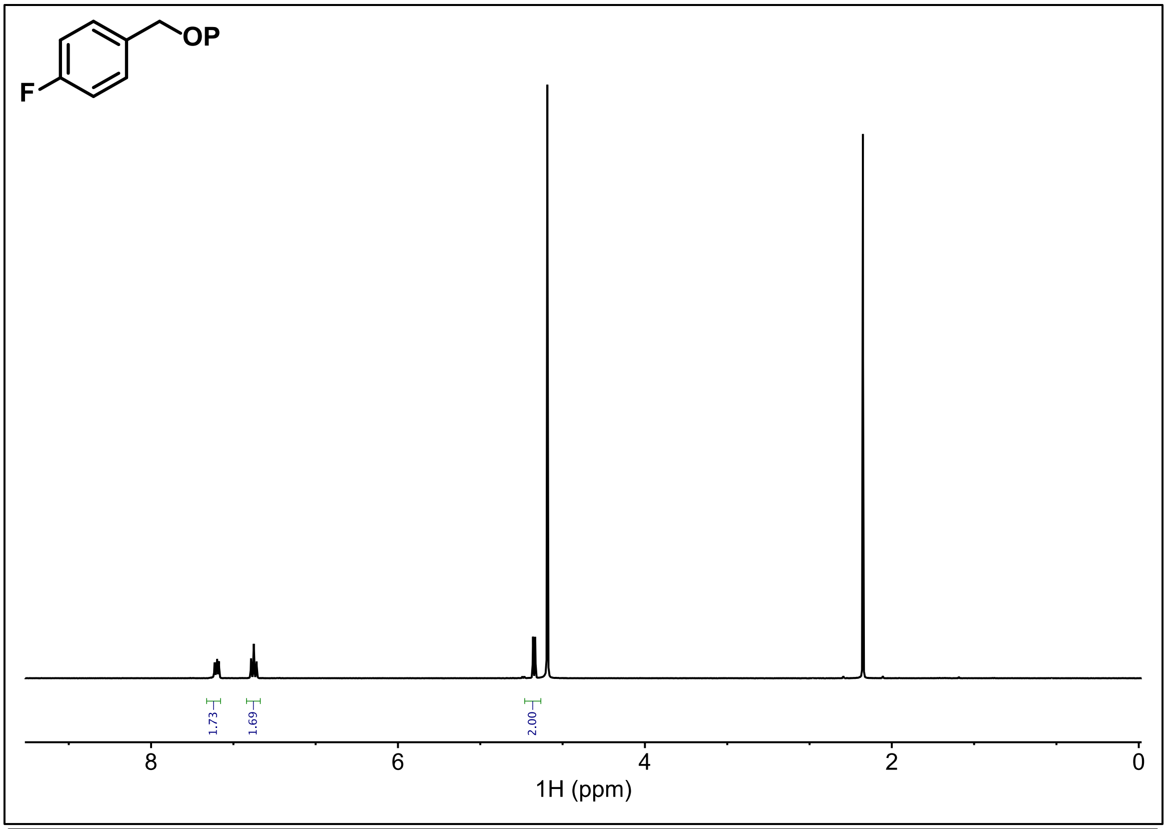

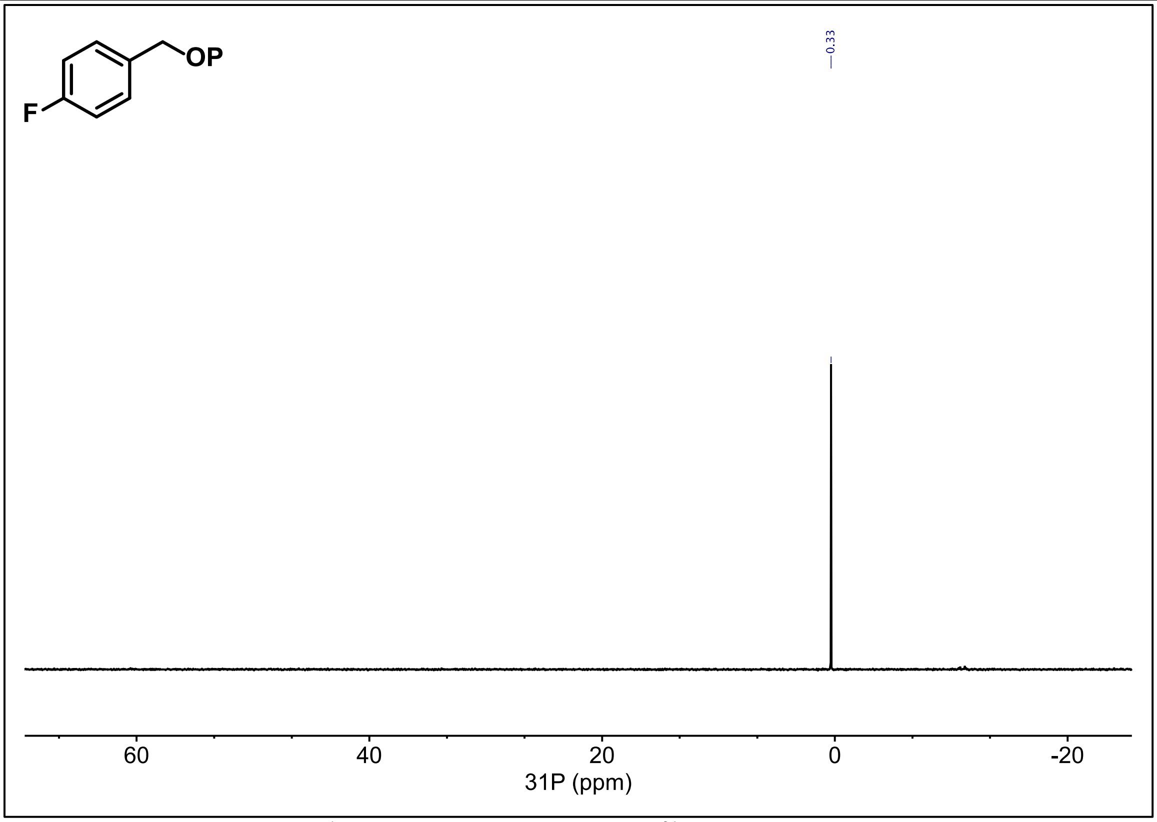

Figure S92. ^{1}H NMR (400 MHz, D_2O) and ^{31}P NMR of **47** (162 MHz, D_2O).

3,5-Difluorobenzyl Phosphate (48):

The title product was obtained as a brown solid from (3,5-difluorophenyl)methanol following the procedure described in *Method 2.7b.*

<u>TLC (^{i}PrOH: NH_4OH: H_2O 7:2:1 v/v):</u> R_f = 0.61.

<u>^{1}H NMR (300 MHz, D_2O):</u> δ 7.01 (d, *J* = 7.4 Hz, 2H), 6.98 – 6.77 (m, 1H), 4.85 (d, *J* = 7.1 Hz, 2H).

<u>^{31}P NMR (122 MHz, D_2O):</u> δ 0.96.

<u>HRMS-ESI:</u> Calculated for $C_7H_6F_2O_4P$ $[M-H]^-$: 222.99717; Found: 222.9980.

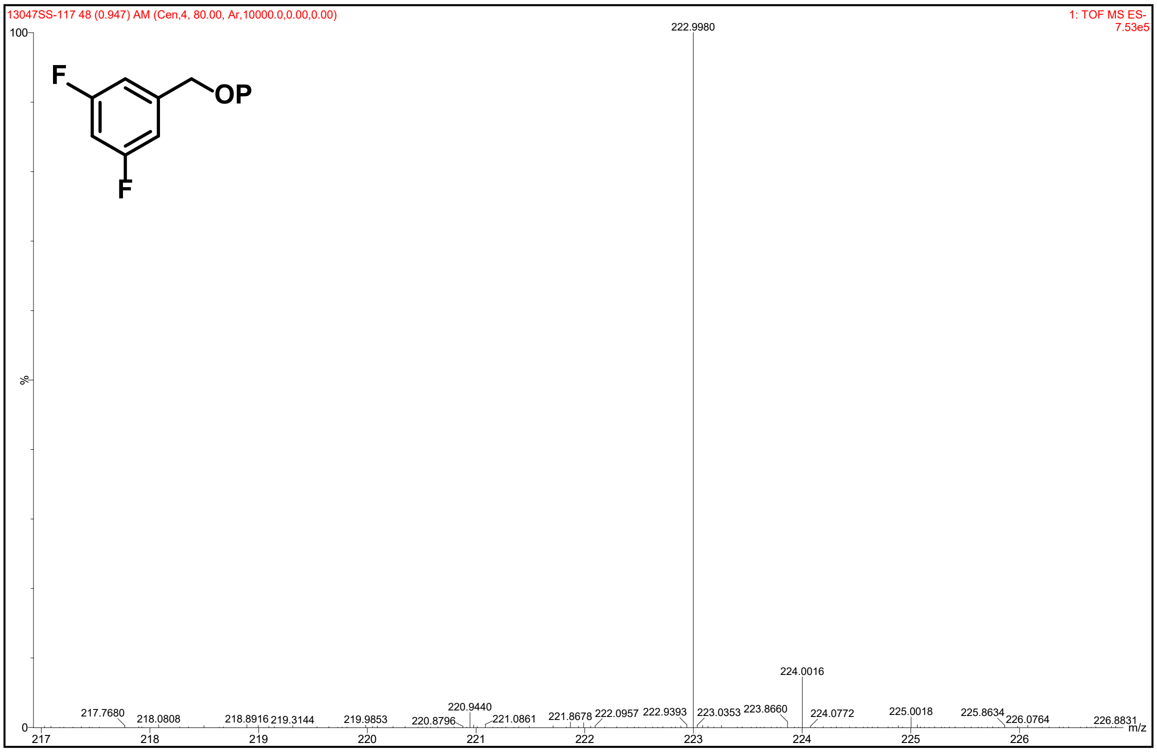

Figure S93. HRMS-ESI$^-$ of **48.**

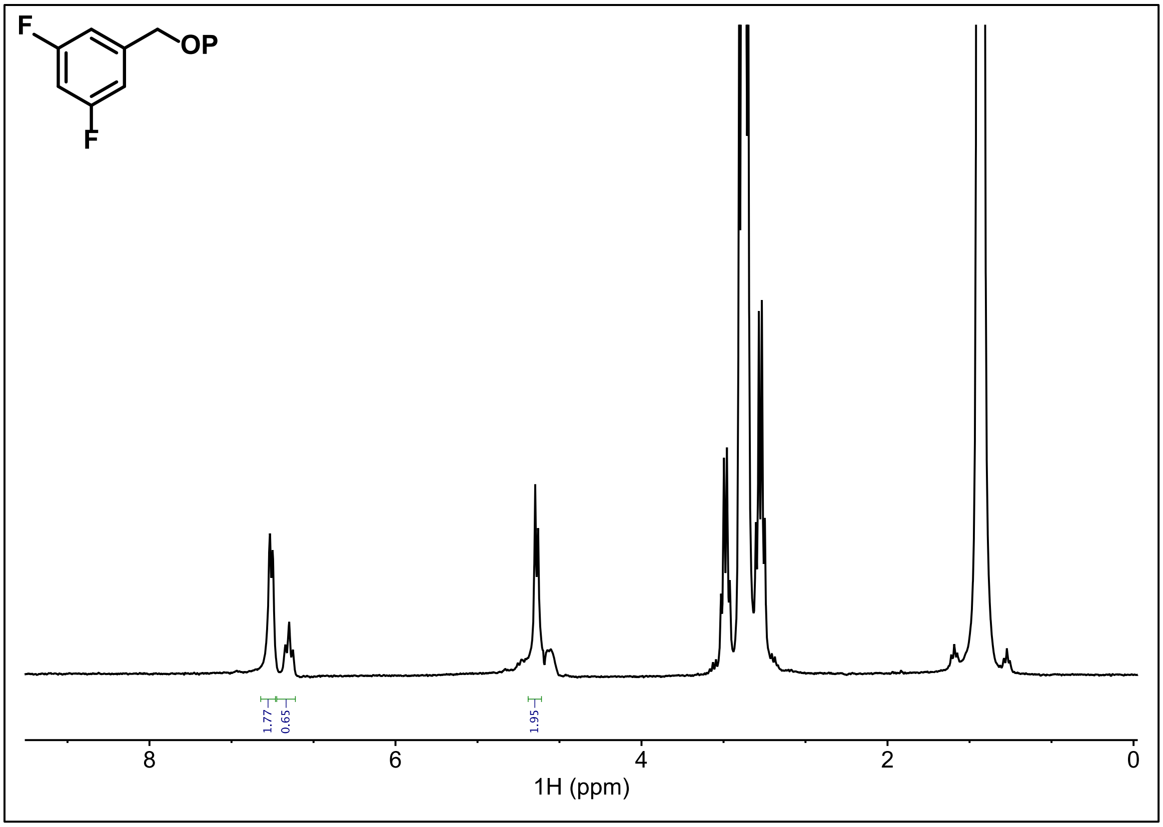

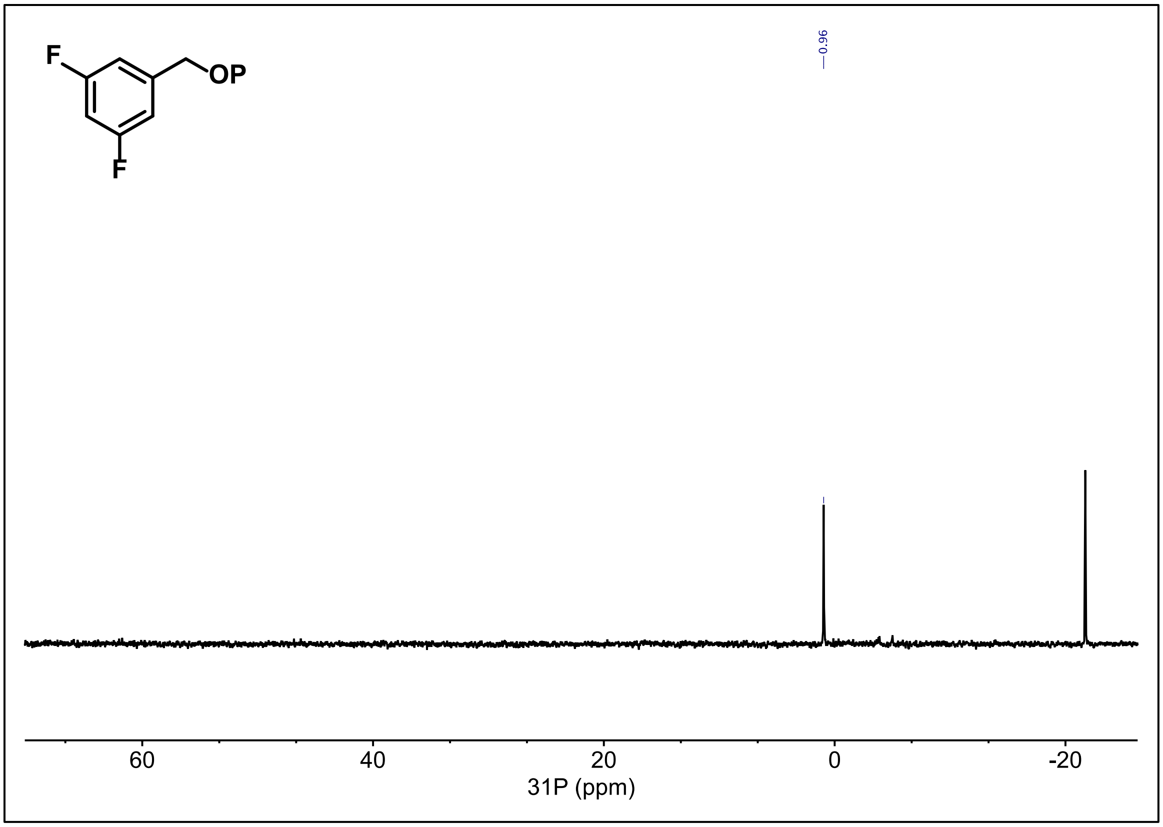

Figure S94. ^{1}H NMR (300 MHz, D_2O) and ^{31}P NMR of **48** (122 MHz, D_2O).

4-Chlorobenzyl Phosphate (49):

The title product was obtained as an ivory solid from ((4-chlorophenyl)methanol following the procedure described in *Method 2.7b.*

TLC (^{i}PrOH: NH_4OH: H_2O 7:2:1 v/v): R_f = 0.60.

^{1}H NMR (300 MHz, D_2O): δ 5.52 (d, *J* = 6.9 Hz, 1H), 5.40 (t, *J* = 7.3 Hz, 1H), 4.32 (t, *J* = 6.6 Hz, 2H), 4.10 (s, 2H), 3.98 (s, 2H), 2.83 (s, 1H), 2.36 – 1.96 (m, 4H), 1.64 (d, *J* = 17.4 Hz, 6H).

^{31}P NMR (122 MHz, D_2O): δ 1.66.

HRMS-ESI: Calculated for $C_7H_7ClO_4P$ $[M-H]^-$: 220.97704; Found: 220.9778.

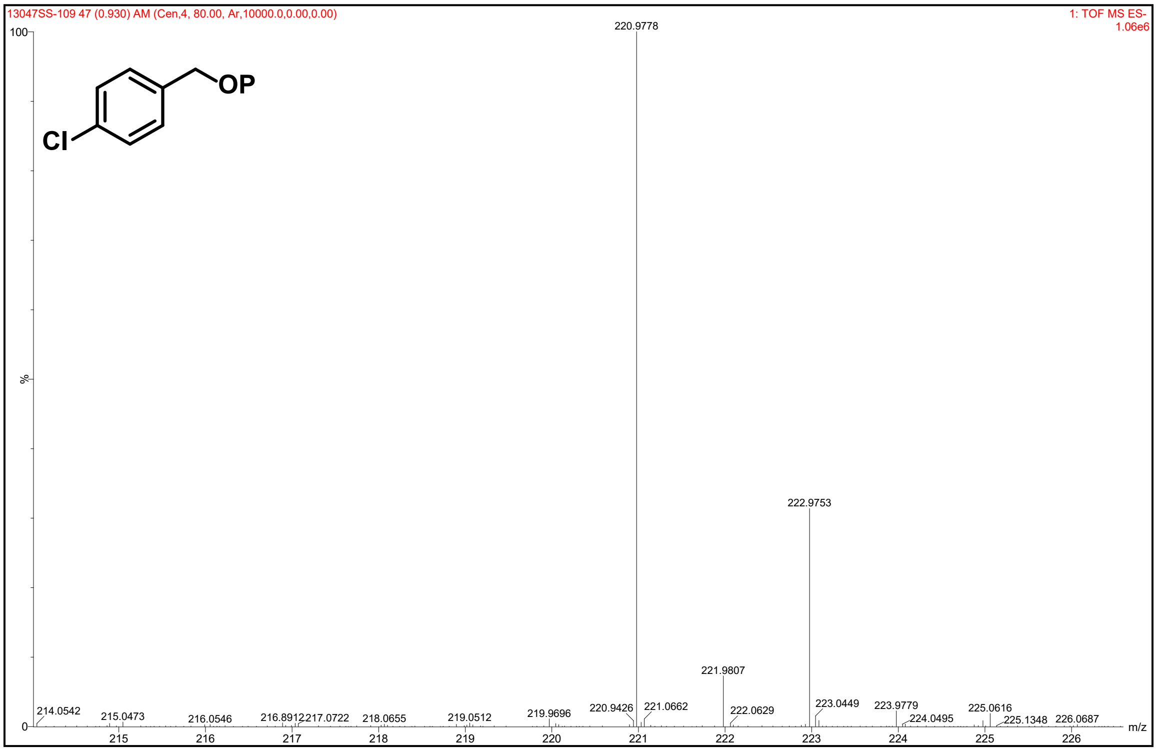

Figure S95. HRMS-ESI$^-$ of **49.**

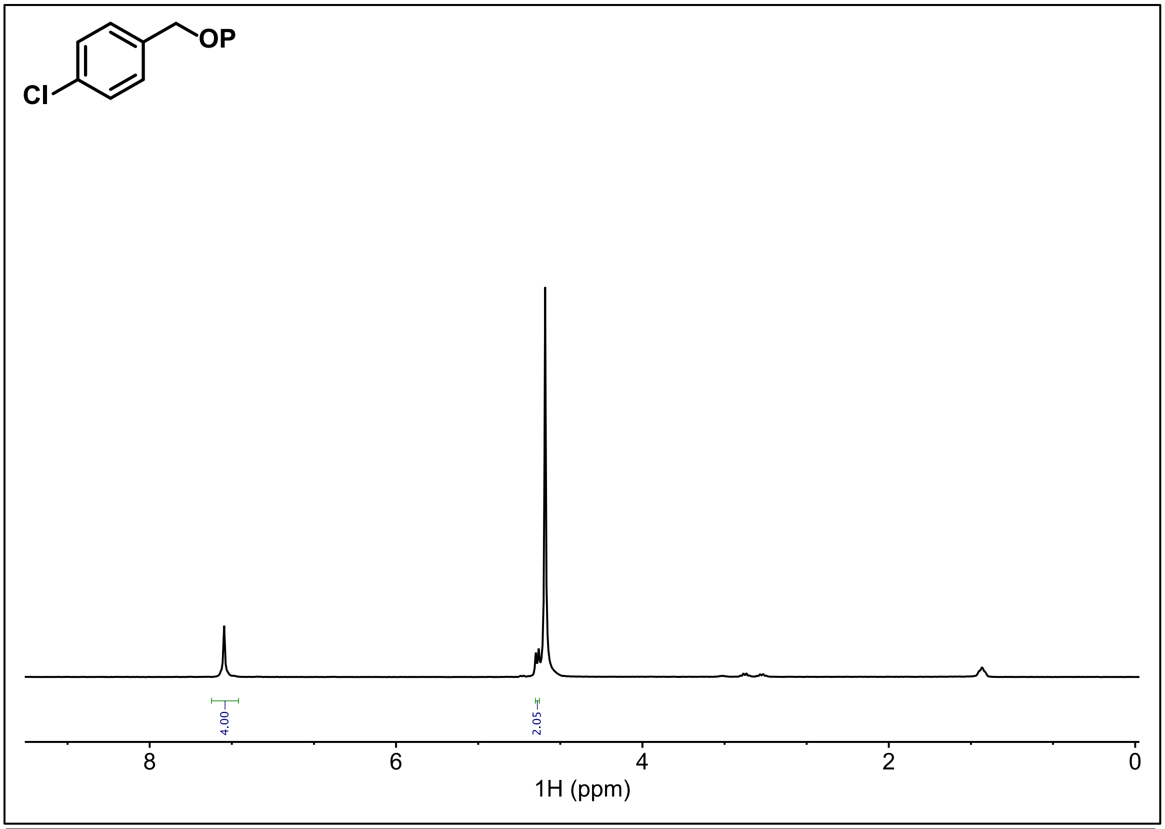

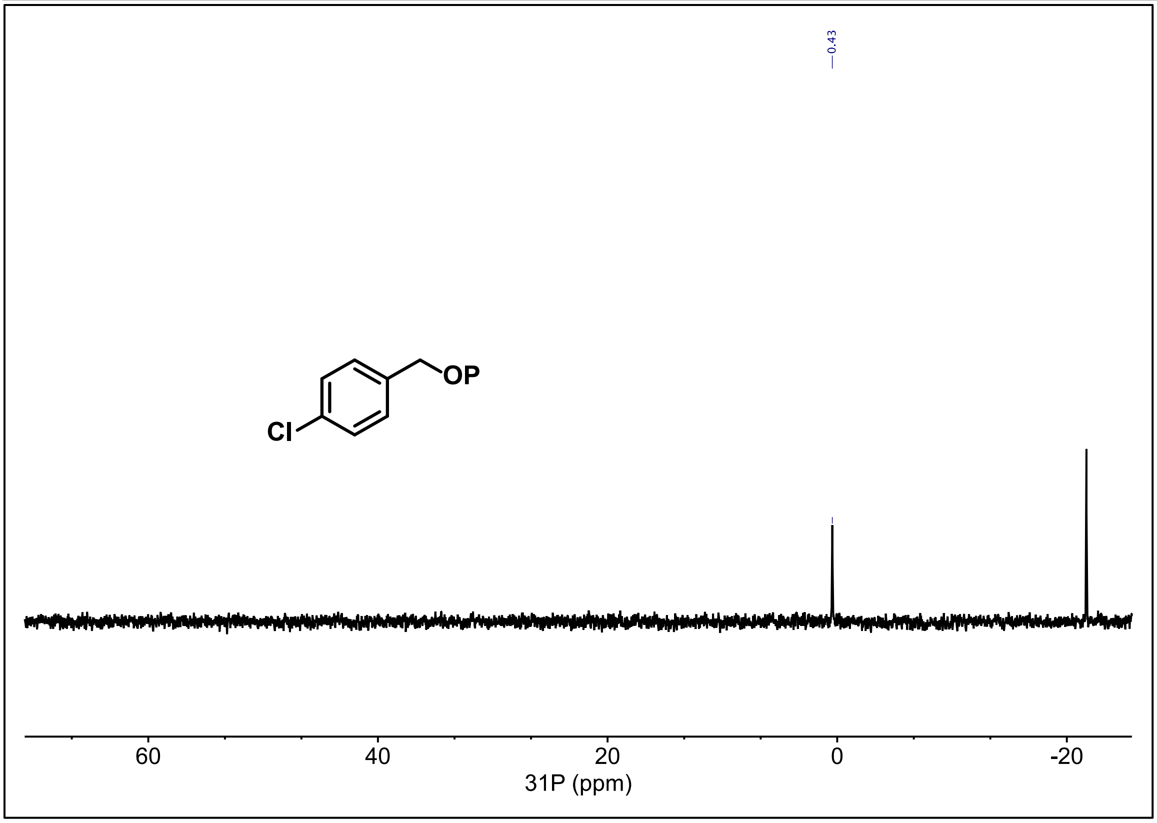

Figure S96. ^{1}H NMR (300 MHz, D_2O) and ^{31}P NMR of **49** (122 MHz, D_2O).

4-Methylbenzyl Phosphate (50):

The title product was obtained as a white-brown solid from *p*-tolylmethanol following the procedure described in *Method 2.7b.*

TLC (^{i}PrOH: NH_4OH: H_2O 7:2:1 v/v): R_f = 0.62.

^{1}H NMR (300 MHz, D_2O): δ 7.32 (d, *J* = 7.8 Hz, 2H), 7.24 (d, *J* = 7.8 Hz, 2H), 4.82 (d, *J* = 6.7 Hz, 2H), 2.31 (s, 3H).

^{31}P NMR (122 MHz, D_2O): δ 0.72.

HRMS-ESI: Calculated for $C_8H_{10}O_4P$ $[M-H]^-$: 201.03166; Found: 201.0326.

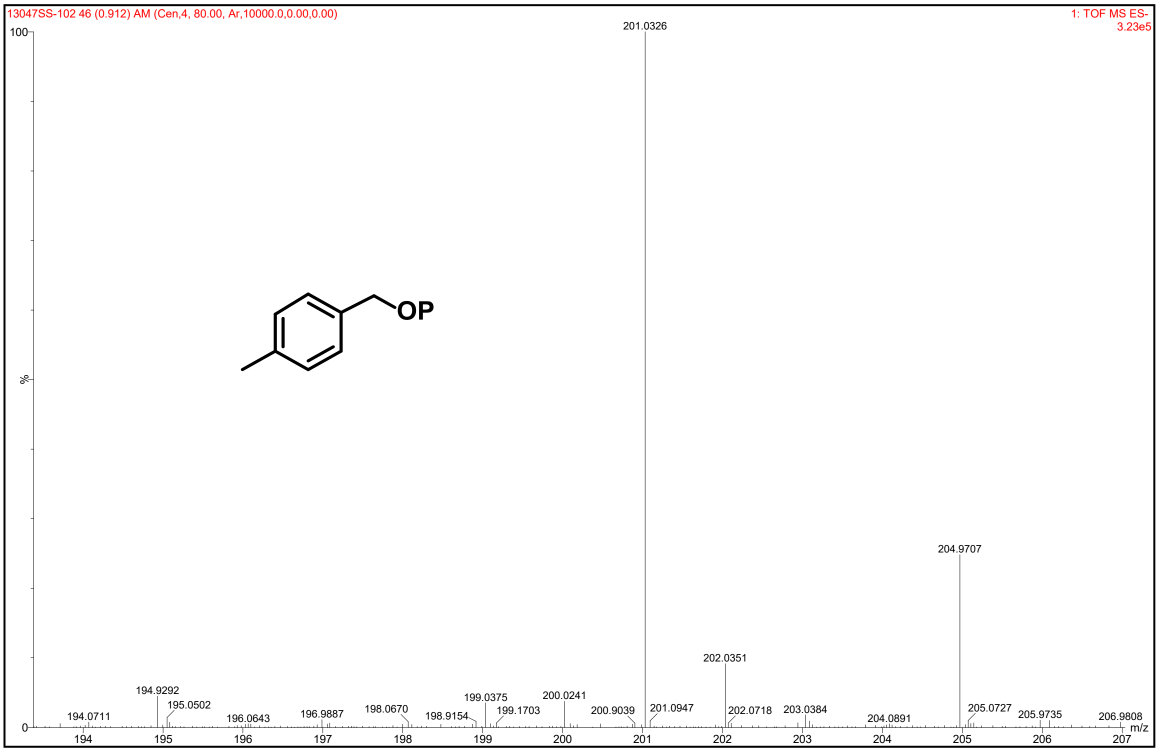

Figure S97. HRMS-ESI$^-$ of **50.**

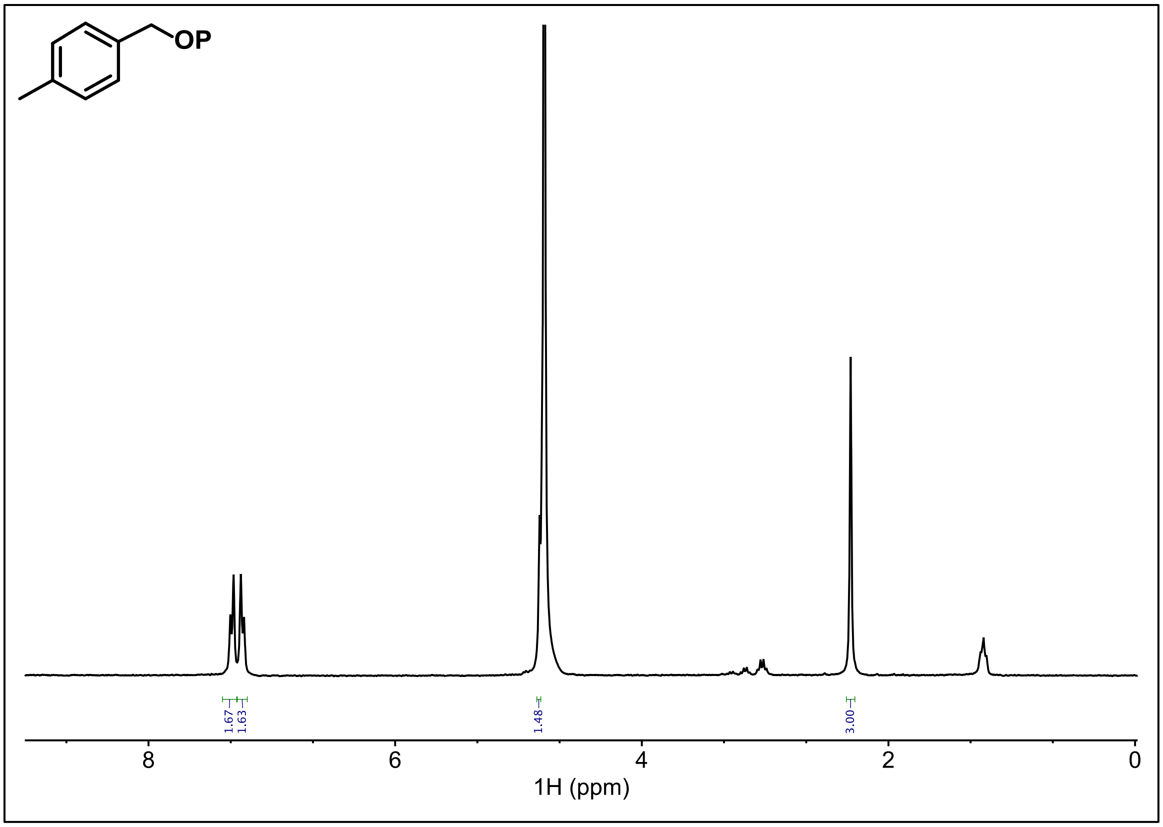

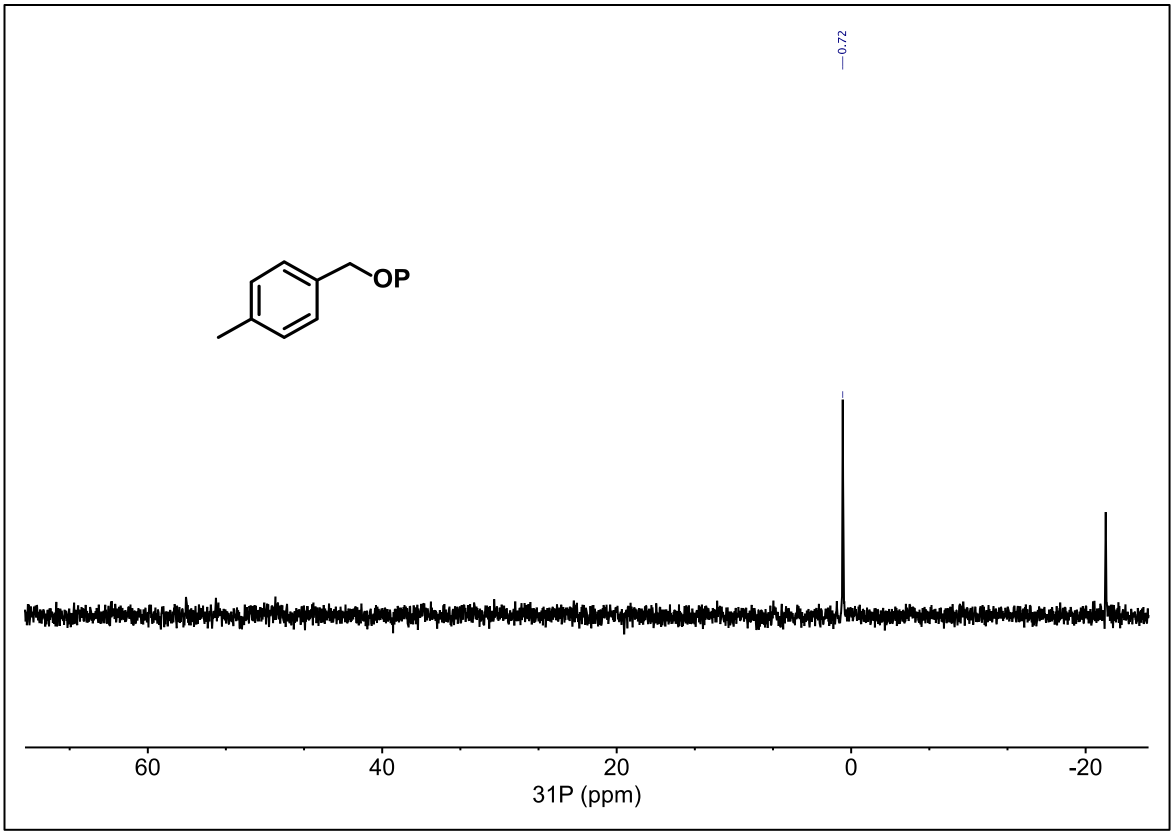

Figure S98. ^{1}H NMR (300 MHz, D_2O) and ^{31}P NMR of **50** (122 MHz, D_2O).

4-Nitrobenzyl Phosphate (51):

The title product was obtained as a yellow-brown solid from (4-nitrophenyl)methanol following the procedure described in *Method 2.7b.*

<u>TLC (^{i}PrOH: NH_4OH: H_2O 7:2:1 v/v):</u> R_f = 0.65.

<u>^{1}H NMR (400 MHz, D_2O):</u> δ 8.27 (d, *J* = 8.8 Hz, 2H), 7.65 (d, *J* = 8.5 Hz, 2H), 5.05 (d, *J* = 7.6 Hz, 2H).

<u>^{31}P NMR (162 MHz, D_2O):</u> δ 0.51.

<u>HRMS-ESI:</u> Calculated for $C_7H_7NO_6P$ $[M-H]^-$: 232.00109; Found: 232.0021.

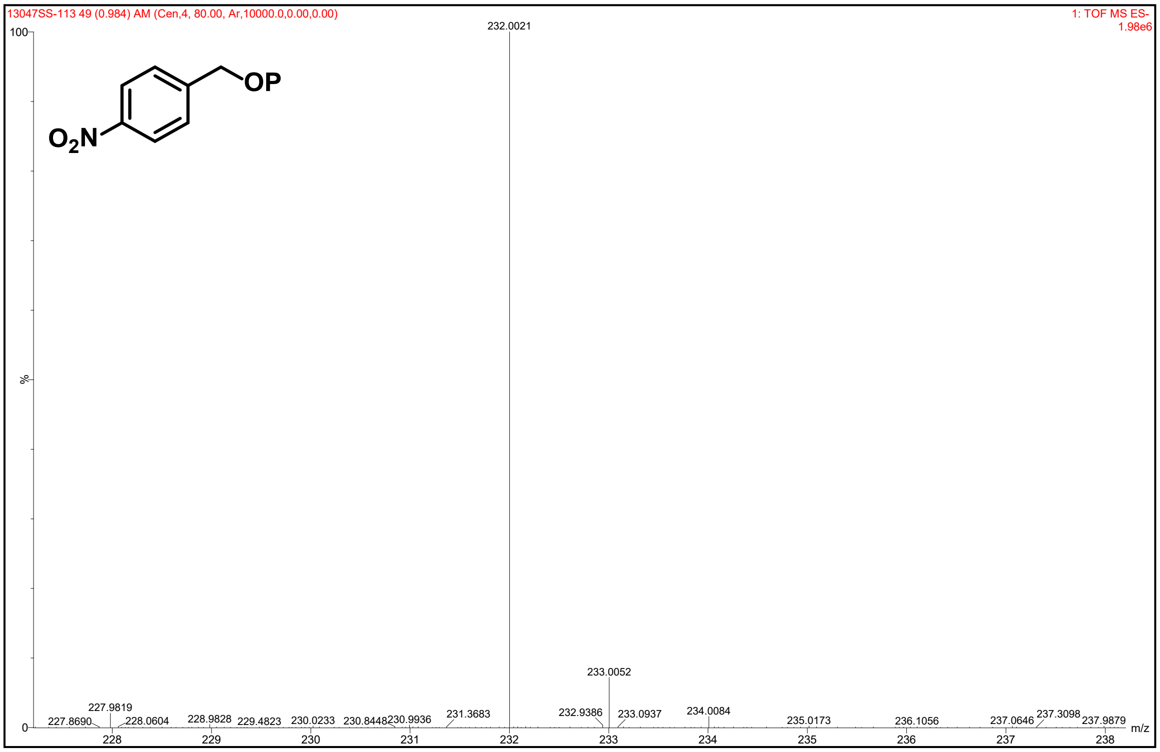

Figure S99. HRMS-ESI$^-$ of **51.**

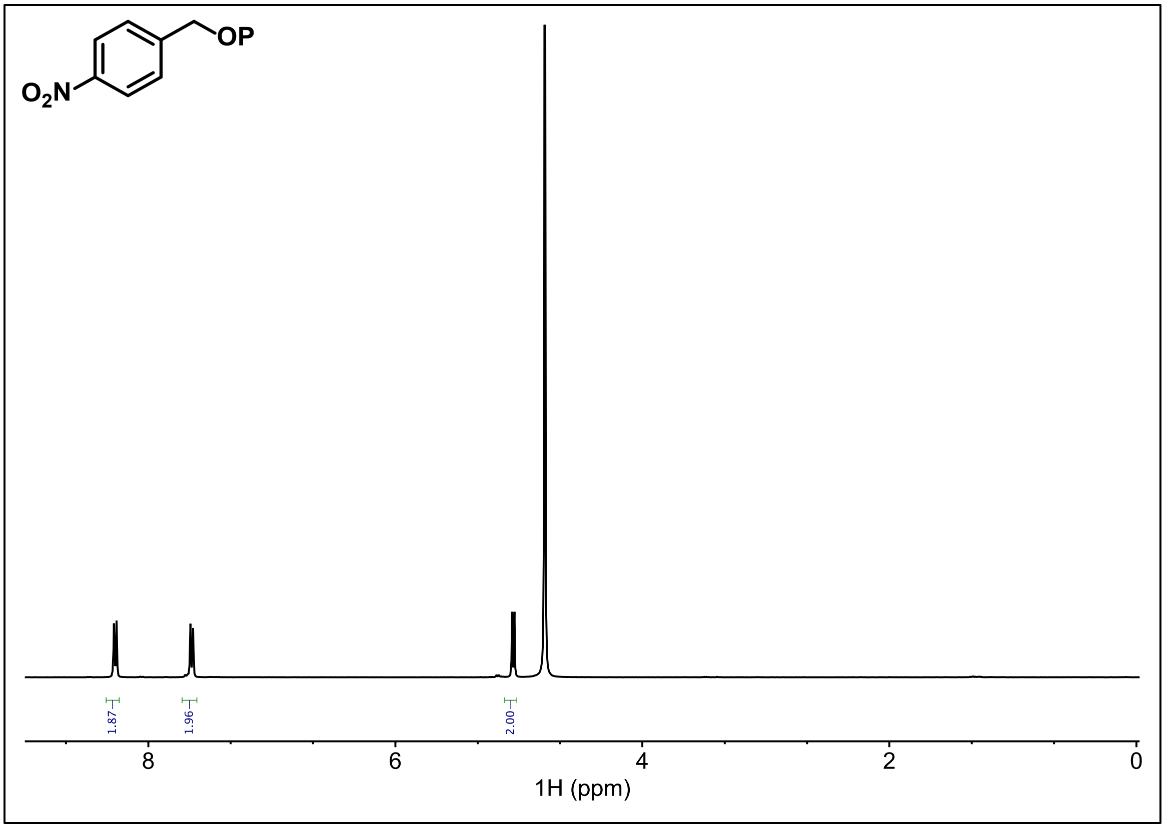

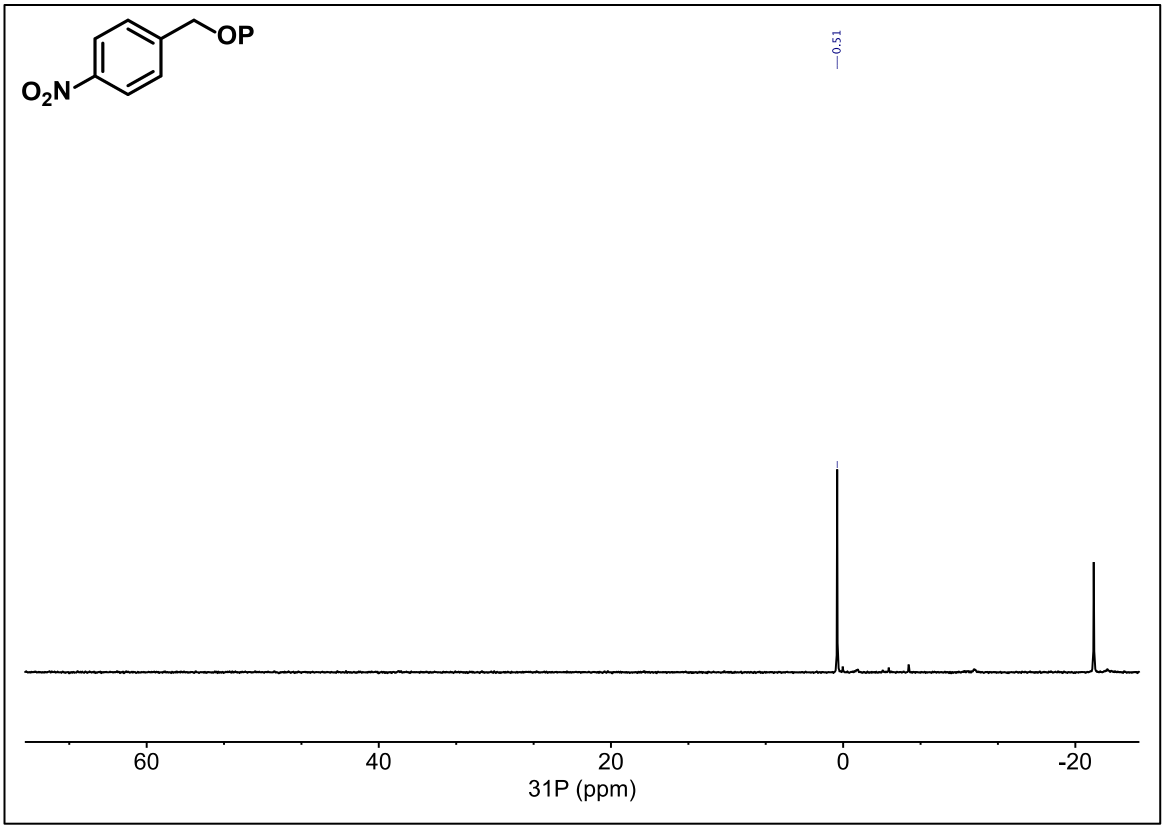

Figure S100. ^{1}H NMR (400 MHz, D_2O) and ^{31}P NMR of **51** (162 MHz, D_2O).

4-Methoxybenzyl Phosphate (52):

The title product was obtained as an ivory solid from (4-methoxyphenyl)methanol following the procedure described in *Method 2.7b*.

TLC (^{i}PrOH: NH_4OH: H_2O 7:2:1 v/v): R_f = 0.61.

^{1}H NMR (400 MHz, D_2O): δ 7.43 (d, *J* = 8.7 Hz, 1H), 7.04 (d, *J* = 8.7 Hz, 1H), 4.87 (d, *J* = 6.6 Hz, 1H), 3.86 (s, 2H).

^{31}P NMR (162 MHz, D_2O): δ 0.24.

HRMS-ESI: Calculated for $C_8H_{10}O_5P$ $[M-H]^-$: 217.02658; Found: 217.0270.

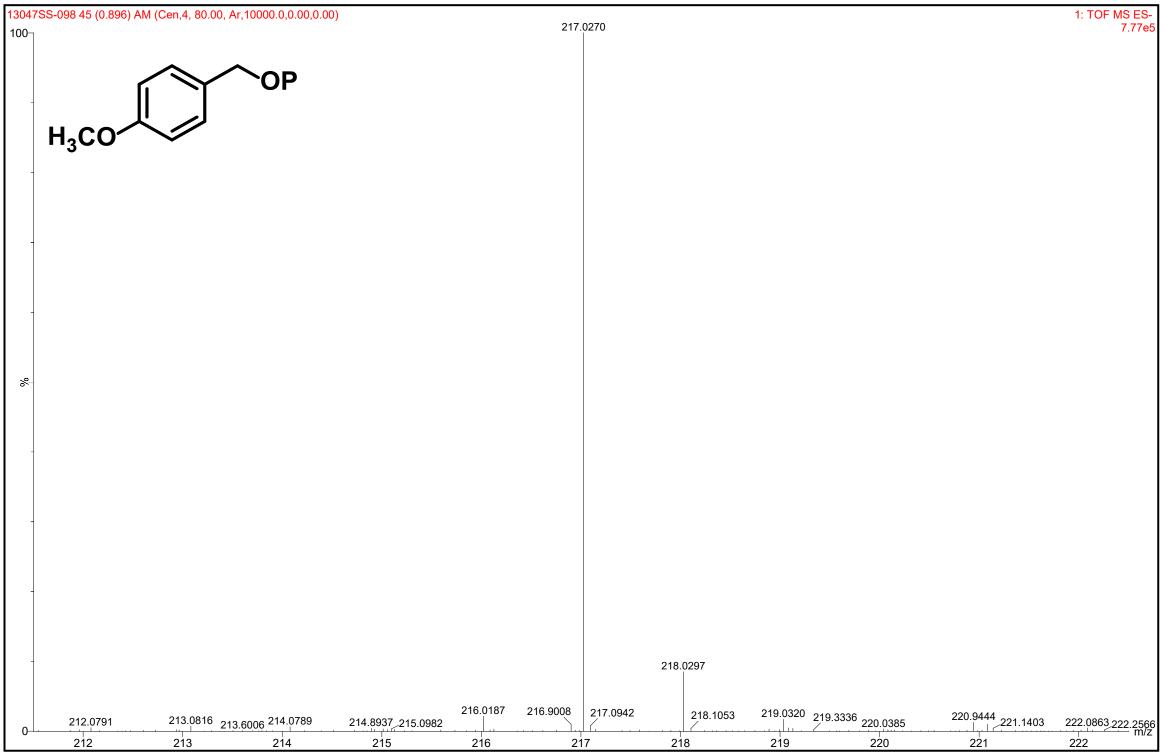

Figure S101. HRMS-ESI$^-$ of **52.**

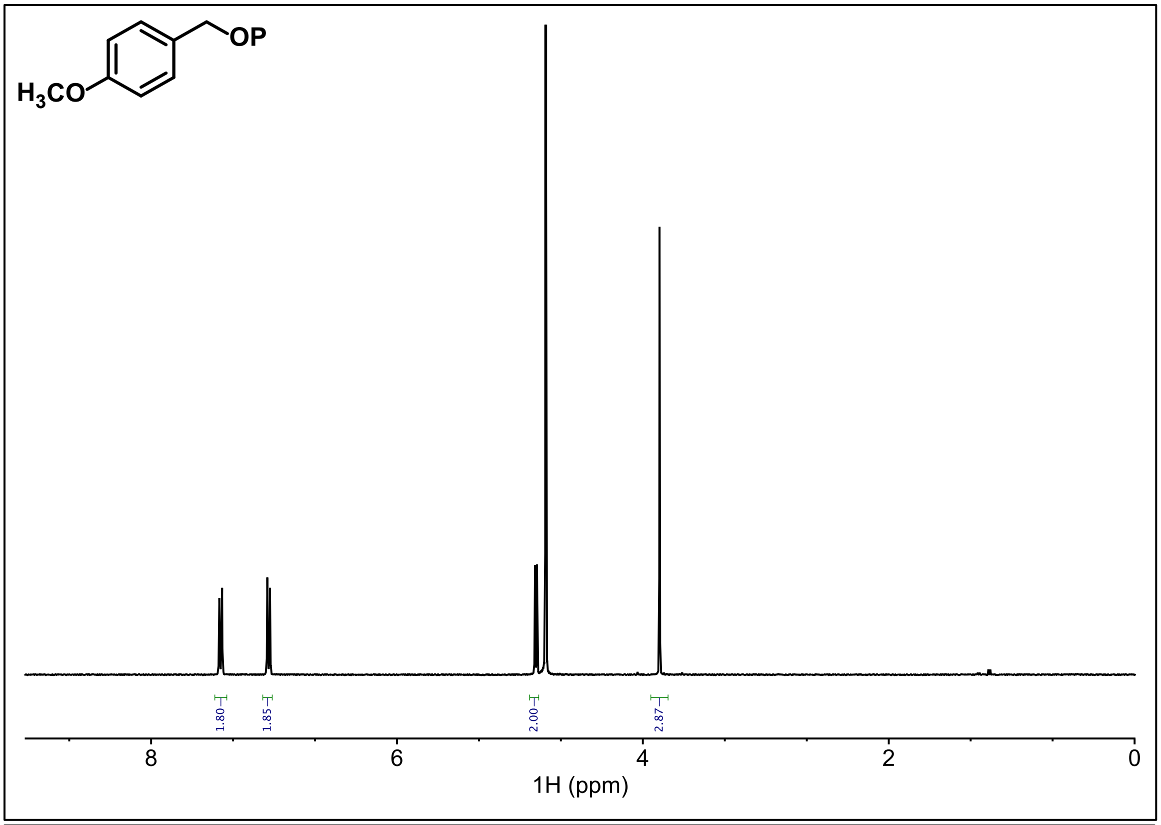

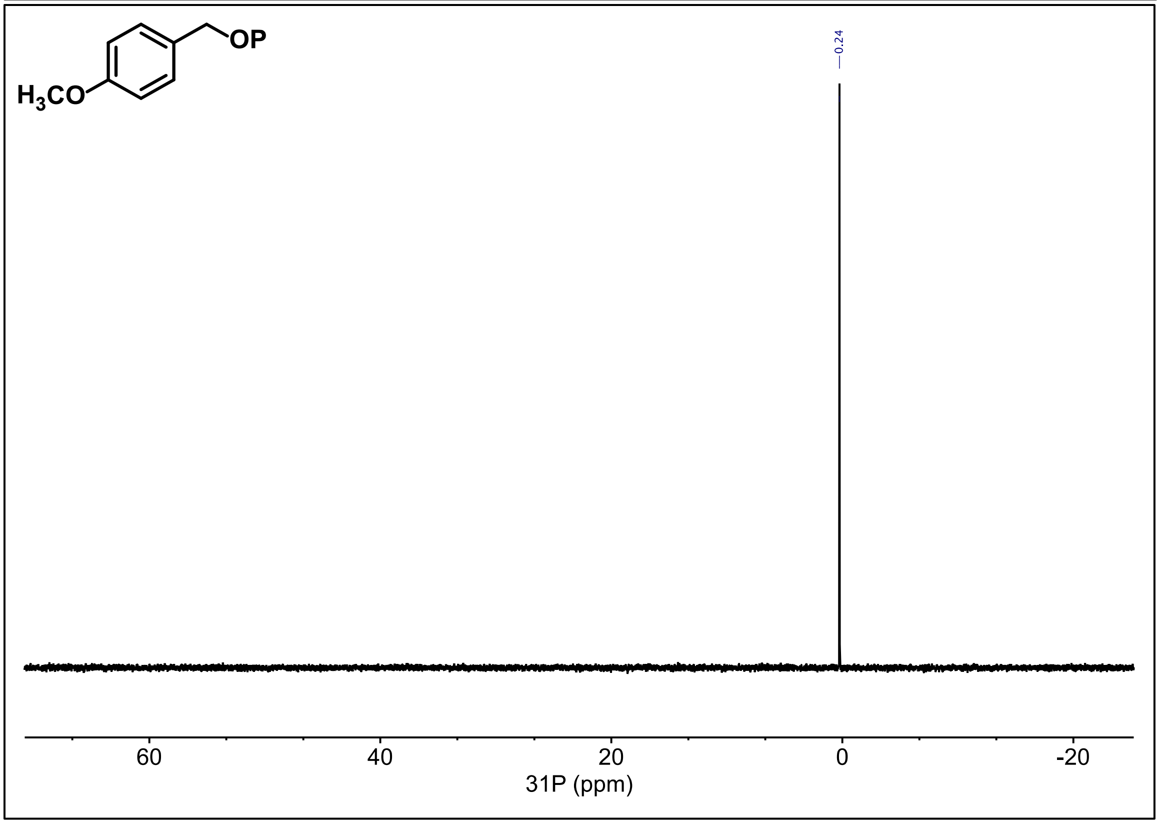

Figure S102. ^{1}H NMR (400 MHz, D_2O) and ^{31}P NMR of **52** (162 MHz, D_2O).

2-Methoxybenzyl Phosphate (53):
The title product was obtained as a brown-white solid from (2-methoxyphenyl)methanol following the procedure described in *Method 2.7b.*
TLC (^{i}PrOH: NH_4OH: H_2O 6:2:1 v/v): R_f = 0.61.
^{1}H NMR (300 MHz, D_2O): δ 7.52 – 7.23 (m, 2H), 7.11 – 6.80 (m, 2H), 4.86 (d, J = 6.1 Hz, 2H), 3.83 (s, 3H).
^{31}P NMR (122 MHz, D_2O): δ 1.78.
HRMS-ESI: Calculated for $C_8H_{10}O_5P$ [M-H]$^-$: 217.02658; Found: 217.0256.

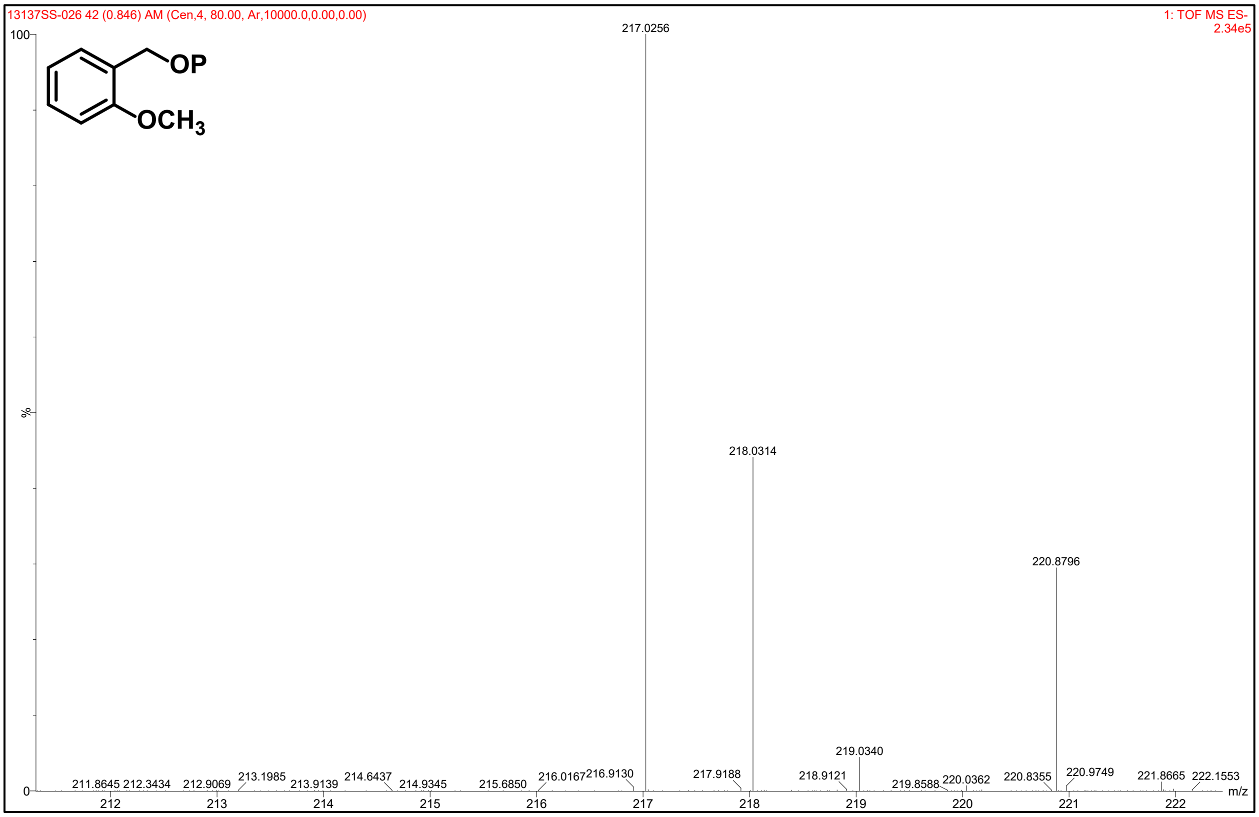

Figure S103. HRMS-ESI$^-$ of **53.**

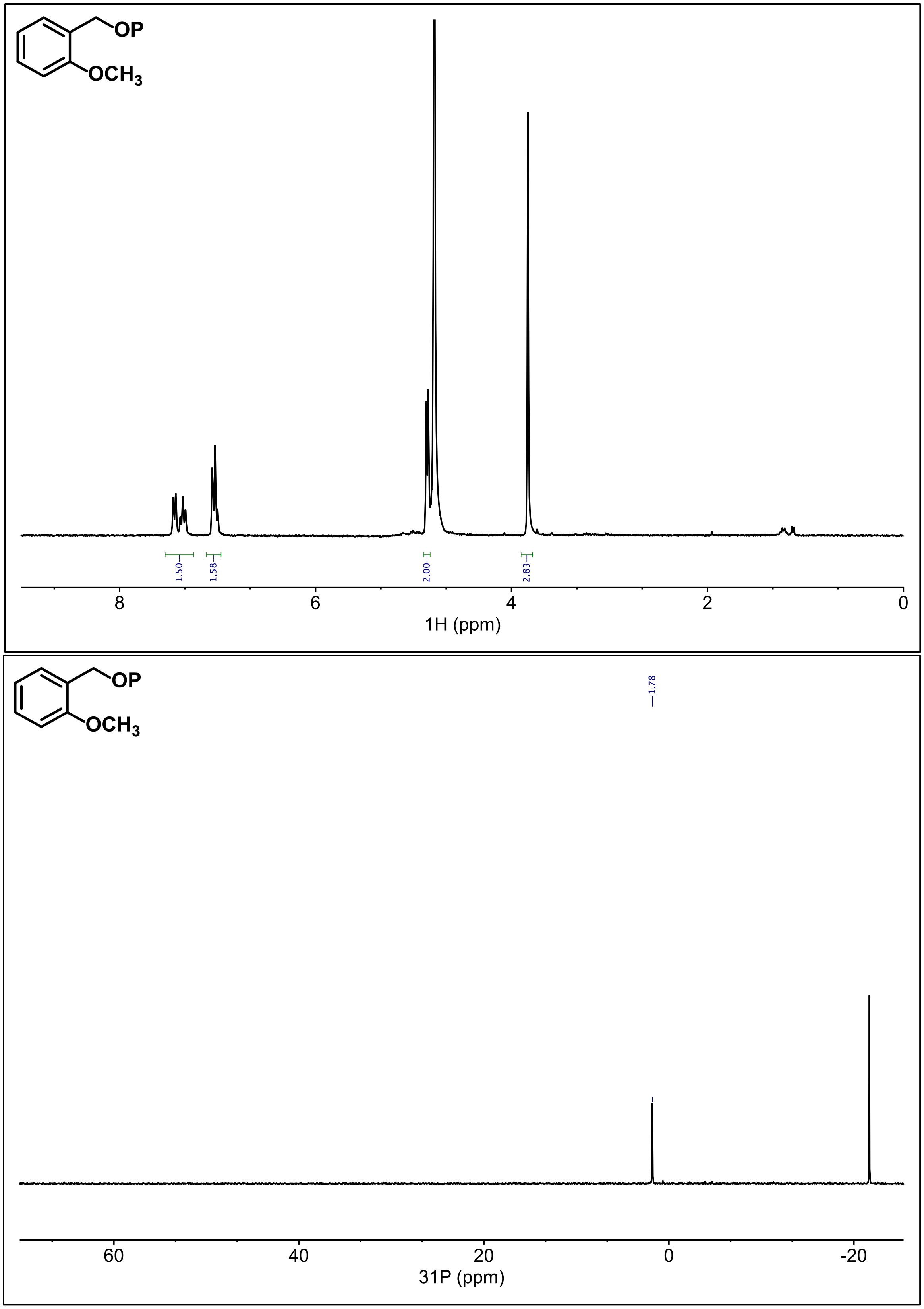

Figure S104. ^{1}H NMR (300 MHz, D_2O) and ^{31}P NMR of **53** (122 MHz, D_2O).

Benzo[*d*][1,3]dioxol-5-ylmethyl Phosphate (54):
The title product was obtained as a white solid from benzo[*d*][1,3]dioxol-5-ylmethanol following the procedure described in *Method 2.7b.*
TLC (^{i}PrOH: NH_4OH: H_2O 7:2:1 v/v): R_f = 0.61.
^{1}H NMR (300 MHz, D_2O): δ 6.89 (dt, *J* = 16.7, 7.9 Hz, 3H), 5.94 (s, 2H), 4.77 (d, *J* = 10.2 Hz, 2H).
^{31}P NMR (122 MHz, D_2O): δ 0.16.
HRMS-ESI: Calculated for $C_8H_8O_6P$ [M-H]$^-$: 231.00584; Found: 231.0070.

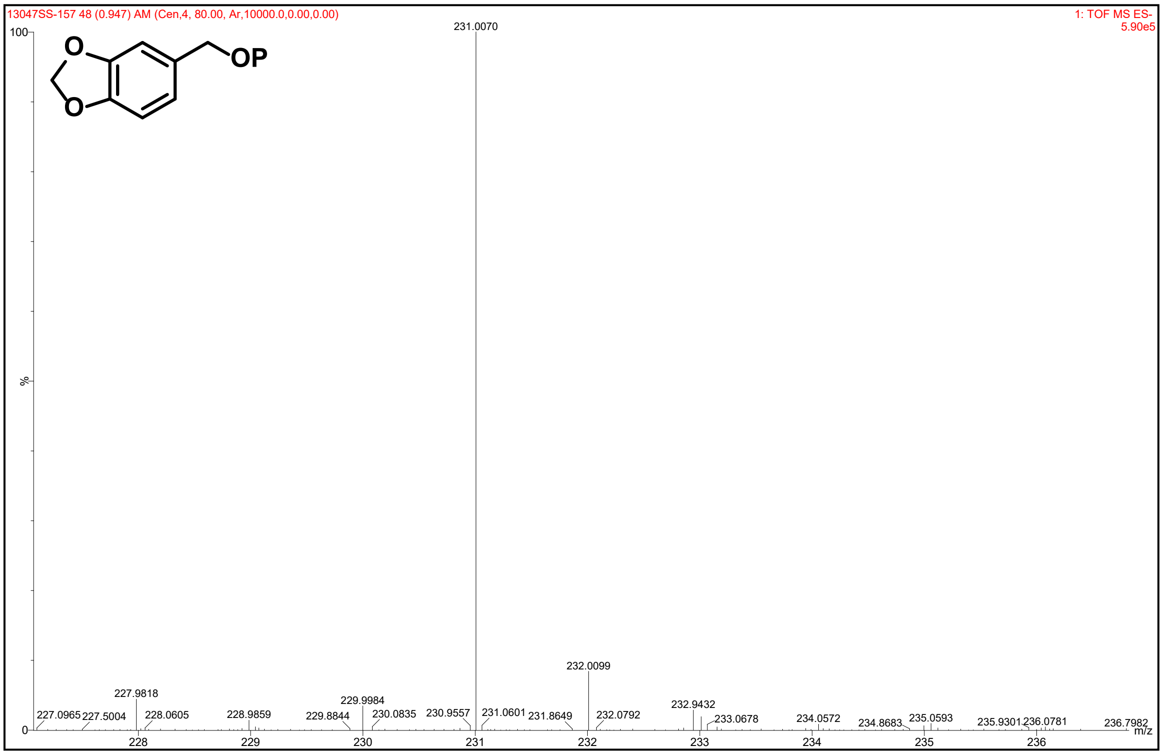

Figure S105. HRMS-ESI$^-$ of **54.**

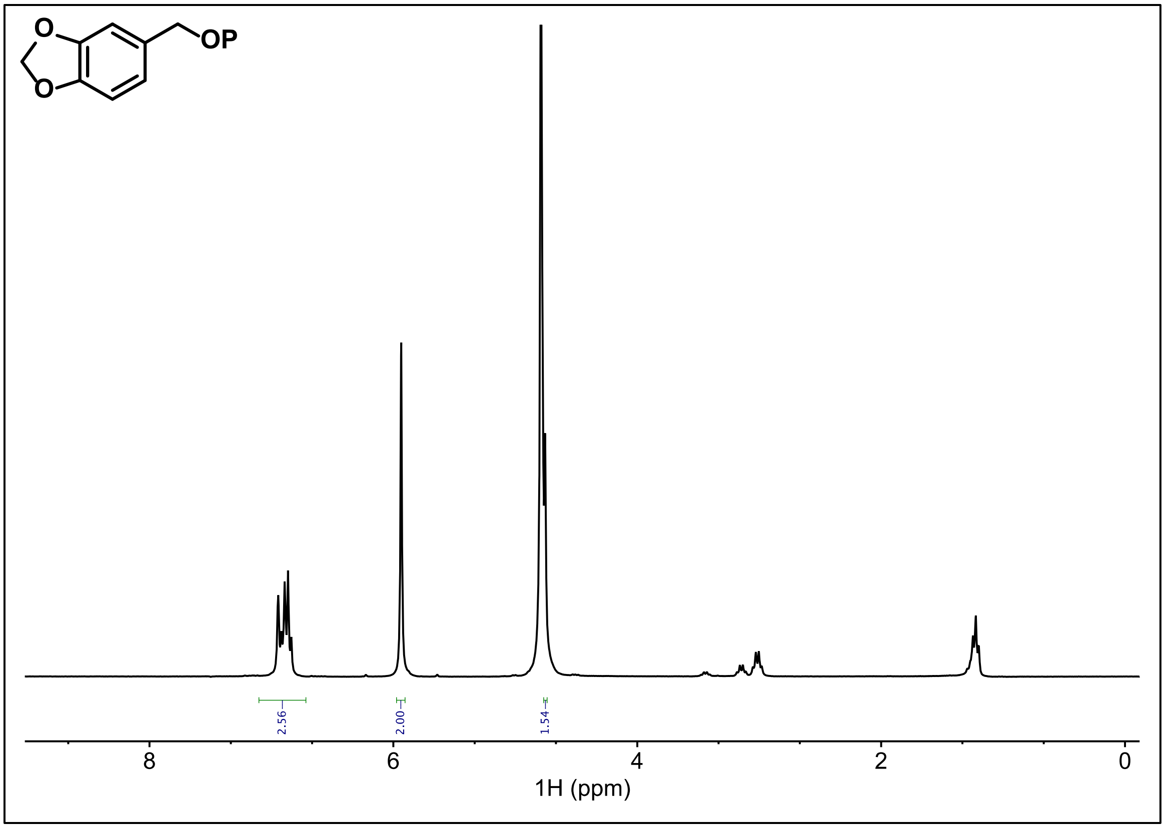

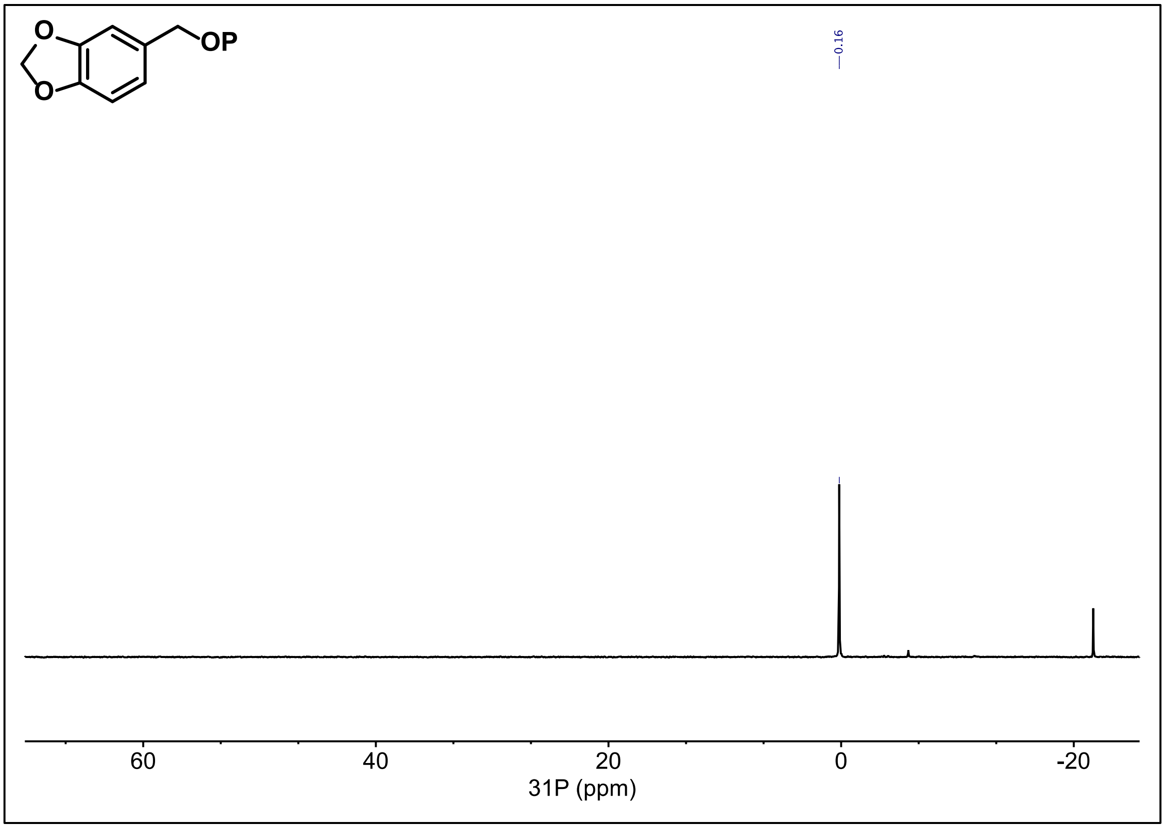

Figure S106. ^{1}H NMR (300 MHz, D_2O) and ^{31}P NMR of **54** (122 MHz, D_2O)**.**

3,4,5-Trimethoxybenzyl Phosphate (55):
The title product was obtained as an ivory solid from (3,4,5-trimethoxyphenyl)methanol following the procedure described in *Method 2.7b.*
TLC (^{i}PrOH: NH_4OH: H_2O 7:2:1 v/v): R_f = 0.62.
^{1}H NMR (400 MHz, D_2O): δ 6.86 (s, 2H), 4.84 (d, J = 6.3 Hz, 2H), 3.90 (s, 6H), 3.79 (s, 3H).
^{31}P NMR (162 MHz, D_2O): δ 1.28.
HRMS-ESI: Calculated for $C_{10}H_{14}O_7P$ $[M-H]^-$: 277.04770; Found: 277.0476.

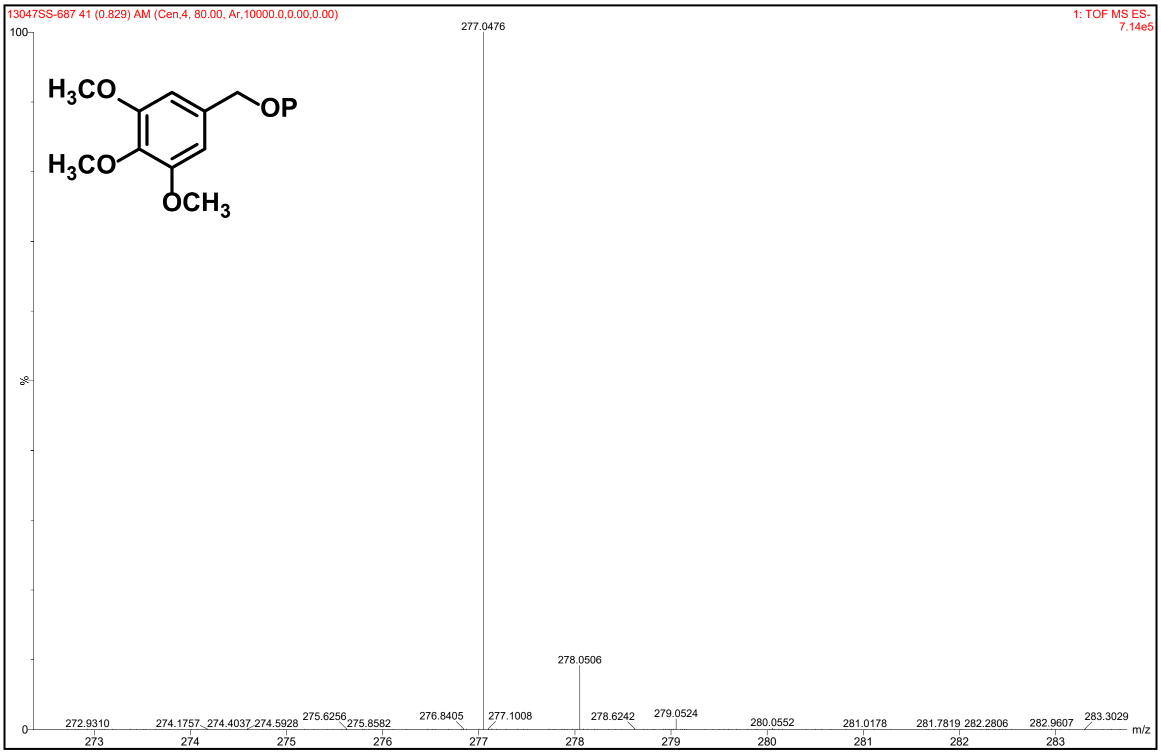

Figure S107. HRMS-ESI$^-$ of **55.**

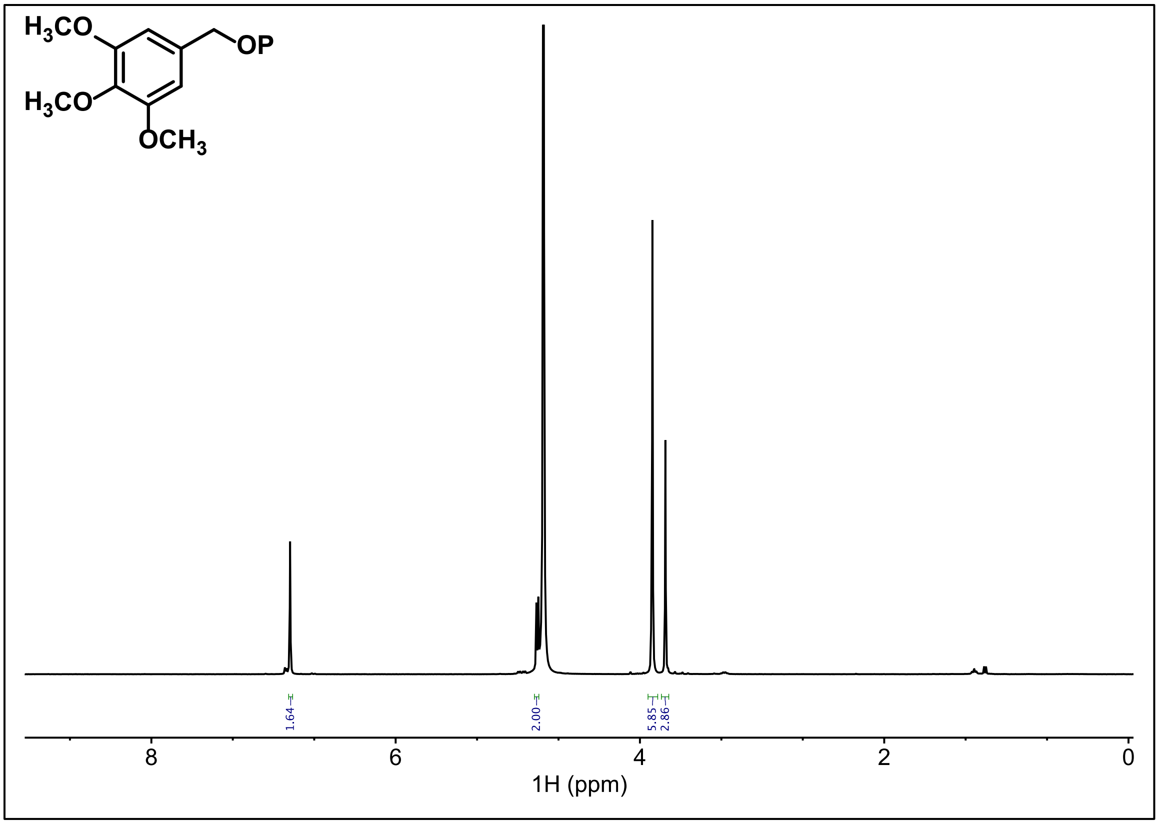

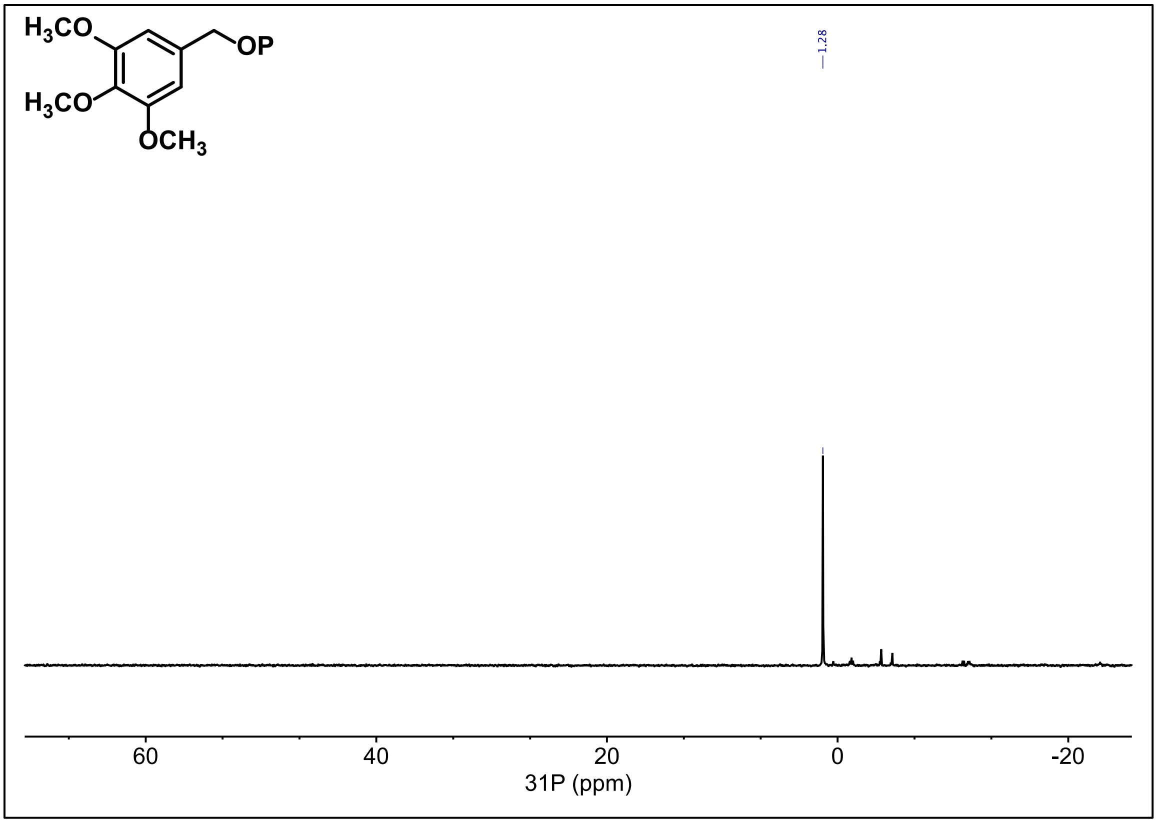

Figure S108. ^{1}H NMR (400 MHz, D_2O) and ^{31}P NMR of **55** (162 MHz, D_2O).

Thiophen-2-ylmethyl Phosphate (56):

The title product obtained as yellow solid from thiophen-2-ylmethanol following the procedure described in *Method 2.7b.*

TLC (^{i}PrOH: NH_4OH: H_2O 7:2:1 v/v): R_f = 0.66.

^{1}H NMR (400 MHz, D_2O): δ 7.32 (d, *J* = 5.1 Hz, 1H), 7.06 (s, 1H), 6.92 (d, *J* = 4.8 Hz, 1H), 5.01 (d, *J* = 6.5 Hz, 2H).

^{31}P NMR (162 MHz, D_2O): δ 1.89.

HRMS-ESI: Calculated for $C_5H_6O_4PS$ $[M-H]^-$: 192.97244; Found:192.9729.

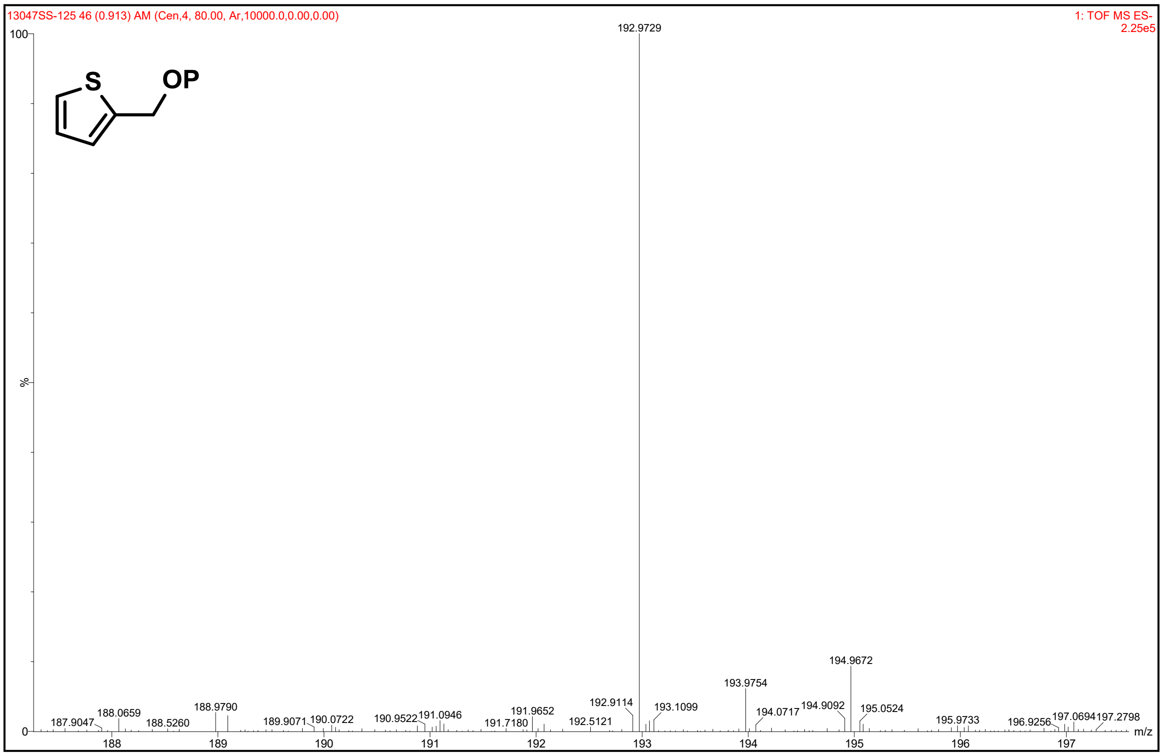

Figure S109. HRMS-ESI$^-$ of **56.**

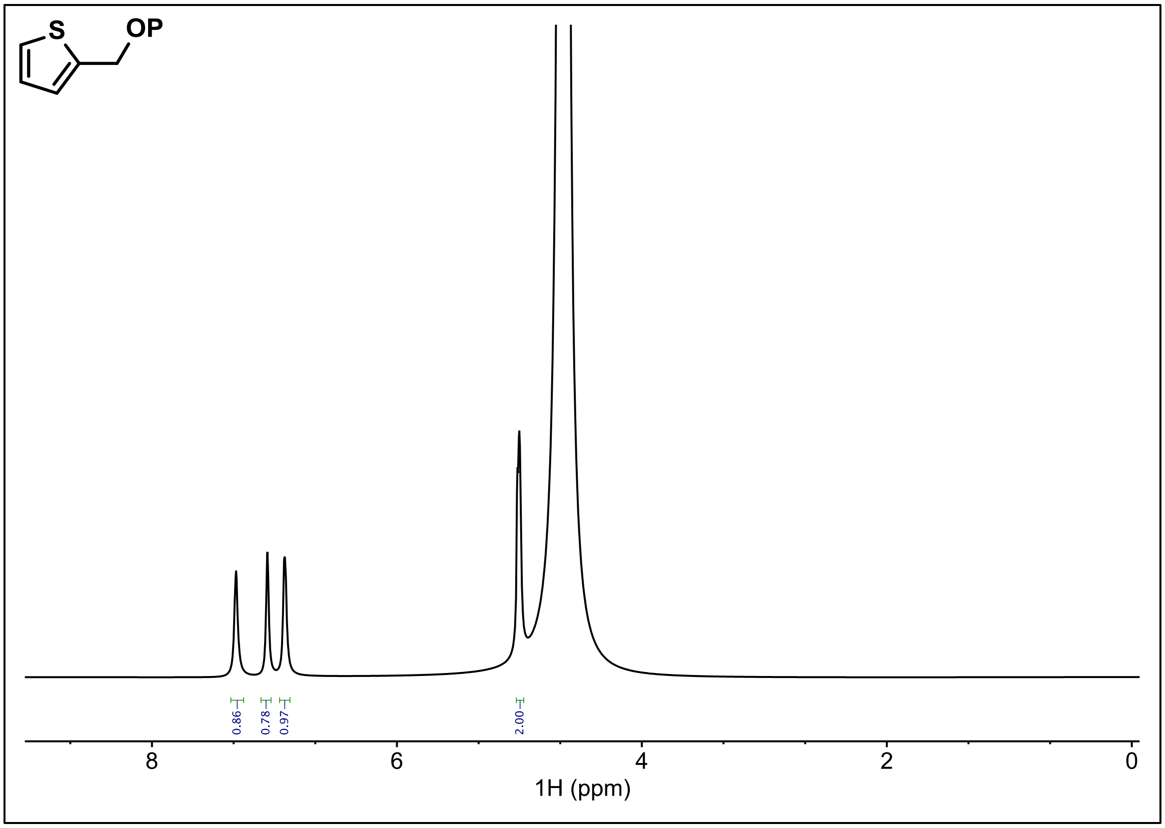

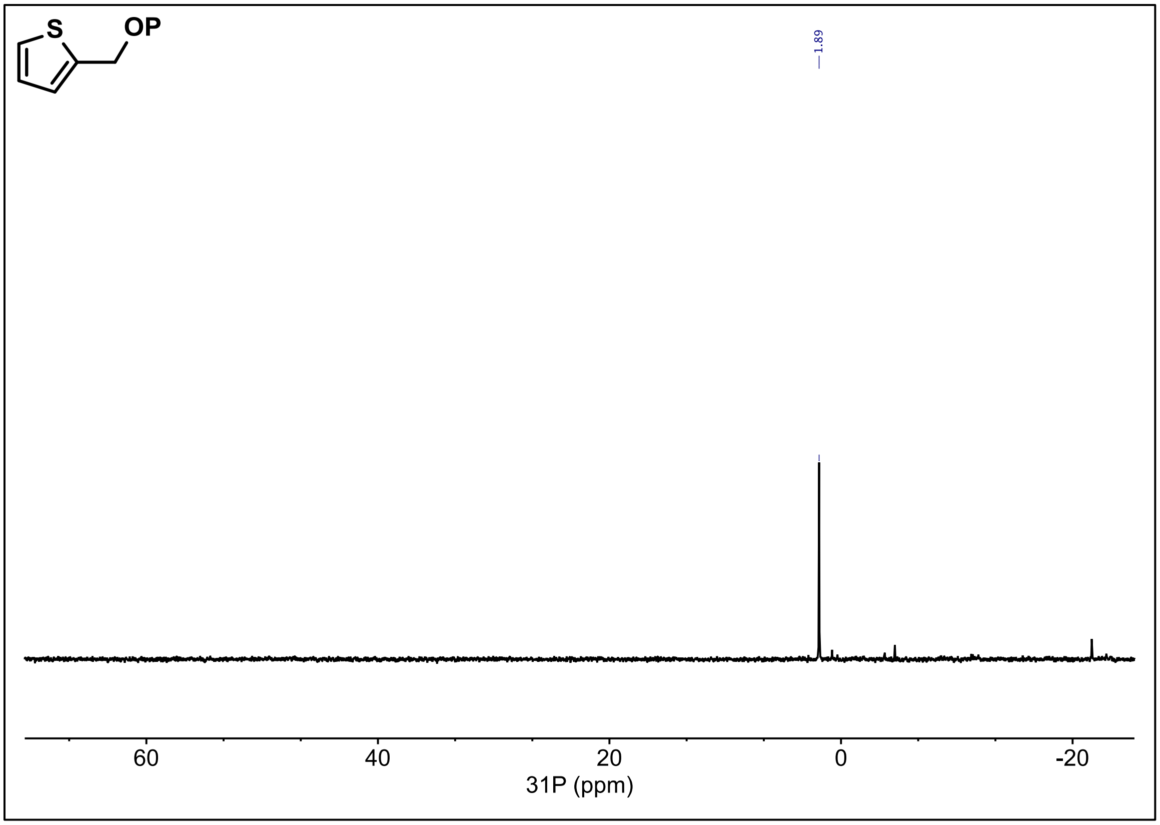

Figure S110. ^{1}H NMR (400 MHz, D_2O) and ^{31}P NMR of **56** (162 MHz, D_2O).

Benzofuran-2-ylmethy Phosphate (57):

The title product was obtained as a white solid from benzofuran-2-ylmethanol following the procedure described in *Method 2.7b.*

TLC (^{i}PrOH: NH_4OH: H_2O 7:2:1 v/v): R_f = 0.65.

^{1}H NMR (300 MHz, D_2O): δ 7.70 – 7.62 (m, 1H), 7.59 – 7.51 (m, 1H), 7.30 (dtd, *J* = 14.8, 7.7, 1.3 Hz, 2H), 6.88 (s, 1H), 4.92 (d, *J* = 6.5 Hz, 2H).

^{31}P NMR (122 MHz, D_2O): δ 2.10.

HRMS-ESI: Calculated for $C_9H_8O_5P$ $[M-H]^-$: 227.01093; Found: 227.0109.

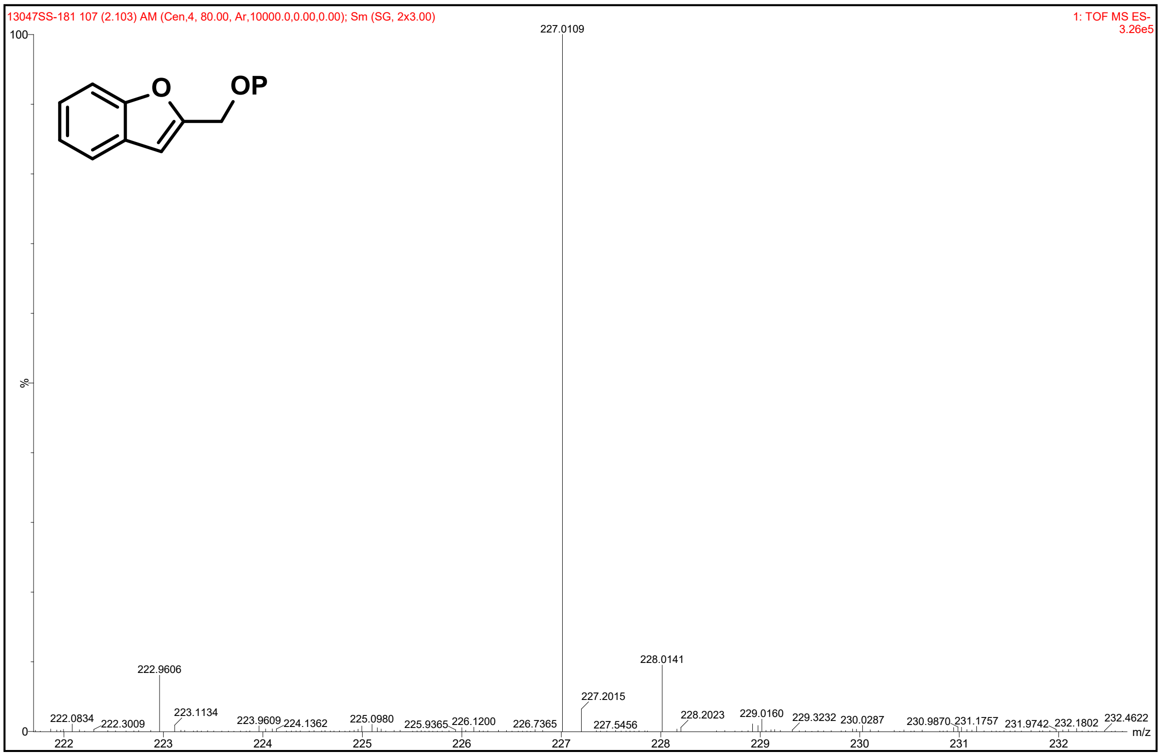

Figure S111. HRMS-ESI$^-$ of **57.**

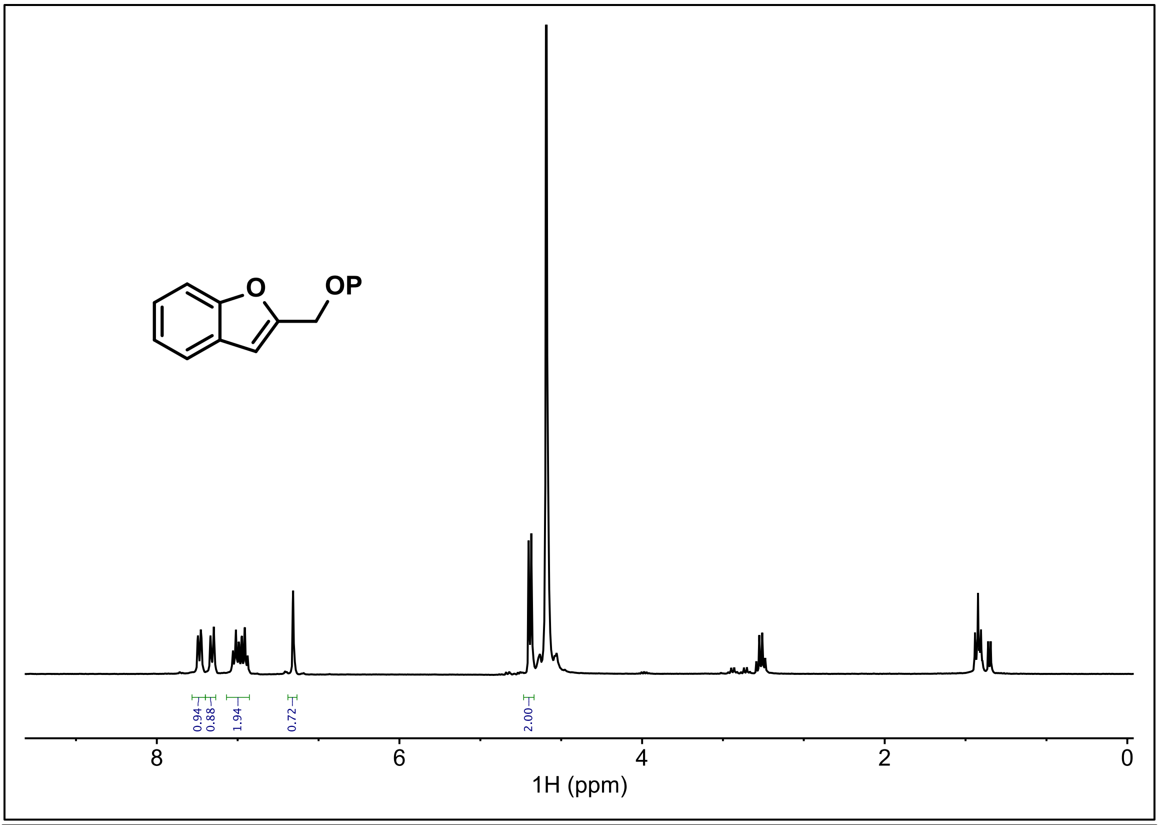

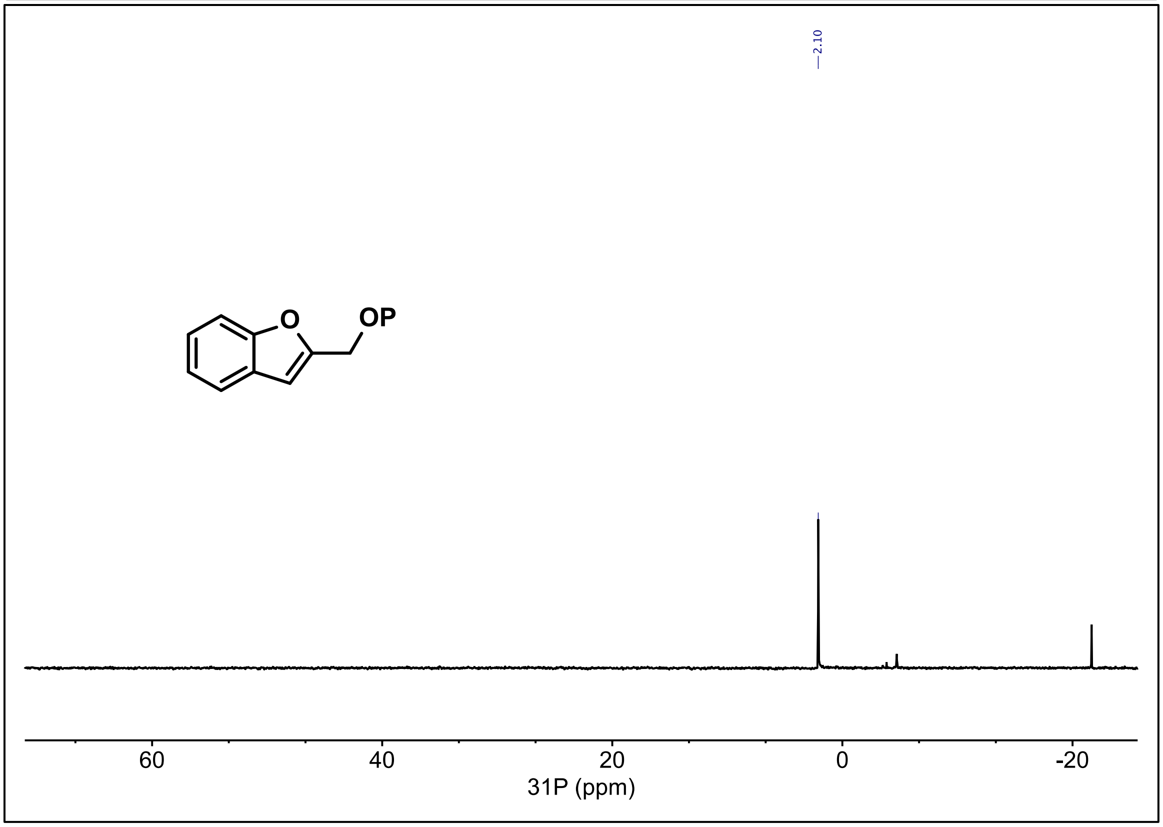

Figure S112. ^{1}H NMR (300 MHz, D_2O) and ^{31}P NMR of **57** (122 MHz, D_2O).

Benzo[*b*]thiophen-3-ylmethyl Phosphate (58):

The title product was obtained as a yellow-white solid from benzo[*b*]thiophen-3-ylmethanol following the procedure described in *Method 2.7b.*

TLC (^{i}PrOH: NH_4OH: H_2O 7:2:1 v/v): R_f = 0.67.

^{1}H NMR (400 MHz, D_2O): δ 5.89 (ddt, *J* = 17.0, 10.3, 6.5 Hz, 1H), 5.51 – 5.38 (m, 1H), 5.24 (t, *J* = 6.7 Hz, 1H), 5.08 (dd, *J* = 17.4, 2.1 Hz, 1H), 5.00 (d, *J* = 10.1 Hz, 1H), 4.40 (t, *J* = 6.7 Hz, 2H), 2.18 (q, *J* = 6.4, 6.0 Hz, 4H), 2.12 (d, *J* = 7.5 Hz, 4H), 1.72 (s, 3H), 1.64 (s, 3H), 1.29 (d, *J* = 4.2 Hz, 3H).

^{31}P NMR (162 MHz, D_2O): δ 0.96.

HRMS-ESI: Calculated for $C_9H_8O_4PS$ $[M-H]^-$: 242.98808; Found: 242.9887.

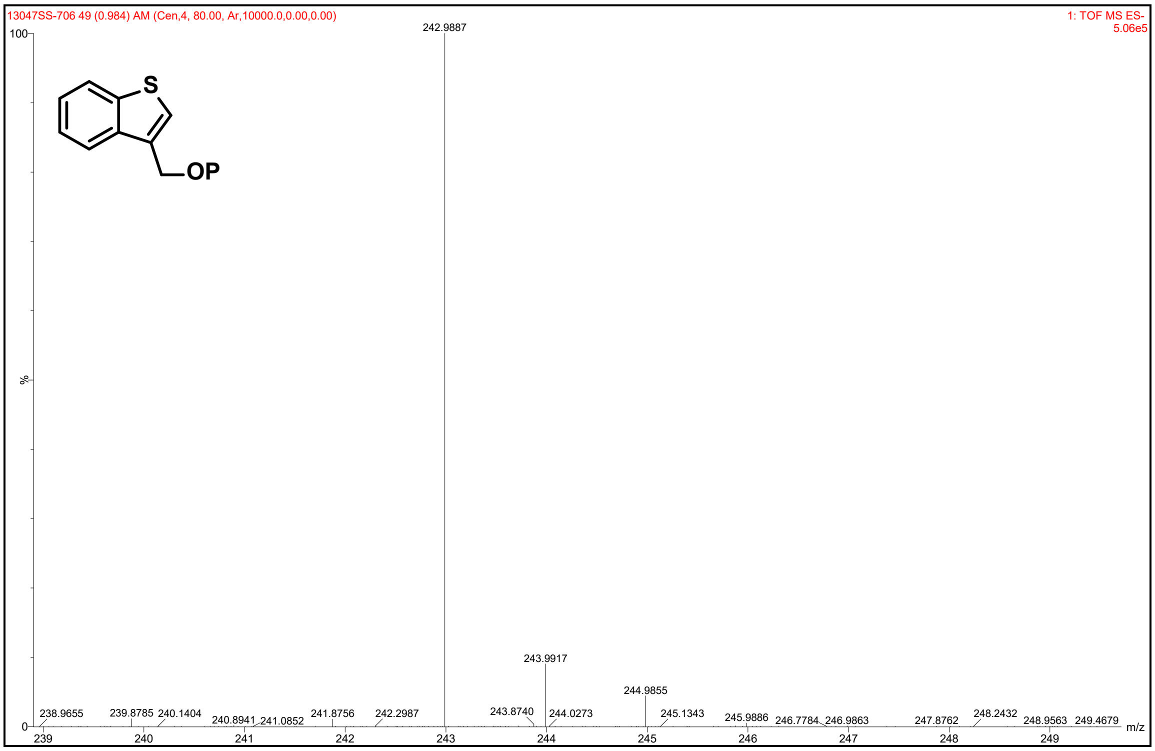

Figure S113. HRMS-ESI$^-$ of **58.**

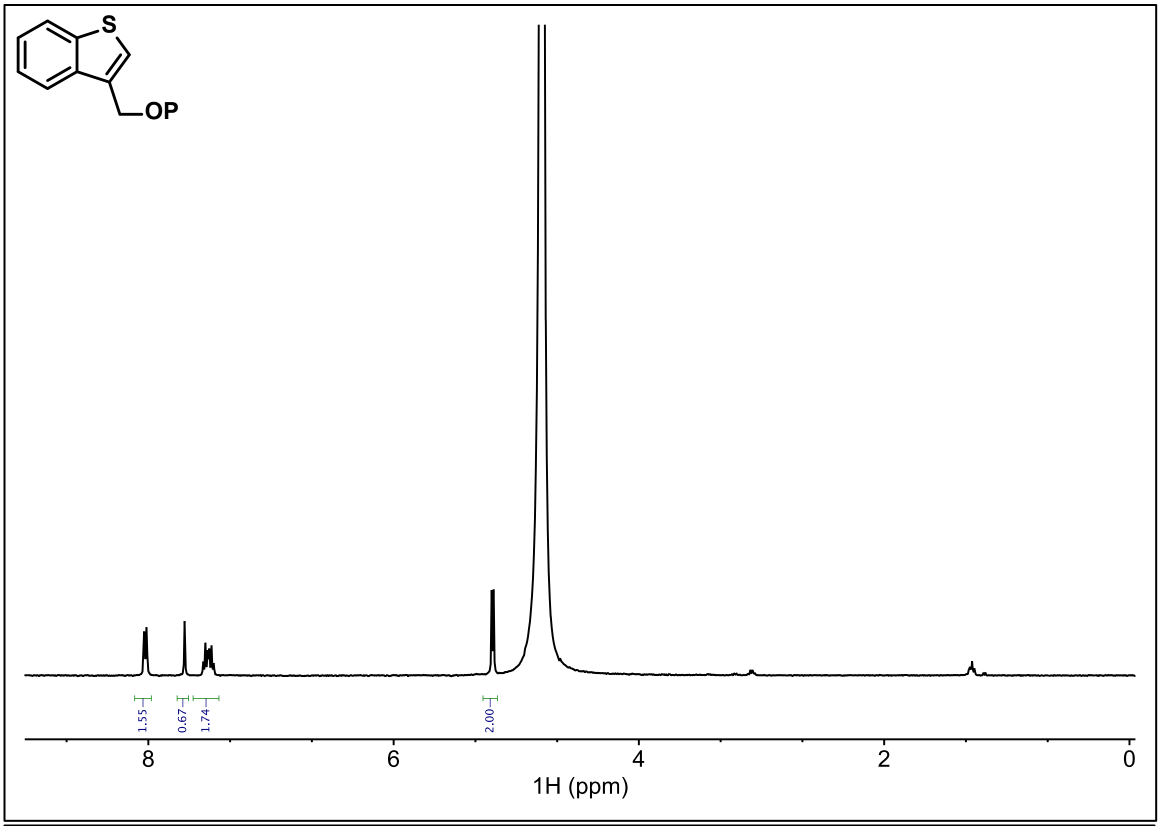

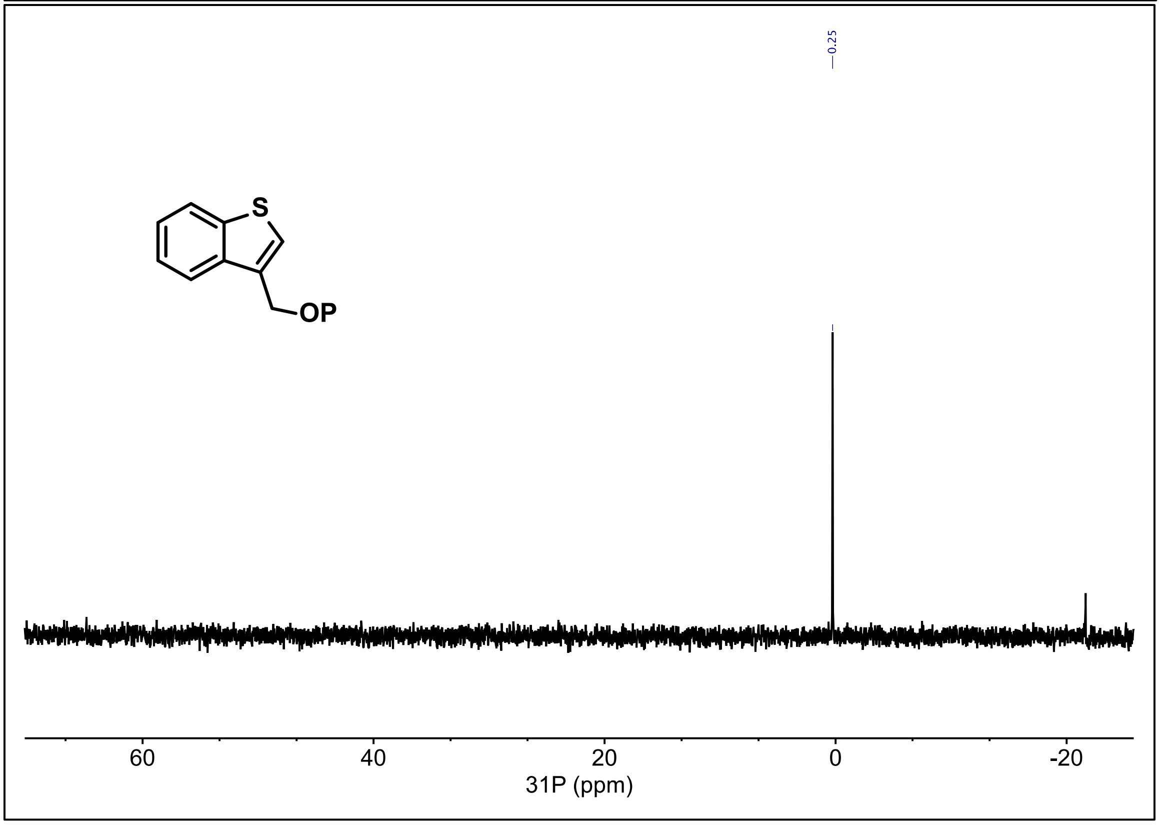

Figure S114. ^{1}H NMR (400 MHz, D_2O) and ^{31}P NMR of **58** (162 MHz, D_2O).

Table S3. Codon-Optimized Genes (CMA, CNG) Used in This Study

cmaIPK	**cat**atgatcctgattaagctgggtggcagcgttatcaccgataaaagcgagtaccacaagtt<br>caacaaagaaaccgtgagccgtctggcggatgagattcgtcgtagcggtcaggacgtgatgg<br>tggttcacggtgcgggcagctttggccacgttatcgcgaagaagtacgcgatccaggatggt<br>cacgttgatgatggtcaaatcccggcggcggcgcgtattatgtgcgacacccgtgagctgag<br>cagcatggtggttgaggaactgctggcgcagggtatcccggctgtgagcgttgcgccgggta<br>gctgcttcgtgatggaagatggcaagctgatcgttgacaacgaggaaccgattcgtcgtctg<br>gcggatctgggtatcatgccggttatgttcggcgacgtggttccggatcgtaagaaaggttt<br>tgcgattgtgagcggcgatcagtgcatggaagtgctgtgccgtatgttcgacccggaaaagg<br>tggttttgttagcgacattgatggtctgtacaccgcggacccgaaaaccgataagaaagcg<br>cgtctgatcggcgaggtgacccgtaagaaactggacgaagcgctgaccgatattaccgtggc<br>ggacgttaccggtggcgttcacagcaaaatggaagcgatgctgcgtatgaccgaccgtaacc<br>gtcgttgctacctggtgaacggtaacgcgccgaaccgtctgtatagcctgctgaagggcgag<br>accgtgacctgcaccgttgcgaaaggtggcatggaataa**gaattc**
cngIPK	**cat**atgcagaagctggcgctgatcaaactgggtggcagcgtggttaccttcaaagacaaacc<br>gctggcggcgaacaccggtgcgattgatggtatcagccgtgtgctggcgcaactgagcaccc<br>cggcgatcattgttcacggtggcggtagctttggccactactggagcgtgaagtatgacatg<br>cacaccaaaccggcgggctacgatccgcacggtgttagcgtggttcacgaaagcatgattgc<br>gctgaaccagatcattatcaacagcatgatccgtgcgggtctgaacccgtacggtatgccgc<br>cgagcgtgtttgcggcgggtcacaagccggttgcggcgaagattaaacaaatctataccatg<br>gcgaaaagcgaagtgatgccggttacctttggcgacgtggttcacatggagggtaacaagta<br>cagcattctgagcggcgatgcgctgatgaccatgctggcgaaagtgctgcgtccgagccgtg<br>tggttttcgcgaccaacgttgacggtatctataaggacatggcgagccgtgagctgatgcgt<br>gagatccgtgtgaccaccggccgtaagaaacgtagcatcgagttcagcaaggcgagcggtgc<br>ggatgtgaccggcggtatgcagcgtaaggttgcggaggcgtttaaaattgcgagccgtggca<br>tggatgtgctgatggttaacggtctgatgccggagcgtatcgcggaagcggtggttgagggt<br>acccgtctgcaagttggcaccgtggttaagggtagccgtcgttaa**gaattc**
Nucleotides in bolded blue were added to insert restriction sites for cloning (N: *NdeI*, C: *EcoRI*).	

Table S4. Codon-Optimized Genes (MHM, MSB, TCP) Used in This Study

mhmIPK	**cat**atgaacgagaagaacagcgacatcaccattctgaaaatcggtggcagcgtgattaccga<br>taagagcagcaacgagggtattgttcgtgaaaaagcgatcattcgtatcgcgcgtgagatta<br>gcttctttgaaggtccgctgatcattgttcatggtgcgggtagctttggtcacccgcaagcg<br>aagcgttacagcctggcgtataagttcagcgcggagggtagcggcgtgacccaccgtagcgt<br>tgcgagcctgaacgaactgttcgtgaacgcgctggcgcagcgtaacgtgaacgcggttggta<br>tccacccgatgggctgcctggttgcgaaagagggtcgtatcagcaaaagctttctgctggcg<br>attcaaatgatgctggaaaagggtatcaccccggtgctgcacggcgacgtggttatggatac<br>cgtgaaaggtagcagcgttatcagcggcgaccagattgttccgtacctggcgagcaagctga<br>acgcgtatcgtattggtgtgggcagcgcggaggacggtgttctggatgaacacggccacccg<br>atcccgctgattaccccggataacttcgagatcgcgagcatgcacattggtggcagcgaaaa<br>caccgacgtgaccggtggcatgctgggcaaggttaaagagatgctggacatgagccgtcaga<br>ccaaagtgccgagctacatcttcaacgcgaacgcgggtggcaacgttatgagctttctgcgt<br>ggtgaaatgatcggcaccgcgattaaggacaaagcgtaa**gaattc**
msbIPK	**cat**atgaacgtgagcaccgagccggttatcctgaagctgggtggcagcgtgattaccgacaa<br>agcggcggatcagggtgtggttcgtgaagacagcctgcgtcgtatcgcgaaagaggtgtctg<br>aataccgtggtaaaatgatcattgttcatggtgcgggtagctttggtcacacctacgcgaag<br>aaatatcaactgggcaaggtttttgacccggagggcgcgatcgtgacccacgaaagcgttaa<br>gaaactggcgagccgtgtggttgataccctgaacgagtacggtgtgcgtgcgatcgcggttc<br>acccgatgtgctgcaccatctgccgtaacggccgtattgagagcatgtatctggataacatt<br>aaactgatgctggaaaacggtctggtgccggttctgcacggcgacgtggttatggatctgga<br>actgcgtgcgtgcgtgctgagcggtgaccagatcgttccgtacctggcgaaggagctgaaaa<br>ttacccgtctgggtctgggcagcgcggaagatggtgtgctggacaacgatggcaagaccgtt<br>ccggagatcaccccgaagaccttcgaagactttaaacactatattcgtggtagcggcagcac<br>cgatgtgaccggtggcatgctgggcaaggttcaagagctgctggaactgagcaaaaccagct<br>gcatcaccagctacattttcaacgcgggcaaggacgataacatctatcgttttctgaacggc<br>gagccgatgggcaccaccattagcccggacaaacgtgtgtaa**gaattc**
tcpIPK	**Cat**atgatcattatcaaactgggtggcagcgtgatcagcgacaaggagaaagaatacagctt<br>ccaccgtcacattgttgagcagatcgcggaggaaattgcgcagttctatccggatgaaagct<br>ttatcctggtgcatggtggcggtagctttggtcacccgaacgcgcgtgagtacaagatcacc<br>gaaggcctggtgggtgacgttgatcgtaaacgtattggttttagcaagacccaccaggcgat<br>gctgaaactgaacgacctgattatccaaaccttcctggagaagggcctgccggcgtacagcg<br>ttagcagcagcagcatctttctgctggagaacaaagaagtggtttatggcgagctggagatc<br>ctgcgtaaactgctggaactgaagttcatcccggtgctgtttggtgacaccgcgattgcgct<br>ggacaagggcattgatatcctgagcggtgatcagatcgttagctacctggcgaagatgctga<br>aaccgagcaaggtgatcttcctgatggacgttgatggtatttatgaccgtaacccgaaagag<br>cgtgatgcgaagctgatcgaggaactgaacgtggaggagatccgtcacctgctggagagcag<br>cgaaagcgcgggcatcgatgttaccggcggtattggtaacaaactgcgtgaggcgctgaaaa<br>tcgcgaagcacagcgaagtgtattttattaacggcaaggttaaagagaacctgggtaaagcg<br>atccgtggcgaaaaagtgggtacccgtctgcgtaaactggaacacccgaagattagctaa**ga<br>attc**

Nucleotides in bolded blue were added to insert restriction sites for cloning (N: *NdeI*, C: *EcoRI*).

Table S5. Amino Acid Sequences of the IPK Homologs Utilized in This Study

CMA	**MGSSHHHHHHSSGLVPRGSH**MILIKLGGSVITDKSEYHKFNKETVSRLADEIRRSGQD<br>VMVVHGAGSFGHVIAKKYAIQDGHVDDGQIPAAARIMCDTRELSSMVVEELLAQGIPA<br>VSVAPGSCFVMEDGKLIVDNEEPIRRLADLGIMPVMFGDVVPDRKKGFAIVSGDQCME<br>VLCRMFDPEKVVFVSDIDGLYTADPKTDKKARLIGEVTRKKLDEALTDITVADVTGGV<br>HSKMEAMLRMTDRNRRCYLVNGNAPNRLYSLLKGETVTCTVAKGGME
CNG	**MGSSHHHHHHSSGLVPRGSH**MQKLALIKLGGSVVTFKDKPLAANTGAIDGISRVLAQL<br>STPAIIVHGGGSFGHYWSVKYDMHTKPAGYDPHGVSVVHESMIALNQIIINSMIRAGL<br>NPYGMPPSVFAAGHKPVAAKIKQIYTMAKSEVMPVTFGDVVHMEGNKYSILSGDALMT<br>MLAKVLRPSRVVFATNVDGIYKDMASRELMREIRVTTGRKKRSIEFSKASGADVTGGM<br>QRKVAEAFKIASRGMDVLMVNGLMPERIAEAVVEGTRLQVGTVVKGSRR
MHM	**MGSSHHHHHHSSGLVPRGSH**MNEKNSDITILKIGGSVITDKSSNEGIVREKAIIRIAR<br>EISFFEGPLIIVHGAGSFGHPQAKRYSLAYKFSAEGSGVTHRSVASLNELFVNALAQR<br>NVNAVGIHPMGCLVAKEGRISKSFLLAIQMMLEKGITPVLHGDVVMDTVKGSSVISGD<br>QIVPYLASKLNAYRIGVGSAEDGVLDEHGHPIPLITPDNFEIASMHIGGSENTDVTGG<br>MLGKVKEMLDMSRQTKVPSYIFNANAGGNVMSFLRGEMIGTAIKDKA
MSB	**MGSSHHHHHHSSGLVPRGSH**MNVSTEPVILKLGGSVITDKAADQGVVREDSLRRIAKE<br>VSEYRGKMIIVHGAGSFGHTYAKKYQLGKVFDPEGAIVTHESVKKLASRVVDTLNEYG<br>VRAIAVHPMCCTICRNGRIESMYLDNIKLMLENGLVPVLHGDVVMDLELRACVLSGDQ<br>IVPYLAKELKITRLGLGSAEDGVLDNDGKTVPEITPKTFEDFKHYIRGSGSTDVTGGM<br>LGKVQELLELSKTSCITSYIFNAGKDDNIYRFLNGEPMGTTISPDKRV
TCP	**MGSSHHHHHHSSGLVPRGSH**MIIIKLGGSVISDKEKEYSFHRHIVEQIAEEIAQFYPD<br>ESFILVHGGGSFGHPNAREYKITEGLVGDVDRKRIGFSKTHQAMLKLNDLIIQTFLEK<br>GLPAYSVSSSSIFLLENKEVVYGELEILRKLLELKFIPVLFGDTAIALDKGIDILSGD<br>QIVSYLAKMLKPSKVIFLMDVDGIYDRNPKERDAKLIEELNVEEIRHLLESSESAGID<br>VTGGIGNKLREALKIAKHSEVYFINGKVKENLGKAIRGEKVGTRLRKLEHPKIS
Bolded residues indicate the His_6-tag inserted by the pET28a vector.	

A

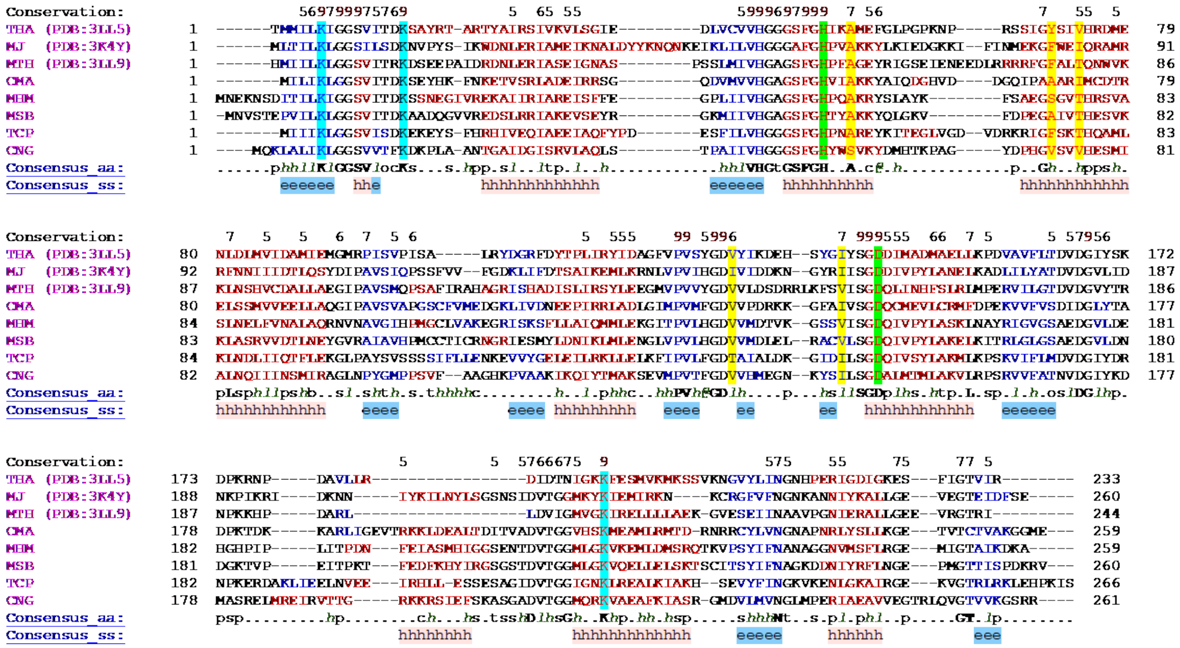

B

	MJ	TCP	CMA	MTH	MSB	MHM	THA	CNG
**MJ**	100	36	29.39	37.94	35.92	36.59	28.15	27.35
**TCP**	36	100	35.04	37.98	32.26	30.08	34.44	31.75
**CMA**	29.39	35.04	100	34.11	28.8	30.12	33.19	26.91
**MTH**	37.94	37.98	34.11	100	39.37	38.98	31.02	27.84
**MSB**	35.92	32.26	28.8	39.37	100	51.55	30.38	25.31
**MHM**	36.59	30.08	30.12	38.98	51.55	100	27.85	26.64
**THA**	28.15	34.44	33.19	31.02	30.38	27.85	100	32.39
**CNG**	27.35	31.75	26.91	27.84	25.31	26.64	32.39	100

Figure S115. Sequence Alignment and Identity Matrix of IPK Homologs. Amino acid sequences of the IPKs utilized in the current study were aligned with homologs whose structures have been solved previously[1] using PROMALS3D's default parameters (**A**).[2] Sequence identity between all eight homologs (**B**) was calculated using Clustal X version 2.1.[3]

Table S6. NADH Conversion Data for WT IPK Homologs.

**Alkyl-P**	**IPK Homolog**				
	**CMA**	**CNG**	**MHM**	**MSB**	**TCP**
**1**	99.815 ± 0.007	100	100	86 ± 7	100
**2**	97.5 ± 0.4	100	100	100	100
**3**	89 ± 7	(8 ± 1) x 10^1	35 ± 6	79 ± 4	(1.0 ± 0.2) x 10^1
**4**	(2 ± 1) x 10^1	ND	6 ± 3	ND	ND
**5**	100	(5.0 ± 0.7) x 10^1	(3 ± 1) x 10^1	53 ± 9	47 ± 4
**6**	100	100	88 ± 8	82 ± 7	100
**7**	100	100	100	100	100
**8**	100	78 ± 1	100	(9.0 ± 0.9) x 10^1	100
**9**	100	100	100	100	100
**10**	100	16 ± 2	21 ± 1	65 ± 3	19 ± 4
**11**	100	79 ± 5	100	100	73 ± 2
**12**	ND	ND	13.0 ± 0.4	72 ± 7	27 ± 3
**13**	96.7 ± 0.3	46.8 ± 0.5	100	(8 ± 2) x 10^1	100
**14**	100	100	15 ± 3	96 ± 3	94 ± 3
**15**	100	6.9 ± 0.7	69 ± 7	61 ± 6	(6 ± 2) x 10^1
**16**	100	94 ± 4	57.2 ± 0.2	100	52.9 ± 0.8
**17**	(6 ± 1) x 10^1	(1.0 ± 0.2) x 10^1	78.1 ± 0.9	55 ± 8	ND
**18**	100	ND	(7 ± 2) x 10^1	69 ± 4	6.6 ± 0.6
**19**	12 ± 5	ND	12 ± 1	17 ± 7	ND
**23**	55 ± 6	(2.0 ± 0.1) x 10^1	31.52 ± 0.08	77 ± 4	7 ± 2
**24**	ND	ND	23.4 ± 0.8	29 ± 5	ND
**26**	ND	ND	70.3 ± 0.6	38.0 ± 0.2	ND
**27**	6 ± 2	4.8 ± 0.6	ND	11.1 ± 0.4	ND
**28**	ND	ND	34 ± 2	63.95 ± 0.03	ND
**29**	ND	ND	19 ± 8	26.3 ± 0.5	ND
**30**	ND	ND	21 ± 6	ND	ND
**32**	(6 ± 1) x 10^1	5 ± 1	27 ± 9	57 ± 2	ND
**34**	ND	ND	ND	6 ± 1	6 ± 1
**35**	ND	ND	ND	3.4 ± 0.4	ND
**36**	ND	ND	19.4 ± 0.8	30.9 ± 1.0	ND
**46**	98.7 ± 0.5	100	68 ± 5	100	ND
**47**	100	16 ± 2	52 ± 6	100	17.1 ± 0.5
**48**	35 ± 2	71 ± 1	ND	38 ± 1	14 ± 6
**49**	8 ± 1	ND	ND	12 ± 2	ND
**50**	ND	ND	29.5 ± 0.4	ND	18.5 ± 0.3
**54**	ND	ND	39.6 ± 0.9	100	ND
**56**	100	44 ± 4	88 ± 3	100	41 ± 3
ND = Either no reaction or lower than the detection limit of the instrument.					

Table S7. HRMS Confirmation of IPK-Catalyzed Reactions

Product	Chemical Formula	Predicted Mass (Da)	Observed Mass (Da)	Error (ppm)
**1**-OPP	$C_5H_{11}O_7P_2$ [M-H]$^-$	244.99799	244.9990	4.1
**2**-OPP	$C_5H_{11}O_7P_2$ [M-H]$^-$	244.99799	244.9983	1.3
**3**-OPP	$C_6H_{13}O_7P_2$ [M-H]$^-$	259.01364	259.0143	2.5
**4**-OPP	$C_3H_5O_7P_2$ [M-H]$^-$	214.95104	214.9517	3.1
**5**-OPP	$C_4H_{11}O_7P_2$ [M-H]$^-$	232.99799	232.9990	4.3
**6**-OPP	$C_4H_{11}O_7P_2$ [M-H]$^-$	232.99799	232.9987	3.0
**7**-OPP	$C_4H_9O_7P_2$ [M-H]$^-$	230.98234	230.9831	3.3
**8**-OPP	$C_4H_7O_7P_2$ [M-H]$^-$	228.96669	228.9669	0.92
**9**-OPP	$C_5H_{13}O_7P_2$ [M-H]$^-$	247.01364	247.0147	4.3
**10**-OPP	$C_3H_8N_3O_7P_2$ [M-H]$^-$	259.98374	259.9841	1.4
**11**-OPP	$C_5H_{13}O_7P_2$ [M-H]$^-$	247.01364	247.0146	3.9
**12**-OPP	$C_3H_7O_7P_2$ [M-H]$^-$	216.96669	216.9667	0.046
**13**-OPP	$C_4H_9O_7P_2$ [M-H]$^-$	230.98234	230.9830	2.9
**14**-OPP	$C_4H_8ClO_7P_2$ [M-H]$^-$	264.94337	264.9421	4.8
**15**-OPP	$C_5H_7O_7P_2$ [M-H]$^-$	240.96669	240.9676	3.8
**16**-OPP	$C_6H_{13}O_7P_2$ [M-H]$^-$	259.01364	259.0132	1.7
**17**-OPP	$C_7H_{15}O_7P_2$ [M-H]$^-$	273.02929	273.0291	0.70
**18**-OPP	$C_7H_{13}O_7P_2$ [M-H]$^-$	271.01364	271.0147	3.9
**19**-OPP	$C_8H_{15}O_7P_2$ [M-H]$^-$	285.02929	285.0291	0.67
**23**-OPP	$C_7H_{15}O_7P_2$ [M-H]$^-$	273.02929	273.0297	1.5
**24**-OPP	$C_8H_{17}O_7P_2$ [M-H]$^-$	287.04494	287.0447	0.84
**26**-OPP	$C_{10}H_{13}O_7P_2$ [M-H]$^-$	307.01364	307.0139	0.85
**27**-OPP	$C_8H_{17}O_7P_2$ [M-H]$^-$	287.04494	287.0443	2.2
**28**-OPP	$C_8H_{15}O_7P_2$ [M-H]$^-$	285.02929	285.0299	2.1
**29**-OPP	$C_8H_{13}O_7P_2$ [M-H]$^-$	283.01364	283.0150	4.8
**30**-OPP	$C_9H_{19}O_7P_2$ [M-H]$^-$	301.06059	301.0600	2.0
**32**-OPP	$C_4H_8N_3O_7P_2$ [M-H]$^-$	271.98374	271.9848	3.9
**34**-OPP	$C_9H_{19}O_7P_2$ [M-H]$^-$	301.06059	301.0596	3.3
**35**-OPP	$C_8H_{13}O_8P_2$ [M-H]$^-$	299.00856	299.0095	3.1
**36**-OPP	$C_6H_{12}N_3O_7P_2$ [M-H]$^-$	300.01504	300.0166	5.2*
**46**-OPP	$C_7H_9O_7P_2$ [M-H]$^-$	266.98234	266.9823	0.15
**47**-OPP	$C_7H_8FO_7P_2$ [M-H]$^-$	284.97292	284.9719	3.6
**48**-OPP	$C_7H_7F_2O_7P_2$ [M-H]$^-$	302.96350	302.9622	4.3
**49**-OPP	$C_7H_8ClO_7P_2$ [M-H]$^-$	300.94337	300.9429	1.6
**50**-OPP	$C_8H_{11}O_7P_2$ [M-H]$^-$	280.99799	280.9973	2.5
**52**-OPP	$C_8H_{11}O_8P_2$ [M-H]$^-$	296.99291	296.9930	0.30
**54**-OPP	$C_8H_9O_9P_2$ [M-H]$^-$	310.97217	310.9716	1.8
**56**-OPP	$C_5H_7O_7P_2S$ [M-H]$^-$	272.93877	272.9395	2.7
*Error too high to be considered "high resolution"				

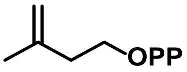

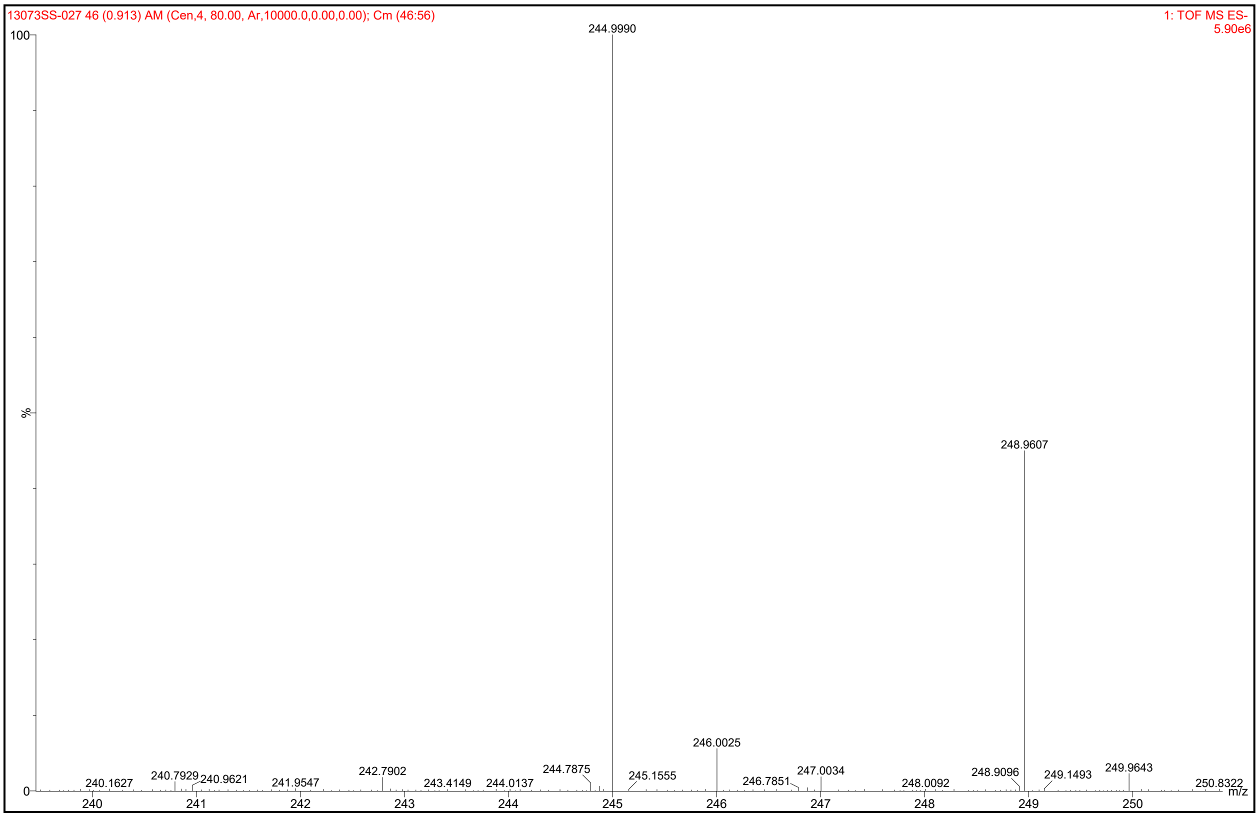

Figure S116. HRMS-ESI$^-$ of **1**-OPP.

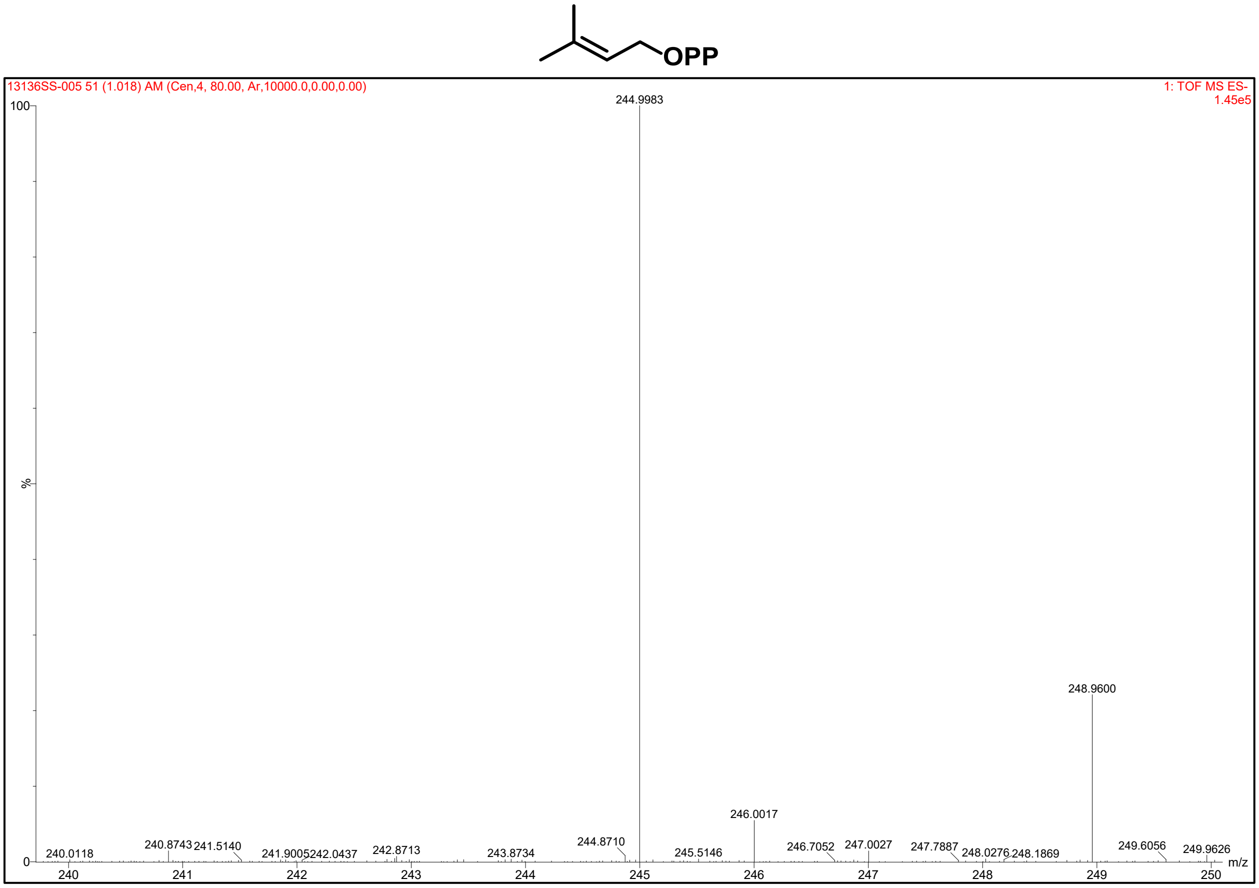

Figure S117. HRMS-ESI$^-$ of **2**-OPP.

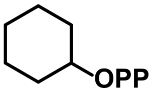

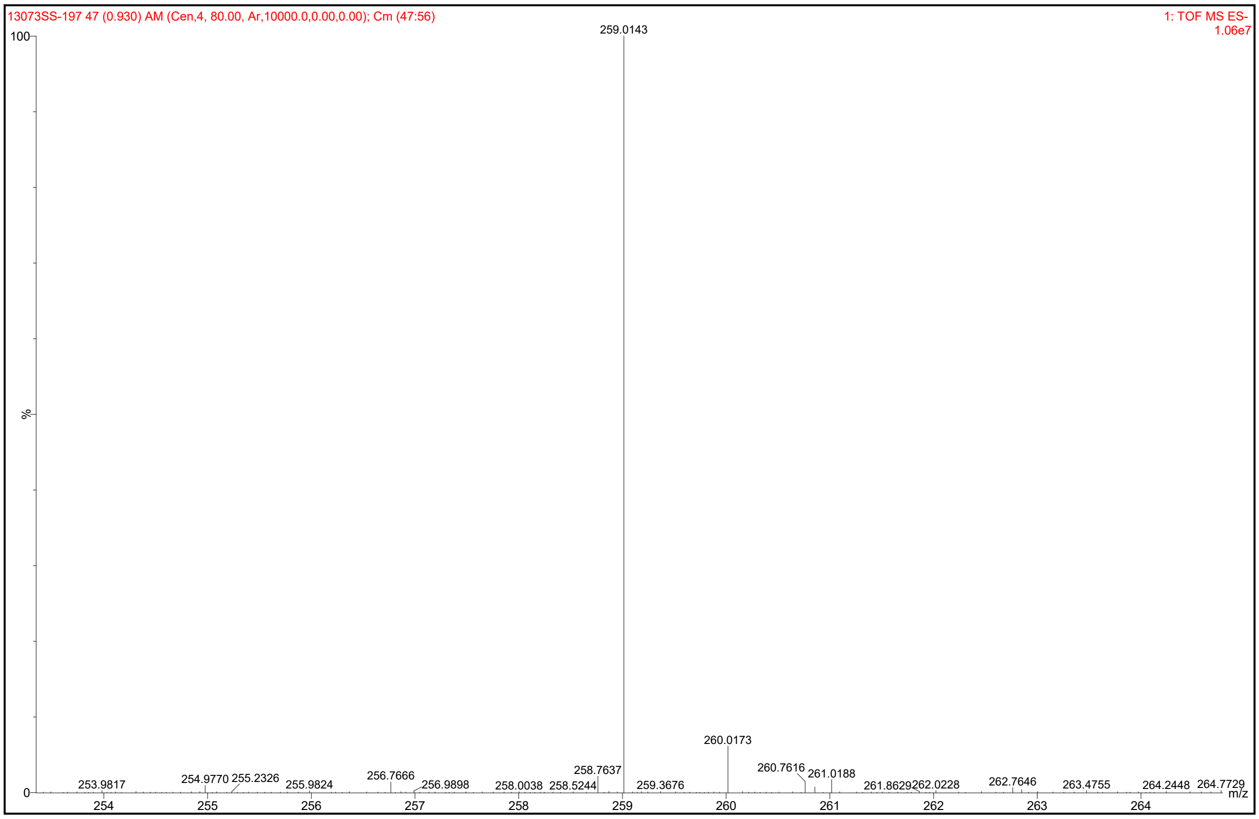

Figure S118. HRMS-ESI$^-$ of **3**-OPP.

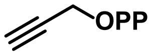

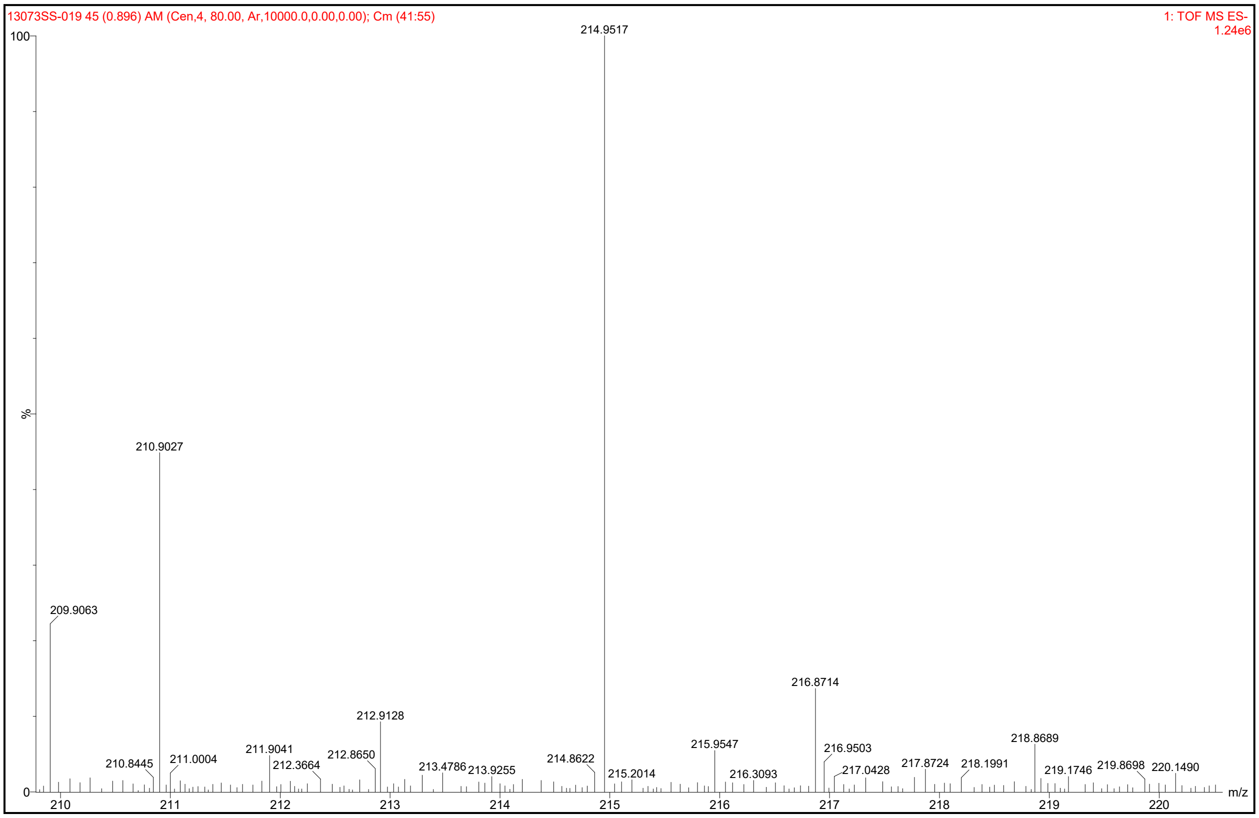

Figure S119. HRMS-ESI$^-$ of **4**-OPP.

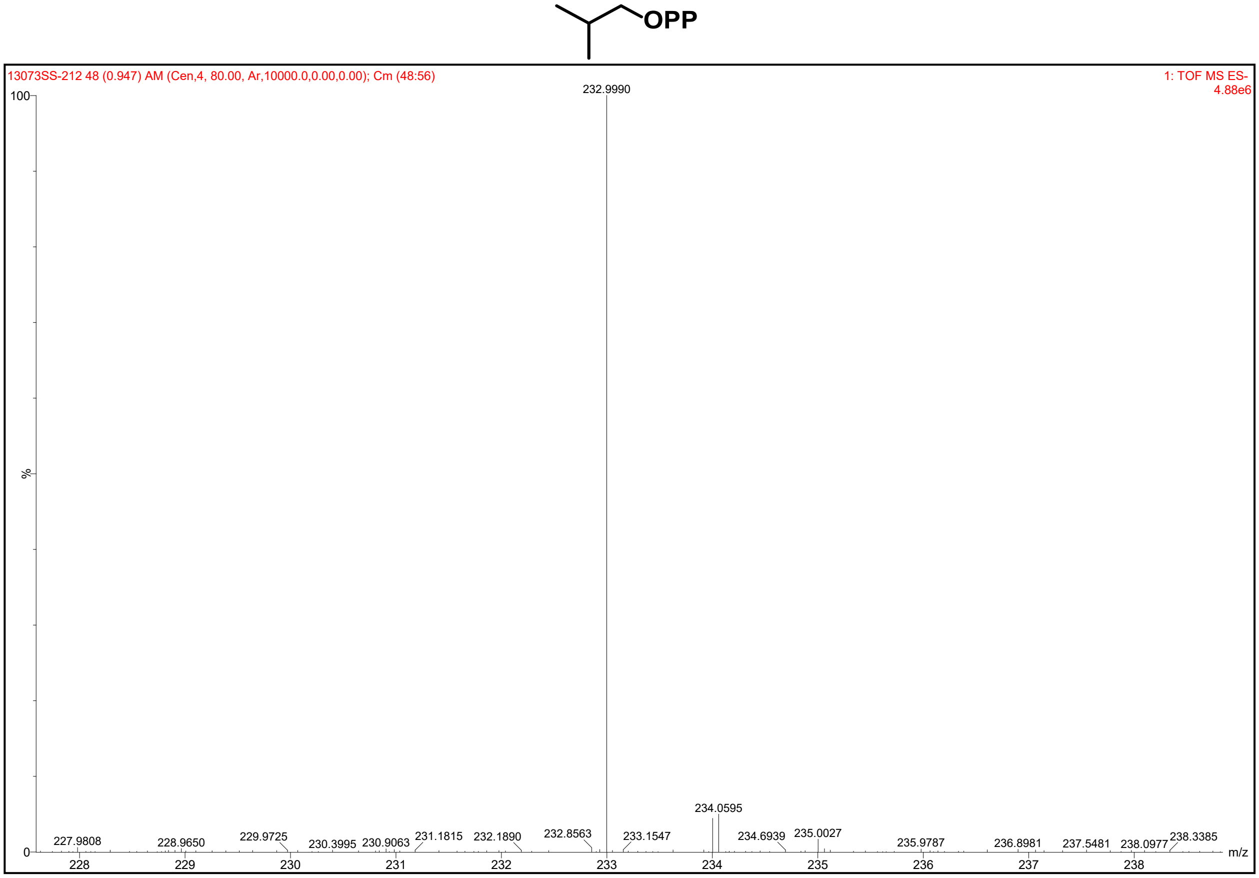

Figure S120. HRMS-ESI⁻ of **5**-OPP.

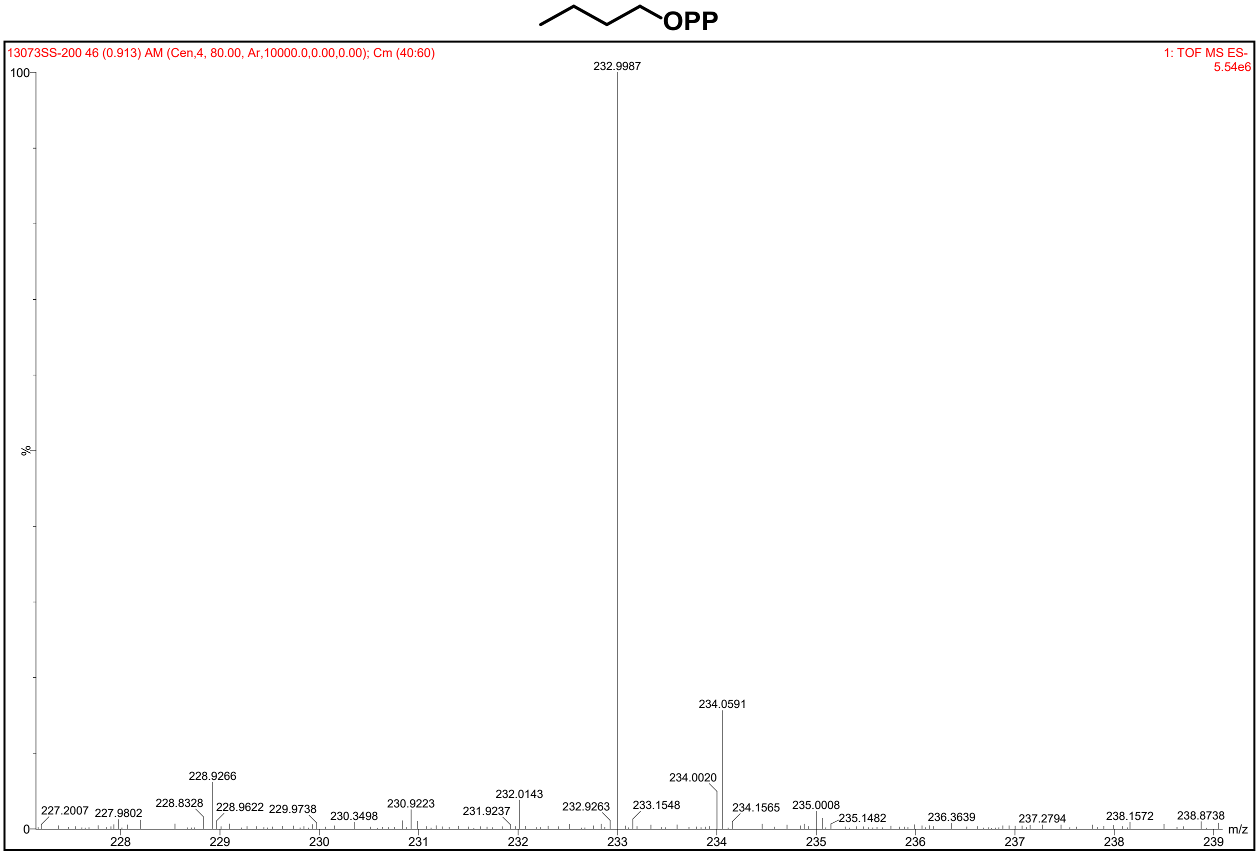

Figure S121. HRMS-ESI- of **6**-OPP.

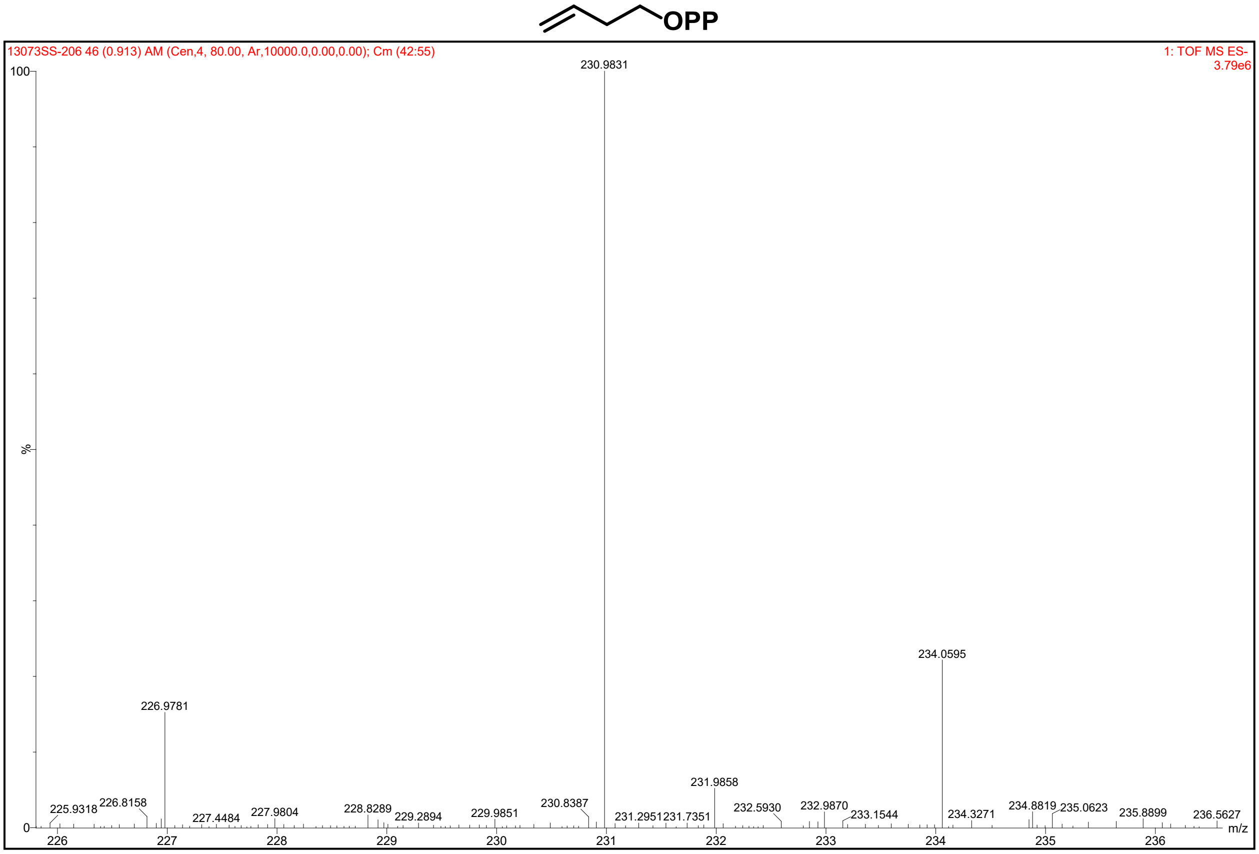

Figure S122. HRMS-ESI⁻ of **7**-OPP.

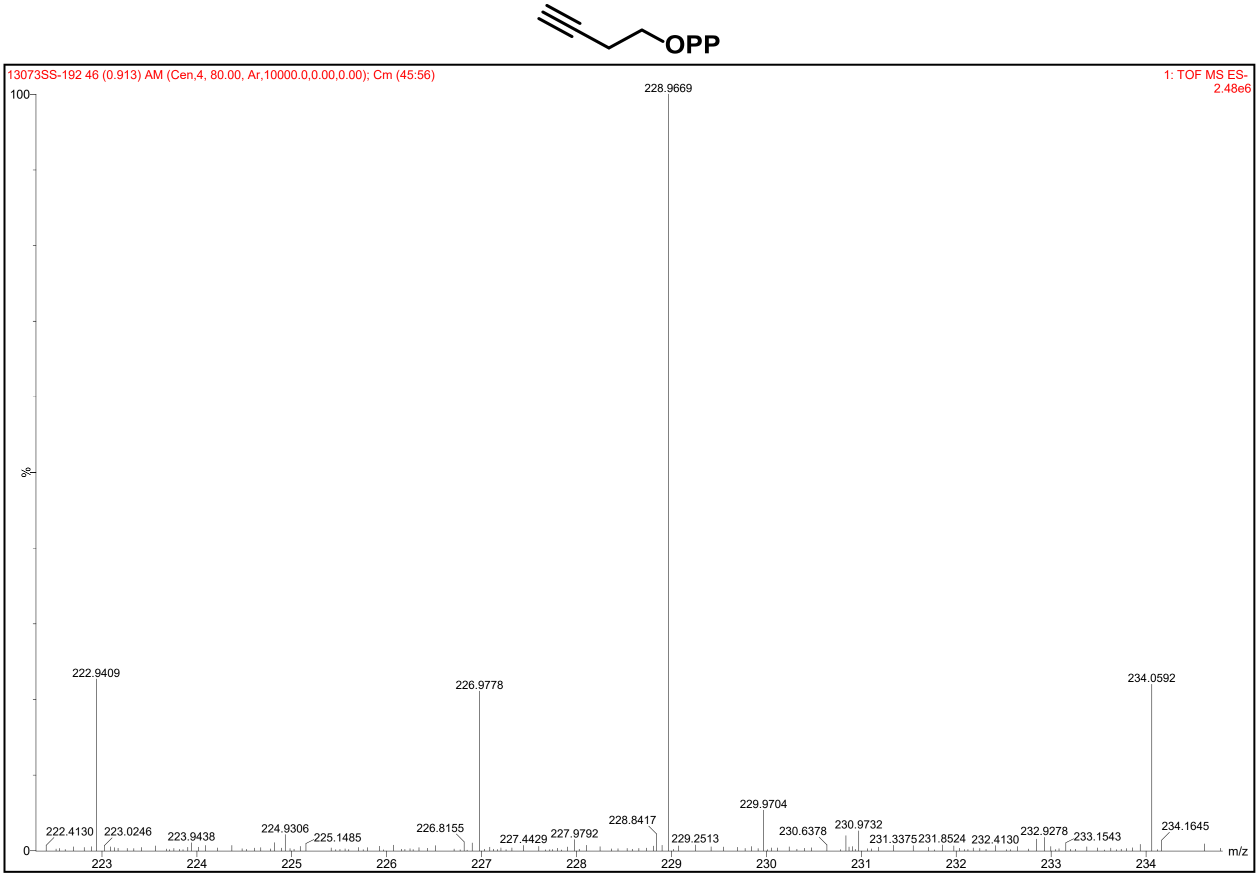

Figure S123. HRMS-ESI$^-$ of **8**-OPP.

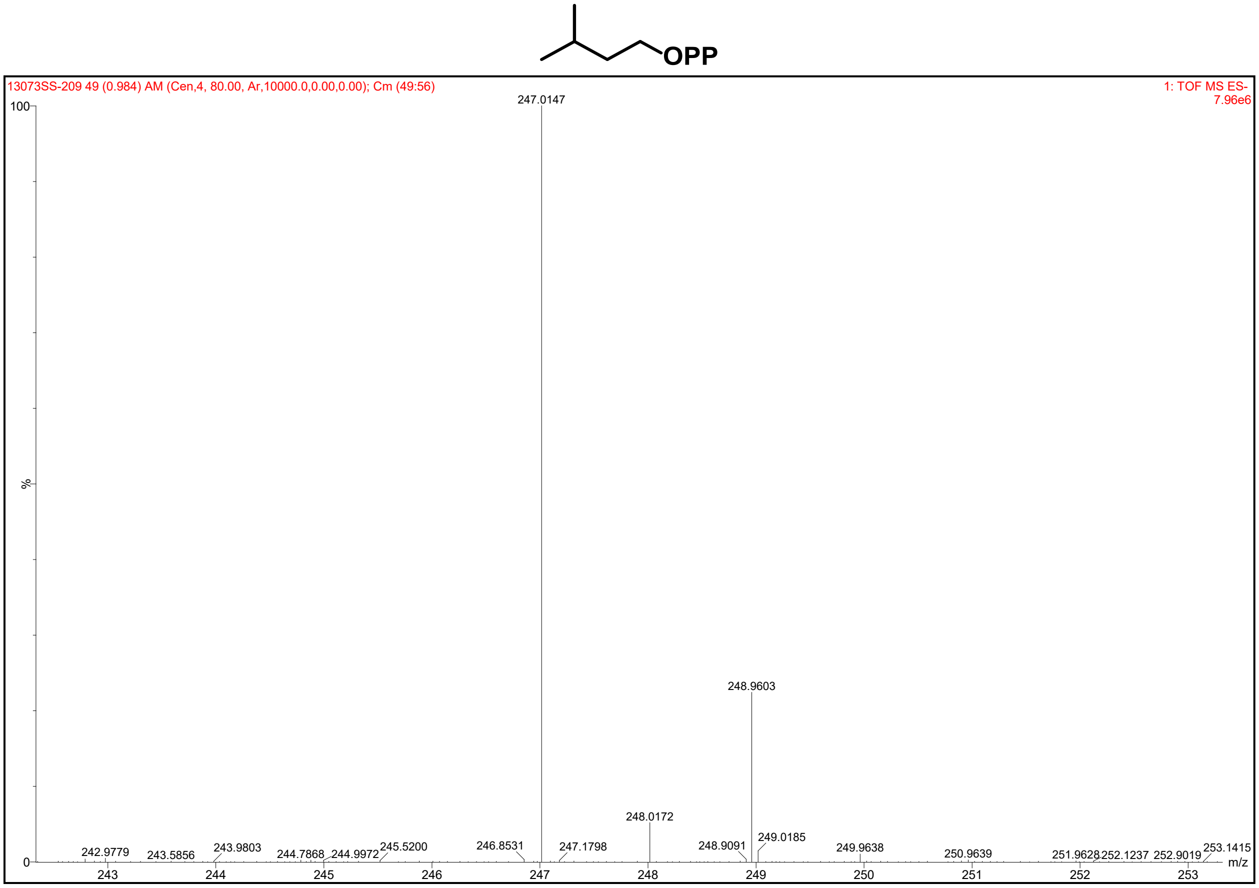

Figure S124. HRMS-ESI$^-$ of **9**-OPP.

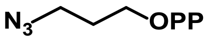

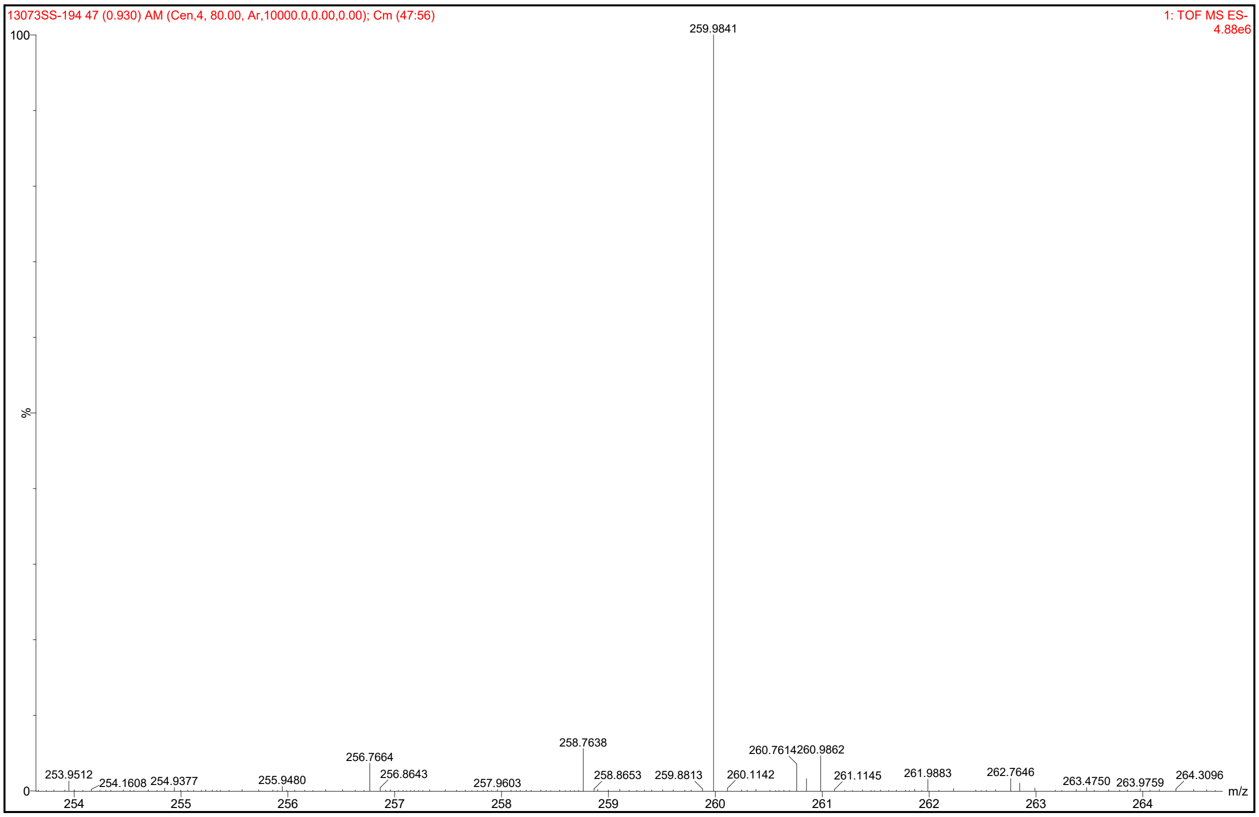

Figure S125. HRMS-ESI⁻ of **10**-OPP.

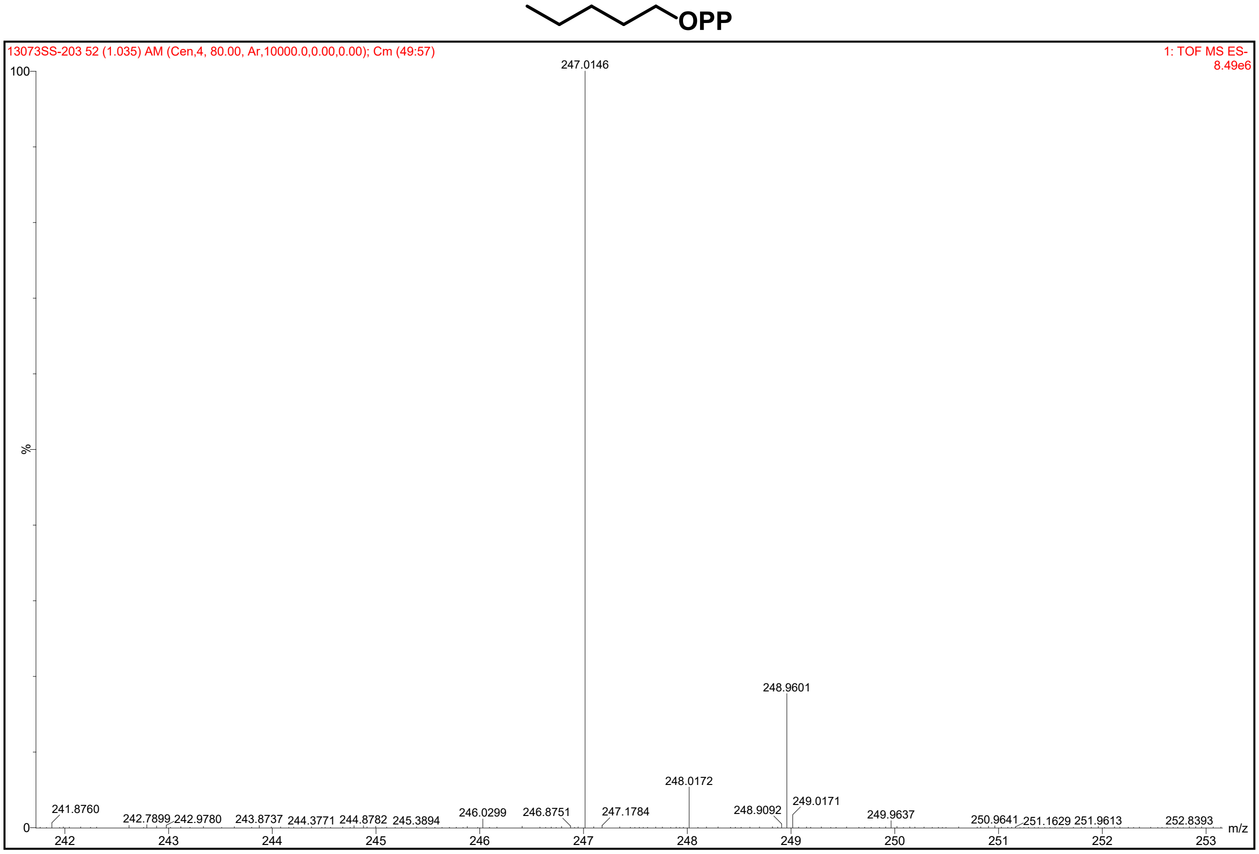

Figure S126. HRMS-ESI$^-$ of **11**-OPP.

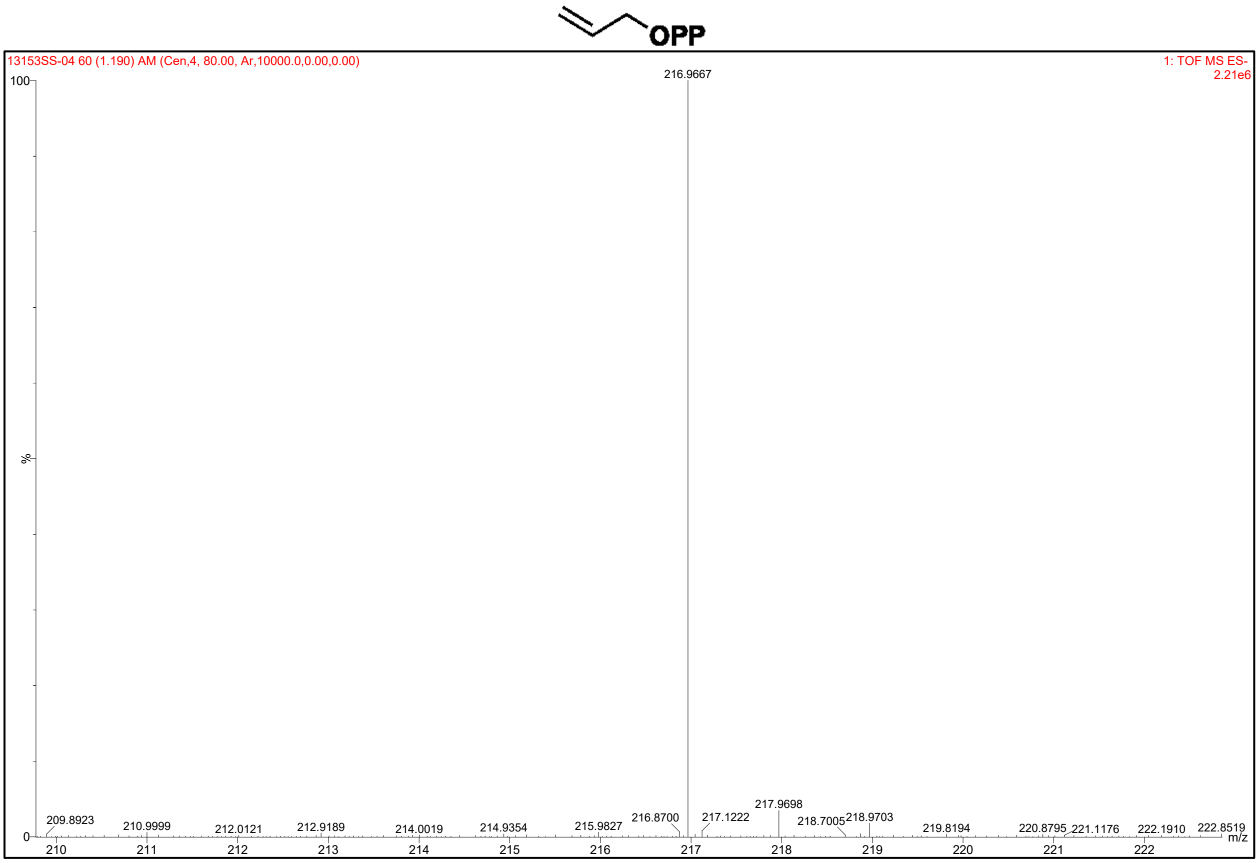

Figure S127. HRMS-ESI- of **12**-OPP.

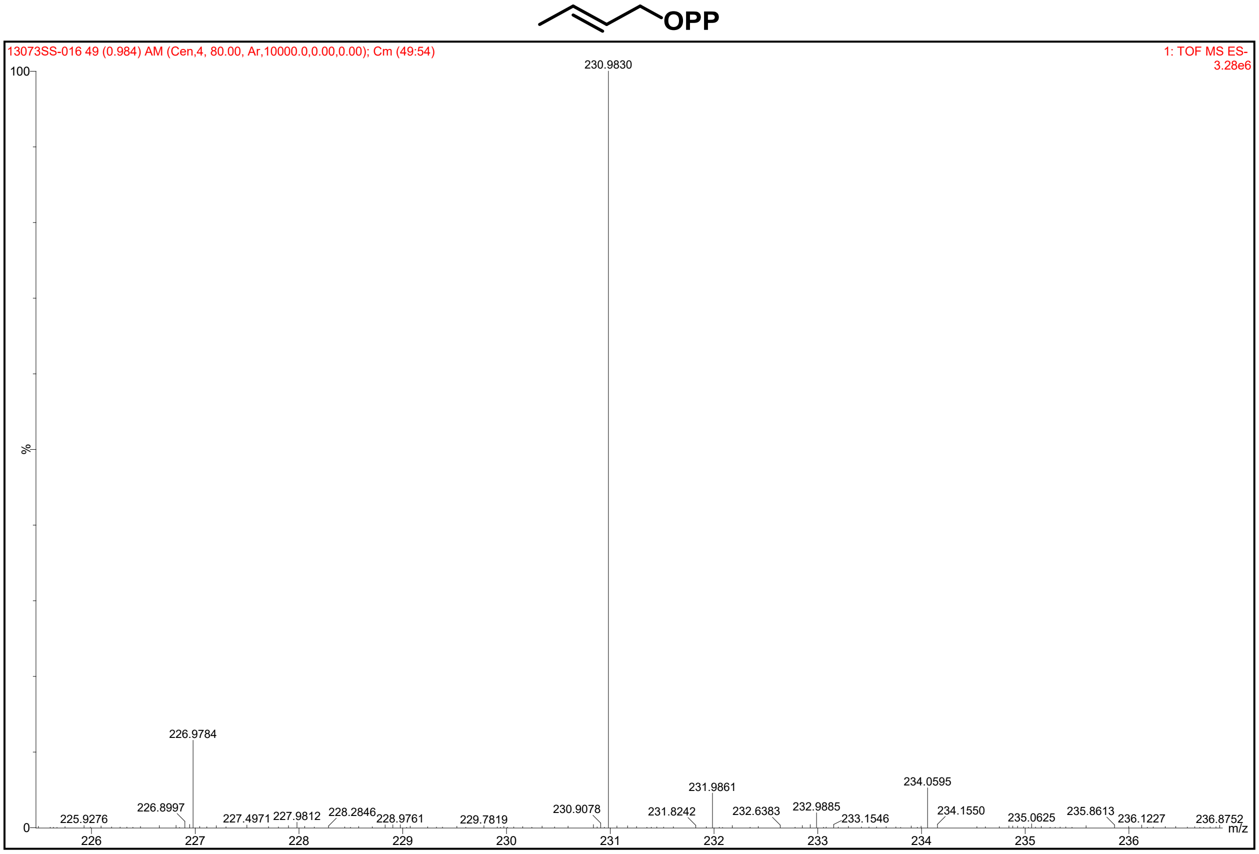

Figure S128. HRMS-ESI⁻ of **13**-OPP.

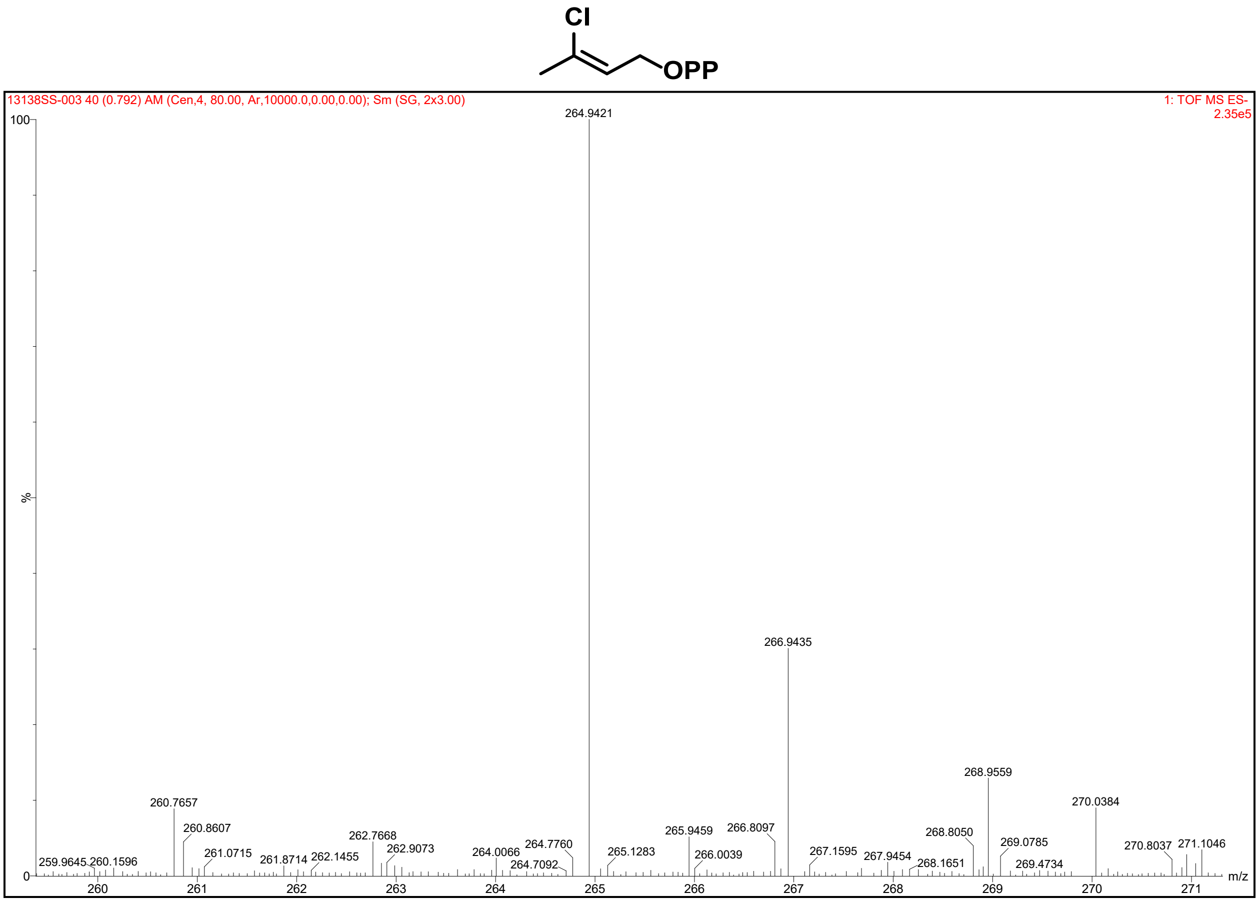

Figure S129. HRMS-ESI$^-$ of **14**-OPP.

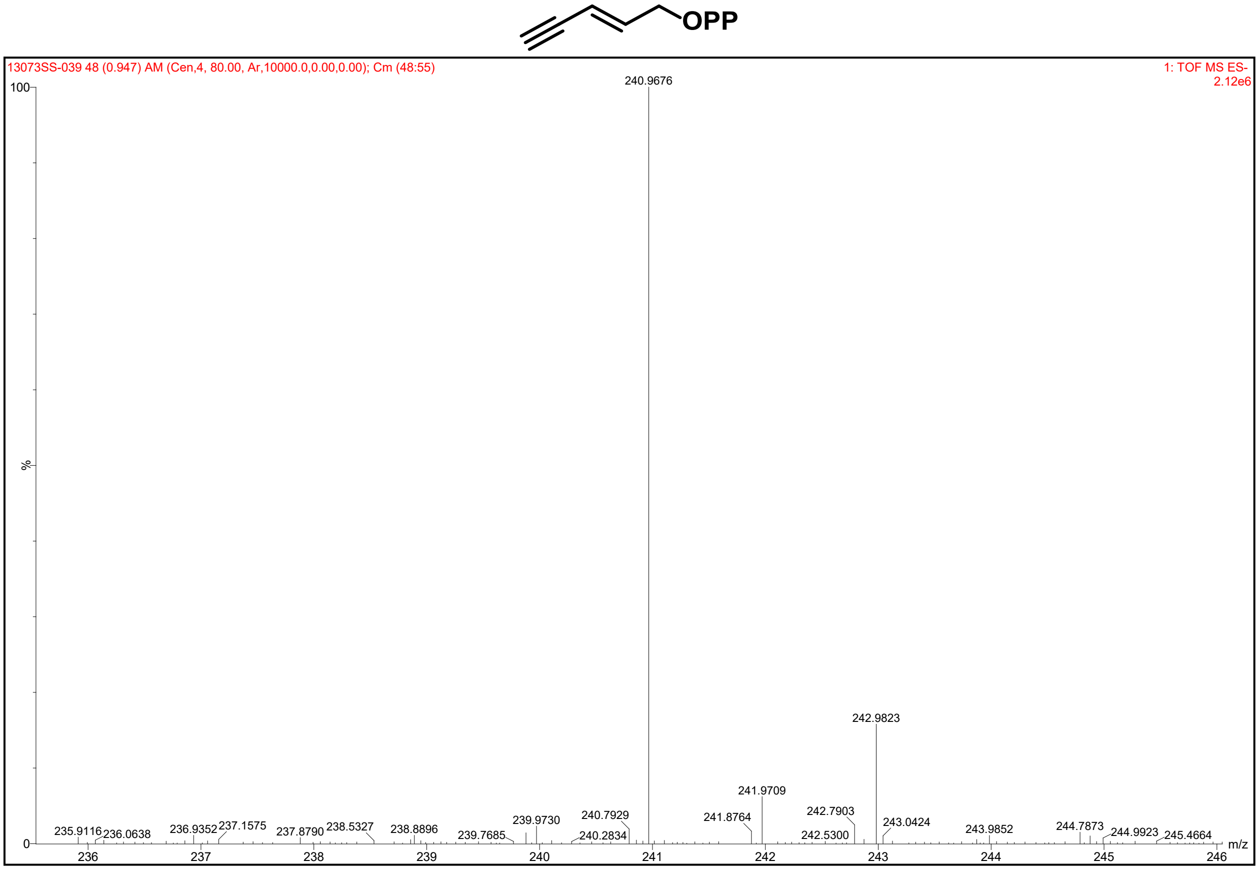

Figure S130. HRMS-ESI⁻ of **15**-OPP**.**

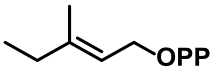

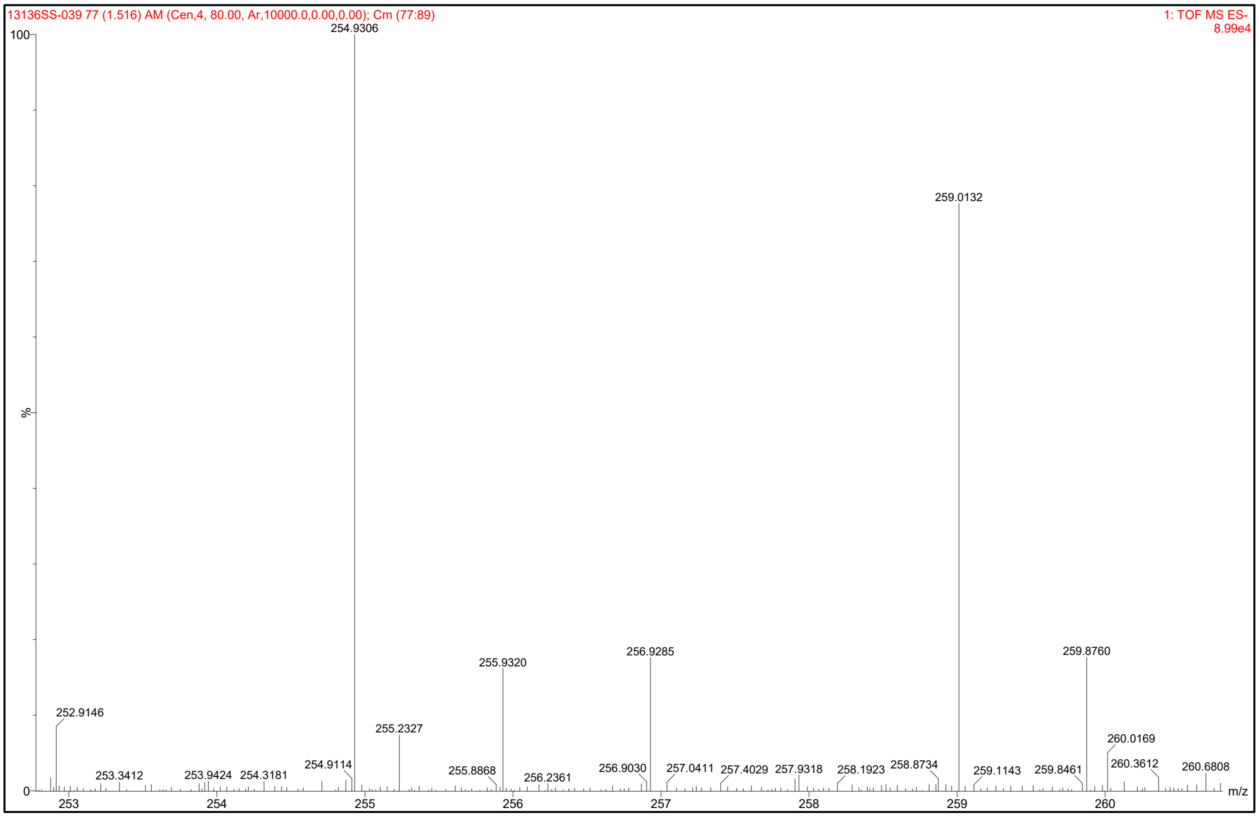

Figure S131. HRMS-ESI⁻ of **16**-OPP.

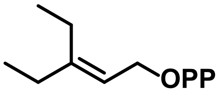

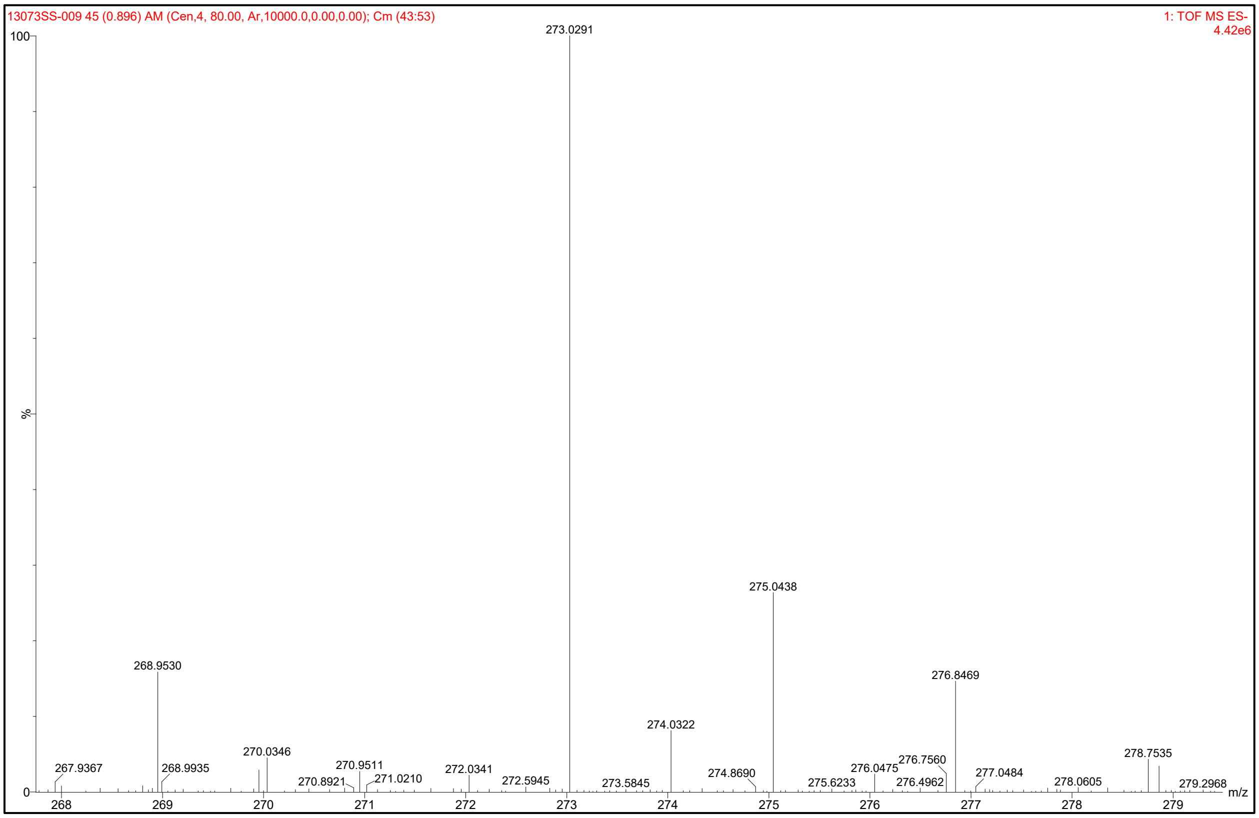

Figure S132. HRMS-ESI$^-$ of **17**-OPP.

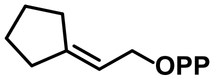

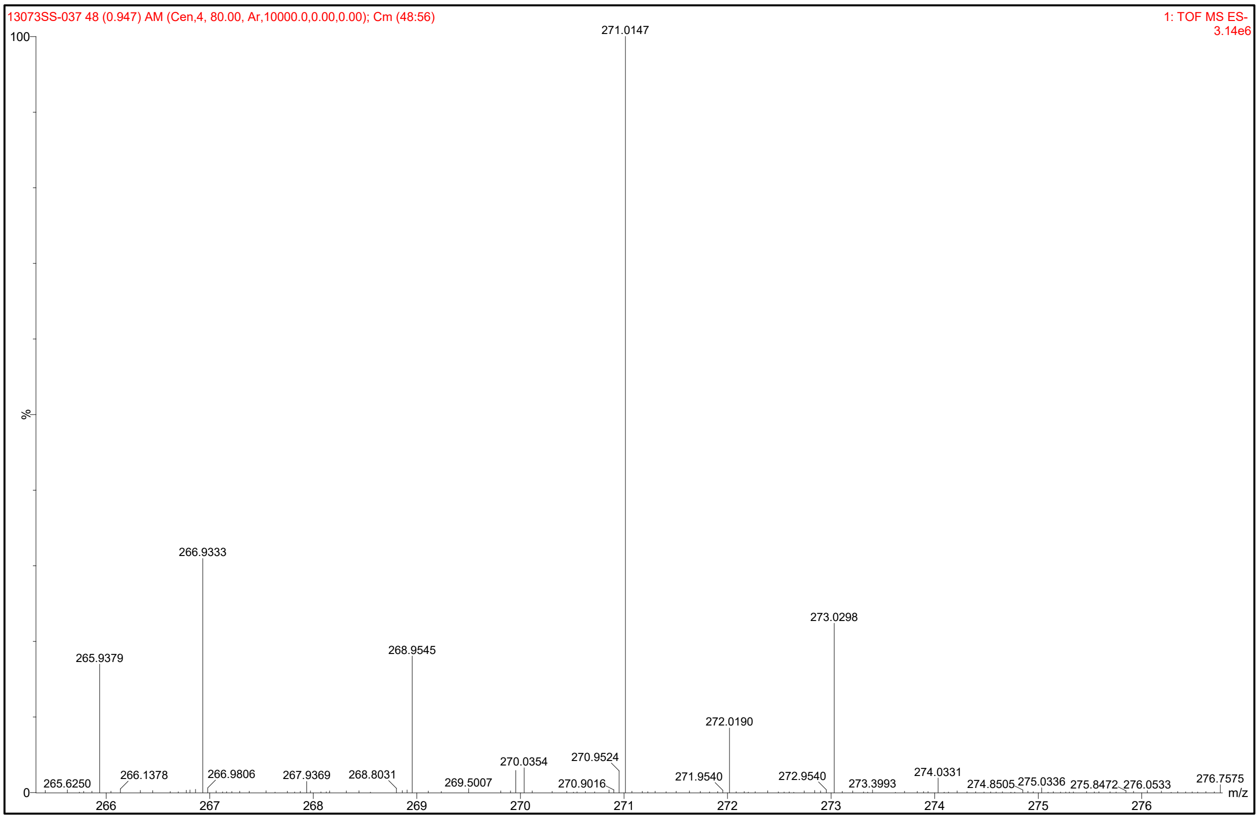

Figure S133. HRMS-ESI- of **18**-OPP.

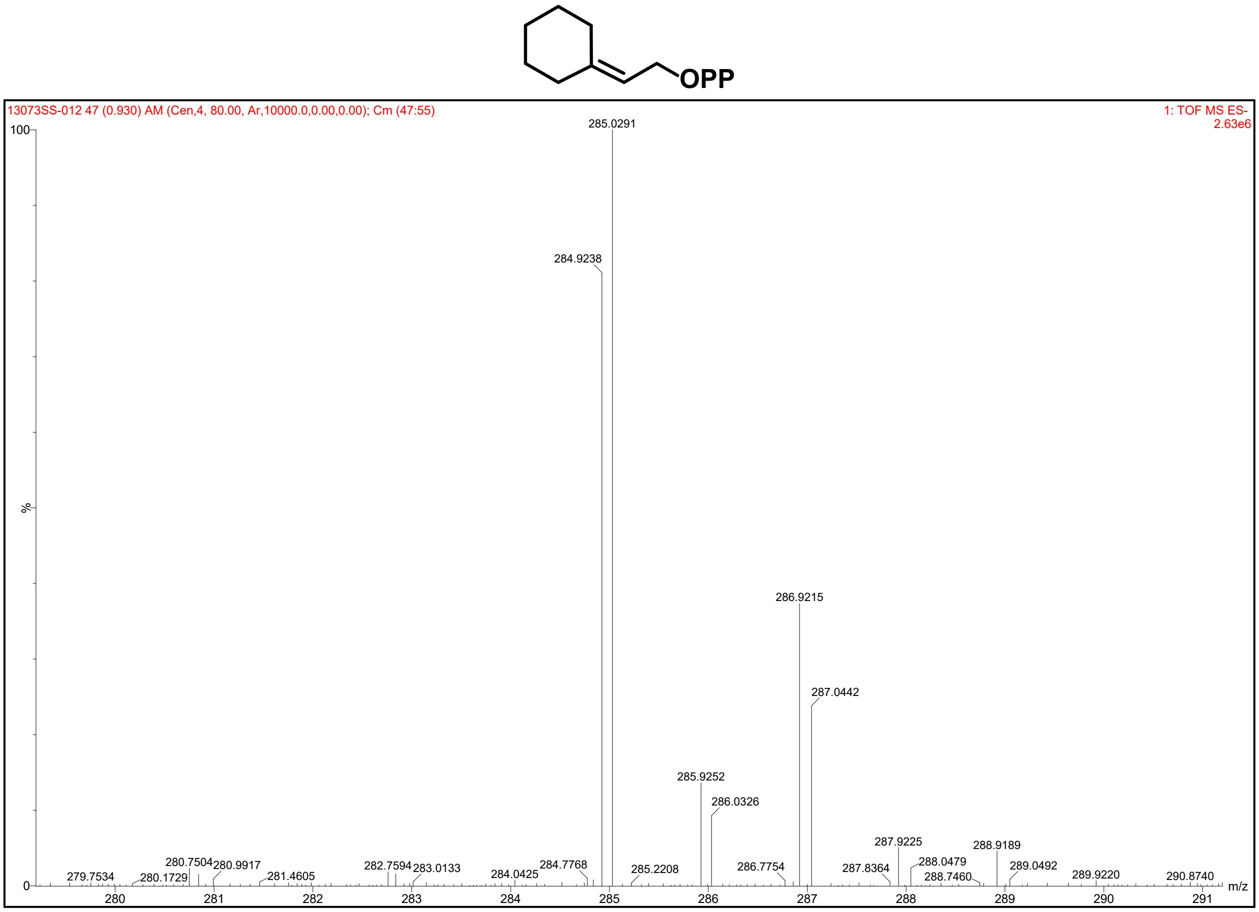

Figure S134. HRMS-ESI⁻ of **19**-OPP.

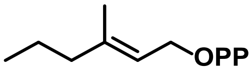

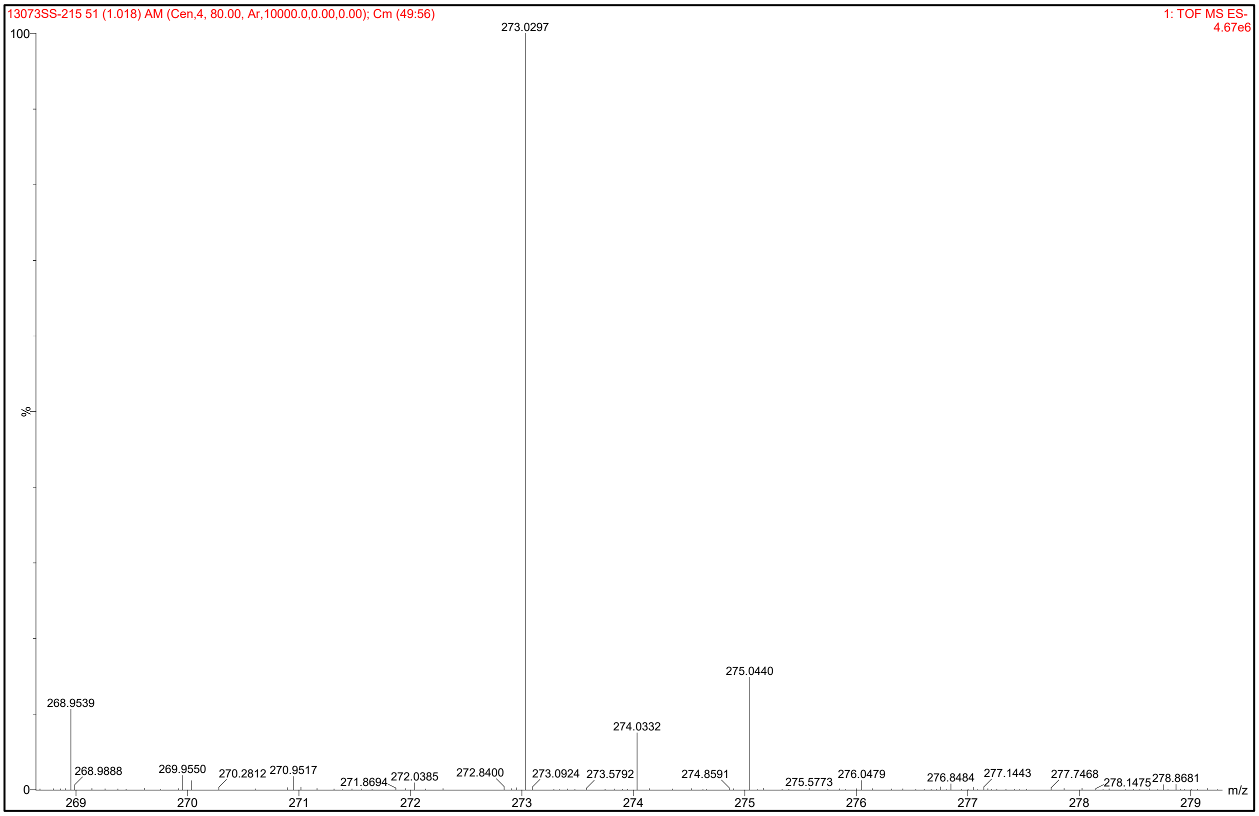

Figure S135. HRMS-ESI$^-$ of **23**-OPP.

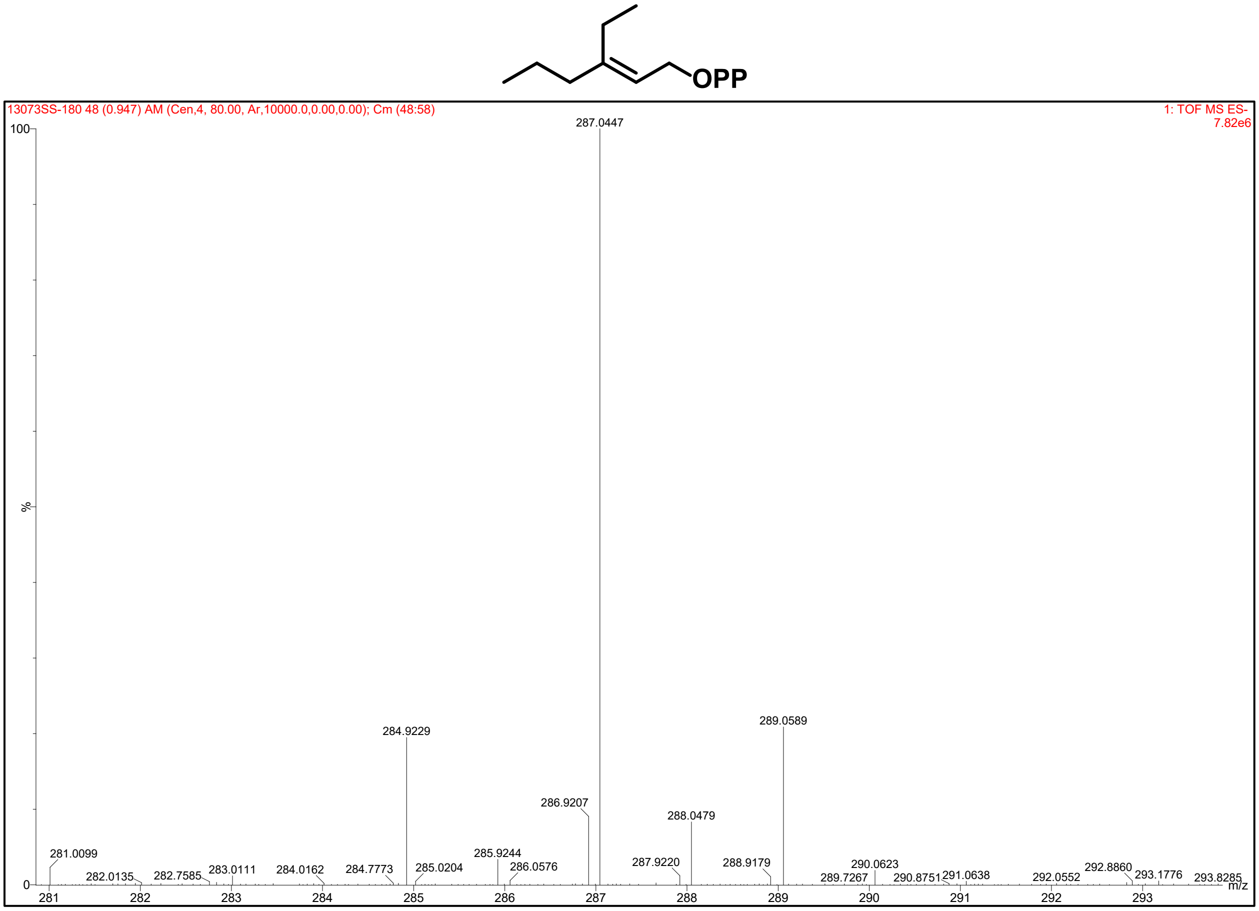

Figure S136. HRMS-ESI$^-$ of **24**-OPP.

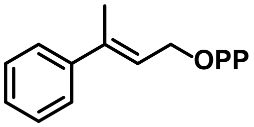

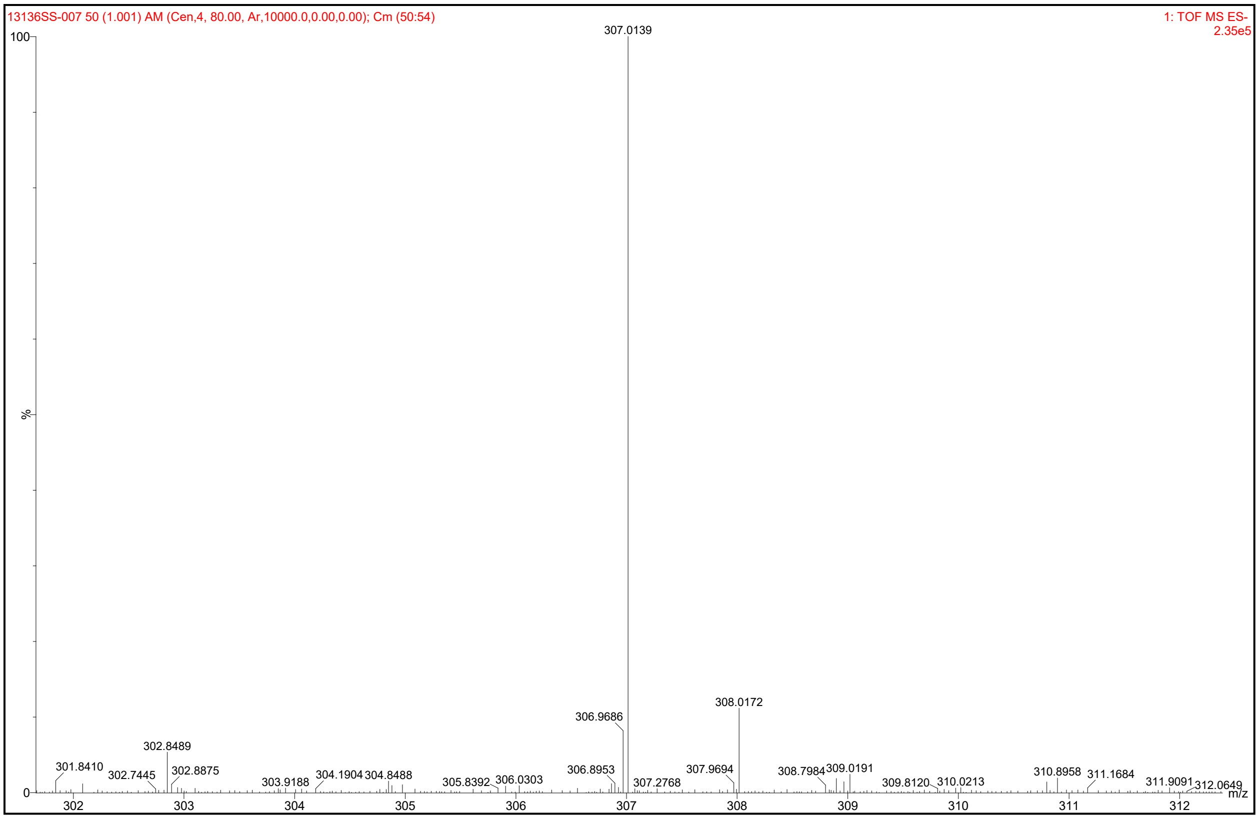

Figure S137. HRMS-ESI- of **26**-OPP.

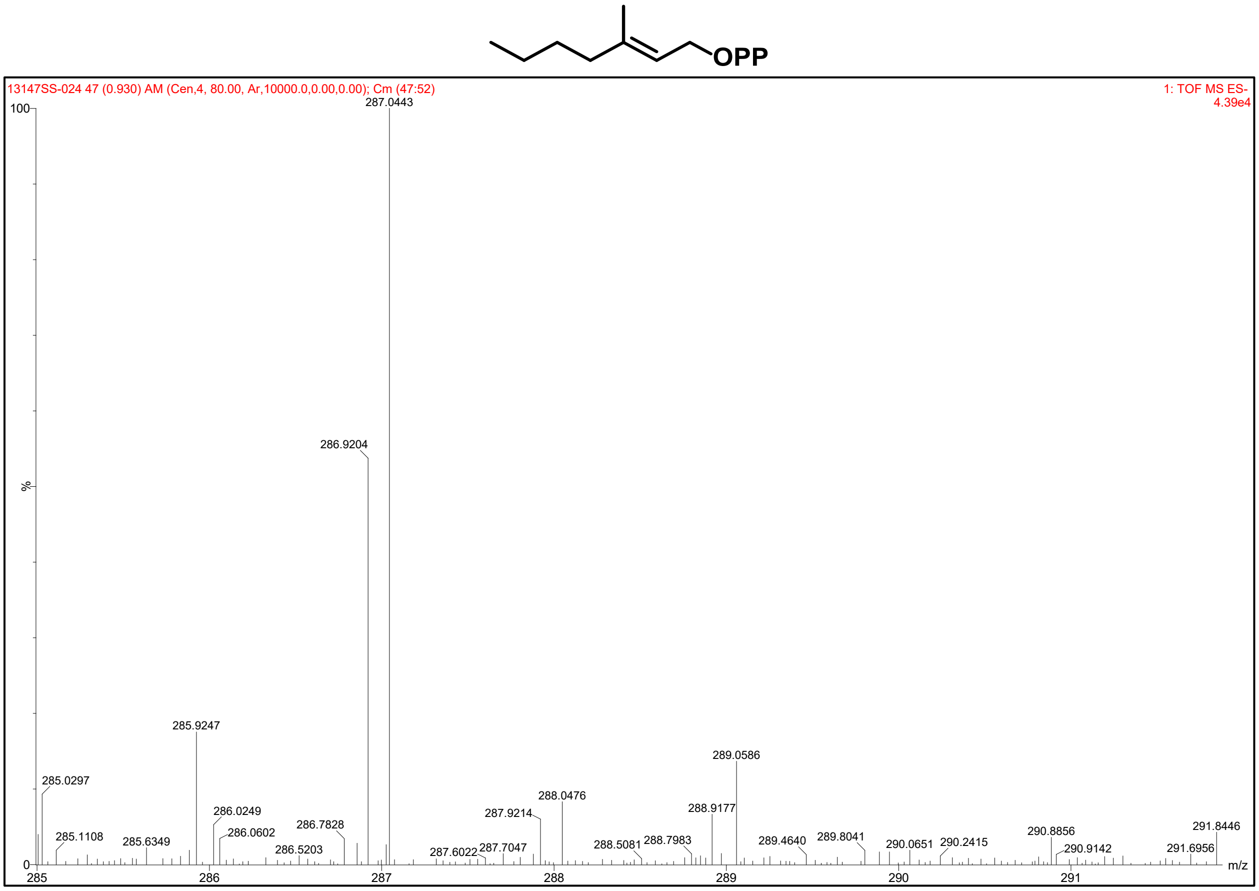

Figure S138. HRMS-ESI^{+} of **27**-OPP.

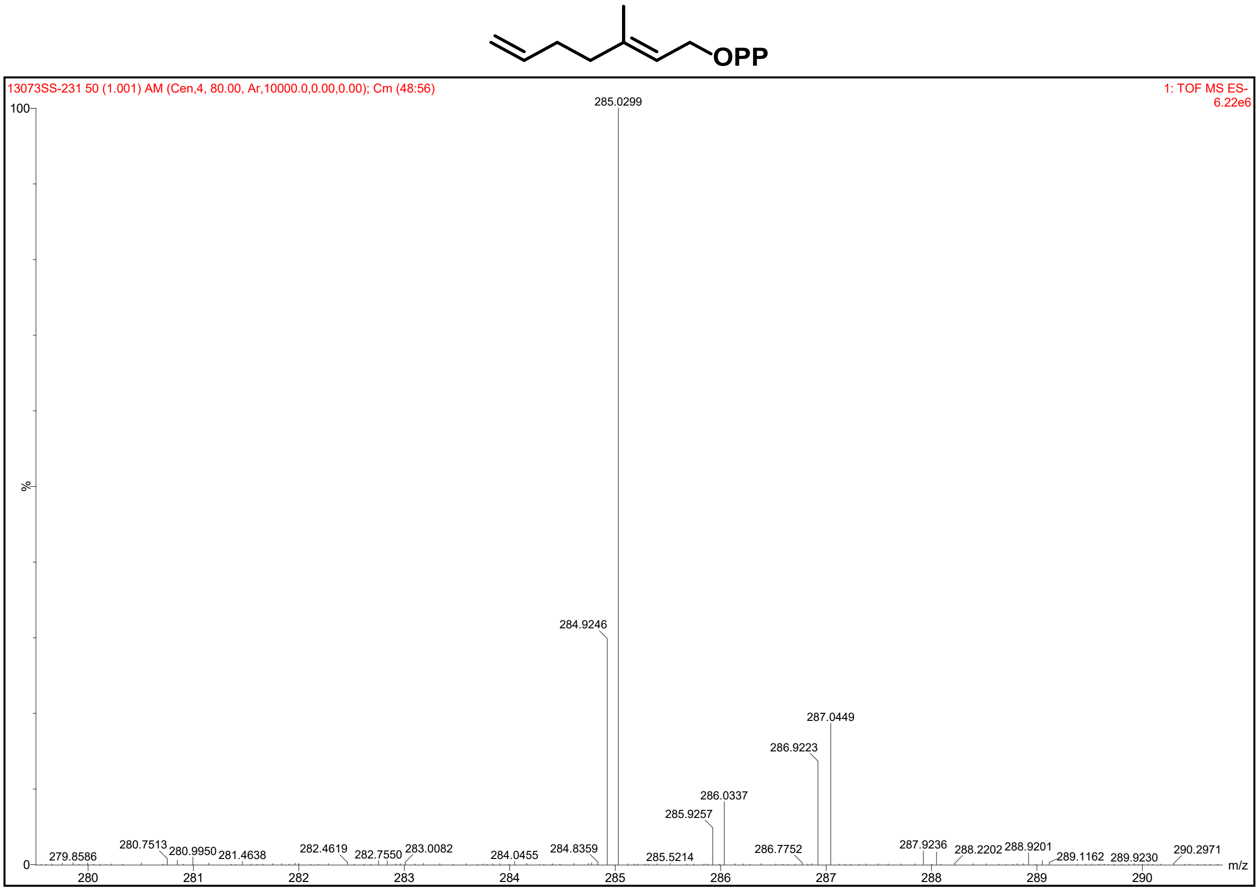

Figure S139. HRMS-ESI⁻ of **28**-OPP.

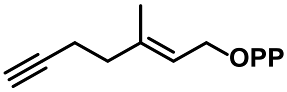

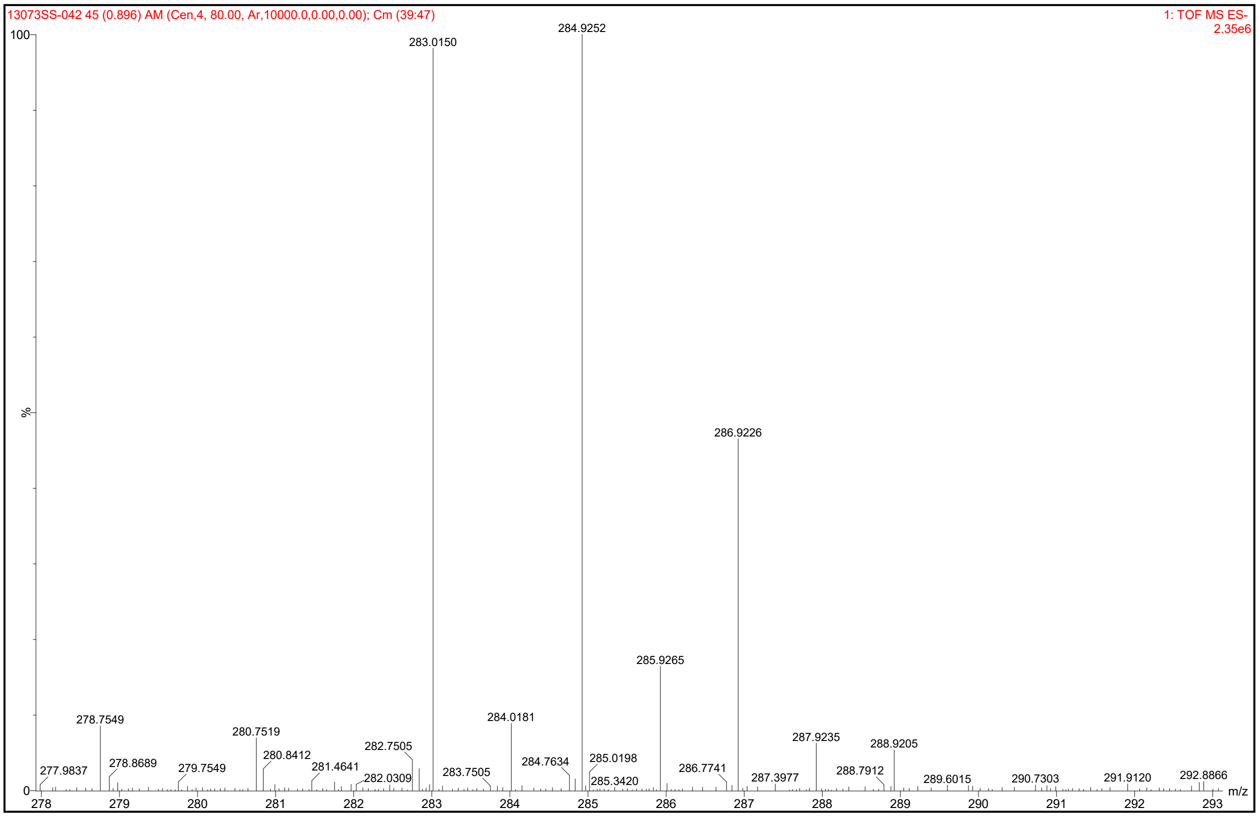

Figure S140. HRMS-ESI$^-$ of **29**-OPP.

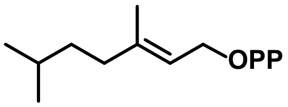

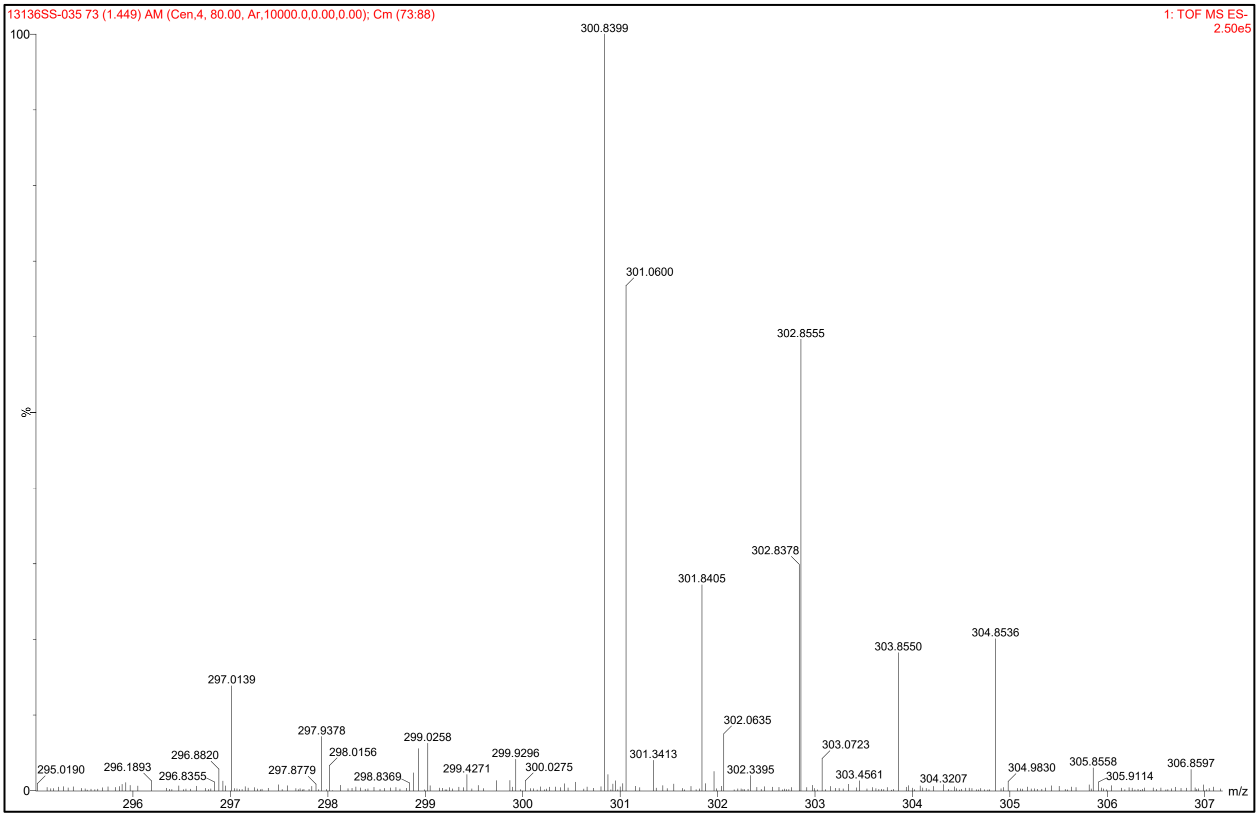

Figure S141. HRMS-ESI$^-$ of **30**-OPP.

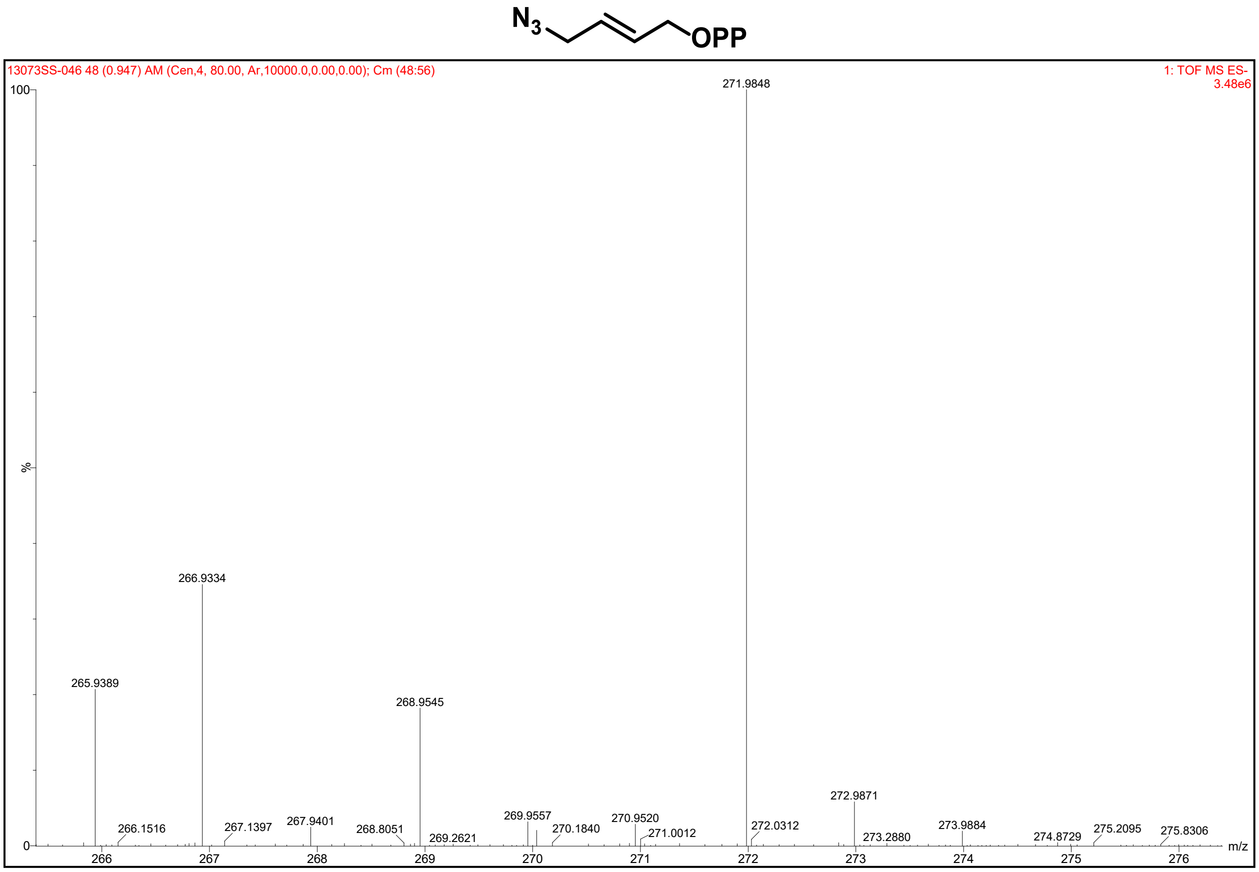

Figure S142. HRMS-ESI^{-} of **32**-OPP.

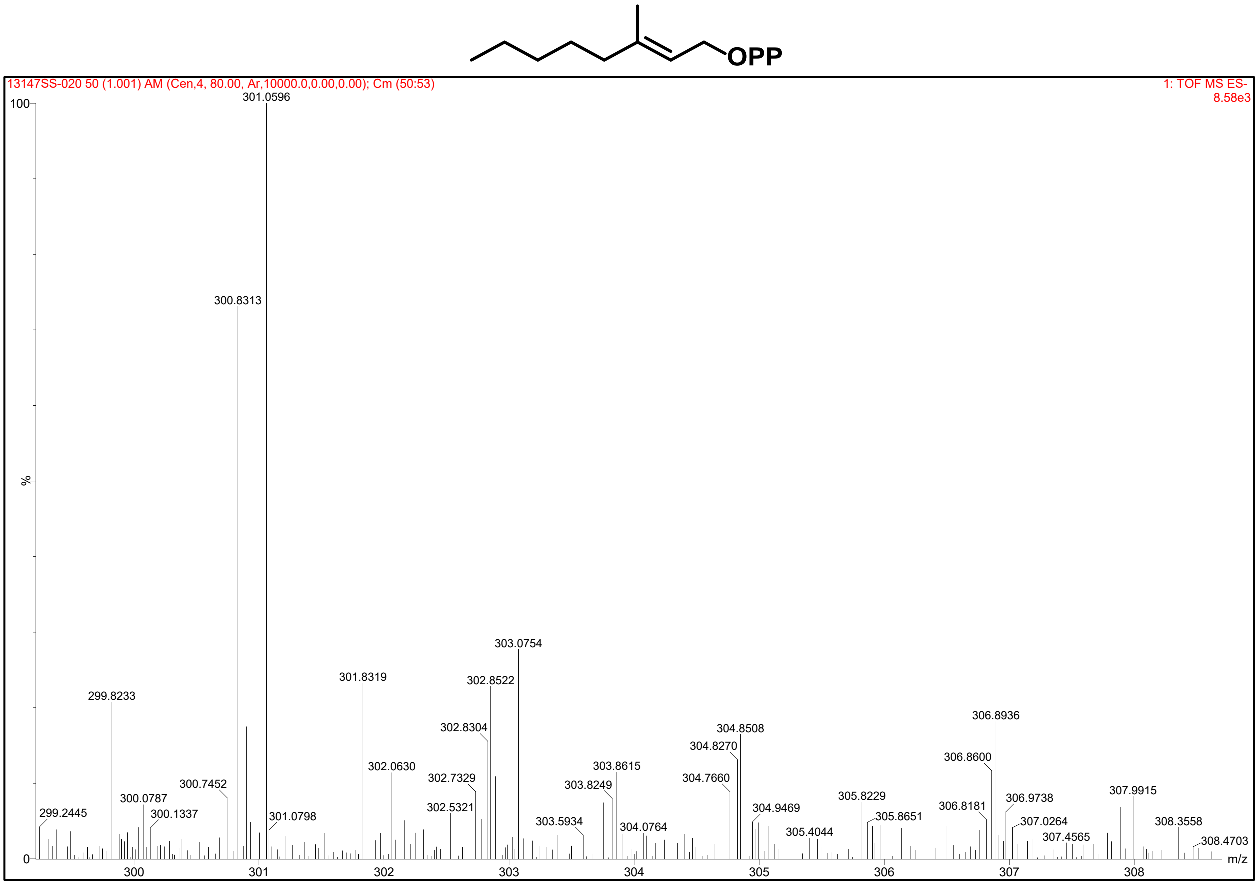

Figure S143. HRMS-ESI$^-$ of **34**-OPP.

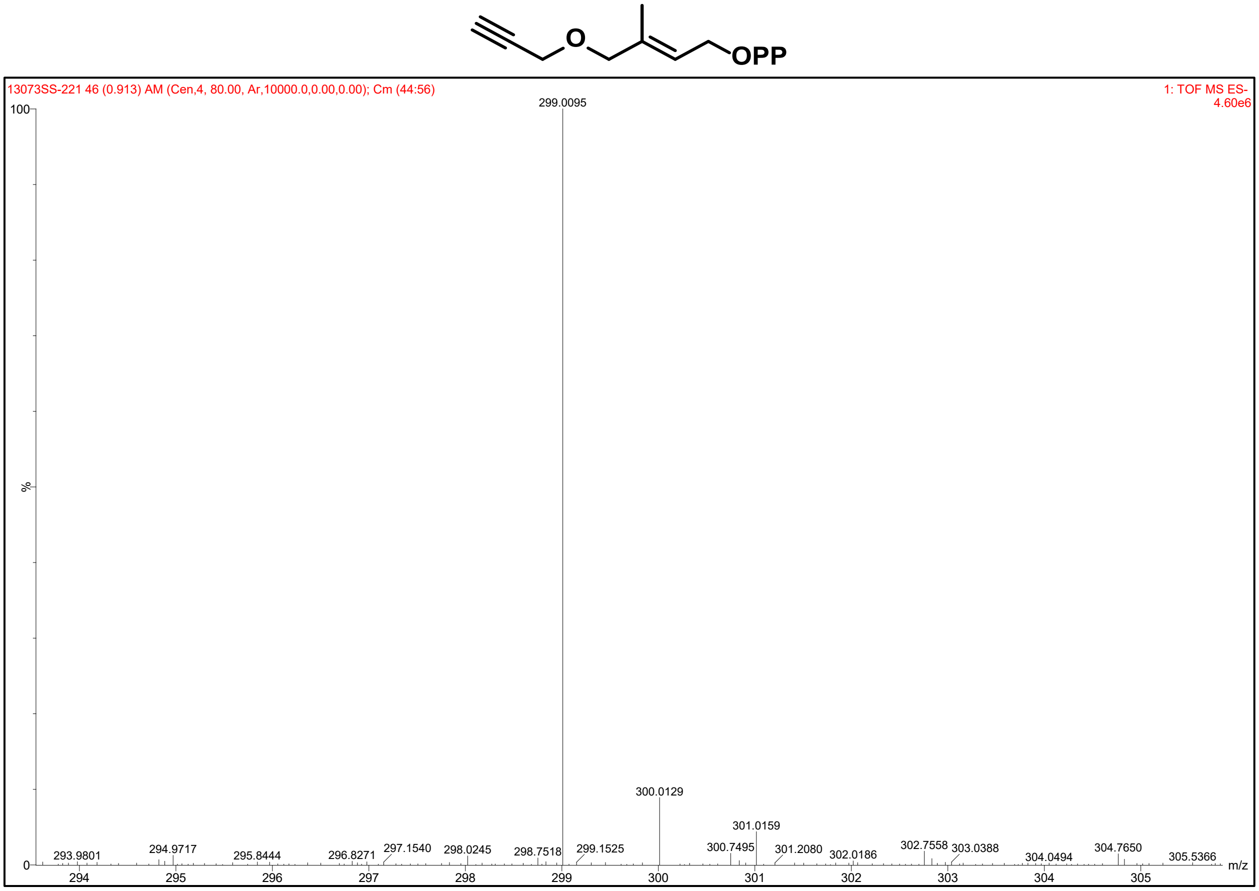

Figure S144. HRMS-ESI⁻ of **35**-OPP.

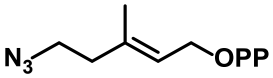

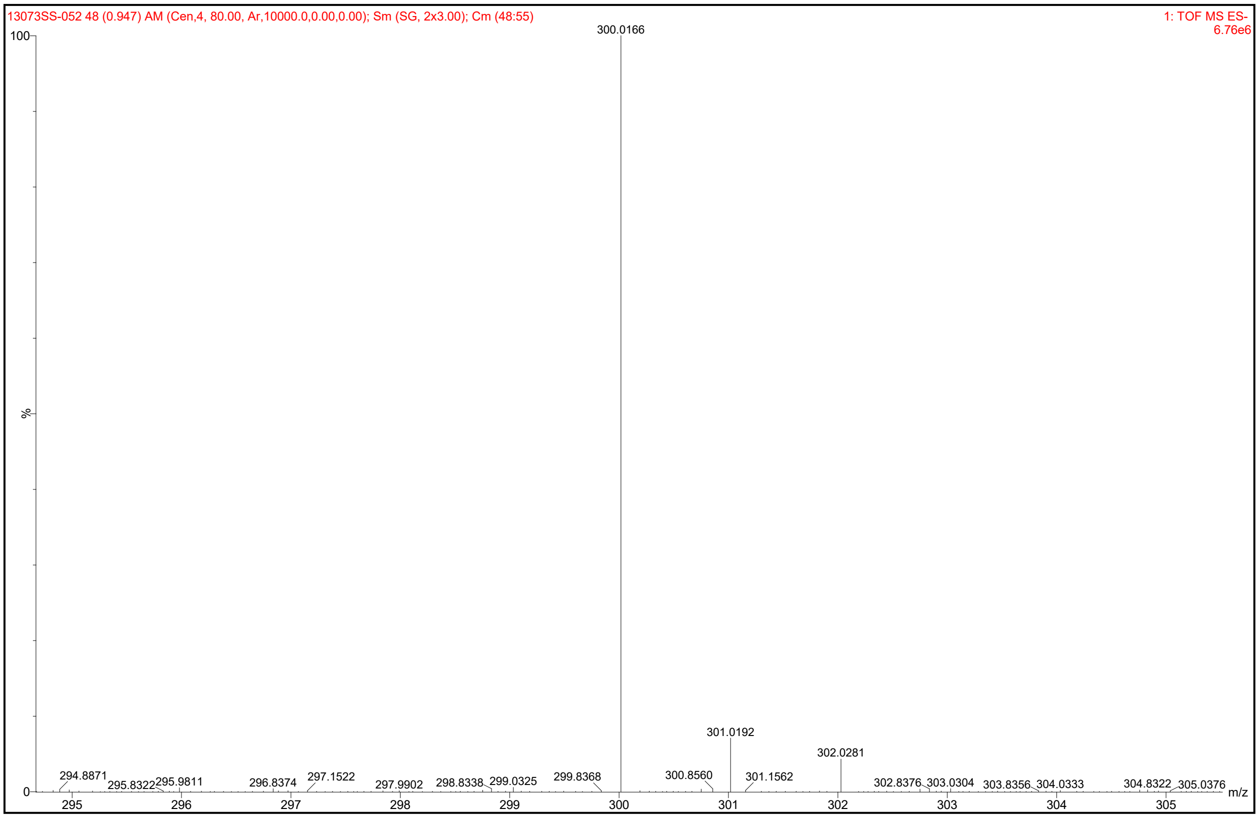

Figure S145. HRMS-ESI$^-$ of **36**-OPP.

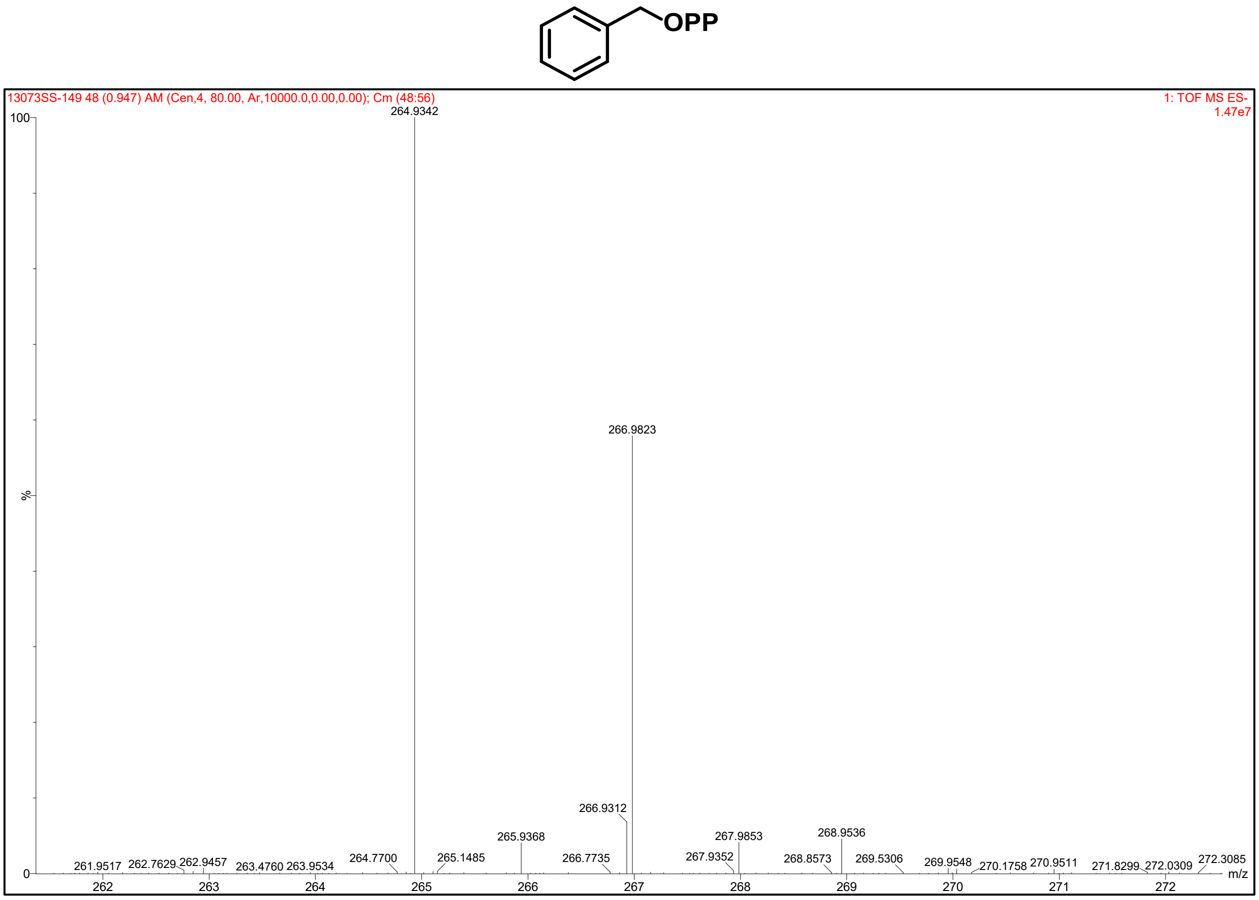

Figure S146. HRMS-ESI^{-} of **46**-OPP.

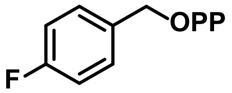

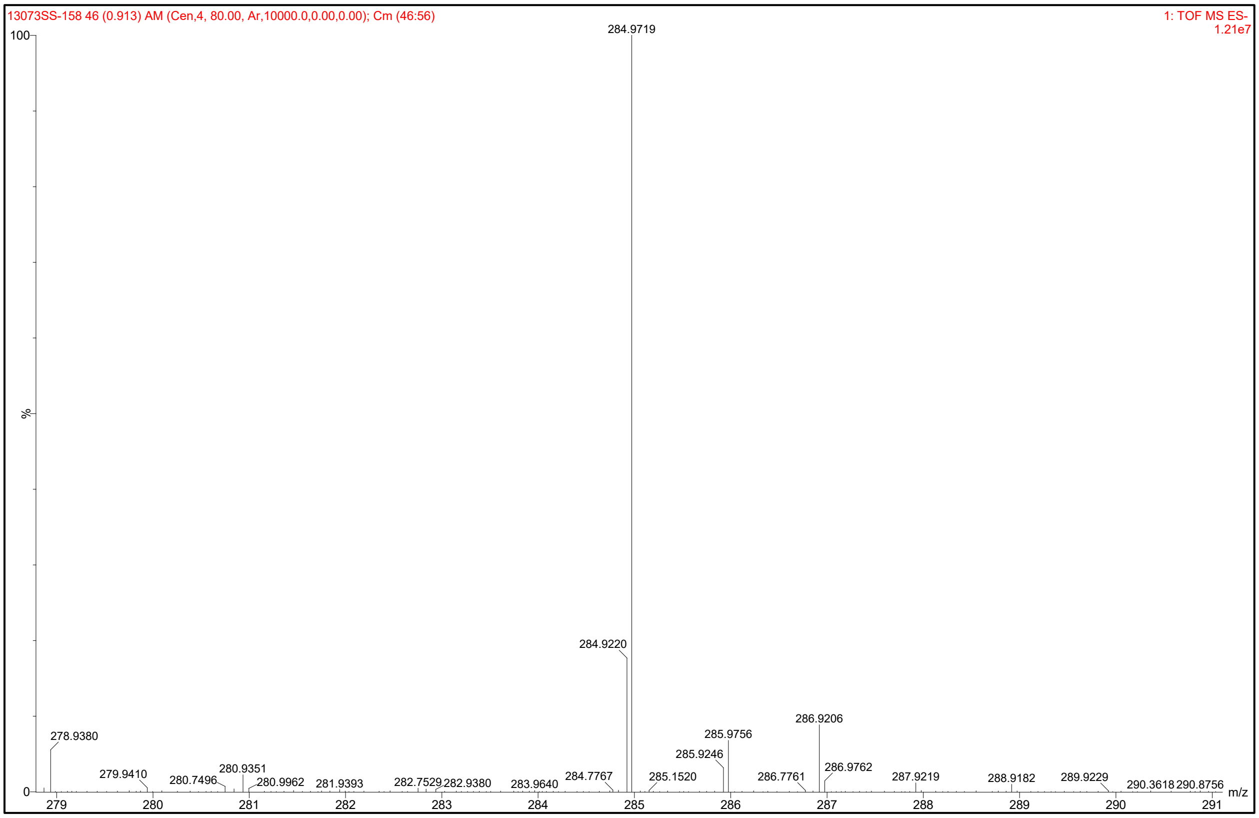

Figure S147. HRMS-ESI^{-} of **47**-OPP**.**

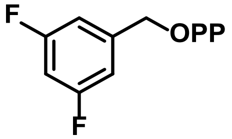

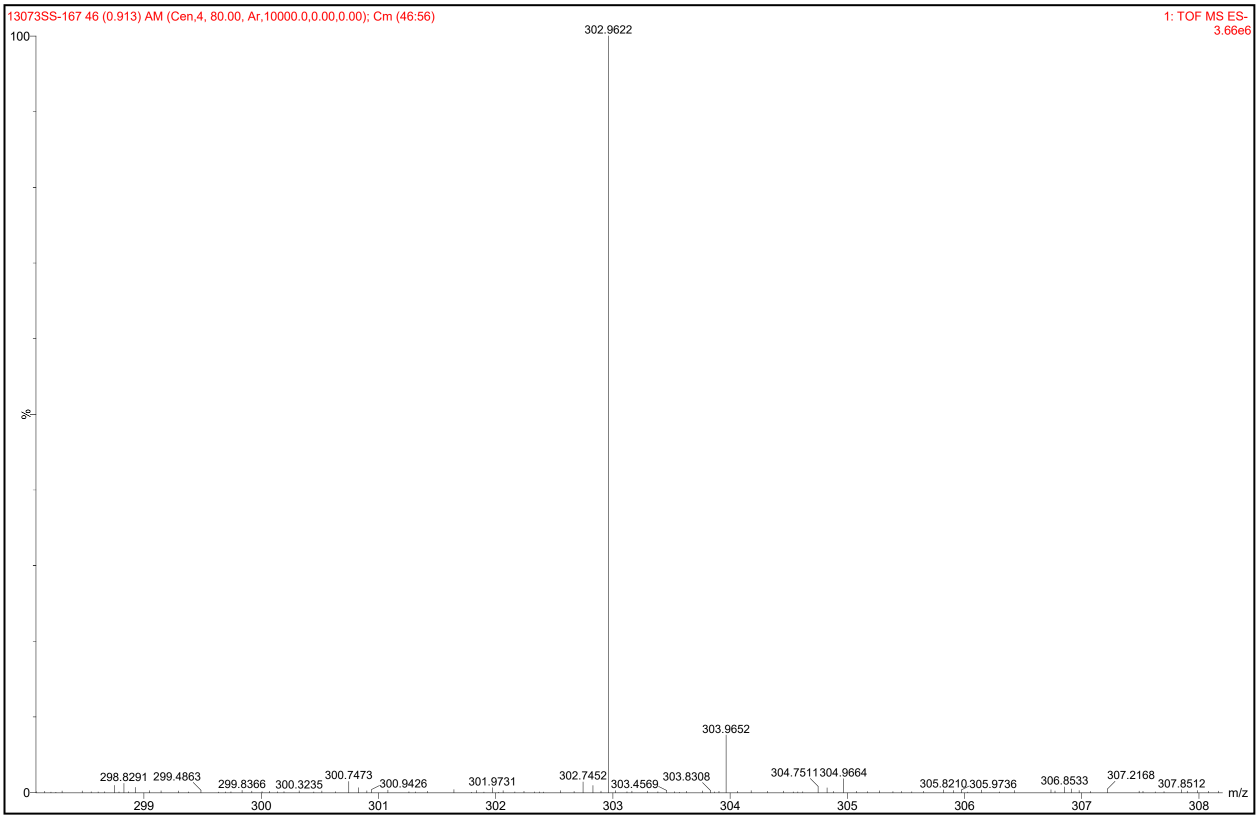

Figure S148. HRMS-ESI⁻ of **48**-OPP.

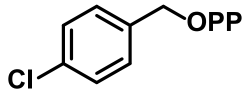

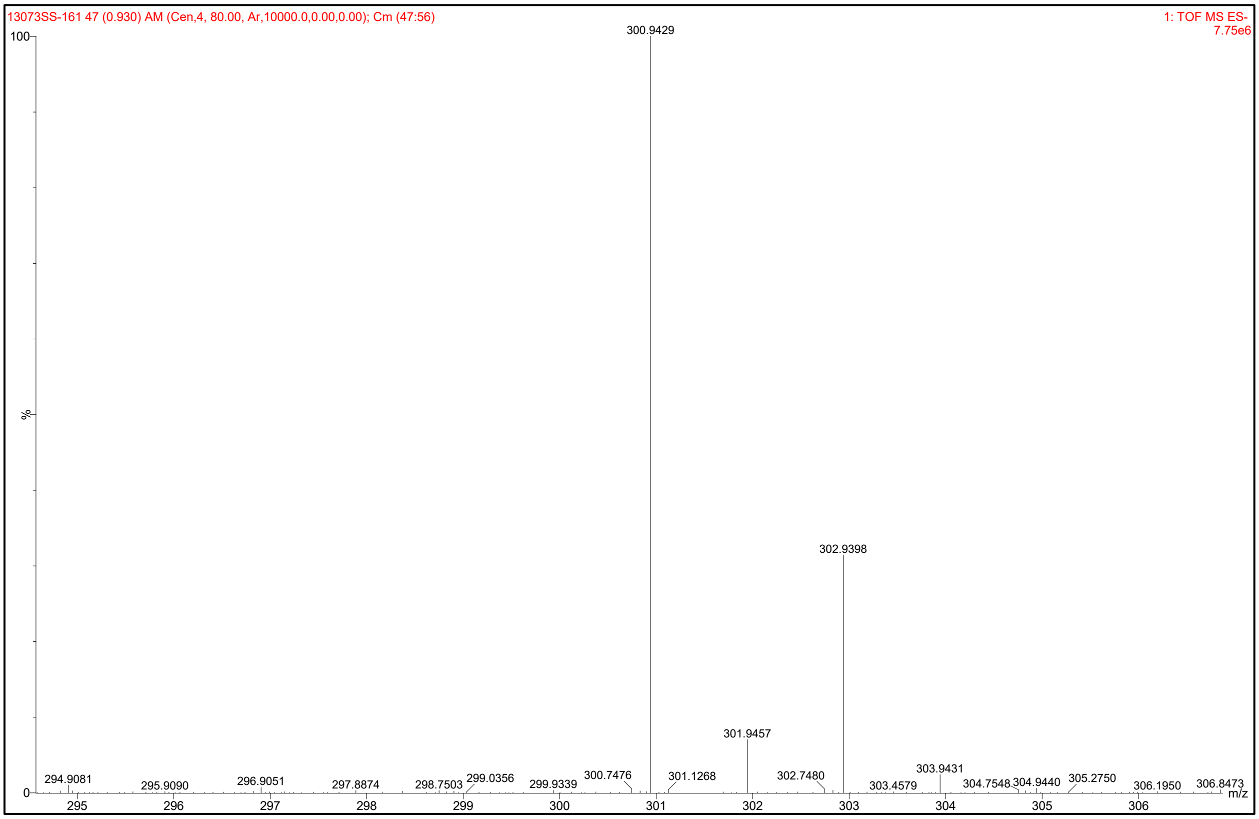

Figure S149. HRMS-ESI⁻ of **49**-OPP.

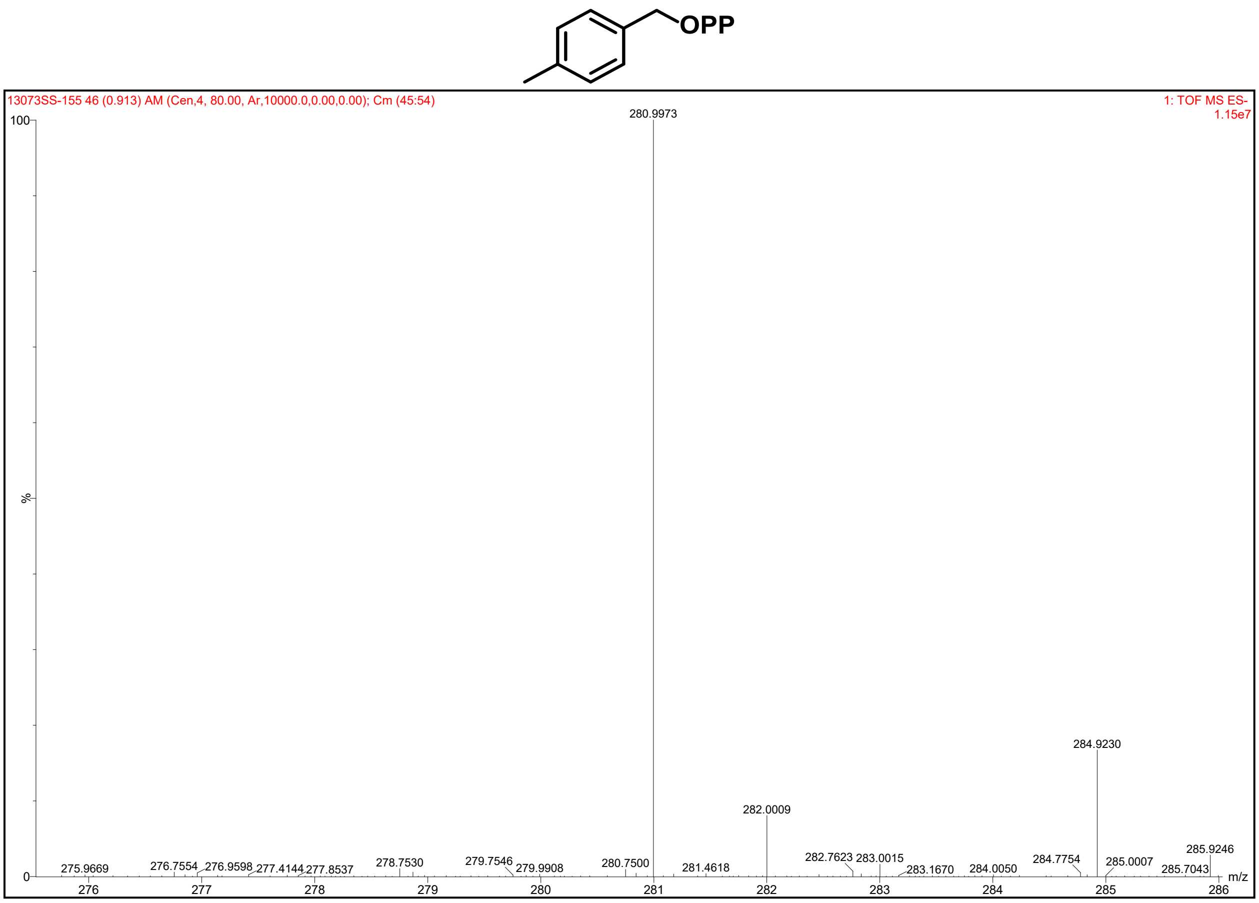

Figure S150. HRMS-ESI^{-} of **50**-OPP.

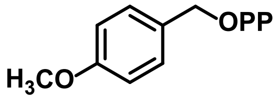

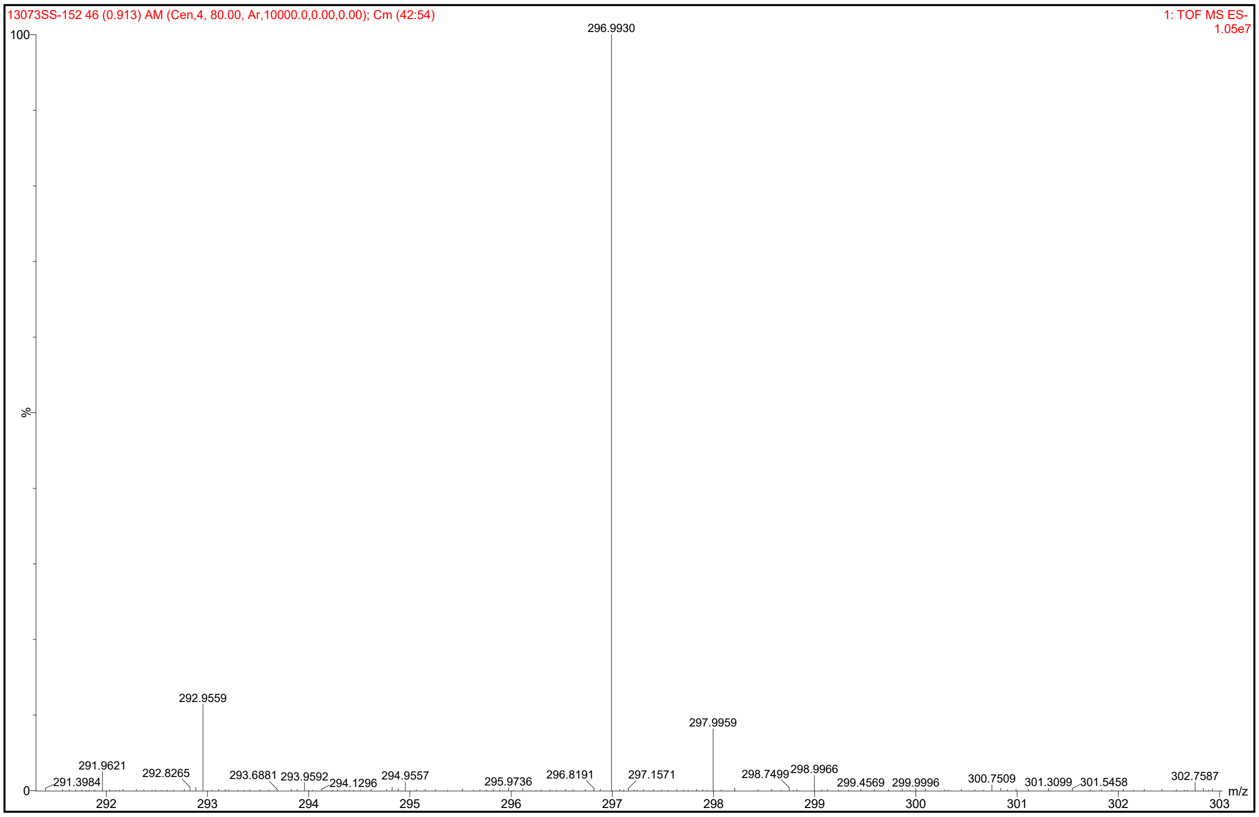

Figure S151. HRMS-ESI- of **52**-OPP.

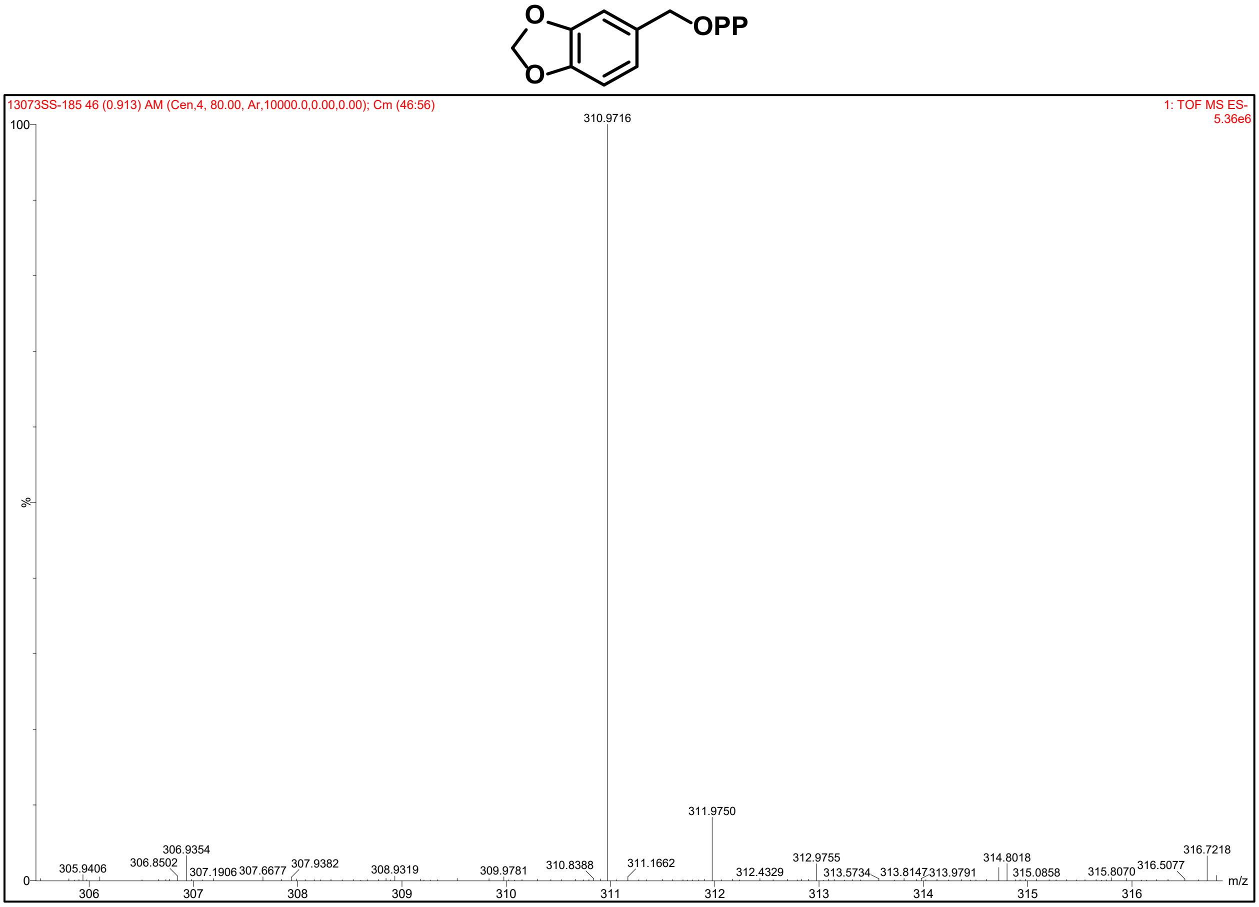

Figure S152. HRMS-ESI$^-$ of **54**-OPP.

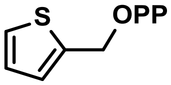

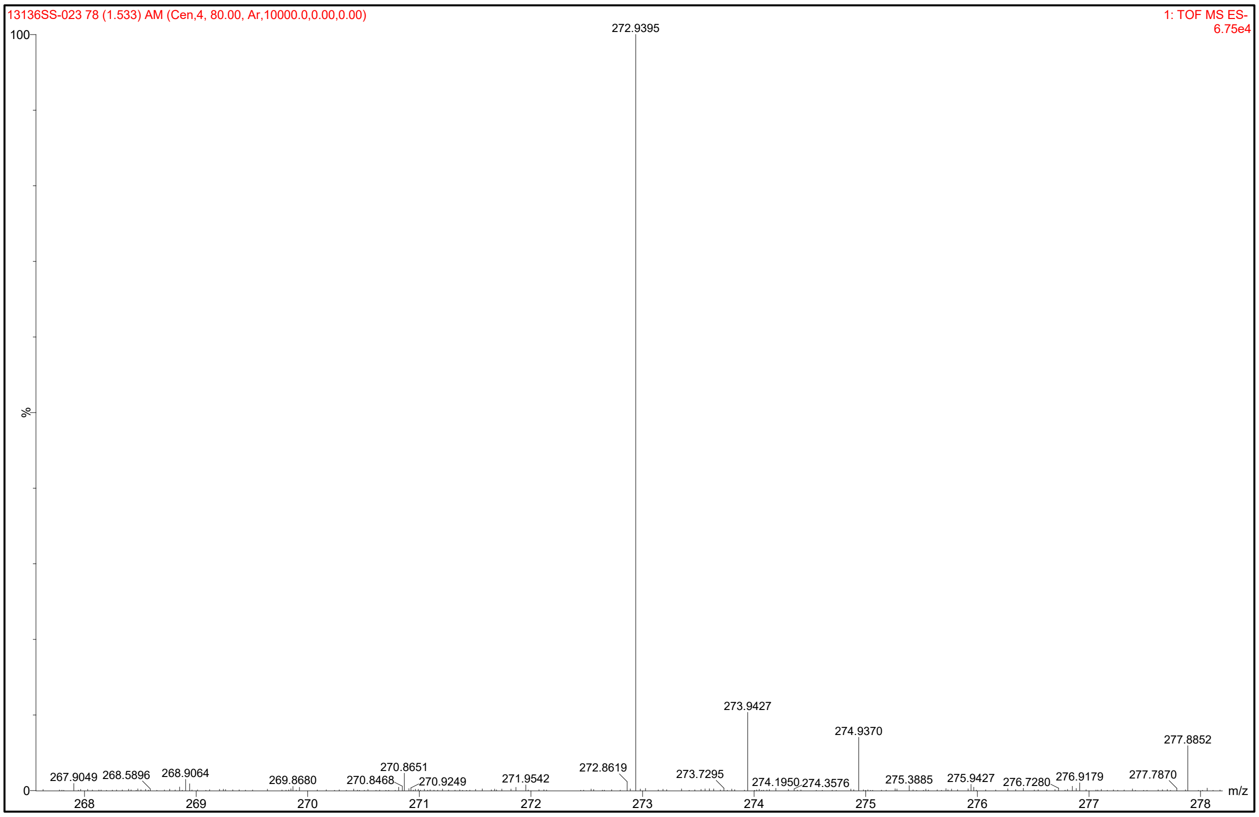

Figure S153. HRMS-ESI^{-} of **56**-OPP.

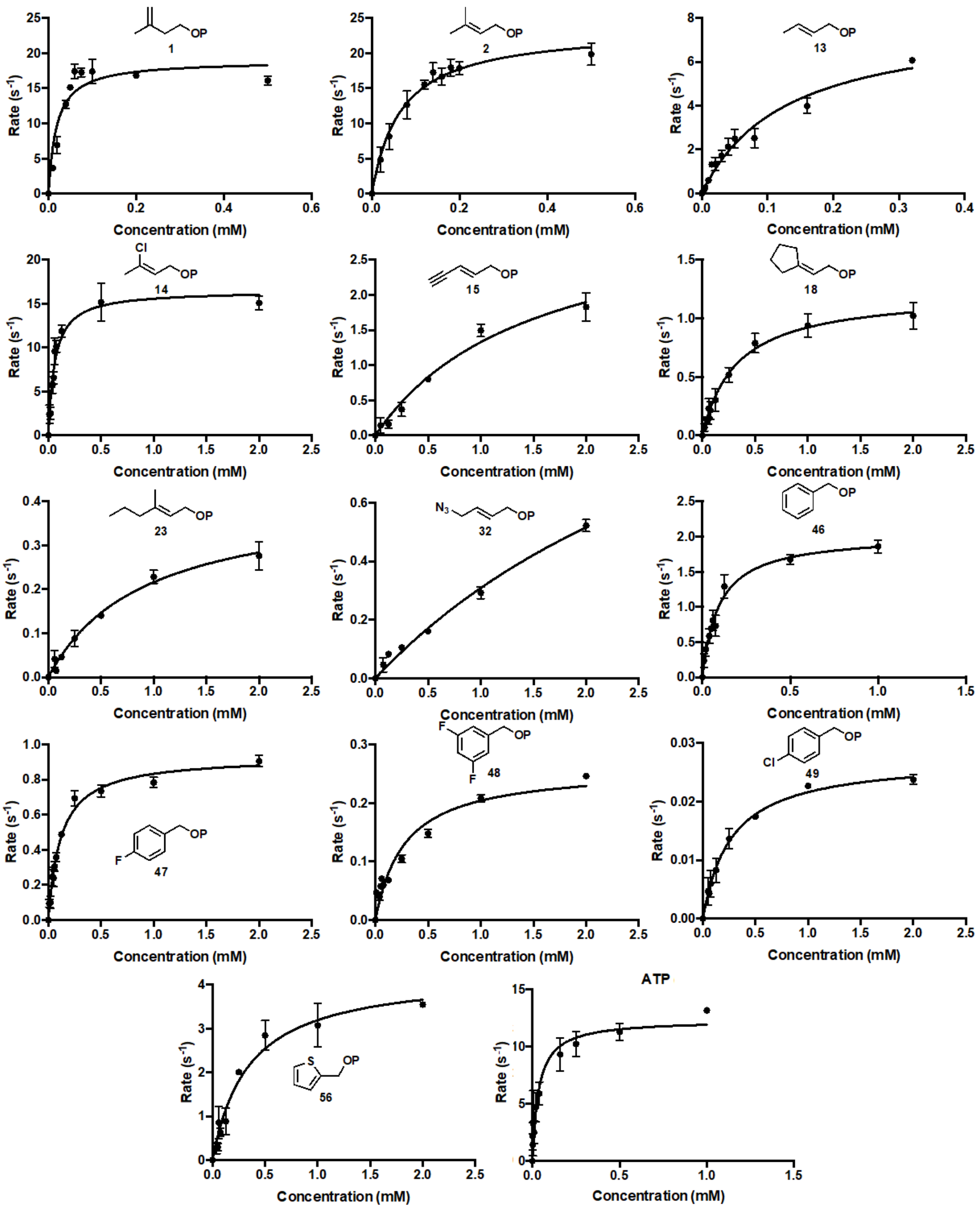

Figure S154. Michealis-Menten Curves for CMA.

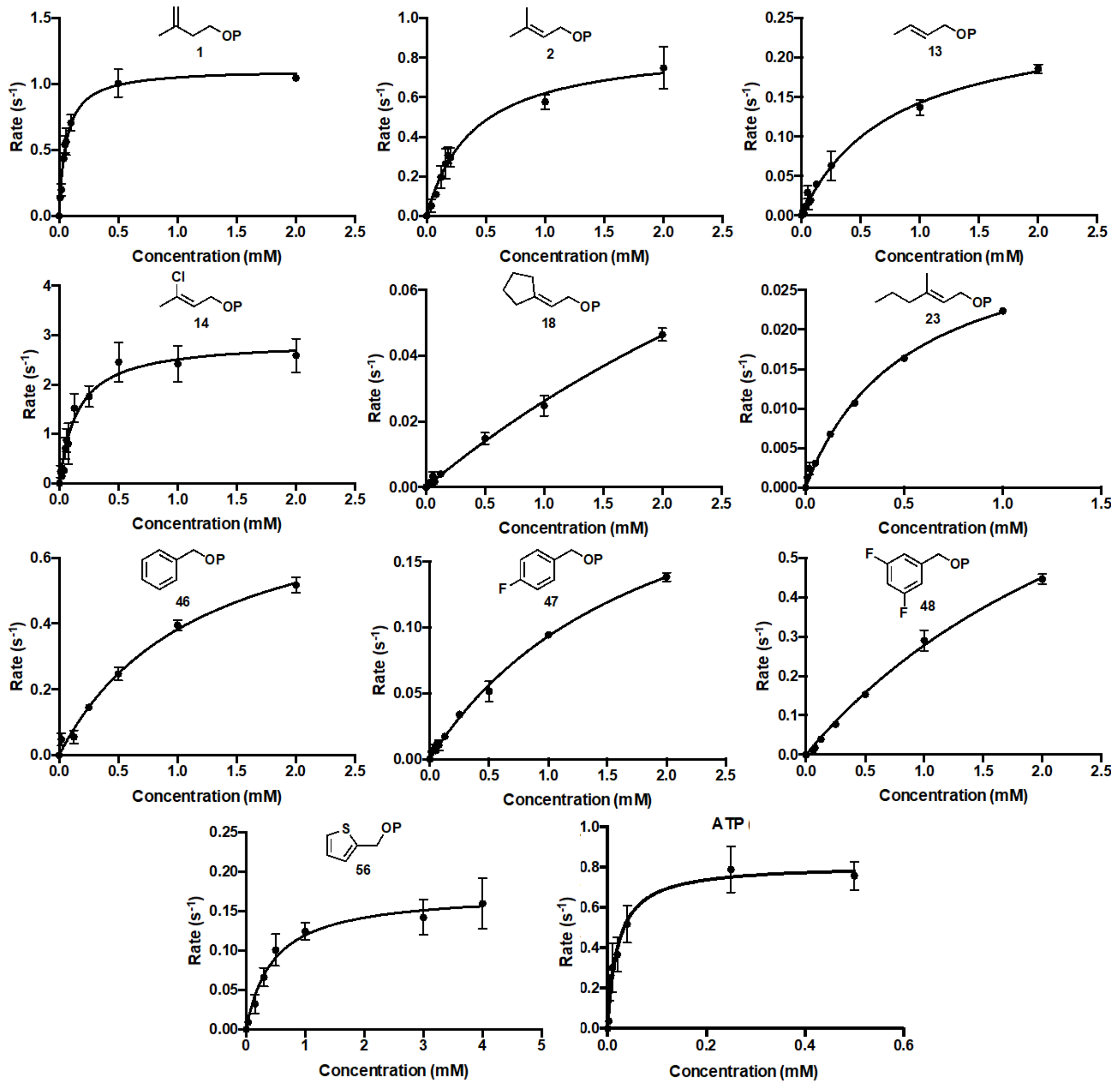

Figure S155. Michealis-Menten Curves for CNG.

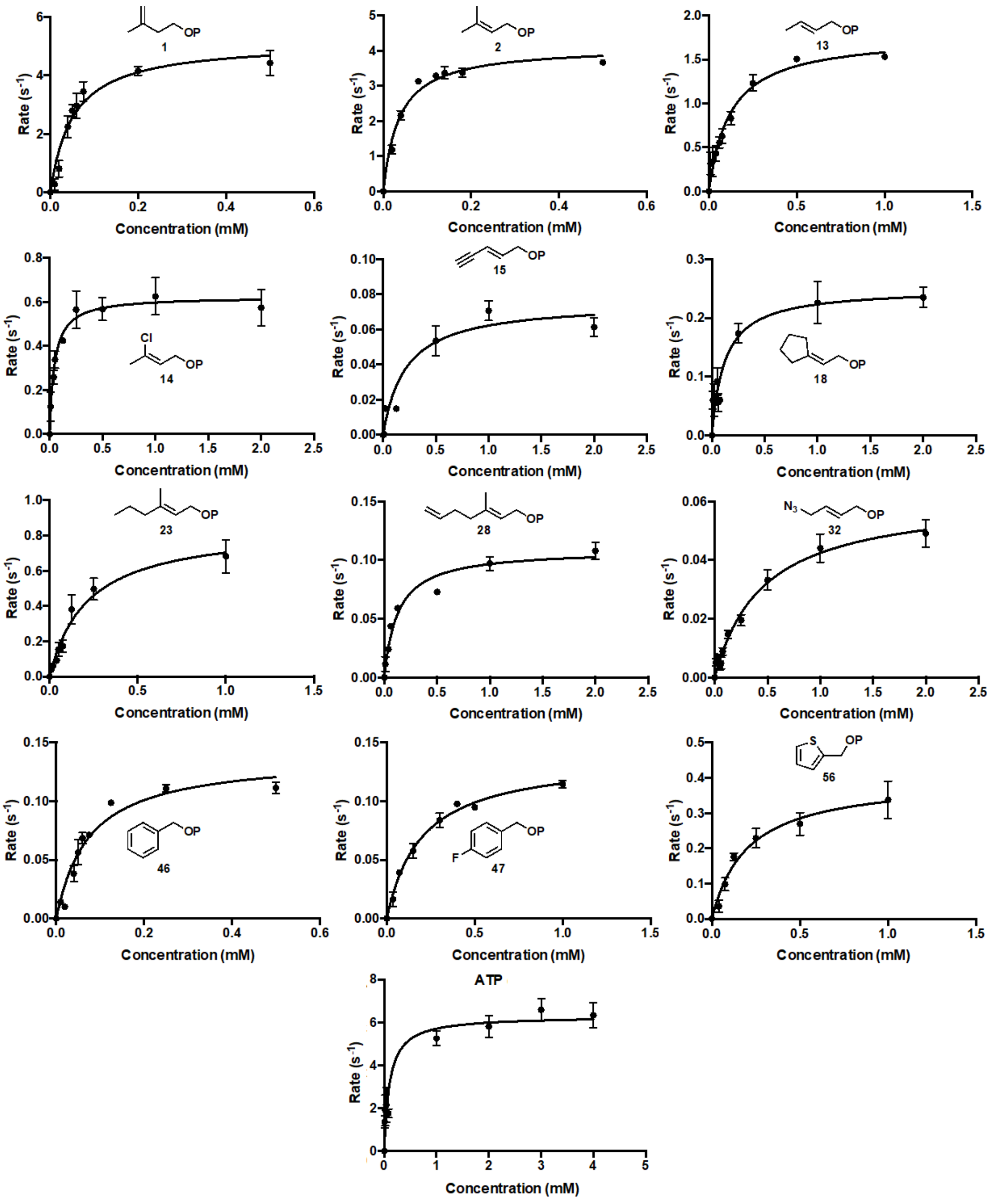

Figure S156. Michealis-Menten Curves for MHM.

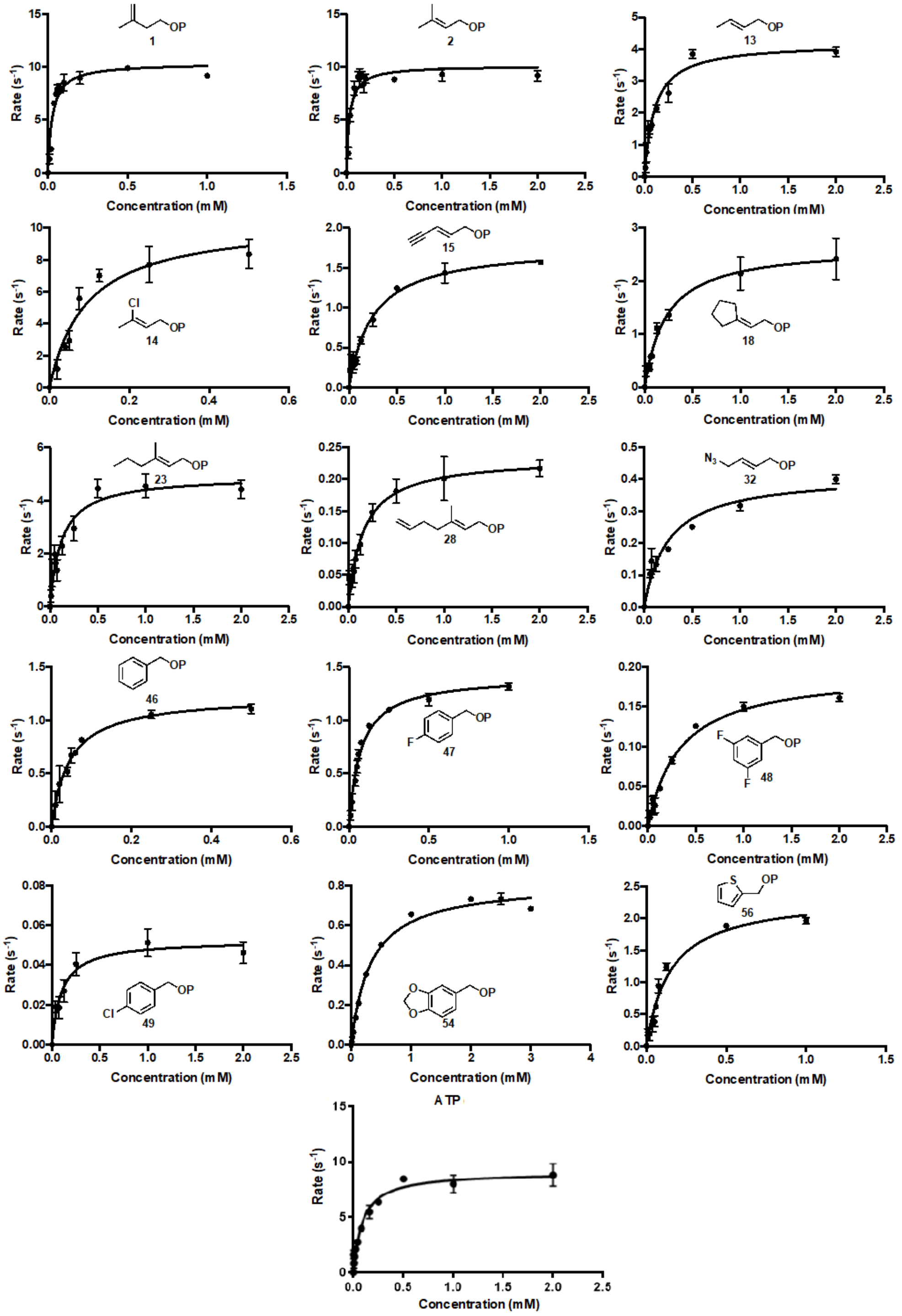

Figure S157. Michealis-Menten Curves for MSB.

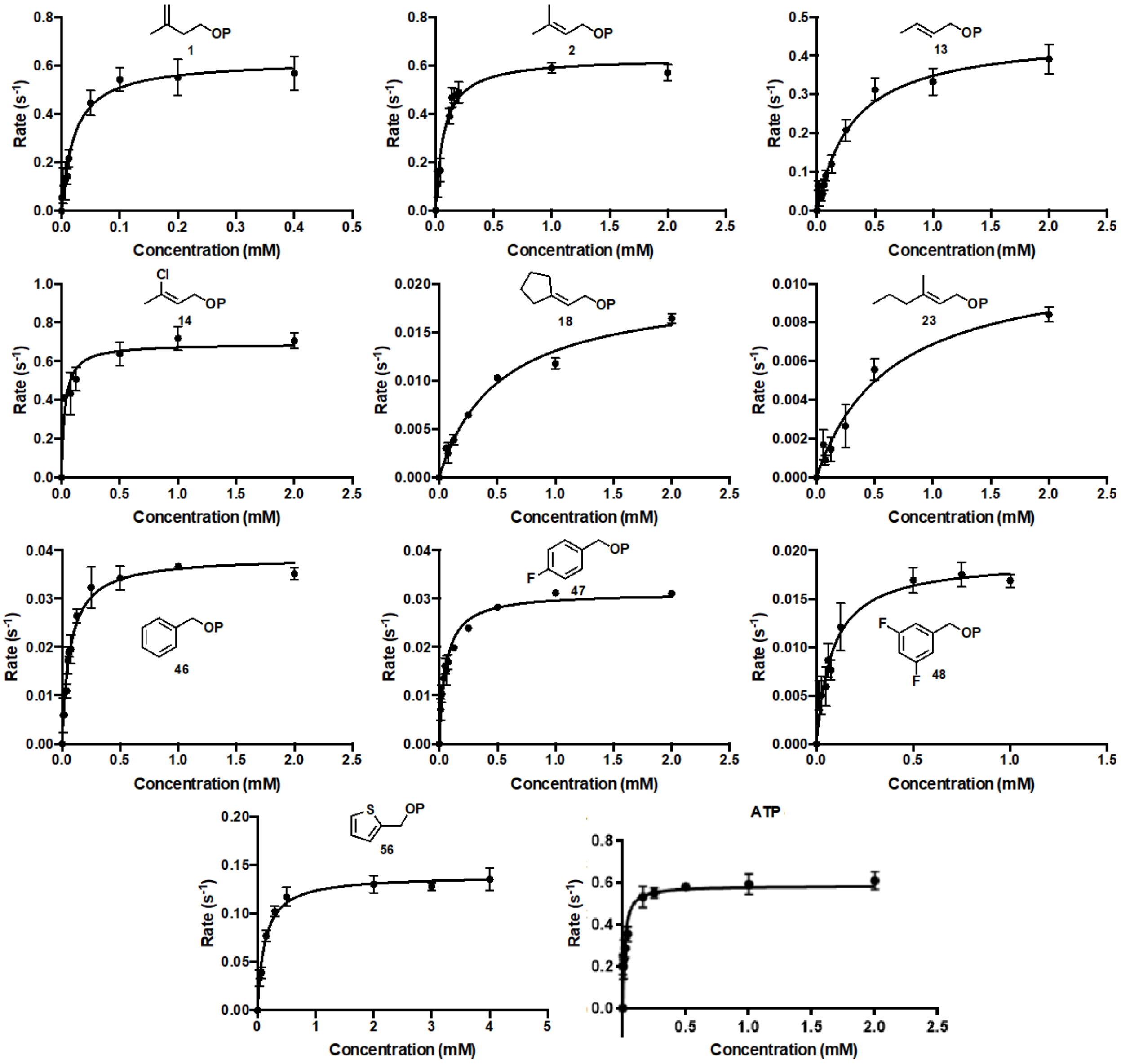

Figure S158. Michealis-Menten Curves for TCP.

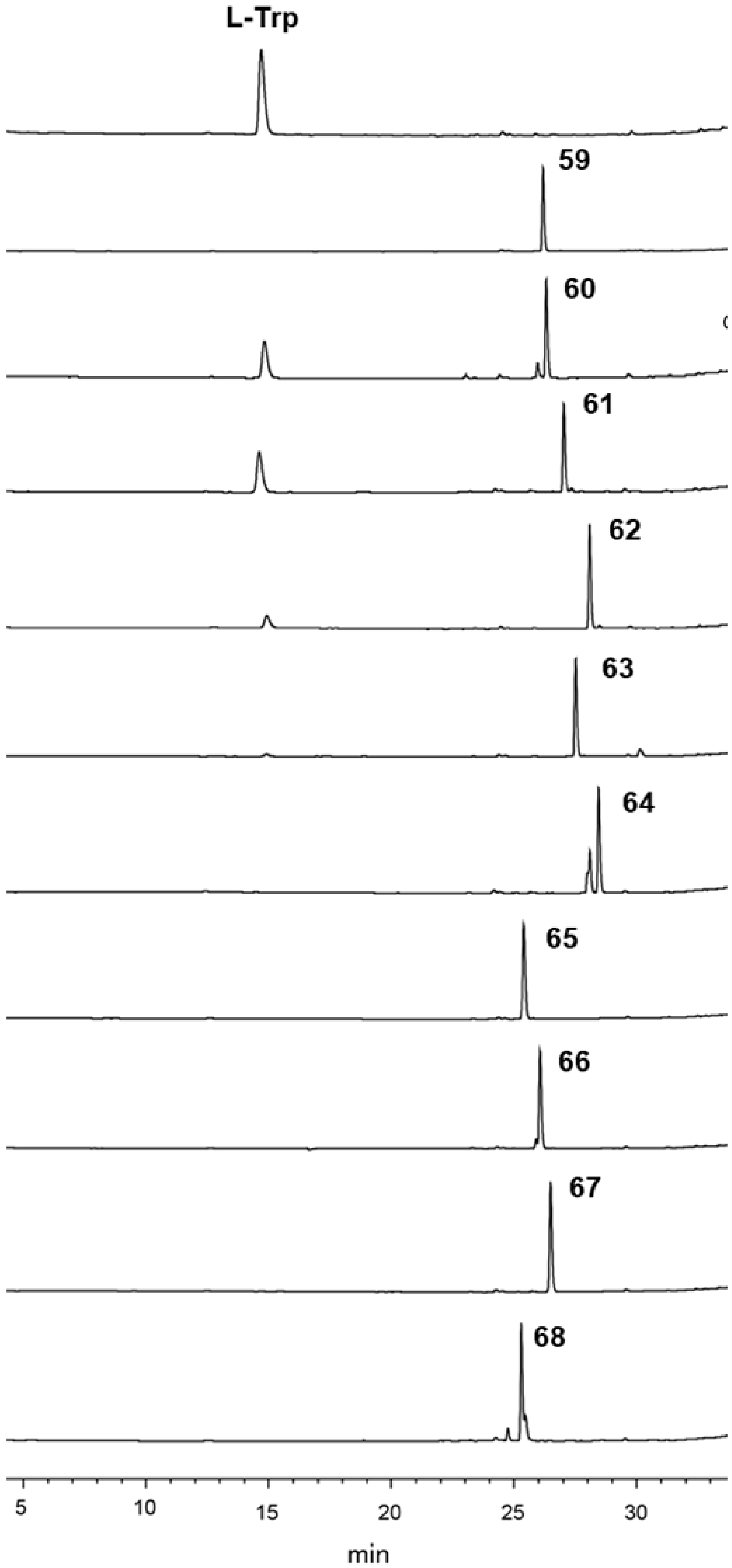

Figure S159. HPLC Traces of MSB-FgaPT2 Coupled Reactions. Peaks corresponding to alkylated products are labeled with product numbers.

Table S8. HRMS Confirmation of MSB-FgaPT2 Coupled Reactions.

Product	Alkyl-P	Chemical Formula	Predicted Mass (Da)	Observed Mass (Da)	Error (ppm)
**59**	**2**	$C_{16}H_{21}N_2O_2$ [M+H]$^+$	273.1603	273.1609	2.2
**60**	**14**	$C_{15}H_{17}ClN_2O_2$ [M+H]$^+$	293.1057	293.1050	2.3
**61**	**16**	$C_{17}H_{23}N_2O_2$ [M+H]$^+$	287.1759	287.1759	0.14
**62**	**17**	$C_{18}H_{25}N_2O_2$ [M+H]$^+$	301.1916	301.1924	2.7
**63**	**18**	$C_{18}H_{22}N_2O_2$ [M+H]$^+$	299.1759	299.1768	2.9
**64**	**23**	$C_{18}H_{25}N_2O_2$ [M+H]$^+$	301.1916	301.1921	1.7
**65**	**32**	$C_{15}H_{18}N_5O_2$ [M+H]$^+$	300.1460	300.1465	1.6
**66**	**46**	$C_{18}H_{19}N_2O_2$ [M+H]$^+$	295.1446	295.1453	2.2
**67**	**47**	$C_{18}H_{18}FN_2O_2$ [M+H]$^+$	313.1352	313.1365	4.1
**68**	**56**	$C_{16}H_{16}N_2O_2S$ [M+H]$^+$	301.1011	301.1012	0.46

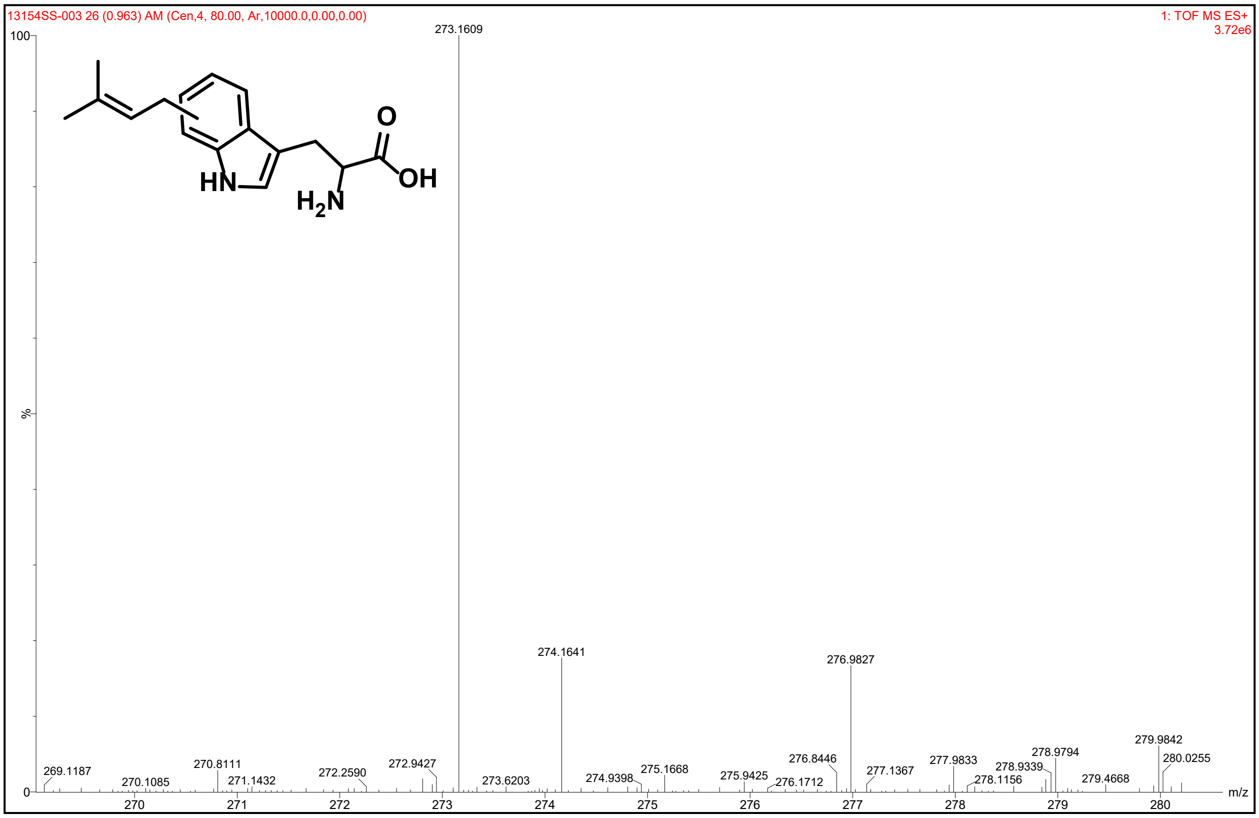

Figure S160. HRMS-ESI$^+$ of **59.**

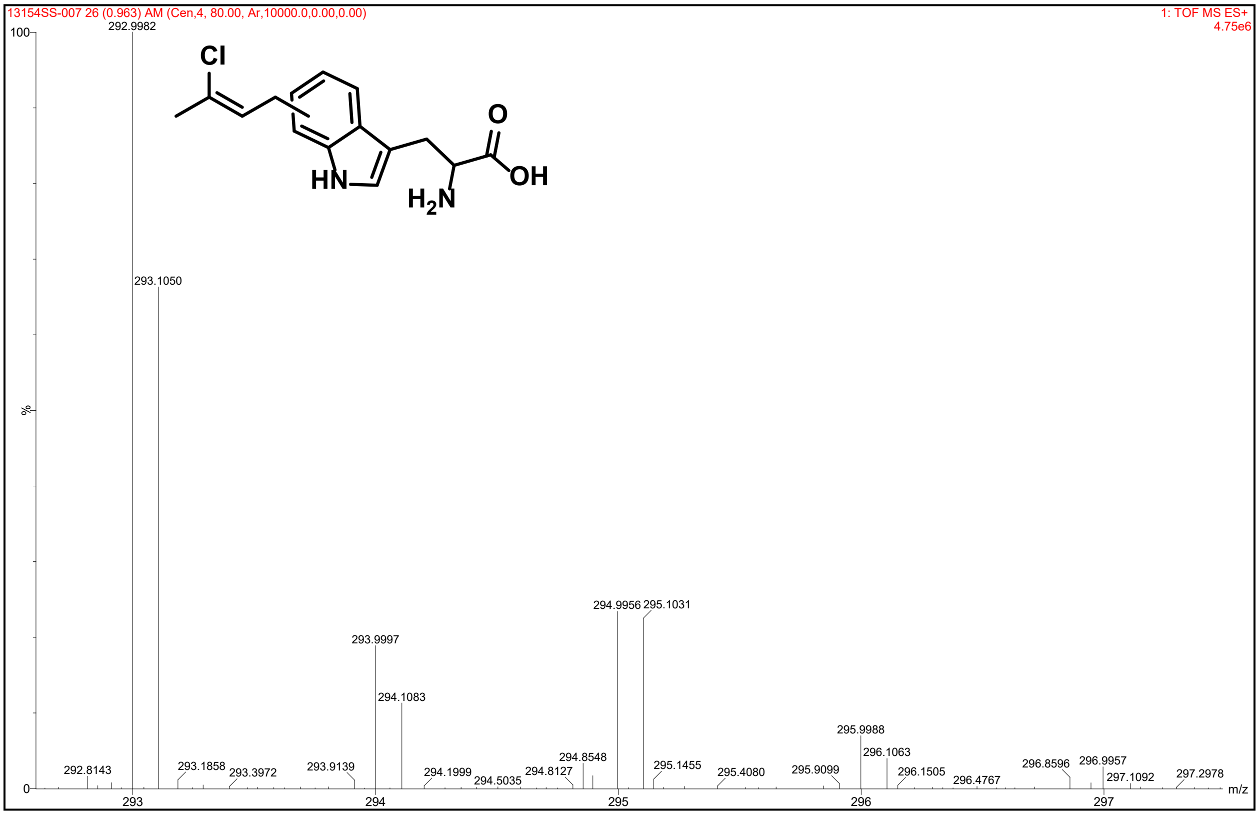

Figure S161. HRMS-ESI$^+$ of **60.**

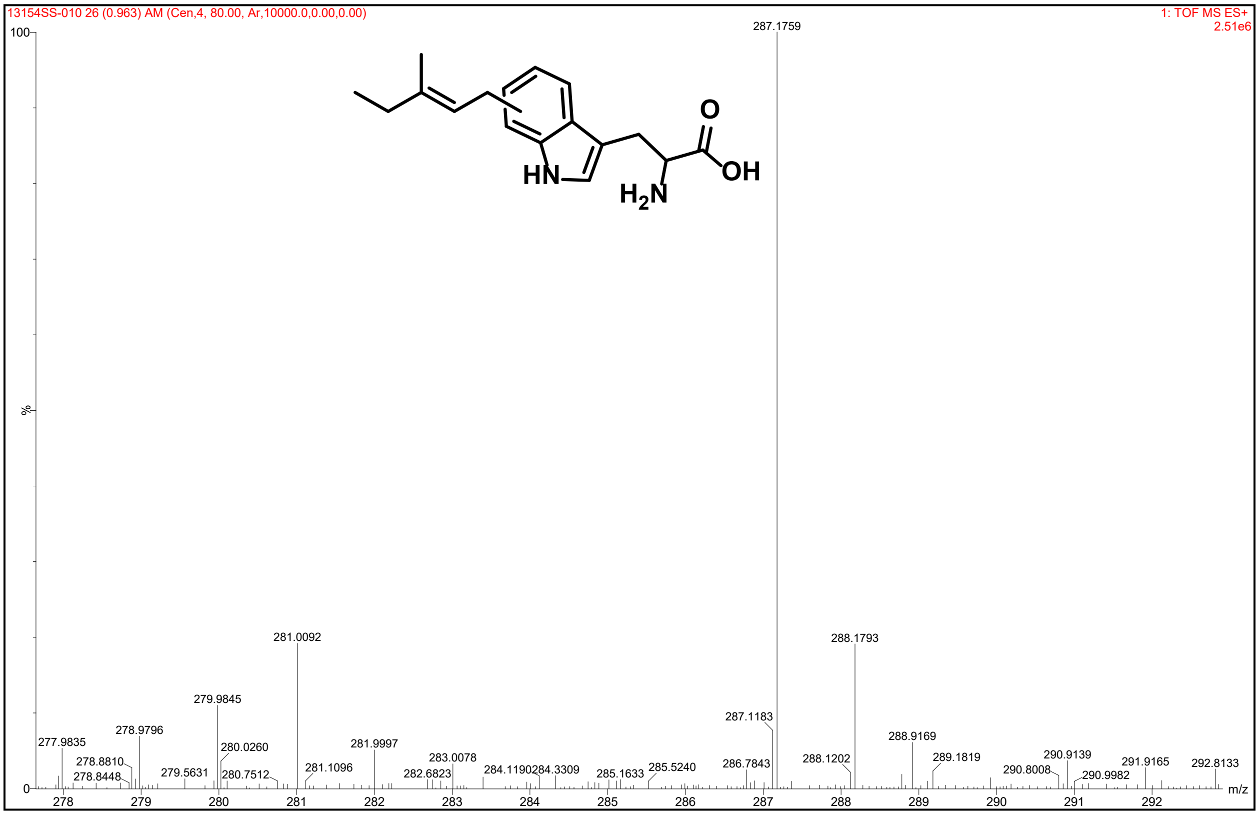

Figure S162. HRMS-ESI$^+$ of **61.**

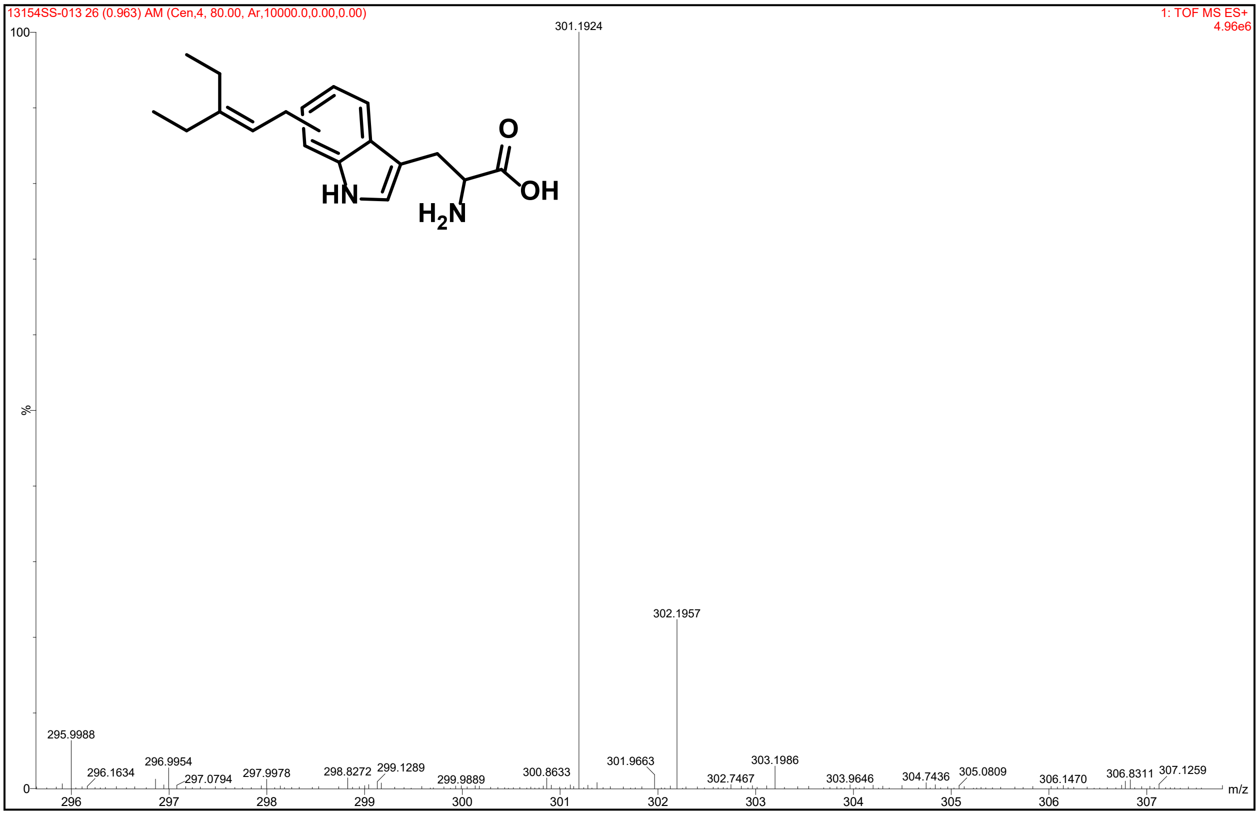

Figure S163. HRMS-ESI$^+$ of **62.**

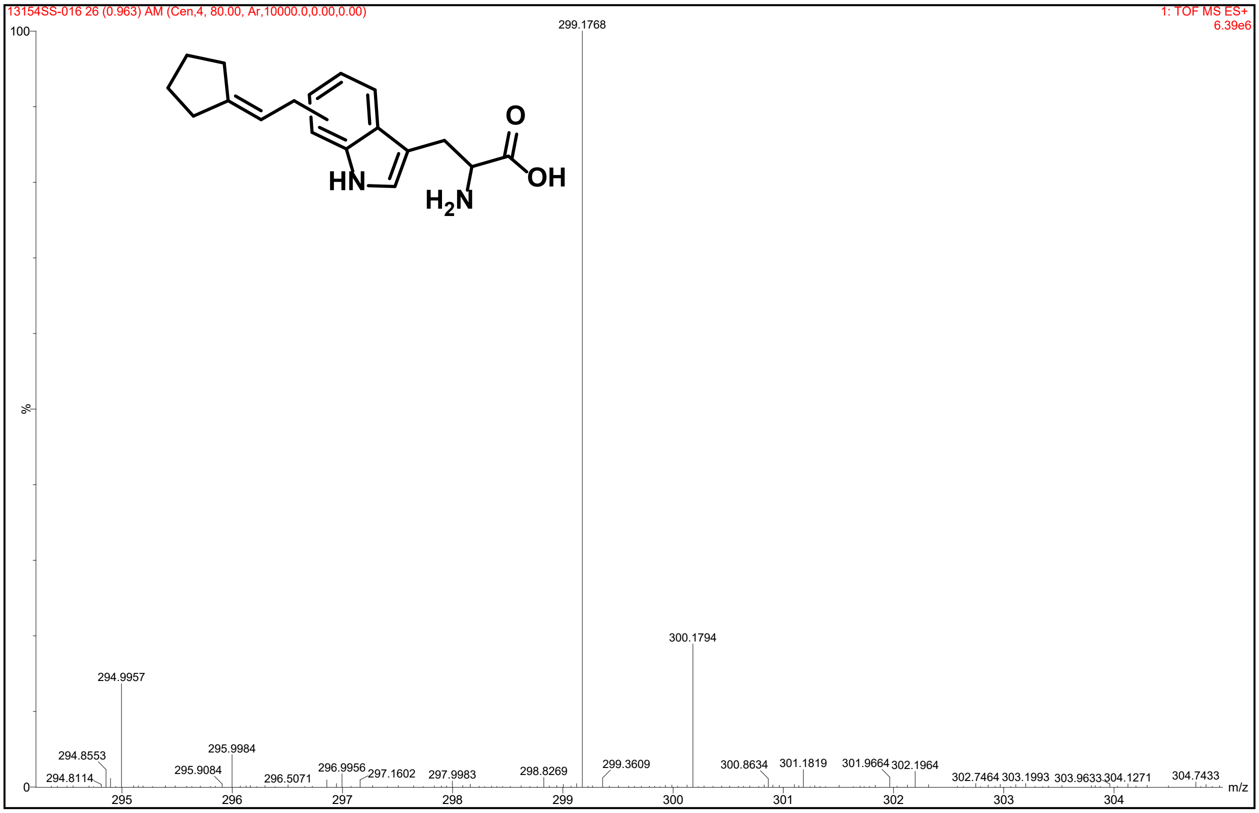

Figure S164. HRMS-ESI$^+$ of **63.**

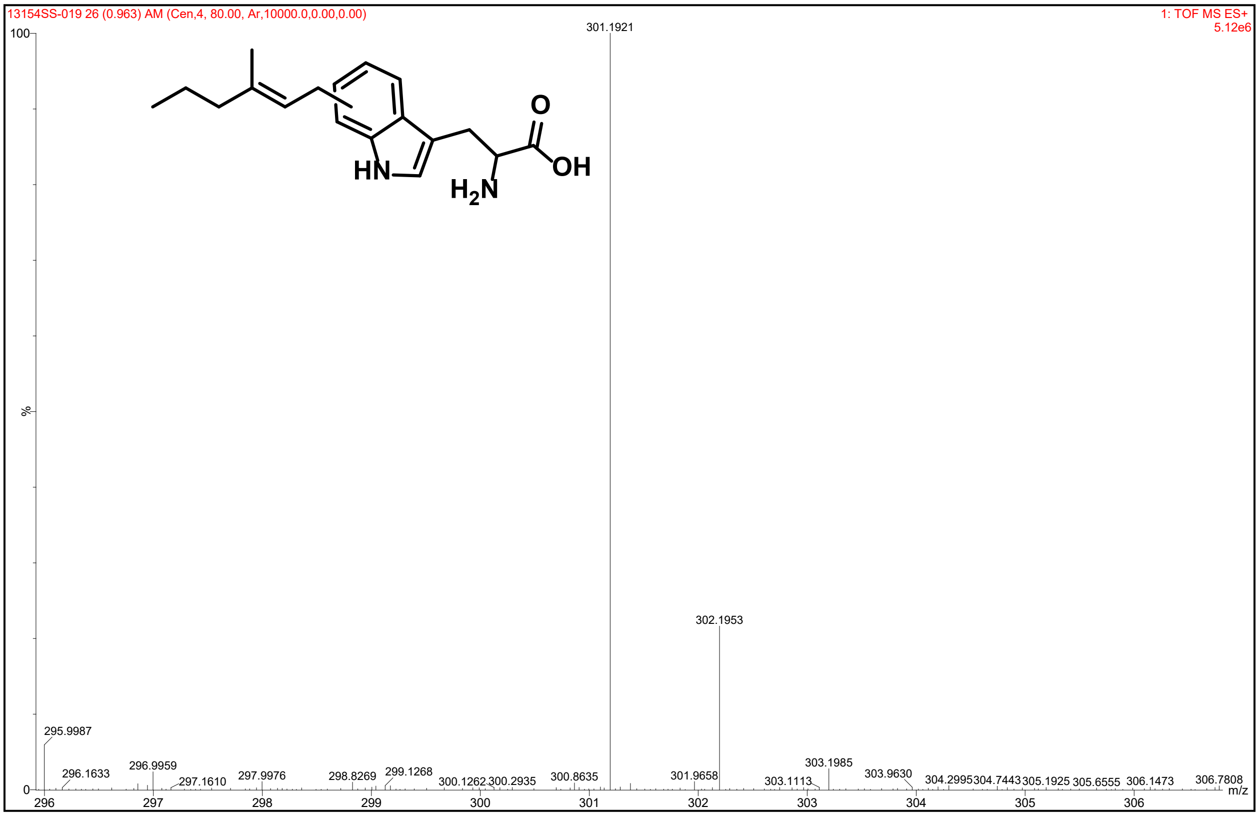

Figure S165. HRMS-ESI$^+$ of **64.**

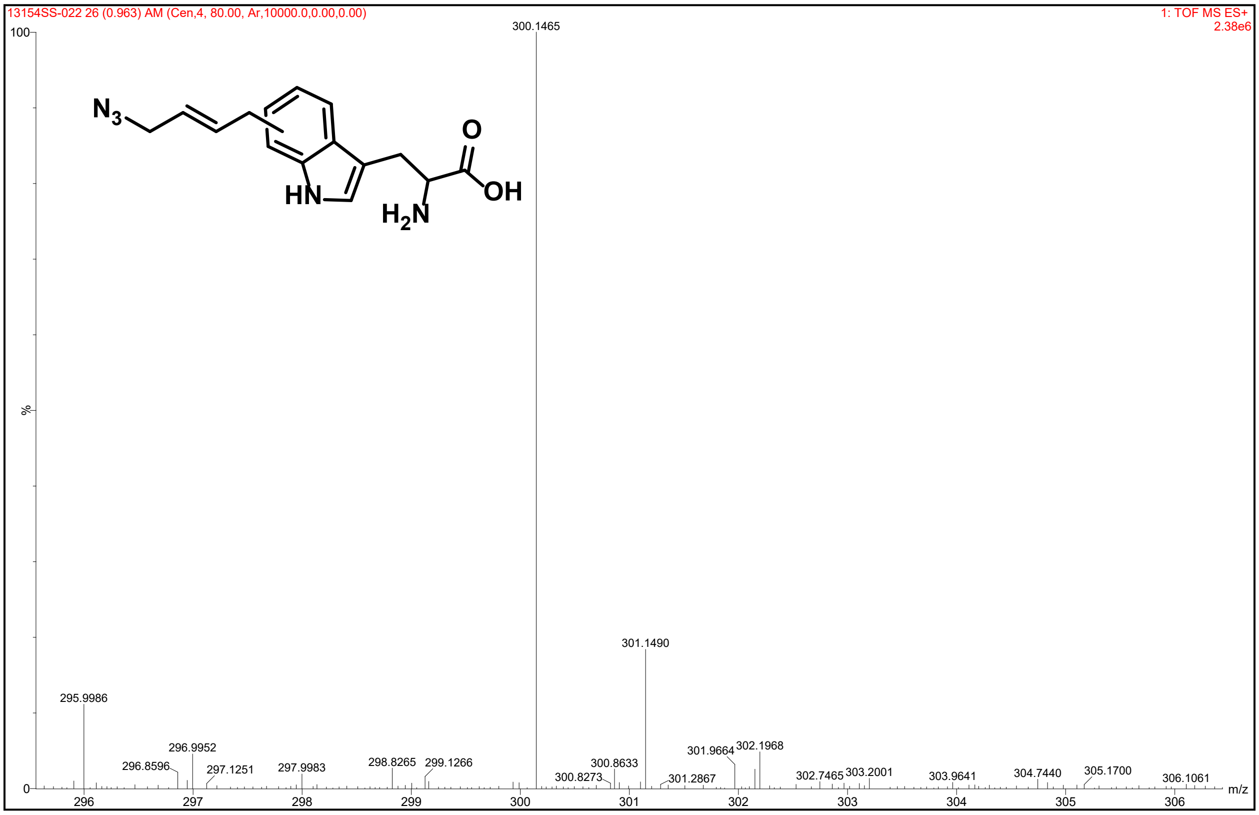

Figure S166. HRMS-ESI$^+$ of **65.**

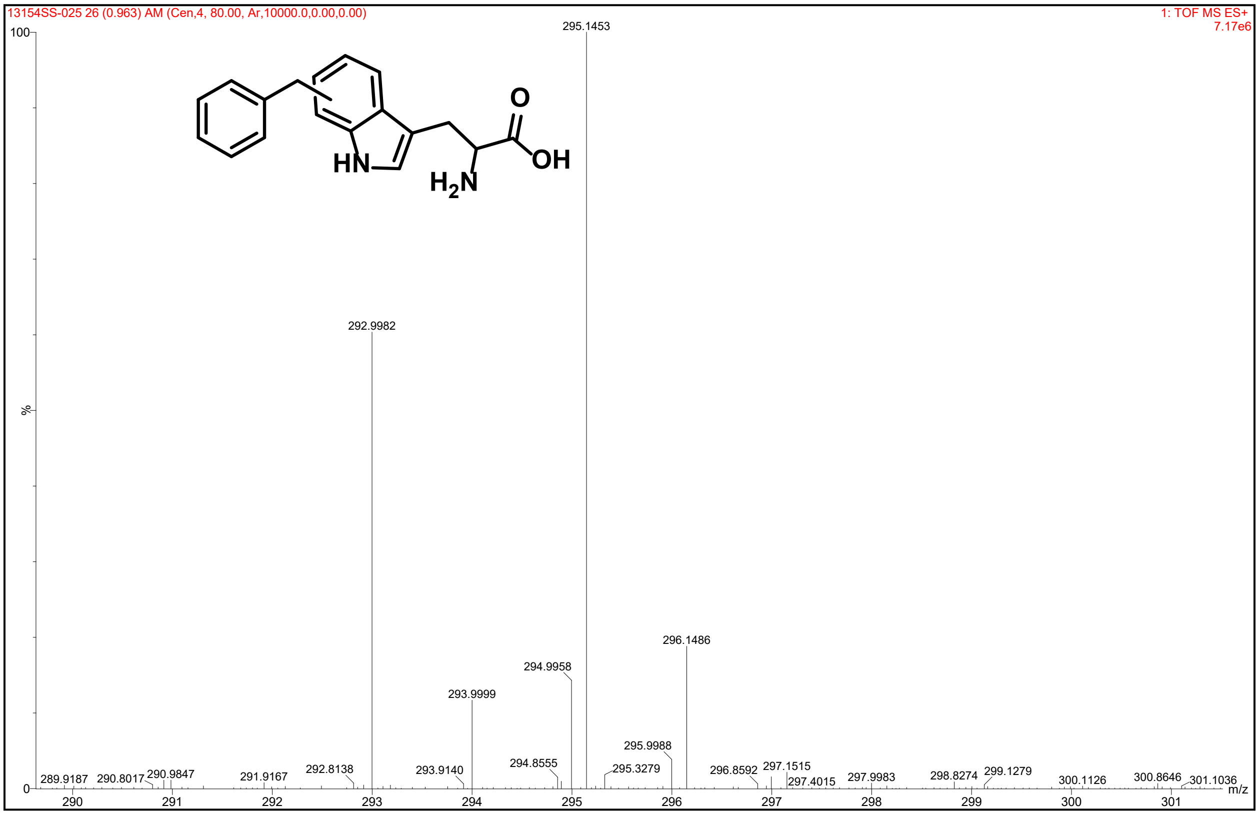

Figure S167. HRMS-ESI+ of **66.**

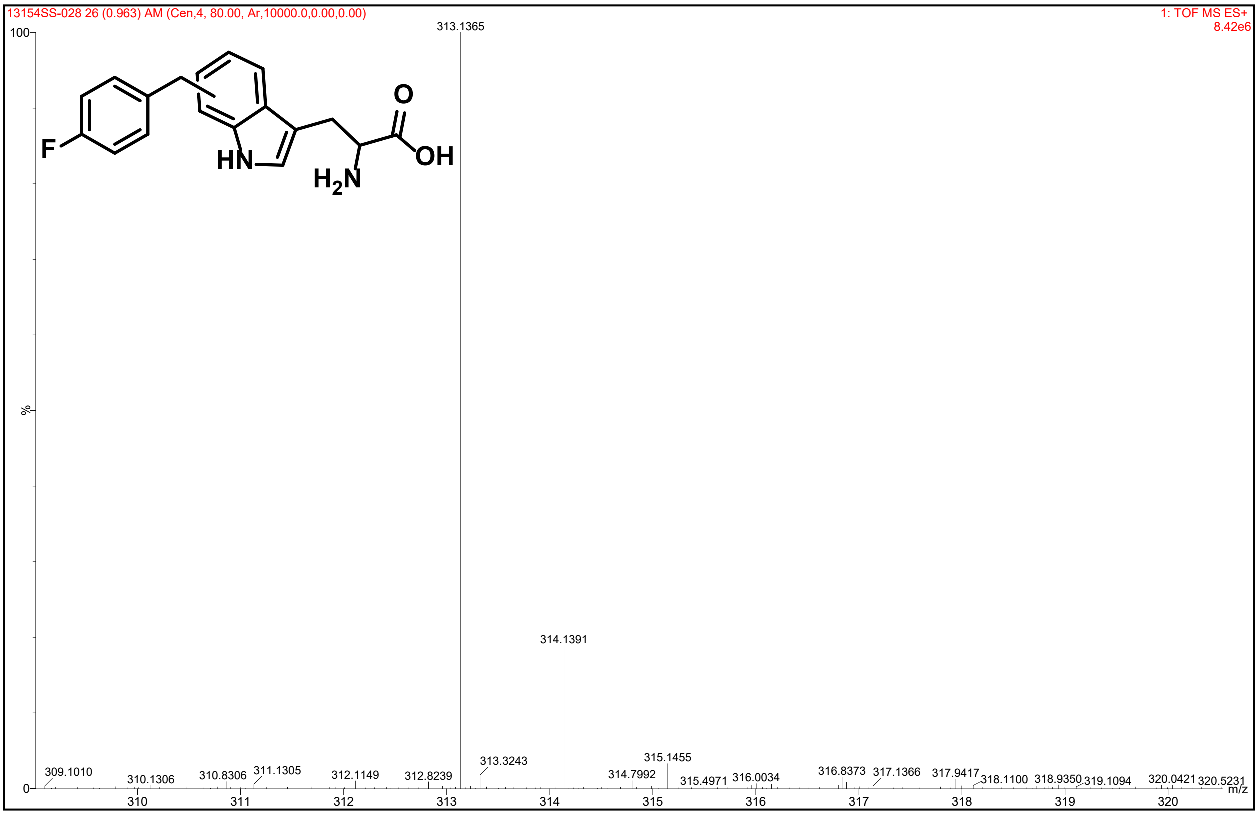

Figure S168. HRMS-ESI$^+$ of **67.**

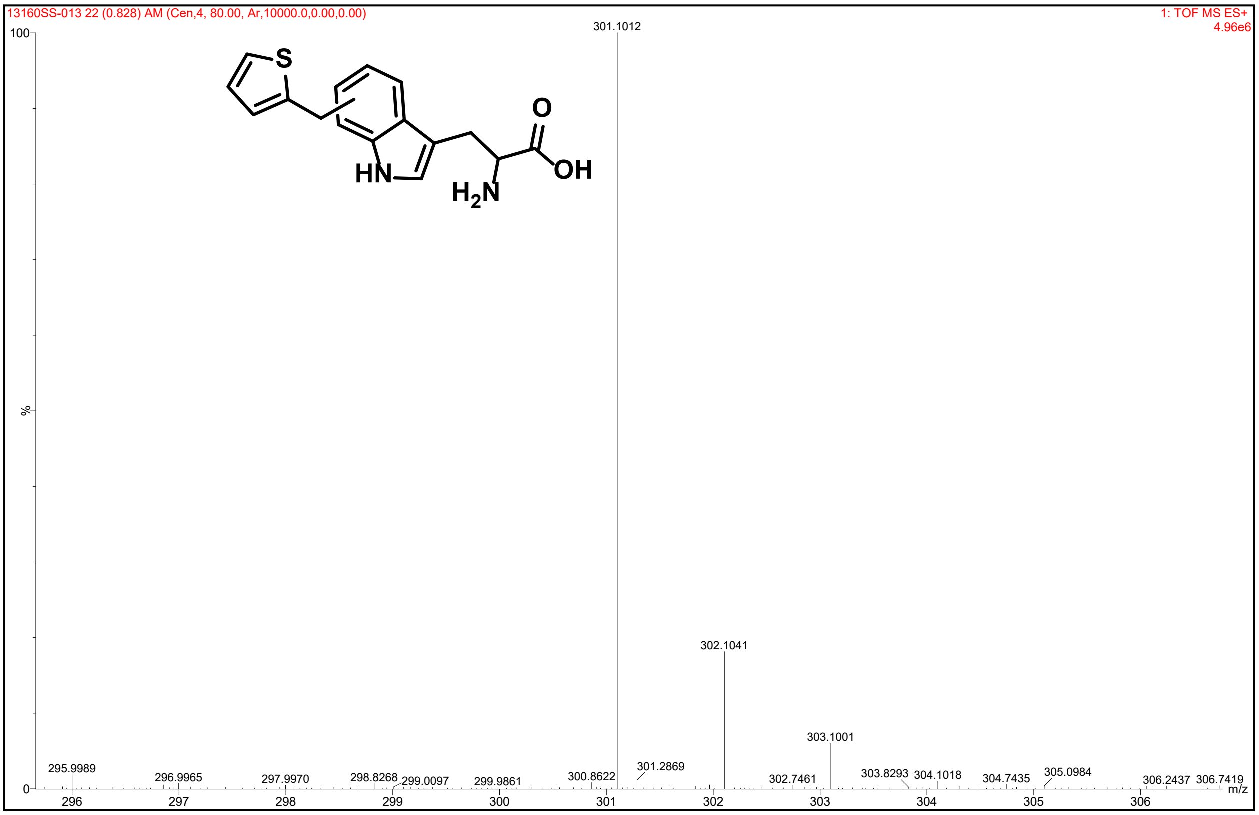

Figure S169. HRMS-ESI^{+} of **68.**

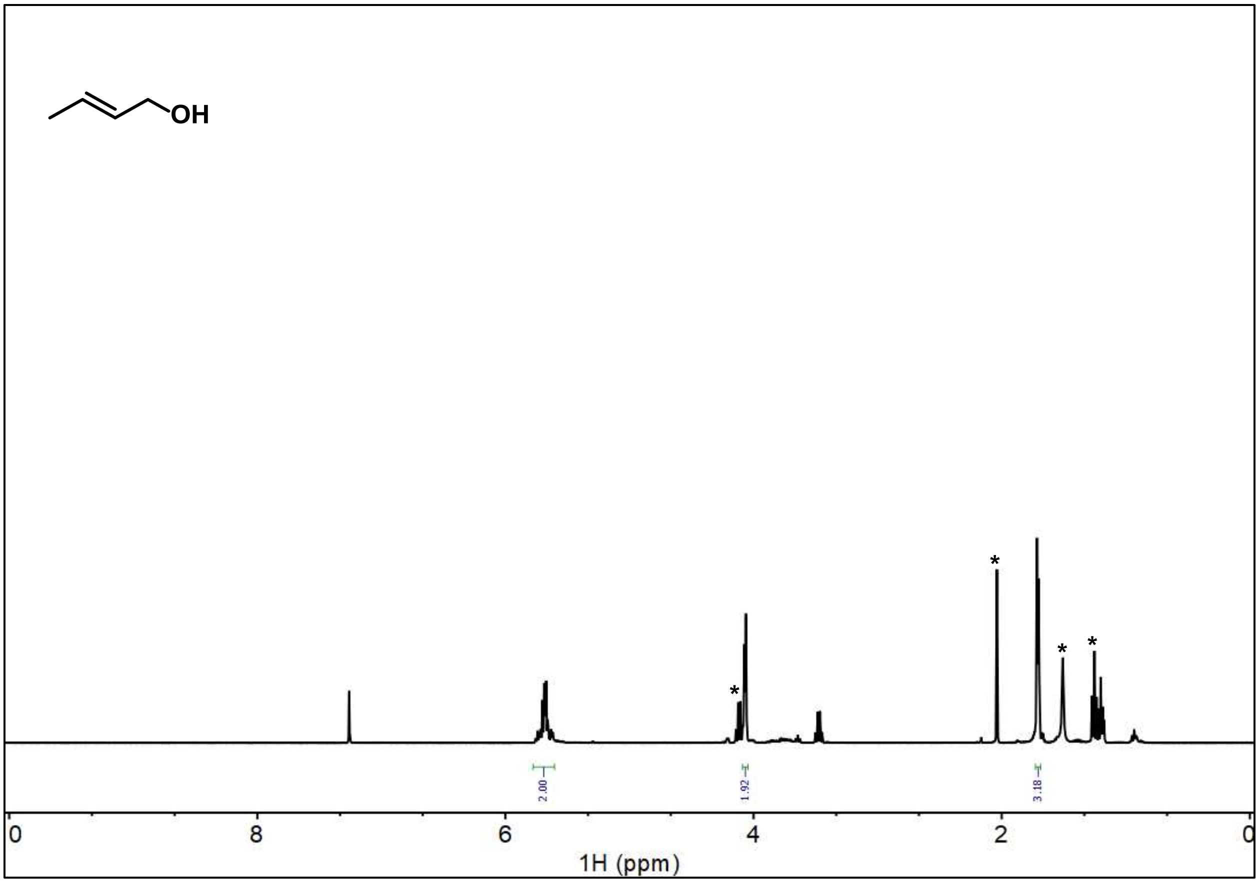

Figure S170. ^{1}H NMR of **(*E*)-but-2-en-1-ol** (400 MHz, $CDCl_3$)**.** [*EtOAc and H_2O]

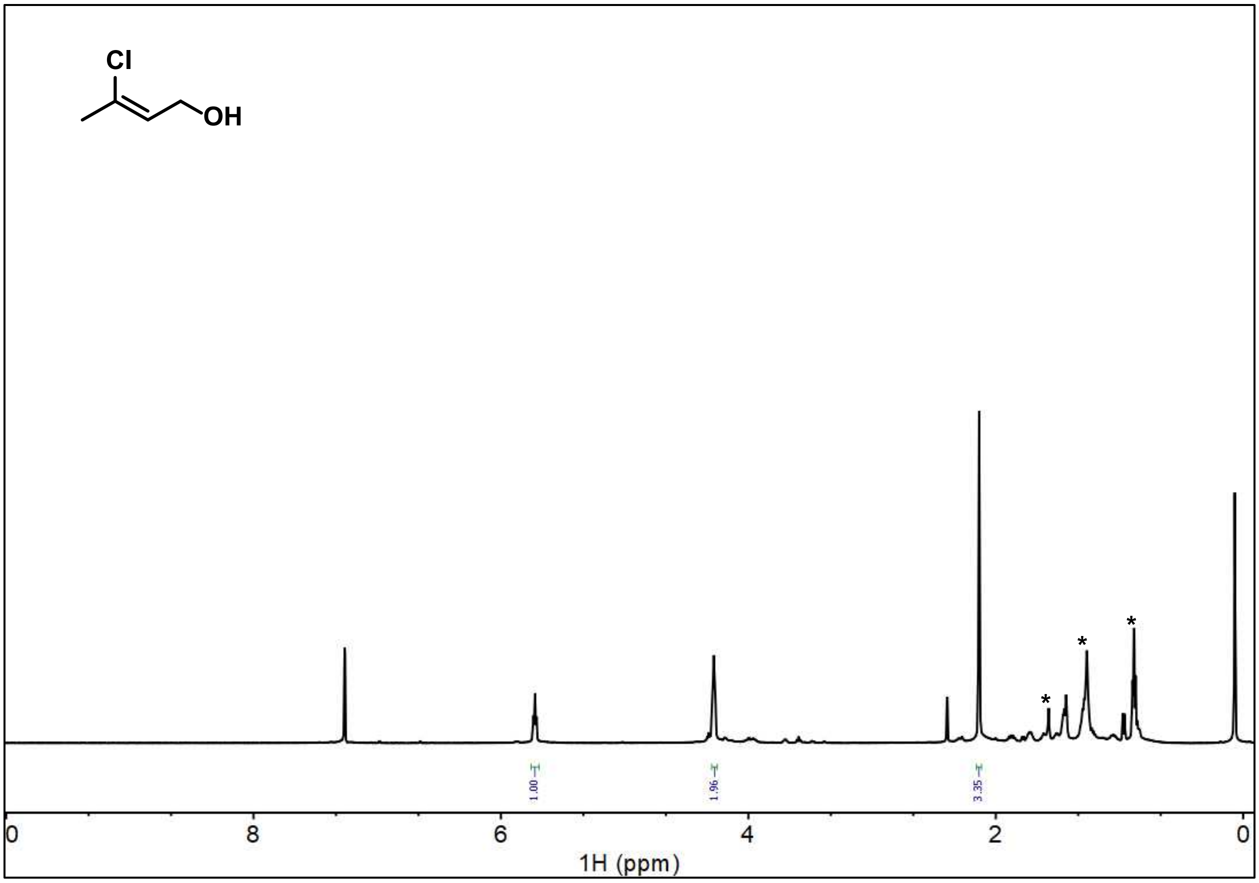

Figure S171. ^{1}H NMR of **(*Z*)-3-chlorobut-2-en-1-ol** (500 MHz, $CDCl_3$)**.** [*Hexanes and H_2O]

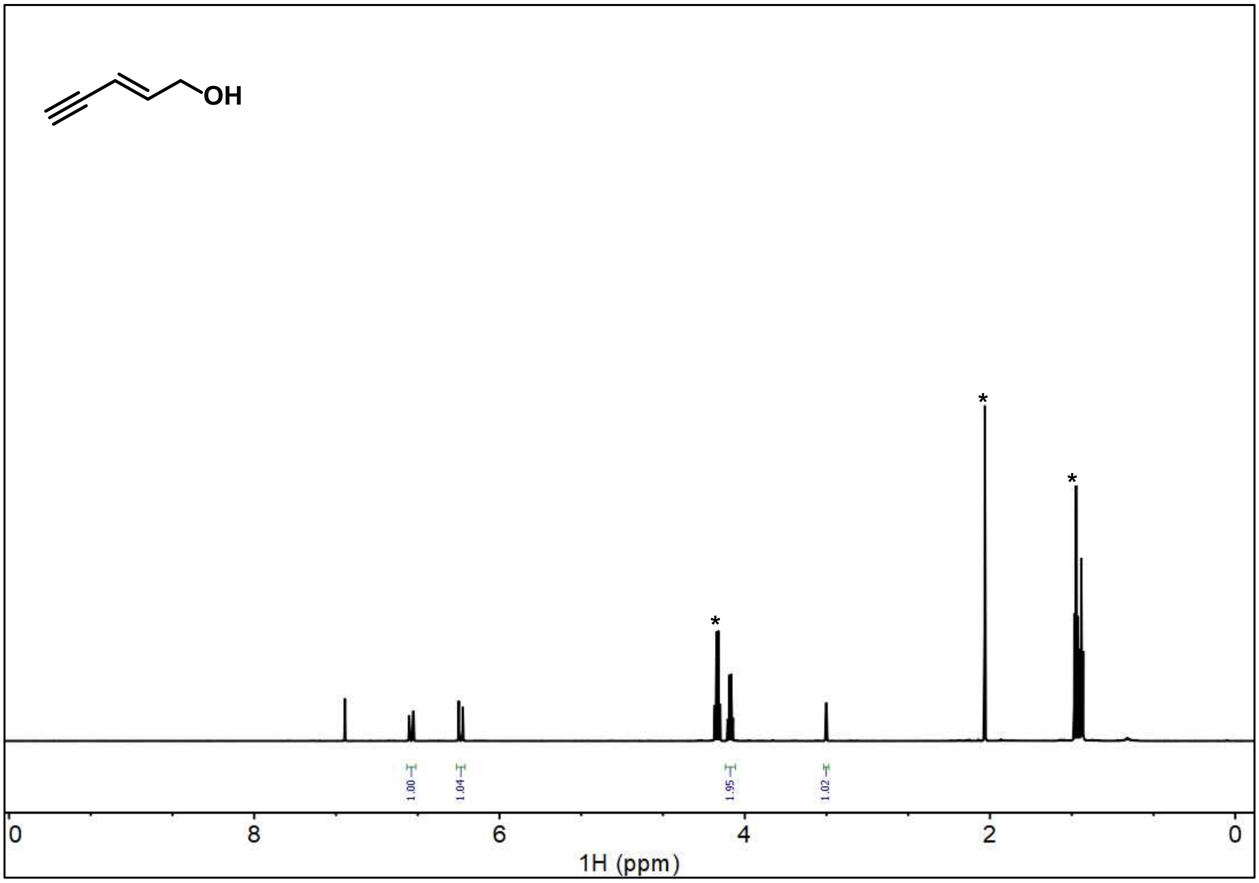

Figure S172. ^{1}H NMR of **(*E*)-pent-2-en-4-yn-1-ol** (500 MHz, $CDCl_3$)**.** [*EtOAc]

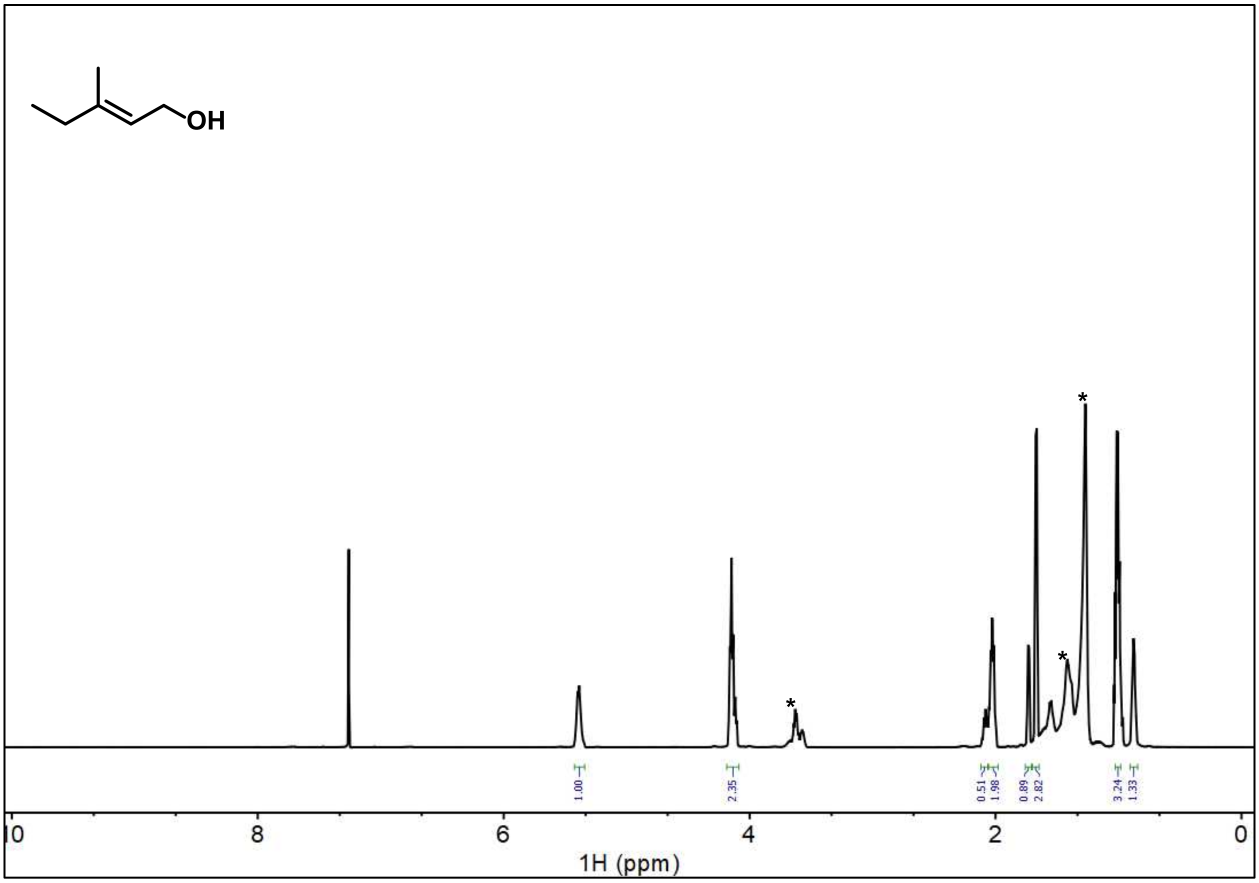

Figure S173. ^{1}H NMR of **(*E*)-3-methylpent-2-en-1-ol** (500 MHz, $CDCl_3$)**.** [*EtOAc and H_2O]

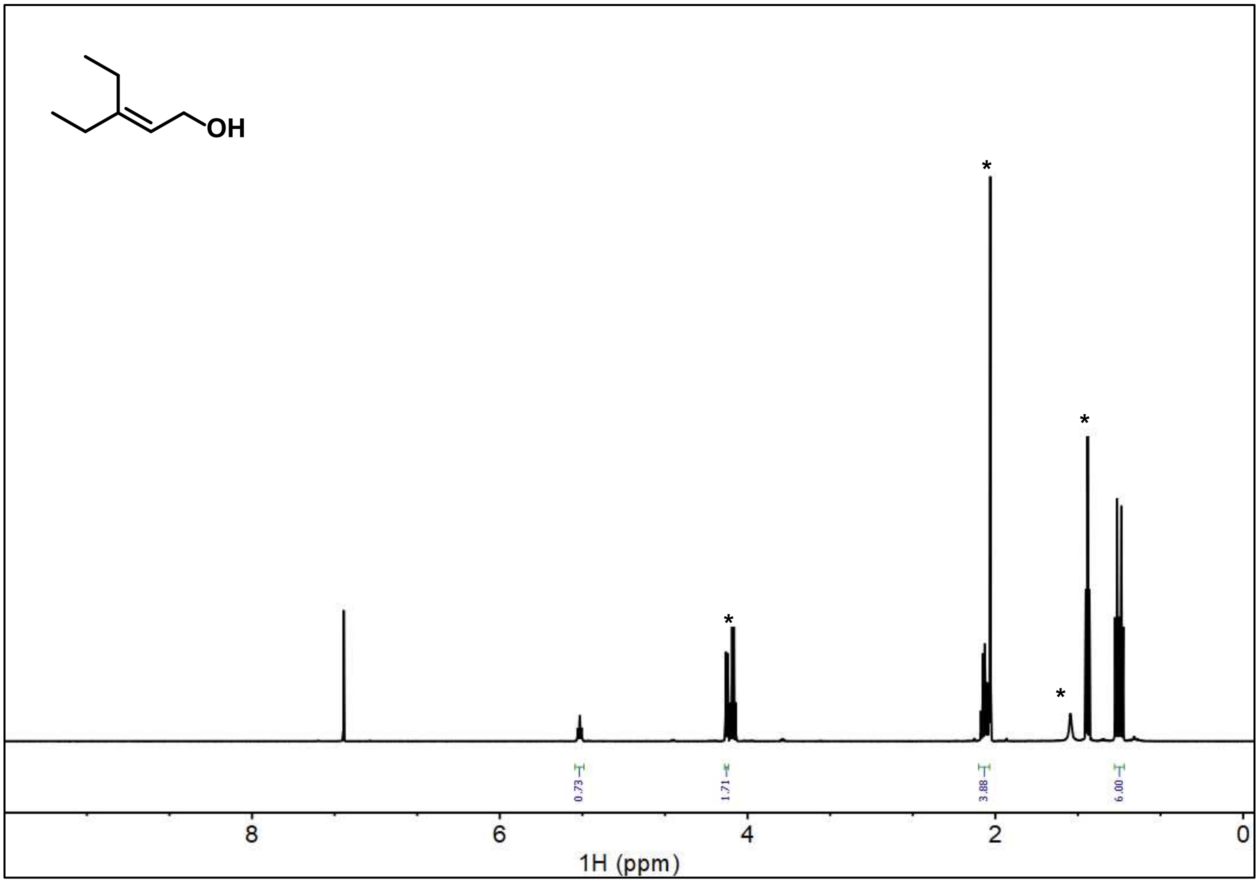

Figure S174. ^{1}H NMR of **3-ethylpent-2-en-1-ol** (500 MHz, $CDCl_3$). [*EtOAc and H_2O]

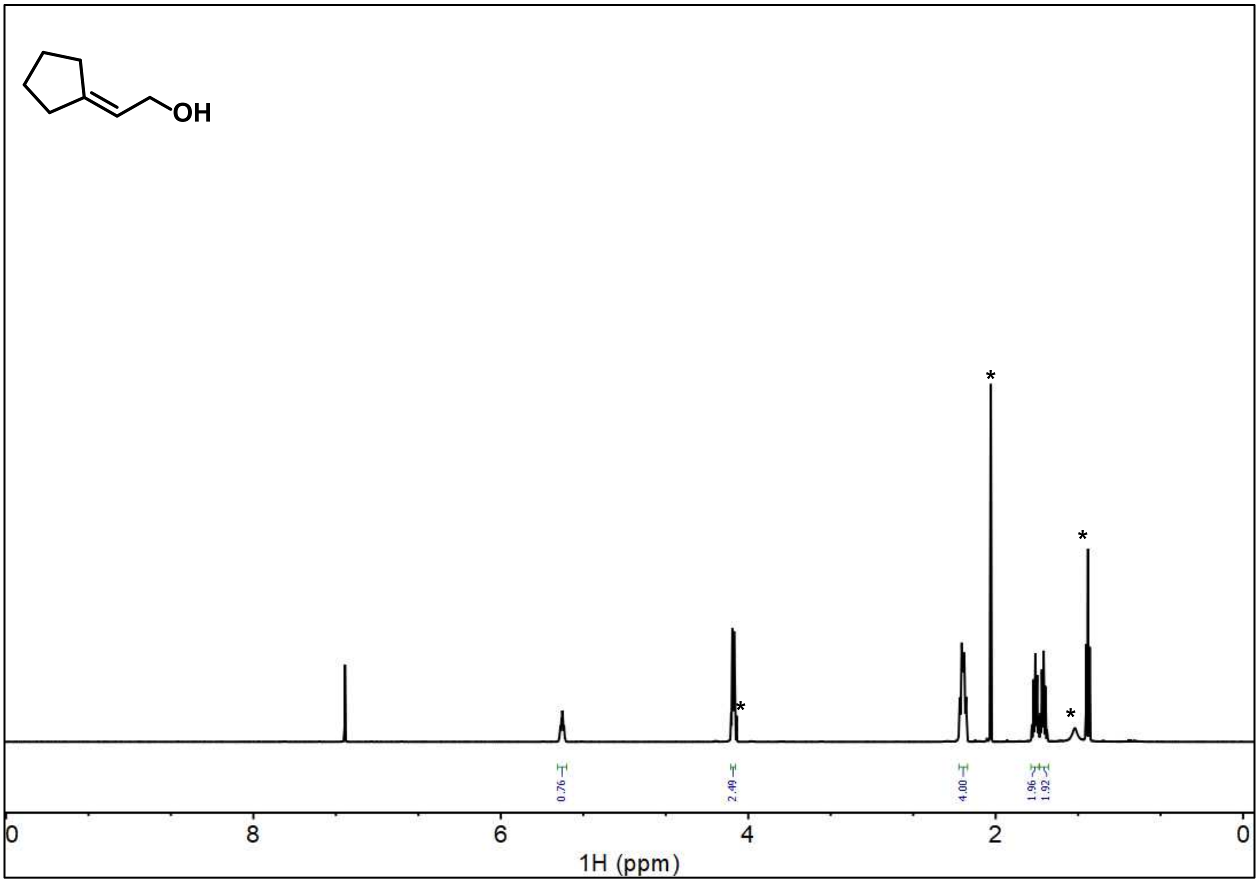

Figure S175. ^{1}H NMR of **2-cyclopentylideneethan-1-ol** (500 MHz, $CDCl_3$)**.** [*EtOAc and H_2O]

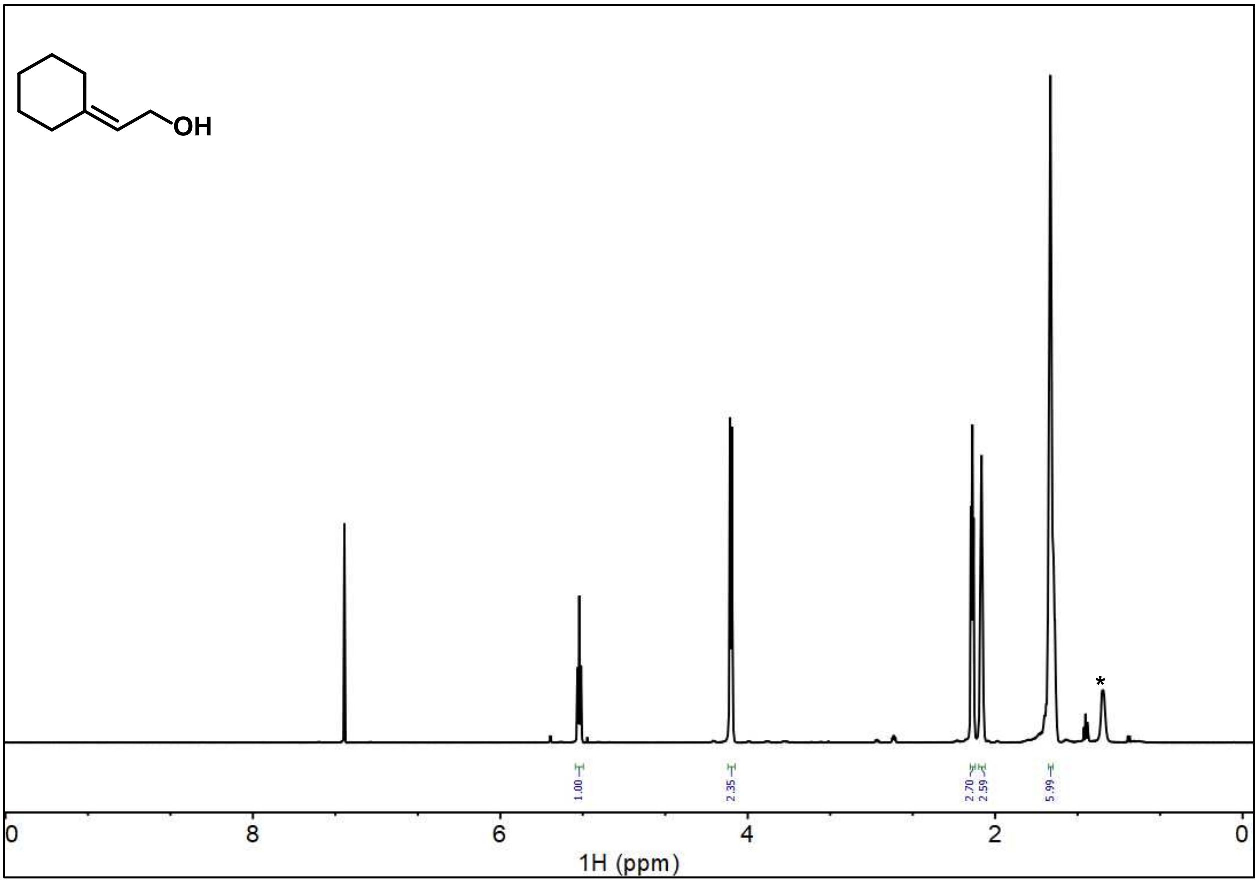

Figure S176. ^{1}H NMR of **2-cyclohexylideneethan-1-ol** (500 MHz, $CDCl_3$)**.** [*H_2O]

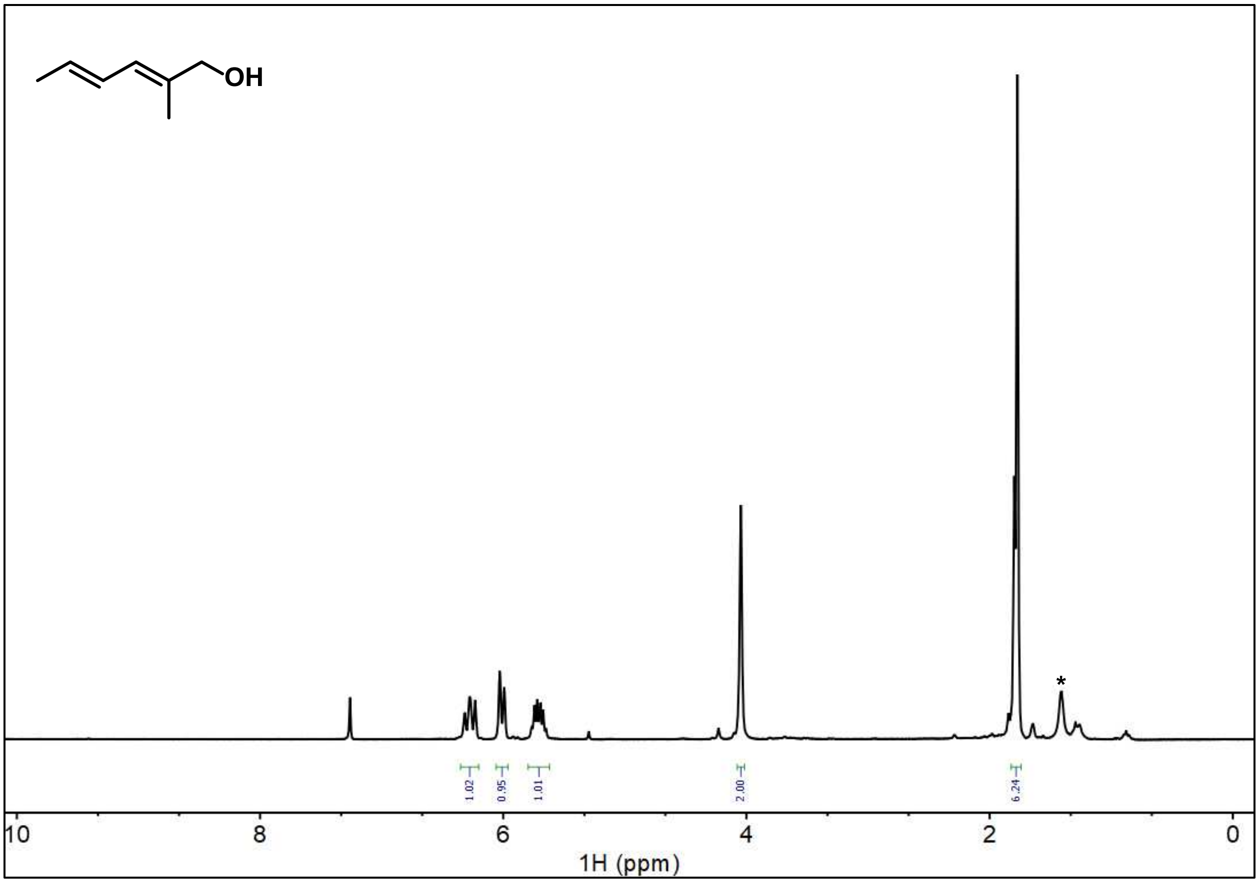

Figure S177. ^{1}H NMR of **(2*E*,4*E*)-2-methylhexa-2,4-dien-1-ol** (300 MHz, $CDCl_3$). [*H_2O]

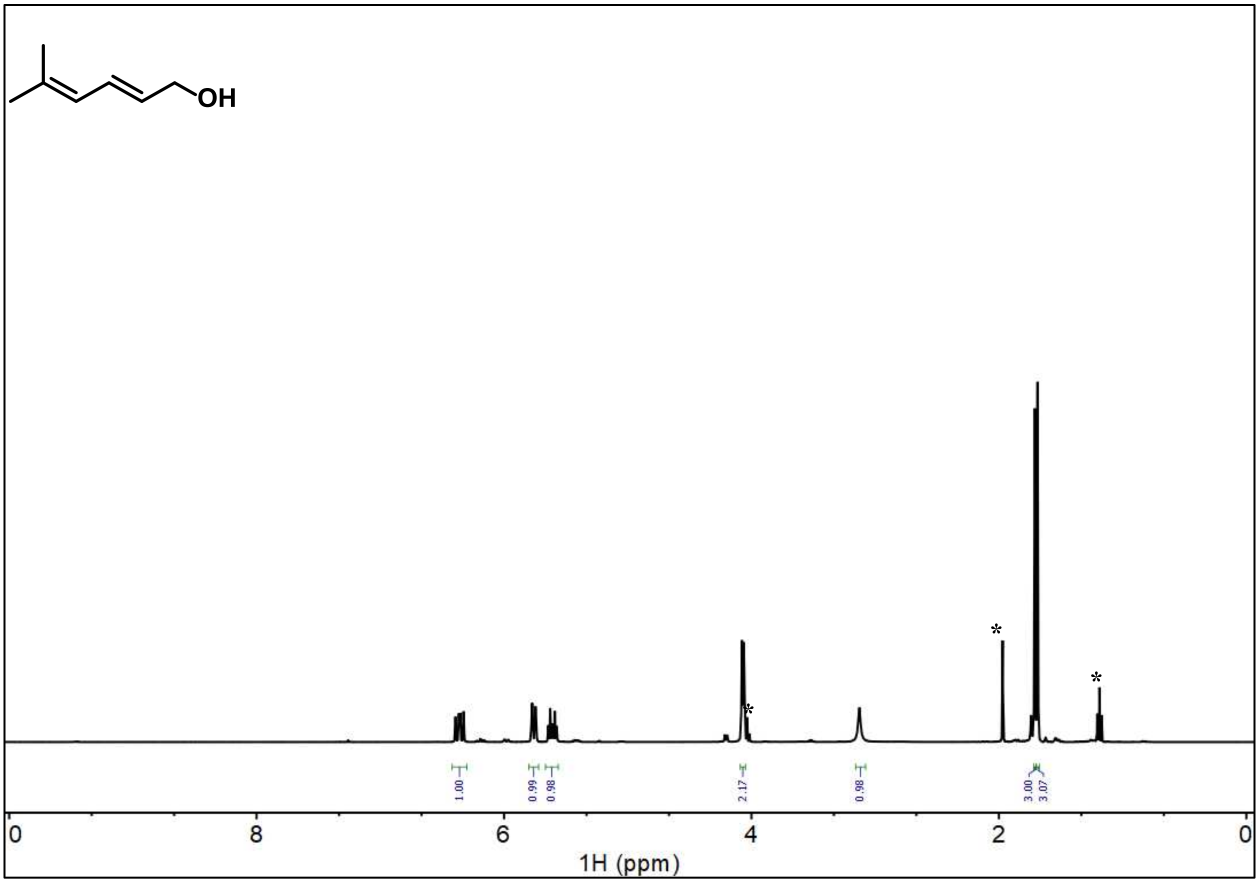

Figure S178. ^{1}H NMR of **(*E*)-5-methylhexa-2,4-dien-1-ol** (400 MHz, $CDCl_3$)**.** [*EtOAc]

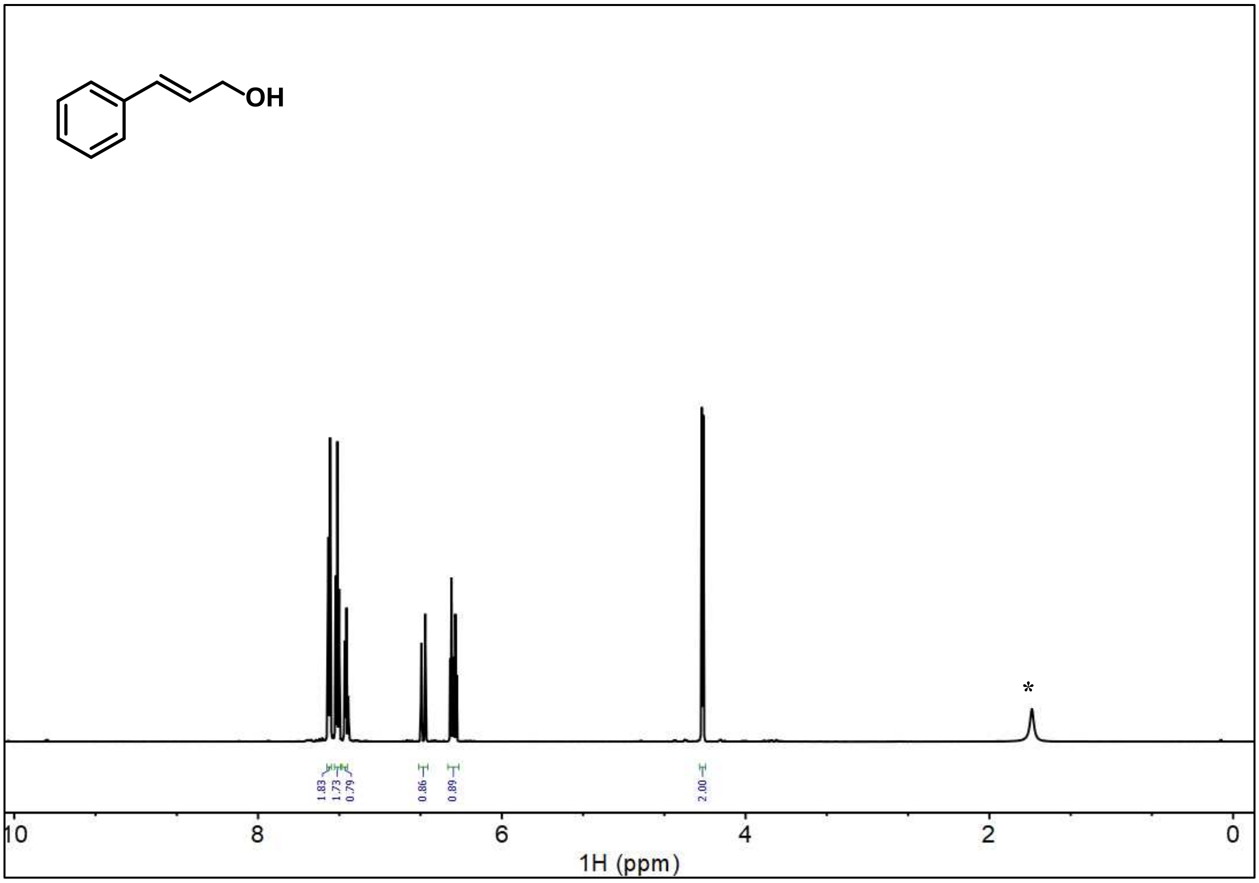

Figure S179. ^{1}H NMR of ***(E)*-3-phenylprop-2-en-1-ol** (500 MHz, $CDCl_3$)**.** [*H_2O]

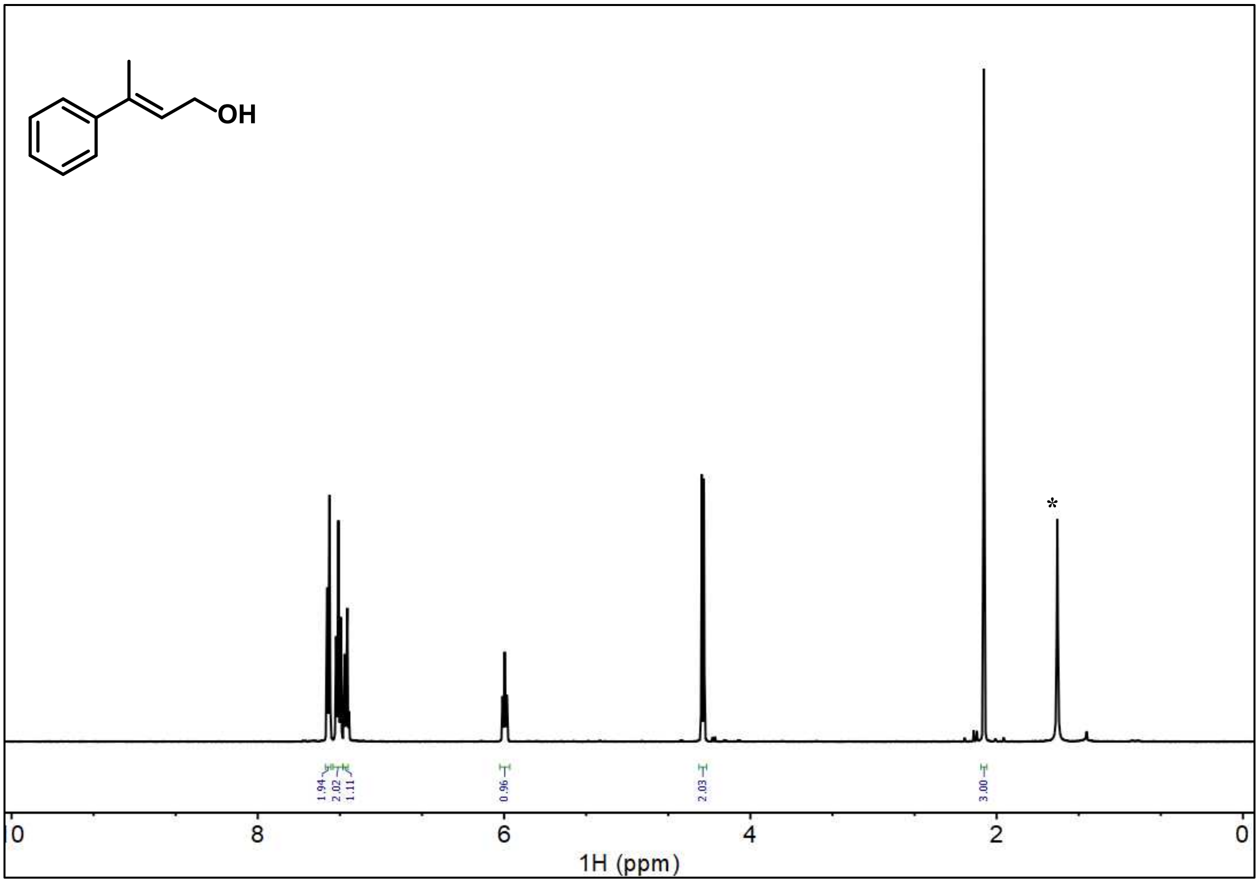

Figure S180. ^{1}H NMR of **(*E*)-3-phenylbut-2-en-1-ol** (400 MHz, $CDCl_3$). [*H_2O]

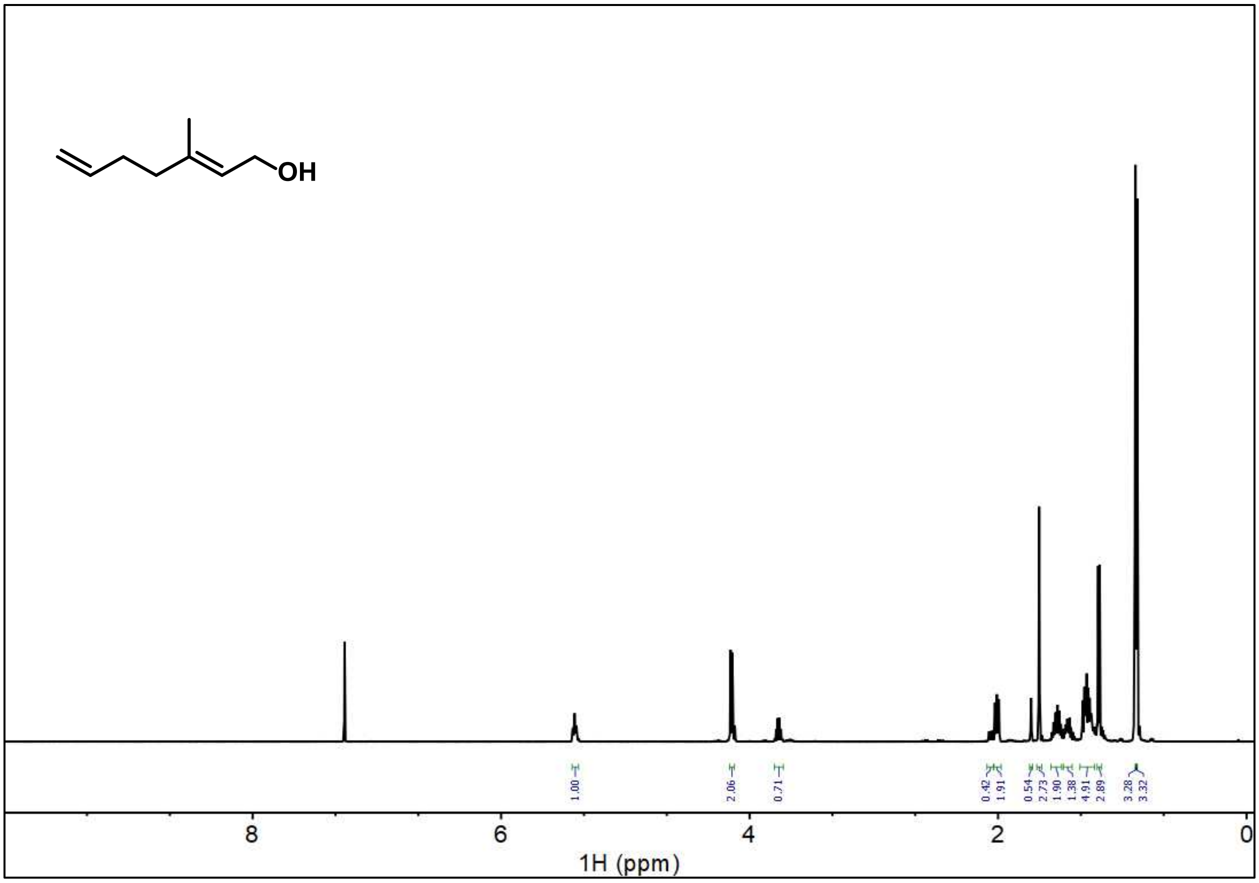

Figure S181. ^{1}H NMR of **(*E*)-3-methylhepta-2,6-dien-1-ol** (500 MHz, $CDCl_3$)**.**

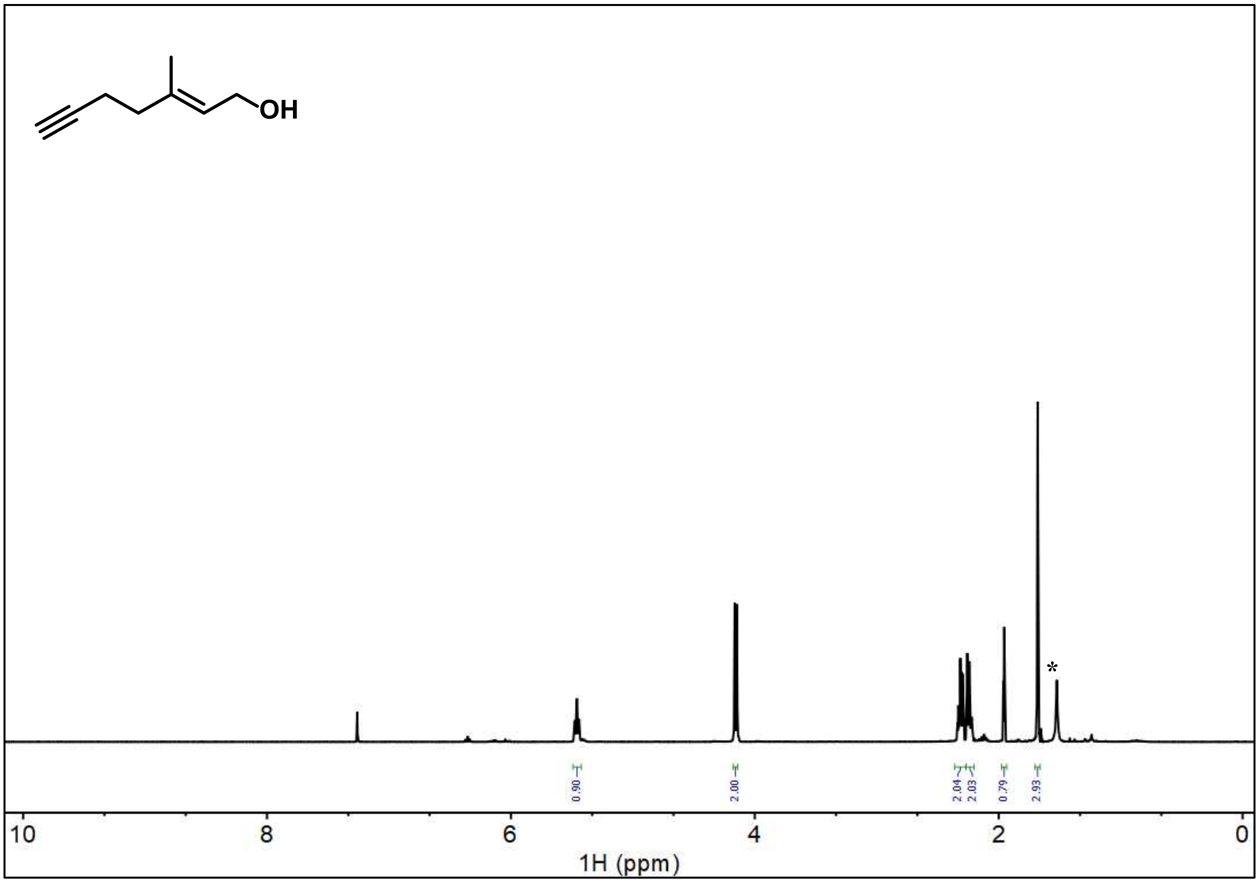

Figure S182. ^{1}H NMR of **(*E*)-3-methylhept-2-en-6-yn-1-ol** (400 MHz, $CDCl_3$)**.** [*H_2O]

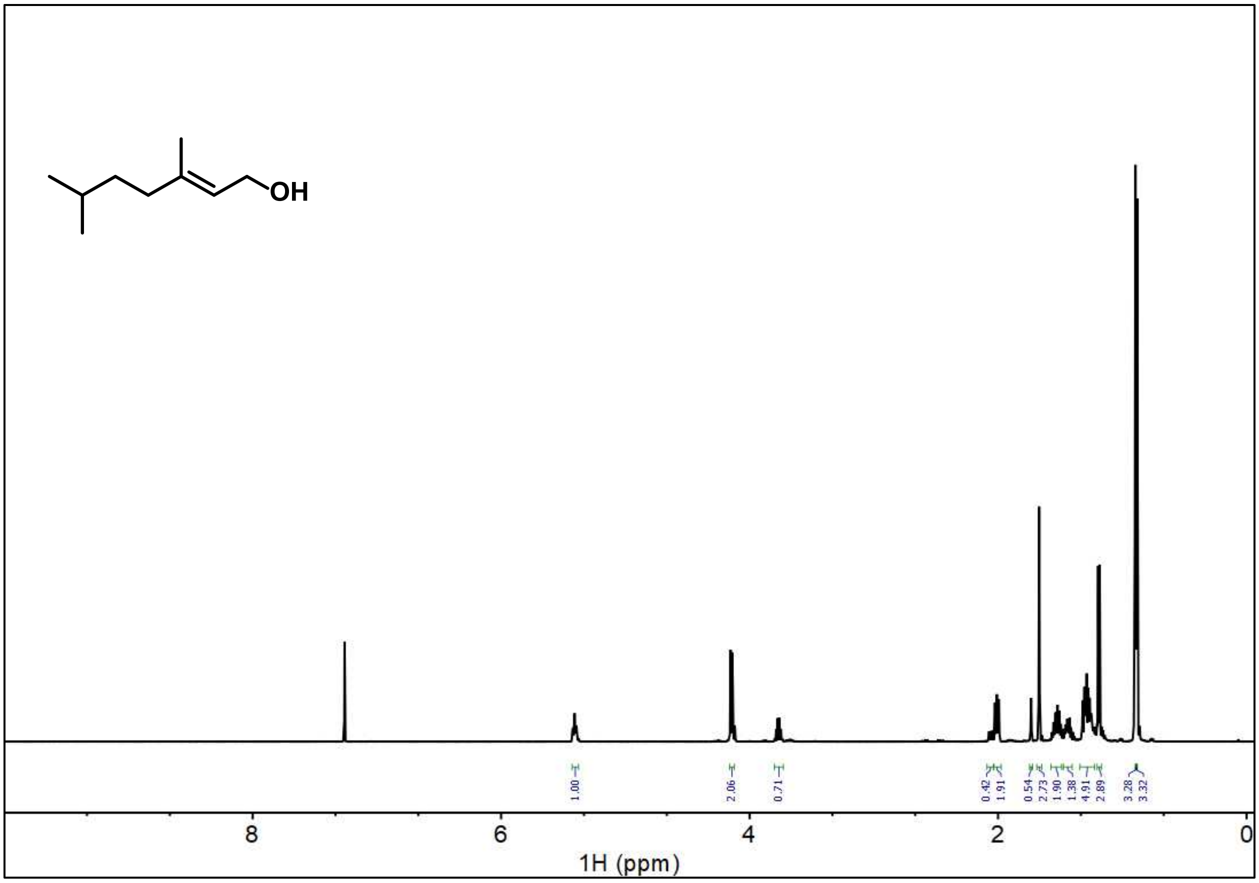

Figure S183. ^{1}H NMR of **(*E*)-3,6-dimethylhept-2-en-1-ol** (500 MHz, $CDCl_3$).

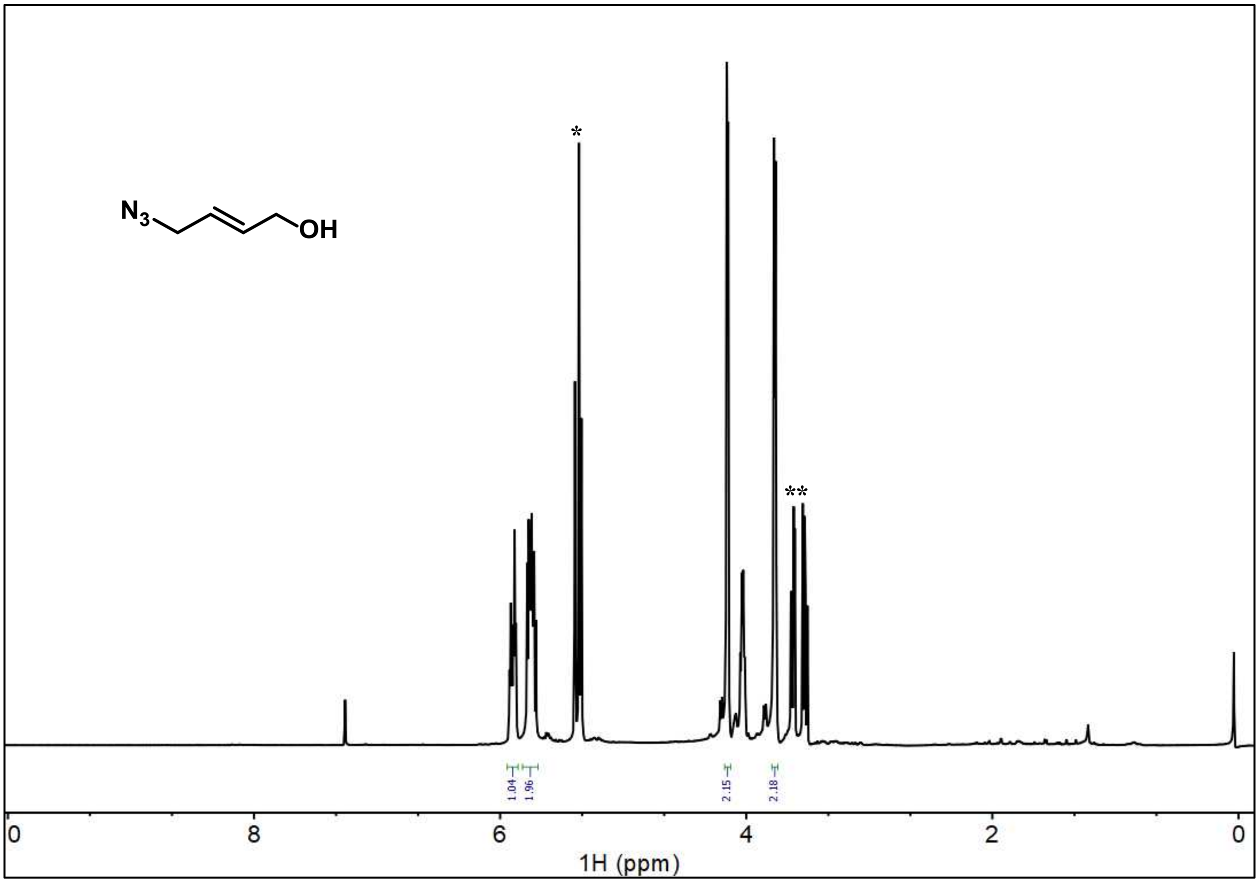

Figure S184. ^{1}H NMR of **(*E*)-4-azidobut-2-en-1-ol** (500 MHz, $CDCl_3$). **[*(*E*)-but-2-ene-1,4-diol]**

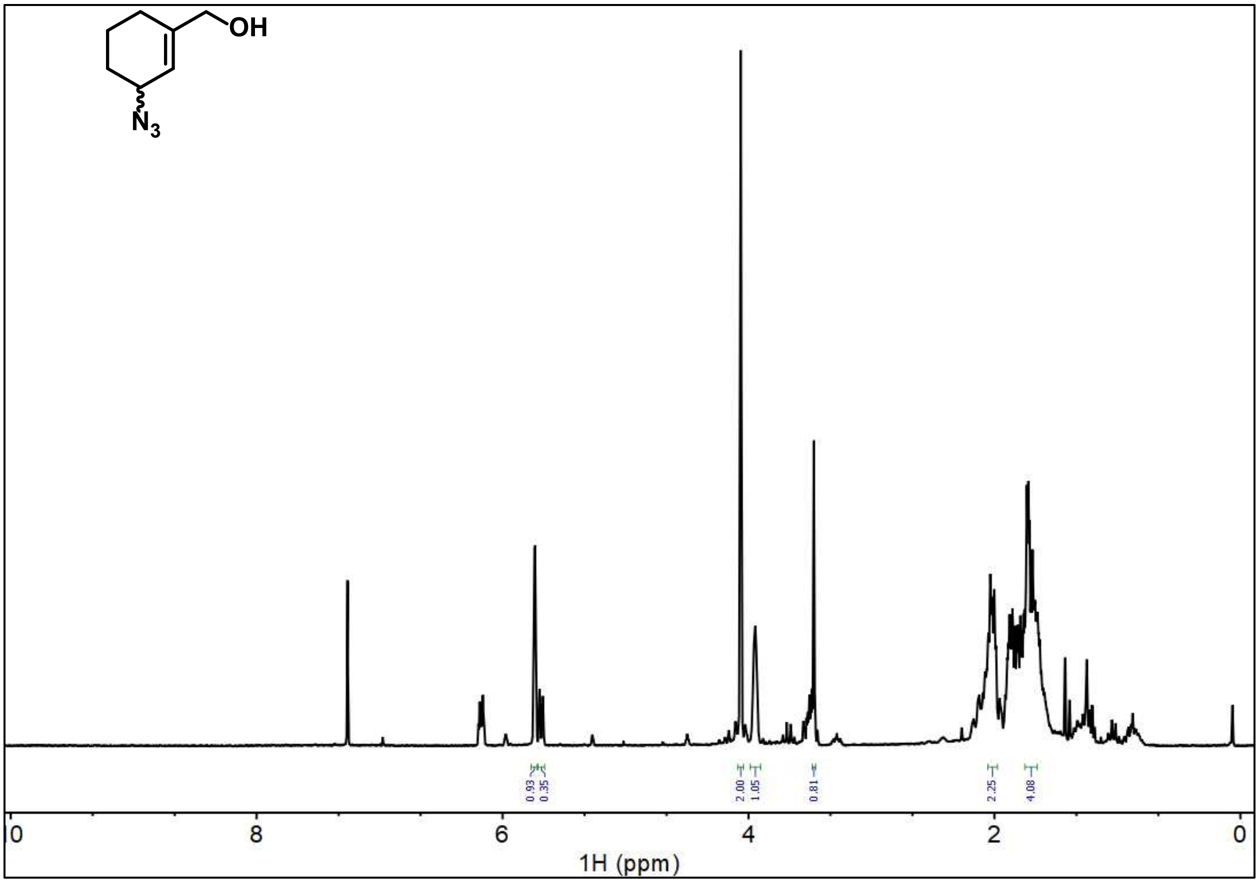

Figure S185. ^{1}H NMR of **(3-azidocyclohex-1-en-1-yl)methanol** (400 MHz, $CDCl_3$).

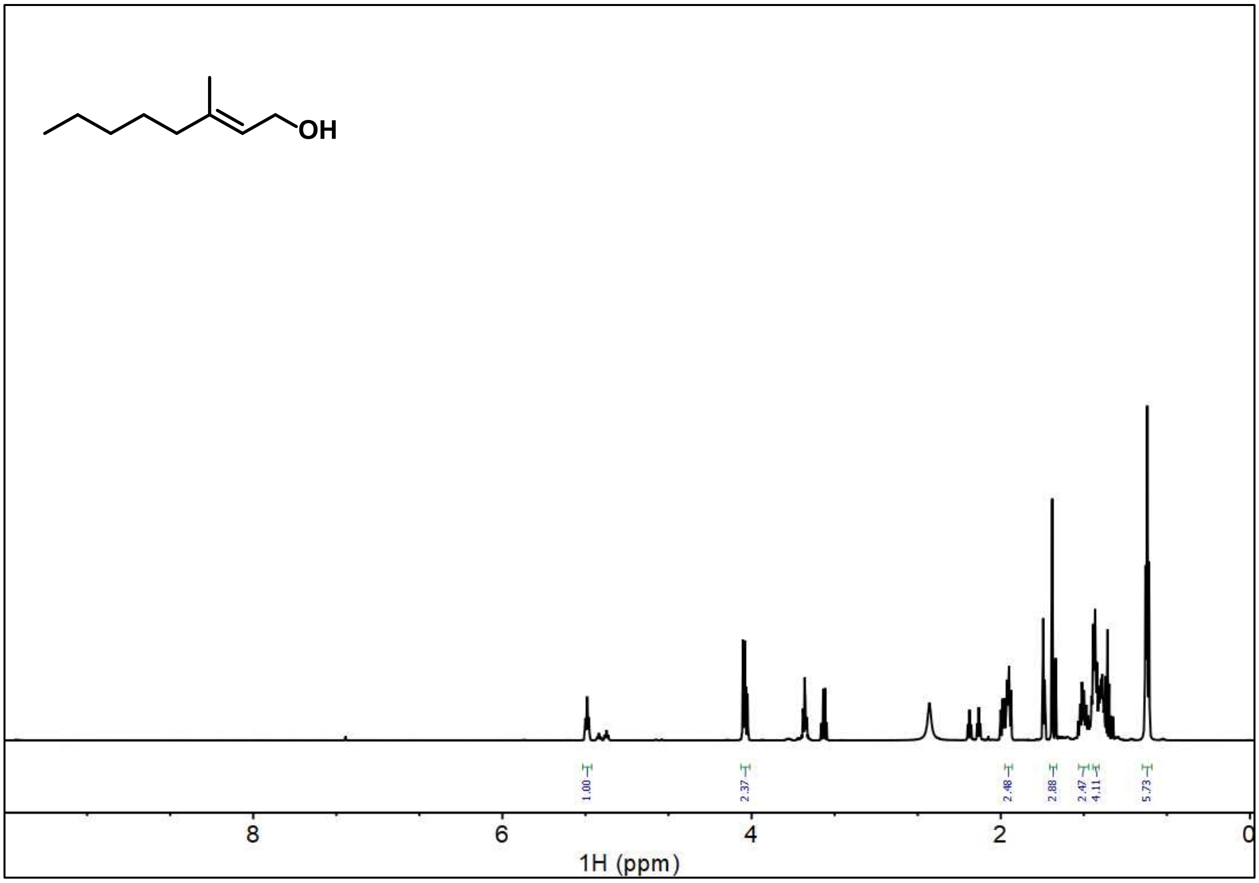

Figure S186. ^{1}H NMR of **(*E*)-3-methyloct-2-en-1-ol** (500 MHz, $CDCl_3$).

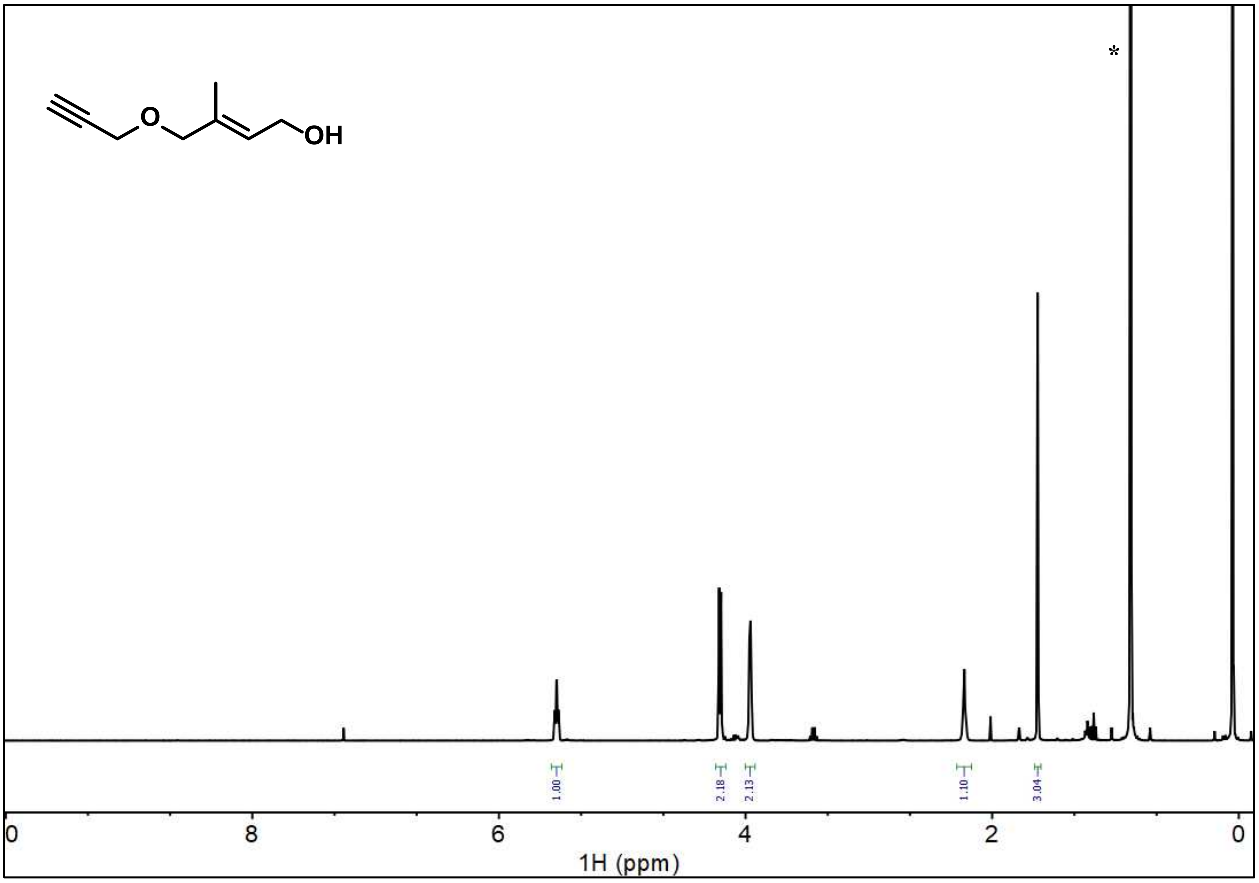

Figure S187. ^{1}H NMR of **(*E*)-5-azido-3-methylpent-2-en-1-ol** (400 MHz, $CDCl_3$)**.** [*H_2O]

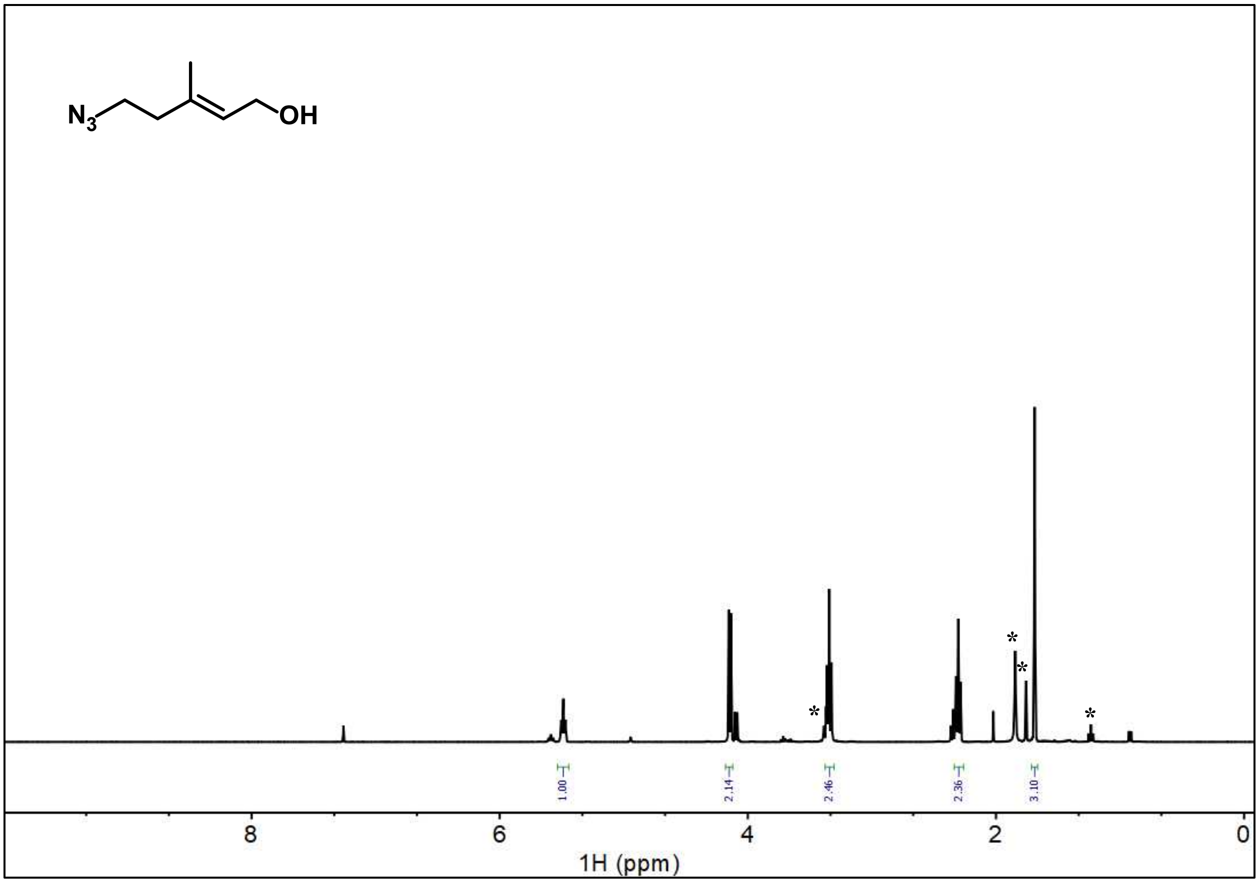

Figure S188. ^{1}H NMR of ***(E)*-5-azido-3-methylpent-2-en-1-ol** (400 MHz, $CDCl_3$). [*EtOAc and H_2O]

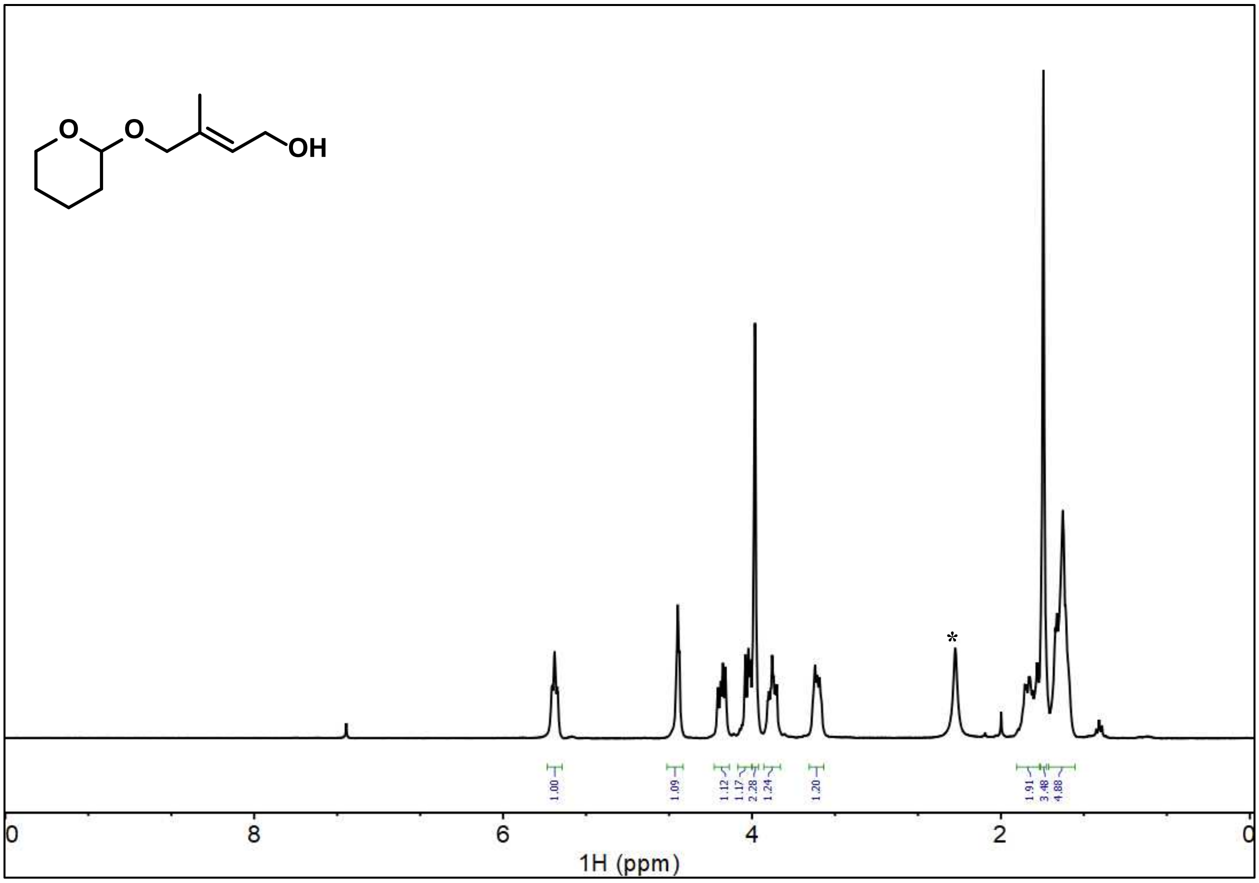

Figure S189. ^{1}H NMR of **(*E*)-3-methyl-4-((tetrahydro-2H-pyran-2-yl)oxy)but-2-en-1-ol** (300 MHz, $CDCl_3$).
[*Acetone]

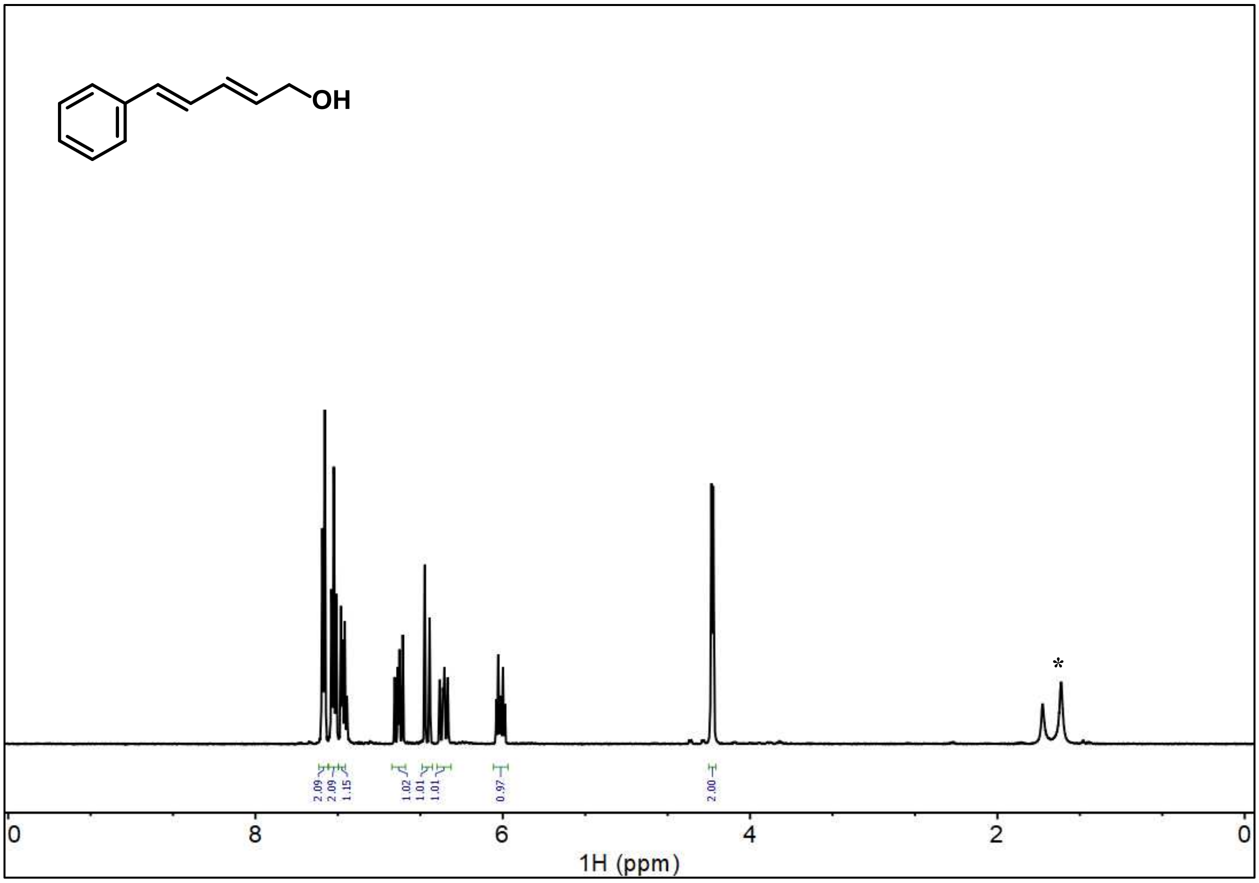

Figure S190. ^{1}H NMR of **(*E*)-4-azidobut-2-en-1-ol** (400 MHz, $CDCl_3$)**.** [*H_2O]

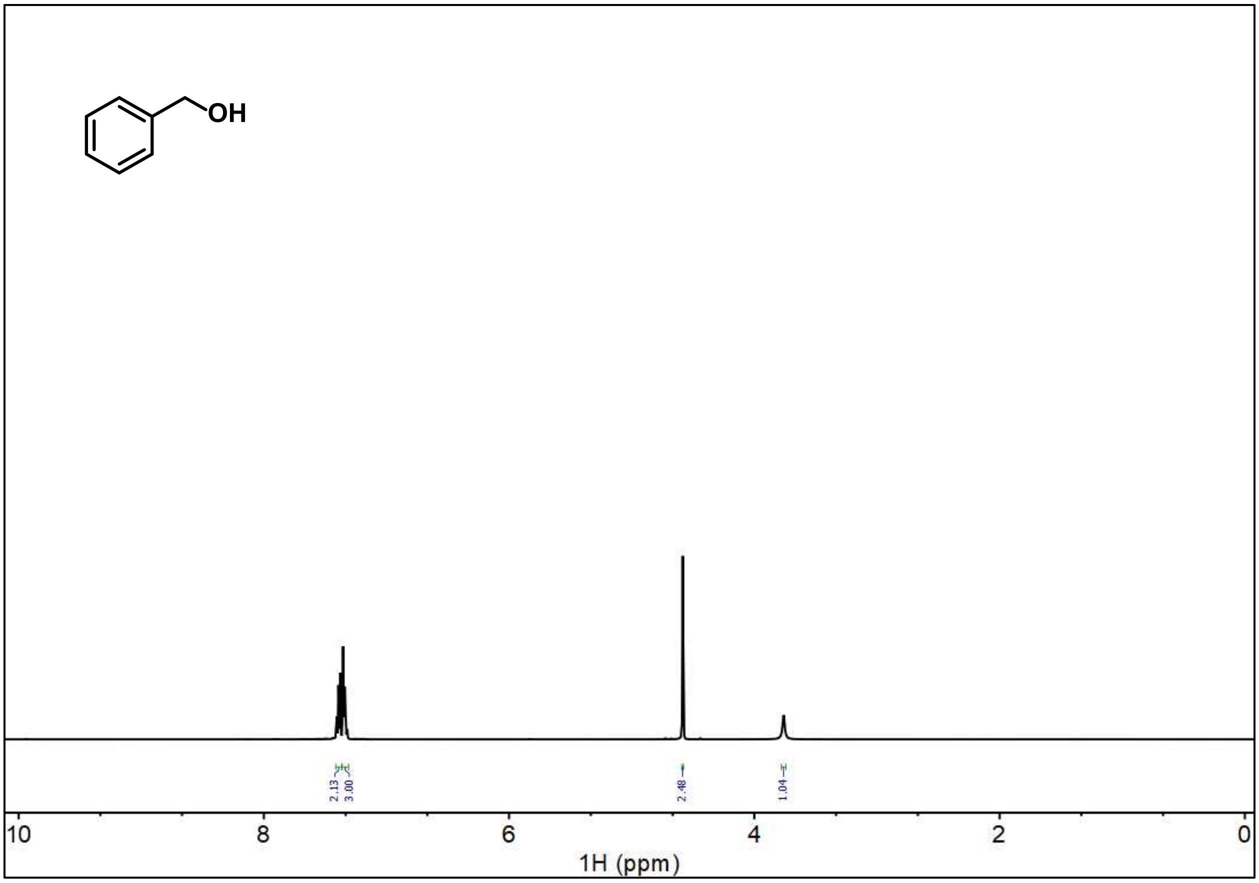

Figure S191. ^{1}H NMR of **phenylmethanol** (500 MHz, $CDCl_3$).

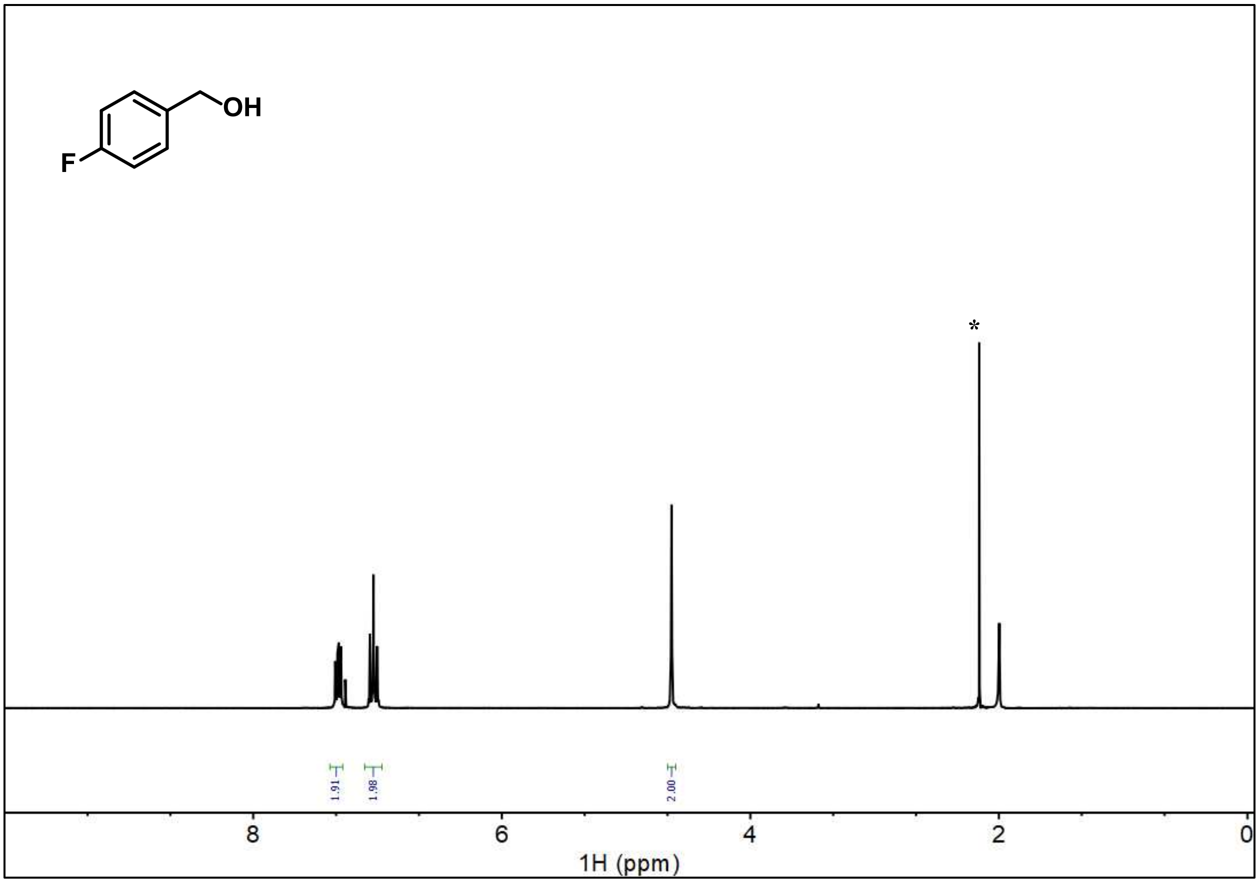

Figure S192. ^{1}H NMR of **(4-fluorophenyl)methanol** (300 MHz, $CDCl_3$)**.** [*Acetone]

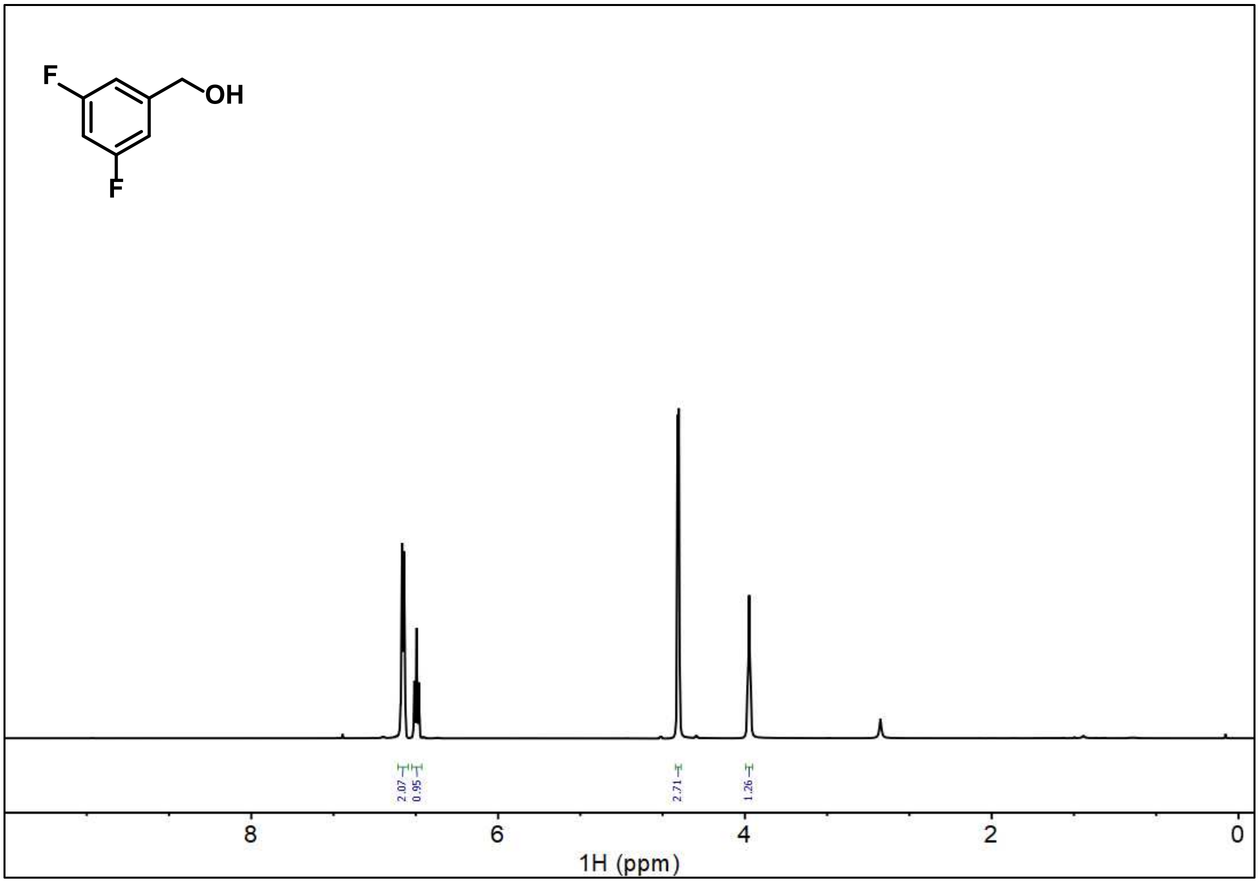

Figure S193. ^{1}H NMR of **(3,5-difluorophenyl)methanol** (500 MHz, $CDCl_3$).

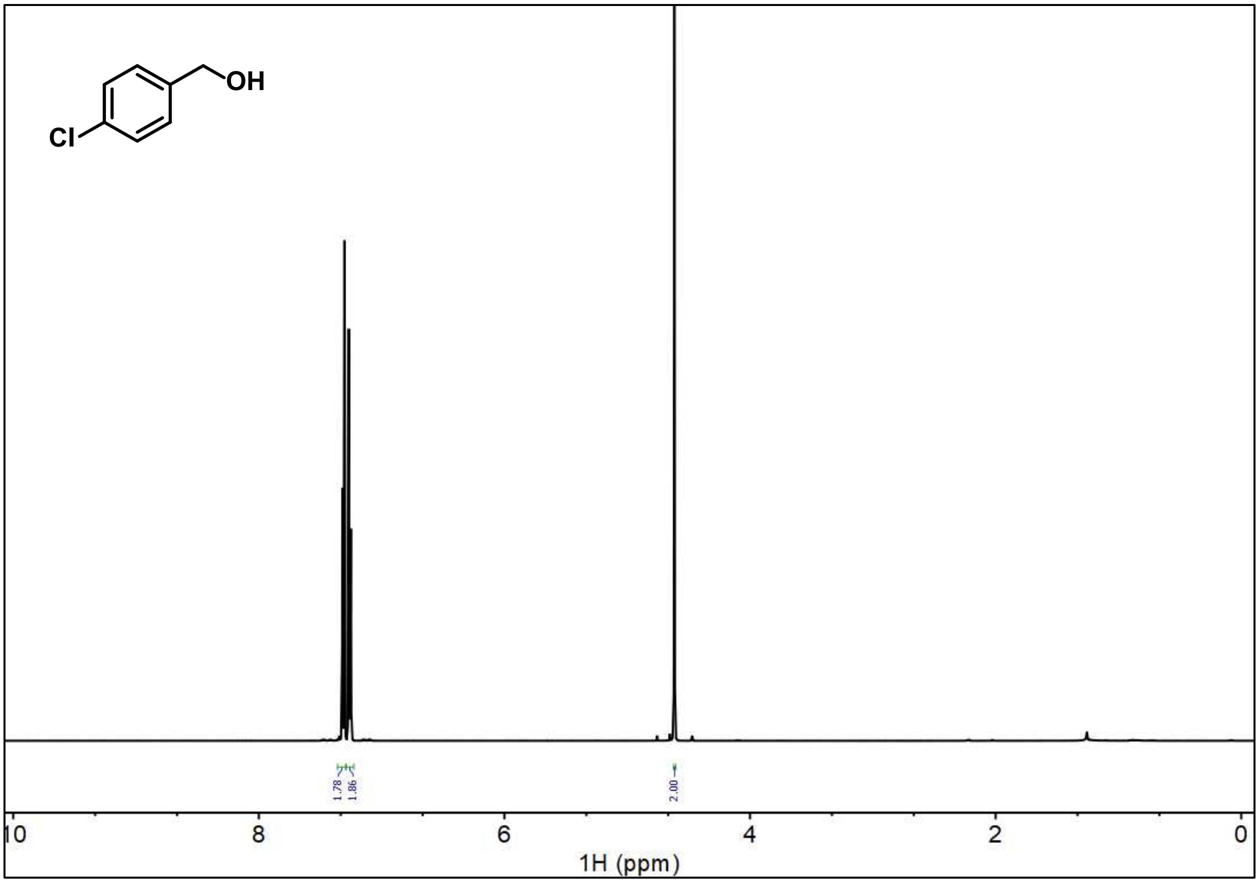

Figure S194. ^{1}H NMR of **(4-chlorophenyl)methanol** (500 MHz, $CDCl_3$).

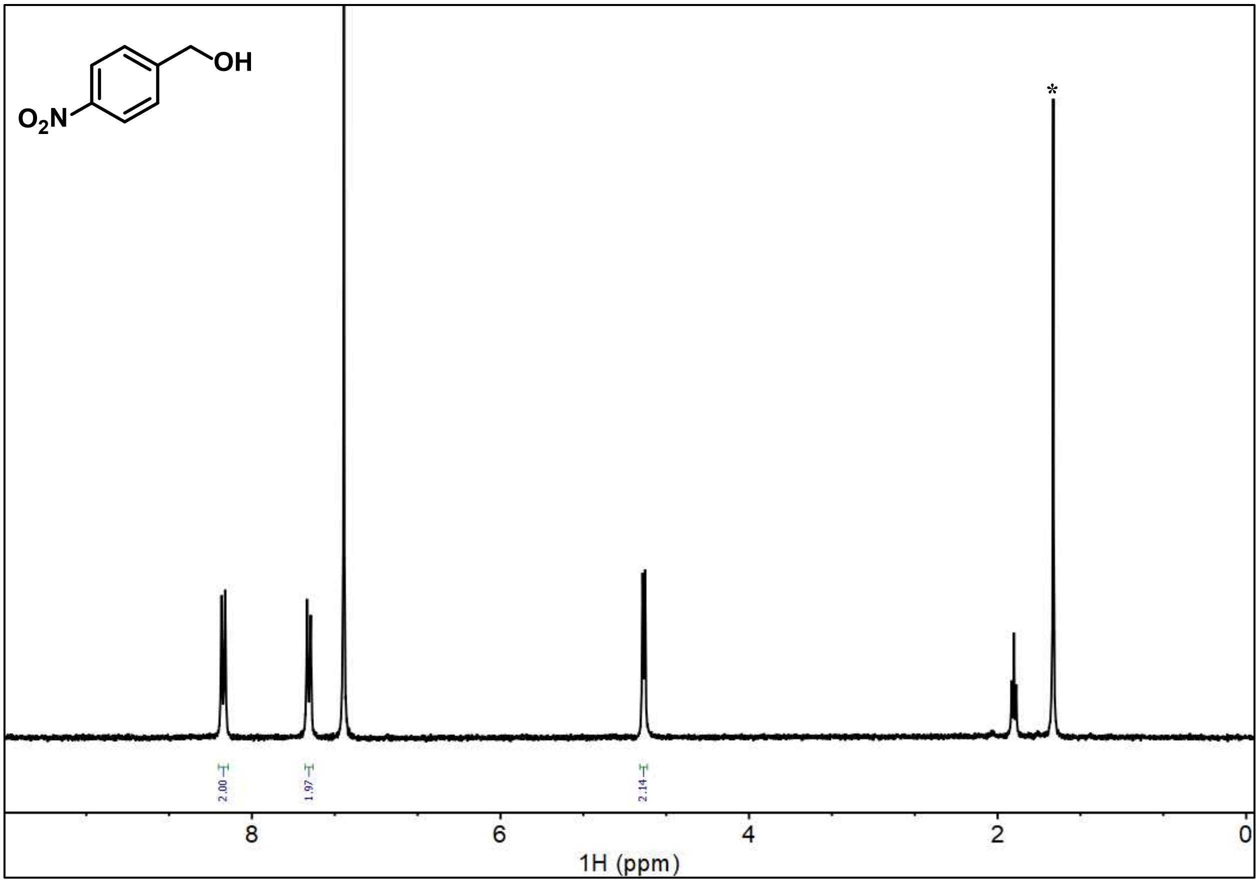

Figure S195. ^{1}H NMR of **(4-nitrophenyl)methanol** (300 MHz, $CDCl_3$)**.** [*H_2O]

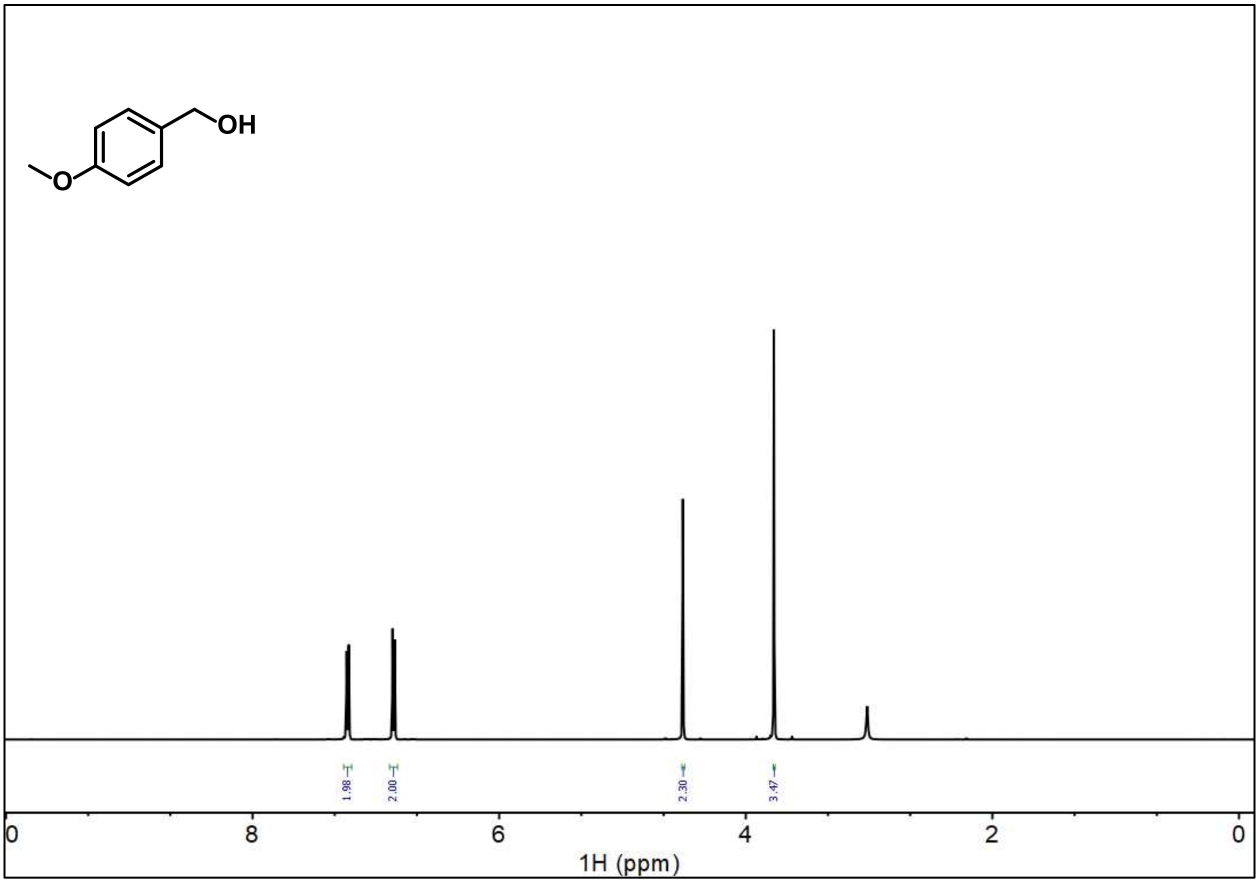

Figure S196. ^{1}H NMR of **(4-methoxyphenyl)methanol** (500 MHz, $CDCl_3$).

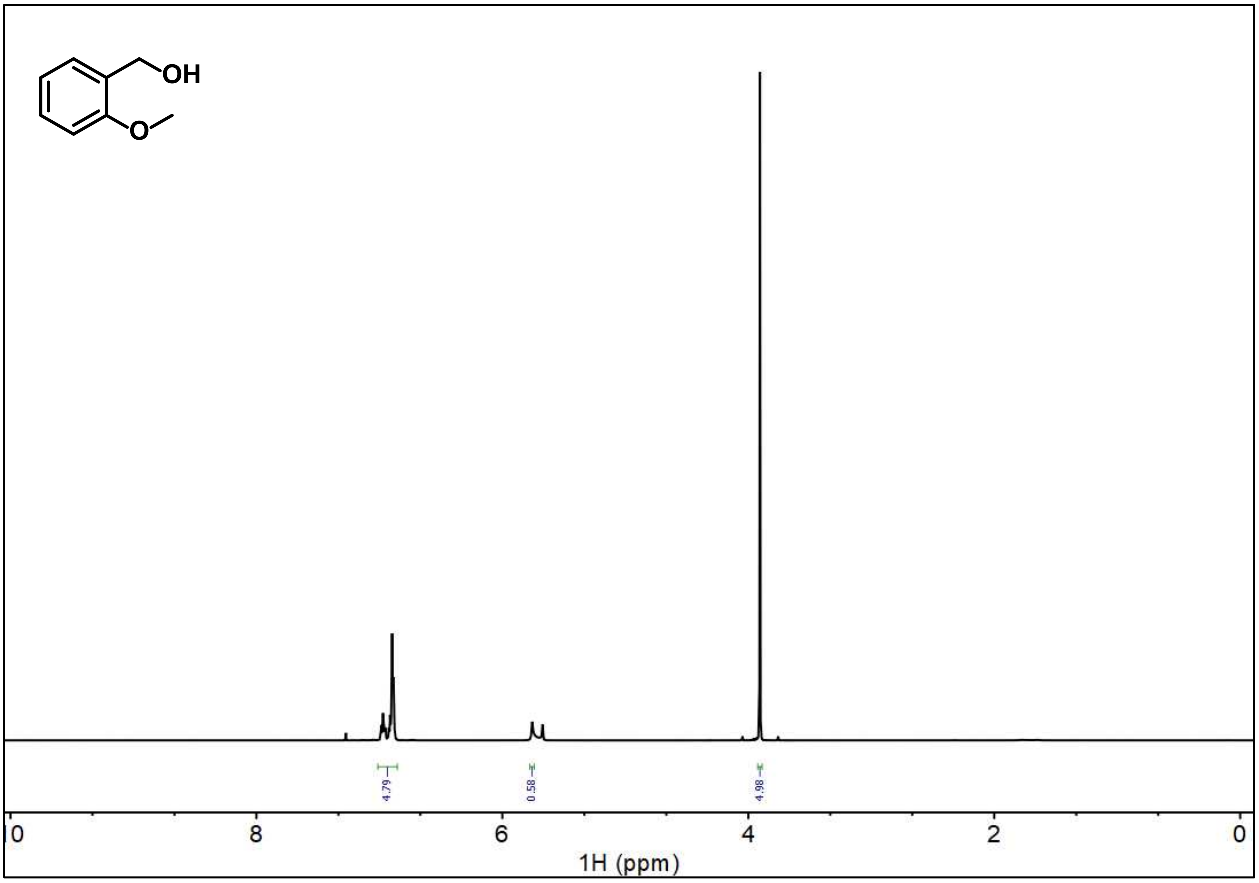

Figure S197. ^{1}H NMR of **(2-methoxyphenyl)methanol** (500 MHz, $CDCl_3$).

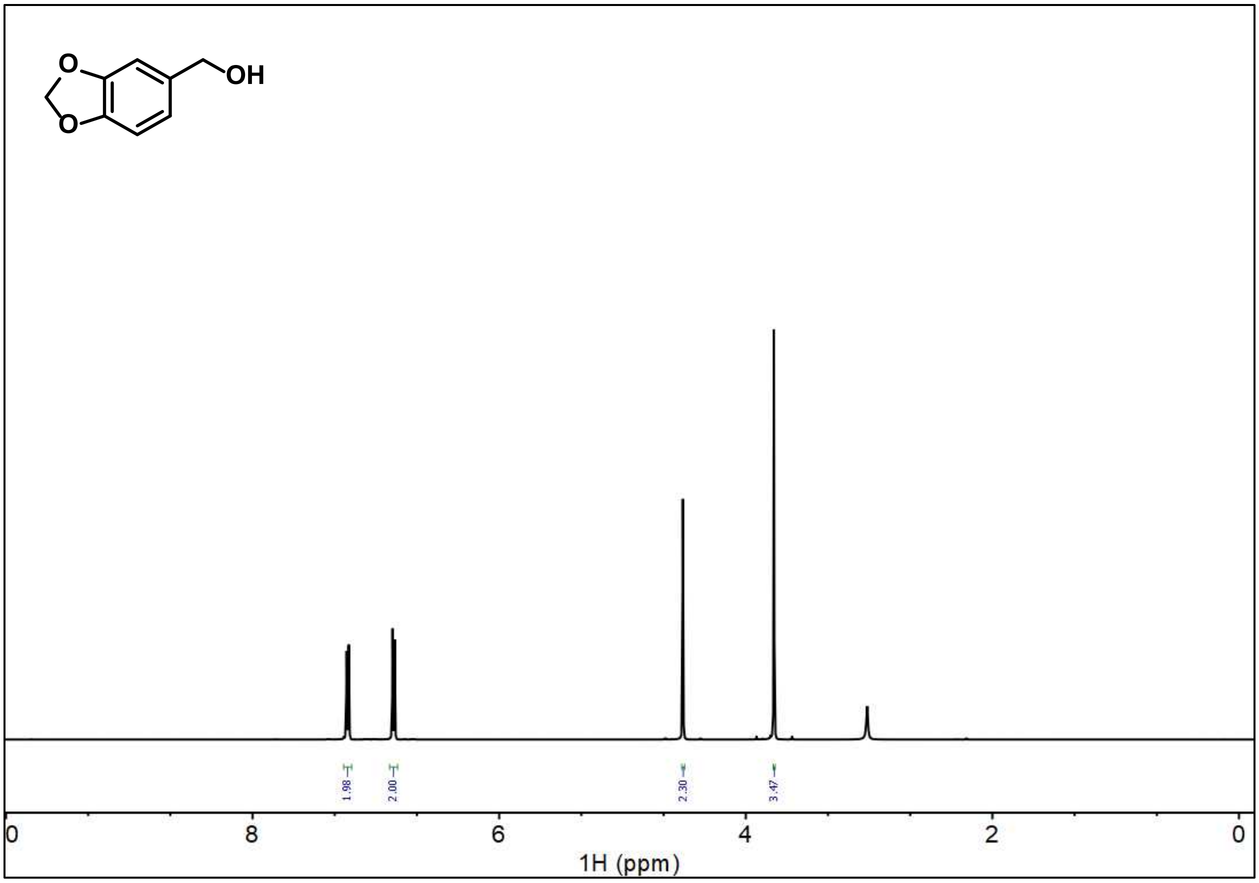

Figure S198. ^{1}H NMR of **benzo[d][1,3]dioxol-5-ylmethanol** (500 MHz, $CDCl_3$).

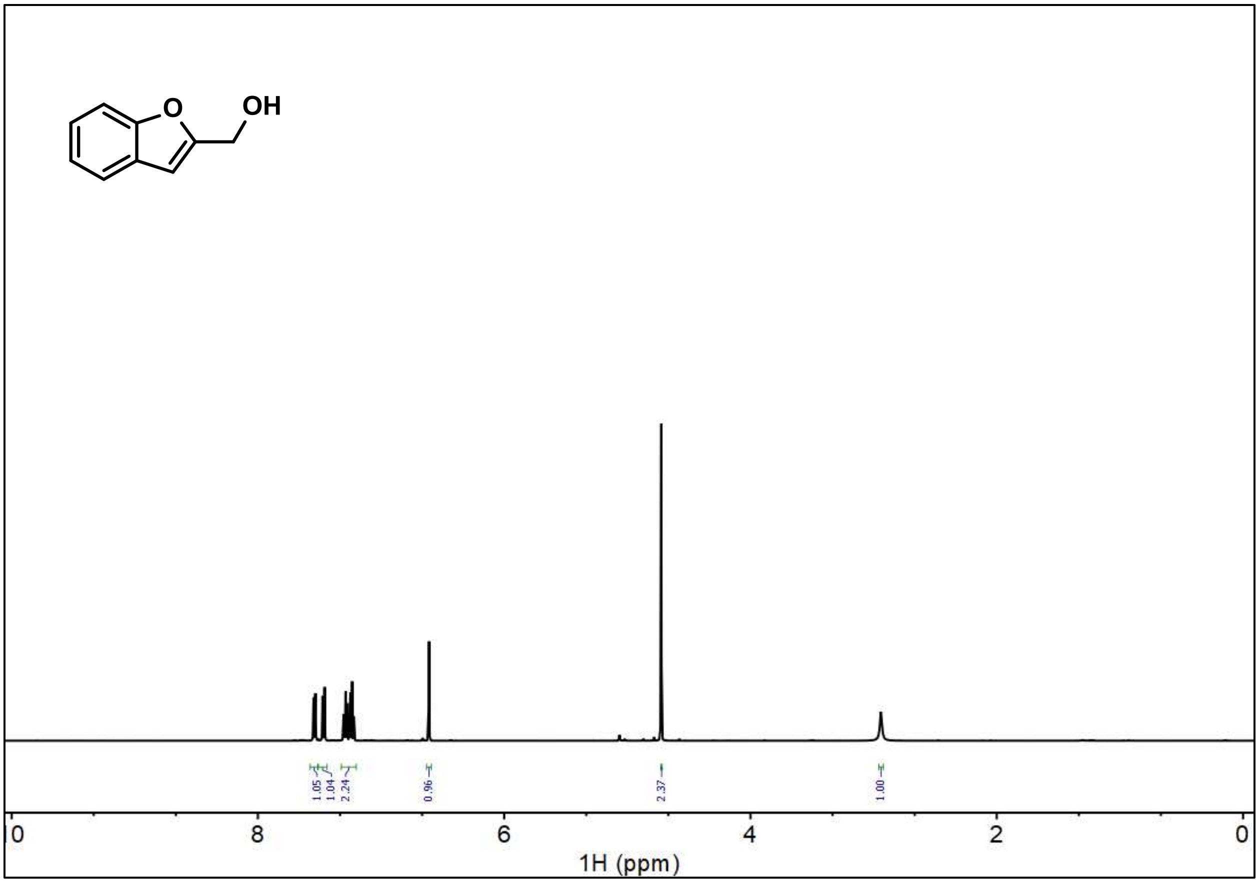

Figure S199. ¹H NMR of **benzofuran-2-ylmethanol** (500 MHz, $CDCl_3$).

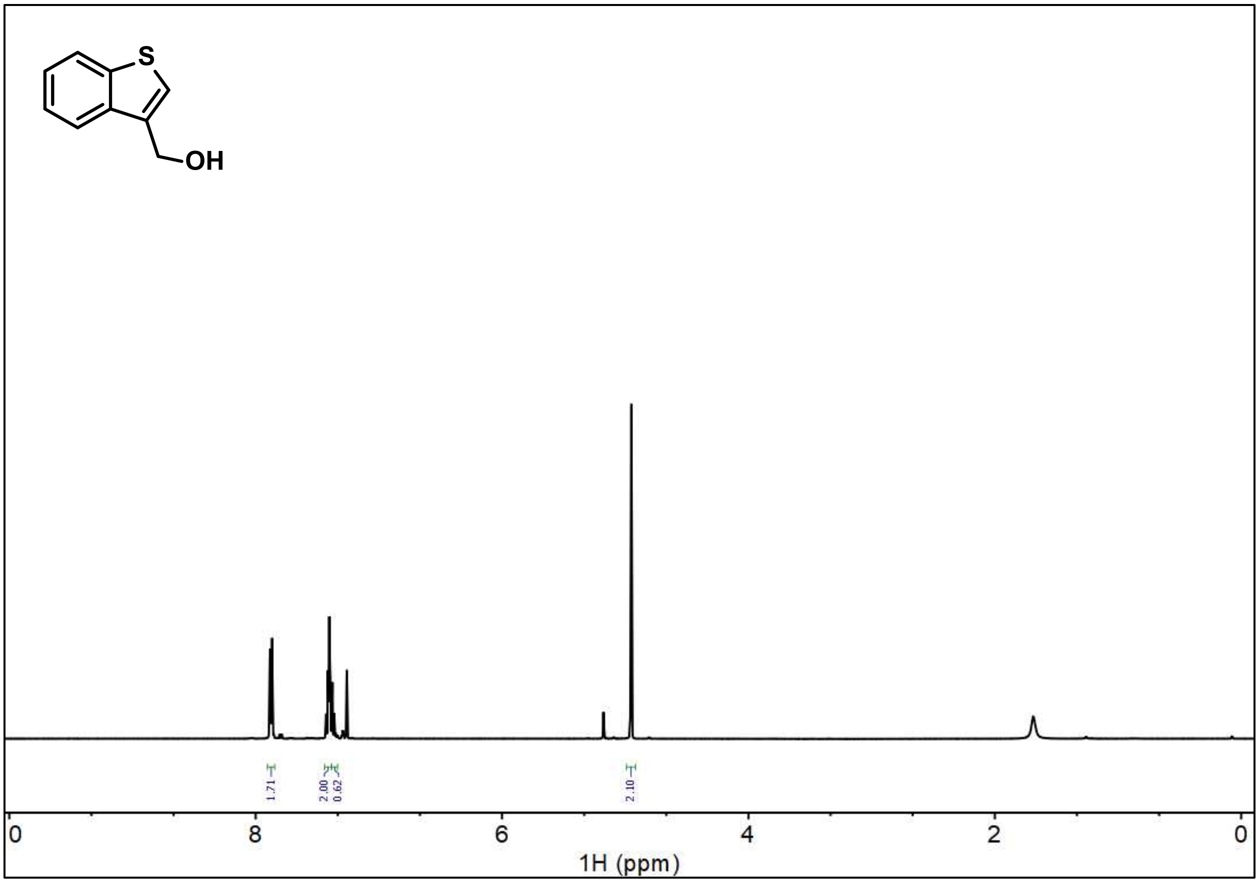

Figure S200. ^{1}H NMR of **benzo[b]thiophen-3-ylmethanol** (500 MHz, $CDCl_3$).

References

[1] a) M. F. Mabanglo, H. L. Schubert, M. Chen, C. P. Hill, C. D. Poulter, *ACS Chem Biol* **2010**, *5*, 517-527; b) N. Dellas, J. P. Noel, *ACS Chem Biol* **2010**, *5*, 589-601.

[2] J. Pei, B. H. Kim, N. V. Grishin, *Nucleic Acids Res* **2008**, *36*, 2295-2300.

[3] M. A. Larkin, G. Blackshields, N. P. Brown, R. Chenna, P. A. McGettigan, H. McWilliam, F. Valentin, I. M. Wallace, A. Wilm, R. Lopez, J. D. Thompson, T. J. Gibson, D. G. Higgins, *Bioinformatics* **2007**, *23*, 2947-2948.
